# Benefits and harms of the human papillomavirus (HPV) vaccines: systematic review with meta-analyses of trial data from clinical study reports

**DOI:** 10.1186/s13643-019-0983-y

**Published:** 2020-02-28

**Authors:** Lars Jørgensen, Peter C. Gøtzsche, Tom Jefferson

**Affiliations:** 1 Nordic Cochrane Centre, Rigshospitalet 7811, Tagensvej 22, 2200 Copenhagen, Denmark; 2 Department of Clinical Medicine, Rigshospitalet, Blegdamsvej 9, 2100 København, Denmark; 3 Institute for Scientific Freedom, Copenhagen, Denmark

**Keywords:** Human papillomavirus vaccine, Systematic review, Meta-analysis, Randomised clinical trial and Clinical study report

## Abstract

**Objective:**

To assess the benefits and harms of the human papillomavirus (HPV) vaccines.

**Data sources:**

Clinical study reports obtained from the European Medicines Agency and GlaxoSmithKline from 2014 to 2017.

**Eligibility criteria:**

Randomised trials that compared an HPV vaccine with a placebo or active comparator in healthy participants of all ages.

**Appraisal and synthesis:**

Two researchers extracted data and judged risk of bias with the Cochrane tool (version 2011). Risk ratio (RR) estimates were pooled using random-effects meta-analysis.

**Outcomes:**

Clinically relevant outcomes in intention to treat populations—including HPV-related cancer precursors irrespective of involved HPV types, treatment procedures and serious and general harms.

**Results:**

Twenty-four of 50 eligible clinical study reports were obtained with 58,412 pages of 22 trials and 2 follow-up studies including 95,670 participants: 79,102 females and 16,568 males age 8–72; 393,194 person-years; and 49 months mean weighted follow-up. We judged all 24 studies to be at high risk of bias. Serious harms were incompletely reported for 72% of participants (68,610/95,670). Nearly all control participants received active comparators (48,289/48,595, 99%). No clinical study report included complete case report forms. At 4 years follow-up, the HPV vaccines reduced HPV-related carcinoma in situ (367 in the HPV vaccine group vs. 490 in the comparator group, RR 0.73 [95% confidence interval, CI, 0.53 to 1.00], number needed to vaccinate [NNV] 387, *P* = 0.05, *I*^2^ = 67%) and HPV-related treatment procedures (1018 vs. 1416, RR 0.71 [95% CI 0.63 to 0.80], NNV 75, *P* < 0.00001, *I*^2^ = 45%). The HPV vaccines increased serious nervous system disorders (exploratory analysis: 72 vs. 46, RR 1.49 [1.02 to 2.16], number needed to harm [NNH] 1325, *P* = 0.040, *I*^2^ = 0%) and general harms (13,248 vs. 12,394, RR 1.07 [95% CI 1.03 to 1.11], NNH 51, *P* = 0.0002, *I*^2^ = 77%) but did not significantly increase fatal harms (45 vs. 38, RR 1.19 [95% CI 0.65 to 2.19], *P* = 0.58, *I*^2^ = 30%) or serious harms (1404 vs. 1357, RR 1.01 [95% CI 0.94 to 1.08], *P* = 0.79, *I*^2^ = 0%).

**Conclusion:**

At 4 years follow-up, the HPV vaccines decreased HPV-related cancer precursors and treatment procedures but increased serious nervous system disorders (exploratory analysis) and general harms. As the included trials were primarily designed to assess benefits and were not adequately designed to assess harms, the extent to which the HPV vaccines’ benefits outweigh their harms is unclear. Limited access to clinical study reports and trial data with case report forms prevented a thorough assessment.

**Systematic review registration:**

CRD42017056093. Our systematic review protocol was registered on PROSPERO in January 2017: https://www.crd.york.ac.uk/PROSPEROFILES/56093_PROTOCOL_20170030.pdf. Two protocol amendments were registered on PROSPERO on November 2017: https://www.crd.york.ac.uk/PROSPEROFILES/56093_PROTOCOL_20171116.pdf. Our index of the HPV vaccine studies was published in Systematic Reviews in January 2018: 10.1186/s13643-018-0675-z. A description of the challenges obtaining the data was published in September 2018: 10.1136/bmj.k3694.

**Electronic supplementary material:**

The online version of this article (10.1186/s13643-019-0983-y) contains supplementary material, which is available to authorized users.

## Introduction

The approved human papillomavirus (HPV) vaccines—GlaxoSmithKline’s Cervarix™ and Merck Sharp and Dohme’s Gardasil™ and Gardasil 9™—are considered safe and effective [1–3]. Recent evidence suggests that the vaccines have significant and long-lasting effects (> 12 years) on cervical cancer [4, 5], better effectiveness when vaccinated below the age of 17 [6], and are possibly able to substantially reduce the global incidence of cervical cancer [7]. However, there are important uncertainties regarding both the benefits and harms of the vaccines.

### Uncertainties of the benefits of the HPV vaccines

The HPV vaccines’ regulatory approvals were mainly based on per-protocol populations and surrogate outcomes of HPV-related lesions, e.g. ‘cervical intraepithelial neoplasia or worse’ (CIN2^+^) infected with an HPV vaccine-specific HPV type, such as HPV types 16 and 18 that are associated with the majority of HPV-related cancers [8–10]. It was considered unfeasible and unethical to use HPV-related cancer as the primary outcome [11, 12], since it takes many years for cancer to develop after an HPV infection and also because cervical screening is an established secondary prevention method that leads to removal of precancerous lesions before they become cancerous. Up to 15% of HPV-related cervical cancers may not contain HPV [13], but HPV may be identified in more cases with newer and more sensitive analysis methods [14]. HPV-related lesions are often infected with more than one HPV type, some of which may not be targeted by the vaccines [15]. This makes it impossible to assess which HPV type caused the lesion. The regulatory vaccine approvals were not based on HPV-related lesions irrespective of HPV type in intention to treat populations, and factors such as antigenic changes and herd immunity may be important in the long-term perspective, as the approved HPV vaccines only target up to 9 of the 25 HPV types considered oncogenic [1].

### Uncertainties of the harms of the HPV vaccines

A Cochrane review from 2018 [3] and most large epidemiological studies [16–20] did not find serious or general harms associated with the HPV vaccines. The Cochrane review was mainly based on journal publications that often are influenced by reporting bias [21–24], and epidemiological studies are influenced by confounding [25].

Acknowledged rare serious harms include anaphylaxis and syncope [8–10]. Some case studies have reported rare neurological harms such as postural orthostatic tachycardia syndrome (POTS) [26, 27] and complex regional pain syndrome (CRPS) [28]. Cluster analyses of individual case safety reports from the World Health Organisation’s (WHOs) VigiBase® revealed additional harms—often serious in nature—that overlapped with the symptomatology of POTS and CRPS [29]. Although the European Medicines Agency’s (EMA) investigation of POTS and CRPS did not find an association with the HPV vaccines [2], EMA’s investigation was based on the HPV vaccine manufacturers’ own assessments [30], and about 30 cases of POTS and CRPS were not recognised in the HPV vaccine manufacturers’ trials [31, 32]. Other reported rare harms have included chronic fatigue syndrome (CFS), Guillain–Barré syndrome (GBS) and premature ovarian failure (POF) [33–35].

### Addressing the uncertainties of the HPV vaccines

To address the uncertainties of the benefits and harms of the HPV vaccines, we conducted a systematic review with meta-analyses of trial data from clinical study reports. As of July 2017, about one third of the HPV vaccine studies had not been published and study results were not posted for about half of the completed studies on ClinicalTrials.gov [36]. Therefore, we based our review on study programmes in order to identify all trials [36] and on clinical study reports [37], as these reports provide vastly more information about a study than a corresponding journal publication [21–24].

## Methods

### Search strategy and study eligibility

**Fig. 1:**
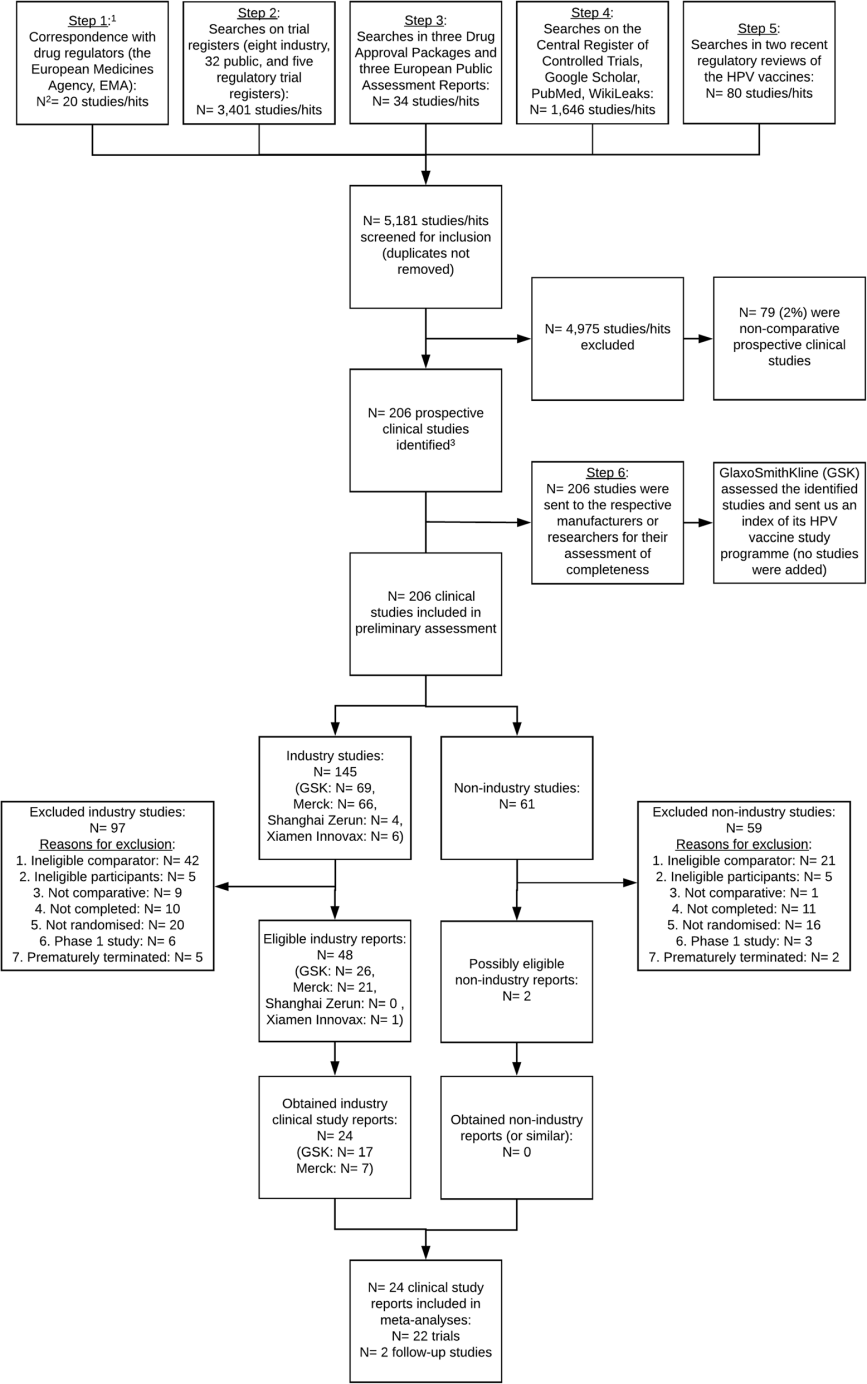
Benefits and harms of the HPV vaccines: flowchart of the inclusion of clinical study reports. For details on the correspondence and searches conducted in steps 1 to 6, see Jørgensen et al. ([36]: Appendices 1 and 2). Two hundred six studies were identified according to our inclusion and classification criteria, see Jørgensen et al. ([36]: Methods). *N* = the number of studies/entries evaluated

Using a six-step process, we constructed and published an index of the HPV vaccine study programmes [36] that included 206 comparative prospective studies (see Fig. 1). Two researchers (LJ and TJ) conducted the six steps that included searches of trial registers, journal publication databases and correspondence with regulators and HPV vaccine manufacturers. It was not feasible to account for duplicate entries, as we indexed studies and searched databases that used different IDs for a unique study (e.g. register ID, study programme ID, manufacturer ID and publication ID) [36].

In May 2014, we requested the study programmes’ corresponding clinical study reports from the European Medicines Agency (EMA; via its policy 0043) and obtained those reports that were freely available on GlaxoSmithKline’s online trial register. We did not request clinical study reports from the manufacturers, as this would limit our ability to use and share the data [38]. In January 2017, we registered our systematic review protocol in PROSPERO (International prospective register of systematic reviews): CRD42017056093 [37].

We included those trials and their follow-up studies of the 206 comparative studies from our index that were randomised clinical phase II, III or IV trials. We aimed to include studies for which we obtained industry clinical study reports or similar non-industry reports. In the event of no clinical study report being available (for an otherwise eligible trial), we did not include data from the trial publication. We also aimed to include periodical safety update reports. PICO criteria (participants, interventions, comparisons and outcomes) were used to select trials that compared an HPV vaccine with a placebo (normal saline) or active comparator (adjuvant or non-HPV vaccine such as a hepatitis vaccine) in healthy participants (see Additional file 1 for our PRISMA checklist).

### Data extraction and risk of bias assessment

One researcher performed trial selection and data extraction (LJ); a second researcher (TJ) checked the selection and extraction; a third researcher (PCG) arbitrated. Cochrane’s tool (version 2011) was used for risk of bias assessments [25].

### Outcome assessment

We assessed the following primary outcomes: all-cause mortality, deaths from and incidence of HPV-related cancers, incidence of histologically confirmed carcinoma in situ and moderate intraepithelial neoplasia, fatal harms, serious harms and harms of special interest (anaphylaxis, chronic fatigue syndrome [CFS], complex regional pain syndrome [CRPS], Guillain-Barré syndrome [GBS], postural orthostatic tachycardia syndrome [POTS], premature ovarian failure [POF] and syncope). Histological outcomes were assessed irrespective of which HPV types were involved.

Secondary outcomes included HPV-related external genital lesions and referral procedures, new onset diseases (reported in the included clinical study reports as ‘medically significant conditions’ and ‘new medical history’) and general harms (reported as 'solicited', ‘unsolicited’ and ‘systemic adverse events’). We did not consider cytological, serological or virological outcomes or local harms due to their lower clinical importance.

The clinical study reports included over 3000 different types of harms that were classified with MedDRA (Medical Dictionary for Regulatory Activities) preferred terms. The harms were often incompletely and heterogeneously reported (see Table 1). We extracted and assessed all individual harms classified with MedDRA-preferred terms. We performed meta-analyses for the five most commonly occurring fatal and serious harms, the five fatal and serious harms that the HPV vaccines increased the most and the five fatal and serious harms that the HPV vaccines decreased the most. For new onset diseases and general harms, we performed meta-analyses for the three most common, increased and decreased harms for each category (‘medically significant conditions’ and ‘new medical history’; and ‘solicited’, ‘unsolicited’ and ‘systemic adverse events’). MedDRA-preferred terms and total harms were reported as the number of participants with one or more harms over the total number of participants.

**Table 1:** Benefits and harms of the HPV vaccines: reporting of harms in included HPV vaccine studies

Reporting of harms^a^	Total		GlaxoSmithKline		Merck Sharp and Dohme	
	Studies (*N* = 24)	Participants (*N* = 96,855)^a^	Studies (*N* = 17)	Participants (*N* = 66,235)^a^	Studies (*N* = 7)	Participants (*N* = 30,620)
Fatal harms						
Reported for the whole study period	23	64,679 (67%)	16	34,059 (51%)	7	30,620 (100%)
Reported for the whole study period for some^b^ participants	1	32,176 (33%)	1	32,176 (49%)	0	0 (0%)
Serious harms^c^						
Reported for the whole study period	14	28,245 (30%)	14	28,245 (42%)	0	0 (0%)
No breakdown into MedDRA-preferred terms	3 (21%)	2586 (9%)	3 (21%)	2586 (9%)	0 (0%)	0 (0%)
Reported 0 to 14 days post-vaccination	7	30,620 (31%)	0	0 (0%)	7	30,620 (100%)
Reported for the 7-month vaccination period	2	5814 (6%)	2	5814 (9%)	0	0 (0%)
Reported for a subset or the serious harms judged vaccine-related by the trial investigators^b^	1	32,176 (33%)	1	32,176 (49%)	0	0 (0%)
New onset diseases^d^						
Reported as ‘medically significant conditions’ for the whole study period	15	65,741 (68%)	15	65,741 (99%)	0	0 (0%)
No breakdown into MedDRA-preferred terms	2 (13%)	33,216 (51%)	2 (13%)	33,216 (51%)	0 (0%)	0 (0%)
Reported as ‘new medical history’ for the whole study period	7	30,620 (31%)	0	0 (0%)	7	30,620 (100%)
Not reported/included in clinical study report	2	494 (1%)	2	494 (1%)	0	0 (0%)
General harms^e^						
Reported as ‘solicited’ and ‘unsolicited’ general harms 7 and 30 days post-vaccination	14	64,010 (66%)	14	64,010 (96%)	0	0 (0%)
Reported for a subset of participants^f^	2 (14%)	7791/50,820	2 (14%)	7791/50,820	0 (0%)	0 (0%)
Reported as ‘systemic adverse events’ 14 days post-vaccination	7	30,620 (31%)	0	0 (0%)	7	30,620 (100%)
No breakdown into MedDRA-preferred terms	3 (43%)	21,441 (70%)	0 (0%)	0 (0%)	3 (43%)	21,441 (70%)
Not reported/included in clinical study report	3	2225 (3%)	3	2225 (4%)	0	0 (0%)

^a^See Additional file 2 for details on the reporting of harms. Table 1 includes all 24 clinical study reports including the two follow-up studies HPV-023 (follow-up for trial HPV-001) of 433 participants and HPV-063 (follow-up for trial HPV-032) of 752 participants, i.e. 1185 participants (433 + 752) are included twice for the trials HPV-001 and HPV-032. The total denominator is 95,670 for the 22 included trials and 96,855 (95,670 + 1185) for the 24 included clinical study reports

^b^In one trial (HPV-040), 12% (3703/32,176) of participants were included in a subset population for fatal and serious harms reporting

^c^(1) GlaxoSmithKline defined serious harms as “any untoward medical occurrence that a resulted in death and b was life-threatening, NOTE: The term ‘life-threatening’ in the definition of ‘serious’ refers to an event in which the subject was at risk of death at the time of the event. It did not refer to an event, which hypothetically might have caused death, if it were more severe. c. required hospitalisation or prolongation of existing hospitalisation, NOTE: In general, hospitalisation signified that the subject had been detained (usually involving at least an overnight stay) at the hospital or emergency ward for observation and/or treatment that would not have been appropriate in the physician’s office or out-patient setting. Complications that occurred during hospitalisation were AEs [adverse events]. If a complication prolonged hospitalisation or fulfilled any other serious criteria, the event was serious. When in doubt as to whether “hospitalisation” occurred or was necessary, the AE was to be considered serious. Hospitalisation for elective treatment of a pre-existing condition that did not worsen from baseline was not considered an AE. d. resulted in disability/incapacity, NOTE: The term disability means a substantial disruption of a person’s ability to conduct normal life functions. This definition was not intended to include experiences of relatively minor medical significance such as uncomplicated headache, nausea, vomiting, diarrhoea, influenza, and accidental trauma (e.g. sprained ankle) which may interfere or prevent everyday life functions but did not constitute a substantial disruption. e. was a congenital anomaly/birth defect in the offspring of a study subject”. (2) Merck Sharp and Dohme defined serious harms as “any adverse experience occurring at any dose that: Results in death; or that is life threatening (places the subject/patient, in the view of the investigator, at immediate risk of death from the experience as it occurred. [Note: This does not include an adverse experience that, had it occurred in a more severe form, might have caused death.]); or that results in a persistent or significant disability/incapacity (substantial disruption of one’s ability to conduct normal life functions); or that results in or prolongs an existing inpatient hospitalisation (hospitalised is defined as an inpatient admission, regardless of length of stay, even if the hospitalisation is a precautionary measure for continued observation.); or ALSO: Other important medical events that may not result in death, not be life threatening, or not require hospitalisation may be considered a serious adverse experience when, based upon appropriate medical judgement, the event may jeopardise the subject/patient and may require medical or surgical intervention to prevent one of the outcomes listed above”

^d^(1) GlaxoSmithKline defined ‘medically significant conditions’ as “Adverse events prompting emergency room or physician visits that are not (1) related to common diseases or (2) routine visits for physical examination or vaccination, or SAEs [serious adverse events] that are not related to common diseases. Serious adverse events related to common diseases were reported but are not classified as medically significant conditions for analysis purposes. Common diseases include: upper respiratory infections, sinusitis, pharyngitis, gastroenteritis, urinary tract infections, cervicovaginal yeast infections, menstrual cycle abnormalities and injury”. (2) Merck Sharp and Dohme did not provide a formal definition for ‘new medical history’ but described ‘new medical history’ as “all new reported diagnoses” in the clinical study report of trial V501-019

^e^(1) GlaxoSmithKline defined ‘solicited’ general adverse events as “Adverse events to be recorded as endpoints in the clinical study [i.e. arthralgia, fatigue, headache, myalgia, pyrexia, rash and urticaria]. The presence/occurrence/intensity of these events is actively solicited from the subject or an observer during a specified post-vaccination follow-up period”. (2) GlaxoSmithKline defined ‘unsolicited’ general adverse event as “Any AE [adverse event] reported in addition to those solicited during the clinical study. Also, any “solicited” symptom with onset outside the specified period of follow-up for solicited symptoms was reported as an unsolicited AE”. (3) Merck Sharp and Dohme defined ‘systemic adverse event’ as “any systemic clinical adverse event that developed on the day of vaccination or during the 14 days after vaccination was recorded on the VRC [vaccination report card] along with the date it started and the last date it was present”

^f^The two trials HPV-008 and HPV-040 only reported general harms for 15% (7791/50,820) of included participants

To check for possible harm clustering on an organ system level, we meta-analysed the MedDRA-preferred terms in their respective system organ classes (for example, the MedDRA-preferred terms ‘dizziness’, ‘pain’ and ‘syncope’ were part of and therefore included in the MedDRA system organ class ‘nervous system disorders’). Only Merck clinical study reports included aggregate numbers for participants with MedDRA system organ class harms, and only for new onset diseases (‘new medical history’) and general harms (‘systemic adverse events’). For all GlaxoSmithKline clinical study reports and for serious harms for Merck clinical study reports, we pooled MedDRA-preferred terms in their respective system organ classes. A participant could potentially be included more than once in a separate analysis (e.g. if a participant experienced a serious ‘headache’ and serious ‘dizziness’, the participant would be counted twice in the MedDRA system organ class analysis of serious nervous system disorders); we therefore consider these MedDRA system organ class analyses exploratory.

### Post hoc exploratory outcome assessment

As we did not obtain complete case report forms or individual participant data for any trial, and as the trials’ harm assessments had low internal and external validity (see Table 1 and the “Discussion” section), we performed post hoc exploratory outcome analyses where we (1) compared the clinical study report data with pharmacovigilance data; and (2) assessed signs and symptoms of POTS and CRPS (see protocol amendment on PROSPERO [39]).

1. We compared the three largest harm clusters reported from pharmacovigilance up to 1 January 2015 to the World Health Organisation’s (WHO) VigiBase® [29] with the clinical study report data (for example, VigiBase’s largest HPV vaccine harm cluster—‘expected systemic reactions’—consists of the MedDRA-preferred terms headache, nausea, pyrexia, dizziness and vomiting). This was done to assess if the pharmacovigilance data were comparable to the clinical study report data. We used the individual harm cluster terms and found the corresponding MedDRA-preferred terms in the clinical study report data. The data were synthesised or those MedDRA-preferred terms included in each harm cluster.
2. POTS and CRPS are rare syndromes that are difficult to identify; as mentioned, about 30 cases of POTS and CRPS were not recognised in the HPV vaccine manufacturers’ trials [31, 32], and there were no reports of POTS and CRPS in the clinical study reports (see Table 9 and the “Results” section). To assess whether signs and symptoms consistent with POTS and CRPS were present in the data, we asked a physician (Louise Brinth) with clinical expertise in POTS and CRPS to assess the reported MedDRA-preferred terms as ‘definitely’, ‘probably’, ‘probably not’ or ‘definitely not’ associated with the syndromes. As an example, the physician judged the MedDRA-preferred terms ‘dizziness postural’ and ‘pain in extremity’ to be ‘definitely’ associated with POTS and CRPS, respectively. The physician was blinded to the allocation groups and outcome data. The data was synthesised for those MedDRA-preferred terms that the physician judged ‘definitely’ associated with POTS or CRPS. (Note that the synthesis of two or more different MedDRA-preferred term categories may include a participant more than once in an analysis.)

### Data synthesis and analysis

Risk ratios were meta-analysed with the random-effects inverse variance method. As small trials carry more weight with this method, we compared random-effects to a fixed-effect risk ratio for all outcomes. Absolute risk estimates were calculated as the number needed to vaccinate (NNV) or harm (NNH). Review Manager 5 was used for data synthesis and the intention to treat principle to calculate effect estimates. Sensitivity and subgroup analyses were conducted to investigate potential sources of heterogeneity by taking account of age, gender, risk of bias [25] and type of HPV vaccine and comparator.

## Results

### Characteristics of included trials

We identified 50 eligible studies: 43 industry trials, 5 industry follow-up studies and 2 non-industry trials (see Fig. 1). We obtained 24 clinical study reports of 58,412 pages from EMA and GlaxoSmithKline for 22 industry trials and 2 industry follow-up studies (17 Cervarix™, 5 Gardasil™, 1 Gardasil 9™ and 1 monovalent Merck HPV type 16 vaccine) with a total of 95,670 participants (79,102 females and 16,568 males age 8–72) and 393,194 person-years (see Tables 2 and 3 and Additional file 2). The 24 clinical study reports included 79% (95,670/121,441) of the total eligible sample of the 50 identified eligible studies. It is possible that for some of these eligible studies clinical study reports were never written but journal articles were published. The mean follow-up time was 49 months (weighted by sample size). About two fifths of the participants in the control groups received the aluminium-based adjuvants that were used in the HPV vaccines (18,192/48,595), three fifths received hepatitis vaccines that also contained the aluminium-based adjuvants that were used in the HPV vaccines—except for the hepatitis vaccine Aimmugen™—(29,500), and less than a thousand participants received carrier solution (597) or saline placebo (306).

**Table 2:** Benefits and harms of the HPV vaccines: number of pages obtained of clinical study reports from the European Medicines Agency and GlaxoSmithKline

	HPV vaccine manufacturer	Study programme ID	Total pages obtained	European Medicines Agency	GlaxoSmithKline
1	GlaxoSmithKline	HPV-001	5813	5813	0
2	GlaxoSmithKline	HPV-003	799	0	799
3	GlaxoSmithKline	HPV-008	11,456	4263	7193
4	GlaxoSmithKline	HPV-013	8323	382	7941
5	GlaxoSmithKline	HPV-015	6290	543	5747
6	GlaxoSmithKline	HPV-023	936	0	936
7	GlaxoSmithKline	HPV-029	1543	0	1543
8	GlaxoSmithKline	HPV-030	1351	0	1351
9	GlaxoSmithKline	HPV-031	476	0	476
10	GlaxoSmithKline	HPV-032	2912	0	2912
11	GlaxoSmithKline	HPV-033	587	0	587
12	GlaxoSmithKline	HPV-035	451	0	451
13	GlaxoSmithKline	HPV-038	957	0	957
14	GlaxoSmithKline	HPV-040	2892	128	2764
15	GlaxoSmithKline	HPV-058	1745	0	1745
16	GlaxoSmithKline	HPV-063	1474	0	1474
17	GlaxoSmithKline	HPV-069	819	0	819
18	Merck Sharp and Dohme	V501-005	357	357	0
19	Merck Sharp and Dohme	V501-013	1797	1797	0
20	Merck Sharp and Dohme	V501-015	713	713	0
21	Merck Sharp and Dohme	V501-018	1014	1014	0
22	Merck Sharp and Dohme	V501-019	2645	2645	0
23	Merck Sharp and Dohme	V501-020	2595	2595	0
24	Merck Sharp and Dohme	V503-006	467	467	0
	Total pages obtained		58,412	20,717	37,695

**Table 3:** Benefits and harms of the HPV vaccines: characteristics of included participants

Characteristics of included participants^a^	Total		HPV vaccine				Comparator		
	HPV vaccine (*N* = 47,075)	Comparator (*N* = 48,595)	Cervarix (*N* = 31,316)	Gardasil (*N* = 13,937)	Gardasil 9 (*N* = 618)	HPV 16 vaccine (*N* = 1204)	Placebo (*N* = 306)	Adjuvant^b^ (*N* = 18,789)	Hepatitis vaccine^c^ (*N* = 29,500)
Participation									
Randomised	47,075	48,595	31,316	13,937	618	1204	306	18,789	29,500
Received one (1) dose	47,012 (99%)	48,556 (99%)	31,291 (99%)	13,927 (99%)	615 (99%)	1193 (99%)	306 (100%)	18,750 (99%)	29,500 (100%)
Received two (2) doses	46,105 (98%)	47,725 (98%)	30,788 (98%)	13,564 (97%)	604 (98%)	1092 (91%)	304 (99%)	18,304 (97%)	29,117 (99%)
Received three (3) doses	45,079 (96%)	46,726 (96%)	30,073 (96%)	13,286 (95%)	597 (97%)	1019 (85%)	300 (98%)	17,906 (96%)	28,520 (97%)
Completed vaccination period	44,202 (94%)	45,862 (94%)	29,331 (94%)	13,156 (94%)	595 (97%)	993 (82%)	300 (98%)	17,809 (95%)	27,753 (94%)
Entered follow-up period	18,540 (39%)	18,059 (37%)	4090 (14%)	13,344 (96%)	Not applicable	1126 (94%)	Not applicable	17,590 (94%)	469 (2%)
Completed follow-up period	15,826 (34%)	14,601 (30%)	2929 (10%)	11,986 (86%)	Not applicable	835 (69%)	Not applicable	14,445 (77%)	156 (1%)
Gender									
Female	42,036 (89%)	37,066 (76%)	28,876 (92%)	11,338 (81%)	618 (100%)	1204 (100%)	306 (100%)	16,481 (88%)	20,279 (69%)
Age									
Mean age in years	20.3	20.2	21.2	21.4	19.0	20.0	19.0	22.9	20.5
Age group range in years	9–72	8–68	9–72	9–45	12–26	16–25	12–26	9–68	8–46
Race									
Asian	7589 (16%)	7295 (15%)	6232 (20%)	1248 (9%)	40 (6%)	69 (6%)	14 (5%)	2678 (14%)	4603 (16%)
Black	1426 (3%)	1492 (3%)	467 (2%)	862 (6%)	3 (1%)	94 (8%)	3 (1%)	1108 (6%)	381 (1%)
Hispanic	4492 (10%)	4378 (9%)	1787 (6%)	2616 (19%)	0 (0%)	89 (7%)	0 (0%)	3403 (18%)	975 (3%)
White	31,743 (67%)	33,558 (69%)	22,335 (70%)	7998 (56%)	483 (78%)	918 (76%)	231 (75%)	9960 (53%)	23,367 (79%)
Other	1625 (3%)	1576 (3%)	297 (1%)	1202 (9%)	92 (15%)	34 (3%)	58 (19%)	1343 (7%)	174 (1%)
Unknown	209 (1%)	296 (1%)	198 (1%)	11 (1%)	0 (0%)	0 (0%)	0 (0%)	297 (2%)	0 (0%)

^a^See Additional file 2 for details on the characteristics of included participants. Table 3 does not include data from the two follow-up studies HPV-023 (follow-up for trial HPV-001) of 433 participants and HPV-063 (follow-up for trial HPV-032) of 752 participants

^b^Adjuvant comparators included amorphous aluminium hydroxyphosphate sulphate (AAHS), aluminium hydroxide (Al[OH]_3_) and Gardasil’s carrier solution (yeast protein, sodium chloride, L-histidine, polysorbate 80 and sodium borate)

^c^Hepatitis vaccines included Aimmugen™ (hepatitis A vaccine), Engerix-B™ (hepatitis B vaccine), Havrix™ (hepatitis A vaccine) and Twinrix Paediatric™ (hepatitis A and B vaccine); see Additional file 2

### Characteristics of potentially eligible studies

For the 26 remaining and potentially eligible studies (23 trials and three follow-up studies) for which no clinical study reports were obtained (or similar reports for the two non-industry trials), numbers of participants were identified for 20 of the 23 industry and 1 of the 2 non-industry trials. The trials included 25,632 and 139 participants, respectively, which were equal to 21% of the total eligible sample (25,771/121,441). These studies were not included in the review or analyses (see Additional file 3).

### Risk of bias of included trials

**Fig. 2:**
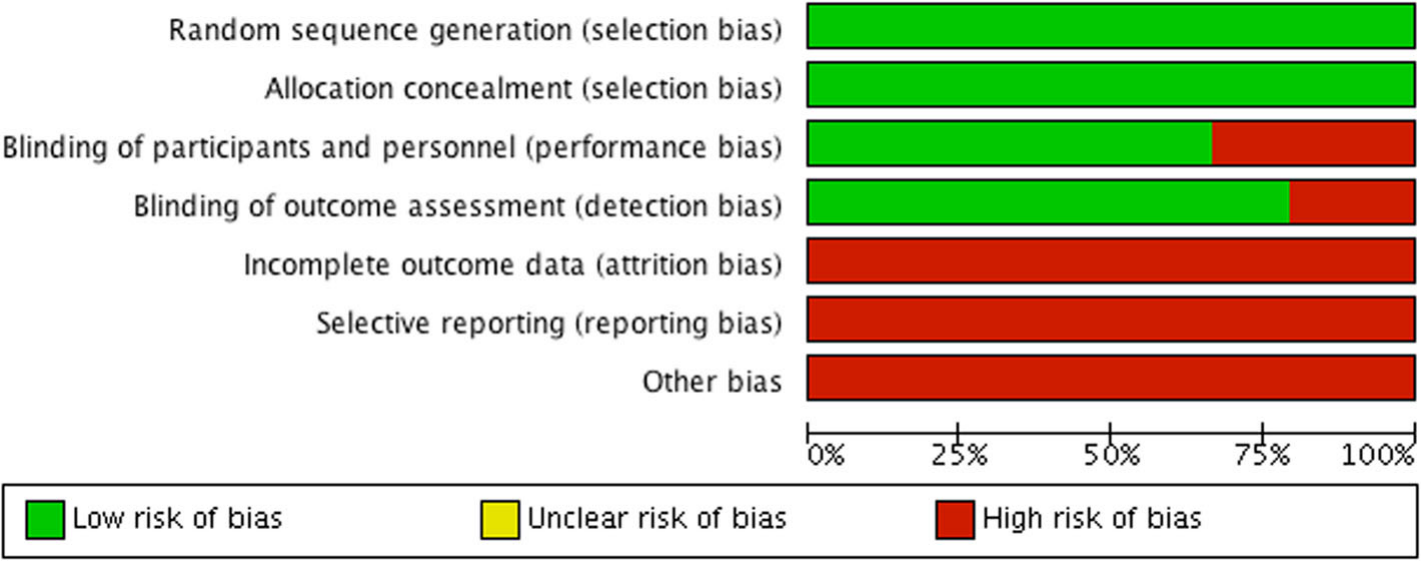
Benefits and harms of the HPV vaccines: risk of bias graph

**Fig. 3:**
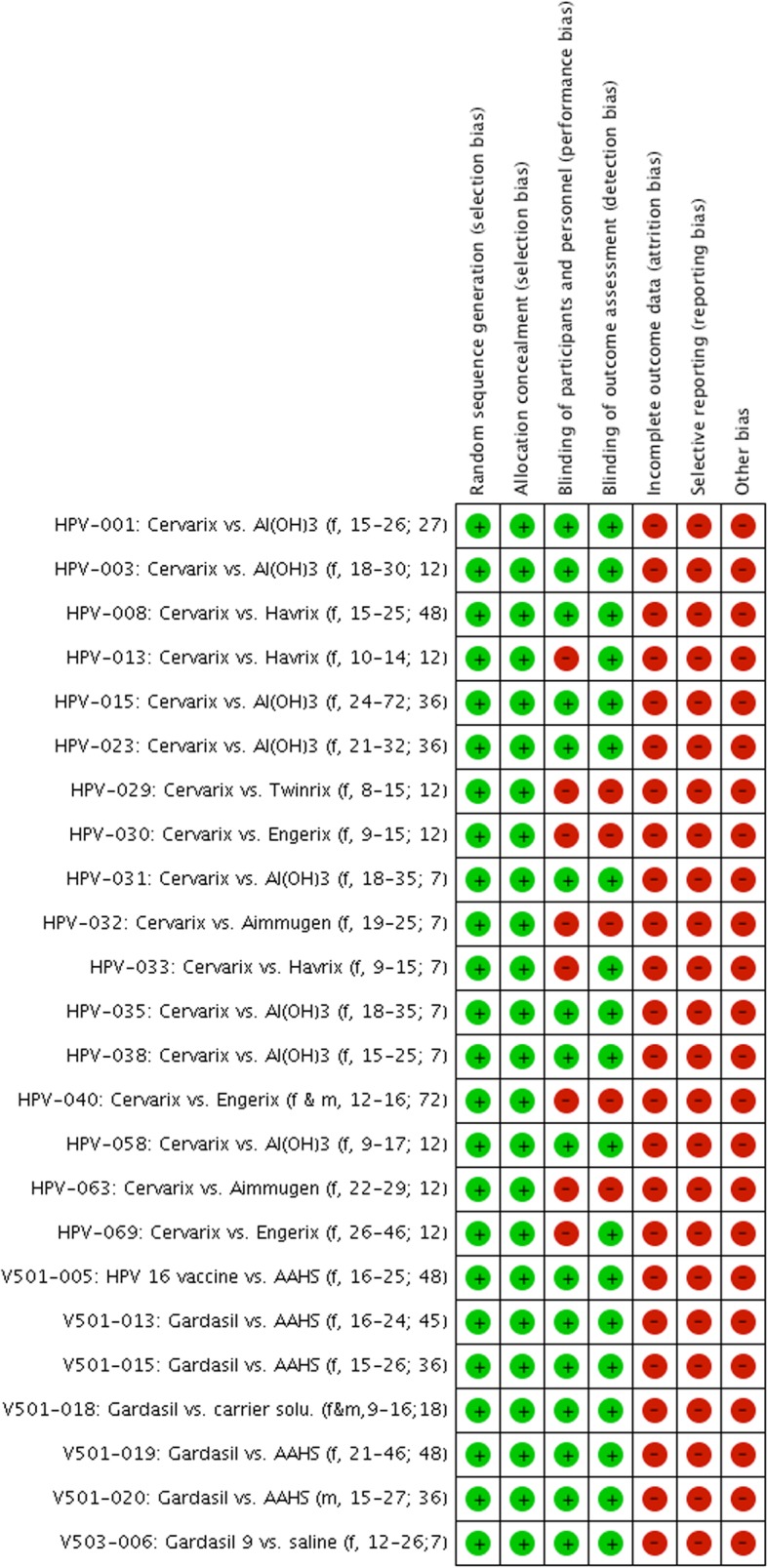
Benefits and harms of the HPV vaccines: risk of bias summary (each study is noted as “manufacturer ID: type of HPV vaccine vs. type of comparator (included gender, age group; months of follow-up)”, e.g. “HPV-001: Cervarix vs. Al(OH)_3_ (f, 15-26; 27)”)

All 22 trials and the 2 follow-up studies were at low risk of bias for ‘sequence generation’ and ‘allocation concealment’, and the majority were at low risk of bias for ‘blinding of outcome assessors’ (19/24) and ‘blinding of participants and personnel’ (16/24; see Figs. 2 and 3 and Additional file 2). However, due to the following reasons, we judged all studies to be at high risk of bias. Nearly all control participants (48,289/48,595, 99%) received an active comparator such as HPV vaccine aluminium-containing adjuvants or hepatitis vaccines. This distorted—to an unknown extent—the assessment of harms, as the trials tested an HPV vaccine vs. an active part of the same HPV vaccine (see reference [38] for additional clarification). Furthermore, serious harms were incompletely reported for 72% of the participants (68,610/95,670; see Table 1 and Additional file 2). All 24 clinical study reports contained redactions—especially of harms—and lacked significant parts such as serious harm narratives and case report forms (except for two reports: HPV-001 and HPV-008, which, however, included less than half of the participants’ case report forms) [38]. These situations are not covered by Cochrane’s risk of bias tool version 2011. Although not related to participant attrition, we judged the lack of serious harm narratives and case report forms as high risk of ‘incomplete outcome data’. In addition, while not related to the availability of study protocols, we judged the redactions of the clinical study reports as high risk of ‘selective outcome reporting’. We decided to conduct meta-analyses, since the high risk of bias mainly constituted situations that to our knowledge are not related to empirically verified bias mechanisms.

### Benefits

Seven clinical study reports assessed histological outcomes of which four reported HPV-related cancer outcomes irrespective of involved HPV types. At 4 years follow-up, the HPV vaccines did not decrease HPV-related cancer (7 in the HPV vaccine groups vs. 3 in the comparator groups, risk ratio [RR] 1.68 [95% confidence interval, CI, 0.51 to 5.49], *P* = 0.39, *I*^2^ = 0%) or deaths hereof (2 vs. 1, RR 1.44 [95% CI 0.23 to 9.12], *P* = 0.70, *I*^2^ = 0%); whereas they decreased HPV-related carcinoma in situ (367 vs. 490, RR 0.73 [95% CI 0.53 to 1.00], number needed to vaccinate [NNV] 387, *P* = 0.05, *I*^2^ = 67%) and the composite surrogate outcome of HPV-related moderate intraepithelial neoplasia or worse (952 vs. 1239, RR 0.78 [95% CI 0.66 to 0.91], NNV 190, *P* = 0.002, *I*^2^ = 53%). The HPV vaccines also decreased HPV-related external genital lesions (289 vs. 582, RR 0.56 [95% CI 0.39 to 0.82], NNV 47, *P* = 0.003, *I*^2^ = 83%) and HPV-related treatment procedures such as cervical conisations (1018 vs. 1416, RR 0.71 [95% CI 0.63 to 0.80], NNV 75, *P* < 0.00001, *I*^2^ = 45%) (see Table 4 and Additional file 4).

**Table 4:** Benefits and harms of the HPV vaccines: summary of HPV-related outcomes

Summary of HPV-related outcomes^a^	HPV vaccine (*N* = 47,075)	Comparator (*N* = 48,595)	Risk ratio^c^ [95% CI]
Cancer mortality			
Total	2	1	1.44 [0.23, 9.12]
Cervical	1 (50%)	0 (0%)	2.99 [0.12, 73.33]
Oropharyngeal	1 (50%)	1 (100%)	1.00 [0.10, 9.58]
Cancer incidence			
Total	7	3	1.68 [0.51, 5.49]
Anal	0 (0%)	0 (0%)	Not applicable
Cervical	3 (43%)	2 (67%)	1.41 [0.19, 10.21]
Oropharyngeal	1 (14%)	1 (33%)	1.00 [0.10, 9.58]
Penile	Not reported	Not reported	Not applicable
Vaginal	1 (14%)	0 (0%)	2.99 [0.12, 73.33]
Vulvar	2 (29%)	0 (0%)	3.01 [0.31, 28.89]
Not HPV-related	20	23	0.90 [0.49, 1.63]
Carcinoma in situ incidence			
Total	367	490	0.73 [0.53, 1.00]
Anal (AIN3)	Not reported	Not reported	Not applicable
Cervical	367 (100%)	490 (100%)	0.73 [0.53, 1.00]
Adenoid type (AIS)	9 (2%)	31 (6%)	0.32 [0.15, 0.66]
Squamous type (CIN3)	358 (98%)	459 (94%)	0.85 [0.61, 1.17]
Penile (PIN3)	Not reported	Not reported	Not applicable
Vaginal (VaIN3)	Not reported	Not reported	Not applicable
Vulvar (VIN3)	Not reported	Not reported	Not applicable
Moderate intraepithelial neoplasia incidence			
Total	538	763	0.81 [0.59, 1.11]
Anal (AIN2)	0 (0%)	0 (0%)	Not applicable
Cervical (CIN2)	538 (100%)	763 (100%)	0.81 [0.59, 1.11]
Penile (PIN2)	Not reported	Not reported	Not applicable
Vaginal (VaIN2)	Not reported	Not reported	Not applicable
Vulvar (VIN2)	Not reported	Not reported	Not applicable
Carcinoma in situ or worse incidence			
Total	372	498	0.79 [0.59, 1.05]
Anal (AIN3^+^)	Not reported	Not reported	Not applicable
Cervical (CIN3^+^, AIS included)	372 (100%)	498 (100%)	0.79 [0.59, 1.05]
Penile (PIN3^+^)	Not reported	Not reported	Not applicable
Vaginal (VaIN3^+^)	Not reported	Not reported	Not applicable
Vulvar (VIN3^+^)	Not reported	Not reported	Not applicable
Moderate intraepithelial neoplasia or worse incidence			
Total	952	1239	0.78 [0.66, 0.91]
Anal (AIN2^+^)	0 (0%)	0 (0%)	Not applicable
Cervical (CIN2^+^)	892 (93%)	1144 (92%)	0.81 [0.68, 0.97]
Penile (PIN2^+^)	3 (1%)	3 (1%)	1.00 [0.20, 4.95]
Vaginal (VaIN2^+^)	17 (2%)	27 (2%)	0.64 [0.32, 1.27]
Vulvar (VIN2^+^)	18 (2%)	36 (3%)	0.49 [0.18, 1.36]
Vaginal or vulvar (VIN2^+^ or VaIN2^+^)	22 (2%)	29 (2%)	0.76 [0.44, 1.32]
External genital lesion (EGL) incidence			
Total	289	582	0.56 [0.39, 0.82]
HPV-related referral procedures^b^			
Any	1941	2264	0.86 [0.81, 0.90]
Biopsy	2449	3021	0.74 [0.62, 0.88]
Endoscopy	4354	4965	0.88 [0.85, 0.91]
Treatment (surgical and non-surgical)	1018	1416	0.71 [0.63, 0.80]

^a^See Additional file 4 sections 1 to 8 for meta-analyses of HPV-related outcomes. It was not feasible to present this summary table for the 16 subgroups (based on age group, gender, type of HPV vaccine and comparator) of the 24 included clinical study reports

^b^Two trials (V501-013 and V501-015) reported ‘any’ procedure, while other trials reported individual outcomes, for example, ‘biopsy’

^c^Risk ratios were calculated with the random-effects inverse variance method

### Harms

#### Serious harms

The HPV vaccines did not significantly increase fatal harms (45 vs. 38, RR 1.19 [95% CI 0.65 to 2.19], *P* = 0.58, *I*^2^ = 30%) or serious harms (1404 vs. 1357, RR 1.01 [95% CI 0.94 to 1.08], *P* = 0.79, *I*^2^ = 0%), and no individual fatal or serious harm classified with a MedDRA-preferred term was significantly increased or decreased by the HPV vaccines (see Table 5 and Additional file 4).

**Table 5:** Benefits and harms of the HPV vaccines: summary of fatal and serious harms

Summary of fatal and serious harms^a^	HPV vaccine (*N* = 47,075)	Comparator (*N* = 48,595)	Risk ratio^e^ [95% CI]
Fatal harms			
Participants with fatal harms^b^	45	38	1.19 [0.65, 2.19]
Number of MedDRA-classified fatal harms^b^	79	51	Not applicable
Number of fatal harms judged HPV vaccine-related	0 (0%)	0 (0%)	Not applicable
Most common fatal harms (MedDRA-preferred terms, *n* = participants)			
Cardiorespiratory arrest	3	2	0.99 [0.13, 7.65]
Completed suicide	4	8	0.58 [0.15, 2.19]
Gunshot wound	2	3	0.74 [0.09, 5.85]
Homicide	2	2	0.95 [0.14, 6.50]
Road traffic accident	5	7	0.77 [0.24, 2.46]
Fatal harms most increased by the HPV vaccines (MedDRA-preferred terms, *n* = participants)			
Cardiac arrest	2	0	3.00 [0.31, 28.82]
Metastases to lung	2	0	3.00 [0.31, 28.82]
Renal failure acute	2	0	3.00 [0.31, 28.82]
Systemic lupus erythematosus	2	0	3.00 [0.31, 28.82]
Traumatic intracranial haemorrhage	2	0	3.00 [0.31, 28.82]
Fatal harms most decreased by the HPV vaccines (MedDRA-preferred terms, *n* = participants)^c^			
Completed suicide	4	8	0.58 [0.15, 2.19]
Gunshot wound	2	3	0.74 [0.09, 5.85]
Road traffic accident	5	7	0.77 [0.24, 2.46]
Serious harms			
Participants with serious harms^d^	1404	1357	1.01 [0.94, 1.08]
Participants that withdrew due to a serious harm	54 (4%)	49 (4%)	1.08 [0.72, 1.61]
Number of MedDRA-classified serious harms^d^	1741	1628	Not applicable
Number of serious harms judged HPV vaccine-related	46 (3%)	44 (3%)	Not applicable
Most common serious harms (MedDRA-preferred terms, *n* = participants)			
Abortion missed	33	41	0.81 [0.51, 1.27]
Abortion spontaneous	89	78	1.14 [0.84, 1.55]
Abortion spontaneous complete	63	62	1.01 [0.71, 1.44]
Abortion spontaneous incomplete	73	54	1.35 [0.95, 1.92]
Appendicitis	72	82	0.85 [0.62, 1.17]
Serious harms most increased by the HPV vaccines (MedDRA-preferred terms, *n* = participants)			
Abortion spontaneous	89	78	1.14 [0.84, 1.55]
Abortion spontaneous incomplete	73	54	1.35 [0.95, 1.92]
Pneumonia	26	15	1.64 [0.87, 3.09]
Pyelonephritis	31	17	1.70 [0.93, 3.10]
Tonsillitis	18	9	1.59 [0.72, 3.49]
Serious harms most decreased by the HPV vaccines (MedDRA-preferred terms, *n* = participants)			
Abortion missed	33	41	0.81 [0.51, 1.27]
Appendicitis	72	82	0.85 [0.62, 1.17]
Ligament rupture	5	12	0.44 [0.15, 1.29]
Ovarian cyst rupture	6	13	0.46 [0.18, 1.21]
Overdose	22	31	0.72 [0.42, 1.23]

^a^See Additional file 4 sections 9 and 10 for fatal and serious harm meta-analyses. The applied harm categories are MedDRA-preferred terms. It was not feasible to present this summary table for the 16 subgroups (based on age group, gender, type of HPV vaccine and comparator) of the 24 included clinical study reports

^b^The clinical study reports reported 130 individual MedDRA-classified fatal harms for 83 participants

^c^There were 20 different MedDRA-preferred term categories of fatal harms with the same non-significant difference, i.e. no fatal harm in the HPV vaccine group and one fatal harm in the comparator group

^d^The clinical study reports reported 3369 individual MedDRA-classified serious harms for 2761 participants, i.e. 1.2 serious harms per participant. Each MedDRA-classified serious harm was reported as the number of participants with a MedDRA-classified serious harm over the total number of participants

^e^Risk ratios were calculated with the random-effects inverse variance method

#### New onset diseases

The HPV vaccines increased new onset back pain (397 vs. 336, RR 1.15 [95% CI 1.00 to 1.33], NNH 589, *P* = 0.05, *I*^2^ = 0%) but decreased new onset gynaecological chlamydia infection (1409 vs. 1512, RR 0.93 [95% CI 0.87 to 1.00], NNV 176, *P* = 0.05, *I*^2^ = 0%) and vaginal infection (369 vs. 420, 0.87 [95% CI 0.76 to 1.00], NNV 150, *P* = 0.05, *I*^2^ = 0%) (see Table 6 and Additional file 4).

**Table 6:** Benefits and harms of the HPV vaccines: summary of new onset diseases

Summary of new onset diseases^a^	HPV vaccine total (*N* = 47,075)	Comparator total (*N* = 48,595)	Risk ratio^f^ total [95% CI]	Risk ratio^f^ MSC [95% CI]	Risk ratio^f^ NMH [95% CI]
Total					
Participants with new onset diseases^b^	14,258	14,014	0.99 [0.97, 1.02]	0.98 [0.90, 1.06]	1.00 [0.97, 1.03]
Follow-up^c^	2296	2365	0.98 [0.94, 1.01]	Not applicable	0.98 [0.94, 1.01]
Number of MedDRA-classified new onset diseases^b^	47,474	46,662	Not applicable	Not applicable	Not applicable
Medically significant conditions (MSC)^d^	7882 (17%)	7994 (17%)	Not applicable	Not applicable	Not applicable
New medical history (NMH)^e^	39,592 (83%)	38,668 (83%)	Not applicable	Not applicable	Not applicable
Most common new onset diseases (MedDRA-preferred terms, *n* = participants)					
*MSC*					
Depression	443	432	1.02 [0.89, 1.16]	1.02 [0.85, 1.23]	1.01 [0.84, 1.22]
Genitourinary tract gonococcal infection	149	162	0.92 [0.74, 1.15]	0.91 [0.73, 1.14]	1.15 [0.37, 3.52]
Gynaecological chlamydia infection	1409	1512	0.93 [0.87, 1.00]	0.95 [0.88, 1.03]	0.87 [0.76, 1.00]
*NMH*					
Vaginal candidiasis	1297	1359	0.95 [0.89, 1.02]	Not applicable	0.95 [0.89, 1.02]
Vaginitis bacterial	1185	1204	0.98 [0.91, 1.06]	Not applicable	0.98 [0.91, 1.06]
Urinary tract infection	1023	1086	0.93 [0.86, 1.01]	0.33 [0.01, 8.19]	0.93 [0.86, 1.02]
New onset diseases most increased by the HPV vaccines (MedDRA-preferred terms, *n* = participants)					
*MSC*					
Abdominal pain	433	374	1.21 [0.98, 1.50]	1.38 [1.00, 1.92]	1.17 [0.87, 1.57]
Back pain	397	336	1.15 [1.00, 1.33]	1.40 [1.05, 1.86]	1.08 [0.91, 1.28]
Headache	771	693	1.06 [0.92, 1.22]	1.29 [0.75, 2.24]	1.04 [0.93, 1.15]
*NMH*					
Amenorrhoea	394	359	1.09 [0.87, 1.37]	0.66 [0.38, 1.15]	1.17 [0.93, 1.48]
Headache	771	693	1.06 [0.92, 1.22]	1.29 [0.75, 2.24]	1.04 [0.93, 1.15]
Joint sprain	113	83	1.18 [0.80, 1.75]	0.60 [0.29, 1.22]	1.45 [0.94, 2.24]
New onset diseases most decreased by the HPV vaccines (MedDRA-preferred terms, *n* = participants)					
*MSC*					
Cystitis	480	502	0.93 [0.77, 1.09]	0.65 [0.44, 0.96]	0.99 [0.87, 1.13]
Gynaecological chlamydia infection	1409	1512	0.93 [0.87, 1.00]	0.95 [0.88, 1.03]	0.87 [0.76, 1.00]
Type 2 diabetes mellitus	31	47	0.89 [0.38, 2.09]	0.62 [0.32, 1.20]	3.00 [0.47, 19.02]
*NMH*					
Urinary tract infection	1023	1086	0.93 [0.86, 1.01]	0.33 [0.01, 8.19]	0.93 [0.86, 1.02]
Vaginal candidiasis	1297	1359	0.95 [0.89, 1.02]	Not applicable	0.95 [0.89, 1.02]
Vaginal infection	369	420	0.87 [0.76, 1.00]	Not applicable	0.87 [0.76, 1.00]

^a^See Additional file 4 section 11 for meta-analyses of new onset diseases. The applied harm categories are MedDRA-preferred terms. New onset diseases consist of ‘medically significant conditions’ (MSC) and ‘new medical history’ (NMH). Numbers for ‘HPV vaccine’ and ‘comparator’ are the total of MSC and NMH. We divided new onset diseases for MSC and NMH, since the definitions for MSC and NMH differed (see Table 1). It was not feasible to present this summary table for the 16 subgroups (based on age group, gender, type of HPV vaccine and comparator) of the 24 included clinical study reports

^b^The clinical study reports reported 94,136 individual MedDRA-preferred term classified new onset diseases for 28,272 participants, i.e. 3.3 new onset diseases per participant. New onset diseases were reported as the number of participants over the total number of participants

^c^‘Follow-up’ represents the trials V501-005, V501-019 and V501-020 that had dichotomized reporting of new medical history (NMH) into the vaccination period (day 0 to month 7) and follow-up period (from month 7 to the last day of follow-up). We included the vaccination periods for these trials in ‘participants with new onset diseases’ and included the follow-up periods in ‘follow-up’

^d^GlaxoSmithKline defined ‘medically significant conditions’ as “Adverse events prompting emergency room or physician visits that are not (1) related to common diseases or (2) routine visits for physical examination or vaccination, or SAEs [serious adverse events] that are not related to common diseases. Serious adverse events related to common diseases were reported but are not classified as medically significant conditions for analysis purposes. Common diseases include: upper respiratory infections, sinusitis, pharyngitis, gastroenteritis, urinary tract infections, cervicovaginal yeast infections, menstrual cycle abnormalities and injury”

^e^Merck Sharp and Dohme did not provide a formal definition for ‘new medical history’ but described ‘new medical history’ as “all new reported diagnoses” in the clinical study report of trial V501-019

^f^Risk ratios were calculated with the random-effects inverse variance method

#### General harms

The HPV vaccines increased general harms (13,248 vs. 12,394, RR 1.07 [95% CI 1.03 to 1.11], NNH 51, *P* = 0.0002, *I*^2^ = 77%)—especially myalgia (3989 vs. 3047, RR 1.41 [95% CI 1.24 to 1.60], NNH 26, *P* < 0.00001, *I*^2^ = 80%), fatigue (4933 vs. 4489, RR 1.13 [95% CI 1.08 to 1.18], NNH 67, *P* < 0.00001, *I*^2^ = 22%) and headache (5561 vs. 5246, RR 1.06 [95% CI 1.02 to 1.11], NNH 83, *P* = 0.009, *I*^2^ = 40%) (see Table 7 and Additional file 4).

**Table 7:** Benefits and harms of the HPV vaccines: summary of general harms

Summary of general harms^a^	HPV vaccine total (*N* = 47,075)	Comparator total (*N* = 48,595)	Risk ratio^f^ total [95% CI]	Risk ratio^f^ SGAE [95% CI]	Risk ratio^f^ UGAE [95% CI]	Risk ratio^f^ SYAE [95% CI]
Total						
Participants with general harms^b^	13,248	12,394	1.07 [1.03, 1.11]	1.11 [1.06, 1.16]^g^	1.11 [1.06, 1.16]^g^	1.01 [0.98, 1.03]
Number of MedDRA-classified general harms^b^	37,999	31,916	Not applicable	Not applicable	Not applicable	Not applicable
Solicited general adverse events (SGAE)^c^	30,408 (80%)	25,300 (79%)	Not applicable	Not applicable	Not applicable	Not applicable
Unsolicited general adverse events (UGAE)^d^	3197 (8%)	3136 (10%)	Not applicable	Not applicable	Not applicable	Not applicable
Systemic adverse events (SYAE)^e^	4394 (12%)	3480 (11%)	Not applicable	Not applicable	Not applicable	Not applicable
Most common general harms (MedDRA-preferred terms, *n* = participants)						
*SGAE and UGAE*						
Fatigue	4933	4489	1.13 [1.08, 1.18]	1.14 [1.09, 1.19]	1.00 [0.15, 6.53]	0.92 [0.70, 1.20]
Headache	5561	5246	1.06 [1.02, 1.11]	1.08 [1.03, 1.14]	1.76 [1.26, 2.47]	0.98 [0.90, 1.07]
Myalgia	3989	3047	1.41 [1.24, 1.60]	1.42 [1.24, 1.63]	1.15 [0.24, 5.57]	1.33 [0.95, 1.85]
*SYAE*						
Headache	5561	5246	1.06 [1.02, 1.11]	1.08 [1.03, 1.14]	1.76 [1.26, 2.47]	0.98 [0.90, 1.07]
Pyrexia	1599	1386	1.12 [1.02, 1.22]	1.15 [1.06, 1.25]	1.47 [0.93, 2.34]	1.05 [0.80, 1.36]
Nasopharyngitis	339	277	1.17 [0.91, 1.50]	Not applicable	1.40 [0.94, 2.09]	0.95 [0.78, 1.16]
General harms most increased by the HPV vaccines (MedDRA-preferred terms, *n* = participants)						
*SGAE and UGAE*						
Fatigue	4933	4489	1.13 [1.08, 1.18]	1.14 [1.09, 1.19]	1.00 [0.15, 6.53]	0.92 [0.70, 1.20]
Headache	5561	5246	1.06 [1.02, 1.11]	1.08 [1.03, 1.14]	1.76 [1.26, 2.47]	0.98 [0.90, 1.07]
Myalgia	3989	3047	1.41 [1.24, 1.60]	1.42 [1.24, 1.63]	1.15 [0.24, 5.57]	1.33 [0.95, 1.85]
*SYAE*						
Myalgia	3989	3047	1.41 [1.24, 1.60]	1.42 [1.24, 1.63]	1.15 [0.24, 5.57]	1.33 [0.95, 1.85]
Nausea	213	148	1.21 [0.89, 1.65]	Not applicable	1.32 [0.35, 4.98]	1.25 [0.84, 1.86]
Pyrexia	1599	1386	1.12 [1.02, 1.22]	1.15 [1.06, 1.25]	1.47 [0.93, 2.34]	1.05 [0.80, 1.36]
General harms most decreased by the HPV vaccines (MedDRA-preferred terms, *n* = participants)						
*SGAE and UGAE*						
Influenza	119	120	0.91 [0.61, 1.36]	Not applicable	0.88 [0.39, 1.97]	0.94 [0.56, 1.58]
Cough	86	87	0.89 [0.65, 1.21]	Not applicable	0.83 [0.46, 1.49]	0.90 [0.60, 1.37]
Oropharyngeal pain	111	97	1.10 [0.80, 1.50]	Not applicable	0.91 [0.58, 1.43]	1.29 [0.75, 2.22]
*SYAE*						
Fungal infection	4	11	0.78 [0.09, 6.43]	Not applicable	3.01 [0.31, 28.83]	0.18 [0.04, 0.82]
Sinus headache	9	15	0.49 [0.21, 1.14]	Not applicable	Not applicable	0.49 [0.21, 1.14]
Joint injury	2	5	0.47 [0.11, 2.01]	Not applicable	3.01 [0.31, 28.83]	0.15 [0.03, 0.88]

^a^See Additional file 4 section 12 for meta-analyses of general harms for the 16 subgroups (based on age group, type of HPV vaccine and comparator) of the 24 included clinical study reports. The applied harm categories are MedDRA-preferred terms. The table contains general harms of ‘solicited general adverse events’ (SGAE), ‘unsolicited general adverse events’ (UGAE) and ‘systemic adverse events’ (SYAE). Numbers for ‘HPV vaccine’ and ‘comparator’ are the total of SGAE, UGAE and SYAE, but to avoid double counting of participants, UGAE (that accounted for less than 10% of the general harms) were dismissed from the total risk ratio for studies that reported SGAE and UGAE separately (SGAE and UGAE were not reported as pooled estimates for individual general harms classified with MedDRA-preferred terms; see Additional file 4). It was not feasible to present this summary table for the 16 subgroups (based on age group, type of HPV vaccine and comparator) of the 24 included clinical study reports

^b^The clinical study reports reported 69,915 individual MedDRA-classified general harms for 25,642 participants, i.e. 2.7 general harms per participant. General harms were reported as the number of participants with a MedDRA-classified general harm over the total number of participants

^c^GlaxoSmithKline defined ‘solicited general adverse events’ (SGAE) as “Adverse events to be recorded [from day 0 to day 6 after each vaccination] as endpoints [arthralgia, fatigue, headache, myalgia, pyrexia, rash and urticaria] in the clinical study”

^d^GlaxoSmithKline defined ‘unsolicited general adverse events’ (UGAE) as “Any AE [adverse event] reported in addition to those solicited during the clinical study. Also, any “solicited” symptom with onset outside the specified period of follow-up for solicited symptoms was reported as an unsolicited AE”

^e^Merck Sharp and Dohme defined ‘systemic adverse events’ (SYAE) as “any systemic clinical adverse event that developed on the day of vaccination or during the 14 days after vaccination was recorded on the VRC [vaccination report card]”

^f^Risk ratios were calculated with the random-effects inverse variance method

^g^The total numbers of participants with general harms in Cervarix studies were reported as ‘solicited [SGAE] and unsolicited [UGAE]’, i.e. the risk ratio is similar for SGAE and UGAE

#### Exploratory harm analyses

The HPV vaccines increased serious nervous system disorders grouped in the MedDRA system organ class (72 vs. 46, RR 1.49 [95% CI 1.02 to 2.16], number needed to harm [NNH] 1325, *P* = 0.04, *I*^2^ = 0%) but decreased new onset vascular disorders grouped in the MedDRA system organ class (234 vs. 294, RR 0.80 [95% CI 0.67 to 0.94], NNV 439, *P* = 0.009, *I*^2^ = 0%) (see Table 8 and Additional file 4).

**Table 8:** Benefits and harms of the HPV vaccines: summary of exploratory harm analyses by MedDRA system organ class

Summary of exploratory harm analyses by MedDRA system organ class^a^	Fatal harms			Serious harms			New onset diseases^b^			General harms^c^		
	HPV vaccine (*N* = 47,075)	Comparator (*N* = 48,595)	Risk ratio^d^ [95% CI]	HPV vaccine (*N* = 47,075)	Comparator (*N* = 48,595)	Risk ratio^d^[95% CI]	HPV vaccine (*N* = 47,075)	Comparator (*N* = 48,595)	Risk ratio^d^ [95% CI]	HPV vaccine (*N* = 47,075)	Comparator (*N* = 48,595)	Risk ratio^d^ [95% CI]
Blood and lymphatic system disorders	0	0	Not applicable	11	12	0.97 [0.44, 2.18]	424	421	1.00 [0.88, 1.15]	17	10	1.33 [0.56, 3.15]
Cardiac disorders	13	5	2.45 [0.87, 6.87]	28	21	1.12 [0.49, 2.55]	108	86	1.09 [0.67, 1.78]	6	5	0.89 [0.26, 2.99]
Congenital, familial and genetic disorders	0	0	Not applicable	1	9	0.32 [0.09, 1.13]	50	67	0.74 [0.51, 1.06]	0	1	0.55 [0.02, 13.40]
Ear and labyrinth disorders	0	0	Not applicable	4	6	0.68 [0.19, 2.45]	154	139	1.04 [0.78, 1.39]	54	40	1.10 [0.71, 1.69]
Endocrine disorders	1	0	2.83 [0.12, 69,36]	7	4	1.60 [0.48, 5.35]	189	207	0.91 [0.74, 1.10]	1	1	0.72 [0.04, 11.85]
Eye disorders	0	0	Not applicable	6	4	1.51 [0.42, 5.37]	296	326	0.89 [0.76, 1.04]	40	39	0.84 [0.53, 1.33]
Gastrointestinal disorders	1	0	2.83 [0.12, 69,36]	107	92	1.12 [0.85, 1.48]	2226	2141	1.05 [0.95, 1.16]	4085	3735	1.09 [1.01, 1.17]
General disorders	0	0	Not applicable	13	9	1.45 [0.60, 3.50]	563	483	1.14 [1.01, 1.28]	6401	5842	1.10 [1.04, 1.17]
Hepatobiliary disorders	1	1	0.94 [0.06, 15.06]	52	48	1.03 [0.70, 1.52]	126	143	0.86 [0.61, 1.22]	2	1	2.00 [0.18, 22.00]
Immune system disorders	1	0	2.83 [0.12, 69,36]	9	14	0.68 [0.30, 1.58]	371	398	0.92 [0.78, 1.08]	35	24	1.29 [0.76, 2.18]
Infections and infestations	8	5	1.51 [0.49, 4.61]	400	367	1.04 [0.80, 1.37]	8970	9025	0.98 [0.96, 1.00]	1141	965	1.14 [1.00, 1.30]
Injury, poisoning and procedural complications	16	18	0.84 [0.43, 1.64]	199	224	0.85 [0.64, 1.13]	1205	1209	0.94 [0.82, 1.06]	142	122	1.03 [0.81, 1.33]
Investigations	0	0	Not applicable	1	2	0.69 [0.11, 4.40]	1438	1429	1.00 [0.94, 1.07]	7	4	1.34 [0.41, 4.41]
Metabolism and nutrition disorders	1	1	0.94 [0.06, 15.06]	19	11	1.44 [0.72, 2.89]	342	355	0.95 [0.82, 1.10]	17	15	0.98 [0.49, 1.96]
Musculoskeletal and connective tissue disorders	2	0	4.71 [0.23, 98.09]	35	31	1.07 [0.66, 1.74]	1263	1213	1.02 [0.94, 1.10]	6005	4683	1.34 [1.21, 1.49]
Neoplasms benign, malignant and unspecified	13	4	3.06 [1.00, 9.39]	68	56	1.20 [0.84, 1.71]	466	421	1.09 [0.96, 1.25]	2	2	0.71 [0.14, 3.51]
Nervous system disorders	4	1	3.77 [0.42, 33.71]	72	46	1.49 [1.02, 2.16]	1410	1305	1.06 [0.97, 1.16]	5967	5422	1.09 [1.04, 1.14]
Pregnancy, puerperium and perinatal conditions	0	0	Not applicable	458	426	1.08 [0.94, 1.23]	726	714	1.02 [0.92, 1.12]	1	0	2.91 [0.12, 68.66]
Psychiatric disorders	4	8	0.47 [0.14, 1.56]	80	87	0.92 [0.68, 1.25]	961	959	0.99 [0.91, 1.08]	49	53	0.90 [0.61, 1.33]
Renal and urinary disorders	2	1	1.88 [0.17, 20.77]	19	17	1.07 [0.57, 2.01]	404	395	1.01 [0.88, 1.16]	12	6	1.32 [0.48, 3.59]
Reproductive system and breast disorders	2	0	4.71 [0.23, 98.09]	72	78	0.90 [0.65, 1.24]	3458	3463	0.99 [0.95, 1.04]	213	158	1.13 [0.92, 1.38]
Respiratory, thoracic and mediastinal disorders	3	2	1.41 [0.24, 8.45]	44	34	1.26 [0.81, 1.97]	795	771	1.00 [0.91, 1.11]	464	394	1.02 [0.89, 1.18]
Skin and subcutaneous tissue disorders	1	0	2.83 [0.12, 69.36]	12	7	1.48 [0.60, 3.63]	1173	1176	0.99 [0.88, 1.12]	997	771	1.21 [1.03, 1.44]
Social circumstances	2	2	0.94 [0.13, 6.69]	2	2	0.95 [0.14, 6.50]	46	42	0.95 [0.62, 1.47]	1	1	1.00 [0.10, 9.60]
Surgical and medical procedures	0	0	Not applicable	6	5	1.15 [0.36, 3.67]	1318	1340	0.97 [0.90, 1.04]	0	0	Not applicable
Vascular disorders	4	3	1.26 [0.28, 5.61]	16	16	0.96 [0.47, 1.99]	234	294	0.80 [0.67, 0.94]	11	12	0.68 [0.28, 1.63]

^a^See Additional file 4 sections 9 to 12 for meta-analyses of MedDRA system organ classes. Only Merck clinical study reports aggregated data for MedDRA system organ classes per participant and only for new onset diseases (‘new medical history’) and general harms (‘systemic adverse events’). A participant may have been counted more than once in the MedDRA system organ class analyses of GlaxoSmithKline clinical study reports and in analyses of fatal and serious harms; we therefore consider these analyses exploratory. Risk ratios for GlaxoSmithKline and Merck studies are provided separately in Additional file 4. It was not feasible to present this summary table for the 16 subgroups (based on age group, gender, type of HPV vaccine and comparator) of the 24 included clinical study reports. To avoid double counting of participants in the total risk ratio estimate we only included the new onset diseases reported in the vaccination period for the trials V501-005, V501-019 and V501-020, and we only included solicited adverse events when studies also reported unsolicited adverse events (see Additional file 4)

^b^New onset diseases were compiled of the harm categories ‘medically significant conditions’ (for Cervarix) and ‘new medical history’ (for Gardasil, Gardasil 9 and the HPV 16 vaccine)

^c^General harms were compiled of the harm categories ‘solicited general adverse events’, ‘unsolicited general adverse events’ (for Cervarix) and ‘systemic adverse events’ (for Gardasil, Gardasil 9 and the HPV 16 vaccine). To avoid double counting of participants, ‘unsolicited general adverse events’ were dismissed from the total risk ratio for studies that reported SGAE and UGAE separately

^d^Risk ratios were calculated with the random-effects inverse variance method

#### Harms of special interest

Cases of anaphylaxis and syncope were evenly distributed. There were no cases of chronic fatigue syndrome (CFS), complex regional pain syndrome (CRPS), Guillain-Barré syndrome (GBS) or postural orthostatic tachycardia syndrome (POTS), but there was one case of premature ovarian failure (POF) in the HPV vaccine group (see Table 9 and Additional file 4).

**Table 9:** Benefits and harms of the HPV vaccines: summary of harms of special interest and post hoc exploratory harm analyses

Summary of harms of special interest and post hoc exploratory harm analyses^a^	Serious harms			New onset diseases^d^			General harms^e^		
	HPV vaccine (*N* = 47,075)	Comparator (*N* = 48,595)	Risk ratio^f^[95% CI]	HPV vaccine (*N* = 47,075)	Comparator (*N* = 48,595)	Risk ratio^f^ [95% CI]	HPV vaccine (*N* = 47,075)	Comparator (*N* = 48,595)	Risk ratio^f^ [95% CI]
Harms of special interest (MedDRA-preferred terms, *n* = participants)									
Anaphylaxis	2	4	0.59 [0.13, 2.82]	11	8	1.18 [0.48, 2.91]	0	0	Not applicable
Chronic fatigue syndrome (CFS)	0	0	Not applicable	0	0	Not applicable	0	0	Not applicable
Chronic regional pain syndrome (CRPS)	0	0	Not applicable	0	0	Not applicable	0	0	Not applicable
Guillain-Barré syndrome (GBS)	0	0	Not applicable	0	0	Not applicable	0	0	Not applicable
Postural orthostatic tachycardia syndrome (POTS)	0	0	Not applicable	0	0	Not applicable	0	0	Not applicable
Premature ovarian failure (POF)	0	0	Not applicable	1	0	3.00 [0.12, 73.48]	0	0	Not applicable
Syncope	4	3	0.94 [0.23, 3.81]	62	60	1.03 [0.58, 1.84]	7	7	0.77 [0.25, 2.34]
Post hoc exploratory analyses of VigiBase® harm clusters^b^									
Expected systemic reactions	25	11	1.96 [0.96, 3.98]	1465	1358	1.03 [0.93, 1.14]	10,926	9948	Not applicable^g^
Allergic/hypersensitivity reactions	2	2	0.96 [0.14, 6.52]	284	279	1.05 [0.82, 1.35]	1912	1469	1.30 [1.18, 1.45]
Vasovagal reactions	9	5	1.31 [0.50, 3.46]	232	212	1.06 [0.78, 1.44]	173	123	1.20 [0.93, 1.55]
Post hoc exploratory analyses of CRPS and POTS^c^									
Harms judged as ‘definitely associated’ with CRPS	95	57	1.54 [1.11, 2.14]	5079	4790	1.04 [0.98, 1.10]	27,899	23,223	Not applicable^g^
Harms judged as ‘definitely associated’ with POTS	56	26	1.92 [1.21, 3.07]	3675	3352	1.08 [1.01, 1.15]	18,207	16,288	Not applicable^g^

^a^See Additional file 4 sections 13 and 14 for meta-analyses of the harms of special interest and post hoc exploratory harm analyses. There was no applicable fatal harm of special interest. It was not feasible to present this summary table for the 16 subgroups (based on age group, gender, type of HPV vaccine and comparator) of the 24 included clinical study reports. As we did not obtain complete case report forms or individual participant data, we could not assign harms to individual participants

^b^As the included studies’ harm assessments were at risk of low internal and external validity (see Table 1 and the “Discussion” section), we compared the three largest harm clusters reported from pharmacovigilance up to 1 January 2015 to the World Health Organisation’s (WHO) VigiBase with the clinical study report data. We did this to see if the pharmacovigilance data were similar to the study data. VigiBase’s largest HPV vaccine harm cluster (expected systemic reactions) consists of ‘headache, nausea, pyrexia, dizziness and vomiting’. VigiBase’s second largest HPV vaccine harm cluster (allergic/hypersensitivity reactions) consists of ‘pruritis, urticaria, rash and erythema’. VigiBase’s third largest HPV vaccine harm cluster (vasovagal reactions) consists of ‘syncope, dizziness, loss of consciousness, pallor and seizure’. As we synthesised individual MedDRA-preferred term classified harms, our post hoc exploratory analyses of VigiBase harm clusters may therefore include a participant more than once in each separate analysis

^c^We asked a physician with clinical expertise in POTS and CRPS to assess the reported MedDRA terms as ‘definitely’, ‘probably’, ‘probably not’ or ‘definitely not’ associated with the syndromes. The physician was blinded to the allocation groups and outcome data. The data was synthesised for those MedDRA-preferred terms that the physician judged ‘definitely’ associated with POTS or CRPS. As we synthesised individual MedDRA-preferred terms, our post hoc exploratory analyses of CRPS and POTS may include a participant more than once in each separate analysis

^d^New onset diseases were compiled of the harm categories ‘medically significant conditions’ (for Cervarix) and ‘new medical history’ (for the HPV 16 vaccine, Gardasil and Gardasil 9)

^e^General harms were compiled of the harm categories ‘solicited general adverse events’, ‘unsolicited general adverse events’ (for Cervarix) and ‘systemic adverse events’ (for Gardasil, Gardasil 9 and the HPV 16 vaccine)

^f^Risk ratios were calculated with the random-effects inverse variance method

^g^Some numerators exceeded the denominators making the result nonsensical. Therefore, we did not perform meta-analyses

#### Post hoc exploratory harm analyses of special interest

The data from the included clinical study reports that corresponded to the three largest harm clusters reported from pharmacovigilance were associated with general harms, but not serious harms or new onset diseases. The serious harms that were judged ‘definitely associated’ with POTS or CRPS by the blinded physician were increased by the HPV vaccines, both for POTS (56 vs. 26, RR 1.92 [95% CI 1.21 to 3.07], NNH 1073, *P* = 0.006, *I*^2^ = 0%) and CRPS (95 vs. 57, RR 1.54 [95% CI 1.11 to 2.14], NNH 906, *P* = 0.010, *I*^2^ = 0%). The new onset diseases that were judged ‘definitely associated’ with POTS were also increased by the HPV vaccines (3675 vs. 3352, RR 1.08 [95% CI 1.01 to 1.15], NNH 144, *P* = 0.03, I^2^ = 29%) (see Table 9 and Additional file 4).

### Subgroup analyses

Younger HPV vaccinated participants were more protected against moderate HPV-related intraepithelial neoplasia or worse than older participants (age 15 to 29: 784 vs. 1079, RR 0.71 [95% CI 0.61 to 0.83]; age 21 to 72: 168 vs. 160, RR 1.04 [95% CI 0.84 to 1.29]; ratio of relative risk [RRR] 1.46 [1.12 to 1.91]) and also experienced fewer fatal harms than older participants (age 15 to 27: 24 vs. 32, RR 0.77 [95% CI 0.45, 1.33]; age 21 to 72: 21 vs. 6, RR 3.13 [95% CI 1.29 to 7.61]; RRR 0.25 [95% CI 0.09 to 0.70]), but there were no differences for serious nervous system disorders (age 10 to 35: 53 vs. 35, RR 1.46 [95% CI 0.95 to 2.25]; age 21 to 72: 19 vs. 11, RR 1.56 [95% CI 0.75 to 3.25]; RRR 0.93 [95% CI 0.40 to 2.19]), serious harms that were judged ‘definitely associated’ with of CRPS (age 9 to 35: 76 vs. 48, RR 1.48 [95% CI 1.03 to 2.12]; age 21 to 72: 19 vs. 9, RR 2.11 [95% CI 0.67 to 6.69]; RRR 0.70 [95% CI 0.21 to 2.34]) or serious harms that were judged ‘definitely associated’ with POTS (age 12 to 35: 43 vs. 21, RR 1.86 [95% CI 1.10, 3.15]; age 21 to 72: 13 vs. 5, RR 2.22 [95 CI 0.76 to 6.47]; RRR 0.84 [95% CI 0.25 to 2.76]) (see Additional file 4; note that the subgroup analyses used overlapping age groups due to the different age groups included in the trials). No significant subgroup differences were identified for subgroup analyses based on gender and control treatment.

### Random-effects vs. fixed-effect

We found similar results with the fixed-effect model but with narrower confidence intervals, as the between-trial variance is not included in this model.

## Discussion

Our systematic review of 24 clinical study reports with 95,670 participants showed that the HPV vaccines within 4 years of follow-up decreased HPV-related carcinoma in situ, which have a high likelihood of progressing to cancer [1], and HPV-related treatment procedures, but the vaccines also increased serious nervous system disorders (exploratory analysis) and general harms. Younger participants who are those primarily intended to receive HPV vaccination [1] were more protected against HPV-related neoplasia and had fewer fatal harms.

### Strengths

Our review was based on study programmes, randomised trials reported in clinical study reports, clinically important pre-specified outcomes, intention to treat analyses, absolute risk estimates and a conservative statistical method based on the random-effects model. There was no heterogeneity for serious nervous system disorders or for the post hoc exploratory harm analyses of serious signs and symptoms judged ‘definitely associated’ with POTS and CRPS by a blinded physician with clinical expertise.

### Limitations

Insufficient trial data access, incomplete reporting, data fragmentation and limited trial follow-up periods were major limitations. It took 3 years to obtain an incomplete subset of the eligible clinical study reports; a process which we have documented in detail elsewhere [38]. Our review is therefore limited by reporting bias—the bias that we aimed to reduce [37]. We did not obtain any periodical safety update reports before our data lock. The inclusion of the remaining participants from the 26 studies with no available clinical study reports included a fifth of the total eligible participants, which could have influenced our review, as some of our results had *P* values around our cut-off of 0.05 and confidence intervals that were wide.

We performed multiple comparisons: 166 meta-analyses of which 31 (19%) showed statistical significance for the total risk ratio estimate. With our *P* value cut-off of 0.05, about eight (166*0.05) or a fourth (8/31) of the significant results are likely to have occurred by chance. We did not use Bonferroni (or similar) corrections [40], as one of our primary outcomes was serious harms, which were affected by incomplete reporting (see Table 1) and lack of saline placebo controls (see Additional file 2).

The 24 included clinical study reports only included one Gardasil 9 trial (V503–006) that was small and did not investigate histological outcomes. Many countries are currently implementing Gardasil 9 as a two-dose regimen in their vaccination programme instead of Cervarix or Gardasil [1]. Two doses of Gardasil 9 may induce fewer harms than three doses, but Gardasil 9 may induce more harms than Gardasil. For example, in the clinical study report that we obtained of phase 3 multicentre trial V503-001/NCT00543543 (not eligible for our systematic review) of 7106 and 7109 healthy females age 16–26 randomised to receive three doses Gardasil 9 or Gardasil, there were more serious harms (233 vs. 183, RR 1.27 [95% CI 1.05 to 1.54], NNH 151, *P* = 0.010; reported from day 0 to 390) and general harms (‘systemic adverse events’: 2086 vs. 1929, RR 1.08 [95% CI 1.03 to 1.14], NNH 75, *P* = 0.003; reported 0–14 days post-vaccination) in the Gardasil 9 group. A 0.5-ml dose of Gardasil 9 contains more virus-like particles (270 μg vs. 100 μg) and aluminium-containing adjuvant (500 μg vs. 225 μg) compared to a 0.5-ml dose of Gardasil, which could explain the harm differences. Although Gardasil 9 targets five more HPV types than Gardasil, Gardasil 9 did not decrease CIN2^+^ more than Gardasil during trial V503-001’s 42-month follow-up (325 vs. 326, RR 1.00 [95% CI 0.86 to 1.16], *P* = 0.97).

A substantial part of our results should be interpreted carefully due to high heterogeneity. We expected the high heterogeneity for several results (e.g. for HPV-related carcinoma in situ), as the included trials comprised 16 different subgroups—based on the type of HPV vaccine, comparator, age and gender. All meta-analyses were divided according to the 16 subgroups to provide heterogeneity measures (see Additional file 4), but the nationality of the participants and regional practices of HPV-related screening and treatment procedures may also have contributed to the heterogeneity.

#### Limitations of benefit assessment

Only 10 HPV-related cancers occurred in the follow-up periods. Extended follow-up was not possible for 75% of the comparator participants (36,344/48,595), as they were offered HPV vaccination at trial completion.

We only included benefit results of intention to treat analyses, which also included participants that were enrolled after they had been infected with HPV. The HPV vaccines have no documented effect on HPV-related neoplasia caused by previous infections [1]. Our benefit results may therefore be skewed toward the null compared to real-life settings where mainly 12-year-old adolescents—that are expected to not be previously HPV-infected—are HPV vaccinated. Getting vaccinated before sexual debut is likely to improve the HPV vaccines’ benefits, but no included trial investigated histological outcomes for participants that were vaccinated under the age of 15.

Three trials—HPV-008, V501-013 and V501-015 that contained 38% (36,266/95,670) of the analysed participants—were stopped early when HPV type 16/18-related cervical intraepithelial neoplasia or worse (CIN2^+^) was significantly reduced for their HPV vaccine per-protocol populations. On average, trials stopped early for benefits exaggerate effects by 29% compared to completed trials of the same intervention [41]. When the three trials were excluded from our CIN2^+^ meta-analysis, CIN2^+^ was not significantly decreased (184 vs. 200, RR 0.85 [95% CI 0.54 to 1.33], *P* = 0.47, *I*^2^ = 77%; see Additional file 4).

One clinical study report (HPV-015) only reported CIN2^+^, although there were three cases of HPV-related cancers in the HPV vaccine group and one in the comparator group (see Additional file 4). These cancers were listed as serious harms and were not mentioned elsewhere in the clinical study report. For transparency, it would have been more appropriate to report each histological outcome (cancer, carcinoma in situ, moderate intraepithelial neoplasia, etc.) than only a composite surrogate outcome such as CIN2^+^.

No trial tested the HPV vaccines’ protection against cervical cancer without using cervical screening. This may reduce external validity, as some studies show that HPV-vaccinated women may tend to avoid cervical screening [42]; although other studies have not shown a clear tendency [43]. The trial personnel often performed cervical screening together with colposcopy every 6 months, and the included participants were often women aged 15–26. In clinical practice, cervical screening is usually performed every 3 to 5 years and recommended after age 25 [44], as most CIN2^+^ lesions in women under age 30 regress spontaneously, which may justify active surveillance rather than immediate intervention [45].

No trial used mandatory biopsies, which may reduce internal validity. For example, the precursor lesion of cervical adenocarcinoma is difficult to detect on colposcopy, but easier to recognise on a biopsy [46]. The incidence of cervical adenocarcinoma is increasing and may more often be HPV negative compared to cervical squamous carcinoma [46], but only 5% (40/857) of the reported cervical carcinoma in situ cases in the included studies were adenocarcinoma in situ (see Table 4).

We did not pre-specify genital warts as an outcome, but the HPV vaccines reduced external genital lesions and there is strong evidence that the HPV vaccines—especially Gardasil and Gardasil 9 that target the HPV types 6 and 11—decrease the incidence of genital warts [47].

#### Limitations of harm assessment

Only Merck clinical study reports reported aggregate numbers for participants with MedDRA system organ classified harms, and only for new onset diseases and general harms. The synthesis of MedDRA system organ classes for all GlaxoSmithKline clinical study reports and for serious harms for Merck clinical study reports may therefore include a participant more than once. As a result, we consider these analyses exploratory.

Serious harms were incompletely reported for 72% of the participants (68,610/95,670; see Table 1 and Additional file 2). There were 2.8 times more serious harms reported in the clinical study reports that reported serious harms for the whole trial period (1838/27,493 vs. 923/38,356). As an example, trial HPV-008 of Cervarix that had reported all serious harms during its 48 months follow-up reported 10 times more participants with serious harms compared to V501-015 of Gardasil that only reported serious harms 14 days post-vaccination (1664/18,644 vs. 102/12,167). In the cluster-randomised trial, HPV-040, 88% (28,473 of 32,176) of the participants were not included for serious harms reporting (see Table 1 and Additional file 2).

The use of active comparators may have underestimated harms related to the HPV vaccines [38]. The aluminium-containing comparators were used, as they provided a similar appearance to that of the HPV vaccines, which enhanced blinding and decreased the risk of performance and detection bias. A single trial—V503-006, of Gardasil 9—used a saline placebo in 306 participants who had previously been vaccinated with Gardasil. It is unlikely that those who had experienced harms following previous Gardasil vaccination would have participated in the Gardasil 9 trial, so the trial’s harm results are not reliable. The trial’s blinding procedure was adequate to ensure low risk of performance and detection bias and could have been used in other trials (see Additional file 2).

Although the manufacturers consider the aluminium-containing comparators to be safe, 52% of the participants (49,301/95,670) were only included in the trials if they had never received the aluminium-containing comparators before. GlaxoSmithKline state that their aluminium-containing comparator induces myalgia (“higher incidences of myalgia might namely be attributable to the higher content of aluminium in the HPV vaccine [450 micrograms Al(OH)_3_] than the content of aluminium in the HAV [hepatitis A] vaccine [225 micrograms Al(OH)_3_]” [48]), which we found was increased by the HPV vaccines (see Table 7).

The clinical study reports, their informed consent forms and corresponding journal publications (for example, V501-013 [49] and V501-015 [50]) often used the term placebo (which is a substance with no active effect) to describe the active aluminium-based comparators.

Two thirds of the participants (63,468/95,670) were only included in the trials if they had no history of immunological or nervous system disorders (see Additional file 2). Such disorders are not listed as warnings or contraindications on the package inserts of the approved HPV vaccines [8–10]. The degree of harms might therefore be higher in clinical practice than in the trials. The HPV vaccines did not increase the three largest HPV vaccine-related VigiBase® harms clusters for serious harms and new onset diseases (see the “Methods” section, Table 9 and Additional file 4), which may reflect the differences between real-life and the trials’ settings and entry criteria.

The exploratory analyses of MedDRA system organ classes may have included a participant more than once. For serious nervous system disorders, this is unlikely, as there were only 118 participants with such disorders (reported as individual MedDRA-preferred terms) for 61,331 participants (see Additional file 4). We note, however, that the serious nervous system disorders consisted of very heterogenous harms, for example, ‘anoxic encephalopathy’, ‘moyamoya disease’ and ‘vertebral artery dissection’.

The serious harm analyses of MedDRA-preferred terms associated with POTS and CRPS may also have included a participant more than once, although this is unlikely as there only were 82 participants with a POTS sign/symptom for 60,058 participants and 152 participants with a CRPS sign/symptom for 60,915 participants. The selection of MedDRA-preferred terms associated with POTS and CRPS was subjective, not verified by other assessors and included some signs/symptoms that do not align well with the diagnostic criteria of POTS or CRPS [51, 52], for example, ‘constipation’, ‘vision blurred’ and ‘vomiting’. Other blinded assessors would possibly assign MedDRA-preferred terms differently, as there were over 3000 different included MedDRA-preferred terms. The post hoc exploratory POTS and CRPS analyses were based on randomised trial data where serious harms were underreported and likely underestimated, but since no complete serious harm narratives or complete case report forms were available, the analyses could not take symptom duration, symptom clustering or the diagnostic criteria into account. Therefore, the analyses do not prove that the HPV vaccines cause POTS and CRPS, but they do provide a signal, which makes it important to carry out independent analyses of POTS and CRPS based on the complete data set with individual participant data.

### Similar studies

In May 2018, a Cochrane review of the HPV vaccines that included 26 trials with 73,428 female participants concluded that the HPV vaccines decrease precursors to cervical cancer and do not increase serious or general harms [3]. The Cochrane review had similar inclusion criteria to our review, but it was mainly based on journal publications and only included phase II and III trials. In comparison, we identified 50 possibly eligible studies for which we obtained clinical study reports for 22 trials and two follow-up studies and included 30% more participants (95,670) than the Cochrane review. We found that the HPV vaccines decrease precursors to HPV-related cancer and treatment procedures but increase serious nervous system disorders (exploratory analyses) and general harms. Another recent review on males [53] and most large epidemiological studies have found no serious harms associated with the HPV vaccines [16–20].

## Conclusion

At 4 years follow-up, the HPV vaccines decreased HPV-related precursors to cervical cancer and treatment procedures but increased serious nervous system disorders (exploratory analysis) and general harms. As the included trials were primarily designed to assess benefits and not adequately designed to assess harms, the extent to which the benefits outweigh the harms is unclear. Limited access to clinical study reports and trial data with case report forms prevented a thorough assessment. An independent assessment of the complete individual participant data is needed. If granted access to the complete data set with individual participant data, we will update this systematic review. A large industry-independent multicentre trial of two doses of Gardasil 9 vs. saline placebo would likely be informative in identifying a more accurate benefit-harm balance, but we recognise that such a trial will be considered unethical in most settings.

## Additional files

Additional file 1: Benefits and harms of the HPV vaccines—PRISMA 2009 checklist. (DOCX 31 kb)

Additional file 2: Benefits and harms of the HPV vaccines—characteristics of included studies. (DOCX 121 kb)

Additional file 3: Benefits and harms of the HPV vaccines—list of excluded studies. (DOCX 63 kb)

Additional file 4: Benefits and harms of the HPV vaccines—meta-analyses. (PDF 26103 kb)

## Abbreviations

AIN: Anal intraepithelial neoplasia
CFS: Chronic fatigue syndrome
CIN: Cervical intraepithelial neoplasia
CRPS: Chronic regional pain syndrome
EGL: External genital lesion
EMA: European Medicines Agency
FDA: Food and Drug Administration
GBS: Guillain-Barré syndrome
GSK: GlaxoSmithKline
HPV: Human papillomavirus
ICH: International Council for Harmonisation of Technical Requirements for Pharmaceuticals for Human Use
MedDRA: Medical Dictionary for Regulatory Activities
Merck: Merck and Co., Inc. or Merck Sharp and Dohme outside the USA and Canada
MSC: Medically significant condition
NMH: New medical history
NNH: Number needed to harm
NNV: Number needed to vaccinate
PICO: Patient, intervention, comparator and outcome
PIN: Penile intraepithelial neoplasia
POF: Premature ovarian failure
POTS: Postural orthostatic tachycardia syndrome
PRISMA: Preferred Reporting Items for Systematic Reviews and Meta-Analyses
PROSPERO: International Prospective Register of Systematic Reviews
SGAE: Solicited general adverse event
SYAE: Systemic adverse event
UGAE: Unsolicited general adverse event
VaIN: Vaginal intraepithelial neoplasia
VIN: Vulvar intraepithelial neoplasia
WHO: World Health Organisation
