# Supplementary material for: Benefits and harms of the human papillomavirus (HPV) vaccines: systematic review with meta-analyses of trial data from clinical study reports
Source: Syst Rev. 2020 Feb 28;9:43. doi: 10.1186/s13643-019-0983-y (PMC7047375; doi:10.1186/s13643-019-0983-y)
Supplement: Supplementary file 2 — Benefits and harms of the HPV vaccines—characteristics of included studies. (DOCX 121 kb) [file 13643_2019_983_MOESM2_ESM.docx]

**Additional file 2**

**Benefits and harms of the HPV vaccines:**

**characteristics of included studies with supporting statements for risk of bias judgements**

Summary table

|  | **HPV vaccine manufacturer** | **Study programme ID** | **N=months follow-up** | **N=participants** | **HPV vaccine** | **Comparator** | **Publication** |
| --- | --- | --- | --- | --- | --- | --- | --- |
| 1 | GlaxoSmithKline | HPV-001 | 27 | 1,113 | Cervarix | Al(OH)_3_ | Harper DM et al. Efficacy of a Bivalent L1 Virus-like Particle Vaccine in Prevention of Infection with Human Papillomavirus Types 16 and 18 in Young Women: A Randomized Controlled Trial. *Lancet*. 2004 Nov 13-19;364(9447):1757-65. doi: 10.1016/S0140-6736(04)17398-4. |
| 2 | GlaxoSmithKline | HPV-003 | 12 | 61 | Cervarix | Al(OH)_3_ | *Not published* |
| 3 | GlaxoSmithKline | HPV-008 | 48 | 18,644 | Cervarix | Havrix | Paavonen J et al. Efficacy of human papillomavirus (HPV)-16/18 AS04-adjuvanted vaccine against cervical infection and precancer caused by oncogenic HPV types (PATRICIA): final analysis of a double-blind, randomized study in young women. *Lancet*. 2009 Jul 25;374(9686):301-14. doi: 10.1016/S0140-6736(09)61248-4. |
| 4 | GlaxoSmithKline | HPV-013 | 12 | 2,067 | Cervarix | Havrix | Medina DM et al. Safety and immunogenicity of the HPV-16/18 AS04-adjuvanted vaccine: a randomized, controlled trial in adolescent girls. *J Adolesc Health*. 2010 May;46(5):414-21. doi: 10.1016/j.jadohealth.2010.02.006. |
| 5 | GlaxoSmithKline | HPV-015 | 36 | 5,753 | Cervarix | Al(OH)_3_ | Skinner S et al. Efficacy, safety, and immunogenicity of the human papillomavirus 16/18 AS04-adjuvanted vaccine in women older than 25 years: 4-year interim follow-up of the phase 3, double-blind, randomized controlled VIVIANE study. *Lancet*. 2014 Dec 20;384(9961):2213-27. doi: 10.1016/S0140-6736(14)60920-X. |
| 6 | GlaxoSmithKline | HPV-023 | 36 | (433*) | Cervarix | Al(OH)_3_ | Naud PS et al. Sustained efficacy, immunogenicity, and safety of the HPV-16/18 AS04-adjuvanted vaccine, *Human Vaccines & Immunotherapeutics*. 2014 Jun 19;10:8. doi: 10.4161/hv.29532. |
| 7 | GlaxoSmithKline | HPV-029 | 12 | 541 | Cervarix | Twinrix | Pedersen C et al. Randomized trial: immunogenicity and safety of coadministered human papillomavirus-16/18 AS04-adjuvanted vaccine and combined hepatitis A and B vaccine in girls. *J Adolesc Health*. 2012 Jan;50(1):38-46. doi: 10.1016/j.jadohealth.2011.10.009. |
| 8 | GlaxoSmithKline | HPV-030 | 12 | 493 | Cervarix | Engerix-B | Schmeink CE et al. Co-administration of human papillomavirus-16/18 AS04-adjuvanted vaccine with hepatitis B vaccine: randomized study in healthy girls. *Vaccine*. 2011 Nov 15;29(49):9276-83. doi: 10.1016/j.vaccine.2011.08.037. |
| 9 | GlaxoSmithKline | HPV-031 | 7 | 354 | Cervarix | Al(OH)_3_ | Bhatla N et al. Immunogenicity and safety of human papillomavirus-16/18 AS04-adjuvanted cervical cancer vaccine in healthy Indian women. *J Obstet Gynaecol Res*. 2010 Feb;36(1):123-32. doi: 10.1111/j.1447-0756.2009.01167.x. |
| 10 | GlaxoSmithKline | HPV-032 | 7 | 1,040 | Cervarix | Aimmugen | Konno R et al. Efficacy of human papillomavirus type 16/18 AS04-adjuvanted vaccine in Japanese women aged 20 to 25 years: final analysis of a phase 2 double-blind, randomized controlled trial. *Int J Gynecol Cancer*. 2010 Jul;20(5):847-55. doi: 10.1111/IGC.0b013e3181da2128. |
| 11 | GlaxoSmithKline | HPV-033 | 7 | 321 | Cervarix | Havrix | Kim YJ et al. Vaccination with a human papillomavirus (HPV)-16/18 AS04-adjuvanted cervical cancer vaccine in Korean girls aged 10-14 years. *J Korean Med Sci*. 2010 Aug;25(8):1197-204. doi: 10.3346/jkms.2010.25.8.1197. |
| 12 | GlaxoSmithKline | HPV-035 | 7 | 300 | Cervarix | Al(OH)_3_ | Ngan HY et al. Human papillomavirus-16/18 AS04-adjuvanted cervical cancer vaccine: immunogenicity and safety in healthy Chinese women from Hong Kong. *Hong Kong Med J*. 2010 Jun;16(3):171-9. |
| 13 | GlaxoSmithKline | HPV-038 | 7 | 225 | Cervarix | Al(OH)_3_ | Kim SC et al. Human papillomavirus 16/18 AS04-adjuvanted cervical cancer vaccine: immunogenicity and safety in 15-25 years old healthy Korean women. *J Gynecol Oncol*. 2011 Jun 30; 22(2): 67–75. doi: 10.3802/jgo.2011.22.2.67. |
| 14 | GlaxoSmithKline | HPV-040 | 72 | 32,176 | Cervarix | Engerix-B | Lehtinen M et al. Safety of the human papillomavirus (HPV)-16/18 AS04-adjuvanted vaccine in adolescents aged 12–15 years: Interim analysis of a large community-randomized controlled trial. *Hum Vaccin Immunother*. 2016 Dec; 12(12): 3177–3185. doi: 10.1080/21645515.2016.1183847. |
| 15 | GlaxoSmithKline | HPV-058 | 12 | 750 | Cervarix | Al(OH)_3_ | Zhu F et al. Immunogenicity and safety of the HPV-16/18 AS04-adjuvanted vaccine in healthy Chinese girls and women aged 9 to 45 years. *Hum Vaccin Immunother*. 2014;10(7):1795-806. doi: 10.4161/hv.28702. |
| 16 | GlaxoSmithKline | HPV-063 | 12 | (752*) | Cervarix | Aimmugen | Konno R et al. Efficacy of the human papillomavirus (HPV)-16/18 AS04-adjuvanted vaccine against cervical intraepithelial neoplasia and cervical infection in young Japanese women. *Hum Vaccin Immunother*. 2014;10(7):1781-94. doi: 10.4161/hv.28712. |
| 17 | GlaxoSmithKline | HPV-069 | 12 | 1,212 | Cervarix | Engerix-B | Zhu F et al. Immunogenicity and safety of the HPV-16/18 AS04-adjuvanted vaccine in healthy Chinese girls and women aged 9 to 45 years. *Hum Vaccin Immunother*. 2014;10(7):1795-806. doi: 10.4161/hv.28702. |
| 18 | Merck | V501-005 | 48 | 2,409 | HPV 16 vaccine | AAHS | Koutsky LA et al. A controlled trial of a human papillomavirus type 16 vaccine. *N Engl J Med*. 2002 Nov 21;347(21):1645-51. doi: 10.1056/NEJMoa020586. |
| 19 | Merck | V501-013 | 45 | 5,455 | Gardasil | AAHS | Garland SM. Quadrivalent vaccine against human papillomavirus to prevent anogenital diseases. *N Engl J Med*. 2007 May 10;356(19):1928-43. doi: 10.1056/NEJMoa061760. |
| 20 | Merck | V501-015 | 36 | 12,167 | Gardasil | AAHS | The FUTURE II Study Group. Quadrivalent vaccine against human papillomavirus to prevent anogenital diseases. *N Engl J Med*. 2007 May 10;356(19):1928-43. doi: 10.1056/NEJMoa061760 |
| 21 | Merck | V501-018 | 18 | 1,781 | Gardasil | Carrier solution | Reisinger KS et al. Safety and persistent immunogenicity of a quadrivalent human papillomavirus types 6, 11, 16, 18 L1 virus-like particle vaccine in preadolescents and adolescents: a randomized controlled trial. *Pediatr Infect Dis J*. 2007 Mar;26(3):201-9. |
| 22 | Merck | V501-019 | 48 | 3,819 | Gardasil | AAHS | Muñoz N et al. Safety, immunogenicity, and efficacy of quadrivalent human papillomavirus (types 6, 11, 16, 18) recombinant vaccine in women aged 24-45 years: a randomized, double-blind trial. *Lancet*. 2009 Jun 6;373(9679):1949-57. doi: 10.1016/S0140-6736(09)60691-7. |
| 23 | Merck | V501-020 | 36 | 4,065 | Gardasil | AAHS | Giuliano AR et al. Efficacy of quadrivalent HPV vaccine against HPV Infection and disease in males. *N Engl J Med*. 2011 Feb 3;364(5):401-11. doi: 10.1056/NEJMoa0909537. |
| 24 | Merck | V503-006 | 7 | 924 | Gardasil 9 | Saline placebo | Garland SM et al. Safety and immunogenicity of a 9-valent HPV vaccine in females 12-26 years of age who previously received the quadrivalent HPV vaccine. *Vaccine*. 2015 Nov 27;33(48):6855-64. doi: 10.1016/j.vaccine.2015.08.059. |

*Studies HPV-023 and HPV-063 were follow-up studies of HPV-001 and HPV-032.

HPV-001 (NCT00689741)

*Title*: “A double blind, placebo-comparator led, randomized, pilot phase IIB study of the efficacy of a human papillomavirus (HPV) HPV-16/18 AS04 vaccine in the prevention of HPV-16 and/or HPV-18 cervical infection in healthy adolescent and young adult women in North America and Brazil.”

*Characteristics*:

| Methods | Randomized, parallel group, double-blind trial that was 27 months long. |
| --- | --- |
| Participants | 1,113 healthy females (560 in the HPV vaccine group and 553 in the comparator group), age 15-26, from USA, Canada and Brazil. Participants were excluded from the trial if they previously had received any of the adjuvants that were part of the HPV vaccine or comparator or had a history of any neurological disease. |
| Interventions | Cervarix (0.5 millilitre) vs. aluminium hydroxide (Al[OH]_3_, 0.5 milligram in 0.5 millilitre saline) given intramuscularly at 0, 1 and 6 months. The batch numbers of the HPV vaccine and comparator were redacted. |
| Outcomes | All-cause mortality, fatal and serious harms, new onset diseases (reported as ‘medically significant conditions’ for the whole study period) and general harms (reported as ‘solicited’ and ‘unsolicited’ general harms 7- and 30-days post-vaccination, respectively). No benefit outcomes were eligible. Serious harms were not reported for individual MedDRA categories. |
| Notes | The maximum blinding time was 27 months and there was an optional extension phase of the trial. |

*Risk of Bias assessment*:

| Domain | Author’s judgment | Supporting statement |
| --- | --- | --- |
| Random sequence generation (selection bias) | Low risk of bias | "Randomization was performed via a SAS program written by GSK Biologicals using a procedure 'Ranuni' and using an Internet-based central randomization system (SBIR). The randomization was stratified by age and region." |
| Allocation concealment (selection bias) | Low risk of bias | “…the randomisation system determined the vaccine number to be used for the subject. The vaccine number was used as patient identification number (PID) for all data collected on the subject under the study. The investigator (or designate) used the eCRF of the RDE system to document the patient identification number (PID).” |
| Blinding of participants and personnel (performance bias) | Low risk of bias | "This was a double-blind study. The investigator and associated study personnel were unaware of the treatment assignments for the subjects. The study statistician at GSK Biologicals, Rixensart, had access to the randomization schedule. Once the final subject was randomized, the study statistician filed all randomization schedule documents in a sealed envelope. The study remains blinded and all analyses have been performed by an external statistician." |
| Blinding of outcome assessment (detection bias) | Low risk of bias | "This was a double-blind study. The investigator and associated study personnel were unaware of the treatment assignments for the subjects. The study statistician at GSK Biologicals, Rixensart, had access to the randomization schedule. Once the final subject was randomized, the study statistician filed all randomization schedule documents in a sealed envelope. The study remains blinded and all analyses have been performed by an external statistician." |
| Incomplete outcome data (attrition bias) | High risk of bias | 958 of 1,113 participants completed follow-up (86%). Less than half of the case report forms were included. |
| Selective reporting (reporting bias) | High risk of bias | The outcome data contained redactions. |
| Other | High risk of bias | The trial was funded by GlaxoSmithKline and used inadequate design and reporting. |

HPV-003 (NCT not available)

*Title*: “A phase I/II study to evaluate the safety and immunogenicity of MEDI-517, a virus-like participle vaccine against human papillomavirus (HPV) types 16 and 18 [i.e., Cervarix], in healthy adult female volunteers who are HPV-16 or HPV-18 DNA positive.”

*Characteristics*:

| Methods | Randomized, parallel group, double-blind trial that was 12 months long. |
| --- | --- |
| Participants | 61 healthy females (31 in the HPV vaccine group and 30 in the comparator group) age 18-30 allocated to 27 different centres in the United States. Participants were excluded from the trial if they previously had received any of the adjuvants that were part of the HPV vaccine or comparator. |
| Interventions | Cervarix (0.5 millilitre) vs. aluminium hydroxide (Al[OH]_3_, 0.5 milligram in 0.5 millilitre saline) given intramuscularly at 0, 1 and 6 months. |
| Outcomes | All-cause mortality, fatal and serious harms and general harms (reported as ‘solicited’ and ‘unsolicited’ general harms 7- and 30-days post-vaccination, respectively). No benefit outcomes were eligible and new onset diseases were not reported. |
| Notes | None. |

*Risk of Bias assessment*:

| Risk of bias domain | Author’s judgment | Supporting statement |
| --- | --- | --- |
| Random sequence generation (selection bias) | Low risk of bias | Computer generated randomization list. |
| Allocation concealment (selection bias) | Low risk of bias | “The investigator assigned a participant identification number”. The number was computer generated. The study pharmacist passed the number to the investigator “but did not reveal treatment assignment to anyone.” |
| Blinding of participants and personnel (performance bias) | Low risk of bias | "This was a double-blind study. The investigator, all clinic staff (other than the study pharmacist), the volunteers and the sponsor's staff (other than the study statistician) were unaware of the treatment assignments of the volunteers." |
| Blinding of outcome assessment (detection bias) | Low risk of bias | "This was a double-blind study. The investigator, all clinic staff (other than the study pharmacist), the volunteers and the sponsor's staff (other than the study statistician) were unaware of the treatment assignments of the volunteers." |
| Incomplete outcome data (attrition bias) | High risk of bias | 47 of 61 participants completed follow-up (77%). The individual participant data was not included. |
| Selective reporting (reporting bias) | High risk of bias | The outcome data contained redactions. The trial only reported serious harms for the seven-month vaccination period and not for the five-month follow-up period. New onset diseases were not reported. |
| Other | High risk of bias | The trial was funded by GlaxoSmithKline and used inadequate design and reporting. |

HPV-008 (NCT00122681)

*Title*: “A Phase III, Double-blind, Randomized, Controlled, Multi-centre Study to Evaluate the Efficacy of GlaxoSmithKline. Biologicals. HPV-16/18 VLP AS04 Vaccine Compared to Hepatitis A Vaccine as Control in Prevention of Persistent HPV-16 or HPV-18 Cervical Infection and Cervical Neoplasia, Administered Intramuscularly According to a 0, 1, 6 Month Schedule in Healthy Females 15-25 Years of Age.”

*Characteristics*:

| Methods | Randomized, parallel group, double-blind trial that was 48 months long. |
| --- | --- |
| Participants | 18,644 healthy females (9,319 in the HPV vaccine group and 9,325 in the comparator group) age 15-25 allocated to 133 different centres in Australia, Belgium, Brazil, Canada, Finland, Germany, Italy, Mexico, Philippines, Spain, Taiwan, Thailand, the United Kingdom and the United States. Participants were excluded from the trial if they previously had received any of the adjuvants that were part of the HPV vaccine or comparator or had a history of any neurological disease. |
| Interventions | Cervarix (0.5 millilitre) vs. Havrix (hepatitis A vaccine, 0.5 millilitre) given intramuscularly at 0, 1 and 6 months. |
| Outcomes | All-cause mortality, mortality from and incidence of HPV-related cancers irrespective of HPV-type, incidence of histologically confirmed carcinoma in situ and moderate abnormal histology irrespective of HPV-type, HPV-related referral procedures, fatal and serious harms, new onset diseases (reported as ‘medically significant conditions’ for the whole study period) and general harms (reported as ‘solicited’ and ‘unsolicited’ general harms 7- and 30-days post-vaccination, respectively, for a subset of participants: 6,159). |
| Notes | The control group was vaccinated with the HPV vaccine at the end of follow-up (“The study IDMC recommended that unblinding and cross-over immunization of both treatment and comparator recipients with the HPV vaccine or licensed Havrix, as appropriate, be offered to subjects after completion of their end-of-study activities”). |

*Risk of Bias assessment*:

| Risk of bias domain | Author’s judgment | Supporting statement |
| --- | --- | --- |
| Random sequence generation (selection bias) | Low risk of bias | "A randomization list was generated at GSK Biologicals, Rixensart, using a standard SAS (Statistical Analysis System) program and was used to number the vaccines. A randomization blocking scheme (1: 1 ratio) was used to ensure that balance between treatments was maintained: a treatment number uniquely identified the vaccine doses to be administered to the same subject.” |
| Allocation concealment (selection bias) | Low risk of bias | “the treatment allocation was performed at the investigator sites using a central randomization system on Internet (SBIR) ... the system allocated a unique treatment number using a minimization algorithm taking into account the study site and the subject age range.” |
| Blinding of participants and personnel (performance bias) | Low risk of bias | "This study was performed in a double-blinded manner. Blinding was maintained for all subjects and investigators and their study staff participating in this study with regard to the individual subject treatment assignments allocated in this study (vaccine or comparator) and the HPV DNA PCR and serological results until all subjects had completed the study, except if the subjects had requested the non-emergency unblinding." |
| Blinding of outcome assessment (detection bias) | Low risk of bias | "This study was performed in a double-blinded manner. Blinding was maintained for all subjects and investigators and their study staff participating in this study with regard to the individual subject treatment assignments allocated in this study (vaccine or comparator) and the HPV DNA PCR and serological results until all subjects had completed the study, except if the subjects had requested the non-emergency unblinding." |
| Incomplete outcome data (attrition bias) | High risk of bias | 15,609 of 18,644 participants completed follow-up (84%). The individual participant data was not included. Reasons for selecting safety diary card subset population by centre were not stated. |
| Selective reporting (reporting bias) | High risk of bias | The outcome data contained redactions. |
| Other | High risk of bias | The trial was funded by GlaxoSmithKline and used inadequate design and reporting. |

HPV-013 (NCT00196924)

*Title*: “A Phase III, double-blind, randomized, comparator led study to evaluate the safety and immunogenicity of GlaxoSmithKline. Biologicals' HPV-16/18 AS04 vaccine administered intramuscularly according to a 0, 1, 6-month schedule in healthy female subjects aged 10-14 years.”

*Characteristics*:

| Methods | Randomized, parallel group, observer-blind trial that was 12 months long. |
| --- | --- |
| Participants | 2,067 healthy females (1,035 in the HPV vaccine group and 1,032 in the comparator group) age 10-14 allocated to 57 different centres in Australia, Colombia, Czech Republic, France, Germany, Honduras, Korea, Norway, Panama, Spain, Sweden and Taiwan. Participants were excluded from the trial if they previously had received any of the adjuvants that were part of the HPV vaccine or comparator, had a history of any neurological or immunological disorder. |
| Interventions | Cervarix (0.5 millilitre) vs. Havrix (hepatitis A vaccine, 0.5 millilitre) given intramuscularly at 0, 1 and 6 months. |
| Outcomes | All-cause mortality, fatal and serious harms, new onset diseases (reported as ‘medically significant conditions’ for the whole study period) and general harms (reported as ‘solicited’ and ‘unsolicited’ general harms 7- and 30-days post-vaccination, respectively). No benefit outcomes were eligible. |
| Notes | None. |

*Risk of Bias assessment*:

| Risk of bias domain | Author’s judgment | Supporting statement |
| --- | --- | --- |
| Random sequence generation (selection bias) | Low risk of bias | "A randomized list was generated at GSK Biologicals, Rixensart, Belgium, using a standard SAS® (Statistical Analysis system) program and was used to number the vaccines. A randomization blocking scheme (1: 1 ratio) was used to ensure that balance between treatments was maintained.” |
| Allocation concealment (selection bias) | Low risk of bias | “…the randomization system [SBIR] determined the treatment number to be allocated to the subject.” |
| Blinding of participants and personnel (performance bias) | High risk of bias | “…the different visual presentations of the HPV -16/18 vaccine and the HAV comparator vaccine (both vaccines contain different quantities of aluminium, which results in a different turbidity of the vaccines and may allow identification)…all subjects and study personnel not involved in preparation and administration of study vaccines were blinded to the individual subject treatment.” |
| Blinding of outcome assessment (detection bias) | Low risk of bias | "Study HPV-013 was conducted as observer-blinded.” |
| Incomplete outcome data (attrition bias) | High risk of bias | 2,027 of 2,067 participants completed follow-up (98%). The individual participant data was not included. |
| Selective reporting (reporting bias) | High risk of bias | The outcome data contained redactions. |
| Other | High risk of bias | The trial was funded by GlaxoSmithKline and used inadequate design and reporting. |

HPV-015 (NCT00294047)

*Title*: “A phase III, double-blind, randomized, comparator-led study to evaluate the safety, immunogenicity and efficacy of GlaxoSmithKline Biologicals' HPV-16/18 AS04 vaccine administered intramuscularly according to a three-dose schedule (0, 1, 6 month) in healthy adult female subjects aged 26 years and above.”

*Characteristics*:

| Methods | Randomized, parallel group, double-blind trial that was 36 months long. |
| --- | --- |
| Participants | 5,753 healthy females (2,882 in the HPV vaccine group and 2,871 in the comparator group) age 24-72 allocated to 76 different centres in Australia, Canada, Mexico, the Netherlands, Peru, Philippines, Portugal, Russia, Singapore, Thailand, the United Kingdom and the United States. Participants were excluded from the trial if they previously had received any of the adjuvants that were part of the HPV vaccine or comparator or had a history of any neurological or immunological disorder. |
| Interventions | Cervarix (0.5 millilitre) vs. aluminium hydroxide (Al[OH]_3_, 0.5 milligram in 0.5 millilitre saline) given intramuscularly at 0, 1 and 6 months. |
| Outcomes | All-cause mortality, HPV-related cancer and referral procedures, fatal and serious harms, new onset diseases (reported as ‘medically significant conditions’ for the whole study period) and general harms (reported as ‘solicited’ and ‘unsolicited’ general harms 7- and 30-days post-vaccination, respectively). HPV-015 only reported combined surrogate outcomes (e.g., CIN2^+^) for benefits. |
| Notes | None. |

*Risk of Bias assessment*:

| Risk of bias domain | Author’s judgment | Supporting statement |
| --- | --- | --- |
| Random sequence generation (selection bias) | Low risk of bias | "A randomization list was generated at GSK Biologicals, Rixensart, using a standard SAS® (Statistical Analysis System) program and was used to number the vaccines. A randomization blocking scheme (1:1 ratio) was used to ensure that balance between treatments was maintained." |
| Allocation concealment (selection bias) | Low risk of bias | “The treatment allocation at the investigator site was performed using a central randomisation system on internet (SBIR)…When a subject was eligible and informed consent was obtained, the person in charge of the vaccination accessed SBIR…the randomisation system determined the treatment number to be used for the subject.” |
| Blinding of participants and personnel (performance bias) | Low risk of bias | "This study was performed in a double-blinded manner: all subjects, investigators and study staff participating in this study were blinded to the individual subject treatment (HPV vaccine or comparator) and HPV DNA PCR results. GSK personnel directly involved in the conduct of the study were also blinded to the individual subject treatment. The study blind was maintained until the end of the study." |
| Blinding of outcome assessment (detection bias) | Low risk of bias | "This study was performed in a double-blinded manner: all subjects, investigators and study staff participating in this study were blinded to the individual subject treatment (HPV vaccine or comparator) and HPV DNA PCR results. GSK personnel directly involved in the conduct of the study were also blinded to the individual subject treatment. The study blind was maintained until the end of the study." |
| Incomplete outcome data (attrition bias) | High risk of bias | 3,785 of 5,753 participants completed follow-up (66%). The individual participant data was not included. |
| Selective reporting (reporting bias) | High risk of bias | The outcome data contained redactions. The trial only reported investigator judged serious harms in the follow-up (i.e., "From Visit 12 to Visit 17, only SAEs [serious harms] related to vaccination, other GSK medications or study procedures, any fatal SAE and AEs/SAEs leading to premature discontinuation of the study"). The pathologists categorized abnormal histology as, “…CIN2, CIN3, AIS, MALIGN [i.e., cancer],” but the trial only reported CIN2^+^, although there were three cases of HPV-related cancers in the HPV vaccine group and one in the comparator group. The cancers were tabulated as serious harms and not mentioned elsewhere in the trial report. |
| Other | High risk of bias | The trial was funded by GlaxoSmithKline and used inadequate design and reporting. |

HPV-023 (NCT00518336)

*Title*: “A blinded long-term follow-up study of the efficacy of candidate HPV-16/18 L1 VLP AS04 vaccine in young adult women in Brazil vaccinated in the phase IIb, double-blind, multicentre primary study HPV-001 and having participated in the follow-up study HPV-007.”

*Characteristics*:

| Methods | Randomized, parallel group, double-blind trial that was 36 months long. |
| --- | --- |
| Participants | 433 healthy females (222 in the HPV vaccine group and 211 in the comparator group) age 21-32 allocated to five different centres in Brazil. Participants were excluded from the trial if they previously had received any of the adjuvants that were part of the HPV vaccine or comparator or had a history of any neurological disease. |
| Interventions | Cervarix (0.5 millilitre) vs. aluminium hydroxide (Al[OH]_3_, 0.5 milligram in 0.5 millilitre saline) given intramuscularly at 0, 1 and 6 months. |
| Outcomes | All-cause mortality and fatal and serious harms. No benefit outcomes were eligible or happened (e.g., “During the first year of follow-up in study HPV-023, there were no new cases of VIN or VaIN associated with oncogenic types") during the follow-up. Serious harms were not reported for individual MedDRA categories. New onset diseases and general harms were not reported. |
| Notes | None. |

*Risk of Bias assessment*:

| Risk of bias domain | Author’s judgment | Supporting statement |
| --- | --- | --- |
| Random sequence generation (selection bias) | Low risk of bias | "No treatment was given in study HPV-023. The randomization that occurred in primary study HPV-001 was maintained in follow-up study HPV-007 and the current follow-up study HPV-023." |
| Allocation concealment (selection bias) | Low risk of bias | “…the randomisation system determined the vaccine number to be used for the subject. The vaccine number was used as patient identification number (PID) for all data collected on the subject under the study. The investigator (or designate) used the eCRF of the RDE system to document the patient identification number (PID).” |
| Blinding of participants and personnel (performance bias) | Low risk of bias | "Blinding was to be maintained for all subjects and investigators and their study staff participating in this study with regard to the individual subject treatment (vaccine or placebo) assignments allocated in study HPV-001. GSK personnel directly involved in the conduct of this study (e.g. site monitors, medical monitors, laboratory personnel, etc.) were also blinded to the subjects’ treatment assignments. The site monitors, medical monitors, as well as laboratory and data validation personnel did not have access to any individual unblinded subject data listings from study HPV-001 or HPV-007 from this cohort." |
| Blinding of outcome assessment (detection bias) | Low risk of bias | "Blinding was to be maintained for all subjects and investigators and their study staff participating in this study with regard to the individual subject treatment (vaccine or placebo) assignments allocated in study HPV-001. GSK personnel directly involved in the conduct of this study (e.g. site monitors, medical monitors, laboratory personnel, etc.) were also blinded to the subjects’ treatment assignments. The site monitors, medical monitors, as well as laboratory and data validation personnel did not have access to any individual unblinded subject data listings from study HPV-001 or HPV-007 from this cohort." |
| Incomplete outcome data (attrition bias) | High risk of bias | 428 of 433 participants completed follow-up (99%). The individual participant data was not included. |
| Selective reporting (reporting bias) | High risk of bias | The outcome data contained redactions. The trial reported serious harms for the follow-up period, but these were not included for individual MedDRA categories. New onset diseases and general harms were not reported. |
| Other | High risk of bias | The trial was funded by GlaxoSmithKline and used inadequate design and reporting. |

HPV-029 (NCT00578227)

*Title*: “A phase IIIb, randomized, open, multicentre study to evaluate the immunogenicity and safety of GlaxoSmithKline Biologicals’ HPV-16/18 L1 VLP AS04 vaccine co-administered with GlaxoSmithKline Biologicals’ inactivated hepatitis A and hepatitis B vaccine adsorbed (Twinrix® Paediatric) in healthy female subjects aged 9 - 15 years.”

*Characteristics*:

| Methods | Randomized, parallel group, open trial that was 12 months long. |
| --- | --- |
| Participants | 541 healthy females (270 in the HPV vaccine group and 271 in the comparator group) age 8-15 allocated to 21 different centres in Canada, Denmark, Hungary and Sweden. Participants were excluded from the trial if they previously had received any of the adjuvants that were part of the HPV vaccine or comparator or had a history of any immunological disorder. |
| Interventions | Cervarix (0.5 millilitre) vs. Twinrix (hepatitis A and hepatitis B vaccine, 0.5 millilitre) given intramuscularly at 0, 1 and 6 months. |
| Outcomes | All-cause mortality, fatal and serious harms, new onset diseases (reported as ‘medically significant conditions’ for the whole study period) and general harms (reported as ‘solicited’ and ‘unsolicited’ general harms 7- and 30-days post-vaccination, respectively). No benefit outcomes were eligible. |
| Notes | The control group was vaccinated with the HPV vaccine at the end of follow-up ("After completion of the safety follow-up and outside the study protocol, vaccination with Twinrix® Paediatric will be offered to the HPV group and vaccination with GSK Biologicals’ HPV-16/18 L1 VLP AS04 vaccine will be offered to the HAB group, if commercially available in the participating country”). |

*Risk of Bias assessment*:

| Risk of bias domain | Author’s judgment | Supporting statement |
| --- | --- | --- |
| Random sequence generation (selection bias) | Low risk of bias | "A randomization list was generated at GSK Biologicals, Rixensart, using a standard SAS (Statistical Analysis System) program, to number the vaccines. A randomization blocking scheme (1:1:1 ratio) was used to ensure that balance between treatments was maintained: a treatment number identified uniquely the vaccine doses administered to the same subject." |
| Allocation concealment (selection bias) | Low risk of bias | “The treatment allocation at the investigator site was performed using a central randomization system on Internet (SBIR) ... The person in charge of the vaccination accessed the randomization system on Internet.” |
| Blinding of participants and personnel (performance bias) | High risk of bias | "This was an open study and therefore the subject and investigator were aware of the group allocated and the treatment given." |
| Blinding of outcome assessment (detection bias) | High risk of bias | "This was an open study and therefore the subject and investigator were aware of the group allocated and the treatment given." |
| Incomplete outcome data (attrition bias) | High risk of bias | 535 of 541 participants completed follow-up (99%). The individual participant data was not included. |
| Selective reporting (reporting bias) | High risk of bias | The outcome data contained redactions. |
| Other | High risk of bias | The trial was funded by GlaxoSmithKline and used inadequate design and reporting. |

HPV-030 (NCT00652938)

*Title*: “A phase IIIb, randomized, open, multicentre study to evaluate the immunogenicity and safety of GlaxoSmithKline Biologicals’ HPV-16/18 L1 VLP AS04 vaccine (Cervarix™) co-administrated with GlaxoSmithKline Biologicals’ Hepatitis B vaccine (Engerix-B™) in healthy female subjects aged 9 - 15 years.”

*Characteristics*:

| Methods | Randomized, parallel group, open trial that was 12 months long. |
| --- | --- |
| Participants | 493 healthy females (247 in the HPV vaccine group and 246 in the comparator group) age 9-15 allocated to seven different centres in the Netherlands and Sweden. Participants were excluded from the trial if they previously had received any of the adjuvants that were part of the HPV vaccine or comparator or had a history of any neurological or immunological disorder. |
| Interventions | Cervarix (0.5 millilitre) vs. Engerix (hepatitis B vaccine, 0.5 millilitre) given intramuscularly at 0, 1 and 6 months. |
| Outcomes | All-cause mortality, fatal harms, serious harms, new onset diseases (reported as ‘medically significant conditions’ for the whole study period), general harms (reported as ‘solicited’ and ‘unsolicited’ general harms 7- and 30-days post-vaccination, respectively). No benefit outcomes were eligible. |
| Notes | The control group was vaccinated with the HPV vaccine at the end of follow-up ("After completion of the safety follow-up and outside the study protocol, Engerix-B will be offered to the HPV group and Cervarix will be offered to the HepB [Engerix-B] group, if commercially available in the participating country”). |

*Risk of Bias assessment*:

| Risk of bias domain | Author’s judgment | Supporting statement |
| --- | --- | --- |
| Random sequence generation (selection bias) | Low risk of bias | "A randomization list was generated at GSK Biologicals, Rixensart, using a standard SAS (Statistical Analysis System) program and was used to number the vaccines. A randomization blocking scheme (1:1:1 ratio) was used to ensure that balance between treatments was maintained: a treatment number was to identify uniquely the vaccine doses to be administered to the same subject." |
| Allocation concealment (selection bias) | Low risk of bias | “The treatment allocation at the investigator site was to be performed using a central randomization system on Internet (SBIR) ... The person in charge of the vaccination was to access the randomization system on Internet.” |
| Blinding of participants and personnel (performance bias) | High risk of bias | "This was an open study and therefore the subject and investigator were aware of the group allocated and the treatment given." |
| Blinding of outcome assessment (detection bias) | High risk of bias | "This was an open study and therefore the subject and investigator were aware of the group allocated and the treatment given." |
| Incomplete outcome data (attrition bias) | High risk of bias | 482 of 494 participants completed follow-up (98%). The individual participant data was not included. |
| Selective reporting (reporting bias) | High risk of bias | The outcome data contained redactions. |
| Other | High risk of bias | The trial was funded by GlaxoSmithKline and used inadequate design and reporting. |

HPV-031 (NCT00344032)

*Title*: “A phase IIIb, double-blind, randomized, comparator-led study to evaluate the immunogenicity and safety of GlaxoSmithKline (GSK) Biologicals’ HPV-16/18 L1 VLP AS04 vaccine administered intramuscularly according to a 0, 1, 6 months schedule in healthy Indian female subjects aged 18 – 35 years.”

*Characteristics*:

| Methods | Randomized, parallel group, double-blind trial that was 7 months long. |
| --- | --- |
| Participants | 354 healthy females (176 in the HPV vaccine group and 178 in the comparator group) age 18-35 allocated to four different centres in India. Participants were excluded from the trial if they previously had received any of the adjuvants that were part of the HPV vaccine or comparator or had a history of any immunological disorder. |
| Interventions | Cervarix (0.5 millilitre) vs. aluminium hydroxide (Al[OH]_3_, 0.5 milligram in 0.5 millilitre saline) given intramuscularly at 0, 1 and 6 months. |
| Outcomes | All-cause mortality, fatal harms, serious harms, new onset diseases (reported as ‘medically significant conditions’ for the whole study period), general harms (reported as ‘solicited’ and ‘unsolicited’ general harms 7- and 30-days post-vaccination, respectively). No benefit outcomes were eligible. |
| Notes | None. |

*Risk of Bias assessment*:

| Risk of bias domain | Author’s judgment | Supporting statement |
| --- | --- | --- |
| Random sequence generation (selection bias) | Low risk of bias | "The randomization was performed at GSK Biologicals, Rixensart, using a standard SAS® (Statistical Analysis System) program. A randomization blocking scheme (1:1 ratio) was used to ensure that the balance between treatments was maintained: throughout the study, a single treatment number identified uniquely the vaccine/placebo doses to be administered to the same subject." |
| Allocation concealment (selection bias) | Low risk of bias | This was not explicitly described, but the trial was allegedly “double blind,” and we therefore assumed that the allocation concealment probably was adequate. |
| Blinding of participants and personnel (performance bias) | Low risk of bias | "This study was conducted in a double-blind manner. All subjects, the investigator and all study personnel involved in clinical evaluation of subjects were blinded to the individual subject treatment allocation (HPV-16/18 L1 VLP AS04 vaccine or placebo). GSK personnel directly involved in the conduct of the study were also blinded to the individual subject treatment. Blinding was maintained for the entire study period until the database was frozen." |
| Blinding of outcome assessment (detection bias) | Low risk of bias | "This study was conducted in a double-blind manner. All subjects, the investigator and all study personnel involved in clinical evaluation of subjects were blinded to the individual subject treatment allocation (HPV-16/18 L1 VLP AS04 vaccine or placebo). GSK personnel directly involved in the conduct of the study were also blinded to the individual subject treatment. Blinding was maintained for the entire study period until the database was frozen. " |
| Incomplete outcome data (attrition bias) | High risk of bias | 330 of 354 participants completed follow-up (93%). The individual participant data was not included. |
| Selective reporting (reporting bias) | High risk of bias | The outcome data contained redactions. |
| Other | High risk of bias | The trial was funded by GlaxoSmithKline and used inadequate design and reporting. |

HPV-032 (NCT00316693)

*Title*: “A double-blind (observer-blind), randomized, comparator-led, phase II study to assess the efficacy, immunogenicity and safety of GlaxoSmithKline Biologicals HPV-16/18 L1 VLP AS04 vaccine administered intramuscularly according to a 0, 1, 6-month schedule in healthy Japanese female subjects aged 20-25 years.”

*Characteristics*:

| Methods | Randomized, parallel group, observer-blind trial that was 7 months long. |
| --- | --- |
| Participants | 1,040 healthy females (519 in the HPV vaccine group and 521 in the comparator group) age 19-25 allocated to 13 different centres in Japan. Participants were excluded from the trial if they previously had received any of the adjuvants that were part of the HPV vaccine or comparator or had a history of any neurological or immunological disorder. |
| Interventions | Cervarix (0.5 millilitre) vs. Aimmugen (hepatitis A vaccine, 0.5 millilitre) given intramuscularly at 0, 1 and 6 months. |
| Outcomes | All-cause mortality and fatal harms. No benefit outcomes were eligible. Serious harms were not reported for individual MedDRA categories. New onset diseases and general harms were not reported. |
| Notes | None. |

*Risk of Bias assessment*:

| Risk of bias domain | Author’s judgment | Supporting statement |
| --- | --- | --- |
| Random sequence generation (selection bias) | Low risk of bias | "Randomization of supplies A randomization list was generated at GSK Biologicals, Rixensart, using a standard SAS (Statistical Analysis System) program and was used to number the vaccines. A randomization blocking scheme (1:1 ratio) was used to ensure that balance between treatments was maintained: a treatment number identified uniquely the vaccine doses to be administered to the same subject." |
| Allocation concealment (selection bias) | Low risk of bias | “The treatment allocation at the investigator/co-investigator site was performed using a central randomization system on Internet (SBIR) ... When a subject was eligible and informed consent had been obtained, the person in charge of randomization accessed SBIR. Upon providing the subject number, the randomization system used the minimization algorithm to determine the treatment number used for the subject.” |
| Blinding of participants and personnel (performance bias) | High risk of bias | "The study was double-blind (observer-blind) such that subjects and all study personnel involved in clinical evaluation of subjects were blinded to treatment allocation. Study personnel involved in vaccine administration were aware of treatment assignment but only participated in vaccine/comparator administration during the study. It was an observer blind study as the vaccines have different presentations. All GSK personnel, investigators/co-investigators and their study staff as well as study subjects remained blinded to the individual subject assignments and will remain such until the conclusion of the study (Month 24)." |
| Blinding of outcome assessment (detection bias) | High risk of bias | "The study was double-blind (observer-blind) such that subjects and all study personnel involved in clinical evaluation of subjects were blinded to treatment allocation. Study personnel involved in vaccine administration were aware of treatment assignment but only participated in vaccine/comparator administration during the study. It was an observer blind study as the vaccines have different presentations. All GSK personnel, investigators/co-investigators and their study staff as well as study subjects remained blinded to the individual subject assignments and will remain such until the conclusion of the study (Month 24)." |
| Incomplete outcome data (attrition bias) | High risk of bias | 941 of 1,040 participants completed follow-up (90%) The individual participant data was not included. |
| Selective reporting (reporting bias) | High risk of bias | The outcome data contained redactions. New onset diseases and general harms were not reported. |
| Other | High risk of bias | The trial was funded by GlaxoSmithKline and used inadequate design and reporting. |

HPV-033 (NCT00290277)

*Title*: “A phase III, double-blind, randomized, comparator-led study to evaluate the immunogenicity and safety of GSK Biologicals’ HPV-16/18 L1 VLP AS04 vaccine administered intramuscularly according to a 1, 1, 6 month schedule in healthy female subjects aged 10 – 14 years.”

*Characteristics*:

| Methods | Randomized, parallel group, observer-blind trial that was 7 months long. |
| --- | --- |
| Participants | 321 healthy females (160 in the HPV vaccine group and 161 in the comparator group) age 9-15 allocated to 8 different centres in South Korea. Participants were excluded from the trial if they previously had received any of the adjuvants that were part of the HPV vaccine or comparator or had a history of any neurological or immunological disorder. |
| Interventions | Cervarix (0.5 millilitre) vs. Havrix (hepatitis A vaccine, 0.5 millilitre) given intramuscularly at 0, 1 and 6 months. The batch numbers of the HPV vaccine and comparator were redacted. |
| Outcomes | All-cause mortality, fatal harms, serious harms, new onset diseases (reported as ‘medically significant conditions’ for the whole study period), general harms (reported as ‘solicited’ and ‘unsolicited’ general harms 7- and 30-days post-vaccination, respectively). No benefit outcomes were eligible. |
| Notes | None. |

*Risk of Bias assessment*:

| Risk of bias domain | Author’s judgment | Supporting statement |
| --- | --- | --- |
| Random sequence generation (selection bias) | Low risk of bias | Central randomization system. |
| Allocation concealment (selection bias) | Low risk of bias | “The treatment allocation was performed using a central randomization system on Internet (SBIR) ... When a subject was eligible and informed consent had been obtained, the person in charge of randomization accessed SBIR.” |
| Blinding of participants and personnel (performance bias) | High risk of bias | “All subjects and study personnel involved in study conduct including safety assessments and not involved in preparation and administration of study vaccines were blinded to the individual subject treatment…This was due to differences in the appearance of the HPV-16/18 vaccine and HAV comparator vaccine.” |
| Blinding of outcome assessment (detection bias) | Low risk of bias | “ ... study personnel involved in study conduct including safety assessments and not involved in preparation and administration of study vaccines were blinded to the individual subject treatment.” |
| Incomplete outcome data (attrition bias) | High risk of bias | 319 of 321 participants completed follow-up (99%). The individual participant data was not included. |
| Selective reporting (reporting bias) | High risk of bias | The outcome data contained redactions. |
| Other | High risk of bias | The trial was funded by GlaxoSmithKline and used inadequate design and reporting. |

HPV-035 (NCT00306241)

*Title*: “A Phase III, Double-blind, Randomized, Controlled Study to Evaluate Immunogenicity & Safety of GSK Biologicals' HPV-16/18 L1 VLP AS04 Vaccine, Administered Intramuscularly (0, 1, 6 Month Schedule) in Healthy Females Aged 18 - 35 Years.”

*Characteristics*:

| Methods | Randomized, parallel group, double-blind trial that was 7 months long. |
| --- | --- |
| Participants | 300 healthy females (150 in the HPV vaccine group and 150 in the comparator group) age 18-35 allocated in one centre in Hong Kong. Participants were excluded from the trial if they previously had received any of the adjuvants that were part of the HPV vaccine or comparator or had a history of any neurological or immunological disorder. |
| Interventions | Cervarix (0.5 millilitre) vs. aluminium hydroxide (Al[OH]_3_, 0.5 milligram in 0.5 millilitre saline) given intramuscularly at 0, 1 and 6 months. The batch numbers of the HPV vaccine and comparator were redacted. |
| Outcomes | All-cause mortality, fatal harms, serious harms, new onset diseases (reported as ‘medically significant conditions’ for the whole study period), general harms (reported as ‘solicited’ and ‘unsolicited’ general harms 7- and 30-days post-vaccination, respectively). No benefit outcomes were eligible. |
| Notes | The control group was vaccinated with the HPV vaccine at the end of follow-up. |

*Risk of Bias assessment*:

| Risk of bias domain | Author’s judgment | Supporting statement |
| --- | --- | --- |
| Random sequence generation (selection bias) | Low risk of bias | Central randomization list. |
| Allocation concealment (selection bias) | Low risk of bias | “allocation at the investigator site was performed using a central randomisation system on Internet (SBIR).” |
| Blinding of participants and personnel (performance bias) | Low risk of bias | This was not explicitly described, but the trial was allegedly “double blind,” and we therefore assumed that the blinding of participants and personnel probably was adequate. |
| Blinding of outcome assessment (detection bias) | Low risk of bias | This was not explicitly described, but the trial was allegedly “double blind,” and we therefore assumed that the blinding of outcome assessors probably was adequate. |
| Incomplete outcome data (attrition bias) | High risk of bias | 294 of 300 participants completed follow-up (98%) The individual participant data was not included. |
| Selective reporting (reporting bias) | High risk of bias | The outcome data contained redactions. |
| Other | High risk of bias | The trial was funded by GlaxoSmithKline and used inadequate design and reporting. |

HPV-038 (NCT00485732)

*Title*: “A phase IIIb, double-blind, randomized, comparatorled study to evaluate the immunogenicity and safety of GlaxoSmithKline (GSK) Biologicals HPV-16/18 L1 VLP AS04 vaccine, administered intramuscularly according to a 0, 1, 6 month schedule in healthy female subjects aged 15 – 25 years.”

*Characteristics*:

| Methods | Randomized, parallel group, double-blind trial that was 7 months long. |
| --- | --- |
| Participants | 225 healthy females (149 in the HPV vaccine group and 76 in the comparator group) age 15-25 allocated to six different centres in South Korea. Participants were excluded from the trial if they previously had received any of the adjuvants that were part of the HPV vaccine or comparator or had a history of any neurological or immunological disorder. |
| Interventions | Cervarix (0.5 millilitre) vs. aluminium hydroxide (Al[OH]_3_, 0.5 milligram in 0.5 millilitre saline) given intramuscularly at 0, 1 and 6 months. |
| Outcomes | All-cause mortality, fatal harms, serious harms, new onset diseases (reported as ‘medically significant conditions’ for the whole study period), general harms (reported as ‘solicited’ and ‘unsolicited’ general harms 7- and 30-days post-vaccination, respectively). No benefit outcomes were eligible. |
| Notes | None. |

*Risk of Bias assessment*:

| Risk of bias domain | Author’s judgment | Supporting statement |
| --- | --- | --- |
| Random sequence generation (selection bias) | Low risk of bias | "A randomization list was generated at GSK Biologicals, Rixensart, using a standard SAS (Statistical Analysis System) program and was used to number the vaccines. A randomization blocking scheme (2:1 ratio) was used to ensure that balance between treatments was maintained: throughout the study a single treatment number uniquely identified the vaccine doses to be administered to the same subject." |
| Allocation concealment (selection bias) | Low risk of bias | “The treatment allocation at the investigator site was performed using a central randomisation system on Internet (SBIR). The randomisation algorithm used a minimisation procedure. The person in charge of the vaccination accessed the randomisation system on Internet. Upon providing a subject number and the age for the subject, the randomisation system used the minimisation algorithm accounting for centre and age.” |
| Blinding of participants and personnel (performance bias) | Low risk of bias | "The study was performed in a double-blind manner. All subjects and study personnel were blinded to the individual subject treatment (HPV vaccine or placebo). GSK Biologicals’ personnel directly involved in the conduct of the study were also blinded to the individual subject treatment. Blinding was maintained for the whole study period (until the last subject enrolled completed the last visit at Month 7) and until the database was frozen." |
| Blinding of outcome assessment (detection bias) | Low risk of bias | "The study was performed in a double-blind manner. All subjects and study personnel were blinded to the individual subject treatment (HPV vaccine or placebo). GSK Biologicals’ personnel directly involved in the conduct of the study were also blinded to the individual subject treatment. Blinding was maintained for the whole study period (until the last subject enrolled completed the last visit at Month 7) and until the database was frozen." |
| Incomplete outcome data (attrition bias) | High risk of bias | 208 of 225 participants completed follow-up (92%). The individual participant data was not included. |
| Selective reporting (reporting bias) | High risk of bias | The outcome data contained redactions. |
| Other | High risk of bias | The trial was funded by GlaxoSmithKline and used inadequate design and reporting. |

HPV-040 (NCT00534638)

*Title*: “A phase III/IV, community-randomized, comparator-led study to evaluate the effectiveness of two vaccination strategies using GlaxoSmithKline Biologicals’ HPV-16/18 L1 VLP AS04 vaccine in reducing the prevalence of HPV-16/18 infection when administered intramuscularly according to a 0, 1, 6-month schedule in healthy female and male study participants aged 12 – 15 years.”

*Characteristics*:

| Methods | Cluster-randomized, community stratified, open trial that was 72 months long. |
| --- | --- |
| Participants | 32,176 healthy males and females (14,838 in the HPV vaccine group and 17,338 in the comparator group) age 12-16 allocated to 250 different clusters in 33 communities in Finland. |
| Interventions | Cervarix (0.5 millilitre) vs. Engerix-B (hepatitis B vaccine, 0.5 millilitre) given intramuscularly at 0, 1 and 6 months. |
| Outcomes | All-cause mortality, fatal and serious harms, general harms (reported as ‘solicited’ and ‘unsolicited’ general harms 7- and 30-days post-vaccination, respectively). No benefit outcomes were eligible. New onset diseases were reported as ‘medically significant conditions,’ but the results were redacted. |
| Notes | The control group was vaccinated with the HPV vaccine at the end of follow-up ("Cross-over vaccination will be offered to all female study participants immunized in the trial, when invited for the effectiveness evaluation phase (i.e., subjects that had received HPV vaccine will be offered vaccination with Hepatitis B vaccine and subjects that received Hepatitis B vaccine will be offered HPV vaccine). Similarly, males who received HPV vaccine during the Immunization phase will be invited to receive cross-over vaccination with Hepatitis B vaccine. After study completion, GSK’s HPV vaccine will be offered to male study participants who have received the Hepatitis B vaccine during the immunization phase if the results from the study demonstrate that male vaccination provides a meaningful benefit on the prevalence of HPV infection in females and if the vaccine is licensed for use in males”). |

*Risk of Bias assessment*:

| Risk of bias domain | Author’s judgment | Supporting statement |
| --- | --- | --- |
| Random sequence generation (selection bias) | Low risk of bias | “A randomization list was generated at GSK Biologicals, Rixensart, using a standard SAS® (Statistical Analysis System) program and was used to number the vaccines. A randomization blocking scheme (9:1 ratio) was used to ensure that balance between treatments was maintained: a treatment number identified uniquely the vaccine doses to be administered to the same study participant. The vaccine doses were distributed to each study centre, respecting the randomization block size.” |
| Allocation concealment (selection bias) | Low risk of bias | The treatment allocation at the investigator site was performed using SBIR. The randomization algorithm used a minimization procedure. The person in charge of the vaccination was to access the randomization system on Internet. |
| Blinding of participants and personnel (performance bias) | High risk of bias | "All study participants know in which intervention arm their community has been assigned." |
| Blinding of outcome assessment (detection bias) | High risk of bias | This was not explicitly described, but the trial was allegedly “open,” and we therefore assumed that the blinding of outcome assessors probably was inadequate. |
| Incomplete outcome data (attrition bias) | High risk of bias | 31,901 of 32,176 participants completed follow-up (99%). The individual participant data was not included. |
| Selective reporting (reporting bias) | High risk of bias | The outcome data contained redactions. Only a limited and varied number of participants were included in the harm analyses, e.g., serious harms for 3,703 participants or the serious harms that were judged vaccine-related by investigators were reported ("Any SAEs reported to the investigator and considered by the investigator as possibly related to vaccination were to be reported to GSK Biologicals”) and only general harms for 1,628 participants. |
| Other | High risk of bias | The trial was funded by GlaxoSmithKline and used inadequate design and reporting. |

HPV-058 (NCT00996125)

*Title*: “A phase III, double-blind, randomized, comparator-led study to evaluate the immunogenicity and safety of GSK Biologicals’ HPV-16/18 L1 VLP AS04 vaccine administered intramuscularly according to a 0, 1, 6-month schedule in healthy Chinese female subjects aged 9-17 years.”

*Characteristics*:

| Methods | Randomized, parallel group, double-blind trial that was 12 months long. |
| --- | --- |
| Participants | 750 healthy females (374 in the HPV vaccine group and 376 in the comparator group) age 9-17 allocated to one centre in China. Participants were excluded from the trial if they previously had received any of the adjuvants that were part of the HPV vaccine or comparator or had a history of any neurological or immunological disorder. |
| Interventions | Cervarix (0.5 millilitre) vs. aluminium hydroxide (Al[OH]_3_, 0.5 milligram in 0.5 millilitre saline) given intramuscularly at 0, 1 and 6 months. |
| Outcomes | All-cause mortality, fatal and serious harms, new onset diseases (reported as ‘medically significant conditions’ for the whole study period), general harms (reported as ‘solicited’ and ‘unsolicited’ general harms 7- and 30-days post-vaccination, respectively). No benefit outcomes were eligible. |
| Notes | None. |

*Risk of Bias assessment*:

| Risk of bias domain | Author’s judgment | Supporting statement |
| --- | --- | --- |
| Random sequence generation (selection bias) | Low risk of bias | Central MATEX randomization. |
| Allocation concealment (selection bias) | Low risk of bias | “The treatment allocation at the investigator site was performed using a central randomisation system on Internet (SBIR) … the study staff accessed the randomisation system on Internet.” |
| Blinding of participants and personnel (performance bias) | Low risk of bias | "Blinding was maintained for all subjects and investigators and their study staff" |
| Blinding of outcome assessment (detection bias) | Low risk of bias | "Blinding was maintained for all subjects and investigators and their study staff" |
| Incomplete outcome data (attrition bias) | High risk of bias | 734 of 750 participants completed follow-up (98%). The individual participant data was not included. |
| Selective reporting (reporting bias) | High risk of bias | The outcome data contained redactions. |
| Other | High risk of bias | The trial was funded by GlaxoSmithKline and used inadequate design and reporting. |

HPV-063 (NCT00929526)

*Title*: “An open, multi-centre, long term extension study to the primary vaccination phase II study HPV-032 to assess the efficacy of HPV-16/18 L1 VLP AS04 vaccine in the prevention of HPV-16 and/or HPV-18 associated cervical intraepithelial neoplasia (CIN) and cervical cancer, to assess the immunogenicity of HPV-16/18 L1 VLP AS04 vaccine and to assess safety up to 48 months after administration of the first dose of HPV-16/18 L1 VLP AS04 vaccine to healthy Japanese women vaccinated at 20 - 25 years of age.”

*Characteristics*:

| Methods | Randomized, parallel group, open trial that was 12 months long (follow-up to trial HPV-032). |
| --- | --- |
| Participants | 752 healthy females (375 in the HPV vaccine group and 377 in the comparator group) age 22-29 (at the time of this follow-up) allocated to 13 different centres in Japan. Participants had been excluded from the trial if they previously had received any of the adjuvants that were part of the HPV vaccine or comparator or had a history of any neurological or immunological disorder. |
| Interventions | Cervarix (0.5 millilitre) vs. Aimmugen (hepatitis A vaccine, 0.5 millilitre) given intramuscularly at 0, 1 and 6 months. |
| Outcomes | All-cause mortality, fatal harms, serious harms, new onset diseases (reported as ‘medically significant conditions’ for the whole study period). No benefit outcomes were eligible except for combined cervical surrogate outcomes (i.e., CIN2^+^ and CIN3^+^). General harms were reported in trial HPV-032. |
| Notes | None. |

*Risk of Bias assessment*:

| Risk of bias domain | Author’s judgment | Supporting statement |
| --- | --- | --- |
| Random sequence generation (selection bias) | Low risk of bias | "Randomization of supplies A randomization list was generated at GSK Biologicals, Rixensart, using a standard SAS (Statistical Analysis System) program and was used to number the vaccines. A randomization blocking scheme (1:1 ratio) was used to ensure that balance between treatments was maintained: a treatment number identified uniquely the vaccine doses to be administered to the same subject." |
| Allocation concealment (selection bias) | Low risk of bias | “The treatment allocation at the investigator/co-investigator site was performed using a central randomization system on Internet (SBIR) ... When a subject was eligible and informed consent had been obtained, the person in charge of randomization accessed SBIR. Upon providing the subject number, the randomization system used the minimization algorithm to determine the treatment number used for the subject.” |
| Blinding of participants and personnel (performance bias) | High risk of bias | "The study was open such that the subjects, the investigator/co-investigator and all study staff involved in clinical evaluation of subjects were aware of the previous treatment allocation in study HPV-032. However, to maintain the scientific integrity of the study, the laboratories in charge of the laboratory testing remained blinded to the treatment." |
| Blinding of outcome assessment (detection bias) | High risk of bias | "The study was open such that the subjects, the investigator/co-investigator and all study staff involved in clinical evaluation of subjects were aware of the previous treatment allocation in study HPV-032. However, to maintain the scientific integrity of the study, the laboratories in charge of the laboratory testing remained blinded to the treatment." |
| Incomplete outcome data (attrition bias) | High risk of bias | 706 of 752 participants completed follow-up (94%). The individual participant data was not included. |
| Selective reporting (reporting bias) | High risk of bias | The outcome data contained redactions. |
| Other | High risk of bias | The trial was funded by GlaxoSmithKline and used inadequate design and reporting. |

HPV-069 (NCT01277042)

*Title*: “A phase III, observer-blind, randomized, comparator-led study to evaluate the immunogenicity and safety of GlaxoSmithKline (GSK) Biologicals’ HPV-16/ 18 L1 VLP AS04 vaccine administered intramuscularly according to a 0, 1, 6-month schedule in healthy adult Chinese female subjects aged 26-45 years.”

*Characteristics*:

| Methods | Randomized, parallel group, observer-blind trial that was 12 months long. |
| --- | --- |
| Participants | 1,212 healthy females (606 in the HPV vaccine group and 606 in the comparator group) age 26-46 allocated to one centre in China. Participants were excluded from the trial if they previously had received any of the adjuvants that were part of the HPV vaccine or comparator or had a history of any immunological disorder. |
| Interventions | Gardasil (0.5 millilitre) vs. Engerix-B (hepatitis B vaccine, 0.5 millilitre) given intramuscularly at 0, 2 and 6 months. |
| Outcomes | All-cause mortality, fatal harms, serious harms, new onset diseases (reported as ‘medically significant conditions’ for the whole study period), general harms (reported as ‘solicited’ and ‘unsolicited’ general harms 7- and 30-days post-vaccination, respectively). No benefit outcomes were eligible. |
| Notes | None. |

*Risk of Bias assessment*:

| Risk of bias domain | Author’s judgment | Supporting statement |
| --- | --- | --- |
| Random sequence generation (selection bias) | Low risk of bias | "The randomization was performed at GSK Biologicals, Rixensart, using MATEX, a program developed for use in Statistical Analysis System (SAS®) (Cary, NC, USA) by GSK Biologicals. The vaccine doses were distributed to the study centre while respecting the randomization block size." |
| Allocation concealment (selection bias) | Low risk of bias | “The treatment allocation at the investigator site was performed using a central randomisation system on internet (SBIR) … the study staff in charge of the vaccination accessed SBIR.” |
| Blinding of participants and personnel (performance bias) | High risk of bias | "Because of the difference in volume and appearance between the HPV vaccine and Engerix-B, the vaccines were prepared and administrated by qualified medical personnel not otherwise involved in the conduct of the study or in the assessment of symptoms. Study staff involved in the assessment of subjects was blinded." |
| Blinding of outcome assessment (detection bias) | Low risk of bias | "Data was collected in an observer-blind manner. By observer-blind, it was meant that during the course of the study, the vaccine recipient and those responsible for the evaluation of any study endpoint (e.g. safety, reactogenicity and immunogenicity) were all unaware of which vaccine was administered. To do so, vaccine preparation and administration was done by authorised medical personnel who did not participate in any of the study clinical evaluation assays. Blinding was maintained for all subjects and investigators and their study staff participating in this study with regard to the individual subject treatment (HPV vaccine or comparator vaccine) assignments allocated in this study during the entire study period. GSK personnel directly involved in the conduct of this study (e.g. site monitors, medical monitors, laboratory personnel, etc.) were also blinded to the subject’s treatment assignments during the entire study period. The GSK statistician and authorised staff remained blinded until the complete freezing of the database." |
| Incomplete outcome data (attrition bias) | High risk of bias | 1,199 of 1,212 participants completed follow-up (99%). The individual participant data was not included. |
| Selective reporting (reporting bias) | High risk of bias | The outcome data contained redactions. |
| Other | High risk of bias | The trial was funded by GlaxoSmithKline and used inadequate design and reporting. |

V501-005 (NCT00365378)

*Title*: “Study of Pilot Manufacturing Lot of HPV 16 Virus-Like Particle (VLP) Vaccine in the Prevention of HPV 16 Infection in 16- to 23-Year-Old Females.”

*Characteristics*:

| Methods | Randomized, parallel group, double-blind trial that was 48 months long. |
| --- | --- |
| Participants | 2,409 healthy females (1,204 in the HPV vaccine group and 1,205 in the comparator group) age 16-25 allocated to 16 different centres in the United States. Participants were excluded from the trial if they previously had received any of the adjuvants that were part of the HPV vaccine or comparator or had a history of any immunological disorder. |
| Interventions | HPV 16 vaccine (0.5 millilitre) vs. amorphous aluminium hydroxyphosphate sulphate (AAHS, 0.225 milligram in 0.5 millilitre saline) given intramuscularly at 0, 2 and 6 months. The batch numbers of the HPV vaccine and comparator were redacted. |
| Outcomes | All-cause mortality, fatal and serious harms (reported 14 days post-vaccination), new onset diseases (reported as ‘new medical history’ for the whole study period), general harms (reported as ‘systemic clinical adverse evens’ 14 days post-vaccination). No benefit outcomes were eligible in the acquired clinical study report, but according to the table of contents eligible benefit outcomes were reported. |
| Notes | None. |

*Risk of Bias assessment*:

| Risk of bias domain | Author’s judgment | Supporting statement |
| --- | --- | --- |
| Random sequence generation (selection bias) | Low risk of bias | "A randomization schedule was generated by the Clinical Biostatistics department of MRL" |
| Allocation concealment (selection bias) | Low risk of bias | “… computer-generated allocation schedule … study subjects, study staff, and all Merck personnel were blinded to individual treatment allocation throughout the duration of the study.” |
| Blinding of participants and personnel (performance bias) | Low risk of bias | "The study was conducted under double-blind (with in house blinding) conditions. Thus, study subjects, study staff and all Merck Sharp & Dohme personnel were blinded to individual treatment allocation throughout the duration of the study." |
| Blinding of outcome assessment (detection bias) | Low risk of bias | "The study was conducted under double-blind (with in house blinding) conditions. Thus, study subjects, study staff and all Merck Sharp & Dohme personnel were blinded to individual treatment allocation throughout the duration of the study." |
| Incomplete outcome data (attrition bias) | High risk of bias | 1,671 of 2,409 participants completed follow-up (69%). The individual participant data was not included. |
| Selective reporting (reporting bias) | High risk of bias | The outcome data contained redactions. The trial only reported serious harms "…within 14 days following any vaccination visit.” |
| Other | High risk of bias | The trial was funded by Merck Sharp & Dohme and used inadequate design and reporting. |

V501-013 (NCT00092521)

*Title*: “A Study to Evaluate the Efficacy of Quadrivalent HPV Vaccine in Reducing the Incidence of HPV 6-, 11-, 16- and 18-Related CIN, AIS and Cervical Cancer and HPV 6-, 11-, 16- and 18-Related External Genital Warts, Vulvar Intraepithelial Neoplasia Vaginal Intraepithelial Neoplasia, Vulvar Cancer and Vaginal Cancer in 16- to 23-Year-Old Women.”

*Characteristics*:

| Methods | Randomized, parallel group, double-blind trial that was 45 months long. |
| --- | --- |
| Participants | 5,455 healthy females (2,732 in the HPV vaccine group and 2,723 in the comparator group) age 16-45 allocated to 62 different centres in Australia, Brazil, Canada, Colombia, Czech Republic, Germany, Hong Kong, Italy, Mexico, New Zealand, Peru, Puerto Rico, Russia, Thailand, the United Kingdom and the United States. Participants were excluded from the trial if they previously had received any of the adjuvants that were part of the HPV vaccine or comparator, had a history of any neurological disease or had a history of any immunological disorder. |
| Interventions | Gardasil (0.5 millilitre) vs. amorphous aluminium hydroxyphosphate sulphate (AAHS, 0.225 milligram in 0.5 millilitre saline) given intramuscularly at 0, 2 and 6 months. The batch numbers of the HPV vaccine and comparator were redacted. |
| Outcomes | All-cause mortality, mortality from and incidence of HPV-related cancers irrespective of HPV-type, incidence of histologically confirmed carcinoma in situ and moderate abnormal histology irrespective of HPV-type, external genital lesions, HPV-related referral procedures, fatal and serious harms (reported 14 days post-vaccination) and new onset diseases (reported as ‘new medical history’ for the whole study period). The trial did not report general harms. |
| Notes | The trial shortened the study period from 48 to 45 months. The control group was vaccinated with the HPV vaccine at the end of follow-up. |

*Risk of Bias assessment*:

| Risk of bias domain | Author’s judgment | Supporting statement |
| --- | --- | --- |
| Random sequence generation (selection bias) | Low risk of bias | Described as “randomized” but not how the randomization was performed (e.g., computer generated). |
| Allocation concealment (selection bias) | Low risk of bias | “Only the team responsible for the immunogenicity and safety analyses had access to the allocation schedule and unblinded database … individual treatment allocations were not revealed … Allocation schedules were generated by the Clinical Biostatistics department of MRL … The clinical, data management and statistics personnel at the Sponsor remained blinded to individual vaccination allocation through the completion of data review” |
| Blinding of participants and personnel (performance bias) | Low risk of bias | "Protocol 013 was double-blind, operating under in-house blinding procedures. In addition to the subject and the investigator, all laboratory personnel conducting the clinical assays, the clinical, statistical and data management data review team and the Pathology Panel members were blinded. This being an ongoing study, the investigator, including other study personnel, subjects, laboratory personnel and Pathology Panel members are to remain blinded until all subjects have completed the study at the Month 48 visit and the data are screened for completeness and accuracy." |
| Blinding of outcome assessment (detection bias) | Low risk of bias | "Protocol 013 was double-blind, operating under in-house blinding procedures. In addition to the subject and the investigator, all laboratory personnel conducting the clinical assays, the clinical, statistical and data management data review team and the Pathology Panel members were blinded.” |
| Incomplete outcome data (attrition bias) | High risk of bias | 4,313 of 5,455 participants completed follow-up (79%). The individual participant data was not included. |
| Selective reporting (reporting bias) | High risk of bias | The outcome data contained redactions. The trial only reported serious harms 14 days post-vaccination ("Investigators were instructed to report any serious adverse experience, including death due to any cause, occurring in any subject from the time the consent form was signed through 14 days following the first vaccination and from the time of any subsequent vaccinations through 14 days thereafter, whether or not related to the investigational product"). |
| Other | High risk of bias | The trial was funded by Merck Sharp & Dohme and used inadequate design and reporting. |

V501-015 (NCT00092534)

*Title*: “A Randomized Worldwide, Placebo-Controlled, Double-Blind Study to Investigate the Safety, Immunogenicity and Efficacy on the Incidence of HPV 16-/18-Related CIN 2/3 or Worse of the Quadrivalent HPV (Types 6, 11, 16, 18] Ll Virus-Like Particle (VLP) Vaccine in 16- to 23-Year-Old Women- The FUTURE II Study (Females United to Unilaterally Reduce Endo/Ectocervical Disease).”

*Characteristics*:

| Methods | Randomized, parallel group, double-blind trial that was 36 months long. |
| --- | --- |
| Participants | 12,167 healthy females (6,087 in the HPV vaccine group and 6,080 in the comparator group) age 15-26 allocated to 90 different centres in Brazil, Colombia, Denmark, Finland, Iceland, Mexico, Norway, Peru, Poland, Singapore, Sweden, the United Kingdom and the United States. Participants were excluded from the trial if they had a history of any immunological disorder. |
| Interventions | Gardasil (0.5 millilitre) vs. amorphous aluminium hydroxyphosphate sulphate (AAHS, 0.225 milligram in 0.5 millilitre saline) given intramuscularly at 0, 2 and 6 months. The batch numbers of the HPV vaccine and comparator were redacted. |
| Outcomes | All-cause mortality, mortality from and incidence of HPV-related cancers irrespective of HPV-type, incidence of histologically confirmed carcinoma in situ and moderate abnormal histology irrespective of HPV-type, external genital lesions, HPV-related referral procedures, fatal and serious harms (reported 14 days post-vaccination) and new onset diseases (reported as ‘new medical history’ for the whole study period). V501-015 did not report individual general harms. |
| Notes | The trial shortened the study period from 48 to 36 months. The control group was vaccinated with the HPV vaccine at the end of follow-up. |

*Risk of Bias assessment*:

| Risk of bias domain | Author’s judgment | Supporting statement |
| --- | --- | --- |
| Random sequence generation (selection bias) | Low risk of bias | Described as “randomized” but not how the randomization was performed (e.g., computer generated). |
| Allocation concealment (selection bias) | Low risk of bias | “Allocation schedules were generated by the Clinical Biostatistics department … An Interactive Voice Response System (IVRS) was used to allocate subjects. At the enrollment visit, the IVRS assigned the subject an AN [allocation number] from those allocated to the study site [16.1.7.1]. The IVRS subsequently assigned the appropriate vial identification number for HPV vaccine/placebo that corresponded to the subject's AN and vaccination group.” |
| Blinding of participants and personnel (performance bias) | Low risk of bias | "Protocol 015 was conducted under double-blind (with in-house blinding) procedures. In addition to the subject and the investigator, all laboratory personnel conducting the clinical assays and the Pathology Panel members were blinded. The investigator and his/her staff, subjects, laboratory personnel and Pathology Panel members were to remain blinded until all subjects completed the study, the data were screened for completeness and accuracy and all protocol violators were identified." |
| Blinding of outcome assessment (detection bias) | Low risk of bias | "Protocol 015 was conducted under double-blind (with in-house blinding) procedures. In addition to the subject and the investigator, all laboratory personnel conducting the clinical assays and the Pathology Panel members were blinded. The investigator and his/her staff, subjects, laboratory personnel and Pathology Panel members were to remain blinded until all subjects completed the study, the data were screened for completeness and accuracy and all protocol violators were identified." |
| Incomplete outcome data (attrition bias) | High risk of bias | 11,453 of 12,167 participants completed follow-up (94%). The individual participant data was not included. |
| Selective reporting (reporting bias) | High risk of bias | The outcome data contained redactions. The trial only reported serious harms 14 days post-vaccination ("All investigators were instructed to report any serious adverse experience, including death due to any cause, occurring in any subject from the time the consent form was signed through 14 days following the first vaccination and from the time of any subsequent vaccinations through 14 days thereafter, whether or not related to the investigational product”). |
| Other | High risk of bias | The trial was funded by Merck Sharp & Dohme and used inadequate design and reporting. |

V501-018 (NCT00092547)

*Title*: “A Safety and Immunogenicity Study of Quadrivalent HPV (Types 6, 11, 16, 18) L1 Virus-Like Particle (VLP) Vaccine in Preadolescents and Adolescents (Base Study). A Long Term Immunogenicity, Safety and Effectiveness Study of GARDASIL (Human Papillomavirus [Types 6, 11, 16, 18] Recombinant Vaccine) Among Adolescents Who Received GARDASIL at 9-18 Years of Age (Extension Study).”

*Characteristics*:

| Methods | Randomized, parallel group, observer-blind trial where we obtained data for the first 18 of 96 months. |
| --- | --- |
| Participants | 1,781 healthy males and females (1,184 in the HPV vaccine group and 597 in the comparator group) age 9-16 allocated to 47 different centres in Colombia, Denmark, Mexico, Norway, Portugal, Spain, Taiwan, Thailand, the United Kingdom and the United States. Participants were excluded from the trial if they had a history of any immunological disorder. |
| Interventions | Gardasil (0.5 millilitre) vs. carrier solution (yeast protein, sodium chloride, L-histidine, polysorbate 80 and sodium borate in 0.5 millilitre saline) given intramuscularly at 0, 2 and 6 months. The batch numbers of the HPV vaccine and comparator were redacted. |
| Outcomes | All-cause mortality, fatal and serious harms (reported 14 days post-vaccination), new onset diseases (reported as ‘new medical history’ for the whole study period), general harms (reported as ‘systemic clinical adverse evens’ 14 days post-vaccination). No benefit outcomes were eligible. |
| Notes | None. |

*Risk of Bias assessment*:

| Risk of bias domain | Author’s judgment | Supporting statement |
| --- | --- | --- |
| Random sequence generation (selection bias) | Low risk of bias | Described as “randomized” but not how the randomization was performed (e.g., computer generated). |
| Allocation concealment (selection bias) | Low risk of bias | “An Interactive Voice Response System (IVRS) was used to allocate study subjects … At the first visit, study personnel accessed the IVRS after a subject's parent/legal guardian had signed informed consent … The IVRS assigned the subject an allocation number (AN) and a unique vial identification number for the vial of clinical material that the subject should have received at that visit.” |
| Blinding of participants and personnel (performance bias) | Low risk of bias | "Because of the differences in the appearance of the quadrivalent HPV (Types 6, 11, 16, 18) LI VLP vaccine and placebo, administration of study material required both unblinded and blinded personnel to minimize bias. The subjects were seen first by the blinded personnel, who provided subjects with an informational brochure and obtained eligible subjects' consent/assent. The blinded and unblinded study personnel accessed IVRS, which assigned the subject with an AN and a unique vial identification number for the vial of clinical material that the subject should have received at that visit … The unblinded study personnel were considered unblinded during the course of the study because of their responsibilities in preparation and administration of the clinical material. As a result, they were NOT involved with subject management. Subjects were monitored by the blinded study personnel after vaccination was completed ... The unblinded study personnel were responsible for obtaining the subject's AN from the blinded study personnel, selecting the appropriate vial from the refrigerator, withdrawing and verifying the volume and contents of the syringe. The unblinded personnel recorded the subject's AN, date and their own initials onto the appropriate worksheet. Only the unblinded personnel had the responsibility for documentation that dealt with vaccine accountability. The unblinded study personnel wrapped the syringe with the non-transparent label provided by the Sponsor to mask the slight difference in appearance between quadrivalent HPV (Types 6, 11, 16, 18) L 1 VLP vaccine and placebo. After completing administration of the study material, the unblinded study personnel left the examination room immediately and had no further contact with the subject or parent/legal guardian during the remainder of the visit or during the 14-day follow-up period.” |
| Blinding of outcome assessment (detection bias) | Low risk of bias | “Review of medical history and the physical examination was conducted by the blinded personnel; the demographic information needed for vaccine/placebo preparation including the body weight, was provided to the unblinded personnel." |
| Incomplete outcome data (attrition bias) | High risk of bias | 1,680 of 1,781 participants completed follow-up until 18 months (94%). The individual participant data was not included. |
| Selective reporting (reporting bias) | High risk of bias | The outcome data contained redactions. The trial only reported serious harms 14 days post-vaccination ("Investigators were instructed to report any serious adverse experience, including death due to any cause, occurring in any subject from the time the consent form was signed through 14 days following the first vaccination and from the time of any subsequent vaccinations through 14 days thereafter"). |
| Other | High risk of bias | The trial was funded by Merck Sharp & Dohme and used inadequate design and reporting. |

V501-019 (NCT00090220)

*Title*: “Safety, Immunogenicity and Efficacy of Gardasil (V501 (Human Papilloma Virus [Types 6, 11, 16, 18] Recombinant Vaccine) in Mid-Adult Women - The FUTURE III (Females United to Unilaterally Reduce Endo/Ectocervical Cancer) Study.”

*Characteristics*:

| Methods | Randomized, parallel group, double-blind trial that was 48 months long. |
| --- | --- |
| Participants | 3,819 healthy females (1,911 in the HPV vaccine group and 1,908 in the comparator group) age 21-46 allocated to 38 different centres in Colombia, France, Germany, Philippines, Spain, Thailand and the United States. Participants were excluded from the trial if they previously had received any of the adjuvants that were part of the HPV vaccine or comparator or had a history of any neurological disorder. |
| Interventions | Gardasil (0.5 millilitre) vs. amorphous aluminium hydroxyphosphate sulphate (AAHS, 0.225 milligram in 0.5 millilitre saline) given intramuscularly at 0, 2 and 6 months. The batch numbers of the HPV vaccine and comparator were redacted. |
| Outcomes | All-cause mortality, mortality from and incidence of HPV-related cancers irrespective of HPV-type, incidence of histologically confirmed carcinoma in situ and moderate abnormal histology irrespective of HPV-type, external genital lesions, HPV-related referral procedures, fatal and serious harms (reported 14 days post-vaccination) and new onset diseases (reported as ‘new medical history’ for the whole study period). The trial did not report general harms. |
| Notes | None. |

*Risk of Bias assessment*:

| Risk of bias domain | Author’s judgment | Supporting statement |
| --- | --- | --- |
| Random sequence generation (selection bias) | Low risk of bias | Described as “randomized” but not how the randomization was performed (e.g., computer generated). |
| Allocation concealment (selection bias) | Low risk of bias | “The Clinical Biostatistics department of MRL generated the allocation schedule for randomizing study participants to the 2 treatment groups. Throughout the study and across all study sites, there was no repetition of an allocation number. Subjects were assigned an allocation number at randomization.” |
| Blinding of participants and personnel (performance bias) | Low risk of bias | "…study personnel, study subjects, laboratory personnel (including the Sponsor's laboratory staff) and Pathology Panel members remain blinded until all subjects complete the study (through Month 48), the data is screened for completeness and accuracy and all protocol violators are identified." |
| Blinding of outcome assessment (detection bias) | Low risk of bias | "This study was double-blind, operating under in-house blinding procedures. In addition to the subject and the investigator, the laboratory personnel conducting the clinical assays, the clinical, statistical and data management data review team and the Pathology Panel members were blinded." |
| Incomplete outcome data (attrition bias) | High risk of bias | 3,381 of 3,819 participants completed follow-up (89%). The individual participant data was not included. |
| Selective reporting (reporting bias) | High risk of bias | The outcome data contained redactions. The trial only reported serious harms 14 days post-vaccination ("Any SAE, including death due to any cause, which occurred to any subject in the study from the time the consent was signed through 14 days following the first vaccination and from the time of any subsequent vaccinations through 14 days thereafter"). |
| Other | High risk of bias | The trial was funded by Merck Sharp & Dohme and used inadequate design and reporting. |

V501-020 (NCT00090285)

*Title*: “A Study to Evaluate the Efficacy of GARDASIL in Reducing the Incidence of HPV 6-, 11-, 16- and 18-Related External Genital Warts, PIN, Penile, Perianal and Perineal Cancer and the Incidence of HPV 6-, 11-, 16- and 18-Related Genital Infection in Young Men.”

*Characteristics*:

| Methods | Randomized, parallel group, double-blind trial that was 36 months long. |
| --- | --- |
| Participants | 4,065 healthy males (2,032 in the HPV vaccine group and 2,033 in the comparator group) age 15-27 allocated to 71 different centres in Australia, Brazil, Canada, Costa Rica, Croatia, Finland, Germany, Mexico, the Netherlands, Norway, Peru, Philippines, Portugal, South Africa, Spain, Sweden, Taiwan and the United States. Participants were excluded from the trial if they had a history of any immunological disorder. |
| Interventions | Gardasil (0.5 millilitre) vs. amorphous aluminium hydroxyphosphate sulphate (AAHS, 0.225 milligram in 0.5 millilitre saline) given intramuscularly at 0, 2 and 6 months. The batch numbers of the HPV vaccine and comparator were redacted. |
| Outcomes | All-cause mortality, external genital lesions, HPV-related referral procedures, fatal and serious harms (reported 14 days post-vaccination), new onset diseases (reported as ‘new medical history’ for the whole study period) and general harms (reported as ‘systemic clinical adverse evens’ 14 days post-vaccination). The only eligible primary histological benefit outcome was the combined surrogate of PIN2^+^. |
| Notes | None. |

*Risk of Bias assessment*:

| Risk of bias domain | Author’s judgment | Supporting statement |
| --- | --- | --- |
| Random sequence generation (selection bias) | Low risk of bias | Described as “randomized” but not how the randomization was performed (e.g., computer generated). |
| Allocation concealment (selection bias) | Low risk of bias | "The Clinical Biostatistics department of Merck Sharp & Dohme Research Laboratories (MRL) generated the allocation schedule for randomizing study participants." |
| Blinding of participants and personnel (performance bias) | Low risk of bias | "This study was double-blind, operating under in-house blinding procedures. In addition to the subject and the investigator, the laboratory personnel conducting the clinical assays, the clinical, statistical and data management data review team and the Pathology Panel members were blinded. Every attempt was to be made to contact one of the individuals listed on the Sponsor Contact Information page of the protocol prior to unblinding the vaccination group for a specific case. If unblinding did occur (e.g., accidental unblinding, emergency unblinding due to a serious adverse experience), the investigator was instructed to promptly document the circumstances and immediately notify the MRL clinical monitor listed on the Sponsor Contact Information page of the protocol." |
| Blinding of outcome assessment (detection bias) | Low risk of bias | "This study was double-blind, operating under in-house blinding procedures. In addition to the subject and the investigator, the laboratory personnel conducting the clinical assays, the clinical, statistical and data management data review team and the Pathology Panel members were blinded." |
| Incomplete outcome data (attrition bias) | High risk of bias | 3,632 of 4,065 participants completed follow-up (89%). The individual participant data was not included. |
| Selective reporting (reporting bias) | High risk of bias | The outcome data contained redactions. The trial only reported serious harms 14 days post-vaccination ("Investigators were instructed to report any serious clinical adverse experience, including death due to any cause, occurring in any subject from the time the consent was signed through 14 days following the first vaccination and from the time of any subsequent vaccinations through 14 days thereafter, whether or not related to the investigational product”). |
| Other | High risk of bias | The trial was funded by Merck Sharp & Dohme and used inadequate design and reporting. |

V503-006 (NCT01047345)

*Title*: “A Phase III Randomized, International, Placebo-Controlled, Double-Blind Clinical Trial to Study the Tolerability and Immunogenicity of V 503, a Multivalent Human Papillomavirus (HPV) L1 Virus-Like Particle (VLP) Vaccine, Given to Females 12-26 Years of Age Who Have Previously Received GARDASIL™ (Protocol 006) 1.”

*Characteristics*:

| Methods | Randomized, parallel group, double-blind trial that was 7 months long. |
| --- | --- |
| Participants | 924 healthy females (618 in the HPV vaccine group and 306 in the comparator group) age 12-26 allocated to 32 different centres in Australia, Canada, Colombia, Denmark, Hong Kong, Mexico, Sweden and the United States. Participants were excluded from the trial if they previously had received any of the adjuvants that were part of the HPV vaccine or comparator or had a history of any immunological disorder. |
| Interventions | Gardasil 9 (0.5 millilitre) vs. saline placebo (0.5 millilitre) given intramuscularly at 0, 2 and 6 months. |
| Outcomes | All-cause mortality, fatal and serious harms (reported 14 days post-vaccination), new onset diseases (reported as ‘new medical history’ for the whole study period) and general harms (reported as ‘systemic clinical adverse evens’ 14 days post-vaccination). No benefit outcomes were eligible. |
| Notes | All participants were vaccinated with three doses of Gardasil more than 12 months before entering the study. |

*Risk of Bias assessment*:

| Risk of bias domain | Author’s judgment | Supporting statement |
| --- | --- | --- |
| Random sequence generation (selection bias) | Low risk of bias | “The study used central randomization.” |
| Allocation concealment (selection bias) | Low risk of bias | “An Interactive Voice Response System (IVRS) was used to allocate study subjects and assist with the vaccine supply management at the study site. At the first visit, study personnel accessed the IVRS after the subject had signed informed consent (or for minors, after a subject's parent/legal guardian had signed informed consent and the subject had signed assent) and after the subject had met all inclusion and none of the exclusion criteria. The IVRS assigned the subject allocation number (AN) and a unique vial identification number for the vial of clinical material that the subject was to receive at that visit.” |
| Blinding of participants and personnel (performance bias) | Low risk of bias | "The subjects, investigators (and his/her staff), laboratory staff and Sponsor remained blinded to subject vaccine allocation for the duration of the study. Because the 9vHPV vaccine and normal saline placebo can be visibly distinguished, the vaccine/placebo in this study had to be prepared by an unblinded third party who was otherwise not involved in the conduct of the study. The unblinded third party was responsible for all procedures involving clinical supplies." |
| Blinding of outcome assessment (detection bias) | Low risk of bias | "The subjects, investigators (and his/her staff), laboratory staff and Sponsor remained blinded to subject vaccine allocation for the duration of the study. Because the 9vHPV vaccine and normal saline placebo can be visibly distinguished, the vaccine/placebo in this study had to be prepared by an unblinded third party who was otherwise not involved in the conduct of the study. The unblinded third party was responsible for all procedures involving clinical supplies." |
| Incomplete outcome data (attrition bias) | High risk of bias | 895 of 924 participants completed follow-up (97%). The individual participant data was not included. |
| Selective reporting (reporting bias) | High risk of bias | The outcome data contained redactions. The trial only reported serious harms 14 days post-vaccination ("…serious adverse events within 14 days following any vaccination visit, vaccine-related serious adverse events observed during any time in the study…”). |
| Other | High risk of bias | The trial was funded by Merck Sharp & Dohme and used inadequate design and reporting. |
