# Supplementary material for: Benefits and harms of the human papillomavirus (HPV) vaccines: systematic review with meta-analyses of trial data from clinical study reports
Source: Syst Rev. 2020 Feb 28;9:43. doi: 10.1186/s13643-019-0983-y (PMC7047375; doi:10.1186/s13643-019-0983-y)
Supplement: Supplementary file 3 — Benefits and harms of the HPV vaccines—list of excluded studies. (DOCX 63 kb) [file 13643_2019_983_MOESM3_ESM.docx]

**Additional file 3**

**Benefits and harms of the HPV vaccines: list of excluded studies**

List of excluded industry funded studies

| **No.** | **Reason for exclusion** | **Manufacturer ID** | **Study ID** | **NCT ID** | **Title** |
| --- | --- | --- | --- | --- | --- |
| 1 | *Ineligible comparator* | HPV-002 | 580299/002 | Not identified | Phase I, Open-label Study to Evaluate the Safety, Tolerability and Immunogenicity of a Fourth Dose of Human Papillomavirus (HPV) DNA Plasmid (VGX-3100) + Electroporation (EP) in Adult Females Previously Immunized With VGX-3100 |
| 2 | *Ineligible comparator* | HPV-004 | 580299/004 | NCT00693615 | Study to Evaluate the Safety and Immunogenicity of MEDI-517, a Virus-Like Particle Vaccine Against Human Papillomavirus Types 16 and 18, When Formulated With Aluminum Hydroxide, AS04, or Without Adjuvant, in Healthy Adult Female Volunteers |
| 3 | *Ineligible comparator* | HPV-010 | 108933 | NCT00423046 | Phase IIIb, Observer-blind Study to Compare Immunogenicity of GSK Biologicals' HPV-16/18 L1/AS04 Vaccine Versus Gardasil® [Quadrivalent Human Papillomavirus (HPV-6,11,16,18 L1 VLP) Recombinant Vaccine Merck & Co., Inc.] |
| 4 | *Ineligible comparator* | HPV-012 | 107479 | NCT00337818 | A Long-term, Open, Follow-up of the Immunogenicity and Safety of GlaxoSmithKline Biologicals' HPV-16/18 L1/AS04 Vaccine in Healthy Female Subjects Vaccinated Either Pre- or Post-menarche in the Primary Study |
| 5 | *Ineligible comparator* | HPV-016 | 104772 | NCT00250276 | Assess Lot-to-lot Consistency of GSK Biologicals' HPV-16/18 L1/AS04 Vaccine Following Manufacturing Adjustments Administered Intramuscularly According to a 0,1,6-mth Schedule in Healthy Female Subjects (18-25 y) |
| 6 | *Ineligible comparator* | HPV-018 | 107682 | NCT00369824 | A Randomized, Open Study to Evaluate the Safety and Immunogenicity of GlaxoSmithKline Biologicals' HPV Vaccine Co-administered Intramuscularly With Boostrix® and/or Menactra™ in Healthy Female Subjects Aged 11-18 Years |
| 7 | *Ineligible comparator* | HPV-019 | 109823 | NCT01031069 | Safety and Immunogenicity of Cervarix™ in Human Immunodeficiency Virus Infected Females |
| 8 | *Ineligible comparator* | HPV-024 | 109628 | NCT00546078 | Safety and Immunogenicity Study of an Additional Dose of HPV Vaccine (580299) in Young, Adult Women in North America. |
| 9 | *Ineligible comparator* | HPV-026 | 111567 | NCT00637195 | Evaluation of the Immunogenicity and Safety of a Commercially Available Vaccine When Co-administered With GlaxoSmithKline Biologicals' HPV Vaccine (580299) in Healthy Female Subjects. |
| 10 | *Ineligible comparator* | HPV-042 | 108464 | NCT00426361 | A Multicentre Study to Evaluate the Immunogenicity and Safety of GSK Biologicals' HPV Vaccine (580299) Co-administered With Boostrix Polio (dTpa-IPV) in Healthy Female Subjects Aged 10-18 Years |
| 11 | *Ineligible comparator* | HPV-044 | 109179 | NCT00552279 | Immunogenicity and Safety Study of GlaxoSmithKline Biologicals' HPV Vaccine GSK580299 Administered According to an Alternative Dosing Schedule as Compared to the Standard Dosing Schedule in Young Female Subjects Aged 15-25 Years |
| 12 | *Ineligible comparator* | HPV-049 | Not identified | Not identified | No title. |
| 13 | *Ineligible comparator* | HPV-051 | 102115 | NCT00231413 | A Dose-range Study to Assess the Safety and Immunogenicity of a Novel HPV Vaccine When Administered Intramuscularly According to a 3-dose Schedule (0,1,6-month) in Healthy Adult Females (18-25 Years of Age) |
| 14 | *Ineligible comparator* | HPV-051  follow-up | 108052 | NCT00359619 | A Long-term, Follow-up of the Immunogenicity and Safety of GlaxoSmithKline Biologicals' Novel HPV Vaccine in Healthy Female Subjects Vaccinated in the Primary Study |
| 15 | *Ineligible comparator* | HPV-055 | 111758 | NCT00849381 | No title. |
| 16 | *Ineligible comparator* | HPV-060 | 112772 | NCT00947115 | Follow-up Study to Evaluate the Long-term Immunogenicity and Safety of GlaxoSmithKline Biologicals' HPV (580299) Vaccine in Healthy Female Subjects |
| 17 | *Ineligible comparator* | HPV-070 | 114700 | NCT01381575 | Immunogenicity and Safety Study of GlaxoSmithKline Biologicals' HPV-16/18 L1 AS04 Vaccine When Administered According to Alternative 2-dose Schedules in 9 - 14-Year-Old Females |
| 18 | *Ineligible comparator* | HPV-071 | 115411 | NCT01462357 | Immunogenicity and Safety Study of GlaxoSmithKline Biologicals' HPV-16/18 L1 AS04 Vaccine and Merck's Gardasil® Vaccine When Administered According to Alternative 2-dose Schedules in 9-14-Year-Old Females |
| 19 | *Ineligible comparator* | HPV-071-PRI | 2011-002035-26 | Not identified | A Phase IIIb observer-blind, randomized, multicentre primary immunization study to evaluate the immunogenicity and safety of GSK Biologicals’ HPV-16/18 L1 VLP AS04 vaccine and Merck's Quadrivalent Human Papillomavirus (Types 6, 11, 16, 18) Recombinant Vaccine, when administered intramuscularly according to alternative 2-dose schedules in 9-14 year old healthy females |
| 20 | *Ineligible comparator* | V501-016 | 2004_083 | NCT00092495 | A Study to Demonstrate Immunogenicity and Tolerability of Gardasil (V501) Quadrivalent HPV (Types 6, 11, 16, 18) L1 Virus-Like Particle (VLP) Vaccine in Preadolescents, and To Determine End-Expiry Specifications for the Vaccine |
| 21 | *Ineligible comparator* | V501-024 | 2005_093 | NCT00337428 | An Open-Label, Randomized, Multicenter Study of the Safety, Tolerability, and Immunogenicity of Gardasil (V501) Given Concomitantly With REPEVAX™ in Healthy Adolescents 11-17 Years of Age |
| 22 | *Ineligible comparator* | V501-025 | 2005_092 | NCT00325130 | An Open-Label, Randomized, Multicentre Study of the Safety, Tolerability, and Immunogenicity of V501 Given Concomitantly With Menactra™ and ADACEL™ in Healthy Adolescents 11-17 Years of Age |
| 23 | *Ineligible comparator* | V502-001 | 2005_086 | NCT00260039 | A Randomized, International, Double-Blinded (With In-House Blinding), GARDASIL-Controlled, Dose-Ranging Study of Octavalent Human Papillomavirus (HPV) L1 Virus-like Particle (VLP) Vaccine Administered to 16- to 23- Year-Old Women |
| 24 | *Ineligible comparator* | V503-001 | 2007_538 | NCT00543543 | A Randomized, International, Double-Blinded (With In-House Blinding), Controlled With GARDASIL, Dose-Ranging, Tolerability, Immunogenicity, and Efficacy Study of a Multivalent Human Papillomavirus (HPV) L1 Virus-Like Particle (VLP) Vaccine Administered to 16- to 26- Year-Old Women |
| 25 | *Ineligible comparator* | V503-001-04 | PER-090-13 | Not identified | A RANDOMIZED, INTERNATIONAL, DOUBLE-BLINDED (WITH IN-HOUSE BLINDING), CONTROLLED WITH GARDASIL™, DOSE-RANGING, TOLERABILITY, IMMUNOGENICITY, AND EFFICACY STUDY OF A MULTIVALENT HUMAN PAPILLOMAVIRUS (HPV) L1 VIRUS-LIKE PARTICLE (VLP) VACCINE ADMINISTERED TO 16- TO 26-YEAR-OLD WOMEN |
| 26 | *Ineligible comparator* | V503-002 (immune-bridging study) | Not identified | NCT00943722 | Immunogenicity and Safety of a 9-Valent HPV Vaccine |
| 27 | *Ineligible comparator* | V503-002  (lot consistency study) | 2009_611 | NCT00943722 | A Phase III Clinical Trial to Study the Immunogenicity, Tolerability, and Manufacturing Consistency of V503 (A Multivalent Human Papillomavirus [HPV] L1 Virus-Like Particle [VLP] Vaccine) in Preadolescents and Adolescents (9 to 15 Year Olds) With a Comparison to Young Women (16 to 26 Year Olds) |
| 28 | *Ineligible comparator* | V503-002 follow-up | PER-120-12 | Not identified | A PHASE III CLINICAL TRIAL TO STUDY THE IMMUNOGENICITY, TOLERABILITY, AND MANUFACTURING CONSISTENCY OF V503 (A MULTIVALENT HUMAN PAPILLOMAVIRUS [HPV] L1 VIRUS-LIKE PRTICLE [VLP] VACCINE) IN PREADOLESCENTS AND ADOLESCENTS (9 TO 15 YEAR OLDS) WITH A COMPARISON TO YOUNG WOMEN (16 TO 26 YEAR OLDS) |
| 29 | *Ineligible comparator* | V503-003 | 2012-002758-22 | NCT01651949 | A Phase III Clinical Trial to Study the Tolerability and Immunogenicity of 9vHPV (V503), a Multivalent Human Papillomavirus (HPV) L1 Virus-Like Particle (VLP) Vaccine, in 16- to 26-Year-Old Men and 16- to 26-Year-Old Women |
| 30 | *Ineligible comparator* | V503-005 | Not identified | NCT00988884 | A Phase III Open-Label Clinical Trial to Study the Immunogenicity and Tolerability of V503 (A Multivalent Human Papillomavirus [HPV] L1 Virus-Like Particle [VLP] Vaccine) Given Concomitantly With Menactra™ and Adacel™ in Preadolescents and Adolescents (11 to 15 Year Olds) |
| 31 | *Ineligible comparator* | V503-007 | 2010_512 | NCT01073293 | A Phase III Open-Label Clinical Trial to Study the Immunogenicity and Tolerability of V503, a Multivalent Human Papillomavirus (HPV) L1 Virus-Like Particle (VLP) Vaccine, Given Concomitantly With REPEVAX™ in Preadolescents and Adolescents (11 to 15 Year Olds) |
| 32 | *Ineligible comparator* | V503-009 | GDS01C | NCT01304498 | A Randomized, Double-Blind, Phase III Study of the Immunogenicity and Safety of a 9-Valent Human Papillomavirus L1 Virus-Like Particle Vaccine (V503) Versus Gardasil® in 9-15-Year-Old Girls. Vesikari T, Brodszki N, van Damme P, et al. Pediatr Infect Dis J. 2015; 34(9):992-998. |
| 33 | *Ineligible comparator* | V503-010 | 2013-001314-15 | NCT01984697 | A Phase III Clinical Trial to Study the Tolerability and Immunogenicity of a 2-dose Regimen of V503, a Multivalent Human Papillomavirus (HPV) L1 Virus-Like Particle (VLP) Vaccine, Administered in Preadolescents and Adolescents (9 to 14 Year Olds) With a Comparison to Young Women (16 to 26 Year Olds) |
| 34 | *Ineligible comparator* | V503-020 | GDS07C | NCT02114385 | A Randomized, Double-Blinded, Controlled With GARDASIL (Human Papillomavirus Vaccine [HPV] [Types 6, 11, 16, 18] (Recombinant, Adsorbed)), Phase 3 Clinical Trial to Study the Immunogenicity and Tolerability of V503 (9-Valent Human Papillomavirus L1 Virus-Like Particle [VLP] Vaccine) in 16- to 26-year-old Men |
| 35 | *Ineligible comparator* | V503-021 | Not identified | NCT02653118 | A Registry-Based Extension of Protocol V503-001 in Countries With Centralized Cervical Cancer Screening Infrastructures to Evaluate the Long-Term Effectiveness, Immunogenicity, and Safety of Multivalent Human Papillomavirus (HPV) L1 Virus- Like Particle (VLP) Vaccine as Administered to 16- to 26- Year- Old Women |
| 36 | *Ineligible comparator* | V504-001 | 2007_566 | NCT00551187 | A Randomized, Double-Blinded, Tolerability and Immunogenicity Study of a Multivalent Human Papillomavirus (HPV) L1 Virus-Like Particle (VLP) Vaccine Administered Concomitantly With GARDASIL to 16- to 26- Year-Old Women |
| 37 | *Ineligible comparator* | Not identified | 2016-001963-35 | NCT02199691 | Immunogenicity and Safety of an Investigational Quadrivalent Meningococcal Conjugate Vaccine in Healthy Adolescents |
| 38 | *Ineligible comparator* | Not identified | Not identified | NCT02993757 | Immunogenicity and Safety of a Tetravalent Dengue Vaccine Administered Concomitantly or Sequentially With Gardasil® in Healthy Subjects Aged 9 to 13 Years in Malaysia |
| 39 | *Ineligible comparator* | Not identified | Not identified | NCT02979535 | Immunogenicity and Safety of a Tetravalent Dengue Vaccine Administered Concomitantly or Sequentially With Cervarix® in Healthy Female Subjects Aged 9 to 14 Years in Mexico |
| 40 | *Ineligible comparator* | Not identified | Merck IISP 35706 IRB00001625 | NCT00925288 | Acceptability and Feasibility of a Modified HPV Vaccine Schedule in Brothel Based Female Sex Workers in Peru |
| 41 | *Ineligible comparator* | Not identified | MERCK 08 | NCT02968420 | Long Term Immune Memory Responses to Human Papillomavirus (HPV) Vaccination Following 2 Verses 3 Doses of Quadrivalent HPV Vaccine (Merck08) |
| 42 | *Ineligible comparator* | Not identified | 32090 | NCT00572832 | Randomized Trial of Alternative Quadrivalent Human Papilloma Virus (HPV) Vaccination Schedules in a University Setting |
| 43 | *Ineligible participants* | HPV-005 | 580299/005 | NCT00693966 | A Phase II Double-Blind, Randomized, Dose-Comparison Study to Evaluate the Safety and Immunogenicity of MEDI-517, a Virus-Like Particle Vaccine Against Human Papillomavirus Types 16 and 18, in Healthy Adult Female Volunteers |
| 44 | *Ineligible participants* | HPV-020 | 107863 | NCT00586339 | Evaluation of the Safety and Immunogenicity of GlaxoSmithKline Biologicals' HPV Vaccine 580299 (Cervarix TM) in Adult Human Immunodeficiency Virus (HIV) Infected Female Subjects |
| 45 | *Ineligible participants* | V501-011 | 2007_576 | NCT00517309 | Immunogenicity and Safety of Quadrivalent HPV L1 Virus-Like Particle (VLP) Vaccine in 16- to 23-Year-Old Women When Administered Alone or Concomitantly With Hepatitis B Vaccine (Recombinant)--the F.U.T.U.R.E. Study (Females United to Unilaterally Reduce Endo/Ectocervical Disease |
| 46 | *Ineligible participants* | V501-012 | 2004_080 | NCT00092482 | Immunogenicity and Safety of Gardasil (V501) Quadrivalent HPV (Types 6, 11, 16, 18) L1 Virus-Like Particle (VLP) Vaccine in Consistency Lots for 16- to 23-Year-Old Women With and Additional Immunogenicity Bridge to the Monovalent HPV 16 Vaccine Pilot Manufacturing Lot Study-The F.U.T.U.R.E. Study (Females United to Unilaterally Reduce Endo/Ectocervical Disease) [participants were included in V501-013] |
| 47 | *Ineligible participants* | Not identified | QHPV-RTC | NCT01928225 | A Randomized, Placebo-controlled Trial of Pre-treatment HPV Vaccination on Outcomes to LEEP Treatment of Cervical High Grade Squamous Intraepithelial Lesions in HIV-infected Women. |
| 48 | *Not comparative* | HPV-023  follow-up | 114379 | NCT01418937 | Safety Study of GSK Biologicals' Human Papillomavirus Vaccine (GSK-580299) in Healthy Female Control Subjects From the GSK HPV-023 Study |
| 49 | *Not comparative* | HPV-025 | 111375 | NCT00877877 | Follow-up Study to Evaluate the Long-term Immunogenicity and Safety of a HPV Vaccine (GSK 580299) in Healthy Female Subjects |
| 50 | *Not comparative* | HPV-052 | 112024 | NCT00937950 | Gynaecological follow-up of a subset of 580299/008 study subjects |
| 51 | *Not comparative* | HPV-056 | 111712 | NCT00811798 | Safety Study of GSK Biologicals' Human Papillomavirus Vaccine (GSK-580299) in Healthy Female Subjects. |
| 52 | *Not comparative* | HPV-057 | 111955 | NCT00799825 | Safety Study of GSK Biologicals' Human Papillomavirus Vaccine (GSK580299) in Female American and Canadian Subjects Who Had Received Control Vaccine in Study 580299/008 |
| 53 | *Not comparative* | HPV-062 | 113617 | NCT01190176 | Gynaecological Follow-up of a Subset of HPV-015 Study Subjects |
| 54 | *Not comparative* | HPV-066 | 113618 | NCT01249365 | Safety Study of GSK Biologicals' Human Papillomavirus Vaccine (GSK-580299) in Healthy Female Control Subjects From the Primary NCT00294047 Study |
| 55 | *Not comparative* | HPV-067 | 113621 | NCT01190189 | Safety Study of GSK Biologicals' Human Papillomavirus Vaccine (GSK-580299) in Healthy Female Control Subjects From the Primary NCT00294047 Study |
| 56 | *Not comparative* | V501-030-1 follow up | Not identified | NCT01427777 | The Thirty-six-Month Immunogenicity Evaluation of Quadrivalent HPV (Types 6, 11, 16, 18) L1 Virus-Like Particle (VLP) Vaccine in Chinese Female Subjects Aged 9 to 45 Years and Male Subjects Aged 9 to 15 Years |
| 57 | *Not completed/ongoing as of 1 July 2017* | 311-HPV-1001 | Not identified | NCT03085381 | A Randomized, Double-Blind and Placebo-Controlled Phase I Study to Evaluate the Safety and Primary Immunogenicity of the Quadrivalent Human Papillomavirus (Types 6, 11, 16, 18) Recombinant Vaccine (Hansenula Polymorpha) in Chinese Female Subjects Aged 9-45 Years |
| 58 | *Not completed/ongoing as of 1 July 2017* | 311-HPV-1002 | Not identified | NCT02740790 | Immunogenicity and Safety of Recombinant Human Papillomavirus Bivalent(Type 16 and 18) Vaccine (Yeast) in Healthy Females |
| 59 | *Not completed/ongoing as of 1 July 2017* | 311-HPV-1003 | Not identified | NCT02733068 | A Phase III Double Blinded, Randomized Controlled Study to Evaluate Efficacy of Protection Against HPV-16 and 18 Related Diseases, Immunogenicity and Safety of HPV-16/18 Vaccine in Healthy Females Aged 18-30 Years |
| 60 | *Not completed/ongoing as of 1 July 2017* | 311-HPV-1004 | Not identified | NCT02740777 | Immunogenicity Study of a 2-dose Immunization Schedule of Recombinant Human Papillomavirus Virus-like Particle Vaccine (Type 16 and 18 L1 Proteins, Yeast) in Adolescent Females Aged 9 to 14 Years |
| 61 | *Not completed/ongoing as of 1 July 2017* | HPV-023-EPI | 109624 | NCT00518336 | Brazilian cohort of women vaccinated in the phase IIb, blinded, primary study 580299/001 |
| 62 | *Not completed/ongoing as of 1 July 2017* | HPV-027 | 115006 | NCT01393470 | A Long-term Follow-up Registry-based Cohort Study of HPV Vaccine Efficacy Against Cervical Pre-cancerous Lesions and Cervical Cancers in a Cohort of Females Previously Enrolled From Finland in Study HPV-008, as Compared to a Non-intervention Population -Based Reference Cohort of Females From Finland |
| 63 | *Not completed/ongoing as of 1 July 2017* | HPV-111103-EPI | 111103 | Not identified | A phase IV, randomized, open-label, controlled, post-licensure study to evaluate the safety of GlaxoSmithKline Biologicals’ HPV-16/18 L1 VLP AS04 vaccine (Cervarix®) when administered intramuscularly according to a 0, 1, 6-month schedule in females aged 18-25 years. - EPI-HPV-111103 |
| 64 | *Not completed/ongoing as of 1 July 2017* | HPV-PRO-003 | Not identified | NCT01735006 | A Phase III Multicentre, Randomized, Double-Blind, Placebo(Hepatitis E Vaccine) Controlled Study to Evaluate the Efficacy, Immunogenicity and Safety of a Recombinant (E.coli) Human Papillomavirus Bivalent Vaccine in Healthy Women |
| 65 | *Not completed/ongoing as of 1 July 2017* | HPV-PRO-005 | Not identified | NCT02710851 | A Phase Ⅱ Randomized, Double-Blinded, Placebo Controlled Study to Evaluate the Immunogenicity of the Recombinant (E.coli) Human Papillomavirus Type 6/11 Bivalent Vaccine in Healthy Volunteers Aged 18-55 Years |
| 66 | *Not completed/ongoing as of 1 July 2017* | V501-122 | 132237 | NCT01862874 | A Phase III Placebo-controlled Clinical Trial to Study the Tolerability, Immunogenicity and Efficacy of V501 in 16- to 26-year-old Japanese Men |
| 67 | *Not randomised* | HPV-014 | 103514 | NCT00196937 | Phase 3, Open, Age-stratified Study to Assess Immunogenicity and Safety of GSK Biologicals' HPV-16/18 Vaccine Administered Intramuscularly According to 3-dose Schedule (0,1,6 Months) in Healthy Female Subjects Aged 15 - 55 Years and Long-Term Follow-up |
| 68 | *Not randomised* | HPV-018-EPI | 114101 | NCT01905462 | Post-marketing Safety Study to Assess the Risk of Spontaneous Abortions in Women Exposed to Cervarix in the United Kingdom |
| 69 | *Not randomised* | HPV-040-EPI | 116239 | NCT01953821 | An Observational Cohort Study to Assess the Risk of Autoimmune Diseases in Adolescent and Young Adult Women Aged 9 to 25 Years Exposed to Cervarix® in the United Kingdom |
| 70 | *Not randomised* | HPV-PRO-006 | Not identified | NCT02562508 | Immunogenicity and Safety Study of a Bivalent Human Papillomavirus (Type 16, 18) Recombinant Vaccine (E.coli) in Healthy Female Subjects Aged 9 to 17 Years |
| 71 | *Not randomised* | HPV-PRO-006-2 | Not identified | NCT03206255 | Immuno-persistence Study of a Bivalent Human Papillomavirus (Type 16, 18) Recombinant Vaccine (E.coli) in Healthy Female Subjects Aged 9 to 17 Years |
| 72 | *Not randomised* | V501-031 | 2010_019 | NCT01078220 | A Post-licensure Surveillance Program for the Safety of GARDASIL™ in a Managed Care Organization Setting |
| 73 | *Not randomised* | V501-033 | 2010_018 | NCT01077856 | GARDASIL™ Vaccine Impact in Population Study |
| 74 | *Not randomised* | V501-070 | EP08014.070 | NCT01567813 | Post-Licensure Observational Study of the Safety of GARDASIL™ in Males |
| 75 | *Not randomised* | V503-004 | Not identified | NCT03158220 | An Open-Label Phase III Clinical Study to Study the Immunogenicity and Tolerability of GARDASIL®9 (A Multivalent Human Papillomavirus [HPV] L1 Virus-Like Particle [VLP] Vaccine) in Adult Women (27- to 45-Year-Olds) Compared to Young Adult Women (16 to 26 Year Olds) |
| 76 | *Not randomised* | V503-008 | Not identified | NCT01254643 | A Phase III Open-label, Safety, Tolerability and Immunogenicity Study of a 9-Valent Human Papillomavirus (HPV) L1 Virus-Like Particle (VLP) Vaccine Administered to 9- to 15-Year-Old Japanese Preadolescent and Adolescent Girls |
| 77 | *Not randomised* | Not identified | 112677 | NCT01498627 | Cervarix Long-term Safety Surveillance Using the PGRx Information System (PGRx Study) |
| 78 | *Not randomised* | Not identified | 113522 | NCT01153906 | Post-marketing Safety Study of Autoimmune Diseases Following Cervarix® Vaccination in Females Aged 9-25 Years in the US |
| 79 | *Not randomised* | Not identified | 07-09-0344 | NCT00727636 | Pilot Study of Immunogenicity and Tolerability to the Quadrivalent Human Papillomavirus Virus-like Particle (VLP) Vaccine (Gardasil) Among IBD Patients on Immunosuppressive Therapy Compared to Healthy Children and Youth Adult Females |
| 80 | *Not randomised* | Not identified | 35384 CT.2006.33111.004 | NCT00944879 | Preparing for Adolescent HIV Vaccine Trials in South Africa: a Multi-centre Study to Evaluate Acceptability of the HPV Vaccine in Adolescents |
| 81 | *Not randomised* | Not identified | 0807-02 | NCT00767897 | The Impact of the Human Papilloma Virus in Paediatric Chronic Kidney Disease, Dialysis, and Transplant Patients |
| 82 | *Not randomised* | Not identified | MERCK_33610 | NCT00806676 | Antibody Response to Human Papillomavirus Recombinant Vaccine (Gardasil®) in Girls and Young Women with Chronic Kidney Disease |
| 83 | *Not randomised* | Not identified | 1000037024 | NCT02624349 | Immunogenicity and Safety of Human Papilloma Virus Vaccine in Solid Organ Transplant Recipients |
| 84 | *Not randomised* | Not identified | 091-2014 | NCT02382900 | Evaluation of a Two-dose Schedule of Quadrivalent Human Papilloma Virus (Types 6, 11, 16, 18) Recombinant Vaccine in 11-year-old Boys in Mexico City (Gardasil® Merck and Co.). |
| 85 | *Not randomised* | Not identified | Gardasil in JIA | NCT00573651 | Pilot Study of the Safety and Efficacy of Quadrivalent Human Papillomavirus Vaccine (Gardasil®) in Female Subjects with Juvenile Idiopathic Arthritis (JIA)/ Seronegative Arthritis |
| 86 | *Not randomised* | Not identified | GINI | NCT01924754 | Gardasil Immunogenicity with Needle-Free Injection |
| 87 | *Phase 1 study* | HPV-048 | 110659 | NCT00541970 | Evaluation of the Safety and Immunogenicity of GSK Biologicals' HPV Vaccine 580299 When Administered in Healthy Females Aged 9 - 25 Years Using an Alternative Schedule and an Alternative Dosing as Compared to the Standard Schedule and Dosing |
| 88 | *Phase 1 study* | HPV-PRO-004 | Not identified | NCT02405520 | A Phase I Randomized, Double-Blinded, Placebo Controlled Study to Evaluate the Safety and Immunogenicity of the Recombinant (E.coli) Human Papillomavirus Type 6/11 Bivalent Vaccine in Healthy Volunteers Aged 18-55 Years |
| 89 | *Phase 1 study* | V501-001 | Not identified | Not identified | No title. |
| 90 | *Phase 1 study* | V502-002 | 2009_552 | NCT00851643 | A Randomized, Double-Blind, Multicentre, Biphasic, Controlled With GARDASIL™ Dose-Escalation Study of Octavalent Human Papillomavirus (HPV) L1 Virus-Like Particle (VLP) Vaccine Adjuvanted With Amorphous Aluminium Hydroxyphosphate Sulphate (AAHS) and ISCOMATRIX™ (IMX) |
| 91 | *Phase 1 study* | Not identified | 109836 | NCT00478621 | A Multicentre Study to Evaluate the Safety and Immunogenicity of GSK Biologicals' HPV Vaccine (GSK1674330A) in Healthy Female Subjects Aged 18-25 Years. |
| 92 | *Phase 1 study* | Not identified | KCT0000604 | Not identified | Assessment of the safety, tolerance, and immunogenicity of EG-HPV (human papillomavirus vaccine) in healthy male adult volunteers: A double-blinded, randomized, adjuvant vehicle-controlled trial |
| 93 | *Prematurely terminated* | HPV-020-EPI | 114176 | NCT01290393 | Post-marketing Safety Study to Assess the Risk of Spontaneous Abortion Following Administration of CERVARIX in the United States and Canada |
| 94 | *Prematurely terminated* | HPV-078 | 117099 | NCT02082639 | Immunogenicity and Safety Study of GSK Biologicals' Human Papillomavirus (HPV) Vaccine (Cervarix™) (GSK-580299) When Co-administered With GSK Biologicals' Hepatitis A Vaccine (Havrix®) (GSK-208109) in Healthy Female Adolescents Aged 9-14 Years |
| 95 | *Prematurely terminated* | HPV-081 (HPV-048 follow-up) | 200255 | Not identified | A phase IIIb, open-label, non-randomised, multicentre study to assess the immunogenicity and safety of GSK Biologicals’ HPV-16/18 L1 VLP AS04 vaccine when administered intramuscularly according to a 2-dose schedule in healthy female adolescents or intramuscularly according to a 3-dose schedule in healthy female adults, 6.5 years after first vaccine dose in study HPV-048 PRI (110659). - HPV-081 EXT:048 Y6.5 |
| 96 | *Prematurely terminated* | V502-003 | 2006_503 | NCT00365443 | No title. |
| 97 | *Prematurely terminated* | Not identified | 113763 | Not identified | Post-marketing surveillance (PMS) of GlaxoSmithKline (GSK) Biologicals’ human papillomavirus (HPV) -16/18 vaccine, Cervarix™ when administered to healthy females according to the Prescribing Information in Sri Lanka |
| 98 | *No clinical study report obtained* | V501-002 | Not identified | Not identified | MRL Clinical Study Report (Synopsis): Safety tolerability and Immunogenicity of a Research Lot of HPV 16 Virus-Like Particle (VLP) Vaccine in College-Age Women (Protocol 002). |
| 99 | *No clinical study report obtained* | V501-004 | Not identified | Not identified | No title. |
| 100 | *No clinical study report obtained* | V501-028 | 2006_052 | NCT00411749 | V501 Phase II Immunogenicity Study in Females Aged 9 to 17 Years |
| 101 | *No clinical study report obtained* | V503-018 | Not identified | Not identified | No title. |
| 102 | *No clinical study report obtained* | V503-019 | Not identified | Not identified | No title. |
| 103 | *No clinical study report obtained* | V505-001 | 2007_567 | NCT00520598 | A Phase IIa Randomized, Double-Blind Controlled with Gardasil, Clinical Trial to Study the tolerability and Immunogenicity of V505 (a Multivalent Human Papilloma Virus [HPV] L1 Virus Like Particle [VLP] Vaccine) in Healthy 16 to 26-Year-Old Women |
| 104 | *No clinical study report obtained* | MENACWY-TT-054 | 113823 | NCT01755689 | Immunogenicity and Safety Study of GSK Biologicals' Meningococcal Vaccine 134612 With or Without Co-administration of Cervarix and Boostrix in Female Adolescents and Young Adults at 9-25 Years of Age |
| 105 | *No clinical study report obtained* | HPV-073 | 115887 | NCT01627561 | Safety and Immunogenicity of GSK Biologicals' HPV-16/18 L1 VLP AS04 Vaccine (GSK-580299) in Healthy Female Children 4-6 Years Old |
| 106 | *No clinical study report obtained* | HPV-007 | 580299/007 | NCT00120848 | Study of the Efficacy of Candidate HPV 16/18 VLP Vaccine in the Prevention of HPV-16 and/or HPV-18 Cervical Infection in Adolescent & Young Adult Women in North America and Brazil Vaccinated in Primary Study 580299/001 |
| 107 | *No clinical study report obtained* | HPV-009 | 580299/009 | NCT00128661 | Efficacy of the HPV-16/18 Vaccine: Final according to protocol results from the blinded phase of the randomized Costa Rica HPV-16/18 Vaccine Trial |
| 108 | *No clinical study report obtained* | HPV-011 | 580299/011 | NCT00309166 | An Observer-blind, Randomized, Controlled Study to Assess the Immunogenicity and Safety of GlaxoSmithKline Biologicals' HPV Vaccine Administered Intramuscularly According to a 0, 1, 6 Month Schedule in Healthy Male Subjects Aged 10-18 Years |
| 109 | *No clinical study report obtained* | HPV-013  follow-up | 104896 | NCT00316706 | A Long-term, Open Follow-up of the Immunogenicity and Safety of GSK Biologicals' HPV Vaccine (580299) in Healthy Female Subjects Vaccinated in Study HPV-013 |
| 110 | *No clinical study report obtained* | HPV-021 | 106069 | NCT00481767 | Study to Assess the Immunogenicity and Safety of GlaxoSmithKline Biologicals' HPV Vaccine GSK580299 in Healthy Female Subjects Aged 10-25 Years |
| 111 | *No clinical study report obtained* | HPV-036 | 105926 | NCT00345878 | Phase IIIb, Double-blind, Randomized, Controlled Study to Evaluate the Immunogenicity & Safety of GSK Biologicals' HPV-16/18 L1 VLP AS04 Vaccine Administered Intramuscularly (0, 1, 6 Month Schedule) in Healthy Women from Malaysia. |
| 112 | *No clinical study report obtained* | HPV-039 | 107638 | NCT00779766 | Efficacy, Immunogenicity and Safety of GlaxoSmithKline Biologicals' HPV GSK 580299 Vaccine in Healthy Chinese Female Subjects |
| 113 | *No clinical study report obtained* | HPV-PRO-002 | Not identified | NCT01356823 | A Randomized, Double-Blinded, Placebo-Controlled, Dose-Ranging Study of Recombinant Human Papillomavirus Virus 16/18 Bivalent Vaccine (E.coli) in Healthy Female Subjects Aged 18 to 25 Years |
| 114 | *No clinical study report obtained* | V501-005  follow-up | PMC2749988 | Not identified | Longer-term efficacy of a prophylactic monovalent human papillomavirus type 16 vaccine |
| 115 | *No clinical study report obtained* | V501-007 | 2006_516 | NCT00365716 | A Placebo-Controlled, Dose-Ranging Study of Quadrivalent HPV Virus-Like Particle (VLP) Vaccine in 16- to 23-Year-Old Women |
| 116 | *No clinical study report obtained* | V501-023 | 2005_066 | NCT00157950 | An Immunogenicity and Safety Study of Gardasil (V501 (Human Papillomavirus [Types 6, 11, 16, 18] Recombinant Vaccine)) in Females 9 to 23 Years of Age in Korea |
| 117 | *No clinical study report obtained* | V501-027 | 2006_032 | NCT00378560 | V501 Phase II Efficacy Study in Women Aged 18 to 26 |
| 118 | *No clinical study report obtained* | V501-030 | 2007_021 | NCT00496626 | An Immunogenicity and Safety Study of Quadrivalent HPV (Types 6, 11, 16, 18) Virus-Like Particle (VLP) Vaccine in Chinese Female Subjects Aged 9 to 45 Years and Male Subjects Aged 9 to 15 Years |
| 119 | *No clinical study report obtained* | V501-041 | 2009_532 | NCT00834106 | A Randomized, Placebo-Controlled, Double-Blind Study of Quadrivalent HPV (Types 6, 11, 16, 18) L1 Virus-Like Particle (VLP) Vaccine to Investigate the Safety, and Efficacy in Chinese 20 - to 45-Years-Old Women |
| 120 | *No clinical study report obtained* | V501-046 | Not identified | NCT01245764 | Evaluation of Safety and Immunogenicity of GARDASIL™ in Healthy Females Between 9 and 26 Years of Age in Sub Saharan Africa |
| 121 | *No clinical study report obtained* | Not identified | PMC4378717 | NCT01489527 | High HIV, HPV, and STI prevalence among young Western Cape, South African women: EVRI HIV prevention preparedness trial |

List of excluded non-industry funded studies

| **No.** | **Reason for exclusion** | **Study ID** | **NCT ID** | **Title** |
| --- | --- | --- | --- | --- |
| 1 | *Ineligible comparator* | 122.05.01 9427-L1802/1-21C | NCT01456715 | Immunogenicity and Safety of Gardasil and Twinrix Vaccines Co-administered or Administered a Month Apart, according to the 0, 6 Months Schedule and the Effect of a Third Dose of Gardasil or Cervarix Administered 42 Months Later. |
| 2 | *Ineligible comparator* | EUCTR2011-001871-37-DK | Not identified | Comparison of vaccine effects of two different vaccines against Human Papillomavirus in HIV infected people |
| 3 | *Ineligible comparator* | 2013/422 2013-002340-90 | NCT01914367 | Study of the Molecular Mechanisms Underlying the Cross-neutralizing Capacity of AS04-adjuvanted HPV Vaccine (Cervarix®) in Comparison With the Aluminium hydroxyphosphate Sulphate Adjuvanted HPV Vaccine (Gardasil®) |
| 4 | *Ineligible comparator* | ACTRN12608000339358 | Not identified | A pilot non-inferiority immunogenicity single blind randomised study of two human papillomavirus vaccines administered intradermally to females aged 18 to 26 years to bridge previous efficacy findings of intramuscularly administered vaccine |
| 5 | *Ineligible comparator* | CTRI/2013/11/004140 | Not identified | Comparison of Post-licensure safety surveillance of Bivalent and Quadrivalent Human Papillomavirus vaccines in Healthy Women. |
| 6 | *Ineligible comparator* | ESCUDDO | NCT03180034 | A Scientific Evaluation of One or Two Doses of Vaccine Against Human Papillomavirus: the ESCUDDO Study |
| 7 | *Ineligible comparator* | FASTER-Tialpan Study | NCT03105856 | HPV Vaccination Impact on Cervical Cancer Screening Program: FASTER-Tialpan Study in Mexico |
| 8 | *Ineligible comparator* | H07-00928 | NCT00501137 | A Controlled Trial to Assess the Immunogenicity of a Proposed Paediatric Dosing Schedule of Human Papillomavirus Vaccine |
| 9 | *Ineligible comparator* | HLS04/2011 | NCT01512784 | Long Term Immunogenicity of Quadrivalent Human Papillomavirus Vaccine (Gardasil®)in HIV-infected Adolescents and Young Adults vs. Healthy Adolescents and Young Adults: Non-randomized Controlled Clinical Trial |
| 10 | *Ineligible comparator* | HPV 2355 | NCT02567955 | Immunogenicity and Safety of Gardasil-9 and Cervarix When Administered to 9-10-year-old Subjects According to 0-6 Month Schedule |
| 11 | *Ineligible comparator* | HPV CSP01 | NCT00956553 | A Phase IV, Randomised Study to Evaluate the Immune Responses of UK Adolescent Girls Receiving Cervarix^TM^ or Gardasil^TM^ Human Papillomavirus Vaccines |
| 12 | *Ineligible comparator* | HPV01 | NCT00524745 | Comparison of the Immunogenicity and Reactogenicity of Alternative Schedules of Gardasil Vaccine to Prevent HPV Infection |
| 13 | *Ineligible comparator* | ICI-VPH-1 | NCT02009800 | ICI-VPH: Impact Des Calendriers d'Immunisation Contre Les HPV |
| 14 | *Ineligible comparator* | IRB00046117 | NCT01505049 | Immune Memory After Papillomavirus Vaccination (IMAP-I) Study. DMID 10-0014 |
| 15 | *Ineligible comparator* | ISRCTN98283094 | NCT00923702 | Randomised Trial of Two Versus Three Doses of Human Papillomavirus (HPV) Vaccine in India |
| 16 | *Ineligible comparator* | LTN0001 | NCT01386164 | Immune Response to Bivalent and Tetravalent Human Papillomavirus Vaccine in HIV Infected Adults |
| 17 | *Ineligible comparator* | MITU-001 | NCT01173900 | Delivery, Uptake and Acceptability of HPV Vaccination in Tanzanian Girls |
| 18 | *Ineligible comparator* | MITU-002 | NCT02834637 | A Dose Reduction Immuno-bridging and Safety Study of Two HPV Vaccines in Tanzanian Girls |
| 19 | *Ineligible comparator* | <https://doi.org/10.1089/jwh.2009.1753> | Not identified | Randomized trial of an alternate human papillomavirus vaccine administration schedule in college-aged women. |
| 20 | *Ineligible comparator* | <http://dx.doi.org/10.1080/21645515.2016.1277846> | Not identified | Reactogenicity of Cervarix and Gardasil human papillomavirus (HPV) vaccines in a randomized single blind trial in healthy UK adolescent females |
| 21 | *Ineligible comparator* | [PMID: 23770335 DOI: 10.1016/j.vaccine.2013.06.034](https://dx.doi.org/10.1016/j.vaccine.2013.06.034) | Not identified | A pilot randomized study to assess immunogenicity, reactogenicity, safety and tolerability of two human papillomavirus vaccines administered intramuscularly and intradermally to females aged 18-26 years. |
| 22 | *Ineligible participants* | A5298 11798 | NCT01461096 | A Randomized, Double-Blinded, Placebo-Controlled, Phase III Trial of the Quadrivalent HPV Vaccine to Prevent Anal Human Papillomavirus Infection in HIV-Infected Men and Women |
| 23 | *Ineligible participants* | HRPO-07-0648 | NCT00941889 | The Effect of Human Papillomavirus Vaccination on Recurrence Rates in HIV Positive Patients Treated for Anal Condylomata |
| 24 | *Ineligible participants* | ISRCTN14732216 | Not identified | Effectiveness of the quadrivalent human papillomavirus (qHPV) vaccine in HIV-positive Spanish men having sex with men (MSM): Double-bind randomised Clinical Trial |
| 25 | *Ineligible participants* | ISRCTN14732216 follow-up | Not identified | No title. |
| 26 | *Ineligible participants* | P1047 | NCT00339040 | Phase II Safety and Immunogenicity Study of Quadrivalent Human Papillomavirus (Types 6, 11, 16, 18) L1 Virus-Like Particle (VLP) Vaccine in HIV Infected Children 7 to 12 Years of Age |
| 27 | *Not comparative* | 342/2009 | NCT02296255 | Effective Surveillance and Impact of HPV Vaccination on Screening for Cervical Cancer in Tuscany |
| 28 | *Not completed/ongoing as of 1 July 2017* | 1603017415 | NCT02864147 | Treatment of High-Grade Pre-Neoplastic Cervical Lesions (CIN 2/3) Using a Novel "Prime and Pull" Strategy |
| 29 | *Not completed/ongoing as of 1 July 2017* | EUCTR2012-004007-13-DE | Not identified | A randomized, placebo-controlled, phase IIIb HPV vaccination trial with Gardasil® in patients with recurrent condylomata acuminata |
| 30 | *Not completed/ongoing as of 1 July 2017* | EUCTR2013-002009-70-NL | Not identified | Quadrivalent HPV vaccination after effective treatment of Anal Intraepithelial Neoplasia in HIV+ men - VACCAIN-P |
| 31 | *Not completed/ongoing as of 1 July 2017* | 2016-002083-13 | Not identified | An observational follow up study of a randomised parallel group phase IV study to evaluate the duration of the immune response to vaccine and non-vaccine HPV types in UK adolescent females who received either Cervarix or Gardasil Human Papillomavirus (HPV) vaccines. |
| 32 | *Not completed/ongoing as of 1 July 2017* | 2016-002455-20 | Not identified | Efficacy study of the quadrivalent Human Papilloma Virus (HPV) vaccine to prevent recurrence of External Genital Warts (EGW) in patients who were cured in the first place |
| 33 | *Not completed/ongoing as of 1 July 2017* | 999909106 09-C-N106 | NCT00867464 | Extended Follow-up of Young Women in Costa Rica Who Received Vaccination Against Human Papillomavirus Types 16 and 18 and Unvaccinated Controls |
| 34 | *Not completed/ongoing as of 1 July 2017* | ACTRN12613001207707 | Not identified | Pilot Study for a trial in Human Papillomavirus (HPV) vaccinated and unvaccinated women presenting for cervical screening of 6 yearly HPV screening versus 3-yearly cytology screening to assess the feasibility via recruitment rates, cross sectional positivity rate and laboratory implementation. |
| 35 | *Not completed/ongoing as of 1 July 2017* | CIHR-MOP-125949 | NCT01824537 | Transmission Reduction and Prevention With HPV Vaccination (TRAP-HPV) Study: A Randomized Controlled Trial of the Efficacy of HPV Vaccination in Preventing Transmission of HPV Infection in Heterosexual Couples |
| 36 | *Not completed/ongoing as of 1 July 2017* | GINI Study | NCT02363660 | A Randomized, Parallel Design Study to Compare the Safety and Effectiveness of Gardasil When Delivered Per Standard Practice (Full Dose, Intramuscular (IM) Delivery Using a Needle and Syringe) vs. Full Dose, IM Delivery Via Needle-free Jet Injection or Reduced Dose, Intradermal Delivery Via Needle-free Jet Injection |
| 37 | *Not completed/ongoing as of 1 July 2017* | Merck-MISP-53183 | NCT02750202 | Prophylactic Vaccines as Therapy: Prevention of Recurrence of Extensive Genital Warts |
| 38 | *Not completed/ongoing as of 1 July 2017* | NL45200.018.13 | NCT02087384 | Quadrivalent HPV Vaccination After Effective Treatment of Anal Intraepithelial Neoplasia in HIV+ Men |
| 39 | *Not randomised* | 883 | NCT01717118 | Evaluation of Immunogenicity Levels in Women With HPV Vaccine in Mexico |
| 40 | *Not randomised* | N01AI80006C | NCT01030562 | Immunogenicity of the HPV-6, 11, 16, 18 Vaccine Among Adolescent Girls Who Receive Vaccine Doses at Non-recommended Intervals and Factors Related to Non-adherence |
| 41 | *Not randomised* | 09-C-0024 | NCT00798265 | A Phase I Study of Quadrivalent Human Papilloma Virus (HPV) (Types 6, 11, 16, 18) Recombinant Vaccine in HIV-Infected and HIV-Negative Pre-Adolescents, Adolescents and Young Adults |
| 42 | *Not randomised* | [2014.5.FNRERC.5.SU](http://2014.5.FNRERC.5.SU) | NCT02276521 | Evaluation of Long-term Immunological Responses Following Reduced Dose Quadrivalent Human Papillomavirus (HPV) Vaccine Schedules: A Phase II/III Clinical Trial |
| 43 | *Not randomised* | A5240 | NCT00604175 | A Phase II Study to Evaluate the Immunogenicity and Safety of a Quadrivalent Human Papillomavirus Vaccine in HIV-1-Infected Females |
| 44 | *Not randomised* | ATN 064 | NCT00710593 | Immunogenicity, Safety, Tolerability, and Behavioural Consequences of an HPV-6, -11, -16, -18 Vaccine in HIV-Infected Young Women |
| 45 | *Not randomised* | DDEAMC | NCT00501189 | Gardasil Vaccination as Therapy in Low Grade Cervical Abnormalities |
| 46 | *Not randomised* | DRKS00005278 | Not identified | Immunity against Human Papillomavirus (HPV) in vaccinated and non-vaccinated probands |
| 47 | *Not randomised* | HP-00041372 | NCT00257738 | A Phase 1 Open Label, Dose Escalation Study to Evaluate the Effect of Four Doses of MAGE-A3/HPV 16 Trojan Peptides 0001 and 0002 Administered Subcutaneously in Combination With Montanide and GM-CSF on Immunological Response, Safety, Tolerability, and Preliminary Efficacy in Patients With Squamous Cell Carcinoma of the Head and Neck |
| 48 | *Not randomised* | HP-41372 | NCT00704041 | A Phase 1 Open Label, Dose Escalation Study to Evaluate the Effect of Four Doses of MAGE-A3/HPV 16 Trojan Peptides 0001 and 0002 Administered Subcutaneously |
| 49 | *Not randomised* | Not identified | NCT01896986 | HPV Immunisation Protecting Special Risk Group Patients From Cervical Cancer: 5 Year Follow-up Post-vaccination |
| 50 | *Not randomised* | NL26.113.000.08 | NCT00815282 | Immune Response After Human Papillomavirus Vaccination in Patients With Autoimmune Disease |
| 51 | *Not randomised* | NTWC/CREC/15035 | NCT02477254 | Long-term Immunogenicity of a Quadrivalent Human Papillomavirus (HPV) Vaccine in Patients With Systemic Lupus Erythematosus: a Case-control Study |
| 52 | *Not randomised* | Pro00014388 | NCT00862810 | Alternate Dosing Schedules Study for HPV Vaccine |
| 53 | *Not randomised* | Pro00014388_1 | NCT02280642 | Alternate Dosing Schedules Study for HPV Vaccine |
| 54 | *Not randomised* | SG09-EN01 | NCT00949572 | Characterisation of Human Disseminated Cellular and Humoral Immune Responses Following Sublingual or Intramuscular Deposition of Antigens |
| 55 | *Phase 1 study* | cycdc2016-4 | NCT02888418 | Random, Double Blind, Placebo Controlled Phase I Clinical Trials to Estimate the Safety and Preliminary Immunogenicity of Tetravalent Recombinant Human Papilloma Virus Vaccine (6,11,16,18 Type) (Hansenula Polymorpha) in Women of 9 to 30 Years Old and Men of 9to 17 Years Old |
| 56 | *Phase 1 study* | <https://doi.org/10.1093/jnci/93.4.284> | Not identified | Safety and immunogenicity trial in adult volunteers of a human papillomavirus 16 L1 virus-like particle vaccine. |
| 57 | *Phase 1 study* | PE1201 | NCT02564237 | A Phase I, Randomized, Observer-blind, Comparator-controlled, Study of the Safety and Immunogenicity of StreptAnova™ Vaccine in Healthy Adults Age ≥ 18 - 50 Years |
| 58 | *Prematurely terminated* | EUCTR2012-000445-12-DK | Not identified | Immune Response to Bivalent or Quadrivalent Human Papilloma Virus vaccination in Patients with Chronic Renal Failure - IPAR |
| 59 | *Prematurely terminated* | LIS144 | NCT01082861 | A Phase 4, Randomized, Open Label Clinical Trial to Determine Efficacy and Immunomodulation of Simultaneous HPV/HBV Vaccination Tango-trial) |
| 60 | *No report obtained* | ISRCTN32729817 | Not identified | Human papillomavirus infection: a randomised controlled trial of Imiquimod cream (5%) versus Podophyllotoxin cream (0.15%), in combination with quadrivalent human papillomavirus or control vaccination in the treatment and prevention of recurrence of anogenital warts (HIPvac Trial) |
| 61 | *No report obtained* | NCI-2012-02623 | NCT00091130 | An Exploratory Study to Evaluate the Effect of HPV 16 Vaccine on the Reduction of Viral Load in HPV 16 Positive Women With Persistent Viral Infection, But Low Grade Disease (ASCUS/LSIL) |
