# Supplementary material for: Benefits and harms of the human papillomavirus (HPV) vaccines: systematic review with meta-analyses of trial data from clinical study reports
Source: Syst Rev. 2020 Feb 28;9:43. doi: 10.1186/s13643-019-0983-y (PMC7047375; doi:10.1186/s13643-019-0983-y)
Supplement: Supplementary file 4 — Benefits and harms of the HPV vaccines—meta-analyses. (PDF 26103 kb) [file 13643_2019_983_MOESM4_ESM.pdf]

Additional file 4

Benefits and harms of the HPV vaccines: meta-analyses

Table of contents

1. Combined results of HPV-related outcomes 2
2. Anal outcomes 10
3. Cervical outcomes 11
4. Oropharyngeal outcomes 18
5. Penile outcomes 21
6. Vaginal outcomes 23
7. Vulvar outcomes 27
8. Referral procedures 31
9. Fatal harms 35
10. Serious harms 37
11. New onset diseases 82
12. General harms 126
13. Harms of special interest 167
14. Post hoc exploratory harm analyses 172
15. Definitions of harm categories 202
16. References 204

1. Combined results of HPV-related outcomes

1.1. All-cause mortality/deaths*: intention to treat analysis

Study or Subgroup	HPV vaccine Events	HPV vaccine Total	Control Events	Control Total	Weight	Risk Ratio IV, Random, 95% CI
**1.1.1 All-cause mortality/deaths: Cervarix vs. Al(OH)3 (females, age 24-72; follow-up until 36 months)**						
HPV-015: Cervarix vs. Al(OH)3 (f, 24-72; 36)	13	2882	5	2871	18.7%	2.59 [0.92, 7.26]
**Subtotal (95% CI)**		**2882**		**2871**	**18.7%**	**2.59 [0.92, 7.26]**
Total events	13		5			
Heterogeneity: Not applicable						
Test for overall effect: Z = 1.81 (P = 0.07)						
**1.1.2 All-cause mortality/deaths: Cervarix vs. Aimmugen: hepatitis A vaccine (females, age 19-25; follow-up until 7 months)**						
HPV-032: Cervarix vs. Aimmugen (f, 19-25; 7)	1	519	0	521	3.3%	3.01 [0.12, 73.76]
**Subtotal (95% CI)**		**519**		**521**	**3.3%**	**3.01 [0.12, 73.76]**
Total events	1		0			
Heterogeneity: Not applicable						
Test for overall effect: Z = 0.68 (P = 0.50)						
**1.1.3 All-cause mortality/deaths: Cervarix vs. Engerix: hepatitis B vaccine (females, age 26-46; follow-up until 12 months)**						
HPV-069: Cervarix vs. Engerix (f, 26-46; 12)	1	606	0	606	3.3%	3.00 [0.12, 73.50]
**Subtotal (95% CI)**		**606**		**606**	**3.3%**	**3.00 [0.12, 73.50]**
Total events	1		0			
Heterogeneity: Not applicable						
Test for overall effect: Z = 0.67 (P = 0.50)						
**1.1.4 All-cause mortality/deaths: Cervarix vs. Havrix: hepatitis A vaccine (females, age 15-25; follow-up until 48 months)**						
HPV-008: Cervarix vs. Havrix (f, 15-25; 48)	10	9319	13	9325	23.2%	0.77 [0.34, 1.75]
**Subtotal (95% CI)**		**9319**		**9325**	**23.2%**	**0.77 [0.34, 1.75]**
Total events	10		13			
Heterogeneity: Not applicable						
Test for overall effect: Z = 0.62 (P = 0.53)						
**1.1.5 All-cause mortality/deaths: Gardasil vs. AAHS (females, age 15-26; follow-up until 45 months)**						
V501-013: Gardasil vs. AAHS (f, 16-24; 45)	2	2723	3	2732	9.0%	0.67 [0.11, 4.00]
V501-015: Gardasil vs. AAHS (f, 15-26; 36)	7	6087	5	6080	16.6%	1.40 [0.44, 4.40]
**Subtotal (95% CI)**		**8810**		**8812**	**25.6%**	**1.13 [0.43, 2.96]**
Total events	9		8			
Heterogeneity: $Tau^2 = 0.00$; $Chi^2 = 0.46$, df = 1 (P = 0.50); $I^2 = 0\%$						
Test for overall effect: Z = 0.24 (P = 0.81)						
**1.1.6 All-cause mortality/deaths: Gardasil vs. AAHS (females, age 21-46; follow-up until 48 months)**						
V501-019: Gardasil vs. AAHS (f, 21-46; 48)	7	1911	1	1908	7.0%	6.99 [0.86, 56.75]
**Subtotal (95% CI)**		**1911**		**1908**	**7.0%**	**6.99 [0.86, 56.75]**
Total events	7		1			
Heterogeneity: Not applicable						
Test for overall effect: Z = 1.82 (P = 0.07)						
**1.1.7 All-cause mortality/deaths: Gardasil vs. AAHS (males, age 15-27; follow-up until 36 months)**						
V501-020: Gardasil vs. AAHS (m, 15-27; 36)	3	2032	10	2033	14.4%	0.30 [0.08, 1.09]
**Subtotal (95% CI)**		**2032**		**2033**	**14.4%**	**0.30 [0.08, 1.09]**
Total events	3		10			
Heterogeneity: Not applicable						
Test for overall effect: Z = 1.83 (P = 0.07)						
**1.1.8 All-cause mortality/deaths: HPV 16 vaccine vs. AAHS (females, age 16-25; follow-up until 48 months)**						
V501-005: HPV 16 vaccine vs. AAHS (f, 16-25; 48)	1	1204	1	1205	4.3%	1.00 [0.06, 15.98]
**Subtotal (95% CI)**		**1204**		**1205**	**4.3%**	**1.00 [0.06, 15.98]**
Total events	1		1			
Heterogeneity: Not applicable						
Test for overall effect: Z = 0.00 (P = 1.00)						
**Total (95% CI)**		**27283**		**27281**	**100.0%**	**1.19 [0.65, 2.19]**
Total events	45		38			
Heterogeneity: $Tau^2 = 0.24$; $Chi^2 = 11.49$, df = 8 (P = 0.18); $I^2 = 30\%$						
Test for overall effect: Z = 0.56 (P = 0.58)						
Test for subgroup differences: $Chi^2 = 11.02$, df = 7 (P = 0.14), $I^2 = 36.5\%$						

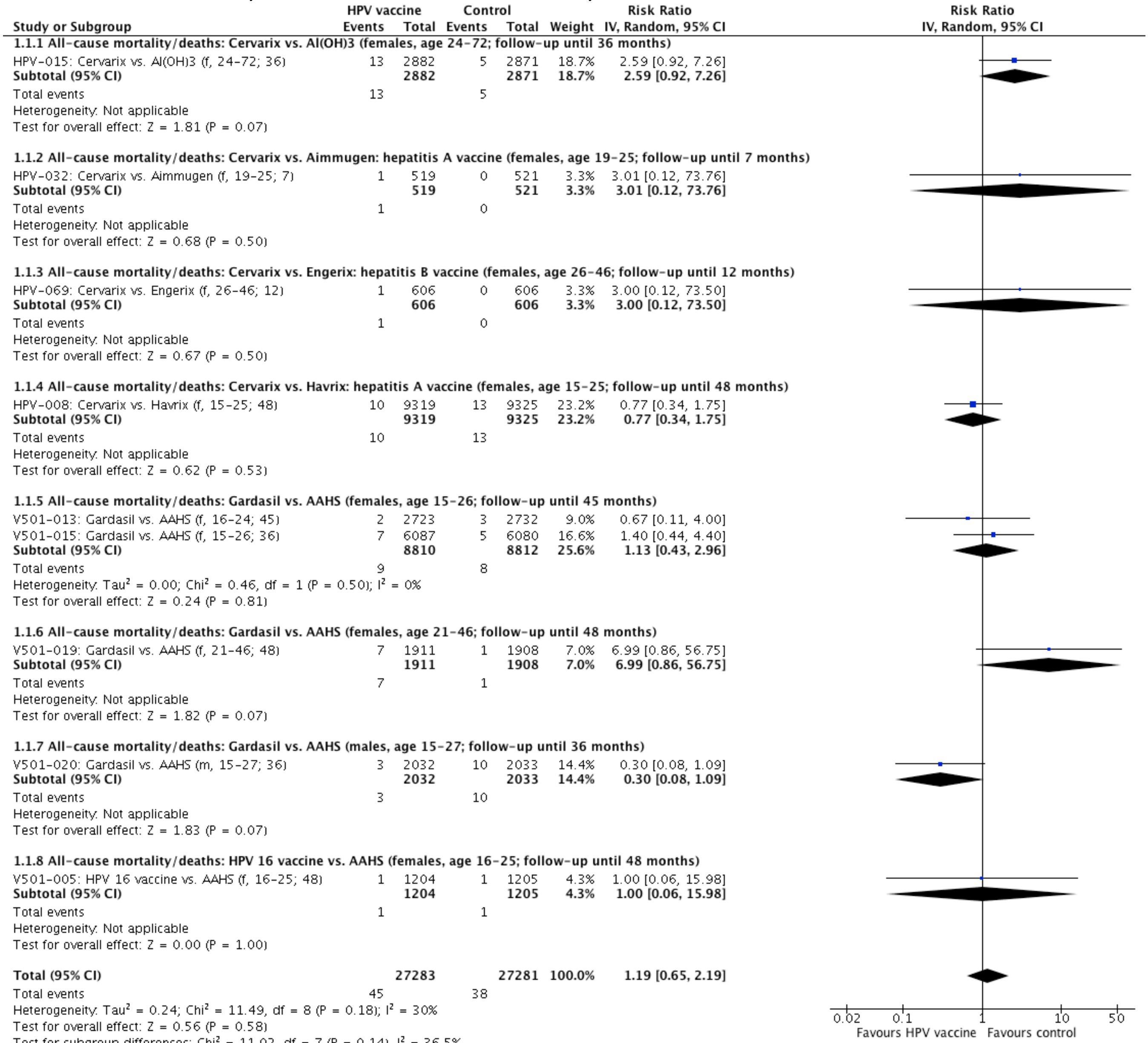

*1.1. Risk ratio for GlaxoSmithKline studies (i.e., HPV-0xx): 1.43 [0.65, 3.15]; risk ratio for Merck Sharp & Dohme studies (i.e., V50x-xxx): 1.08 [0.40, 2.96].

1.2. Mortality/deaths from HPV-related cancers (anal, cervical, oropharyngeal, penile, vaginal and vulvar) irrespective of HPV type*: intention to treat analysis

Study or Subgroup	HPV vaccine Events	HPV vaccine Total	Control Events	Control Total	Weight	Risk Ratio IV, Random, 95% CI
**1.2.1 Cervical cancer: Cervarix (females, age 24–72; follow-up until 36 months)**						
HPV-015: Cervarix vs. Al(OH)3 (f, 24-72; 36)	1	2882	0	2871	33.3%	2.99 [0.12, 73.33]
**Subtotal (95% CI)**		**2882**		**2871**	**33.3%**	**2.99 [0.12, 73.33]**
Total events	1		0			
Heterogeneity: Not applicable						
Test for overall effect: Z = 0.67 (P = 0.50)						
**1.2.2 Oropharyngeal cancer: Cervarix (females, age 24–72; follow-up until 36 months)**						
HPV-015: Cervarix vs. Al(OH)3 (f, 24-72; 36)	0	2882	1	2871	33.3%	0.33 [0.01, 8.15]
**Subtotal (95% CI)**		**2882**		**2871**	**33.3%**	**0.33 [0.01, 8.15]**
Total events	0		1			
Heterogeneity: Not applicable						
Test for overall effect: Z = 0.68 (P = 0.50)						
**1.2.3 Oropharyngeal cancer: Gardasil (females, age 21–46; follow-up until 48 months)**						
V501-019: Gardasil vs. AAHS (f, 21-46; 48)	1	1911	0	1908	33.3%	3.00 [0.12, 73.48]
**Subtotal (95% CI)**		**1911**		**1908**	**33.3%**	**3.00 [0.12, 73.48]**
Total events	1		0			
Heterogeneity: Not applicable						
Test for overall effect: Z = 0.67 (P = 0.50)						
**Total (95% CI)**		**7675**		**7650**	**100.0%**	**1.44 [0.23, 9.12]**
Total events	2		1			
Heterogeneity: $Tau^2 = 0.00$; $Chi^2 = 1.21$, df = 2 (P = 0.55); $I^2 = 0\%$						
Test for overall effect: Z = 0.39 (P = 0.70)						
Test for subgroup differences: $Chi^2 = 1.21$, df = 2 (P = 0.55), $I^2 = 0\%$						

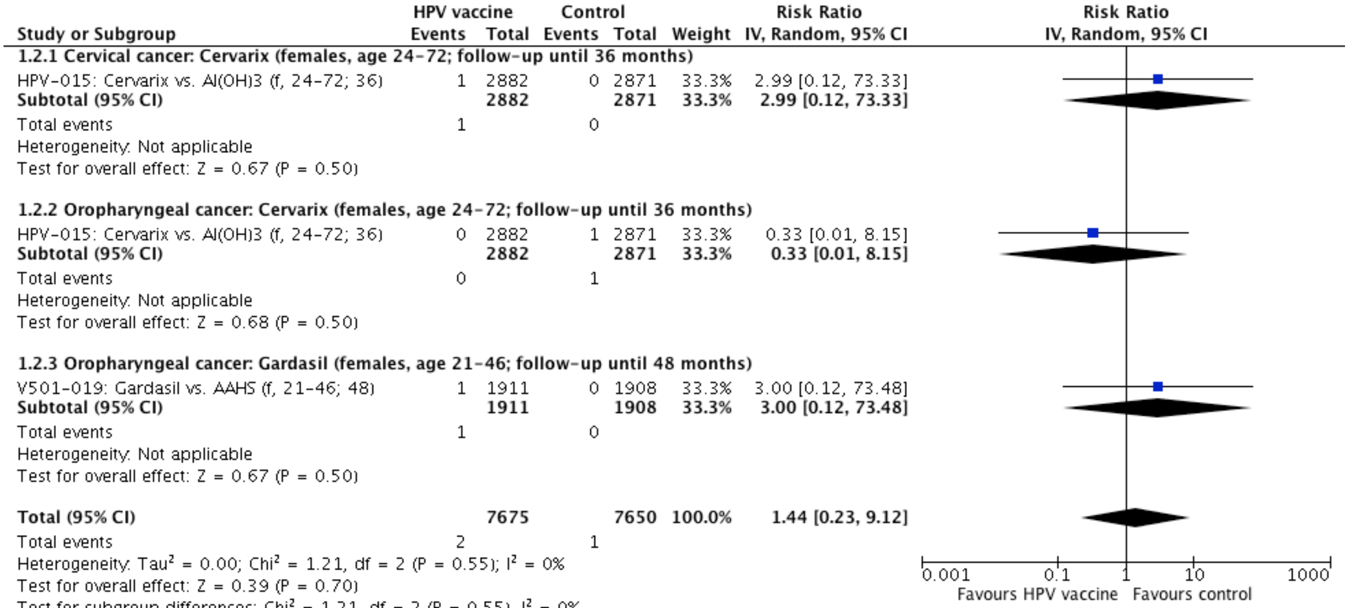

*1.2. Risk ratio for GlaxoSmithKline studies (i.e., HPV-0xx): 1.00 [0.10, 9.57]; risk ratio for Merck Sharp & Dohme studies (i.e., V50x-xxx): 3.00 [0.12, 73.48].

1.3. Incidence of HPV-related cancers (anal, cervical, oropharyngeal, penile, vaginal and vulvar) irrespective of HPV type*: intention to treat analysis

Study or Subgroup	HPV vaccine Events	HPV vaccine Total	Control Events	Control Total	Weight	Risk Ratio IV, Random, 95% CI
**1.3.1 Cervical cancer: Cervarix (females, age 24-72; follow-up until 36 months)**						
HPV-015: Cervarix vs. Al(OH)3 (f, 24-72; 36)	3	2882	0	2871	16.0%	6.97 [0.36, 134.94]
**Subtotal (95% CI)**		**2882**		**2871**	**16.0%**	**6.97 [0.36, 134.94]**
Total events	3		0			
Heterogeneity: Not applicable						
Test for overall effect: Z = 1.28 (P = 0.20)						
**1.3.2 Cervical cancer: Gardasil (females, age 21-46; follow-up until 48 months)**						
V501-019: Gardasil vs. AAHS (f, 21-46; 48)	0	1911	2	1908	15.3%	0.20 [0.01, 4.16]
**Subtotal (95% CI)**		**1911**		**1908**	**15.3%**	**0.20 [0.01, 4.16]**
Total events	0		2			
Heterogeneity: Not applicable						
Test for overall effect: Z = 1.04 (P = 0.30)						
**1.3.3 Oropharyngeal cancer: Cervarix (females, age 24-72; follow-up until 36 months)**						
HPV-015: Cervarix vs. Al(OH)3 (f, 24-72; 36)	0	2882	1	2871	13.7%	0.33 [0.01, 8.15]
**Subtotal (95% CI)**		**2882**		**2871**	**13.7%**	**0.33 [0.01, 8.15]**
Total events	0		1			
Heterogeneity: Not applicable						
Test for overall effect: Z = 0.68 (P = 0.50)						
**1.3.4 Oropharyngeal cancer: Gardasil (females, age 21-46; follow-up until 48 months)**						
V501-019: Gardasil vs. AAHS (f, 21-46; 48)	1	1911	0	1908	13.7%	3.00 [0.12, 73.48]
**Subtotal (95% CI)**		**1911**		**1908**	**13.7%**	**3.00 [0.12, 73.48]**
Total events	1		0			
Heterogeneity: Not applicable						
Test for overall effect: Z = 0.67 (P = 0.50)						
**1.3.5 Vaginal cancer: Cervarix (females, age 24-72; follow-up until 36 months)**						
HPV-015: Cervarix vs. Al(OH)3 (f, 24-72; 36)	1	2882	0	2871	13.7%	2.99 [0.12, 73.33]
**Subtotal (95% CI)**		**2882**		**2871**	**13.7%**	**2.99 [0.12, 73.33]**
Total events	1		0			
Heterogeneity: Not applicable						
Test for overall effect: Z = 0.67 (P = 0.50)						
**1.3.6 Vulvar cancer: Cervarix (females, age 15-25; follow-up until 48 months)**						
HPV-008: Cervarix vs. Havrix (f, 15-25; 48)	1	9319	0	9325	13.7%	3.00 [0.12, 73.68]
**Subtotal (95% CI)**		**9319**		**9325**	**13.7%**	**3.00 [0.12, 73.68]**
Total events	1		0			
Heterogeneity: Not applicable						
Test for overall effect: Z = 0.67 (P = 0.50)						
**1.3.7 Vulvar cancer: Gardasil (females, age 15-26; follow-up until 45 months)**						
V501-013: Gardasil vs. AAHS (f, 16-24; 45)	1	2723	0	2732	13.7%	3.01 [0.12, 73.85]
**Subtotal (95% CI)**		**2723**		**2732**	**13.7%**	**3.01 [0.12, 73.85]**
Total events	1		0			
Heterogeneity: Not applicable						
Test for overall effect: Z = 0.67 (P = 0.50)						
**Total (95% CI)**		**24510**		**24486**	**100.0%**	**1.68 [0.51, 5.49]**
Total events	7		3			
Heterogeneity: $Tau^2 = 0.00$; $Chi^2 = 4.27$, df = 6 (P = 0.64); $I^2 = 0\%$						
Test for overall effect: Z = 0.85 (P = 0.39)						
Test for subgroup differences: $Chi^2 = 4.27$, df = 6 (P = 0.64), $I^2 = 0\%$						

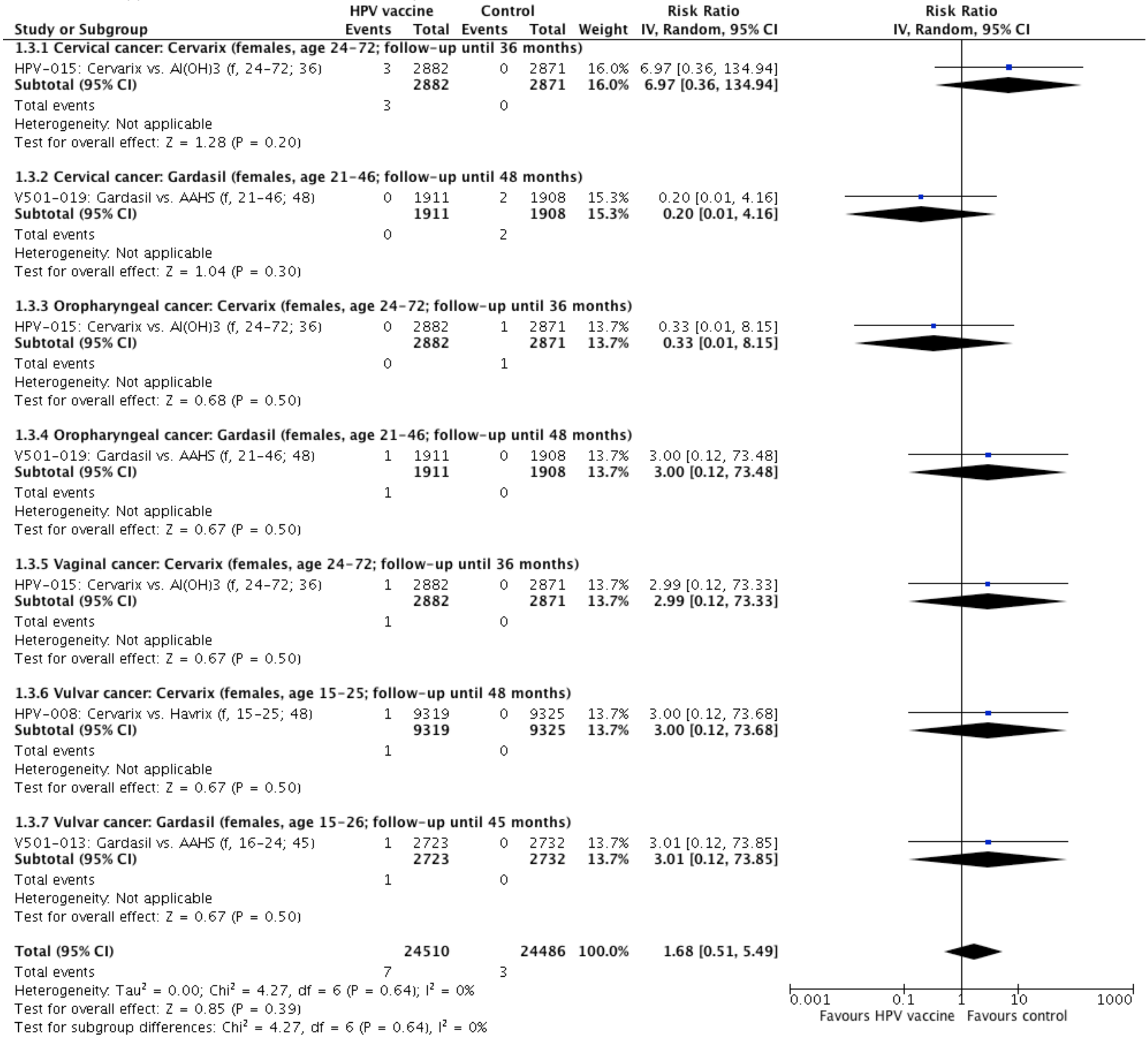

*1.3. Risk ratio for GlaxoSmithKline studies (i.e., HPV-0xx): 2.24 [0.47, 10.74]; risk ratio for Merck Sharp & Dohme studies (i.e., V50x-xxx): 1.14 [0.19, 6.99]. There were no reported cases of anal or penile cancer.

1.4. Incidence of HPV-related carcinoma in situ (anal intraepithelial neoplasia grade 3 [AIN3], cervical adenocarcinoma in situ [AIS], cervical intraepithelial neoplasia grade 3 [CIN3], penile intraepithelial neoplasia grade 3 [PIN3], vaginal intraepithelial neoplasia grade 3 [VIN3] and vulvar intraepithelial neoplasia grade 3 [VaIN3]) irrespective of HPV type*: intention to treat analysis

	HPV vaccine		Control			Risk Ratio
**Study or Subgroup**	**Events**	**Total**	**Events**	**Total**	**Weight**	**IV, Random, 95% CI**
**1.4.1 AIS: Cervarix (females, age 15–25; follow-up until 48 months)**						
HPV-008: Cervarix vs. Havrix (f, 15–25; 48)	3	9319	13	9325	5.2%	0.23 [0.07, 0.81]
**Subtotal (95% CI)**		**9319**		**9325**	**5.2%**	**0.23 [0.07, 0.81]**
Total events	3		13			
Heterogeneity: Not applicable						
Test for overall effect: Z = 2.29 (P = 0.02)						
**1.4.2 AIS: Gardasil (females, age 15–26; follow-up until 45 months)**						
V501-013: Gardasil vs. AAHS (f, 16–24; 45)	1	2723	6	2732	2.1%	0.17 [0.02, 1.39]
V501-015: Gardasil vs. AAHS (f, 15–26; 36)	5	6087	10	6080	6.6%	0.50 [0.17, 1.46]
**Subtotal (95% CI)**		**8810**		**8812**	**8.7%**	**0.40 [0.15, 1.04]**
Total events	6		16			
Heterogeneity: $Tau^2 = 0.00$; $Chi^2 = 0.82$, df = 1 (P = 0.37); $I^2 = 0\%$						
Test for overall effect: Z = 1.88 (P = 0.06)						
**1.4.3 AIS: Gardasil (females, age 21–46; follow-up until 48 months)**						
V501-019: Gardasil vs. AAHS (f, 21–46; 48)	0	1911	2	1908	1.1%	0.20 [0.01, 4.16]
**Subtotal (95% CI)**		**1911**		**1908**	**1.1%**	**0.20 [0.01, 4.16]**
Total events	0		2			
Heterogeneity: Not applicable						
Test for overall effect: Z = 1.04 (P = 0.30)						
**1.4.4 CIN3: Cervarix (females, age 15–25; follow-up until 48 months)**						
HPV-008: Cervarix vs. Havrix (f, 15–25; 48)	83	9319	145	9325	22.6%	0.57 [0.44, 0.75]
**Subtotal (95% CI)**		**9319**		**9325**	**22.6%**	**0.57 [0.44, 0.75]**
Total events	83		145			
Heterogeneity: Not applicable						
Test for overall effect: Z = 4.07 (P < 0.0001)						
**1.4.5 CIN3: Gardasil (females, age 15–26; follow-up until 45 months)**						
V501-013: Gardasil vs. AAHS (f, 16–24; 45)	84	2723	76	2732	21.5%	1.11 [0.82, 1.51]
V501-015: Gardasil vs. AAHS (f, 15–26; 36)	153	6087	208	6080	24.1%	0.73 [0.60, 0.90]
**Subtotal (95% CI)**		**8810**		**8812**	**45.7%**	**0.89 [0.59, 1.33]**
Total events	237		284			
Heterogeneity: $Tau^2 = 0.07$; $Chi^2 = 4.79$, df = 1 (P = 0.03); $I^2 = 79\%$						
Test for overall effect: Z = 0.58 (P = 0.56)						
**1.4.6 CIN3: Gardasil (females, age 21–46; follow-up until 48 months)**						
V501-019: Gardasil vs. AAHS (f, 21–46; 48)	38	1911	30	1908	16.8%	1.26 [0.79, 2.03]
**Subtotal (95% CI)**		**1911**		**1908**	**16.8%**	**1.26 [0.79, 2.03]**
Total events	38		30			
Heterogeneity: Not applicable						
Test for overall effect: Z = 0.97 (P = 0.33)						
**Total (95% CI)**		**40080**		**40090**	**100.0%**	**0.73 [0.53, 1.00]**
Total events	367		490			
Heterogeneity: $Tau^2 = 0.10$; $Chi^2 = 21.43$, df = 7 (P = 0.003); $I^2 = 67\%$						
Test for overall effect: Z = 1.94 (P = 0.05)						
Test for subgroup differences: $Chi^2 = 14.44$, df = 5 (P = 0.01), $I^2 = 65.4\%$						

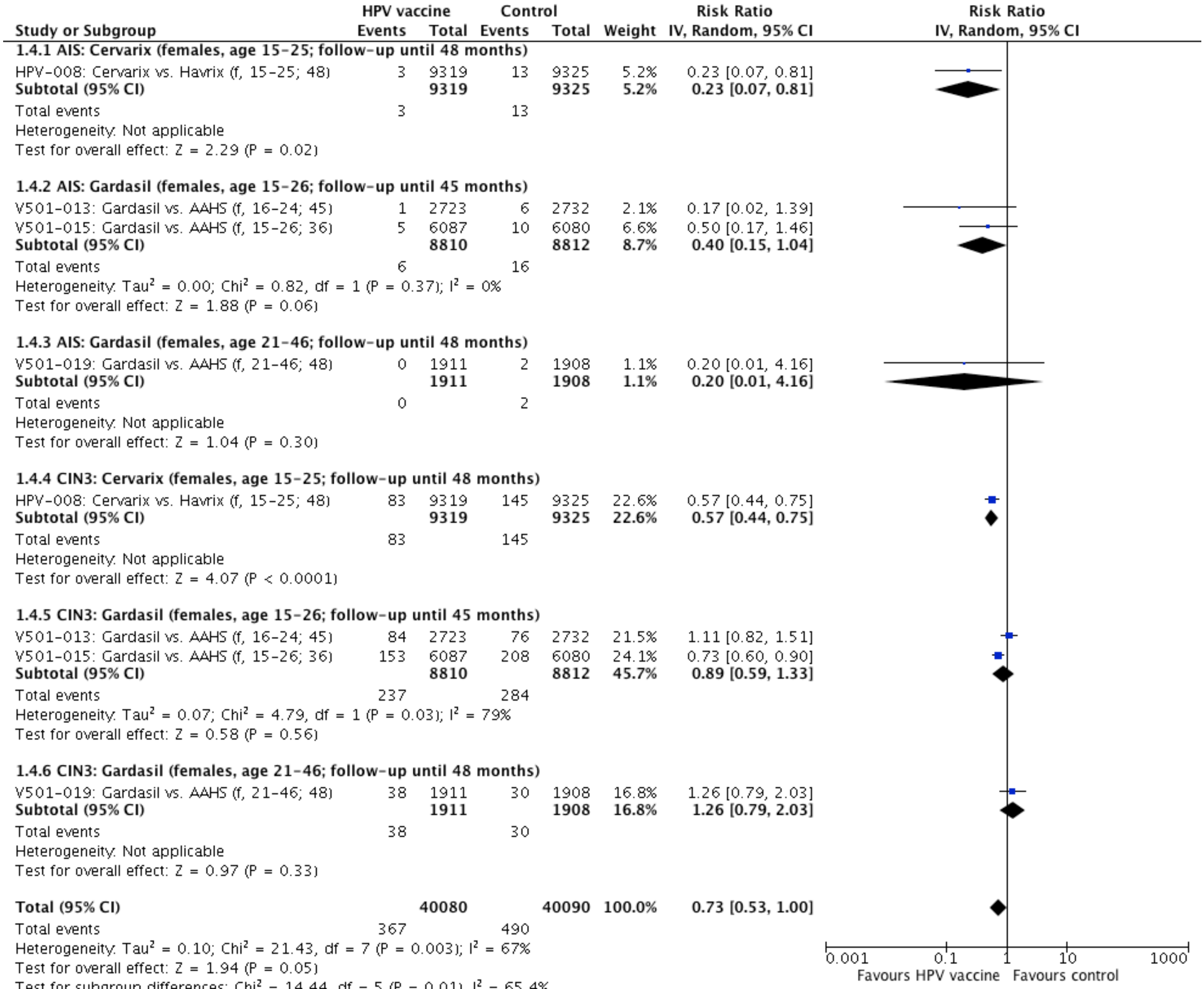

*1.4. Risk ratio for GlaxoSmithKline studies (i.e., HPV-0xx): **0.45 [0.21, 0.99]**; risk ratio for Merck Sharp & Dohme studies (i.e., V50x-xxx): 0.87 [0.62, 1.22]. There were no reports of AIN3, PIN3, VIN3 or VaIN3 irrespective of HPV type.

1.5. Incidence of HPV-related moderate intraepithelial neoplasia (anal intraepithelial neoplasia grade 2 [AIN2], cervical intraepithelial neoplasia grade 2 [CIN2], penile intraepithelial neoplasia grade 2 [PIN2], vaginal intraepithelial neoplasia grade 2 [VIN2] and vulvar intraepithelial neoplasia grade 2 [VaIN2]) irrespective of HPV type*: intention to treat analysis

Study or Subgroup	HPV vaccine Events	HPV vaccine Total	Control Events	Control Total	Weight	Risk Ratio IV, Random, 95% CI
**1.5.1 CIN2: Cervarix (females, age 15-25; follow-up until 48 months)**						
HPV-008: Cervarix vs. Havrix (f, 15-25; 48)	201	9319	370	9325	28.2%	0.54 [0.46, 0.64]
**Subtotal (95% CI)**		**9319**		**9325**	**28.2%**	**0.54 [0.46, 0.64]**
Total events	201		370			
Heterogeneity: Not applicable						
Test for overall effect: Z = 7.06 (P < 0.00001)						
**1.5.2 CIN2: Gardasil (females, age 15-26; follow-up until 45 months)**						
V501-013: Gardasil vs. AAHS (f, 16-24; 45)	110	2723	125	2732	25.7%	0.88 [0.69, 1.13]
V501-015: Gardasil vs. AAHS (f, 15-26; 36)	185	6087	238	6080	27.6%	0.78 [0.64, 0.94]
**Subtotal (95% CI)**		**8810**		**8812**	**53.3%**	**0.81 [0.70, 0.95]**
Total events	295		363			
Heterogeneity: $Tau^2 = 0.00$; $Chi^2 = 0.64$, df = 1 (P = 0.42); $I^2 = 0\%$						
Test for overall effect: Z = 2.68 (P = 0.007)						
**1.5.3 CIN2: Gardasil (females, age 21-46; follow-up until 48 months)**						
V501-019: Gardasil vs. AAHS (f, 21-46; 48)	42	1911	30	1908	18.5%	1.40 [0.88, 2.22]
**Subtotal (95% CI)**		**1911**		**1908**	**18.5%**	**1.40 [0.88, 2.22]**
Total events	42		30			
Heterogeneity: Not applicable						
Test for overall effect: Z = 1.41 (P = 0.16)						
**Total (95% CI)**		**20040**		**20045**	**100.0%**	**0.81 [0.59, 1.11]**
Total events	538		763			
Heterogeneity: $Tau^2 = 0.09$; $Chi^2 = 21.50$, df = 3 (P < 0.0001); $I^2 = 86\%$						
Test for overall effect: Z = 1.31 (P = 0.19)						
Test for subgroup differences: $Chi^2 = 20.86$, df = 2 (P < 0.0001), $I^2 = 90.4\%$						

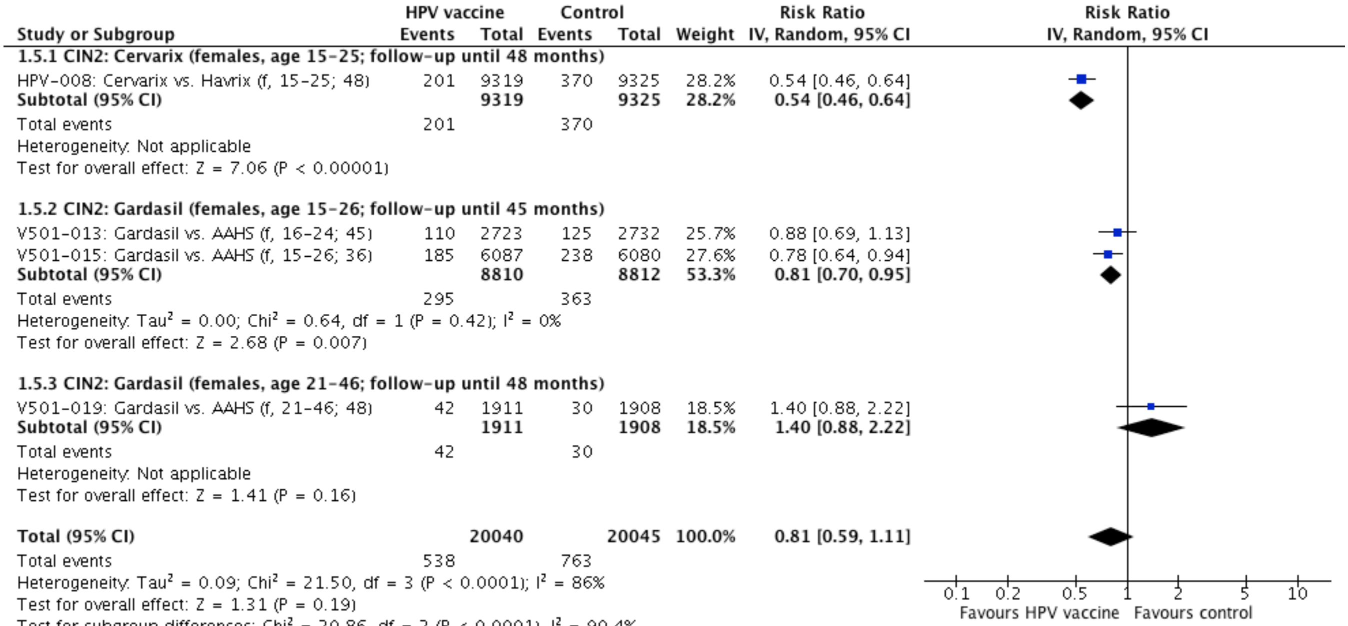

*1.5. Risk ratio for GlaxoSmithKline studies (i.e., HPV-0xx): **0.54 [0.46, 0.64]**; risk ratio for Merck Sharp & Dohme studies (i.e., V50x-xxx): 0.92 [0.70, 1.19]. There were no reports of AIN2, PIN2, VIN2 or VaIN2 irrespective of HPV type.

1.6. Incidence of HPV-related carcinoma in situ or worse (AIN3$^+$, CIN3$^+$, PIN3$^+$, VIN3$^+$, VaIN3$^+$) irrespective of HPV type*: intention to treat analysis

Study or Subgroup	HPV vaccine Events	HPV vaccine Total	Control Events	Control Total	Weight	Risk Ratio IV, Random, 95% CI
**1.6.1 CIN3+: Cervarix (females, age 15–25; follow-up until 48 months)**						
HPV-008: Cervarix vs. Havrix (f, 15–25; 48)	86	9319	158	9325	24.5%	0.54 [0.42, 0.71]
**Subtotal (95% CI)**		**9319**		**9325**	**24.5%**	**0.54 [0.42, 0.71]**
Total events	86		158			
Heterogeneity: Not applicable						
Test for overall effect: Z = 4.56 (P < 0.00001)						
**1.6.2 CIN3+: Cervarix (females, age 22–29; follow-up until 12 months)**						
HPV-063: Cervarix vs. Aimmugen (f, 22–29; 12)	9	464	14	463	8.7%	0.64 [0.28, 1.47]
**Subtotal (95% CI)**		**464**		**463**	**8.7%**	**0.64 [0.28, 1.47]**
Total events	9		14			
Heterogeneity: Not applicable						
Test for overall effect: Z = 1.05 (P = 0.29)						
**1.6.3 CIN3+: Gardasil (females, age 15–26; follow-up until 45 months)**						
V501-013: Gardasil vs. AAHS (f, 16–24; 45)	84	2723	78	2732	22.9%	1.08 [0.80, 1.46]
V501-015: Gardasil vs. AAHS (f, 15–26; 36)	155	6087	214	6080	26.6%	0.72 [0.59, 0.89]
**Subtotal (95% CI)**		**8810**		**8812**	**49.6%**	**0.87 [0.59, 1.29]**
Total events	239		292			
Heterogeneity: Tau^2 = 0.06; Chi^2 = 4.62, df = 1 (P = 0.03); I^2 = 78%						
Test for overall effect: Z = 0.70 (P = 0.48)						
**1.6.4 CIN3+: Gardasil (females, age 21–46; follow-up until 48 months)**						
V501-019: Gardasil vs. AAHS (f, 21–46; 48)	38	1911	34	1908	17.3%	1.12 [0.71, 1.76]
**Subtotal (95% CI)**		**1911**		**1908**	**17.3%**	**1.12 [0.71, 1.76]**
Total events	38		34			
Heterogeneity: Not applicable						
Test for overall effect: Z = 0.47 (P = 0.64)						
**Total (95% CI)**		**20504**		**20508**	**100.0%**	**0.79 [0.59, 1.05]**
Total events	372		498			
Heterogeneity: Tau^2 = 0.07; Chi^2 = 14.47, df = 4 (P = 0.006); I^2 = 72%						
Test for overall effect: Z = 1.62 (P = 0.11)						
Test for subgroup differences: Chi^2 = 8.74, df = 3 (P = 0.03), I^2 = 65.7%						

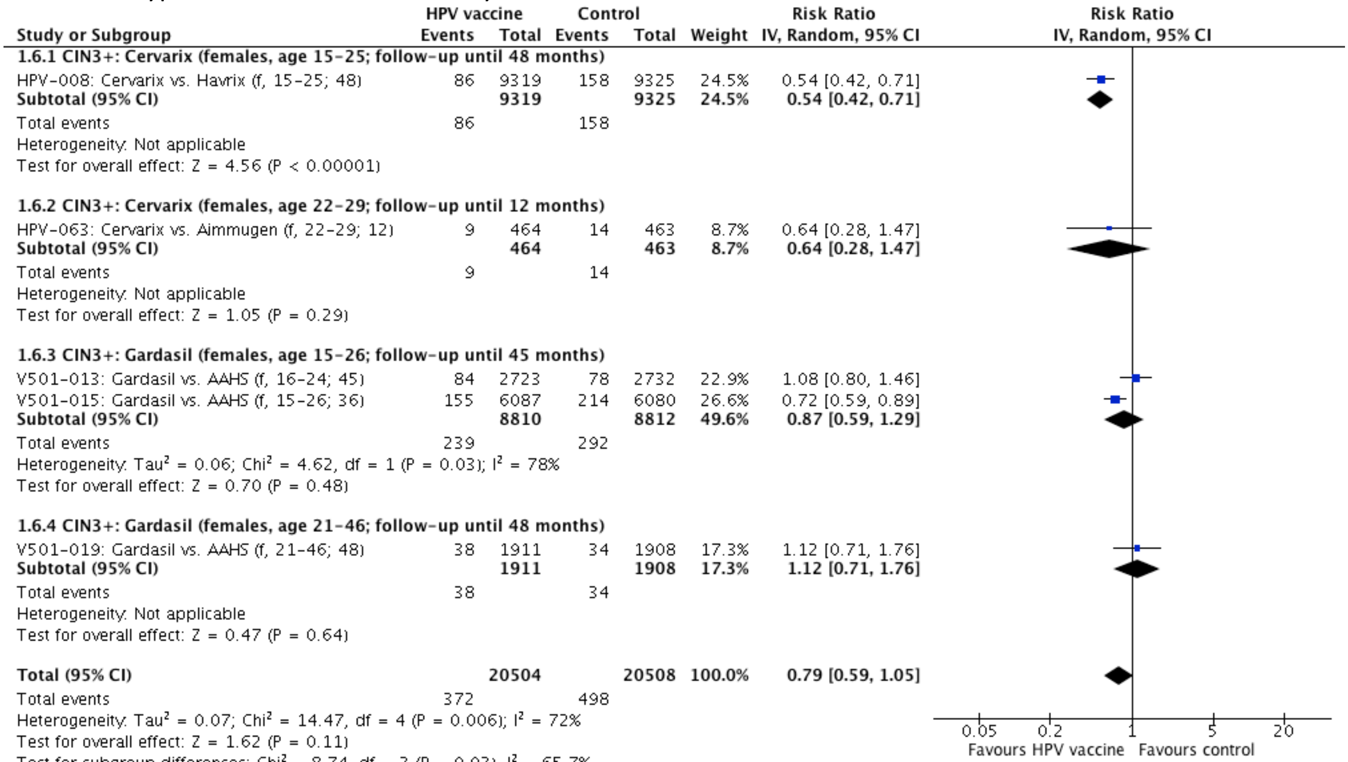

*1.6. Risk ratio for GlaxoSmithKline studies (i.e., HPV-0xx): **0.55 [0.43, 0.71]**; risk ratio for Merck Sharp & Dohme studies (i.e., V50x-xxx): 0.92 [0.67, 1.26]. There were no reports of AIN3$^+$, PIN3$^+$, VIN3$^+$ or VaIN3$^+$ irrespective of HPV type.

1.7. Incidence of HPV-related moderate intraepithelial neoplasia or worse (AIN2$^+$, CIN2$^+$, PIN2$^+$, VIN2$^+$, VaIN2$^+$) irrespective of HPV type*: intention to treat analysis

Study or Subgroup	HPV vaccine Events	HPV vaccine Total	Control Events	Control Total	Weight	Risk Ratio IV, Random, 95% CI
**1.7.1 CIN2+: Cervarix (females, age 15-25; follow-up until 48 months)**						
HPV-008: Cervarix vs. Havrix (f, 15-25; 48)	287	9319	428	9325	18.0%	0.67 [0.58, 0.78]
**Subtotal (95% CI)**		**9319**		**9325**	**18.0%**	**0.67 [0.58, 0.78]**
Total events	287		428			
Heterogeneity: Not applicable						
Test for overall effect: Z = 5.33 (P < 0.00001)						
**1.7.2 CIN2+: Cervarix (females, age 22-29; follow-up until 12 months)**						
HPV-063: Cervarix vs. Aimmugen (f, 22-29; 12)	19	464	41	463	6.4%	0.46 [0.27, 0.78]
**Subtotal (95% CI)**		**464**		**463**	**6.4%**	**0.46 [0.27, 0.78]**
Total events	19		41			
Heterogeneity: Not applicable						
Test for overall effect: Z = 2.86 (P = 0.004)						
**1.7.3 CIN2+: Cervarix (females, age 24-72; follow-up until 36 months)**						
HPV-015: Cervarix vs. Al(OH)3 (f, 24-72; 36)	103	2882	108	2871	13.4%	0.95 [0.73, 1.24]
**Subtotal (95% CI)**		**2882**		**2871**	**13.4%**	**0.95 [0.73, 1.24]**
Total events	103		108			
Heterogeneity: Not applicable						
Test for overall effect: Z = 0.38 (P = 0.70)						
**1.7.4 CIN2+: Gardasil (females, age 15-26; follow-up until 45 months)**						
V501-013: Gardasil vs. AAHS (f, 16-24; 45)	152	2723	166	2732	15.4%	0.92 [0.74, 1.14]
V501-015: Gardasil vs. AAHS (f, 15-26; 36)	269	6087	350	6080	17.7%	0.77 [0.66, 0.90]
**Subtotal (95% CI)**		**8810**		**8812**	**33.1%**	**0.83 [0.70, 0.98]**
Total events	421		516			
Heterogeneity: Tau² = 0.01; Chi² = 1.78, df = 1 (P = 0.18); I² = 44%						
Test for overall effect: Z = 2.15 (P = 0.03)						
**1.7.5 CIN2+: Gardasil (females, age 21-46; follow-up until 48 months)**						
V501-019: Gardasil vs. AAHS (f, 21-46; 48)	62	1911	51	1908	10.1%	1.21 [0.84, 1.75]
**Subtotal (95% CI)**		**1911**		**1908**	**10.1%**	**1.21 [0.84, 1.75]**
Total events	62		51			
Heterogeneity: Not applicable						
Test for overall effect: Z = 1.04 (P = 0.30)						
**1.7.6 PIN2+: Gardasil (males, age 15-27; follow-up until 36 months)**						
V501-020: Gardasil vs. AAHS (m, 15-27; 36)	3	2032	3	2033	1.0%	1.00 [0.20, 4.95]
**Subtotal (95% CI)**		**2032**		**2033**	**1.0%**	**1.00 [0.20, 4.95]**
Total events	3		3			
Heterogeneity: Not applicable						
Test for overall effect: Z = 0.00 (P = 1.00)						
**1.7.7 VIN2+: Gardasil (females, age 15-26; follow-up until 45 months)**						
V501-013: Gardasil vs. AAHS (f, 16-24; 45)	12	2723	15	2732	3.7%	0.80 [0.38, 1.71]
V501-015: Gardasil vs. AAHS (f, 15-26; 36)	6	6087	21	6080	2.7%	0.29 [0.12, 0.71]
**Subtotal (95% CI)**		**8810**		**8812**	**6.4%**	**0.49 [0.18, 1.36]**
Total events	18		36			
Heterogeneity: Tau² = 0.35; Chi² = 2.94, df = 1 (P = 0.09); I² = 66%						
Test for overall effect: Z = 1.37 (P = 0.17)						
**1.7.8 VaIN2+: Gardasil (females, age 15-26; follow-up until 45 months)**						
V501-013: Gardasil vs. AAHS (f, 16-24; 45)	8	2723	17	2732	3.1%	0.47 [0.20, 1.09]
V501-015: Gardasil vs. AAHS (f, 15-26; 36)	6	6087	9	6080	2.1%	0.67 [0.24, 1.87]
**Subtotal (95% CI)**		**8810**		**8812**	**5.2%**	**0.54 [0.28, 1.04]**
Total events	14		26			
Heterogeneity: Tau² = 0.00; Chi² = 0.26, df = 1 (P = 0.61); I² = 0%						
Test for overall effect: Z = 1.85 (P = 0.06)						
**1.7.9 VaIN2+: Gardasil (females, age 21-46; follow-up until 48 months)**						
V501-019: Gardasil vs. AAHS (f, 21-46; 48)	3	1911	1	1908	0.5%	3.00 [0.31, 28.77]
**Subtotal (95% CI)**		**1911**		**1908**	**0.5%**	**3.00 [0.31, 28.77]**
Total events	3		1			
Heterogeneity: Not applicable						
Test for overall effect: Z = 0.95 (P = 0.34)						
**1.7.10 VIN2+ or VaIN2+: Cervarix (females, age 15-25; follow-up until 48 months)**						
HPV-008: Cervarix vs. Havrix (f, 15-25; 48)	22	9319	29	9325	6.0%	0.76 [0.44, 1.32]
**Subtotal (95% CI)**		**9319**		**9325**	**6.0%**	**0.76 [0.44, 1.32]**
Total events	22		29			
Heterogeneity: Not applicable						
Test for overall effect: Z = 0.98 (P = 0.33)						
**Total (95% CI)**		**54268**		**54269**	**100.0%**	**0.78 [0.66, 0.91]**
Total events	952		1239			
Heterogeneity: Tau² = 0.03; Chi² = 25.46, df = 12 (P = 0.01); I² = 53%						
Test for overall effect: Z = 3.11 (P = 0.002)						
Test for subgroup differences: Chi² = 19.34, df = 9 (P = 0.02), I² = 53.5%						

Risk Ratio IV, Random, 95% CI

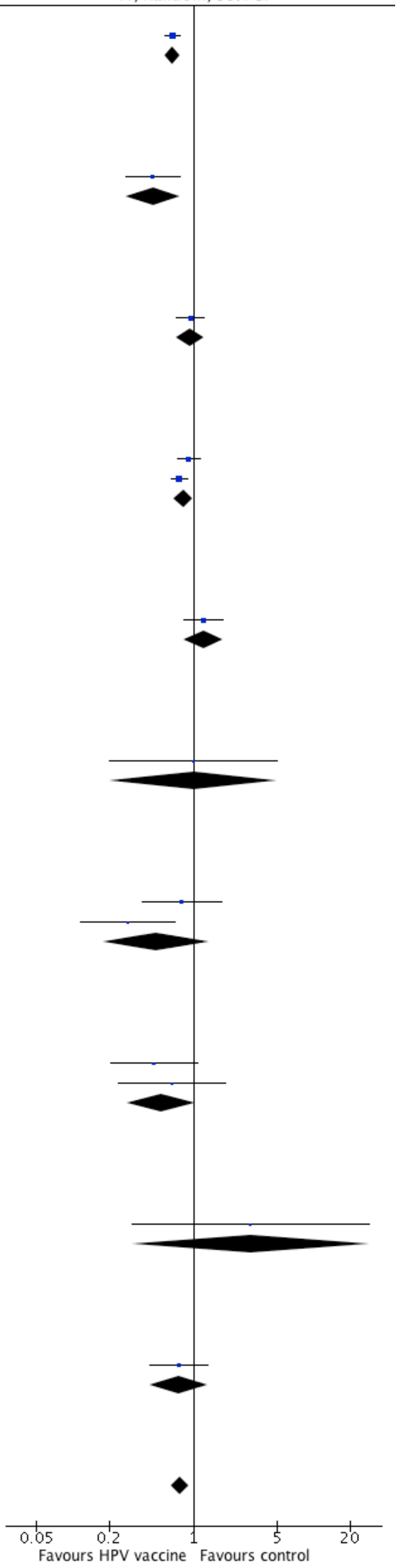

*1.7. Risk ratio for GlaxoSmithKline studies (i.e., HPV-0xx): **0.72 [0.55, 0.93]**; risk ratio for Merck Sharp & Dohme studies (i.e., V50x-xxx): 0.82 [0.66, 1.02]. There were no reports of AIN2$^+$ irrespective of HPV type.

1.8. Incidence of HPV-related external genital lesions (EGL) irrespective of HPV type*: intention to treat analysis

Study or Subgroup	HPV vaccine Events	HPV vaccine Total	Control Events	Control Total	Weight	Risk Ratio IV, Random, 95% CI
**1.8.1 EGL: Gardasil (females, age 15-26; follow-up until 45 months)**						
V501-013: Gardasil vs. AAHS (f, 16-24; 45)	110	2723	180	2732	28.8%	0.61 [0.49, 0.77]
V501-015: Gardasil vs. AAHS (f, 15-26; 36)	118	6087	293	6080	29.3%	0.40 [0.33, 0.50]
**Subtotal (95% CI)**		**8810**		**8812**	**58.1%**	**0.50 [0.33, 0.75]**
Total events	228		473			
Heterogeneity: $Tau^2 = 0.08$; $Chi^2 = 6.97$, df = 1 (P = 0.008); $I^2 = 86\%$						
Test for overall effect: Z = 3.33 (P = 0.0009)						
**1.8.2 EGL: Gardasil (females, age 21-46; follow-up until 48 months)**						
V501-019: Gardasil vs. AAHS (f, 21-46; 48)	23	1911	17	1908	17.3%	1.35 [0.72, 2.52]
**Subtotal (95% CI)**		**1911**		**1908**	**17.3%**	**1.35 [0.72, 2.52]**
Total events	23		17			
Heterogeneity: Not applicable						
Test for overall effect: Z = 0.95 (P = 0.34)						
**1.8.3 EGL: Gardasil (males, age 15-27; follow-up until 36 months)**						
V501-020: Gardasil vs. AAHS (m, 15-27; 36)	38	2032	92	2033	24.6%	0.41 [0.28, 0.60]
**Subtotal (95% CI)**		**2032**		**2033**	**24.6%**	**0.41 [0.28, 0.60]**
Total events	38		92			
Heterogeneity: Not applicable						
Test for overall effect: Z = 4.64 (P < 0.00001)						
**Total (95% CI)**		**12753**		**12753**	**100.0%**	**0.56 [0.39, 0.82]**
Total events	289		582			
Heterogeneity: $Tau^2 = 0.12$; $Chi^2 = 17.84$, df = 3 (P = 0.0005); $I^2 = 83\%$						
Test for overall effect: Z = 2.95 (P = 0.003)						
Test for subgroup differences: $Chi^2 = 10.45$, df = 2 (P = 0.005), $I^2 = 80.9\%$						

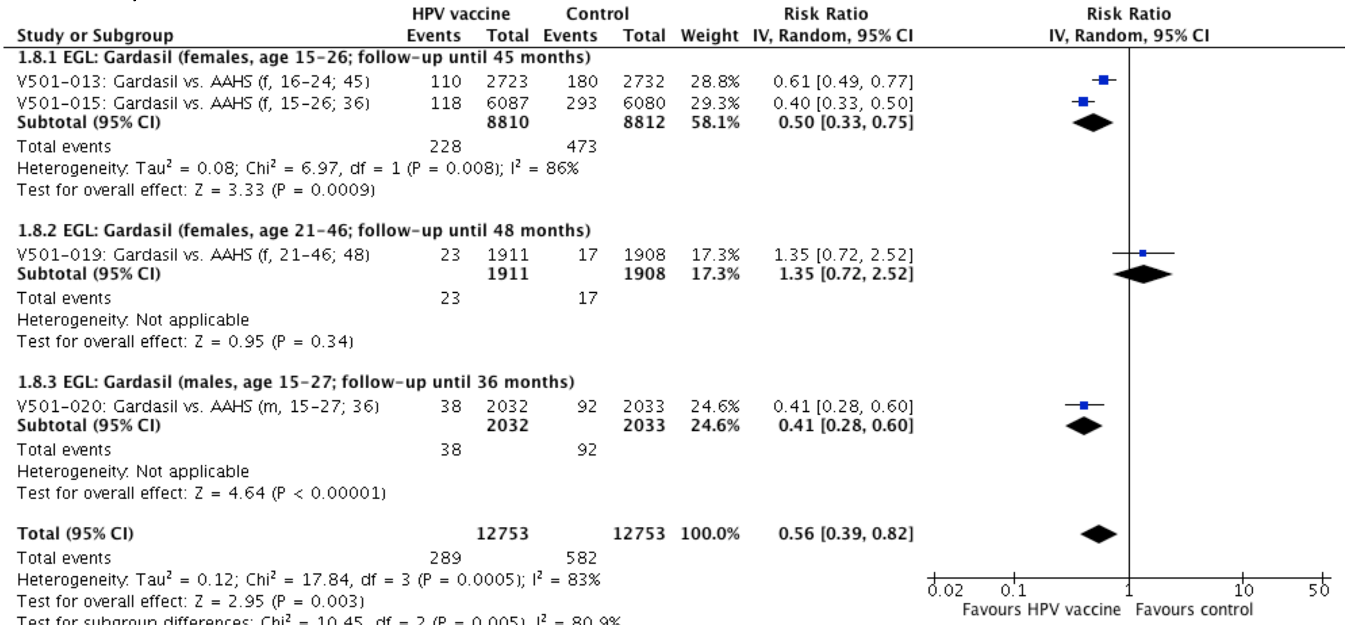

*1.8. Risk ratio for GlaxoSmithKline studies (i.e., HPV-0xx): not applicable; risk ratio for Merck Sharp & Dohme studies (i.e., V50x-xxx): **0.56 [0.39, 0.82]**.

2. Anal outcomes

2.1. Mortality from anal cancer irrespective of HPV type: intention to treat analysis
No cases/data/reports.

2.2. Incidence of anal cancer irrespective of HPV type: intention to treat analysis
No cases/data/reports.

2.3. Incidence of high grade anal intraepithelial neoplasia (AIN3) irrespective of HPV type: intention to treat analysis
No cases/data/reports.

2.4. Incidence of moderate grade anal intraepithelial neoplasia (AIN2) irrespective of HPV type: intention to treat analysis
No cases/data/reports.

2.5. Incidence of high grade anal intraepithelial neoplasia or worse ($AIN3^+$) irrespective of HPV type: intention to treat analysis
No cases/data/reports.

2.6. Incidence of moderate grade anal intraepithelial neoplasia or worse ($AIN2^+$) irrespective of HPV type: intention to treat analysis
No cases/data/reports.

3. Cervical outcomes

3.1. Mortality from cervical cancer irrespective of HPV type*: intention to treat analysis

Study or Subgroup	HPV vaccine Events	HPV vaccine Total	Control Events	Control Total	Weight	Risk Ratio IV, Random, 95% CI	Risk Ratio IV, Random, 95% CI
**3.1.1 Mortality from cervical cancer: Cervarix (females, age 24-72; follow-up until 36 months)**							
HPV-015: Cervarix vs. Al(OH)3 (f, 24-72; 36)	1	2882	0	2871	100.0%	2.99 [0.12, 73.33]	
**Subtotal (95% CI)**		**2882**		**2871**	**100.0%**	**2.99 [0.12, 73.33]**	
Total events	1		0				
Heterogeneity: Not applicable							
Test for overall effect: Z = 0.67 (P = 0.50)							
**Total (95% CI)**		**2882**		**2871**	**100.0%**	**2.99 [0.12, 73.33]**	
Total events	1		0				
Heterogeneity: Not applicable							
Test for overall effect: Z = 0.67 (P = 0.50)							
Test for subgroup differences: Not applicable							

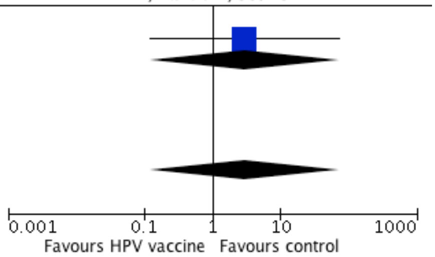

*3.1. Risk ratio for GlaxoSmithKline studies (i.e., HPV-0xx): 2.99 [0.12, 73.33]; risk ratio for Merck Sharp & Dohme studies (i.e., V50x-xxx): not applicable.

3.2. Incidence of cervical cancer irrespective of HPV type*: intention to treat analysis

Study or Subgroup	HPV vaccine Events	HPV vaccine Total	Control Events	Control Total	Weight	Risk Ratio IV, Random, 95% CI
**3.2.1 Cervical adenocarcinoma: Cervarix (females, age 24-72; follow-up until 36 months)**						
HPV-015: Cervarix vs. Al(OH)3 (f, 24-72; 36)	1	2882	0	2871	31.5%	2.99 [0.12, 73.33]
**Subtotal (95% CI)**		**2882**		**2871**	**31.5%**	**2.99 [0.12, 73.33]**
Total events	1		0			
Heterogeneity: Not applicable						
Test for overall effect: Z = 0.67 (P = 0.50)						
**3.2.2 Cervical squamous cell carcinoma: Cervarix (females, age 24-72; follow-up until 36 months)**						
HPV-015: Cervarix vs. Al(OH)3 (f, 24-72; 36)	2	2882	0	2871	34.3%	4.98 [0.24, 103.70]
**Subtotal (95% CI)**		**2882**		**2871**	**34.3%**	**4.98 [0.24, 103.70]**
Total events	2		0			
Heterogeneity: Not applicable						
Test for overall effect: Z = 1.04 (P = 0.30)						
**3.2.3 Cervical squamous cell carcinoma: Gardasil (females, age 21-46; follow-up until 48 months)**						
V501-019: Gardasil vs. AAHS (f, 21-46; 48)	0	1911	2	1908	34.3%	0.20 [0.01, 4.16]
**Subtotal (95% CI)**		**1911**		**1908**	**34.3%**	**0.20 [0.01, 4.16]**
Total events	0		2			
Heterogeneity: Not applicable						
Test for overall effect: Z = 1.04 (P = 0.30)						
**Total (95% CI)**		**7675**		**7650**	**100.0%**	**1.41 [0.19, 10.21]**
Total events	3		2			
Heterogeneity: $Tau^2 = 0.58$; $Chi^2 = 2.47$, df = 2 (P = 0.29); $I^2 = 19\%$						
Test for overall effect: Z = 0.34 (P = 0.73)						
Test for subgroup differences: $Chi^2 = 2.47$, df = 2 (P = 0.29), $I^2 = 19.0\%$						

Risk Ratio IV, Random, 95% CI

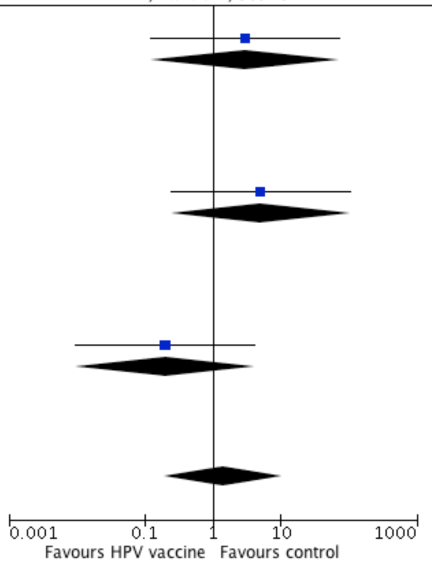

*3.2. Risk ratio for GlaxoSmithKline studies (i.e., HPV-0xx): 3.91 [0.43, 35.38]; risk ratio for Merck Sharp & Dohme studies (i.e., V50x-xxx): 0.20 [0.01, 4.16].

3.3. Incidence of cervical adenocarcinoma in situ (AIS) irrespective of HPV type*: intention to treat analysis

Study or Subgroup	HPV vaccine Events	HPV vaccine Total	Control Events	Control Total	Weight	Risk Ratio IV, Random, 95% CI
**3.3.1 AIS: Cervarix (females, age 15–25; follow-up until 48 months)**						
HPV-008: Cervarix vs. Havrix (f, 15-25; 48)	3	9319	13	9325	34.6%	0.23 [0.07, 0.81]
**Subtotal (95% CI)**		**9319**		**9325**	**34.6%**	**0.23 [0.07, 0.81]**
Total events	3		13			
Heterogeneity: Not applicable						
Test for overall effect: Z = 2.29 (P = 0.02)						
**3.3.2 AIS: Gardasil (females, age 15–26; follow-up until 45 months)**						
V501-013: Gardasil vs. AAHS (f, 16-24; 45)	1	2723	6	2732	12.2%	0.17 [0.02, 1.39]
V501-015: Gardasil vs. AAHS (f, 15-26; 36)	5	6087	10	6080	47.3%	0.50 [0.17, 1.46]
**Subtotal (95% CI)**		**8810**		**8812**	**59.5%**	**0.40 [0.15, 1.04]**
Total events	6		16			
Heterogeneity: Tau² = 0.00; Chi² = 0.82, df = 1 (P = 0.37); I² = 0%						
Test for overall effect: Z = 1.88 (P = 0.06)						
**3.3.3 AIS: Gardasil (females, age 21–46; follow-up until 48 months)**						
V501-019: Gardasil vs. AAHS (f, 21-46; 48)	0	1911	2	1908	5.9%	0.20 [0.01, 4.16]
**Subtotal (95% CI)**		**1911**		**1908**	**5.9%**	**0.20 [0.01, 4.16]**
Total events	0		2			
Heterogeneity: Not applicable						
Test for overall effect: Z = 1.04 (P = 0.30)						
**Total (95% CI)**		**20040**		**20045**	**100.0%**	**0.32 [0.15, 0.66]**
Total events	9		31			
Heterogeneity: Tau² = 0.00; Chi² = 1.37, df = 3 (P = 0.71); I² = 0%						
Test for overall effect: Z = 3.05 (P = 0.002)						
Test for subgroup differences: Chi² = 0.56, df = 2 (P = 0.76), I² = 0%						

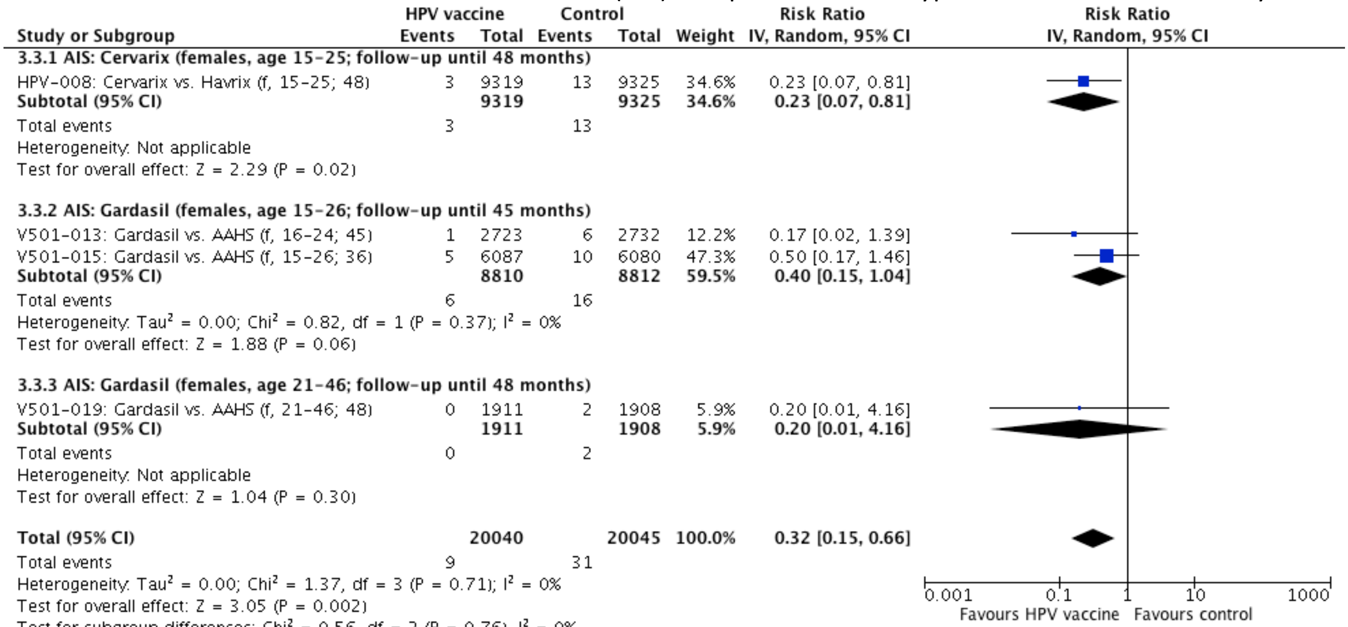

*3.3. Risk ratio for GlaxoSmithKline studies (i.e., HPV-0xx): **0.23 [0.07, 0.81]**; risk ratio for Merck Sharp & Dohme studies (i.e., V50x-xxx): **0.37 [0.15, 0.93]**.

3.4. Incidence of cervical intraepithelial neoplasia grade 3 (CIN3) irrespective of HPV type*: intention to treat analysis

Study or Subgroup	HPV vaccine Events	HPV vaccine Total	Control Events	Control Total	Weight	Risk Ratio IV, Random, 95% CI
**3.4.1 CIN3: Cervarix (females, age 15-25; follow-up until 48 months)**						
HPV-008: Cervarix vs. Havrix (f, 15-25; 48)	83	9319	145	9325	26.7%	0.57 [0.44, 0.75]
**Subtotal (95% CI)**		**9319**		**9325**	**26.7%**	**0.57 [0.44, 0.75]**
Total events	83		145			
Heterogeneity: Not applicable						
Test for overall effect: Z = 4.07 (P < 0.0001)						
**3.4.2 CIN3: Gardasil (females, age 15-26; follow-up until 45 months)**						
V501-013: Gardasil vs. AAHS (f, 16-24; 45)	84	2723	76	2732	25.3%	1.11 [0.82, 1.51]
V501-015: Gardasil vs. AAHS (f, 15-26; 36)	153	6087	208	6080	28.9%	0.73 [0.60, 0.90]
**Subtotal (95% CI)**		**8810**		**8812**	**54.1%**	**0.89 [0.59, 1.33]**
Total events	237		284			
Heterogeneity: $Tau^2 = 0.07$; $Chi^2 = 4.79$, df = 1 (P = 0.03); $I^2 = 79\%$						
Test for overall effect: Z = 0.58 (P = 0.56)						
**3.4.3 CIN3: Gardasil (females, age 21-46; follow-up until 48 months)**						
V501-019: Gardasil vs. AAHS (f, 21-46; 48)	38	1911	30	1908	19.2%	1.26 [0.79, 2.03]
**Subtotal (95% CI)**		**1911**		**1908**	**19.2%**	**1.26 [0.79, 2.03]**
Total events	38		30			
Heterogeneity: Not applicable						
Test for overall effect: Z = 0.97 (P = 0.33)						
**Total (95% CI)**		**20040**		**20045**	**100.0%**	**0.85 [0.61, 1.17]**
Total events	358		459			
Heterogeneity: $Tau^2 = 0.08$; $Chi^2 = 14.49$, df = 3 (P = 0.002); $I^2 = 79\%$						
Test for overall effect: Z = 1.01 (P = 0.31)						
Test for subgroup differences: $Chi^2 = 9.19$, df = 2 (P = 0.01), $I^2 = 78.2\%$						

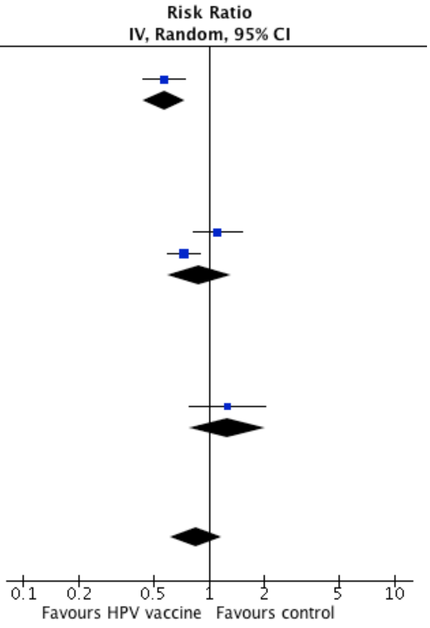

*3.4. Risk ratio for GlaxoSmithKline studies (i.e., HPV-0xx): **0.57 [0.44, 0.75]**; risk ratio for Merck Sharp & Dohme studies (i.e., V50x-xxx): 0.97 [0.69, 1.37].

3.5. Incidence of cervical intraepithelial neoplasia grade 2 (CIN2) irrespective of HPV type*: intention to treat analysis

	HPV vaccine		Control			Risk Ratio
Study or Subgroup	Events	Total	Events	Total	Weight	IV, Random, 95% CI
**3.5.1 CIN2: Cervarix (females, age 15-25; follow-up until 48 months)**						
HPV-008: Cervarix vs. Havrix (f, 15-25; 48)	201	9319	370	9325	28.2%	0.54 [0.46, 0.64]
**Subtotal (95% CI)**		**9319**		**9325**	**28.2%**	**0.54 [0.46, 0.64]**
Total events	201		370			
Heterogeneity: Not applicable						
Test for overall effect: Z = 7.06 (P < 0.00001)						
**3.5.2 CIN2: Gardasil (females, age 15-26; follow-up until 45 months)**						
V501-013: Gardasil vs. AAHS (f, 16-24; 45)	110	2723	125	2732	25.7%	0.88 [0.69, 1.13]
V501-015: Gardasil vs. AAHS (f, 15-26; 36)	185	6087	238	6080	27.6%	0.78 [0.64, 0.94]
**Subtotal (95% CI)**		**8810**		**8812**	**53.3%**	**0.81 [0.70, 0.95]**
Total events	295		363			
Heterogeneity: $Tau^2 = 0.00$; $Chi^2 = 0.64$, df = 1 (P = 0.42); $I^2 = 0\%$						
Test for overall effect: Z = 2.68 (P = 0.007)						
**3.5.3 CIN2: Gardasil (females, age 21-46; follow-up until 48 months)**						
V501-019: Gardasil vs. AAHS (f, 21-46; 48)	42	1911	30	1908	18.5%	1.40 [0.88, 2.22]
**Subtotal (95% CI)**		**1911**		**1908**	**18.5%**	**1.40 [0.88, 2.22]**
Total events	42		30			
Heterogeneity: Not applicable						
Test for overall effect: Z = 1.41 (P = 0.16)						
**Total (95% CI)**		**20040**		**20045**	**100.0%**	**0.81 [0.59, 1.11]**
Total events	538		763			
Heterogeneity: $Tau^2 = 0.09$; $Chi^2 = 21.50$, df = 3 (P < 0.0001); $I^2 = 86\%$						
Test for overall effect: Z = 1.31 (P = 0.19)						
Test for subgroup differences: $Chi^2 = 20.86$, df = 2 (P < 0.0001), $I^2 = 90.4\%$						

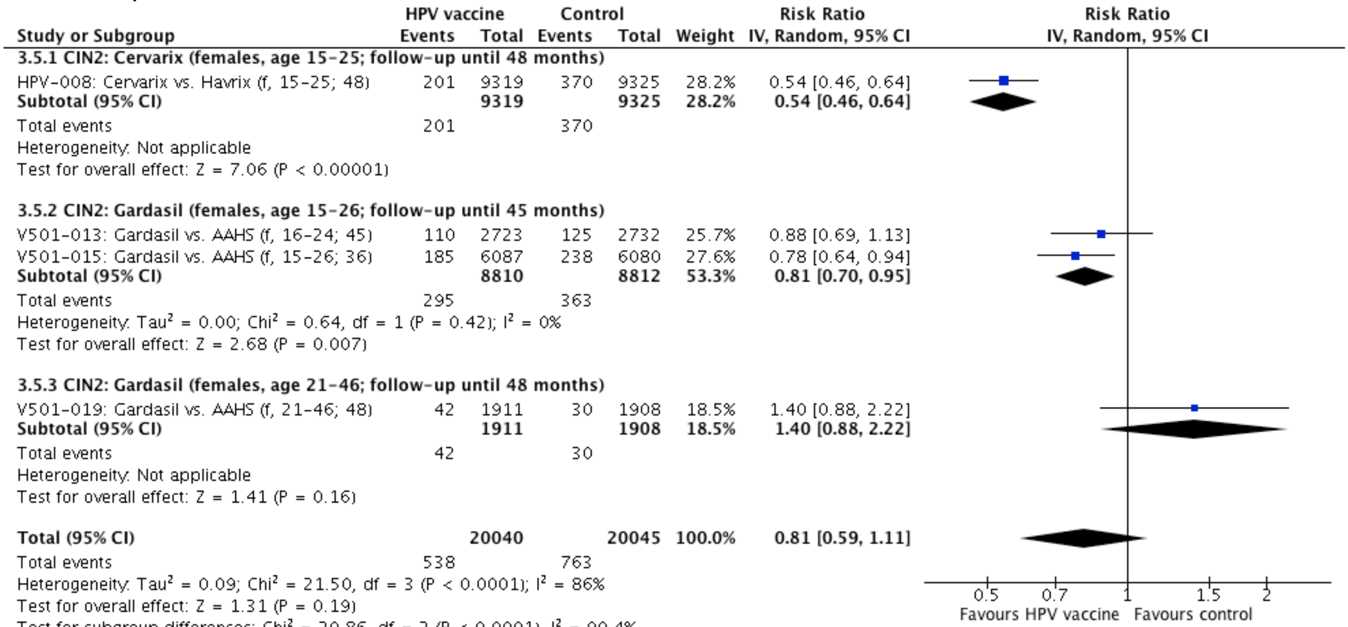

*3.5. Risk ratio for GlaxoSmithKline studies (i.e., HPV-0xx): **0.54 [0.46, 0.64]**; risk ratio for Merck Sharp & Dohme studies (i.e., V50x-xxx): 0.92 [0.70, 1.19].

3.6. Incidence of cervical intraepithelial neoplasia grade 3 or worse (CIN3^{+}) irrespective of HPV type*: intention to treat analysis

Study or Subgroup	HPV vaccine Events	HPV vaccine Total	Control Events	Control Total	Weight	Risk Ratio IV, Random, 95% CI
**3.6.1 CIN3+: Cervarix (females, age 15-25; follow-up until 48 months)**						
HPV-008: Cervarix vs. Havrix (f, 15-25; 48)	86	9319	158	9325	24.5%	0.54 [0.42, 0.71]
**Subtotal (95% CI)**		**9319**		**9325**	**24.5%**	**0.54 [0.42, 0.71]**
Total events	86		158			
Heterogeneity: Not applicable						
Test for overall effect: Z = 4.56 (P < 0.00001)						
**3.6.2 CIN3+: Cervarix (females, age 22-29; follow-up until 12 months)**						
HPV-063: Cervarix vs. Aimmugen (f, 22-29; 12)	9	464	14	463	8.7%	0.64 [0.28, 1.47]
**Subtotal (95% CI)**		**464**		**463**	**8.7%**	**0.64 [0.28, 1.47]**
Total events	9		14			
Heterogeneity: Not applicable						
Test for overall effect: Z = 1.05 (P = 0.29)						
**3.6.3 CIN3+: Gardasil (females, age 15-26; follow-up until 45 months)**						
V501-013: Gardasil vs. AAHS (f, 16-24; 45)	84	2723	78	2732	22.9%	1.08 [0.80, 1.46]
V501-015: Gardasil vs. AAHS (f, 15-26; 36)	155	6087	214	6080	26.6%	0.72 [0.59, 0.89]
**Subtotal (95% CI)**		**8810**		**8812**	**49.6%**	**0.87 [0.59, 1.29]**
Total events	239		292			
Heterogeneity: Tau² = 0.06; Chi² = 4.62, df = 1 (P = 0.03); I² = 78%						
Test for overall effect: Z = 0.70 (P = 0.48)						
**3.6.4 CIN3+: Gardasil (females, age 21-46; follow-up until 48 months)**						
V501-019: Gardasil vs. AAHS (f, 21-46; 48)	38	1911	34	1908	17.3%	1.12 [0.71, 1.76]
**Subtotal (95% CI)**		**1911**		**1908**	**17.3%**	**1.12 [0.71, 1.76]**
Total events	38		34			
Heterogeneity: Not applicable						
Test for overall effect: Z = 0.47 (P = 0.64)						
**Total (95% CI)**		**20504**		**20508**	**100.0%**	**0.79 [0.59, 1.05]**
Total events	372		498			
Heterogeneity: Tau² = 0.07; Chi² = 14.47, df = 4 (P = 0.006); I² = 72%						
Test for overall effect: Z = 1.62 (P = 0.11)						
Test for subgroup differences: Chi² = 8.74, df = 3 (P = 0.03), I² = 65.7%						

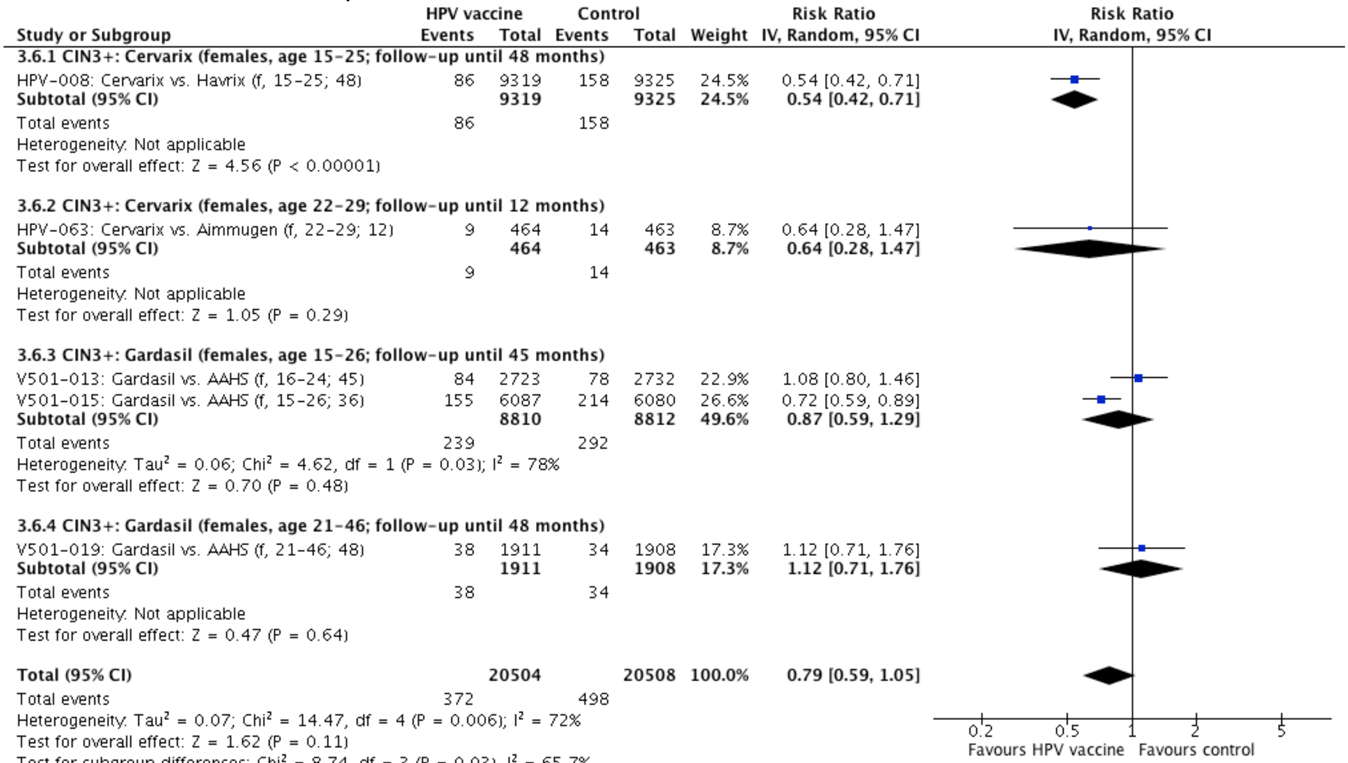

*3.6. Risk ratio for GlaxoSmithKline studies (i.e., HPV-0xx): **0.55 [0.43, 0.71]**; risk ratio for Merck Sharp & Dohme studies (i.e., V50x-xxx): 0.92 [0.67, 1.26].

3.7. Incidence of cervical intraepithelial neoplasia grade 2 or worse ($CIN2^+$) irrespective of HPV type*: intention to treat analysis

Study or Subgroup	HPV vaccine Events	HPV vaccine Total	Control Events	Control Total	Weight	Risk Ratio IV, Random, 95% CI
**3.7.1 CIN2+: Cervarix (females, age 15-25; follow-up until 48 months)**						
HPV-008: Cervarix vs. Havrix (f, 15-25; 48)	287	9319	428	9325	22.0%	0.67 [0.58, 0.78]
**Subtotal (95% CI)**		**9319**		**9325**	**22.0%**	**0.67 [0.58, 0.78]**
Total events	287		428			
Heterogeneity: Not applicable						
Test for overall effect: Z = 5.33 (P < 0.00001)						
**3.7.2 CIN2+: Cervarix (females, age 22-29; follow-up until 12 months)**						
HPV-063: Cervarix vs. Aimmugen (f, 22-29; 12)	19	464	41	463	8.1%	0.46 [0.27, 0.78]
**Subtotal (95% CI)**		**464**		**463**	**8.1%**	**0.46 [0.27, 0.78]**
Total events	19		41			
Heterogeneity: Not applicable						
Test for overall effect: Z = 2.86 (P = 0.004)						
**3.7.3 CIN2+: Cervarix (females, age 24-72; follow-up until 36 months)**						
HPV-015: Cervarix vs. Al(OH)3 (f, 24-72; 36)	103	2882	108	2871	16.6%	0.95 [0.73, 1.24]
**Subtotal (95% CI)**		**2882**		**2871**	**16.6%**	**0.95 [0.73, 1.24]**
Total events	103		108			
Heterogeneity: Not applicable						
Test for overall effect: Z = 0.38 (P = 0.70)						
**3.7.4 CIN2+: Gardasil (females, age 15-26; follow-up until 45 months)**						
V501-013: Gardasil vs. AAHS (f, 16-24; 45)	152	2723	166	2732	19.0%	0.92 [0.74, 1.14]
V501-015: Gardasil vs. AAHS (f, 15-26; 36)	269	6087	350	6080	21.7%	0.77 [0.66, 0.90]
**Subtotal (95% CI)**		**8810**		**8812**	**40.7%**	**0.83 [0.70, 0.98]**
Total events	421		516			
Heterogeneity: $Tau^2 = 0.01$; $Chi^2 = 1.78$, df = 1 (P = 0.18); $I^2 = 44\%$						
Test for overall effect: Z = 2.15 (P = 0.03)						
**3.7.5 CIN2+: Gardasil (females, age 21-46; follow-up until 48 months)**						
V501-019: Gardasil vs. AAHS (f, 21-46; 48)	62	1911	51	1908	12.6%	1.21 [0.84, 1.75]
**Subtotal (95% CI)**		**1911**		**1908**	**12.6%**	**1.21 [0.84, 1.75]**
Total events	62		51			
Heterogeneity: Not applicable						
Test for overall effect: Z = 1.04 (P = 0.30)						
**Total (95% CI)**		**23386**		**23379**	**100.0%**	**0.81 [0.68, 0.97]**
Total events	892		1144			
Heterogeneity: $Tau^2 = 0.03$; $Chi^2 = 17.86$, df = 5 (P = 0.003); $I^2 = 72\%$						
Test for overall effect: Z = 2.25 (P = 0.02)						
Test for subgroup differences: $Chi^2 = 15.98$, df = 4 (P = 0.003), $I^2 = 75.0\%$						

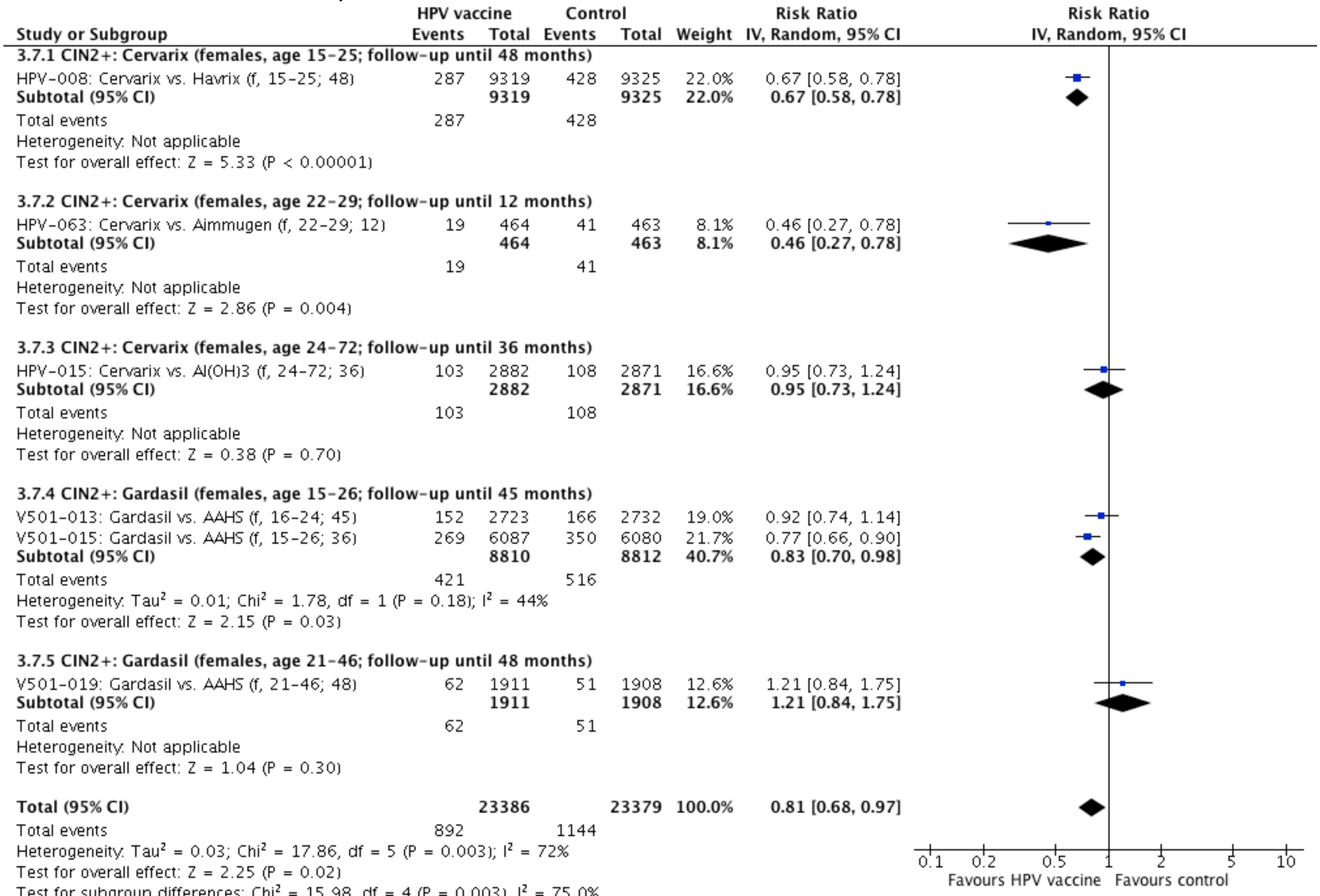

*3.7. Risk ratio for GlaxoSmithKline studies (i.e., HPV-0xx): **0.70 [0.51, 0.97]**; risk ratio for Merck Sharp & Dohme studies (i.e., V50x-xxx): 0.90 [0.72, 1.13].

4. Oropharyngeal outcomes

4.1. Mortality from oropharyngeal cancer irrespective of HPV type*: intention to treat analysis

Study or Subgroup	HPV vaccine Events	HPV vaccine Total	Control Events	Control Total	Weight	Risk Ratio IV, Random, 95% CI
**4.1.1 Mortality from oropharyngeal cancer: Cervarix (females, age 24-72; follow-up until 36 months)**						
HPV-015: Cervarix vs. Al(OH)3 (f, 24-72; 36)	0	2882	1	2871	50.0%	0.33 [0.01, 8.15]
**Subtotal (95% CI)**		**2882**		**2871**	**50.0%**	**0.33 [0.01, 8.15]**
Total events	0		1			
Heterogeneity: Not applicable						
Test for overall effect: Z = 0.68 (P = 0.50)						
**4.1.2 Mortality from oropharyngeal cancer: Gardasil (females, age 21-46; follow-up until 48 months)**						
V501-019: Gardasil vs. AAHS (f, 21-46; 48)	1	1911	0	1908	50.0%	3.00 [0.12, 73.48]
**Subtotal (95% CI)**		**1911**		**1908**	**50.0%**	**3.00 [0.12, 73.48]**
Total events	1		0			
Heterogeneity: Not applicable						
Test for overall effect: Z = 0.67 (P = 0.50)						
**Total (95% CI)**		**4793**		**4779**	**100.0%**	**1.00 [0.10, 9.58]**
Total events	1		1			
Heterogeneity: Tau² = 0.00; Chi² = 0.91, df = 1 (P = 0.34); I² = 0%						
Test for overall effect: Z = 0.00 (P = 1.00)						
Test for subgroup differences: Chi² = 0.91, df = 1 (P = 0.34), I² = 0%						

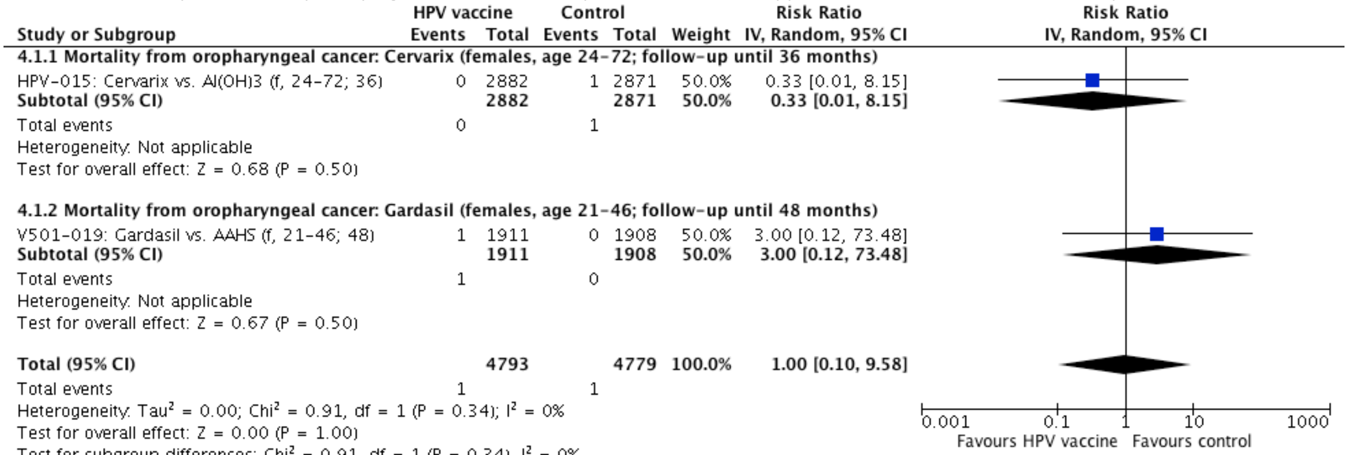

*4.1. Risk ratio for GlaxoSmithKline studies (i.e., HPV-0xx): 0.33 [0.01, 8.15]; risk ratio for Merck Sharp & Dohme studies (i.e., V50x-xxx): not applicable.

4.2. Incidence of oropharyngeal cancer irrespective of HPV type*: intention to treat analysis

Study or Subgroup	HPV vaccine Events	HPV vaccine Total	Control Events	Control Total	Weight	Risk Ratio IV, Random, 95% CI
**4.2.1 Incidende of oropharyngeal cancer: Cervarix (females, age 24–72; follow-up until 36 months)**						
HPV-015: Cervarix vs. Al(OH)3 (f, 24-72; 36)	0	2882	1	2871	50.0%	0.33 [0.01, 8.15]
**Subtotal (95% CI)**		**2882**		**2871**	**50.0%**	**0.33 [0.01, 8.15]**
Total events	0		1			
Heterogeneity: Not applicable						
Test for overall effect: Z = 0.68 (P = 0.50)						
**4.2.2 Incidende of oropharyngeal cancer: Gardasil (females, age 21–46; follow-up until 48 months)**						
V501-019: Gardasil vs. AAHS (f, 21-46; 48)	1	1911	0	1908	50.0%	3.00 [0.12, 73.48]
**Subtotal (95% CI)**		**1911**		**1908**	**50.0%**	**3.00 [0.12, 73.48]**
Total events	1		0			
Heterogeneity: Not applicable						
Test for overall effect: Z = 0.67 (P = 0.50)						
**Total (95% CI)**		**4793**		**4779**	**100.0%**	**1.00 [0.10, 9.58]**
Total events	1		1			
Heterogeneity: Tau² = 0.00; Chi² = 0.91, df = 1 (P = 0.34); I² = 0%						
Test for overall effect: Z = 0.00 (P = 1.00)						
Test for subgroup differences: Chi² = 0.91, df = 1 (P = 0.34), I² = 0%						

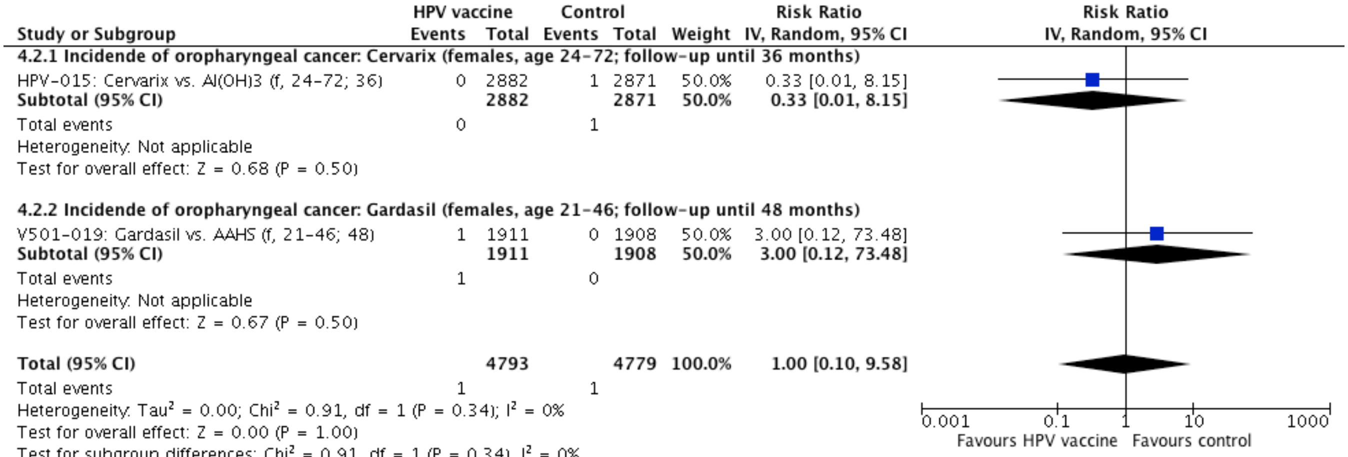

*4.2. Risk ratio for GlaxoSmithKline studies (i.e., HPV-0xx): 0.33 [0.01, 8.15]; risk ratio for Merck Sharp & Dohme studies (i.e., V50x-xxx): 3.00 [0.12, 73.48].

4.3. Incidence of oropharyngeal carcinoma in situ irrespective of HPV type*: intention to treat analysis

Not applicable.

5. Penile outcomes

5.1. Mortality from penile cancer irrespective of HPV type: intention to treat analysis
No cases/data/reports.

5.2. Incidence of penile cancer irrespective of HPV type: intention to treat analysis
No cases/data/reports.

5.3. Incidence of penile intraepithelial neoplasia grade 3 (PIN3) irrespective of HPV type: intention to treat analysis
No cases/data/reports.

5.4. Incidence of penile intraepithelial neoplasia grade 2 (PIN2) irrespective of HPV type: intention to treat analysis
No cases/data/reports.

5.5. Incidence of penile intraepithelial neoplasia grade 3 or worse ($PIN3^{+}$) irrespective of HPV type: intention to treat analysis
No cases/data/reports.

5.6. Incidence of penile intraepithelial neoplasia grade 2 or worse (PIN2+) irrespective of HPV type*: intention to treat analysis

Study or Subgroup	HPV vaccine Events	HPV vaccine Total	Control Events	Control Total	Weight	Risk Ratio IV, Random, 95% CI
V501-020: Gardasil vs. AAHS (m, 15-27; 36)	3	2032	3	2033	100.0%	1.00 [0.20, 4.95]
**Total (95% CI)**		**2032**		**2033**	**100.0%**	**1.00 [0.20, 4.95]**
Total events	3		3			
Heterogeneity: Not applicable						
Test for overall effect: Z = 0.00 (P = 1.00)						

Risk Ratio IV, Random, 95% CI

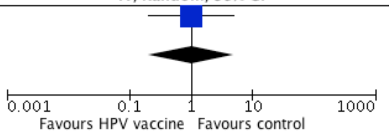

*5.6. Risk ratio for GlaxoSmithKline studies (i.e., HPV-0xx): not applicable; risk ratio for Merck Sharp & Dohme studies (i.e., V50x-xxx): 1.00 [0.20, 4.95].

6. Vaginal outcomes

6.1. Mortality from vaginal cancer irrespective of HPV type: intention to treat analysis
No cases/data/reports.

6.2. Incidence of vaginal cancer irrespective of HPV type*: intention to treat analysis

Study or Subgroup	HPV vaccine Events	HPV vaccine Total	Control Events	Control Total	Weight	Risk Ratio IV, Random, 95% CI
HPV-015: Cervarix vs. Al(OH)3 (f, 24-72; 36)	1	2882	0	2871	100.0%	2.99 [0.12, 73.33]
**Total (95% CI)**		**2882**		**2871**	**100.0%**	**2.99 [0.12, 73.33]**
Total events	1		0			
Heterogeneity: Not applicable						
Test for overall effect: Z = 0.67 (P = 0.50)						

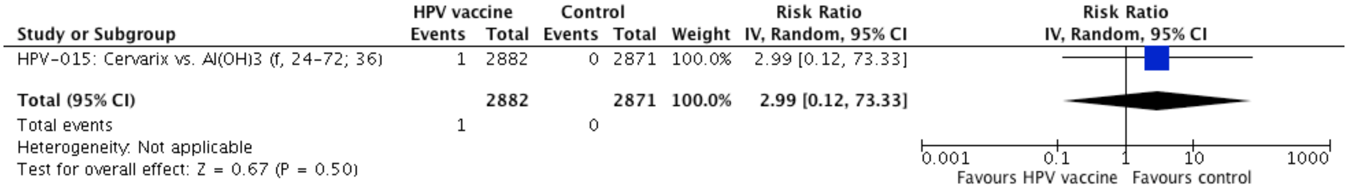

*6.2. Risk ratio for GlaxoSmithKline studies (i.e., HPV-0xx): 2.99 [0.12, 73.33]; risk ratio for Merck Sharp & Dohme studies (i.e., V50x-xxx): not applicable.

6.3. Incidence of vaginal intraepithelial neoplasia grade 3 (VaIN3) irrespective of HPV type: intention to treat analysis
No cases/data/reports.

6.4. Incidence of vaginal intraepithelial neoplasia grade 2 (VaIN2) irrespective of HPV type: intention to treat analysis
No cases/data/reports.

6.5. Incidence of vaginal intraepithelial neoplasia grade 3 or worse (VaIN3$^+$) irrespective of HPV type: intention to treat analysis
No cases/data/reports.

6.6. Incidence of vaginal intraepithelial neoplasia grade 2 or worse (VaIN2+) irrespective of HPV type*: intention to treat analysis

Study or Subgroup	HPV vaccine Events	HPV vaccine Total	Control Events	Control Total	Weight	Risk Ratio IV, Random, 95% CI
**6.6.1 VaIN2+: Gardasil (females, age 15-26; follow-up until 45 months)**						
V501-013: Gardasil vs. AAHS (f, 16-24; 45)	8	2723	17	2732	53.1%	0.47 [0.20, 1.09]
V501-015: Gardasil vs. AAHS (f, 15-26; 36)	6	6087	9	6080	37.9%	0.67 [0.24, 1.87]
**Subtotal (95% CI)**		**8810**		**8812**	**91.0%**	**0.54 [0.28, 1.04]**
Total events	14		26			
Heterogeneity: $Tau^2 = 0.00$; $Chi^2 = 0.26$, df = 1 (P = 0.61); $I^2 = 0\%$						
Test for overall effect: Z = 1.85 (P = 0.06)						
**6.6.2 VaIN2+: Gardasil (females, age 21-46; follow-up until 48 months)**						
V501-019: Gardasil vs. AAHS (f, 21-46; 48)	3	1911	1	1908	9.0%	3.00 [0.31, 28.77]
**Subtotal (95% CI)**		**1911**		**1908**	**9.0%**	**3.00 [0.31, 28.77]**
Total events	3		1			
Heterogeneity: Not applicable						
Test for overall effect: Z = 0.95 (P = 0.34)						
**Total (95% CI)**		**10721**		**10720**	**100.0%**	**0.64 [0.32, 1.27]**
Total events	17		27			
Heterogeneity: $Tau^2 = 0.05$; $Chi^2 = 2.29$, df = 2 (P = 0.32); $I^2 = 12\%$						
Test for overall effect: Z = 1.28 (P = 0.20)						
Test for subgroup differences: $Chi^2 = 2.03$, df = 1 (P = 0.15), $I^2 = 50.7\%$						

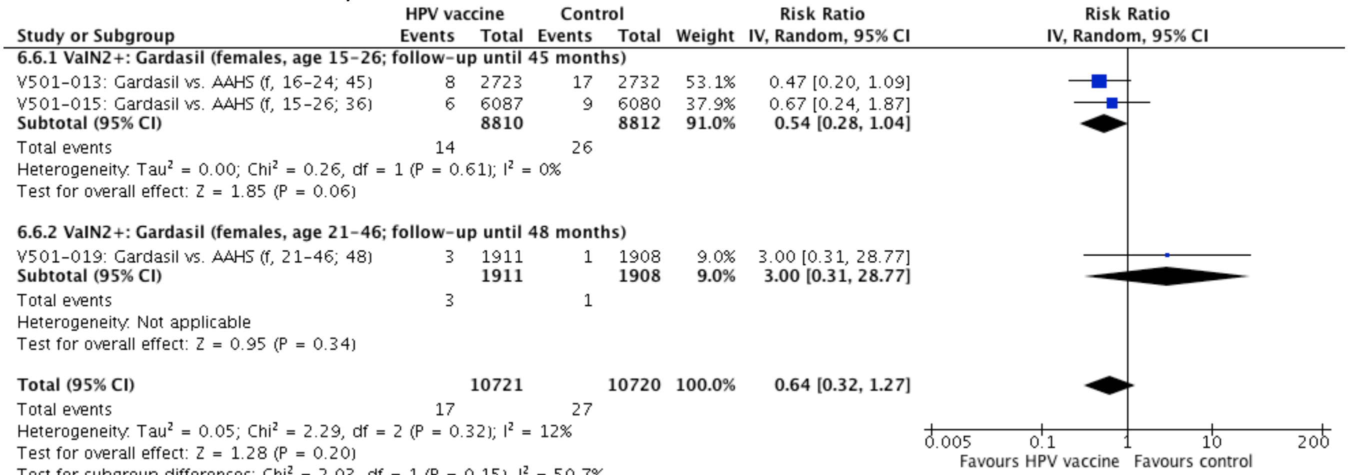

*6.6. Risk ratio for GlaxoSmithKline studies (i.e., HPV-0xx): not applicable; risk ratio for Merck Sharp & Dohme studies (i.e., V50x-xxx): 0.64 [0.32, 1.27].

7. Vulvar outcomes

7.1. Mortality from vulvar cancer irrespective of HPV type: intention to treat analysis
No cases/data/reports.

7.2. Incidence of vulvar cancer irrespective of HPV type*: intention to treat analysis

Study or Subgroup	HPV vaccine Events	HPV vaccine Total	Control Events	Control Total	Weight	Risk Ratio IV, Random, 95% CI
**7.1.1 Incidence of vulvar cancer: Cervarix (females, age 15-25; follow-up until 48 months)**						
HPV-008: Cervarix vs. Havrix (f, 15-25; 48)	1	9319	0	9325	50.0%	3.00 [0.12, 73.68]
**Subtotal (95% CI)**		**9319**		**9325**	**50.0%**	**3.00 [0.12, 73.68]**
Total events	1		0			
Heterogeneity: Not applicable						
Test for overall effect: Z = 0.67 (P = 0.50)						
**7.1.2 Incidence of vulvar cancer: Gardasil (females, age 16-24; follow-up until 45 months)**						
V501-013: Gardasil vs. AAHS (f, 16-24; 45)	1	2723	0	2732	50.0%	3.01 [0.12, 73.85]
**Subtotal (95% CI)**		**2723**		**2732**	**50.0%**	**3.01 [0.12, 73.85]**
Total events	1		0			
Heterogeneity: Not applicable						
Test for overall effect: Z = 0.67 (P = 0.50)						
**Total (95% CI)**		**12042**		**12057**	**100.0%**	**3.01 [0.31, 28.89]**
Total events	2		0			
Heterogeneity: Tau² = 0.00; Chi² = 0.00, df = 1 (P = 1.00); I² = 0%						
Test for overall effect: Z = 0.95 (P = 0.34)						
Test for subgroup differences: Chi² = 0.00, df = 1 (P = 1.00), I² = 0%						

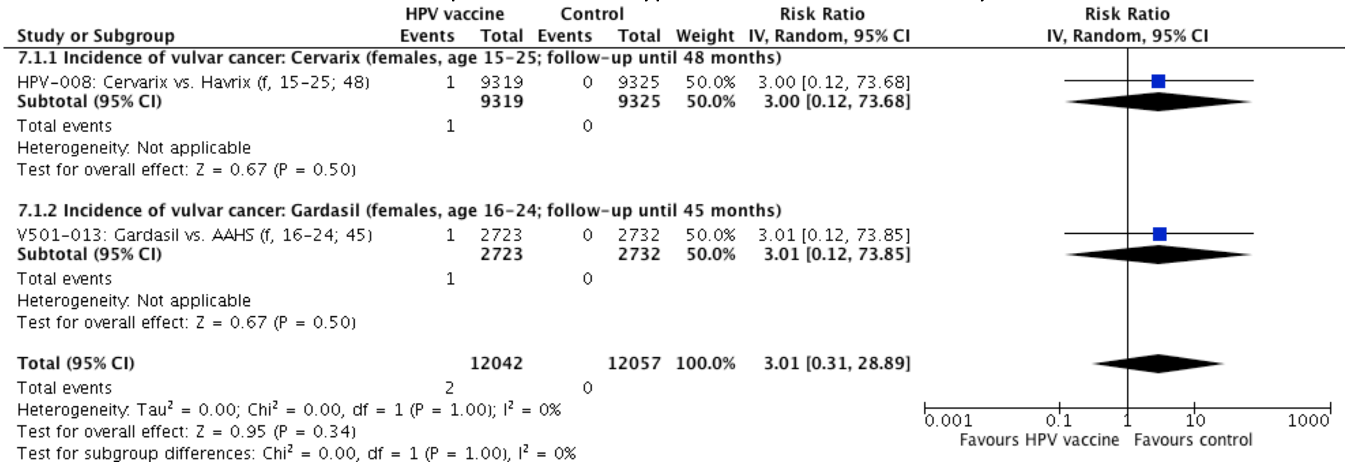

*7.2. Risk ratio for GlaxoSmithKline studies (i.e., HPV-0xx): 3.00 [0.12, 73.68]; risk ratio for Merck Sharp & Dohme studies (i.e., V50x-xxx): 3.00 [0.12, 73.61].

7.3. Incidence of VIN3 irrespective of HPV type: intention to treat analysis
No cases/data/reports.

7.4. Incidence of VIN2 irrespective of HPV type: intention to treat analysis
No cases/data/reports.

7.5. Incidence of VIN3$^+$ irrespective of HPV type: intention to treat analysis
No cases/data/reports.

7.6. Incidence of VIN2$^+$ irrespective of HPV type*: intention to treat analysis

Study or Subgroup	HPV vaccine Events	HPV vaccine Total	Control Events	Control Total	Weight	Risk Ratio IV, Random, 95% CI	Risk Ratio IV, Random, 95% CI
V501-013: Gardasil vs. AAHS (f, 16-24; 45)	12	2723	15	2732	53.0%	0.80 [0.38, 1.71]	
V501-015: Gardasil vs. AAHS (f, 15-26; 36)	6	6087	21	6080	47.0%	0.29 [0.12, 0.71]	
**Total (95% CI)**		**8810**		**8812**	**100.0%**	**0.49 [0.18, 1.36]**	
Total events	18		36				

Heterogeneity: Tau^2 = 0.35; Chi^2 = 2.94, df = 1 (P = 0.09); I^2 = 66%
Test for overall effect: Z = 1.37 (P = 0.17)

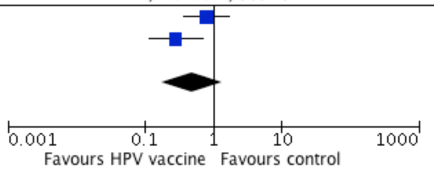

*7.6. Risk ratio for GlaxoSmithKline studies (i.e., HPV-0xx): not applicable; risk ratio for Merck Sharp & Dohme studies (i.e., V50x-xxx): 0.49 [0.18, 1.35].

8. Referral procedures

8.1. Number of participants with ‘any’ referral procedures performed (endoscopy, biopsy, surgical treatment and non-surgical treatment) due to HPV-related diseases irrespective of HPV type*: intention to treat analysis

Study or Subgroup	HPV vaccine Events	HPV vaccine Total	Control Events	Control Total	Weight	Risk Ratio IV, Random, 95% CI
**8.1.1 Referral procedures: Gardasil (females, age 15-26; follow-up until 45 months)**						
V501-013: Gardasil vs. AAHS (f, 16-24; 45)	385	2723	458	2732	17.7%	0.84 [0.74, 0.96]
V501-015: Gardasil vs. AAHS (f, 15-26; 36)	1556	6087	1806	6080	82.3%	0.86 [0.81, 0.91]
**Subtotal (95% CI)**		**8810**		**8812**	**100.0%**	**0.86 [0.81, 0.90]**
Total events	1941		2264			
Heterogeneity: $Tau^2 = 0.00$; $Chi^2 = 0.08$, df = 1 (P = 0.77); $I^2 = 0\%$						
Test for overall effect: Z = 5.75 (P < 0.00001)						
**Total (95% CI)**		**8810**		**8812**	**100.0%**	**0.86 [0.81, 0.90]**
Total events	1941		2264			
Heterogeneity: $Tau^2 = 0.00$; $Chi^2 = 0.08$, df = 1 (P = 0.77); $I^2 = 0\%$						
Test for overall effect: Z = 5.75 (P < 0.00001)						
Test for subgroup differences: Not applicable						

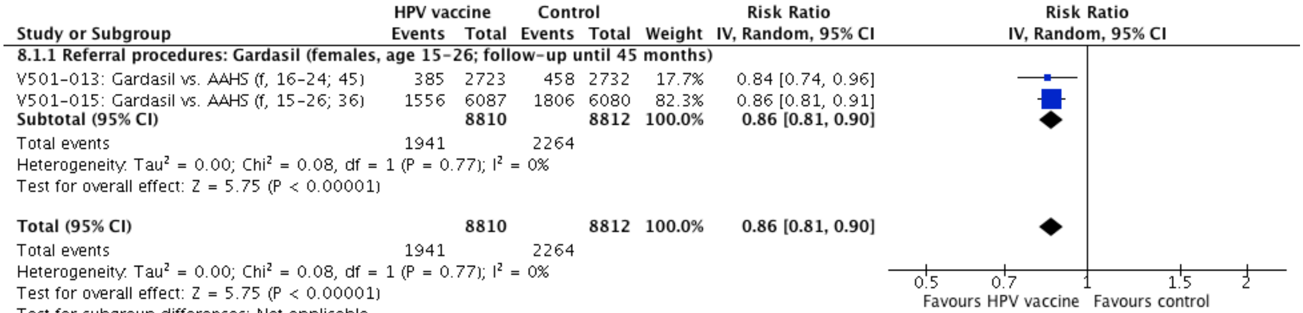

*8.1. Risk ratio for GlaxoSmithKline studies (i.e., HPV-0xx): not applicable; risk ratio for Merck Sharp & Dohme studies (i.e., V50x-xxx): **0.86 [0.81, 0.90]**.

8.2. Number of participants with endoscopies performed (anoscopy, colposcopy and rectoscopy) due to HPV-related diseases irrespective of HPV type*: intention to treat analysis

Study or Subgroup	HPV vaccine Events	HPV vaccine Total	Control Events	Control Total	Weight	Risk Ratio IV, Random, 95% CI
**8.2.1 Colposcopy: Cervarix (females, age 15–25; follow-up until 48 months)**						
HPV-008: Cervarix vs. Havrix (f, 15-25; 48)	1590	9319	1864	9325	35.8%	0.85 [0.80, 0.91]
**Subtotal (95% CI)**		**9319**		**9325**	**35.8%**	**0.85 [0.80, 0.91]**
Total events	1590		1864			
Heterogeneity: Not applicable						
Test for overall effect: Z = 5.14 (P < 0.00001)						
**8.2.2 Colposcopy: Cervarix (females, age 24–72; follow-up until 36 months)**						
HPV-015: Cervarix vs. Al(OH)3 (f, 24-72; 36)	506	2882	560	2871	11.1%	0.90 [0.81, 1.00]
**Subtotal (95% CI)**		**2882**		**2871**	**11.1%**	**0.90 [0.81, 1.00]**
Total events	506		560			
Heterogeneity: Not applicable						
Test for overall effect: Z = 1.90 (P = 0.06)						
**8.2.3 Colposcopy: Gardasil (females, age 15–26; follow-up until 45 months)**						
V501-013: Gardasil vs. AAHS (f, 16-24; 45)	376	2723	444	2732	8.1%	0.85 [0.75, 0.96]
V501-015: Gardasil vs. AAHS (f, 15-26; 36)	1479	6087	1675	6080	36.0%	0.88 [0.83, 0.94]
**Subtotal (95% CI)**		**8810**		**8812**	**44.2%**	**0.88 [0.83, 0.92]**
Total events	1855		2119			
Heterogeneity: $Tau^2 = 0.00$; $Chi^2 = 0.27$, df = 1 (P = 0.60); $I^2 = 0\%$						
Test for overall effect: Z = 4.77 (P < 0.00001)						
**8.2.4 Colposcopy: Gardasil (females, age 21–46; follow-up until 48 months)**						
V501-019: Gardasil vs. AAHS (f, 21-46; 48)	403	1911	422	1908	8.9%	0.95 [0.84, 1.08]
**Subtotal (95% CI)**		**1911**		**1908**	**8.9%**	**0.95 [0.84, 1.08]**
Total events	403		422			
Heterogeneity: Not applicable						
Test for overall effect: Z = 0.77 (P = 0.44)						
**Total (95% CI)**		**22922**		**22916**	**100.0%**	**0.88 [0.85, 0.91]**
Total events	4354		4965			
Heterogeneity: $Tau^2 = 0.00$; $Chi^2 = 3.10$, df = 4 (P = 0.54); $I^2 = 0\%$						
Test for overall effect: Z = 7.11 (P < 0.00001)						
Test for subgroup differences: $Chi^2 = 2.83$, df = 3 (P = 0.42), $I^2 = 0\%$						

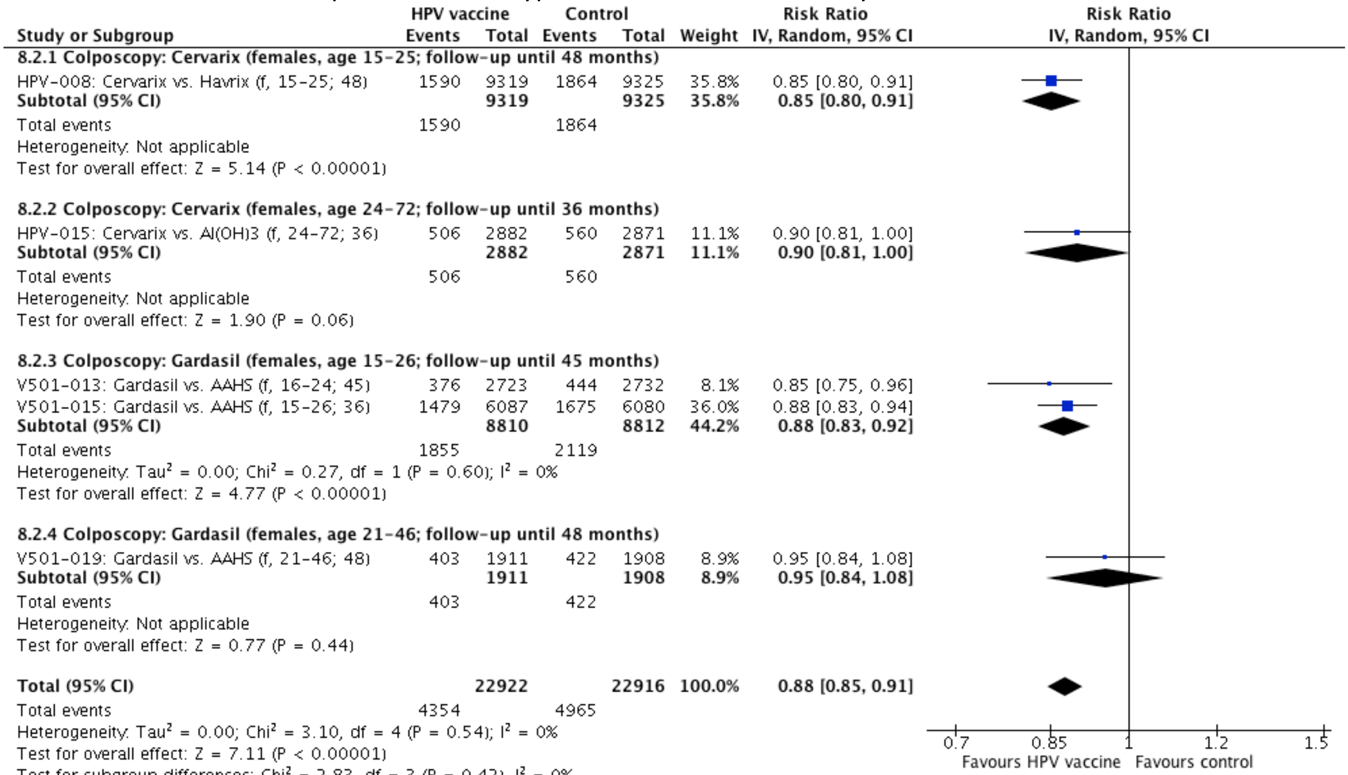

*8.2. Risk ratio for GlaxoSmithKline studies (i.e., HPV-0xx): **0.86 [0.82, 0.91]**; risk ratio for Merck Sharp & Dohme studies (i.e., V50x-xxx): **0.89 [0.85, 0.93]**. There were no reports of anoscopy or rectoscopy irrespective of HPV type.

8.3. Number of participants with biopsies performed due to HPV-related diseases irrespective of HPV type*: intention to treat analysis

Study or Subgroup	HPV vaccine Events	HPV vaccine Total	Control Events	Control Total	Weight	Risk Ratio IV, Random, 95% CI
**8.3.1 Cervical biopsy: Gardasil (females, age 15-26; follow-up until 45 months)**						
V501-013: Gardasil vs. AAHS (f, 16-24; 45)	297	2723	364	2732	15.9%	0.82 [0.71, 0.95]
V501-015: Gardasil vs. AAHS (f, 15-26; 36)	1344	6087	1522	6080	17.4%	0.88 [0.83, 0.94]
**Subtotal (95% CI)**		**8810**		**8812**	**33.3%**	**0.87 [0.82, 0.92]**
Total events	1641		1886			
Heterogeneity: Tau² = 0.00; Chi² = 0.86, df = 1 (P = 0.35); I² = 0%						
Test for overall effect: Z = 4.61 (P < 0.00001)						
**8.3.2 Cervical biopsy: Gardasil (females, age 21-46; follow-up until 48 months)**						
V501-019: Gardasil vs. AAHS (f, 21-46; 48)	365	1911	381	1908	16.2%	0.96 [0.84, 1.09]
**Subtotal (95% CI)**		**1911**		**1908**	**16.2%**	**0.96 [0.84, 1.09]**
Total events	365		381			
Heterogeneity: Not applicable						
Test for overall effect: Z = 0.68 (P = 0.50)						
**8.3.3 External genital lesion (EGL) biopsy: Gardasil (females, age 15-26; follow-up until 45 months)**						
V501-013: Gardasil vs. AAHS (f, 16-24; 45)	72	2723	128	2732	12.0%	0.56 [0.42, 0.75]
V501-015: Gardasil vs. AAHS (f, 15-26; 36)	228	6087	412	6080	15.5%	0.55 [0.47, 0.65]
**Subtotal (95% CI)**		**8810**		**8812**	**27.6%**	**0.56 [0.48, 0.64]**
Total events	300		540			
Heterogeneity: Tau² = 0.00; Chi² = 0.02, df = 1 (P = 0.90); I² = 0%						
Test for overall effect: Z = 8.35 (P < 0.00001)						
**8.3.4 External genital lesion (EGL) biopsy: Gardasil (females, age 21-46; follow-up until 48 months)**						
V501-019: Gardasil vs. AAHS (f, 21-46; 48)	56	1911	57	1908	10.0%	0.98 [0.68, 1.41]
**Subtotal (95% CI)**		**1911**		**1908**	**10.0%**	**0.98 [0.68, 1.41]**
Total events	56		57			
Heterogeneity: Not applicable						
Test for overall effect: Z = 0.10 (P = 0.92)						
**8.3.5 External genital lesion (EGL) biopsy: Gardasil (males, age 15-27; follow-up until 36 months)**						
V501-020: Gardasil vs. AAHS (m, 15-27; 36)	87	2032	157	2033	12.9%	0.55 [0.43, 0.72]
**Subtotal (95% CI)**		**2032**		**2033**	**12.9%**	**0.55 [0.43, 0.72]**
Total events	87		157			
Heterogeneity: Not applicable						
Test for overall effect: Z = 4.54 (P < 0.00001)						
**Total (95% CI)**		**23474**		**23473**	**100.0%**	**0.74 [0.62, 0.88]**
Total events	2449		3021			
Heterogeneity: Tau² = 0.04; Chi² = 51.08, df = 6 (P < 0.00001); I² = 88%						
Test for overall effect: Z = 3.40 (P = 0.0007)						
Test for subgroup differences: Chi² = 50.20, df = 4 (P < 0.00001), I² = 92.0%						

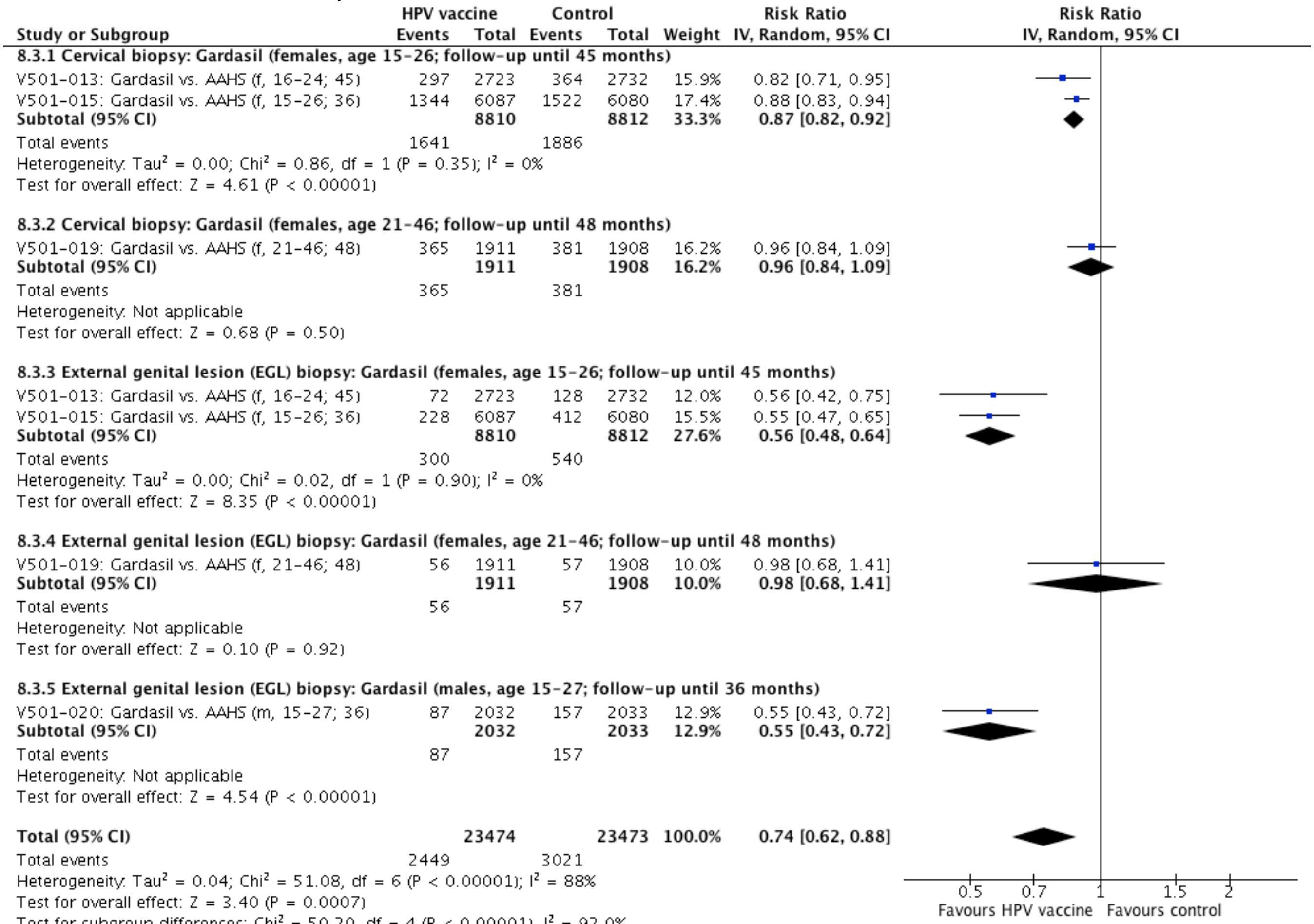

*8.3. Risk ratio for GlaxoSmithKline studies (i.e., HPV-0xx): not applicable; risk ratio for Merck Sharp & Dohme studies (i.e., V50x-xxx): **0.74 [0.62, 0.88]**. Only cervical biopsy: 2,006 in the HPV vaccine group vs. 2,267 in the control group, risk ratio **0.89 [0.83, 0.95]**; only EGL biopsy: 443 vs. 754, risk ratio **0.62 [0.50, 0.76]**.

8.4. Number of treatment procedures (both surgical and non-surgical treatment) due to HPV-related diseases irrespective of HPV type*: intention to treat analysis

Study or Subgroup	HPV vaccine Events	HPV vaccine Total	Control Events	Control Total	Weight	Risk Ratio IV, Random, 95% CI
**8.4.1 Cervical procedure: Cervarix (females, age 15-25; follow-up until 48 months)**						
HPV-008: Cervarix vs. Havrix (f, 15-25; 48)	212	9319	323	9325	18.4%	0.66 [0.55, 0.78]
**Subtotal (95% CI)**		**9319**		**9325**	**18.4%**	**0.66 [0.55, 0.78]**
Total events	212		323			
Heterogeneity: Not applicable						
Test for overall effect: Z = 4.82 (P < 0.00001)						
**8.4.2 Cervical procedure: Cervarix (females, age 24-72; follow-up until 36 months)**						
HPV-015: Cervarix vs. Al(OH)3 (f, 24-72; 36)	88	2882	110	2871	11.3%	0.80 [0.61, 1.05]
**Subtotal (95% CI)**		**2882**		**2871**	**11.3%**	**0.80 [0.61, 1.05]**
Total events	88		110			
Heterogeneity: Not applicable						
Test for overall effect: Z = 1.61 (P = 0.11)						
**8.4.3 Cervical procedure: Gardasil (females, age 15-26; follow-up until 45 months)**						
V501-013: Gardasil vs. AAHS (f, 16-24; 45)	55	2723	94	2732	8.9%	0.59 [0.42, 0.82]
V501-015: Gardasil vs. AAHS (f, 15-26; 36)	361	6087	472	6080	21.9%	0.76 [0.67, 0.87]
**Subtotal (95% CI)**		**8810**		**8812**	**30.8%**	**0.70 [0.55, 0.89]**
Total events	416		566			
Heterogeneity: Tau² = 0.02; Chi² = 2.13, df = 1 (P = 0.14); I² = 53%						
Test for overall effect: Z = 2.88 (P = 0.004)						
**8.4.4 Cervical procedure: Gardasil (females, age 21-46; follow-up until 48 months)**						
V501-019: Gardasil vs. AAHS (f, 21-46; 48)	128	1911	140	1908	13.9%	0.91 [0.72, 1.15]
**Subtotal (95% CI)**		**1911**		**1908**	**13.9%**	**0.91 [0.72, 1.15]**
Total events	128		140			
Heterogeneity: Not applicable						
Test for overall effect: Z = 0.77 (P = 0.44)						
**8.4.5 External genital lesion (EGL) procedure: Gardasil (females, age 16-24; follow-up until 45 months)**						
V501-013: Gardasil vs. AAHS (f, 16-24; 45)	43	2723	85	2732	7.7%	0.51 [0.35, 0.73]
**Subtotal (95% CI)**		**2723**		**2732**	**7.7%**	**0.51 [0.35, 0.73]**
Total events	43		85			
Heterogeneity: Not applicable						
Test for overall effect: Z = 3.66 (P = 0.0002)						
**8.4.6 External genital lesion (EGL) procedure: Gardasil (females, age 21-46; follow-up until 48 months)**						
V501-019: Gardasil vs. AAHS (f, 21-46; 48)	26	1911	30	1908	4.3%	0.87 [0.51, 1.46]
**Subtotal (95% CI)**		**1911**		**1908**	**4.3%**	**0.87 [0.51, 1.46]**
Total events	26		30			
Heterogeneity: Not applicable						
Test for overall effect: Z = 0.54 (P = 0.59)						
**8.4.7 External genital lesion (EGL) procedure: Gardasil (males, age 15-27; follow-up until 36 months)**						
V501-020: Gardasil vs. AAHS (m, 15-27; 36)	105	2032	162	2033	13.5%	0.65 [0.51, 0.82]
**Subtotal (95% CI)**		**2032**		**2033**	**13.5%**	**0.65 [0.51, 0.82]**
Total events	105		162			
Heterogeneity: Not applicable						
Test for overall effect: Z = 3.57 (P = 0.0004)						
**Total (95% CI)**		**29588**		**29589**	**100.0%**	**0.71 [0.63, 0.80]**
Total events	1018		1416			
Heterogeneity: Tau² = 0.01; Chi² = 12.72, df = 7 (P = 0.08); I² = 45%						
Test for overall effect: Z = 5.70 (P < 0.00001)						
Test for subgroup differences: Chi² = 10.43, df = 6 (P = 0.11), I² = 42.4%						

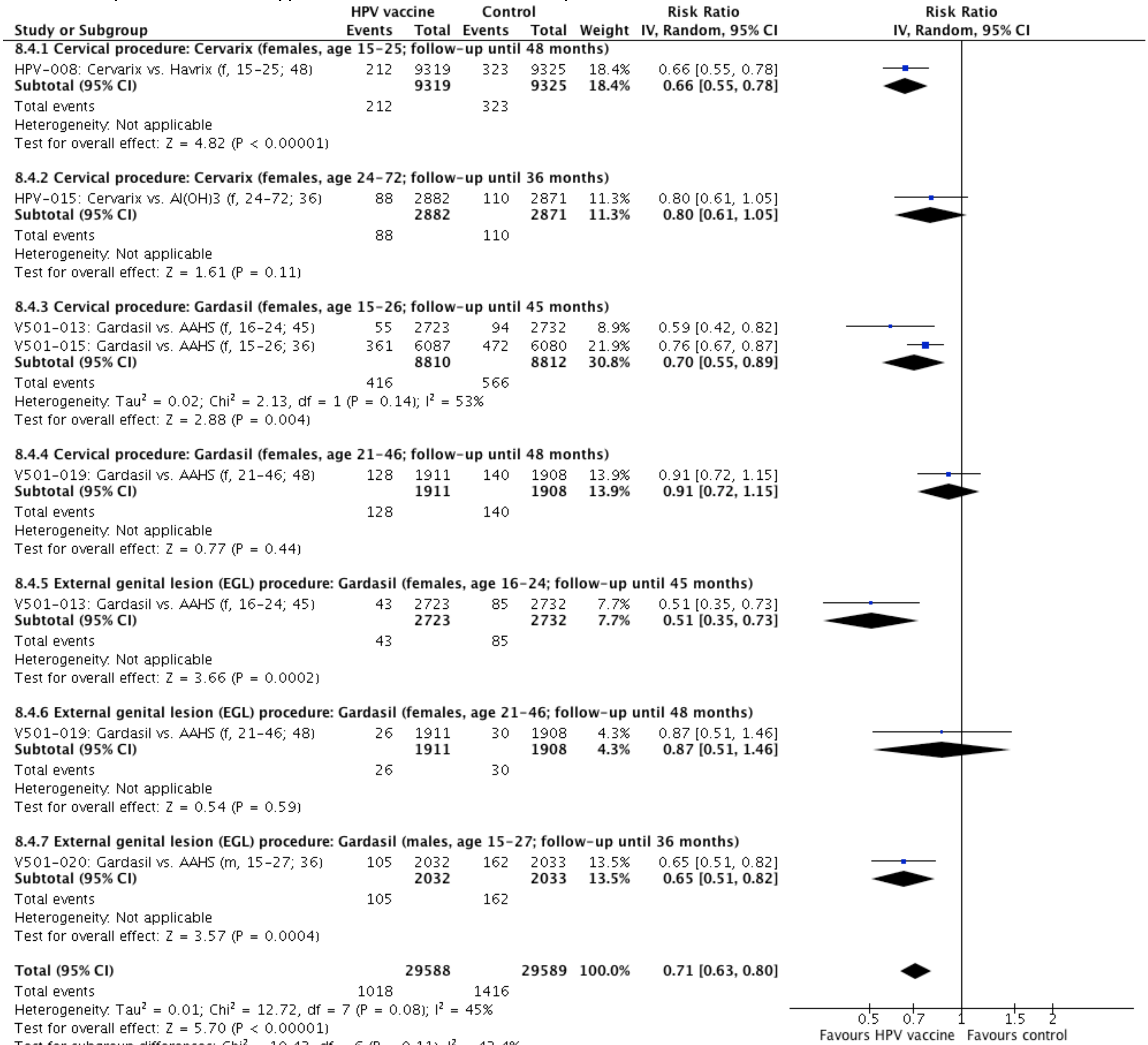

*8.4. Risk ratio for GlaxoSmithKline studies (i.e., HPV-0xx): **0.70 [0.59, 0.84]**; risk ratio for Merck Sharp & Dohme studies (i.e., V50x-xxx): **0.71 [0.60, 0.83]**. Only cervical procedure: 844 in the HPV vaccine group vs. 1,139 in the control group, risk ratio **0.74 [0.65, 0.84]**; only EGL procedure: 174 vs. 277, risk ratio **0.63 [0.50, 0.80]**.

9. <u>Fatal harms</u>

9.1. Number of participants with fatal harms*: intention to treat analysis

Study or Subgroup	HPV vaccine Events	HPV vaccine Total	Control Events	Control Total	Weight	Risk Ratio IV, Random, 95% CI
**9.1.1 Fatal harms: Cervarix vs. Al(OH)3 (females, age 24-72; follow-up until 36 months)**						
HPV-015: Cervarix vs. Al(OH)3 (f, 24-72; 36)	13	2882	5	2871	18.7%	2.59 [0.92, 7.26]
**Subtotal (95% CI)**		**2882**		**2871**	**18.7%**	**2.59 [0.92, 7.26]**
Total events	13		5			
Heterogeneity: Not applicable						
Test for overall effect: Z = 1.81 (P = 0.07)						
**9.1.2 Fatal harms: Cervarix vs. Aimmugen: hepatitis A vaccine (females, age 19-25; follow-up until 7 months)**						
HPV-032: Cervarix vs. Aimmugen (f, 19-25; 7)	1	519	0	521	3.3%	3.01 [0.12, 73.76]
**Subtotal (95% CI)**		**519**		**521**	**3.3%**	**3.01 [0.12, 73.76]**
Total events	1		0			
Heterogeneity: Not applicable						
Test for overall effect: Z = 0.68 (P = 0.50)						
**9.1.3 Fatal harms: Cervarix vs. Engerix: hepatitis B vaccine (females, age 26-46; follow-up until 12 months)**						
HPV-069: Cervarix vs. Engerix (f, 26-46; 12)	1	606	0	606	3.3%	3.00 [0.12, 73.50]
**Subtotal (95% CI)**		**606**		**606**	**3.3%**	**3.00 [0.12, 73.50]**
Total events	1		0			
Heterogeneity: Not applicable						
Test for overall effect: Z = 0.67 (P = 0.50)						
**9.1.4 Fatal harms: Cervarix vs. Havrix: hepatitis A vaccine (females, age 15-25; follow-up until 48 months)**						
HPV-008: Cervarix vs. Havrix (f, 15-25; 48)	10	9319	13	9325	23.2%	0.77 [0.34, 1.75]
**Subtotal (95% CI)**		**9319**		**9325**	**23.2%**	**0.77 [0.34, 1.75]**
Total events	10		13			
Heterogeneity: Not applicable						
Test for overall effect: Z = 0.62 (P = 0.53)						
**9.1.5 Fatal harms: Gardasil vs. AAHS (females, age 15-26; follow-up until 45 months)**						
V501-013: Gardasil vs. AAHS (f, 16-24; 45)	2	2723	3	2732	9.0%	0.67 [0.11, 4.00]
V501-015: Gardasil vs. AAHS (f, 15-26; 36)	7	6087	5	6080	16.6%	1.40 [0.44, 4.40]
**Subtotal (95% CI)**		**8810**		**8812**	**25.6%**	**1.13 [0.43, 2.96]**
Total events	9		8			
Heterogeneity: $Tau^2 = 0.00$; $Chi^2 = 0.46$, df = 1 (P = 0.50); $I^2 = 0\%$						
Test for overall effect: Z = 0.24 (P = 0.81)						
**9.1.6 Fatal harms: Gardasil vs. AAHS (females, age 21-46; follow-up until 48 months)**						
V501-019: Gardasil vs. AAHS (f, 21-46; 48)	7	1911	1	1908	7.0%	6.99 [0.86, 56.75]
**Subtotal (95% CI)**		**1911**		**1908**	**7.0%**	**6.99 [0.86, 56.75]**
Total events	7		1			
Heterogeneity: Not applicable						
Test for overall effect: Z = 1.82 (P = 0.07)						
**9.1.7 Fatal harms: Gardasil vs. AAHS (males, age 15-27; follow-up until 36 months)**						
V501-020: Gardasil vs. AAHS (m, 15-27; 36)	3	2032	10	2033	14.4%	0.30 [0.08, 1.09]
**Subtotal (95% CI)**		**2032**		**2033**	**14.4%**	**0.30 [0.08, 1.09]**
Total events	3		10			
Heterogeneity: Not applicable						
Test for overall effect: Z = 1.83 (P = 0.07)						
**9.1.8 Fatal harms: HPV 16 vaccine vs. AAHS (females, age 16-25; follow-up until 48 months)**						
V501-005: HPV 16 vaccine vs. AAHS (f, 16-25; 48)	1	1204	1	1205	4.3%	1.00 [0.06, 15.98]
**Subtotal (95% CI)**		**1204**		**1205**	**4.3%**	**1.00 [0.06, 15.98]**
Total events	1		1			
Heterogeneity: Not applicable						
Test for overall effect: Z = 0.00 (P = 1.00)						
**Total (95% CI)**		**27283**		**27281**	**100.0%**	**1.19 [0.65, 2.19]**
Total events	45		38			
Heterogeneity: $Tau^2 = 0.24$; $Chi^2 = 11.49$, df = 8 (P = 0.18); $I^2 = 30\%$						
Test for overall effect: Z = 0.56 (P = 0.58)						
Test for subgroup differences: $Chi^2 = 11.02$, df = 7 (P = 0.14), $I^2 = 36.5\%$						

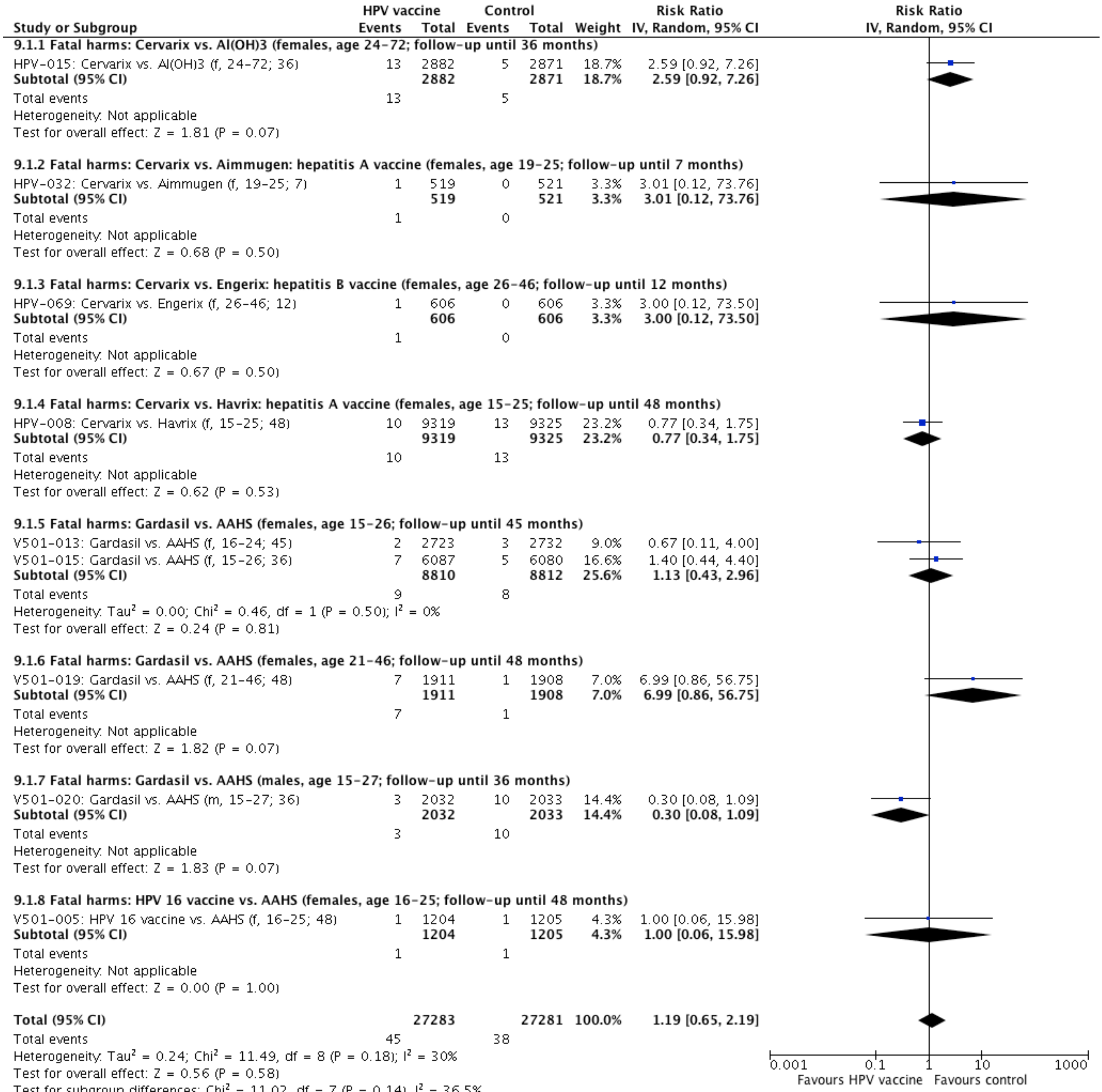

*9.1. Risk ratio for GlaxoSmithKline studies (i.e., HPV-0xx): 1.43 [0.65, 3.15]; risk ratio for Merck Sharp & Dohme studies (i.e., V50x-xxx): 1.08 [0.40, 2.96]. The most common fatal serious harms were: 'road traffic accident' (five in the HPV vaccine group and seven in the control group, risk ratio 0.77 [0.24, 2.46]); 'completed suicide' (four and eight, risk ratio 0.58 [0.15, 2.19]); 'cardiorespiratory arrest' (three and two, risk ratio 0.99 [0.13, 7.65]); 'gunshot wound' (two and three, risk ratio 0.74 [0.09, 5.85]); and 'homicide' (two and two, risk ratio 0.95 [0.14, 6.50]). The fatal serious harms most increased by the HPV vaccines were: 'cardiac arrest' (two in the HPV vaccine group and none in the control group, risk ratio 3.00 [0.31, 28.82]); 'traumatic intracranial haemorrhage' (two and none, risk ratio 3.00 [0.31, 28.82]); 'systemic lupus erythematosus' (two and none, risk ratio 3.00 [0.31, 28.82]); 'metastases to lung' (two and none, risk ratio 3.00 [0.31, 28.82]); and 'renal failure acute' (two and none, risk ratio 3.00 [0.31, 28.82]). The fatal serious harms most decreased by the HPV vaccines were: 'completed suicide' (four in the HPV vaccine group and eight in the control group, risk ratio 0.58 [0.15, 2.19]); and 'road traffic accident' (five and seven, risk ratio 0.77 [0.24, 2.46]).

9.2. Fatal harms judged related to the HPV vaccine by study investigator: intention to treat analysis

No fatal harm was judged HPV vaccine-related by the study investigators.

10. Serious harms

10.1. Serious harms*: intention to treat analysis

Study or Subgroup	HPV vaccine Events	HPV vaccine Total	Control Events	Control Total	Weight	Risk Ratio IV, Random, 95% CI
**10.1.1 Serious harms: Cervarix vs. Al(OH)3 (females, age 9-17; follow-up until 12 months)**						
HPV-058: Cervarix vs. Al(OH)3 (f, 9-17; 12)	5	374	2	376	0.2%	2.51 [0.49, 12.87]
**Subtotal (95% CI)**		**374**		**376**	**0.2%**	**2.51 [0.49, 12.87]**
Total events	5		2			
Heterogeneity: Not applicable						
Test for overall effect: Z = 1.11 (P = 0.27)						
**10.1.2 Serious harms: Cervarix vs. Al(OH)3 (females, age 15-25; follow-up until 7 months)**						
HPV-001: Cervarix vs. Al(OH)3 (f, 15-26; 27)	22	560	19	553	1.4%	1.14 [0.63, 2.09]
HPV-038: Cervarix vs. Al(OH)3 (f, 15-25; 7)	2	149	1	76	0.1%	1.02 [0.09, 11.07]
**Subtotal (95% CI)**		**709**		**629**	**1.5%**	**1.14 [0.63, 2.04]**
Total events	24		20			
Heterogeneity: Tau^2 = 0.00; Chi^2 = 0.01, df = 1 (P = 0.93); I^2 = 0%						
Test for overall effect: Z = 0.43 (P = 0.67)						
**10.1.3 Serious harms: Cervarix vs. Al(OH)3 (females, age 18-35; follow-up until 12 months)**						
HPV-003: Cervarix vs. Al(OH)3 (f, 18-30; 12)	1	31	0	30	0.1%	2.91 [0.12, 68.66]
HPV-023: Cervarix vs. Al(OH)3 (f, 21-32; 36)	4	222	5	211	0.3%	0.76 [0.21, 2.79]
HPV-031: Cervarix vs. Al(OH)3 (f, 18-35; 7)	2	176	4	178	0.2%	0.51 [0.09, 2.73]
HPV-035: Cervarix vs. Al(OH)3 (f, 18-35; 7)	3	150	1	150	0.1%	3.00 [0.32, 28.52]
**Subtotal (95% CI)**		**579**		**569**	**0.6%**	**0.94 [0.38, 2.30]**
Total events	10		10			
Heterogeneity: Tau^2 = 0.00; Chi^2 = 2.13, df = 3 (P = 0.55); I^2 = 0%						
Test for overall effect: Z = 0.14 (P = 0.89)						
**10.1.4 Serious harms: Cervarix vs. Al(OH)3 (females, age 24-72; follow-up until 36 months)**						
HPV-015: Cervarix vs. Al(OH)3 (f, 24-72; 36)	286	2882	266	2871	20.5%	1.07 [0.91, 1.26]
**Subtotal (95% CI)**		**2882**		**2871**	**20.5%**	**1.07 [0.91, 1.26]**
Total events	286		266			
Heterogeneity: Not applicable						
Test for overall effect: Z = 0.85 (P = 0.40)						
**10.1.5 Serious harms: Cervarix vs. Aimmugen: hepatitis A vaccine (females, age 22-29; follow-up until 12 months)**						
HPV-063: Cervarix vs. Aimmugen (f, 22-29; 12)	26	519	34	521	2.1%	0.77 [0.47, 1.26]
**Subtotal (95% CI)**		**519**		**521**	**2.1%**	**0.77 [0.47, 1.26]**
Total events	26		34			
Heterogeneity: Not applicable						
Test for overall effect: Z = 1.04 (P = 0.30)						
**10.1.6 Serious harms: Cervarix vs. Engerix: hepatitis B vaccine (females and males, age 9-16; follow-up until 72 months)**						
HPV-030: Cervarix vs. Engerix (f, 9-15; 12)	1	247	0	246	0.1%	2.99 [0.12, 72.99]
HPV-040: Cervarix vs. Engerix (f & m, 12-16; 72)	58	2436	25	1267	2.4%	1.21 [0.76, 1.92]
**Subtotal (95% CI)**		**2683**		**1513**	**2.5%**	**1.23 [0.78, 1.95]**
Total events	59		25			
Heterogeneity: Tau^2 = 0.00; Chi^2 = 0.30, df = 1 (P = 0.58); I^2 = 0%						
Test for overall effect: Z = 0.88 (P = 0.38)						
**10.1.7 Serious harms: Cervarix vs. Engerix: hepatitis B vaccine (females, age 26-46; follow-up until 12 months)**						
HPV-069: Cervarix vs. Engerix (f, 26-46; 12)	3	606	3	606	0.2%	1.00 [0.20, 4.93]
**Subtotal (95% CI)**		**606**		**606**	**0.2%**	**1.00 [0.20, 4.93]**
Total events	3		3			
Heterogeneity: Not applicable						
Test for overall effect: Z = 0.00 (P = 1.00)						
**10.1.8 Serious harms: Cervarix vs. Havrix: hepatitis A vaccine (females, age 9-15; follow-up until 12 months)**						
HPV-013: Cervarix vs. Havrix (f, 10-14; 12)	10	1035	13	1032	0.8%	0.77 [0.34, 1.74]
HPV-033: Cervarix vs. Havrix (f, 9-15; 7)	0	160	1	161	0.1%	0.34 [0.01, 8.17]
**Subtotal (95% CI)**		**1195**		**1193**	**0.8%**	**0.73 [0.33, 1.61]**
Total events	10		14			
Heterogeneity: Tau^2 = 0.00; Chi^2 = 0.24, df = 1 (P = 0.62); I^2 = 0%						
Test for overall effect: Z = 0.78 (P = 0.43)						
**10.1.9 Serious harms: Cervarix vs. Havrix: hepatitis A vaccine (females, age 15-25; follow-up until 48 months)**						
HPV-008: Cervarix vs. Havrix (f, 15-25; 48)	835	9319	829	9325	61.5%	1.01 [0.92, 1.10]
**Subtotal (95% CI)**		**9319**		**9325**	**61.5%**	**1.01 [0.92, 1.10]**
Total events	835		829			
Heterogeneity: Not applicable						
Test for overall effect: Z = 0.17 (P = 0.87)						
**10.1.10 Serious harms: Cervarix vs. Twinrix: hepatitis A and B vaccine (females, age 8-15; follow-up until 12 months)**						
HPV-029: Cervarix vs. Twinrix (f, 8-15; 12)	3	270	4	271	0.2%	0.75 [0.17, 3.33]
**Subtotal (95% CI)**		**270**		**271**	**0.2%**	**0.75 [0.17, 3.33]**
Total events	3		4			
Heterogeneity: Not applicable						
Test for overall effect: Z = 0.37 (P = 0.71)						
**10.1.11 Serious harms: Gardasil vs. carrier solution (females, age 9-16; follow-up until 18 months)**						
V501-018: Gardasil vs. carrier solu. (f&m,9-16;18)	6	1184	0	597	0.1%	6.56 [0.37, 116.26]
**Subtotal (95% CI)**		**1184**		**597**	**0.1%**	**6.56 [0.37, 116.26]**
Total events	6		0			
Heterogeneity: Not applicable						
Test for overall effect: Z = 1.28 (P = 0.20)						
**10.1.12 Serious harms: Gardasil vs. AAHS (females, age 15-26; follow-up until 45 months)**						
V501-013: Gardasil vs. AAHS (f, 16-24; 45)	49	2723	45	2732	3.2%	1.09 [0.73, 1.63]
V501-015: Gardasil vs. AAHS (f, 15-26; 36)	46	6087	56	6080	3.4%	0.82 [0.56, 1.21]
**Subtotal (95% CI)**		**8810**		**8812**	**6.6%**	**0.94 [0.71, 1.25]**
Total events	95		101			
Heterogeneity: Tau^2 = 0.00; Chi^2 = 1.01, df = 1 (P = 0.31); I^2 = 1%						
Test for overall effect: Z = 0.41 (P = 0.68)						
**10.1.13 Serious harms: Gardasil vs. AAHS (females, age 21-46; follow-up until 48 months)**						
V501-019: Gardasil vs. AAHS (f, 21-46; 48)	14	1911	16	1908	1.0%	0.87 [0.43, 1.78]
**Subtotal (95% CI)**		**1911**		**1908**	**1.0%**	**0.87 [0.43, 1.78]**
Total events	14		16			
Heterogeneity: Not applicable						
Test for overall effect: Z = 0.37 (P = 0.71)						
**10.1.14 Serious harms: Gardasil vs. AAHS (males, age 15-27; follow-up until 36 months)**						
V501-020: Gardasil vs. AAHS (m, 15-27; 36)	8	2032	11	2033	0.6%	0.73 [0.29, 1.81]
**Subtotal (95% CI)**		**2032**		**2033**	**0.6%**	**0.73 [0.29, 1.81]**
Total events	8		11			
Heterogeneity: Not applicable						
Test for overall effect: Z = 0.69 (P = 0.49)						
**10.1.15 Serious harms: Gardasil 9 vs. saline placebo (females, age 12-26; follow-up until 7 months)**						
V503-006: Gardasil 9 vs. saline (f, 12-26;7)	3	618	3	306	0.2%	0.50 [0.10, 2.44]
**Subtotal (95% CI)**		**618**		**306**	**0.2%**	**0.50 [0.10, 2.44]**
Total events	3		3			
Heterogeneity: Not applicable						
Test for overall effect: Z = 0.86 (P = 0.39)						
**10.1.16 Serious harms: HPV 16 vaccine vs. AAHS (females, age 16-25; follow-up until 48 months)**						
V501-005: HPV 16 vaccine vs. AAHS (f, 16-25; 48)	17	1204	19	1205	1.2%	0.90 [0.47, 1.71]
**Subtotal (95% CI)**		**1204**		**1205**	**1.2%**	**0.90 [0.47, 1.71]**
Total events	17		19			
Heterogeneity: Not applicable						
Test for overall effect: Z = 0.33 (P = 0.74)						
**Total (95% CI)**		**34895**		**32735**	**100.0%**	**1.01 [0.94, 1.08]**
Total events	1404		1357			
Heterogeneity: Tau^2 = 0.00; Chi^2 = 11.70, df = 22 (P = 0.96); I^2 = 0%						
Test for overall effect: Z = 0.26 (P = 0.79)						
Test for subgroup differences: Chi^2 = 8.00, df = 15 (P = 0.92), I^2 = 0%						

Risk Ratio
IV, Random, 95% CI

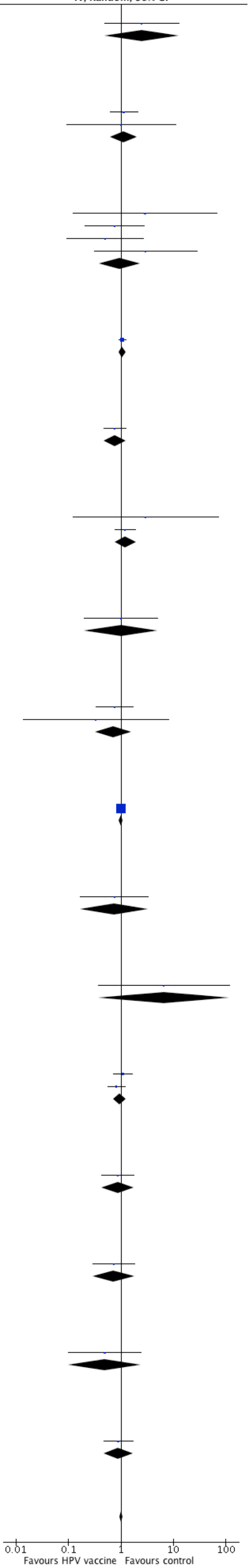

*10.1. Risk ratio for GlaxoSmithKline studies (i.e., HPV-0xx): 1.02 [0.95, 1.10]; risk ratio for Merck Sharp & Dohme studies (i.e., V50x-xxx): 0.91 [0.73, 1.15].

10.2. Serious harms judged related to the HPV vaccine by study investigator*: intention to treat analysis

Study or Subgroup	HPV vaccine Events	HPV vaccine Total	Control Events	Control Total	Weight	Risk Ratio IV, Random, 95% CI
**10.2.1 Serious harms judged HPV vaccine-related: Cervarix vs. Al(OH)3 (females, age 24-72; follow-up until 36 months)**						
HPV-015: Cervarix vs. Al(OH)3 (f, 24-72; 36)	6	2882	8	2871	22.5%	0.75 [0.26, 2.15]
**Subtotal (95% CI)**		**2882**		**2871**	**22.5%**	**0.75 [0.26, 2.15]**
Total events	6		8			
Heterogeneity: Not applicable						
Test for overall effect: Z = 0.54 (P = 0.59)						
**10.2.2 Serious harms judged HPV vaccine-related: Cervarix vs. Aimmugen: hepatitis A vaccine (females, age 19-25; follow-up until 12 months)**						
HPV-032: Cervarix vs. Aimmugen (f, 19-25; 7)	1	519	0	521	3.9%	3.01 [0.12, 73.76]
**Subtotal (95% CI)**		**519**		**521**	**3.9%**	**3.01 [0.12, 73.76]**
Total events	1		0			
Heterogeneity: Not applicable						
Test for overall effect: Z = 0.68 (P = 0.50)						
**10.2.3 Serious harms judged HPV vaccine-related: Cervarix vs. Engerix: hepatitis B vaccine (females and males, age 9-16; follow-up until 72 months)**						
HPV-040: Cervarix vs. Engerix (f & m, 12-16; 72)	26	2436	29	1267	40.6%	0.47 [0.28, 0.79]
**Subtotal (95% CI)**		**2436**		**1267**	**40.6%**	**0.47 [0.28, 0.79]**
Total events	26		29			
Heterogeneity: Not applicable						
Test for overall effect: Z = 2.85 (P = 0.004)						
**10.2.4 Serious harms judged HPV vaccine-related: Cervarix vs. Havrix: hepatitis A vaccine (females, age 15-25; follow-up until 48 months)**						
HPV-008: Cervarix vs. Havrix (f, 15-25; 48)	11	9319	6	9325	24.2%	1.83 [0.68, 4.96]
**Subtotal (95% CI)**		**9319**		**9325**	**24.2%**	**1.83 [0.68, 4.96]**
Total events	11		6			
Heterogeneity: Not applicable						
Test for overall effect: Z = 1.20 (P = 0.23)						
**10.2.5 Serious harms judged HPV vaccine-related: Gardasil vs. AAHS (females, age 15-26; follow-up until 45 months)**						
V501-013: Gardasil vs. AAHS (f, 16-24; 45)	1	2723	0	2732	3.9%	3.01 [0.12, 73.85]
**Subtotal (95% CI)**		**2723**		**2732**	**3.9%**	**3.01 [0.12, 73.85]**
Total events	1		0			
Heterogeneity: Not applicable						
Test for overall effect: Z = 0.67 (P = 0.50)						
**10.2.6 Serious harms judged HPV vaccine-related: Gardasil 9 vs. saline placebo (females, age 12-26; follow-up until 7 months)**						
V503-006: Gardasil 9 vs. saline (f, 12-26;7)	1	618	1	306	5.0%	0.50 [0.03, 7.89]
**Subtotal (95% CI)**		**618**		**306**	**5.0%**	**0.50 [0.03, 7.89]**
Total events	1		1			
Heterogeneity: Not applicable						
Test for overall effect: Z = 0.50 (P = 0.62)						

Risk Ratio
IV, Random, 95% CI

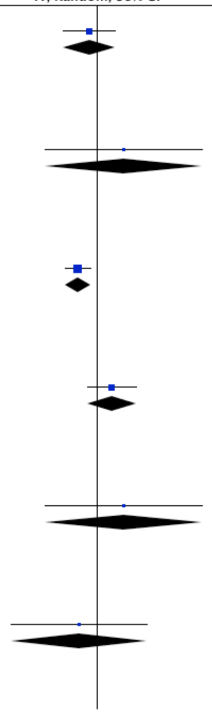

*10.2. Risk ratio for GlaxoSmithKline studies (i.e., HPV-0xx): 0.85 [0.39, 1.87]; risk ratio for Merck Sharp & Dohme studies (i.e., V50x-xxx): 1.07 [0.13, 8.69].

10.3. Number of participants that withdrew due to a serious harm*: intention to treat analysis

Study or Subgroup	HPV vaccine Events	HPV vaccine Total	Control Events	Control Total	Weight	Risk Ratio IV, Random, 95% CI
**10.3.1 Discontinuations due to serious harms: Cervarix vs. Al(OH)3 (females, age 15-26; follow-up until 27 months)**						
HPV-001: Cervarix vs. Al(OH)3 (f, 15-26; 27)	1	560	0	553	1.6%	2.96 [0.12, 72.57]
**Subtotal (95% CI)**		**560**		**553**	**1.6%**	**2.96 [0.12, 72.57]**
Total events	1		0			
Heterogeneity: Not applicable						
Test for overall effect: Z = 0.67 (P = 0.51)						
**10.3.2 Discontinuations due to serious harms: Cervarix vs. Al(OH)3 (females, age 18-35; follow-up until 7 months)**						
HPV-031: Cervarix vs. Al(OH)3 (f, 18-35; 7)	0	176	1	178	1.6%	0.34 [0.01, 8.22]
**Subtotal (95% CI)**		**176**		**178**	**1.6%**	**0.34 [0.01, 8.22]**
Total events	0		1			
Heterogeneity: Not applicable						
Test for overall effect: Z = 0.67 (P = 0.50)						
**10.3.3 Discontinuations due to serious harms: Cervarix vs. Al(OH)3 (females, age 24-72; follow-up until 36 months)**						
HPV-015: Cervarix vs. Al(OH)3 (f, 24-72; 36)	16	2882	9	2871	24.0%	1.77 [0.78, 4.00]
**Subtotal (95% CI)**		**2882**		**2871**	**24.0%**	**1.77 [0.78, 4.00]**
Total events	16		9			
Heterogeneity: Not applicable						
Test for overall effect: Z = 1.37 (P = 0.17)						
**10.3.4 Discontinuations due to serious harms: Cervarix vs. Aimmugen: hepatitis A vaccine (females, age 19-29; follow-up until 12 months)**						
HPV-032: Cervarix vs. Aimmugen (f, 19-25; 7)	1	519	0	521	1.6%	3.01 [0.12, 73.76]
HPV-063: Cervarix vs. Aimmugen (f, 22-29; 12)	2	375	0	377	1.7%	5.03 [0.24, 104.35]
**Subtotal (95% CI)**		**894**		**898**	**3.3%**	**3.94 [0.44, 35.62]**
Total events	3		0			
Heterogeneity: $Tau^2 = 0.00$; $Chi^2 = 0.05$, df = 1 (P = 0.82); $I^2 = 0\%$						
Test for overall effect: Z = 1.22 (P = 0.22)						
**10.3.5 Discontinuations due to serious harms: Cervarix vs. Engerix: hepatitis B vaccine (females, age 26-46; follow-up until 12 months)**						
HPV-069: Cervarix vs. Engerix (f, 26-46; 12)	1	606	1	606	2.1%	1.00 [0.06, 15.95]
**Subtotal (95% CI)**		**606**		**606**	**2.1%**	**1.00 [0.06, 15.95]**
Total events	1		1			
Heterogeneity: Not applicable						
Test for overall effect: Z = 0.00 (P = 1.00)						
**10.3.6 Discontinuations due to serious harms: Cervarix vs. Havrix: hepatitis A vaccine (females, age 10-14; follow-up until 12 months)**						
HPV-013: Cervarix vs. Havrix (f, 10-14; 12)	0	1035	1	1032	1.6%	0.33 [0.01, 8.15]
**Subtotal (95% CI)**		**1035**		**1032**	**1.6%**	**0.33 [0.01, 8.15]**
Total events	0		1			
Heterogeneity: Not applicable						
Test for overall effect: Z = 0.67 (P = 0.50)						
**10.3.7 Discontinuations due to serious harms: Cervarix vs. Havrix: hepatitis A vaccine (females, age 15-25; follow-up until 48 months)**						
HPV-008: Cervarix vs. Havrix (f, 15-25; 48)	12	9319	15	9325	27.7%	0.80 [0.37, 1.71]
**Subtotal (95% CI)**		**9319**		**9325**	**27.7%**	**0.80 [0.37, 1.71]**
Total events	12		15			
Heterogeneity: Not applicable						
Test for overall effect: Z = 0.57 (P = 0.57)						
**10.3.8 Discontinuations due to serious harms: Gardasil vs. carrier solution (females and males, age 9-16; follow-up until 18 months)**						
V501-018: Gardasil vs. carrier solu. (f&m,9-16;18)	1	1184	0	597	1.6%	1.51 [0.06, 37.11]
**Subtotal (95% CI)**		**1184**		**597**	**1.6%**	**1.51 [0.06, 37.11]**
Total events	1		0			
Heterogeneity: Not applicable						
Test for overall effect: Z = 0.25 (P = 0.80)						
**10.3.9 Discontinuations due to serious harms: Gardasil vs. AAHS (females, age 15-26; follow-up until 45 months)**						
V501-013: Gardasil vs. AAHS (f, 16-24; 45)	2	2723	3	2732	5.0%	0.67 [0.11, 4.00]
V501-015: Gardasil vs. AAHS (f, 15-26; 36)	7	6087	6	6080	13.4%	1.17 [0.39, 3.47]
**Subtotal (95% CI)**		**8810**		**8812**	**18.4%**	**1.00 [0.40, 2.54]**
Total events	9		9			
Heterogeneity: $Tau^2 = 0.00$; $Chi^2 = 0.27$, df = 1 (P = 0.60); $I^2 = 0\%$						
Test for overall effect: Z = 0.01 (P = 1.00)						
**10.3.10 Discontinuations due to serious harms: Gardasil vs. AAHS (females, age 21-46; follow-up until 48 months)**						
V501-019: Gardasil vs. AAHS (f, 21-46; 48)	7	1911	2	1908	6.5%	3.49 [0.73, 16.80]
**Subtotal (95% CI)**		**1911**		**1908**	**6.5%**	**3.49 [0.73, 16.80]**
Total events	7		2			
Heterogeneity: Not applicable						
Test for overall effect: Z = 1.56 (P = 0.12)						
**10.3.11 Discontinuations due to serious harms: Gardasil vs. AAHS (males, age 15-27; follow-up until 36 months)**						
V501-020: Gardasil vs. AAHS (m, 15-27; 36)	3	2032	10	2033	9.6%	0.30 [0.08, 1.09]
**Subtotal (95% CI)**		**2032**		**2033**	**9.6%**	**0.30 [0.08, 1.09]**
Total events	3		10			
Heterogeneity: Not applicable						
Test for overall effect: Z = 1.83 (P = 0.07)						
**10.3.12 Discontinuations due to serious harms: HPV 16 vaccine vs. AAHS (females, age 16-25; follow-up until 48 months)**						
V501-005: HPV 16 vaccine vs. AAHS (f, 16-25; 48)	1	1204	1	1205	2.1%	1.00 [0.06, 15.98]
**Subtotal (95% CI)**		**1204**		**1205**	**2.1%**	**1.00 [0.06, 15.98]**
Total events	1		1			
Heterogeneity: Not applicable						
Test for overall effect: Z = 0.00 (P = 1.00)						
**Total (95% CI)**		**30613**		**30018**	**100.0%**	**1.08 [0.72, 1.61]**
Total events	54		49			
Heterogeneity: $Tau^2 = 0.00$; $Chi^2 = 11.09$, df = 13 (P = 0.60); $I^2 = 0\%$						
Test for overall effect: Z = 0.37 (P = 0.71)						
Test for subgroup differences: $Chi^2 = 10.77$, df = 11 (P = 0.46), $I^2 = 0\%$						

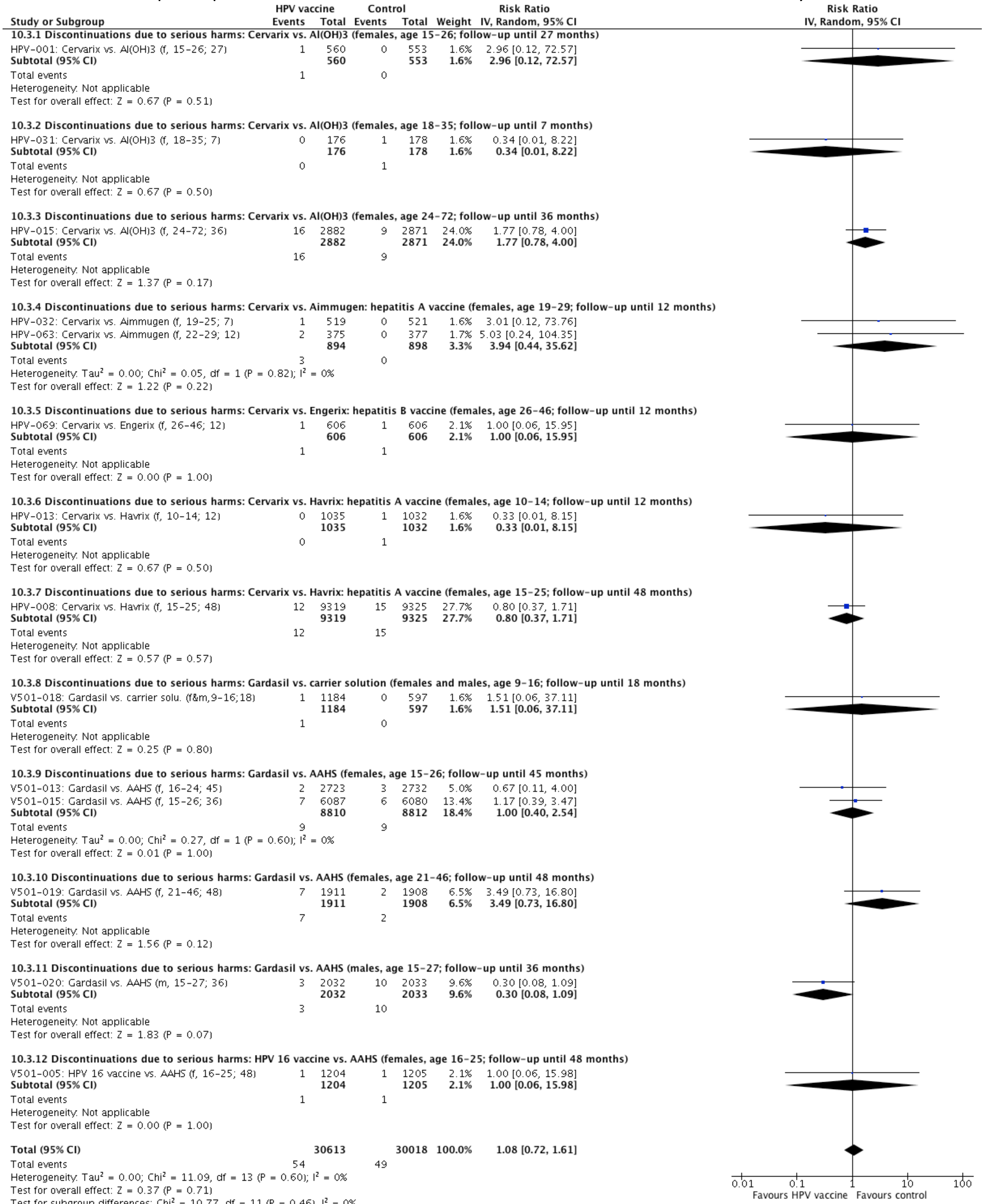

*10.3. Risk ratio for GlaxoSmithKline studies (i.e., HPV-0xx): 1.18 [0.71, 1.97]; risk ratio for Merck Sharp & Dohme studies (i.e., V50x-xxx): 0.94 [0.45, 1.98].

10.4. Serious harms reported within the MedDRA system organ class 'blood and lymphatic system disorders (10005329)'*: intention to treat analysis

Study or Subgroup	HPV vaccine Events	HPV vaccine Total	Control Events	Control Total	Weight	Risk Ratio IV, Random, 95% CI
**10.4.1 Serious blood and lymphatic system disorders: Cervarix vs. Al(OH)3 (females, age 24-72; follow-up until 36 months)**						
HPV-015: Cervarix vs. Al(OH)3 (f, 24-72; 36)	0	2882	4	2871	7.6%	0.11 [0.01, 2.05]
**Subtotal (95% CI)**		**2882**		**2871**	**7.6%**	**0.11 [0.01, 2.05]**
Total events	0		4			
Heterogeneity: Not applicable						
Test for overall effect: Z = 1.48 (P = 0.14)						
**10.4.2 Serious blood and lymphatic system disorders: Cervarix vs. Aimmugen: hepatitis A vaccine (females, age 22-29; follow-up until 12 months)**						
HPV-063: Cervarix vs. Aimmugen (f, 22-29; 12)	1	375	0	377	6.3%	3.02 [0.12, 73.80]
**Subtotal (95% CI)**		**375**		**377**	**6.3%**	**3.02 [0.12, 73.80]**
Total events	1		0			
Heterogeneity: Not applicable						
Test for overall effect: Z = 0.68 (P = 0.50)						
**10.4.3 Serious blood and lymphatic system disorders: Cervarix vs. Engerix: hepatitis B vaccine (females and males, age 12-16; follow-up until 72 months)**						
HPV-040: Cervarix vs. Engerix (f & m, 12-16; 72)	1	2436	0	1267	6.3%	1.56 [0.06, 38.29]
**Subtotal (95% CI)**		**2436**		**1267**	**6.3%**	**1.56 [0.06, 38.29]**
Total events	1		0			
Heterogeneity: Not applicable						
Test for overall effect: Z = 0.27 (P = 0.79)						
**10.4.4 Serious blood and lymphatic system disorders: Cervarix vs. Havrix: hepatitis A vaccine (females, age 10-14; follow-up until 12 months)**						
HPV-013: Cervarix vs. Havrix (f, 10-14; 12)	0	1035	1	1032	6.3%	0.33 [0.01, 8.15]
**Subtotal (95% CI)**		**1035**		**1032**	**6.3%**	**0.33 [0.01, 8.15]**
Total events	0		1			
Heterogeneity: Not applicable						
Test for overall effect: Z = 0.67 (P = 0.50)						
**10.4.5 Serious blood and lymphatic system disorders: Cervarix vs. Havrix: hepatitis A vaccine (females, age 15-25; follow-up until 48 months)**						
HPV-008: Cervarix vs. Havrix (f, 15-25; 48)	7	9319	6	9325	54.5%	1.17 [0.39, 3.47]
**Subtotal (95% CI)**		**9319**		**9325**	**54.5%**	**1.17 [0.39, 3.47]**
Total events	7		6			
Heterogeneity: Not applicable						
Test for overall effect: Z = 0.28 (P = 0.78)						
**10.4.6 Serious blood and lymphatic system disorders: Gardasil vs. carrier solution (females, age 9-16; follow-up until 18 months)**						
V501-018: Gardasil vs. carrier solu. (f&m,9-16;18)	1	617	0	322	6.3%	1.57 [0.06, 38.38]
**Subtotal (95% CI)**		**617**		**322**	**6.3%**	**1.57 [0.06, 38.38]**
Total events	1		0			
Heterogeneity: Not applicable						
Test for overall effect: Z = 0.28 (P = 0.78)						
**10.4.7 Serious blood and lymphatic system disorders: Gardasil vs. AAHS (females, age 16-25; follow-up until 45 months)**						
V501-013: Gardasil vs. AAHS (f, 16-24; 45)	1	2723	0	2732	6.3%	3.01 [0.12, 73.85]
**Subtotal (95% CI)**		**2723**		**2732**	**6.3%**	**3.01 [0.12, 73.85]**
Total events	1		0			
Heterogeneity: Not applicable						
Test for overall effect: Z = 0.67 (P = 0.50)						
**10.4.8 Serious blood and lymphatic system disorders: Gardasil vs. AAHS (females, age 21-46; follow-up until 48 months)**						
V501-019: Gardasil vs. AAHS (f, 21-46; 48)	0	1911	1	1908	6.3%	0.33 [0.01, 8.16]
**Subtotal (95% CI)**		**1911**		**1908**	**6.3%**	**0.33 [0.01, 8.16]**
Total events	0		1			
Heterogeneity: Not applicable						
Test for overall effect: Z = 0.67 (P = 0.50)						
**Total (95% CI)**		**21298**		**19834**	**100.0%**	**0.97 [0.44, 2.18]**
Total events	11		12			
Heterogeneity: $Tau^2 = 0.00$; $Chi^2 = 4.23$, df = 7 (P = 0.75); $I^2 = 0\%$						
Test for overall effect: Z = 0.06 (P = 0.95)						
Test for subgroup differences: $Chi^2 = 4.23$, df = 7 (P = 0.75), $I^2 = 0\%$						

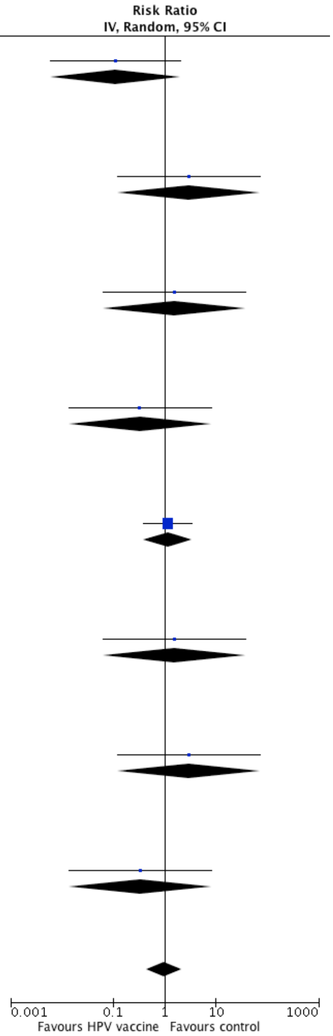

*10.4. Risk ratio for GlaxoSmithKline studies (i.e., HPV-0xx): 0.94 [0.38, 2.29]; risk ratio for Merck Sharp & Dohme studies (i.e., V50x-xxx): 1.16 [0.18, 7.36].

10.5. Serious harms reported within the MedDRA system organ class 'cardiac disorders (10007541)'*: intention to treat analysis

Study or Subgroup	HPV vaccine Events	HPV vaccine Total	Control Events	Control Total	Weight	Risk Ratio IV, Random, 95% CI
**10.5.1 Serious cardiac disorders: Cervarix vs. Al(OH)3 (females, age 24-72; follow-up until 36 months)**						
HPV-015: Cervarix vs. Al(OH)3 (f, 24-72; 36)	5	2882	7	2871	27.1%	0.71 [0.23, 2.24]
**Subtotal (95% CI)**		**2882**		**2871**	**27.1%**	**0.71 [0.23, 2.24]**
Total events	5		7			
Heterogeneity: Not applicable						
Test for overall effect: Z = 0.58 (P = 0.56)						
**10.5.2 Serious cardiac disorders: Cervarix vs. Engerix: hepatitis B vaccine (females, age 26-46; follow-up until 12 months)**						
HPV-069: Cervarix vs. Engerix (f, 26-46; 12)	0	606	3	606	6.8%	0.14 [0.01, 2.76]
**Subtotal (95% CI)**		**606**		**606**	**6.8%**	**0.14 [0.01, 2.76]**
Total events	0		3			
Heterogeneity: Not applicable						
Test for overall effect: Z = 1.29 (P = 0.20)						
**10.5.3 Serious cardiac disorders: Cervarix vs. Havrix: hepatitis A vaccine (females, age 15-25; follow-up until 48 months)**						
HPV-008: Cervarix vs. Havrix (f, 15-25; 48)	11	9319	7	9325	32.6%	1.57 [0.61, 4.05]
**Subtotal (95% CI)**		**9319**		**9325**	**32.6%**	**1.57 [0.61, 4.05]**
Total events	11		7			
Heterogeneity: Not applicable						
Test for overall effect: Z = 0.94 (P = 0.35)						
**10.5.4 Serious cardiac disorders: Gardasil vs. AAHS (females, age 15-26; follow-up until 36 months)**						
V501-015: Gardasil vs. AAHS (f, 15-26; 36)	3	6087	1	6080	10.7%	3.00 [0.31, 28.80]
**Subtotal (95% CI)**		**6087**		**6080**	**10.7%**	**3.00 [0.31, 28.80]**
Total events	3		1			
Heterogeneity: Not applicable						
Test for overall effect: Z = 0.95 (P = 0.34)						
**10.5.5 Serious cardiac disorders: Gardasil vs. AAHS (females, age 21-46; follow-up until 48 months)**						
V501-019: Gardasil vs. AAHS (f, 21-46; 48)	8	1911	0	1908	7.2%	16.97 [0.98, 293.86]
**Subtotal (95% CI)**		**1911**		**1908**	**7.2%**	**16.97 [0.98, 293.86]**
Total events	8		0			
Heterogeneity: Not applicable						
Test for overall effect: Z = 1.95 (P = 0.05)						
**10.5.6 Serious cardiac disorders: Gardasil vs. AAHS (males, age 15-27; follow-up until 36 months)**						
V501-020: Gardasil vs. AAHS (m, 15-27; 36)	1	2032	2	2033	9.7%	0.50 [0.05, 5.51]
**Subtotal (95% CI)**		**2032**		**2033**	**9.7%**	**0.50 [0.05, 5.51]**
Total events	1		2			
Heterogeneity: Not applicable						
Test for overall effect: Z = 0.57 (P = 0.57)						
**10.5.7 Serious cardiac disorders: HPV 16 vaccine vs. AAHS (females, age 16-25; follow-up until 48 months)**						
V501-005: HPV 16 vaccine vs. AAHS (f, 16-25; 48)	0	1204	1	1205	5.9%	0.33 [0.01, 8.18]
**Subtotal (95% CI)**		**1204**		**1205**	**5.9%**	**0.33 [0.01, 8.18]**
Total events	0		1			
Heterogeneity: Not applicable						
Test for overall effect: Z = 0.67 (P = 0.50)						
**Total (95% CI)**		**24041**		**24028**	**100.0%**	**1.12 [0.49, 2.55]**
Total events	28		21			
Heterogeneity: $Tau^2 = 0.31$; $Chi^2 = 8.15$, df = 6 (P = 0.23); $I^2 = 26\%$						
Test for overall effect: Z = 0.27 (P = 0.79)						
Test for subgroup differences: $Chi^2 = 8.15$, df = 6 (P = 0.23), $I^2 = 26.4\%$						

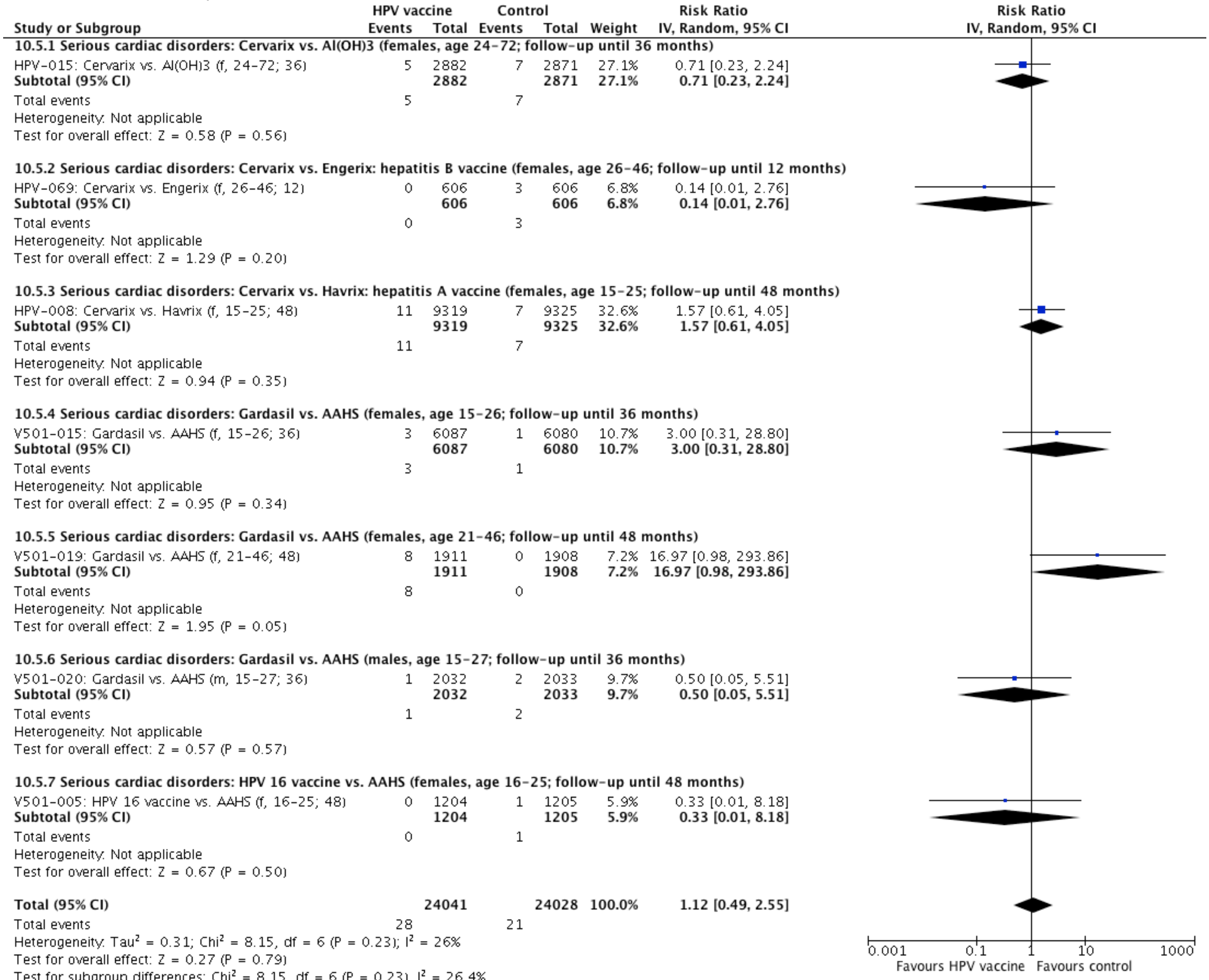

*10.5. Risk ratio for GlaxoSmithKline studies (i.e., HPV-0xx): 0.92 [0.36, 2.33]; risk ratio for Merck Sharp & Dohme studies (i.e., V50x-xxx): 1.76 [0.34, 9.21].

10.6. Serious harms reported within the MedDRA system organ class 'congenital familial and genetic disorders (10010331)'*: intention to treat analysis

Study or Subgroup	HPV vaccine Events	HPV vaccine Total	Control Events	Control Total	Weight	Risk Ratio IV, Random, 95% CI
**10.6.1 Serious congenital disorders: Cervarix vs. Al(OH)3 (females, age 18–35; follow-up until 7 months)**						
HPV-031: Cervarix vs. Al(OH)3 (f, 18–35; 7)	0	176	1	178	15.9%	0.34 [0.01, 8.22]
**Subtotal (95% CI)**		**176**		**178**	**15.9%**	**0.34 [0.01, 8.22]**
Total events	0		1			
Heterogeneity: Not applicable						
Test for overall effect: Z = 0.67 (P = 0.50)						
**10.6.2 Serious congenital disorders: Cervarix vs. Al(OH)3 (females, age 24–72; follow-up until 36 months)**						
HPV-015: Cervarix vs. Al(OH)3 (f, 24–72; 36)	0	2882	2	2871	17.6%	0.20 [0.01, 4.15]
**Subtotal (95% CI)**		**2882**		**2871**	**17.6%**	**0.20 [0.01, 4.15]**
Total events	0		2			
Heterogeneity: Not applicable						
Test for overall effect: Z = 1.04 (P = 0.30)						
**10.6.3 Serious congenital disorders: Cervarix vs. Aimmugen: hepatitis A vaccine (females, age 22–29; follow-up until 12 months)**						
HPV-063: Cervarix vs. Aimmugen (f, 22–29; 12)	0	375	1	377	15.9%	0.34 [0.01, 8.20]
**Subtotal (95% CI)**		**375**		**377**	**15.9%**	**0.34 [0.01, 8.20]**
Total events	0		1			
Heterogeneity: Not applicable						
Test for overall effect: Z = 0.67 (P = 0.50)						
**10.6.4 Serious congenital disorders: Cervarix vs. Engerix: hepatitis B vaccine (females and males, age 12–16; follow-up until 72 months)**						
HPV-040: Cervarix vs. Engerix (f & m, 12–16; 72)	1	2436	0	1267	15.8%	1.56 [0.06, 38.29]
**Subtotal (95% CI)**		**2436**		**1267**	**15.8%**	**1.56 [0.06, 38.29]**
Total events	1		0			
Heterogeneity: Not applicable						
Test for overall effect: Z = 0.27 (P = 0.79)						
**10.6.5 Serious congenital disorders: Cervarix vs. Engerix: hepatitis B vaccine (females, age 26–46; follow-up until 12 months)**						
HPV-069: Cervarix vs. Engerix (f, 26–46; 12)	0	606	1	606	15.8%	0.33 [0.01, 8.17]
**Subtotal (95% CI)**		**606**		**606**	**15.8%**	**0.33 [0.01, 8.17]**
Total events	0		1			
Heterogeneity: Not applicable						
Test for overall effect: Z = 0.67 (P = 0.50)						
**10.6.6 Serious congenital disorders: Cervarix vs. Havrix: hepatitis A vaccine (females, age 15–25; follow-up until 48 months)**						
HPV-008: Cervarix vs. Havrix (f, 15–25; 48)	0	9319	4	9325	19.0%	0.11 [0.01, 2.06]
**Subtotal (95% CI)**		**9319**		**9325**	**19.0%**	**0.11 [0.01, 2.06]**
Total events	0		4			
Heterogeneity: Not applicable						
Test for overall effect: Z = 1.47 (P = 0.14)						
**Total (95% CI)**		**15794**		**14624**	**100.0%**	**0.32 [0.09, 1.13]**
Total events	1		9			
Heterogeneity: Tau² = 0.00; Chi² = 1.54, df = 5 (P = 0.91); I² = 0%						
Test for overall effect: Z = 1.77 (P = 0.08)						
Test for subgroup differences: Chi² = 1.54, df = 5 (P = 0.91), I² = 0%						

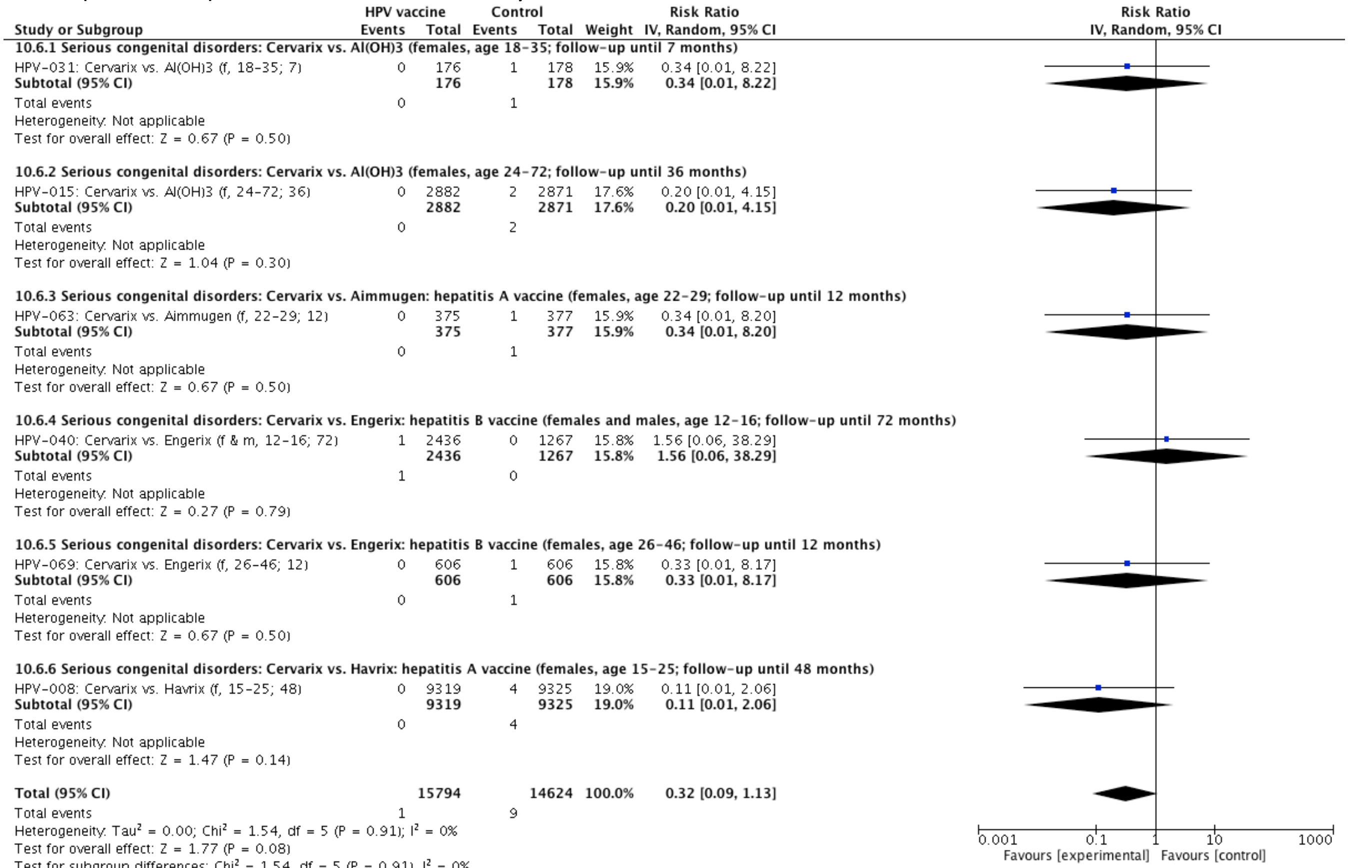

*10.6. Risk ratio for GlaxoSmithKline studies (i.e., HPV-0xx): 0.31 [0.08, 1.25]; risk ratio for Merck Sharp & Dohme studies (i.e., V50x-xxx): not applicable.

10.7. Serious harms reported within the MedDRA system organ class 'ear and labyrinth disorders (10013993)'*: intention to treat analysis

Study or Subgroup	HPV vaccine Events	HPV vaccine Total	Control Events	Control Total	Weight	Risk Ratio IV, Random, 95% CI
**10.7.1 Serious ear and labyrinth disorders: Cervarix vs. Al(OH)3 (females, age 24-72; follow-up until 36 months)**						
HPV-015: Cervarix vs. Al(OH)3 (f, 24-72; 36)	1	2882	3	2871	32.3%	0.33 [0.03, 3.19]
**Subtotal (95% CI)**		**2882**		**2871**	**32.3%**	**0.33 [0.03, 3.19]**
Total events	1		3			
Heterogeneity: Not applicable						
Test for overall effect: Z = 0.95 (P = 0.34)						
**10.7.2 Serious ear and labyrinth disorders: Cervarix vs. Havrix: hepatitis A vaccine (females, age 15-25; follow-up until 48 months)**						
HPV-008: Cervarix vs. Havrix (f, 15-25; 48)	2	9319	3	9325	51.6%	0.67 [0.11, 3.99]
**Subtotal (95% CI)**		**9319**		**9325**	**51.6%**	**0.67 [0.11, 3.99]**
Total events	2		3			
Heterogeneity: Not applicable						
Test for overall effect: Z = 0.44 (P = 0.66)						
**10.7.3 Serious ear and labyrinth disorders: Gardasil vs. AAHS (females, age 21-46; follow-up until 48 months)**						
V501-019: Gardasil vs. AAHS (f, 21-46; 48)	1	1911	0	1908	16.1%	3.00 [0.12, 73.48]
**Subtotal (95% CI)**		**1911**		**1908**	**16.1%**	**3.00 [0.12, 73.48]**
Total events	1		0			
Heterogeneity: Not applicable						
Test for overall effect: Z = 0.67 (P = 0.50)						
**Total (95% CI)**		**14112**		**14104**	**100.0%**	**0.68 [0.19, 2.45]**
Total events	4		6			
Heterogeneity: $Tau^2 = 0.00$; $Chi^2 = 1.21$, df = 2 (P = 0.55); $I^2 = 0\%$						
Test for overall effect: Z = 0.59 (P = 0.55)						
Test for subgroup differences: $Chi^2 = 1.21$, df = 2 (P = 0.55), $I^2 = 0\%$						

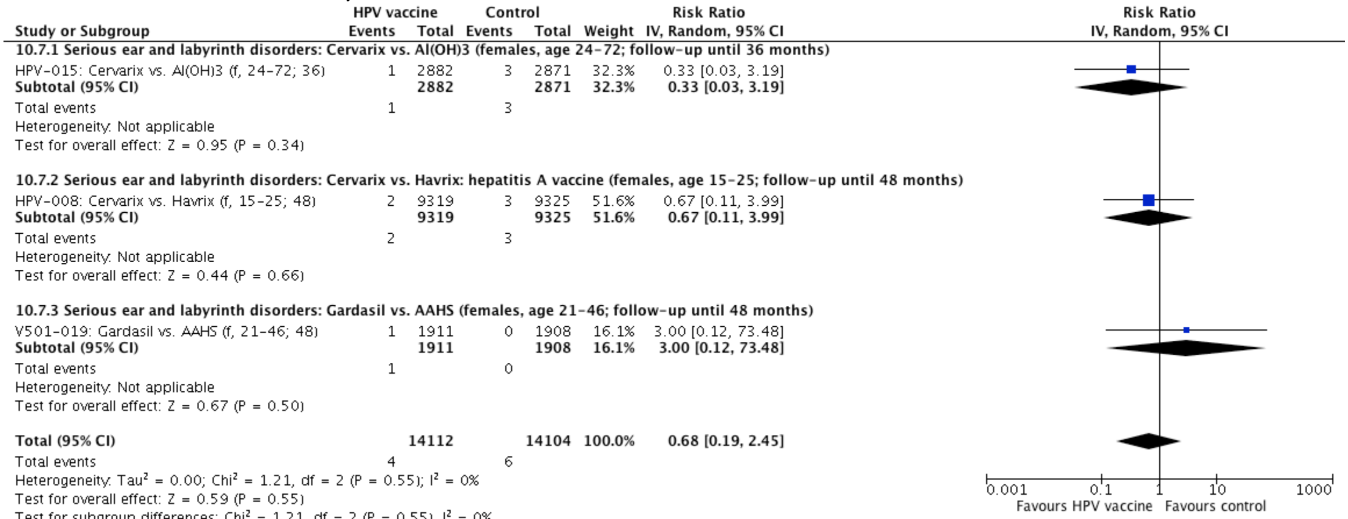

*10.7. Risk ratio for GlaxoSmithKline studies (i.e., HPV-0xx): 0.51 [0.13, 2.08]; risk ratio for Merck Sharp & Dohme studies (i.e., V50x-xxx): 3.00 [0.12, 73.48].

10.8. Serious harms reported within the MedDRA system organ class 'endocrine disorders (10014698)'*: intention to treat analysis

Study or Subgroup	HPV vaccine Events	HPV vaccine Total	Control Events	Control Total	Weight	Risk Ratio IV, Random, 95% CI
**10.8.1 Serious endocrine disorders: Cervarix vs. Al(OH)3 (females, age 24-72; follow-up until 36 months)**						
HPV-015: Cervarix vs. Al(OH)3 (f, 24-72; 36)	3	2882	3	2871	57.2%	1.00 [0.20, 4.93]
**Subtotal (95% CI)**		**2882**		**2871**	**57.2%**	**1.00 [0.20, 4.93]**
Total events	3		3			
Heterogeneity: Not applicable						
Test for overall effect: Z = 0.00 (P = 1.00)						
**10.8.2 Serious endocrine disorders: Cervarix vs. Havrix: hepatitis A vaccine (females, age 15-25; follow-up until 48 months)**						
HPV-008: Cervarix vs. Havrix (f, 15-25; 48)	3	9319	1	9325	28.6%	3.00 [0.31, 28.85]
**Subtotal (95% CI)**		**9319**		**9325**	**28.6%**	**3.00 [0.31, 28.85]**
Total events	3		1			
Heterogeneity: Not applicable						
Test for overall effect: Z = 0.95 (P = 0.34)						
**10.8.3 Serious endocrine disorders: Gardasil vs. AAHS (females, age 21-46; follow-up until 48 months)**						
V501-019: Gardasil vs. AAHS (f, 21-46; 48)	1	1911	0	1908	14.3%	3.00 [0.12, 73.48]
**Subtotal (95% CI)**		**1911**		**1908**	**14.3%**	**3.00 [0.12, 73.48]**
Total events	1		0			
Heterogeneity: Not applicable						
Test for overall effect: Z = 0.67 (P = 0.50)						
**Total (95% CI)**		**14112**		**14104**	**100.0%**	**1.60 [0.48, 5.35]**
Total events	7		4			
Heterogeneity: $Tau^2 = 0.00$; $Chi^2 = 0.78$, $df = 2$ ($P = 0.68$); $I^2 = 0\%$						
Test for overall effect: Z = 0.76 (P = 0.45)						
Test for subgroup differences: $Chi^2 = 0.78$, $df = 2$ ($P = 0.68$), $I^2 = 0\%$						

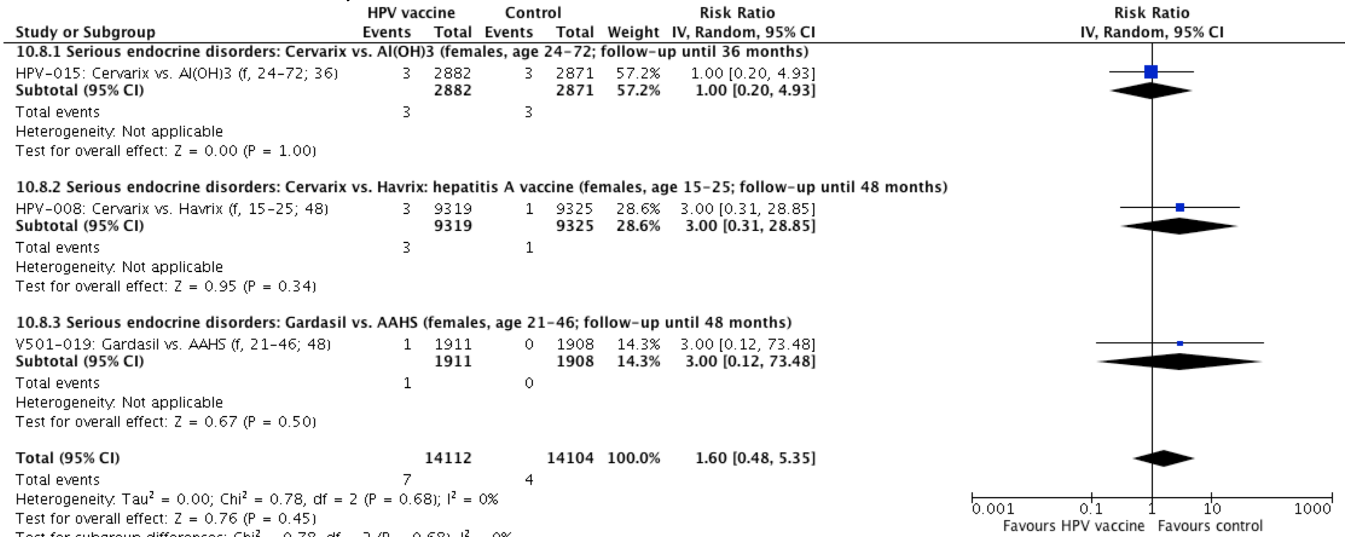

*10.8. Risk ratio for GlaxoSmithKline studies (i.e., HPV-0xx): 1.44 [0.39, 5.31]; risk ratio for Merck Sharp & Dohme studies (i.e., V50x-xxx): 3.00 [0.12, 73.48].

10.9. Serious harms reported within the MedDRA system organ class 'eye disorders (10015919)'*: intention to treat analysis

Study or Subgroup	HPV vaccine Events	HPV vaccine Total	Control Events	Control Total	Weight	Risk Ratio IV, Random, 95% CI
**10.9.1 Serious eye disorders: Cervarix vs. Al(OH)3 (females, age 18–35; follow-up until 7 months)**						
HPV-031: Cervarix vs. Al(OH)3 (f, 18–35; 7)	0	176	1	178	15.8%	0.34 [0.01, 8.22]
**Subtotal (95% CI)**		**176**		**178**	**15.8%**	**0.34 [0.01, 8.22]**
Total events	0		1			
Heterogeneity: Not applicable						
Test for overall effect: Z = 0.67 (P = 0.50)						
**10.9.2 Serious eye disorders: Cervarix vs. Al(OH)3 (females, age 24–72; follow-up until 36 months)**						
HPV-015: Cervarix vs. Al(OH)3 (f, 24–72; 36)	2	2882	1	2871	28.1%	1.99 [0.18, 21.96]
**Subtotal (95% CI)**		**2882**		**2871**	**28.1%**	**1.99 [0.18, 21.96]**
Total events	2		1			
Heterogeneity: Not applicable						
Test for overall effect: Z = 0.56 (P = 0.57)						
**10.9.3 Serious eye disorders: Cervarix vs. Havrix: hepatitis A vaccine (females, age 15–25; follow-up until 48 months)**						
HPV-008: Cervarix vs. Havrix (f, 15–25; 48)	4	9319	2	9325	56.1%	2.00 [0.37, 10.92]
**Subtotal (95% CI)**		**9319**		**9325**	**56.1%**	**2.00 [0.37, 10.92]**
Total events	4		2			
Heterogeneity: Not applicable						
Test for overall effect: Z = 0.80 (P = 0.42)						
**Total (95% CI)**		**12377**		**12374**	**100.0%**	**1.51 [0.42, 5.37]**
Total events	6		4			
Heterogeneity: Tau² = 0.00; Chi² = 1.00, df = 2 (P = 0.61); I² = 0%						
Test for overall effect: Z = 0.63 (P = 0.53)						
Test for subgroup differences: Chi² = 1.00, df = 2 (P = 0.61), I² = 0%						

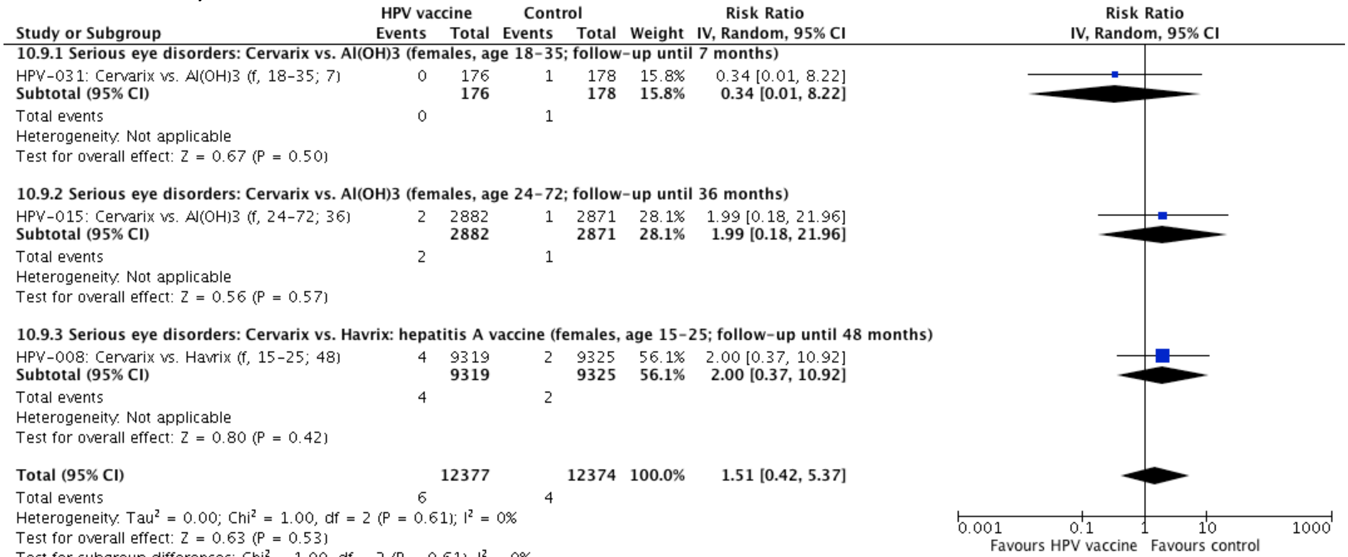

*10.9. Risk ratio for GlaxoSmithKline studies (i.e., HPV-0xx): 1.51 [0.42, 5.37]; risk ratio for Merck Sharp & Dohme studies (i.e., V50x-xxx): not applicable.

10.10. Serious harms reported within the MedDRA system organ class 'gastrointestinal disorders (10017947)'*: intention to treat analysis

Study or Subgroup	HPV vaccine Events	HPV vaccine Total	Control Events	Control Total	Weight	Risk Ratio IV, Random, 95% CI
**10.10.1 Serious gastrointestinal disorders: Cervarix vs. Al(OH)3 (females, age 18-35; follow-up until 7 months)**						
HPV-035: Cervarix vs. Al(OH)3 (f, 18-35; 7)	2	150	0	150	0.9%	5.00 [0.24, 103.28]
**Subtotal (95% CI)**		**150**		**150**	**0.9%**	**5.00 [0.24, 103.28]**
Total events	2		0			
Heterogeneity: Not applicable						
Test for overall effect: Z = 1.04 (P = 0.30)						
**10.10.2 Serious gastrointestinal disorders: Cervarix vs. Al(OH)3 (females, age 24-72; follow-up until 36 months)**						
HPV-015: Cervarix vs. Al(OH)3 (f, 24-72; 36)	24	2882	21	2871	23.1%	1.14 [0.64, 2.04]
**Subtotal (95% CI)**		**2882**		**2871**	**23.1%**	**1.14 [0.64, 2.04]**
Total events	24		21			
Heterogeneity: Not applicable						
Test for overall effect: Z = 0.44 (P = 0.66)						
**10.10.3 Serious gastrointestinal disorders: Cervarix vs. Aimmugen: hepatitis A vaccine (females, age 22-29; follow-up until 12 months)**						
HPV-063: Cervarix vs. Aimmugen (f, 22-29; 12)	0	375	3	377	0.9%	0.14 [0.01, 2.77]
**Subtotal (95% CI)**		**375**		**377**	**0.9%**	**0.14 [0.01, 2.77]**
Total events	0		3			
Heterogeneity: Not applicable						
Test for overall effect: Z = 1.29 (P = 0.20)						
**10.10.4 Serious gastrointestinal disorders: Cervarix vs. Engerix: hepatitis B vaccine (females and males, age 12-16; follow-up until 72 months)**						
HPV-040: Cervarix vs. Engerix (f & m, 12-16; 72)	5	2436	0	1267	0.9%	5.72 [0.32, 103.42]
**Subtotal (95% CI)**		**2436**		**1267**	**0.9%**	**5.72 [0.32, 103.42]**
Total events	5		0			
Heterogeneity: Not applicable						
Test for overall effect: Z = 1.18 (P = 0.24)						
**10.10.5 Serious gastrointestinal disorders: Cervarix vs. Havrix: hepatitis A vaccine (females, age 10-14; follow-up until 12 months)**						
HPV-013: Cervarix vs. Havrix (f, 10-14; 12)	2	1035	2	1032	2.0%	1.00 [0.14, 7.07]
**Subtotal (95% CI)**		**1035**		**1032**	**2.0%**	**1.00 [0.14, 7.07]**
Total events	2		2			
Heterogeneity: Not applicable						
Test for overall effect: Z = 0.00 (P = 1.00)						
**10.10.6 Serious gastrointestinal disorders: Cervarix vs. Havrix: hepatitis A vaccine (females, age 15-25; follow-up until 48 months)**						
HPV-008: Cervarix vs. Havrix (f, 15-25; 48)	66	9319	61	9325	65.3%	1.08 [0.77, 1.53]
**Subtotal (95% CI)**		**9319**		**9325**	**65.3%**	**1.08 [0.77, 1.53]**
Total events	66		61			
Heterogeneity: Not applicable						
Test for overall effect: Z = 0.45 (P = 0.65)						
**10.10.7 Serious gastrointestinal disorders: Cervarix vs. Twinrix: hepatitis A and B vaccine (females, age 8-15; follow-up until 12 months)**						
HPV-029: Cervarix vs. Twinrix (f, 8-15; 12)	0	270	1	271	0.8%	0.33 [0.01, 8.18]
**Subtotal (95% CI)**		**270**		**271**	**0.8%**	**0.33 [0.01, 8.18]**
Total events	0		1			
Heterogeneity: Not applicable						
Test for overall effect: Z = 0.67 (P = 0.50)						
**10.10.8 Serious gastrointestinal disorders: Gardasil vs. carrier solution (females, age 9-16; follow-up until 18 months)**						
V501-018: Gardasil vs. carrier solu. (f&m,9-16;18)	1	1184	0	597	0.8%	1.51 [0.06, 37.11]
**Subtotal (95% CI)**		**1184**		**597**	**0.8%**	**1.51 [0.06, 37.11]**
Total events	1		0			
Heterogeneity: Not applicable						
Test for overall effect: Z = 0.25 (P = 0.80)						
**10.10.9 Serious gastrointestinal disorders: Gardasil vs. AAHS (females, age 15-26; follow-up until 36 months)**						
V501-015: Gardasil vs. AAHS (f, 15-26; 36)	2	6087	2	6080	2.0%	1.00 [0.14, 7.09]
**Subtotal (95% CI)**		**6087**		**6080**	**2.0%**	**1.00 [0.14, 7.09]**
Total events	2		2			
Heterogeneity: Not applicable						
Test for overall effect: Z = 0.00 (P = 1.00)						
**10.10.10 Serious gastrointestinal disorders: Gardasil vs. AAHS (females, age 21-46; follow-up until 48 months)**						
V501-019: Gardasil vs. AAHS (f, 21-46; 48)	2	1911	0	1908	0.9%	4.99 [0.24, 103.91]
**Subtotal (95% CI)**		**1911**		**1908**	**0.9%**	**4.99 [0.24, 103.91]**
Total events	2		0			
Heterogeneity: Not applicable						
Test for overall effect: Z = 1.04 (P = 0.30)						
**10.10.11 Serious gastrointestinal disorders: HPV 16 vaccine vs. AAHS (females, age 16-25; follow-up until 48 months)**						
V501-005: HPV 16 vaccine vs. AAHS (f, 16-25; 48)	3	1204	2	1205	2.5%	1.50 [0.25, 8.97]
**Subtotal (95% CI)**		**1204**		**1205**	**2.5%**	**1.50 [0.25, 8.97]**
Total events	3		2			
Heterogeneity: Not applicable						
Test for overall effect: Z = 0.45 (P = 0.66)						
**Total (95% CI)**		**26853**		**25083**	**100.0%**	**1.12 [0.85, 1.48]**
Total events	107		92			
Heterogeneity: $Tau^2 = 0.00$; $Chi^2 = 5.69$, df = 10 (P = 0.84); $I^2 = 0\%$						
Test for overall effect: Z = 0.79 (P = 0.43)						
Test for subgroup differences: $Chi^2 = 5.69$, df = 10 (P = 0.84), $I^2 = 0\%$						

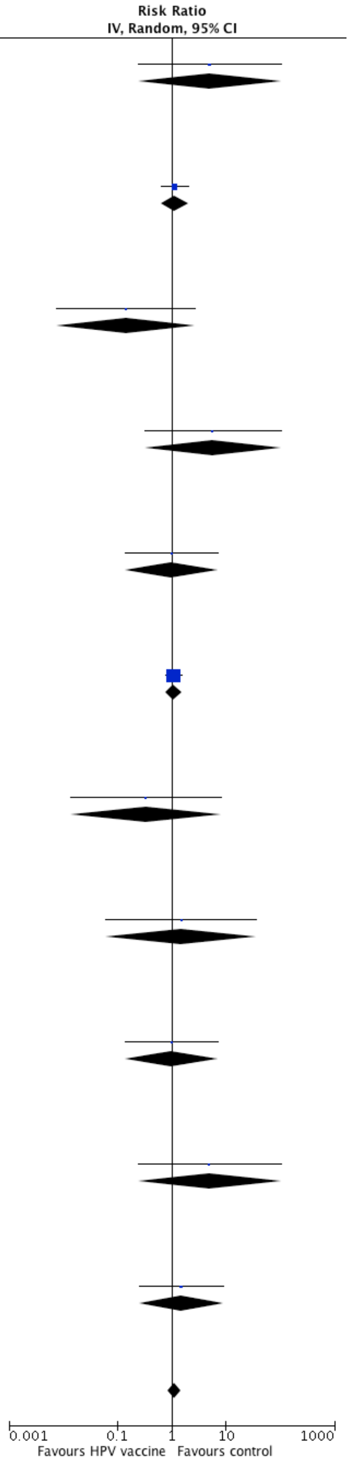

*10.10. Risk ratio for GlaxoSmithKline studies (i.e., HPV-0xx): 1.56 [0.50, 4.83]; risk ratio for Merck Sharp & Dohme studies (i.e., V50x-xxx): 1.10 [0.82, 1.46].

10.11. Serious harms reported within the MedDRA system organ class 'general disorders and administration site conditions (10018065)'*: intention to treat analysis

Study or Subgroup	HPV vaccine Events	HPV vaccine Total	Control Events	Control Total	Weight	Risk Ratio IV, Random, 95% CI
**10.11.1 Serious general disorders and administration site conditions: Cervarix vs. Al(OH)3 (females, age 24-72; follow-up until 36 months)**						
HPV-015: Cervarix vs. Al(OH)3 (f, 24-72; 36)	5	2882	2	2871	29.1%	2.49 [0.48, 12.83]
**Subtotal (95% CI)**		**2882**		**2871**	**29.1%**	**2.49 [0.48, 12.83]**
Total events	5		2			
Heterogeneity: Not applicable						
Test for overall effect: Z = 1.09 (P = 0.28)						
**10.11.2 Serious general disorders and administration site conditions: Cervarix vs. Aimmugen: hepatitis A vaccine (females, age 22-29; follow-up until 12 months)**						
HPV-063: Cervarix vs. Aimmugen (f, 22-29; 12)	0	375	1	377	7.7%	0.34 [0.01, 8.20]
**Subtotal (95% CI)**		**375**		**377**	**7.7%**	**0.34 [0.01, 8.20]**
Total events	0		1			
Heterogeneity: Not applicable						
Test for overall effect: Z = 0.67 (P = 0.50)						
**10.11.3 Serious general disorders and administration site conditions: Cervarix vs. Engerix: hepatitis B vaccine (females and males, age 12-16; follow-up until 72 months)**						
HPV-040: Cervarix vs. Engerix (f & m, 12-16; 72)	2	2436	0	1267	8.5%	2.60 [0.12, 54.15]
**Subtotal (95% CI)**		**2436**		**1267**	**8.5%**	**2.60 [0.12, 54.15]**
Total events	2		0			
Heterogeneity: Not applicable						
Test for overall effect: Z = 0.62 (P = 0.54)						
**10.11.4 Serious general disorders and administration site conditions: Cervarix vs. Havrix: hepatitis A vaccine (females, age 15-25; follow-up until 48 months)**						
HPV-008: Cervarix vs. Havrix (f, 15-25; 48)	5	9319	3	9325	38.2%	1.67 [0.40, 6.98]
**Subtotal (95% CI)**		**9319**		**9325**	**38.2%**	**1.67 [0.40, 6.98]**
Total events	5		3			
Heterogeneity: Not applicable						
Test for overall effect: Z = 0.70 (P = 0.48)						
**10.11.5 Serious general disorders and administration site conditions: Gardasil vs. AAHS (females, age 15-26; follow-up until 36 months)**						
V501-015: Gardasil vs. AAHS (f, 15-26; 36)	0	6087	3	6080	8.9%	0.14 [0.01, 2.76]
**Subtotal (95% CI)**		**6087**		**6080**	**8.9%**	**0.14 [0.01, 2.76]**
Total events	0		3			
Heterogeneity: Not applicable						
Test for overall effect: Z = 1.29 (P = 0.20)						
**10.11.6 Serious general disorders and administration site conditions: Gardasil vs. AAHS (males, age 15-27; follow-up until 36 months)**						
V501-020: Gardasil vs. AAHS (m, 15-27; 36)	1	2032	0	2033	7.6%	3.00 [0.12, 73.64]
**Subtotal (95% CI)**		**2032**		**2033**	**7.6%**	**3.00 [0.12, 73.64]**
Total events	1		0			
Heterogeneity: Not applicable						
Test for overall effect: Z = 0.67 (P = 0.50)						
**Total (95% CI)**		**23131**		**21953**	**100.0%**	**1.45 [0.60, 3.50]**
Total events	13		9			
Heterogeneity: $Tau^2 = 0.00$; $Chi^2 = 3.95$, df = 5 (P = 0.56); $I^2 = 0\%$						
Test for overall effect: Z = 0.82 (P = 0.41)						
Test for subgroup differences: $Chi^2 = 3.95$, df = 5 (P = 0.56), $I^2 = 0\%$						

Risk Ratio
IV, Random, 95% CI

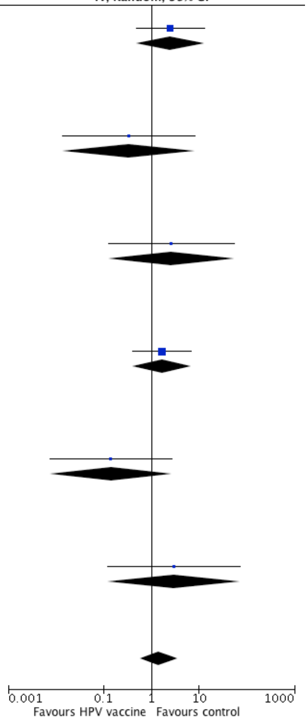

*10.11. Risk ratio for GlaxoSmithKline studies (i.e., HPV-0xx): 1.73 [0.66, 4.56]; risk ratio for Merck Sharp & Dohme studies (i.e., V50x-xxx): 0.61 [0.03, 12.14].

10.12. Serious harms reported within the MedDRA system organ class 'hepatobiliary disorders (10019805)'*: intention to treat analysis

Study or Subgroup	HPV vaccine Events	HPV vaccine Total	Control Events	Control Total	Weight	Risk Ratio IV, Random, 95% CI
**10.12.1 Serious hepatobiliary disorders: Cervarix vs. Al(OH)3 (females, age 24-72; follow-up until 36 months)**						
HPV-015: Cervarix vs. Al(OH)3 (f, 24-72; 36)	13	2882	15	2871	27.7%	0.86 [0.41, 1.81]
**Subtotal (95% CI)**		**2882**		**2871**	**27.7%**	**0.86 [0.41, 1.81]**
Total events	13		15			
Heterogeneity: Not applicable						
Test for overall effect: Z = 0.39 (P = 0.70)						
**10.12.2 Serious hepatobiliary disorders: Cervarix vs. Aimmugen: hepatitis A vaccine (females, age 22-29; follow-up until 12 months)**						
HPV-063: Cervarix vs. Aimmugen (f, 22-29; 12)	1	375	2	377	2.6%	0.50 [0.05, 5.52]
**Subtotal (95% CI)**		**375**		**377**	**2.6%**	**0.50 [0.05, 5.52]**
Total events	1		2			
Heterogeneity: Not applicable						
Test for overall effect: Z = 0.56 (P = 0.57)						
**10.12.3 Serious hepatobiliary disorders: Cervarix vs. Engerix: hepatitis B vaccine (females and males, age 12-16; follow-up until 72 months)**						
HPV-040: Cervarix vs. Engerix (f & m, 12-16; 72)	1	2436	0	1267	1.5%	1.56 [0.06, 38.29]
**Subtotal (95% CI)**		**2436**		**1267**	**1.5%**	**1.56 [0.06, 38.29]**
Total events	1		0			
Heterogeneity: Not applicable						
Test for overall effect: Z = 0.27 (P = 0.79)						
**10.12.4 Serious hepatobiliary disorders: Cervarix vs. Engerix: hepatitis B vaccine (females, age 26-46; follow-up until 12 months)**						
HPV-069: Cervarix vs. Engerix (f, 26-46; 12)	2	606	0	606	1.6%	5.00 [0.24, 103.93]
**Subtotal (95% CI)**		**606**		**606**	**1.6%**	**5.00 [0.24, 103.93]**
Total events	2		0			
Heterogeneity: Not applicable						
Test for overall effect: Z = 1.04 (P = 0.30)						
**10.12.5 Serious hepatobiliary disorders: Cervarix vs. Havrix: hepatitis A vaccine (females, age 15-25; follow-up until 48 months)**						
HPV-008: Cervarix vs. Havrix (f, 15-25; 48)	30	9319	31	9325	60.5%	0.97 [0.59, 1.60]
**Subtotal (95% CI)**		**9319**		**9325**	**60.5%**	**0.97 [0.59, 1.60]**
Total events	30		31			
Heterogeneity: Not applicable						
Test for overall effect: Z = 0.13 (P = 0.90)						
**10.12.6 Serious hepatobiliary disorders: Gardasil vs. AAHS (females, age 15-26; follow-up until 45 months)**						
V501-013: Gardasil vs. AAHS (f, 16-24; 45)	2	2723	0	2732	1.6%	5.02 [0.24, 104.44]
V501-015: Gardasil vs. AAHS (f, 15-26; 36)	1	6087	0	6080	1.5%	3.00 [0.12, 73.54]
**Subtotal (95% CI)**		**8810**		**8812**	**3.1%**	**3.93 [0.43, 35.56]**
Total events	3		0			
Heterogeneity: $Tau^2 = 0.00$; $Chi^2 = 0.05$, df = 1 (P = 0.82); $I^2 = 0\%$						
Test for overall effect: Z = 1.22 (P = 0.22)						
**10.12.7 Serious hepatobiliary disorders: Gardasil vs. AAHS (females, age 21-46; follow-up until 48 months)**						
V501-019: Gardasil vs. AAHS (f, 21-46; 48)	1	1911	0	1908	1.5%	3.00 [0.12, 73.48]
**Subtotal (95% CI)**		**1911**		**1908**	**1.5%**	**3.00 [0.12, 73.48]**
Total events	1		0			
Heterogeneity: Not applicable						
Test for overall effect: Z = 0.67 (P = 0.50)						
**10.12.8 Serious hepatobiliary disorders: HPV 16 vaccine vs. AAHS (females, age 16-25; follow-up until 48 months)**						
V501-005: HPV 16 vaccine vs. AAHS (f, 16-25; 48)	1	1204	0	1205	1.5%	3.00 [0.12, 73.63]
**Subtotal (95% CI)**		**1204**		**1205**	**1.5%**	**3.00 [0.12, 73.63]**
Total events	1		0			
Heterogeneity: Not applicable						
Test for overall effect: Z = 0.67 (P = 0.50)						
**Total (95% CI)**		**27543**		**26371**	**100.0%**	**1.03 [0.70, 1.52]**
Total events	52		48			
Heterogeneity: $Tau^2 = 0.00$; $Chi^2 = 4.06$, df = 8 (P = 0.85); $I^2 = 0\%$						
Test for overall effect: Z = 0.15 (P = 0.88)						
Test for subgroup differences: $Chi^2 = 4.00$, df = 7 (P = 0.78), $I^2 = 0\%$						

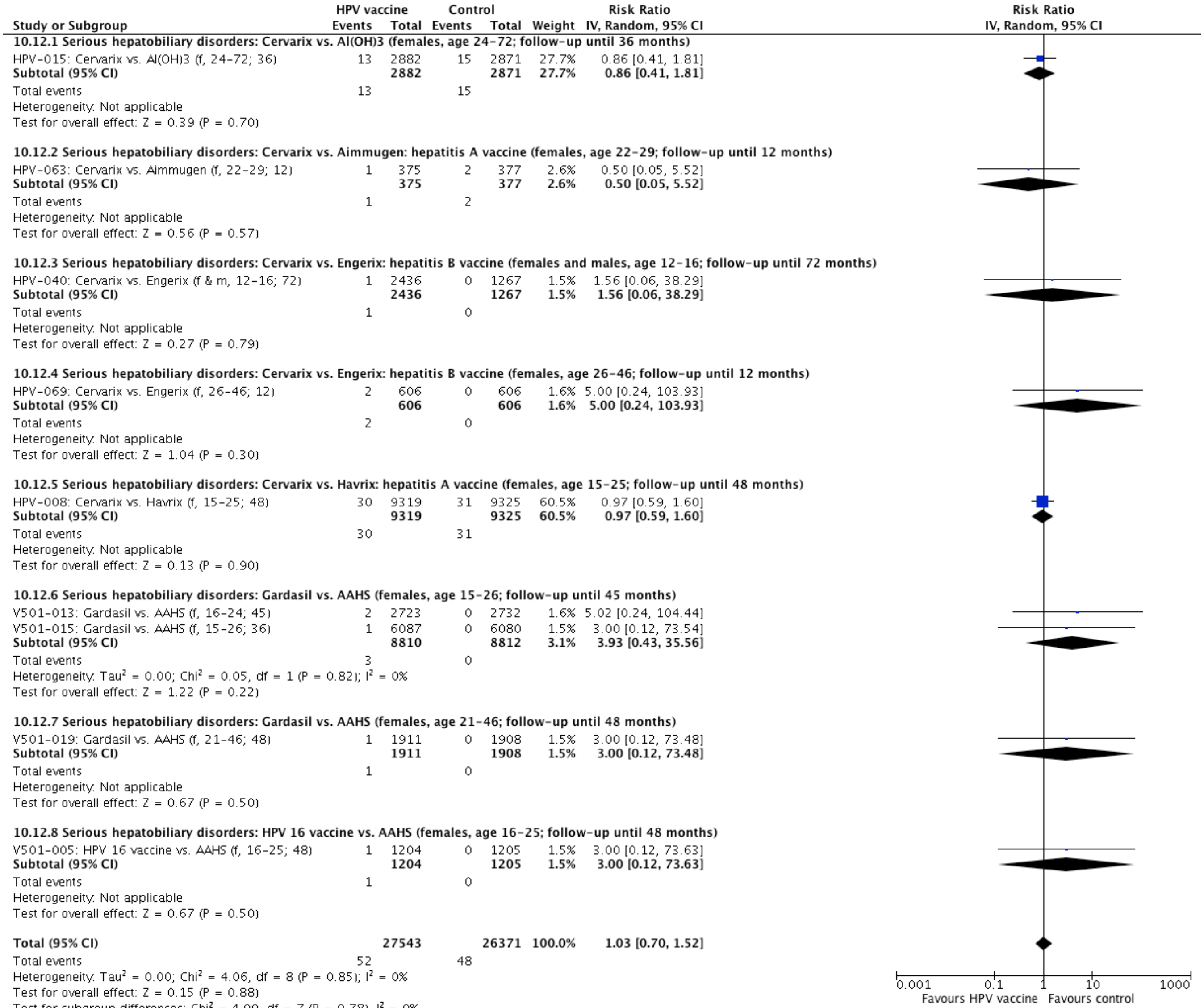

*10.12. Risk ratio for GlaxoSmithKline studies (i.e., HPV-0xx): 1.14 [0.75, 1.74]; risk ratio for Merck Sharp & Dohme studies (i.e., V50x-xxx): 3.44 [0.71, 16.68].

10.13. Serious harms reported within the MedDRA system organ class 'immune system disorders (10021428)'*: intention to treat analysis

Study or Subgroup	HPV vaccine Events	HPV vaccine Total	Control Events	Control Total	Weight	Risk Ratio IV, Random, 95% CI
**10.13.1 Serious immune system disorders: Cervarix vs. Al(OH)3 (females, age 24-72; follow-up until 36 months)**						
HPV-015: Cervarix vs. Al(OH)3 (f, 24-72; 36)	2	2882	5	2871	26.2%	0.40 [0.08, 2.05]
**Subtotal (95% CI)**		**2882**		**2871**	**26.2%**	**0.40 [0.08, 2.05]**
Total events	2		5			
Heterogeneity: Not applicable						
Test for overall effect: Z = 1.10 (P = 0.27)						
**10.13.2 Serious immune system disorders: Cervarix vs. Havrix: hepatitis A vaccine (females, age 15-25; follow-up until 48 months)**						
HPV-008: Cervarix vs. Havrix (f, 15-25; 48)	6	9319	7	9325	59.3%	0.86 [0.29, 2.55]
**Subtotal (95% CI)**		**9319**		**9325**	**59.3%**	**0.86 [0.29, 2.55]**
Total events	6		7			
Heterogeneity: Not applicable						
Test for overall effect: Z = 0.28 (P = 0.78)						
**10.13.3 Serious immune system disorders: Gardasil vs. AAHS (females, age 15-26; follow-up until 36 months)**						
V501-015: Gardasil vs. AAHS (f, 15-26; 36)	0	6087	2	6080	7.6%	0.20 [0.01, 4.16]
**Subtotal (95% CI)**		**6087**		**6080**	**7.6%**	**0.20 [0.01, 4.16]**
Total events	0		2			
Heterogeneity: Not applicable						
Test for overall effect: Z = 1.04 (P = 0.30)						
**10.13.4 Serious immune system disorders: Gardasil vs. AAHS (males, age 15-27; follow-up until 36 months)**						
V501-020: Gardasil vs. AAHS (m, 15-27; 36)	1	2032	0	2033	6.9%	3.00 [0.12, 73.64]
**Subtotal (95% CI)**		**2032**		**2033**	**6.9%**	**3.00 [0.12, 73.64]**
Total events	1		0			
Heterogeneity: Not applicable						
Test for overall effect: Z = 0.67 (P = 0.50)						
**Total (95% CI)**		**20320**		**20309**	**100.0%**	**0.68 [0.30, 1.58]**
Total events	9		14			
Heterogeneity: $Tau^2 = 0.00$; $Chi^2 = 2.03$, df = 3 (P = 0.57); $I^2 = 0\%$						
Test for overall effect: Z = 0.89 (P = 0.38)						
Test for subgroup differences: $Chi^2 = 2.03$, df = 3 (P = 0.57), $I^2 = 0\%$						

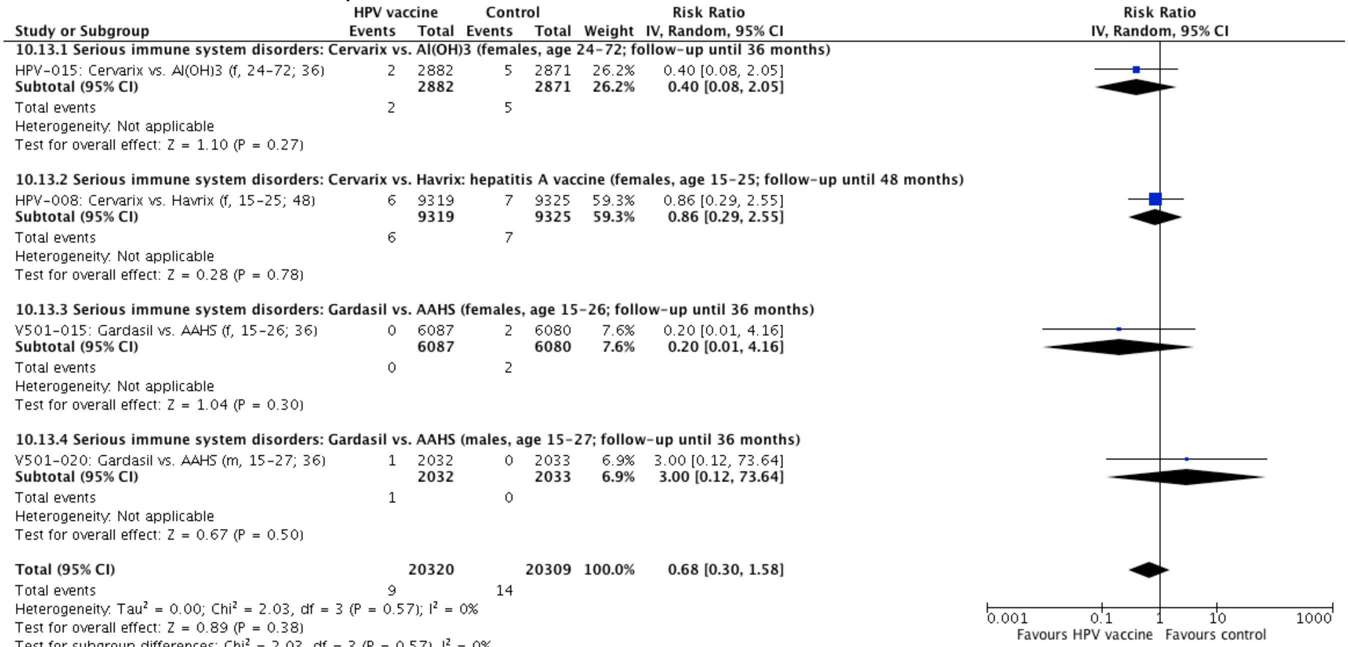

*10.13. Risk ratio for GlaxoSmithKline studies (i.e., HPV-0xx): 0.68 [0.27, 1.68]; risk ratio for Merck Sharp & Dohme studies (i.e., V50x-xxx): 0.74 [0.05, 10.47].

10.14. Serious harms reported within the MedDRA system organ class 'infections and infestations (10021881)'*: intention to treat analysis

Study or Subgroup	HPV vaccine Events	HPV vaccine Total	Control Events	Control Total	Weight	Risk Ratio IV, Random, 95% CI
**10.14.1 Serious infections and infestations: Cervarix vs. Al(OH)3 (females, age 9–17; follow-up until 12 months)**						
HPV-058: Cervarix vs. Al(OH)3 (f, 9–17; 12)	1	374	0	376	0.7%	3.02 [0.12, 73.80]
**Subtotal (95% CI)**		**374**		**376**	**0.7%**	**3.02 [0.12, 73.80]**
Total events	1		0			
Heterogeneity: Not applicable						
Test for overall effect: Z = 0.68 (P = 0.50)						
**10.14.2 Serious infections and infestations: Cervarix vs. Al(OH)3 (females, age 15–25; follow-up until 7 months)**						
HPV-038: Cervarix vs. Al(OH)3 (f, 15–25; 7)	1	149	1	76	0.9%	0.51 [0.03, 8.04]
**Subtotal (95% CI)**		**149**		**76**	**0.9%**	**0.51 [0.03, 8.04]**
Total events	1		1			
Heterogeneity: Not applicable						
Test for overall effect: Z = 0.48 (P = 0.63)						
**10.14.3 Serious infections and infestations: Cervarix vs. Al(OH)3 (females, age 18–35; follow-up until 7 months)**						
HPV-031: Cervarix vs. Al(OH)3 (f, 18–35; 7)	2	176	0	178	0.8%	5.06 [0.24, 104.58]
HPV-035: Cervarix vs. Al(OH)3 (f, 18–35; 7)	0	150	1	150	0.7%	0.33 [0.01, 8.12]
**Subtotal (95% CI)**		**326**		**328**	**1.5%**	**1.36 [0.10, 19.55]**
Total events	2		1			
Heterogeneity: $Tau^2 = 1.18$; $Chi^2 = 1.47$, df = 1 (P = 0.23); $I^2 = 32\%$						
Test for overall effect: Z = 0.23 (P = 0.82)						
**10.14.4 Serious infections and infestations: Cervarix vs. Al(OH)3 (females, age 24–72; follow-up until 36 months)**						
HPV-015: Cervarix vs. Al(OH)3 (f, 24–72; 36)	66	2882	50	2871	23.2%	1.31 [0.91, 1.89]
**Subtotal (95% CI)**		**2882**		**2871**	**23.2%**	**1.31 [0.91, 1.89]**
Total events	66		50			
Heterogeneity: Not applicable						
Test for overall effect: Z = 1.48 (P = 0.14)						
**10.14.5 Serious infections and infestations: Cervarix vs. Aimmugen: hepatitis A vaccine (females, age 22–29; follow-up until 12 months)**						
HPV-063: Cervarix vs. Aimmugen (f, 22–29; 12)	2	375	9	377	2.9%	0.22 [0.05, 1.03]
**Subtotal (95% CI)**		**375**		**377**	**2.9%**	**0.22 [0.05, 1.03]**
Total events	2		9			
Heterogeneity: Not applicable						
Test for overall effect: Z = 1.93 (P = 0.05)						
**10.14.6 Serious infections and infestations: Cervarix vs. Engerix: hepatitis B vaccine (females and males, age 12–16; follow-up until 72 months)**						
HPV-040: Cervarix vs. Engerix (f & m, 12–16; 72)	19	2436	10	1267	9.5%	0.99 [0.46, 2.12]
**Subtotal (95% CI)**		**2436**		**1267**	**9.5%**	**0.99 [0.46, 2.12]**
Total events	19		10			
Heterogeneity: Not applicable						
Test for overall effect: Z = 0.03 (P = 0.98)						
**10.14.7 Serious infections and infestations: Cervarix vs. Engerix: hepatitis B vaccine (females, age 26–46; follow-up until 12 months)**						
HPV-069: Cervarix vs. Engerix (f, 26–46; 12)	0	606	1	606	0.7%	0.33 [0.01, 8.17]
**Subtotal (95% CI)**		**606**		**606**	**0.7%**	**0.33 [0.01, 8.17]**
Total events	0		1			
Heterogeneity: Not applicable						
Test for overall effect: Z = 0.67 (P = 0.50)						
**10.14.8 Serious infections and infestations: Cervarix vs. Havrix: hepatitis A vaccine (females, age 9–15; follow-up until 12 months)**						
HPV-013: Cervarix vs. Havrix (f, 10–14; 12)	6	1035	7	1032	5.3%	0.85 [0.29, 2.53]
HPV-033: Cervarix vs. Havrix (f, 9–15; 7)	0	160	1	161	0.7%	0.34 [0.01, 8.17]
**Subtotal (95% CI)**		**1195**		**1193**	**6.0%**	**0.78 [0.28, 2.17]**
Total events	6		8			
Heterogeneity: $Tau^2 = 0.00$; $Chi^2 = 0.30$, df = 1 (P = 0.59); $I^2 = 0\%$						
Test for overall effect: Z = 0.48 (P = 0.63)						
**10.14.9 Serious infections and infestations: Cervarix vs. Havrix: hepatitis A vaccine (females, age 15–25; follow-up until 48 months)**						
HPV-008: Cervarix vs. Havrix (f, 15–25; 48)	268	9319	260	9325	35.0%	1.03 [0.87, 1.22]
**Subtotal (95% CI)**		**9319**		**9325**	**35.0%**	**1.03 [0.87, 1.22]**
Total events	268		260			
Heterogeneity: Not applicable						
Test for overall effect: Z = 0.36 (P = 0.72)						
**10.14.10 Serious infections and infestations: Cervarix vs. Twinrix: hepatitis A and B vaccine (females, age 8–15; follow-up until 12 months)**						
HPV-029: Cervarix vs. Twinrix (f, 8–15; 12)	1	270	0	271	0.7%	3.01 [0.12, 73.59]
**Subtotal (95% CI)**		**270**		**271**	**0.7%**	**3.01 [0.12, 73.59]**
Total events	1		0			
Heterogeneity: Not applicable						
Test for overall effect: Z = 0.68 (P = 0.50)						
**10.14.11 Serious infections and infestations: Gardasil vs. carrier solution (females and males, age 9–16; follow-up until 18 months)**						
V501-018: Gardasil vs. carrier solu. (f&m,9–16;18)	2	1184	0	597	0.8%	2.52 [0.12, 52.47]
**Subtotal (95% CI)**		**1184**		**597**	**0.8%**	**2.52 [0.12, 52.47]**
Total events	2		0			
Heterogeneity: Not applicable						
Test for overall effect: Z = 0.60 (P = 0.55)						
**10.14.12 Serious infections and infestations: Gardasil vs. AAHS (females, age 15–26; follow-up until 45 months)**						
V501-013: Gardasil vs. AAHS (f, 16–24; 45)	11	2723	2	2732	3.0%	5.52 [1.22, 24.87]
V501-015: Gardasil vs. AAHS (f, 15–26; 36)	12	6087	10	6080	8.2%	1.20 [0.52, 2.77]
**Subtotal (95% CI)**		**8810**		**8812**	**11.1%**	**2.25 [0.52, 9.82]**
Total events	23		12			
Heterogeneity: $Tau^2 = 0.78$; $Chi^2 = 3.02$, df = 1 (P = 0.08); $I^2 = 67\%$						
Test for overall effect: Z = 1.08 (P = 0.28)						
**10.14.13 Serious infections and infestations: Gardasil vs. AAHS (females, age 21–46; follow-up until 48 months)**						
V501-019: Gardasil vs. AAHS (f, 21–46; 48)	3	1911	10	1908	3.9%	0.30 [0.08, 1.09]
**Subtotal (95% CI)**		**1911**		**1908**	**3.9%**	**0.30 [0.08, 1.09]**
Total events	3		10			
Heterogeneity: Not applicable						
Test for overall effect: Z = 1.83 (P = 0.07)						
**10.14.14 Serious infections and infestations: Gardasil vs. AAHS (males, age 15–27; follow-up until 36 months)**						
V501-020: Gardasil vs. AAHS (m, 15–27; 36)	3	2032	0	2033	0.8%	7.00 [0.36, 135.50]
**Subtotal (95% CI)**		**2032**		**2033**	**0.8%**	**7.00 [0.36, 135.50]**
Total events	3		0			
Heterogeneity: Not applicable						
Test for overall effect: Z = 1.29 (P = 0.20)						
**10.14.15 Serious infections and infestations: Gardasil 9 vs. saline placebo (females, age 12–26; follow-up until 7 months)**						
V503-006: Gardasil 9 vs. saline (f, 12–26;7)	2	618	0	306	0.8%	2.48 [0.12, 51.49]
**Subtotal (95% CI)**		**618**		**306**	**0.8%**	**2.48 [0.12, 51.49]**
Total events	2		0			
Heterogeneity: Not applicable						
Test for overall effect: Z = 0.59 (P = 0.56)						
**10.14.16 Serious infections and infestations: HPV 16 vaccine vs. AAHS (females, age 16–25; follow-up until 48 months)**						
V501-005: HPV 16 vaccine vs. AAHS (f, 16–25; 48)	1	1204	5	1205	1.5%	0.20 [0.02, 1.71]
**Subtotal (95% CI)**		**1204**		**1205**	**1.5%**	**0.20 [0.02, 1.71]**
Total events	1		5			
Heterogeneity: Not applicable						
Test for overall effect: Z = 1.47 (P = 0.14)						
**Total (95% CI)**		**33691**		**31551**	**100.0%**	**1.04 [0.80, 1.37]**
Total events	400		367			
Heterogeneity: $Tau^2 = 0.05$; $Chi^2 = 22.13$, df = 18 (P = 0.23); $I^2 = 19\%$						
Test for overall effect: Z = 0.32 (P = 0.75)						
Test for subgroup differences: $Chi^2 = 16.61$, df = 15 (P = 0.34), $I^2 = 9.7\%$						

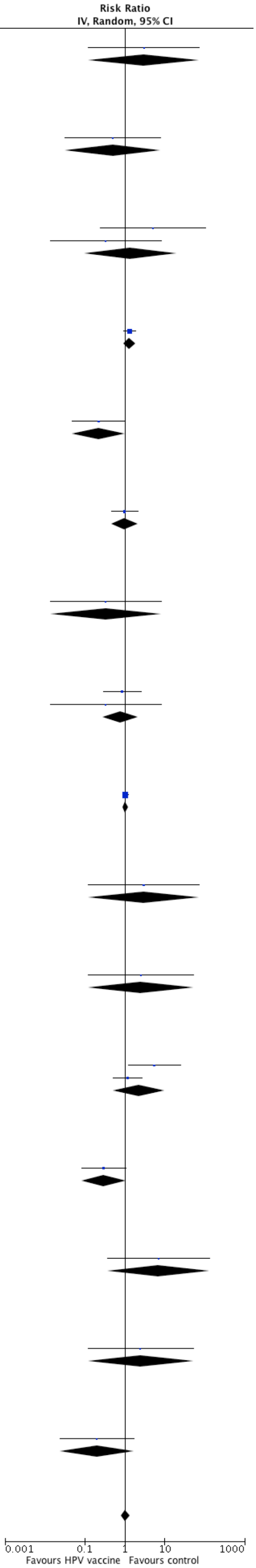

*10.14. Risk ratio for GlaxoSmithKline studies (i.e., HPV-0xx): 1.05 [0.91, 1.22]; risk ratio for Merck Sharp & Dohme studies (i.e., V50x-xxx): 1.23 [0.46, 3.33].

10.15. Serious harms reported within the MedDRA system organ class 'injury poisoning and procedural complications (10022117)'*: intention to treat analysis

Study or Subgroup	HPV vaccine Events	HPV vaccine Total	Control Events	Control Total	Weight	Risk Ratio IV, Random, 95% CI
**10.15.1 Serious injury, poisoning and procedural complications: Cervarix vs. Al(OH)3 (females, age 24-72; follow-up until 36 months)**						
HPV-015: Cervarix vs. Al(OH)3 (f, 24-72; 36)	25	2882	25	2871	16.4%	1.00 [0.57, 1.73]
**Subtotal (95% CI)**		**2882**		**2871**	**16.4%**	**1.00 [0.57, 1.73]**
Total events	25		25			
Heterogeneity: Not applicable						
Test for overall effect: Z = 0.01 (P = 0.99)						
**10.15.2 Serious injury, poisoning and procedural complications: Cervarix vs. Aimmugen: hepatitis A vaccine (females, age 22-29; follow-up until 12 months)**						
HPV-063: Cervarix vs. Aimmugen (f, 22-29; 12)	5	375	3	377	3.6%	1.68 [0.40, 6.96]
**Subtotal (95% CI)**		**375**		**377**	**3.6%**	**1.68 [0.40, 6.96]**
Total events	5		3			
Heterogeneity: Not applicable						
Test for overall effect: Z = 0.71 (P = 0.48)						
**10.15.3 Serious injury, poisoning and procedural complications: Cervarix vs. Engerix: hepatitis B vaccine (females and males, age 12-16; follow-up until 72 months)**						
HPV-040: Cervarix vs. Engerix (f & m, 12-16; 72)	21	2436	9	1267	10.1%	1.21 [0.56, 2.64]
**Subtotal (95% CI)**		**2436**		**1267**	**10.1%**	**1.21 [0.56, 2.64]**
Total events	21		9			
Heterogeneity: Not applicable						
Test for overall effect: Z = 0.49 (P = 0.63)						
**10.15.4 Serious injury, poisoning and procedural complications: Cervarix vs. Havrix: hepatitis A vaccine (females, age 10-14; follow-up until 12 months)**						
HPV-013: Cervarix vs. Havrix (f, 10-14; 12)	4	1035	1	1032	1.6%	3.99 [0.45, 35.62]
**Subtotal (95% CI)**		**1035**		**1032**	**1.6%**	**3.99 [0.45, 35.62]**
Total events	4		1			
Heterogeneity: Not applicable						
Test for overall effect: Z = 1.24 (P = 0.22)						
**10.15.5 Serious injury, poisoning and procedural complications: Cervarix vs. Havrix: hepatitis A vaccine (females, age 15-25; follow-up until 48 months)**						
HPV-008: Cervarix vs. Havrix (f, 15-25; 48)	104	9319	120	9325	32.0%	0.87 [0.67, 1.13]
**Subtotal (95% CI)**		**9319**		**9325**	**32.0%**	**0.87 [0.67, 1.13]**
Total events	104		120			
Heterogeneity: Not applicable						
Test for overall effect: Z = 1.07 (P = 0.28)						
**10.15.6 Serious injury, poisoning and procedural complications: Cervarix vs. Twinrix: hepatitis A and B vaccine (females, age 8-15; follow-up until 12 months)**						
HPV-029: Cervarix vs. Twinrix (f, 8-15; 12)	1	270	3	271	1.5%	0.33 [0.04, 3.20]
**Subtotal (95% CI)**		**270**		**271**	**1.5%**	**0.33 [0.04, 3.20]**
Total events	1		3			
Heterogeneity: Not applicable						
Test for overall effect: Z = 0.95 (P = 0.34)						
**10.15.7 Serious injury, poisoning and procedural complications: Gardasil vs. AAHS (females, age 15-26; follow-up until 45 months)**						
V501-013: Gardasil vs. AAHS (f, 16-24; 45)	21	2723	33	2732	16.7%	0.64 [0.37, 1.10]
V501-015: Gardasil vs. AAHS (f, 15-26; 36)	9	6087	5	6080	5.8%	1.80 [0.60, 5.36]
**Subtotal (95% CI)**		**8810**		**8812**	**22.5%**	**0.96 [0.36, 2.58]**
Total events	30		38			
Heterogeneity: Tau² = 0.34; Chi² = 2.76, df = 1 (P = 0.10); I² = 64%						
Test for overall effect: Z = 0.09 (P = 0.93)						
**10.15.8 Serious injury, poisoning and procedural complications: Gardasil vs. AAHS (females, age 21-46; follow-up until 48 months)**						
V501-019: Gardasil vs. AAHS (f, 21-46; 48)	1	1911	1	1908	1.0%	1.00 [0.06, 15.95]
**Subtotal (95% CI)**		**1911**		**1908**	**1.0%**	**1.00 [0.06, 15.95]**
Total events	1		1			
Heterogeneity: Not applicable						
Test for overall effect: Z = 0.00 (P = 1.00)						
**10.15.9 Serious injury, poisoning and procedural complications: Gardasil vs. AAHS (males, age 15-27; follow-up until 36 months)**						
V501-020: Gardasil vs. AAHS (m, 15-27; 36)	5	2032	9	2033	5.8%	0.56 [0.19, 1.66]
**Subtotal (95% CI)**		**2032**		**2033**	**5.8%**	**0.56 [0.19, 1.66]**
Total events	5		9			
Heterogeneity: Not applicable						
Test for overall effect: Z = 1.05 (P = 0.29)						
**10.15.10 Serious injury, poisoning and procedural complications: Gardasil 9 vs. saline placebo (females, age 12-26; follow-up until 7 months)**						
V503-006: Gardasil 9 vs. saline (f, 12-26;7)	0	618	2	306	0.9%	0.10 [0.00, 2.06]
**Subtotal (95% CI)**		**618**		**306**	**0.9%**	**0.10 [0.00, 2.06]**
Total events	0		2			
Heterogeneity: Not applicable						
Test for overall effect: Z = 1.49 (P = 0.14)						
**10.15.11 Serious injury, poisoning and procedural complications: HPV 16 vaccine vs. AAHS (females, age 16-25; follow-up until 48 months)**						
V501-005: HPV 16 vaccine vs. AAHS (f, 16-25; 48)	3	1204	13	1205	4.6%	0.23 [0.07, 0.81]
**Subtotal (95% CI)**		**1204**		**1205**	**4.6%**	**0.23 [0.07, 0.81]**
Total events	3		13			
Heterogeneity: Not applicable						
Test for overall effect: Z = 2.29 (P = 0.02)						
**Total (95% CI)**		**30892**		**29407**	**100.0%**	**0.85 [0.64, 1.13]**
Total events	199		224			
Heterogeneity: Tau² = 0.05; Chi² = 14.13, df = 11 (P = 0.23); I² = 22%						
Test for overall effect: Z = 1.14 (P = 0.26)						
Test for subgroup differences: Chi² = 11.26, df = 10 (P = 0.34), I² = 11.2%						

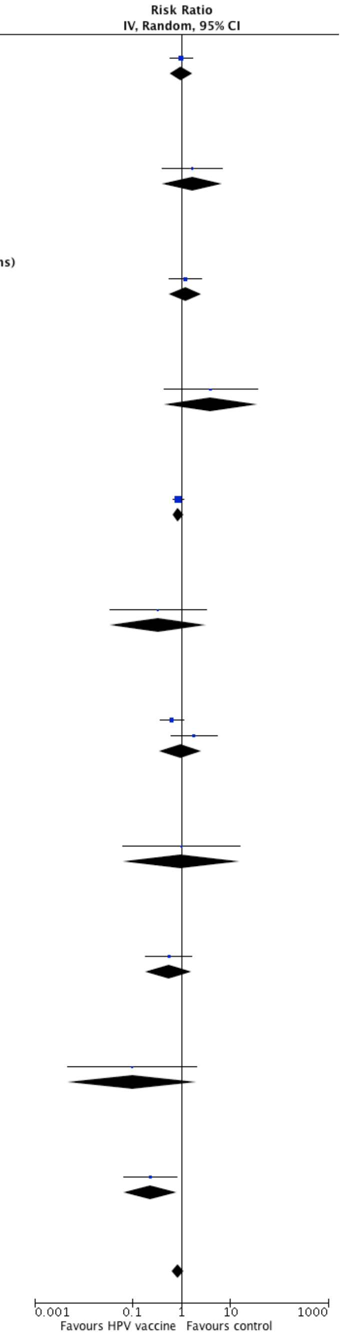

*10.15. Risk ratio for GlaxoSmithKline studies (i.e., HPV-0xx): 0.93 [0.75, 1.16]; risk ratio for Merck Sharp & Dohme studies (i.e., V50x-xxx): 0.61 [0.33, 1.12].

10.16. Serious harms reported within the MedDRA system organ class 'investigations (10022891)'*: intention to treat analysis

Study or Subgroup	HPV vaccine Events	HPV vaccine Total	Control Events	Control Total	Weight	Risk Ratio IV, Random, 95% CI
**10.16.1 Serious investigations: Cervarix vs. Aimmugen: hepatitis A vaccine (females, age 22-29; follow-up until 12 months)**						
HPV-063: Cervarix vs. Aimmugen (f, 22-29; 12)	1	375	0	377	33.4%	3.02 [0.12, 73.80]
**Subtotal (95% CI)**		**375**		**377**	**33.4%**	**3.02 [0.12, 73.80]**
Total events	1		0			
Heterogeneity: Not applicable						
Test for overall effect: Z = 0.68 (P = 0.50)						
**10.16.2 Serious investigations: Cervarix vs. Havrix: hepatitis A vaccine (females, age 10-14; follow-up until 12 months)**						
HPV-013: Cervarix vs. Havrix (f, 10-14; 12)	0	1035	1	1032	33.3%	0.33 [0.01, 8.15]
**Subtotal (95% CI)**		**1035**		**1032**	**33.3%**	**0.33 [0.01, 8.15]**
Total events	0		1			
Heterogeneity: Not applicable						
Test for overall effect: Z = 0.67 (P = 0.50)						
**10.16.3 Serious investigations: Cervarix vs. Havrix: hepatitis A vaccine (females, age 15-25; follow-up until 48 months)**						
HPV-008: Cervarix vs. Havrix (f, 15-25; 48)	0	9319	1	9325	33.3%	0.33 [0.01, 8.19]
**Subtotal (95% CI)**		**9319**		**9325**	**33.3%**	**0.33 [0.01, 8.19]**
Total events	0		1			
Heterogeneity: Not applicable						
Test for overall effect: Z = 0.67 (P = 0.50)						
**Total (95% CI)**		**10729**		**10734**	**100.0%**	**0.69 [0.11, 4.40]**
Total events	1		2			
Heterogeneity: Tau² = 0.00; Chi² = 1.22, df = 2 (P = 0.54); I² = 0%						
Test for overall effect: Z = 0.39 (P = 0.70)						
Test for subgroup differences: Chi² = 1.22, df = 2 (P = 0.54), I² = 0%						

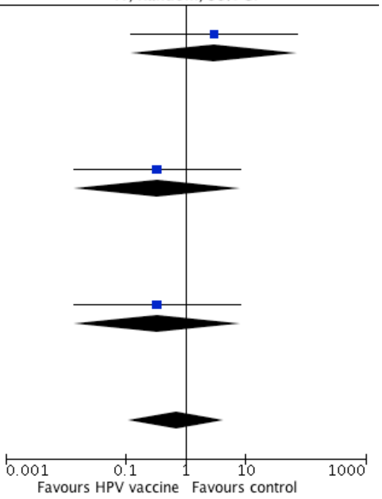

*10.16. Risk ratio for GlaxoSmithKline studies (i.e., HPV-0xx): 0.69 [0.11, 4.40]; risk ratio for Merck Sharp & Dohme studies (i.e., V50x-xxx): not applicable.

10.17. Serious harms reported within the MedDRA system organ class 'metabolism and nutrition disorders (10027433)'*: intention to treat analysis

Study or Subgroup	HPV vaccine Events	HPV vaccine Total	Control Events	Control Total	Weight	Risk Ratio IV, Random, 95% CI
**10.17.1 Serious metabolism and nutrition disorders: Cervarix vs. Al(OH)3 (females, age 24-72; follow-up until 36 months)**						
HPV-015: Cervarix vs. Al(OH)3 (f, 24-72; 36)	4	2882	4	2871	25.1%	1.00 [0.25, 3.98]
**Subtotal (95% CI)**		**2882**		**2871**	**25.1%**	**1.00 [0.25, 3.98]**
Total events	4		4			
Heterogeneity: Not applicable						
Test for overall effect: Z = 0.01 (P = 1.00)						
**10.17.2 Serious metabolism and nutrition disorders: Cervarix vs. Engerix: hepatitis B vaccine (females and males, age 12-16; follow-up until 72 months)**						
HPV-040: Cervarix vs. Engerix (f & m, 12-16; 72)	1	2436	1	1267	6.3%	0.52 [0.03, 8.31]
**Subtotal (95% CI)**		**2436**		**1267**	**6.3%**	**0.52 [0.03, 8.31]**
Total events	1		1			
Heterogeneity: Not applicable						
Test for overall effect: Z = 0.46 (P = 0.64)						
**10.17.3 Serious metabolism and nutrition disorders: Cervarix vs. Havrix: hepatitis A vaccine (females, age 10-14; follow-up until 12 months)**						
HPV-013: Cervarix vs. Havrix (f, 10-14; 12)	1	1035	0	1032	4.7%	2.99 [0.12, 73.34]
**Subtotal (95% CI)**		**1035**		**1032**	**4.7%**	**2.99 [0.12, 73.34]**
Total events	1		0			
Heterogeneity: Not applicable						
Test for overall effect: Z = 0.67 (P = 0.50)						
**10.17.4 Serious metabolism and nutrition disorders: Cervarix vs. Havrix: hepatitis A vaccine (females, age 15-25; follow-up until 48 months)**						
HPV-008: Cervarix vs. Havrix (f, 15-25; 48)	9	9319	6	9325	45.1%	1.50 [0.53, 4.22]
**Subtotal (95% CI)**		**9319**		**9325**	**45.1%**	**1.50 [0.53, 4.22]**
Total events	9		6			
Heterogeneity: Not applicable						
Test for overall effect: Z = 0.77 (P = 0.44)						
**10.17.5 Serious metabolism and nutrition disorders: Gardasil vs. carrier solution (females and males, age 9-16; follow-up until 18 months)**						
V501-018: Gardasil vs. carrier solu. (f&m,9-16;18)	1	1184	0	597	4.7%	1.51 [0.06, 37.11]
**Subtotal (95% CI)**		**1184**		**597**	**4.7%**	**1.51 [0.06, 37.11]**
Total events	1		0			
Heterogeneity: Not applicable						
Test for overall effect: Z = 0.25 (P = 0.80)						
**10.17.6 Serious metabolism and nutrition disorders: Gardasil vs. AAHS (females, age 16-24; follow-up until 45 months)**						
V501-013: Gardasil vs. AAHS (f, 16-24; 45)	1	2723	0	2732	4.7%	3.01 [0.12, 73.85]
**Subtotal (95% CI)**		**2723**		**2732**	**4.7%**	**3.01 [0.12, 73.85]**
Total events	1		0			
Heterogeneity: Not applicable						
Test for overall effect: Z = 0.67 (P = 0.50)						
**10.17.7 Serious metabolism and nutrition disorders: Gardasil vs. AAHS (females, age 21-46; follow-up until 48 months)**						
V501-019: Gardasil vs. AAHS (f, 21-46; 48)	1	1911	0	1908	4.7%	3.00 [0.12, 73.48]
**Subtotal (95% CI)**		**1911**		**1908**	**4.7%**	**3.00 [0.12, 73.48]**
Total events	1		0			
Heterogeneity: Not applicable						
Test for overall effect: Z = 0.67 (P = 0.50)						
**10.17.8 Serious metabolism and nutrition disorders: HPV 16 vaccine vs. AAHS (females, age 16-25; follow-up until 48 months)**						
V501-005: HPV 16 vaccine vs. AAHS (f, 16-25; 48)	1	1204	0	1205	4.7%	3.00 [0.12, 73.63]
**Subtotal (95% CI)**		**1204**		**1205**	**4.7%**	**3.00 [0.12, 73.63]**
Total events	1		0			
Heterogeneity: Not applicable						
Test for overall effect: Z = 0.67 (P = 0.50)						
**Total (95% CI)**		**22694**		**20937**	**100.0%**	**1.44 [0.72, 2.89]**
Total events	19		11			
Heterogeneity: $Tau^2 = 0.00$; $Chi^2 = 1.61$, df = 7 (P = 0.98); $I^2 = 0\%$						
Test for overall effect: Z = 1.04 (P = 0.30)						
Test for subgroup differences: $Chi^2 = 1.61$, df = 7 (P = 0.98), $I^2 = 0\%$						

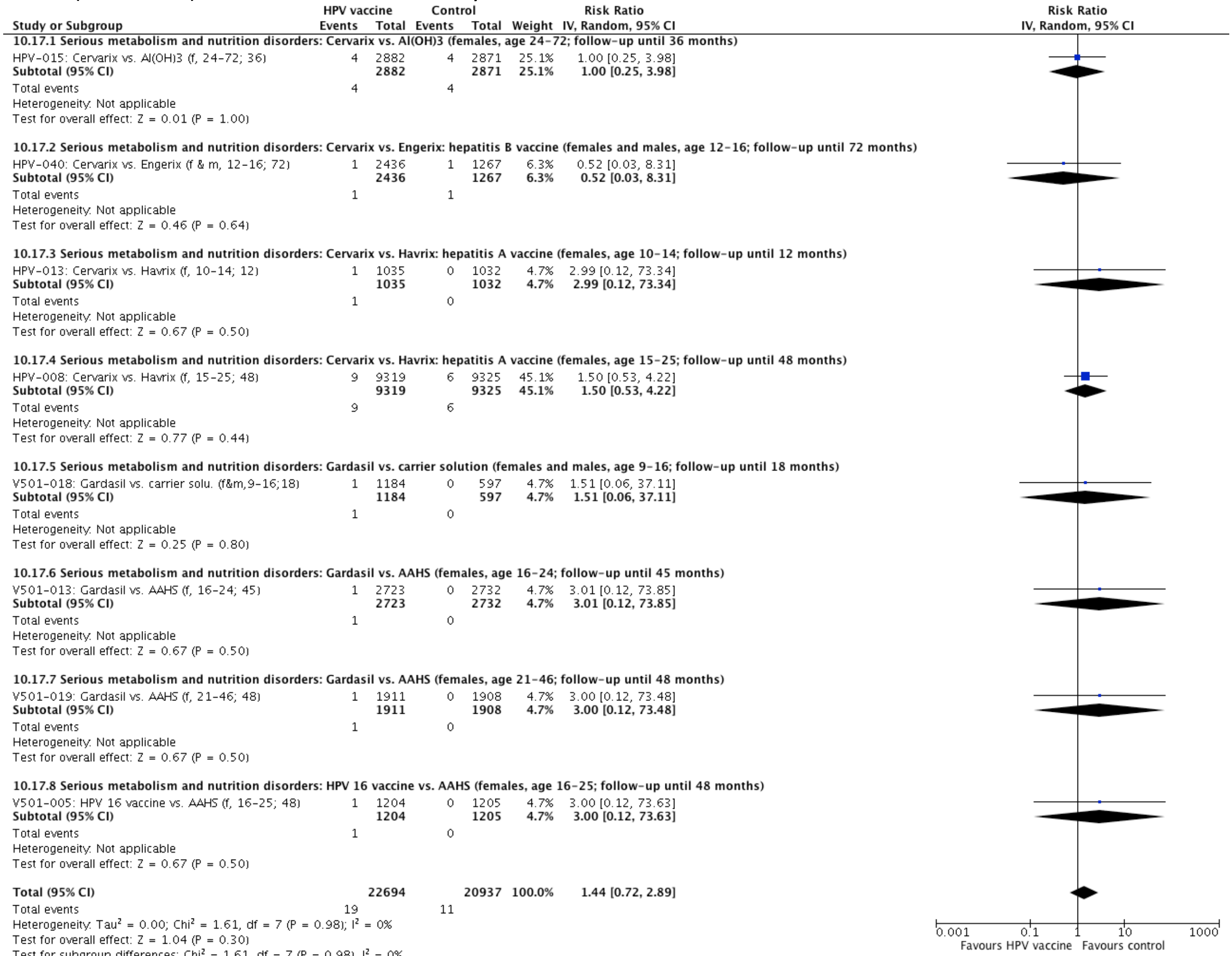

*10.17. Risk ratio for GlaxoSmithKline studies (i.e., HPV-0xx): 1.27 [0.59, 2.74]; risk ratio for Merck Sharp & Dohme studies (i.e., V50x-xxx): 2.50 [0.51, 12.40].

10.18. Serious harms reported within the MedDRA system organ class 'musculoskeletal and connective tissue disorders (10028395)'*: intention to treat analysis

Study or Subgroup	HPV vaccine Events	HPV vaccine Total	Control Events	Control Total	Weight	Risk Ratio IV, Random, 95% CI
**10.18.1 Serious musculoskeletal and connective disorders: Cervarix vs. Al(OH)3 (females, age 24-72; follow-up until 36 months)**						
HPV-015: Cervarix vs. Al(OH)3 (f, 24-72; 36)	9	2882	13	2871	32.3%	0.69 [0.30, 1.61]
**Subtotal (95% CI)**		**2882**		**2871**	**32.3%**	**0.69 [0.30, 1.61]**
Total events	9		13			
Heterogeneity: Not applicable						
Test for overall effect: Z = 0.86 (P = 0.39)						
**10.18.2 Serious musculoskeletal and connective disorders: Cervarix vs. Aimmugen: hepatitis A vaccine (females, age 22-29; follow-up until 12 months)**						
HPV-063: Cervarix vs. Aimmugen (f, 22-29; 12)	0	375	1	377	2.3%	0.34 [0.01, 8.20]
**Subtotal (95% CI)**		**375**		**377**	**2.3%**	**0.34 [0.01, 8.20]**
Total events	0		1			
Heterogeneity: Not applicable						
Test for overall effect: Z = 0.67 (P = 0.50)						
**10.18.3 Serious musculoskeletal and connective disorders: Cervarix vs. Engerix: hepatitis B vaccine (females and males, age 12-16; follow-up until 72 months)**						
HPV-040: Cervarix vs. Engerix (f & m, 12-16; 72)	2	2436	0	1267	2.5%	2.60 [0.12, 54.15]
**Subtotal (95% CI)**		**2436**		**1267**	**2.5%**	**2.60 [0.12, 54.15]**
Total events	2		0			
Heterogeneity: Not applicable						
Test for overall effect: Z = 0.62 (P = 0.54)						
**10.18.4 Serious musculoskeletal and connective disorders: Cervarix vs. Havrix: hepatitis A vaccine (females, age 15-25; follow-up until 48 months)**						
HPV-008: Cervarix vs. Havrix (f, 15-25; 48)	21	9319	14	9325	50.9%	1.50 [0.76, 2.95]
**Subtotal (95% CI)**		**9319**		**9325**	**50.9%**	**1.50 [0.76, 2.95]**
Total events	21		14			
Heterogeneity: Not applicable						
Test for overall effect: Z = 1.18 (P = 0.24)						
**10.18.5 Serious musculoskeletal and connective disorders: Gardasil vs. carrier solution (females and males, age 9-16; follow-up until 18 months)**						
V501-018: Gardasil vs. carrier solu. (f&m,9-16;18)	1	1184	0	597	2.3%	1.51 [0.06, 37.11]
**Subtotal (95% CI)**		**1184**		**597**	**2.3%**	**1.51 [0.06, 37.11]**
Total events	1		0			
Heterogeneity: Not applicable						
Test for overall effect: Z = 0.25 (P = 0.80)						
**10.18.6 Serious musculoskeletal and connective disorders: Gardasil vs. AAHS (females, age 15-26; follow-up until 45 months)**						
V501-013: Gardasil vs. AAHS (f, 16-24; 45)	0	2723	1	2732	2.3%	0.33 [0.01, 8.21]
V501-015: Gardasil vs. AAHS (f, 15-26; 36)	0	6087	1	6080	2.3%	0.33 [0.01, 8.17]
**Subtotal (95% CI)**		**8810**		**8812**	**4.5%**	**0.33 [0.03, 3.21]**
Total events	0		2			
Heterogeneity: $Tau^2 = 0.00$; $Chi^2 = 0.00$, df = 1 (P = 1.00); $I^2 = 0\%$						
Test for overall effect: Z = 0.95 (P = 0.34)						
**10.18.7 Serious musculoskeletal and connective disorders: Gardasil vs. AAHS (females, age 21-46; follow-up until 48 months)**						
V501-019: Gardasil vs. AAHS (f, 21-46; 48)	1	1911	0	1908	2.3%	3.00 [0.12, 73.48]
**Subtotal (95% CI)**		**1911**		**1908**	**2.3%**	**3.00 [0.12, 73.48]**
Total events	1		0			
Heterogeneity: Not applicable						
Test for overall effect: Z = 0.67 (P = 0.50)						
**10.18.8 Serious musculoskeletal and connective disorders: HPV 16 vaccine vs. AAHS (females, age 16-25; follow-up until 48 months)**						
V501-005: HPV 16 vaccine vs. AAHS (f, 16-25; 48)	1	1204	1	1205	3.0%	1.00 [0.06, 15.98]
**Subtotal (95% CI)**		**1204**		**1205**	**3.0%**	**1.00 [0.06, 15.98]**
Total events	1		1			
Heterogeneity: Not applicable						
Test for overall effect: Z = 0.00 (P = 1.00)						
**Total (95% CI)**		**28121**		**26362**	**100.0%**	**1.07 [0.66, 1.74]**
Total events	35		31			
Heterogeneity: $Tau^2 = 0.00$; $Chi^2 = 4.29$, df = 8 (P = 0.83); $I^2 = 0\%$						
Test for overall effect: Z = 0.29 (P = 0.77)						
Test for subgroup differences: $Chi^2 = 4.29$, df = 7 (P = 0.75), $I^2 = 0\%$						

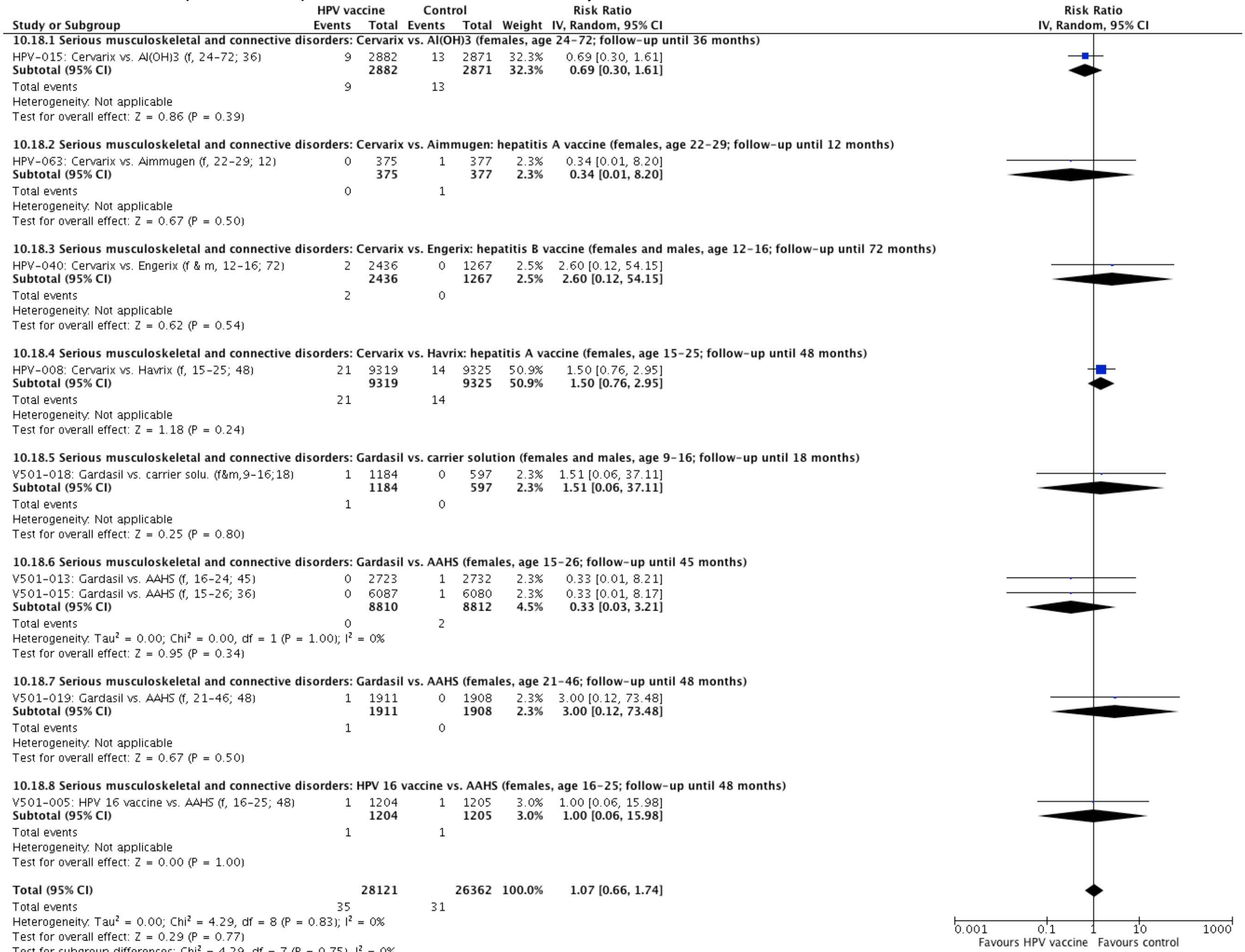

*10.18. Risk ratio for GlaxoSmithKline studies (i.e., HPV-0xx): 1.10 [0.66, 1.84]; risk ratio for Merck Sharp & Dohme studies (i.e., V50x-xxx): 0.87 [0.22, 3.49].

10.19. Serious harms reported within the MedDRA system organ class 'neoplasms benign, malignant and unspecified (incl. cysts and polyps) (10029104)'*: intention to treat analysis

Study or Subgroup	HPV vaccine Events	HPV vaccine Total	Control Events	Control Total	Weight	Risk Ratio IV, Random, 95% CI
**10.19.1 Serious neoplasms: Cervarix vs. Al(OH)3 (females, age 24-72; follow-up until 36 months)**						
HPV-015: Cervarix vs. Al(OH)3 (f, 24-72; 36)	39	2882	28	2871	53.8%	1.39 [0.86, 2.25]
**Subtotal (95% CI)**		**2882**		**2871**	**53.8%**	**1.39 [0.86, 2.25]**
Total events	39		28			
Heterogeneity: Not applicable						
Test for overall effect: Z = 1.33 (P = 0.18)						
**10.19.2 Serious neoplasms: Cervarix vs. Aimmugen: hepatitis A vaccine (females, age 22-29; follow-up until 12 months)**						
HPV-063: Cervarix vs. Aimmugen (f, 22-29; 12)	0	375	1	377	1.2%	0.34 [0.01, 8.20]
**Subtotal (95% CI)**		**375**		**377**	**1.2%**	**0.34 [0.01, 8.20]**
Total events	0		1			
Heterogeneity: Not applicable						
Test for overall effect: Z = 0.67 (P = 0.50)						
**10.19.3 Serious neoplasms: Cervarix vs. Engerix: hepatitis B vaccine (females and males, age 12-16; follow-up until 72 months)**						
HPV-040: Cervarix vs. Engerix (f & m, 12-16; 72)	0	2436	1	1267	1.2%	0.17 [0.01, 4.25]
**Subtotal (95% CI)**		**2436**		**1267**	**1.2%**	**0.17 [0.01, 4.25]**
Total events	0		1			
Heterogeneity: Not applicable						
Test for overall effect: Z = 1.07 (P = 0.28)						
**10.19.4 Serious neoplasms: Cervarix vs. Engerix: hepatitis B vaccine (females, age 26-46; follow-up until 12 months)**						
HPV-069: Cervarix vs. Engerix (f, 26-46; 12)	1	606	1	606	1.6%	1.00 [0.06, 15.95]
**Subtotal (95% CI)**		**606**		**606**	**1.6%**	**1.00 [0.06, 15.95]**
Total events	1		1			
Heterogeneity: Not applicable						
Test for overall effect: Z = 0.00 (P = 1.00)						
**10.19.5 Serious neoplasms: Cervarix vs. Havrix: hepatitis A vaccine (females, age 15-25; follow-up until 48 months)**						
HPV-008: Cervarix vs. Havrix (f, 15-25; 48)	22	9319	22	9325	36.0%	1.00 [0.55, 1.81]
**Subtotal (95% CI)**		**9319**		**9325**	**36.0%**	**1.00 [0.55, 1.81]**
Total events	22		22			
Heterogeneity: Not applicable						
Test for overall effect: Z = 0.00 (P = 1.00)						
**10.19.6 Serious neoplasms: Gardasil vs. AAHS (females, age 15-26; follow-up until 36 months)**						
V501-015: Gardasil vs. AAHS (f, 15-26; 36)	0	6087	1	6080	1.2%	0.33 [0.01, 8.17]
**Subtotal (95% CI)**		**6087**		**6080**	**1.2%**	**0.33 [0.01, 8.17]**
Total events	0		1			
Heterogeneity: Not applicable						
Test for overall effect: Z = 0.67 (P = 0.50)						
**10.19.7 Serious neoplasms: Gardasil vs. AAHS (females, age 21-46; follow-up until 48 months)**						
V501-019: Gardasil vs. AAHS (f, 21-46; 48)	6	1911	2	1908	4.9%	3.00 [0.61, 14.82]
**Subtotal (95% CI)**		**1911**		**1908**	**4.9%**	**3.00 [0.61, 14.82]**
Total events	6		2			
Heterogeneity: Not applicable						
Test for overall effect: Z = 1.34 (P = 0.18)						
**Total (95% CI)**		**23616**		**22434**	**100.0%**	**1.20 [0.84, 1.71]**
Total events	68		56			
Heterogeneity: Tau² = 0.00; Chi² = 4.62, df = 6 (P = 0.59); I² = 0%						
Test for overall effect: Z = 1.01 (P = 0.31)						
Test for subgroup differences: Chi² = 4.62, df = 6 (P = 0.59), I² = 0%						

Risk Ratio
IV, Random, 95% CI

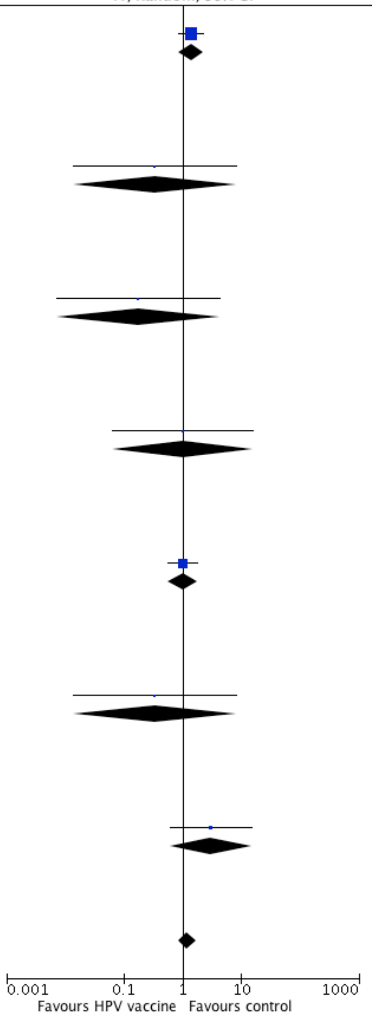

*10.19. Risk ratio for GlaxoSmithKline studies (i.e., HPV-0xx): 1.16 [0.81, 1.68]; risk ratio for Merck Sharp & Dohme studies (i.e., V50x-xxx): 1.57 [0.22, 11.17].

10.20. Serious harms reported within the MedDRA system organ class 'nervous system disorders (10029205)'*: intention to treat analysis

Study or Subgroup	HPV vaccine Events	HPV vaccine Total	Control Events	Control Total	Weight	Risk Ratio IV, Random, 95% CI
**10.20.1 Serious nervous system disorders: Cervarix vs. Al(OH)3 (females, age 18–35; follow-up until 12 months)**						
HPV-003: Cervarix vs. Al(OH)3 (f, 18–30; 12)	1	31	0	30	1.4%	2.91 [0.12, 68.66]
HPV-035: Cervarix vs. Al(OH)3 (f, 18–35; 7)	2	150	0	150	1.5%	5.00 [0.24, 103.28]
**Subtotal (95% CI)**		**181**		**180**	**2.9%**	**3.86 [0.43, 34.36]**
Total events	3		0			
Heterogeneity: Tau² = 0.00; Chi² = 0.06, df = 1 (P = 0.81); I² = 0%						
Test for overall effect: Z = 1.21 (P = 0.23)						
**10.20.2 Serious nervous system disorders: Cervarix vs. Al(OH)3 (females, age 24–72; follow-up until 36 months)**						
HPV-015: Cervarix vs. Al(OH)3 (f, 24–72; 36)	15	2882	11	2871	23.1%	1.36 [0.63, 2.95]
**Subtotal (95% CI)**		**2882**		**2871**	**23.1%**	**1.36 [0.63, 2.95]**
Total events	15		11			
Heterogeneity: Not applicable						
Test for overall effect: Z = 0.77 (P = 0.44)						
**10.20.3 Serious nervous system disorders: Cervarix vs. Aimmugen: hepatitis A vaccine (females, age 22–29; follow-up until 12 months)**						
HPV-063: Cervarix vs. Aimmugen (f, 22–29; 12)	1	375	1	377	1.8%	1.01 [0.06, 16.01]
**Subtotal (95% CI)**		**375**		**377**	**1.8%**	**1.01 [0.06, 16.01]**
Total events	1		1			
Heterogeneity: Not applicable						
Test for overall effect: Z = 0.00 (P = 1.00)						
**10.20.4 Serious nervous system disorders: Cervarix vs. Engerix: hepatitis B vaccine (females and males, age 12–16; follow-up until 72 months)**						
HPV-040: Cervarix vs. Engerix (f & m, 12–16; 72)	2	2436	0	1267	1.5%	2.60 [0.12, 54.15]
**Subtotal (95% CI)**		**2436**		**1267**	**1.5%**	**2.60 [0.12, 54.15]**
Total events	2		0			
Heterogeneity: Not applicable						
Test for overall effect: Z = 0.62 (P = 0.54)						
**10.20.5 Serious nervous system disorders: Cervarix vs. Engerix: hepatitis B vaccine (females, age 26–46; follow-up until 12 months)**						
HPV-069: Cervarix vs. Engerix (f, 26–46; 12)	1	606	0	606	1.4%	3.00 [0.12, 73.50]
**Subtotal (95% CI)**		**606**		**606**	**1.4%**	**3.00 [0.12, 73.50]**
Total events	1		0			
Heterogeneity: Not applicable						
Test for overall effect: Z = 0.67 (P = 0.50)						
**10.20.6 Serious nervous system disorders: Cervarix vs. Havrix: hepatitis A vaccine (females, age 10–14; follow-up until 12 months)**						
HPV-013: Cervarix vs. Havrix (f, 10–14; 12)	0	1035	1	1032	1.4%	0.33 [0.01, 8.15]
**Subtotal (95% CI)**		**1035**		**1032**	**1.4%**	**0.33 [0.01, 8.15]**
Total events	0		1			
Heterogeneity: Not applicable						
Test for overall effect: Z = 0.67 (P = 0.50)						
**10.20.7 Serious nervous system disorders: Cervarix vs. Havrix: hepatitis A vaccine (females, age 15–25; follow-up until 48 months)**						
HPV-008: Cervarix vs. Havrix (f, 15–25; 48)	39	9319	25	9325	55.4%	1.56 [0.95, 2.58]
**Subtotal (95% CI)**		**9319**		**9325**	**55.4%**	**1.56 [0.95, 2.58]**
Total events	39		25			
Heterogeneity: Not applicable						
Test for overall effect: Z = 1.74 (P = 0.08)						
**10.20.8 Serious nervous system disorders: Gardasil vs. AAHS (females, age 15–26; follow-up until 45 months)**						
V501-013: Gardasil vs. AAHS (f, 16–24; 45)	1	2723	5	2732	3.0%	0.20 [0.02, 1.72]
V501-015: Gardasil vs. AAHS (f, 15–26; 36)	4	6087	1	6080	2.9%	4.00 [0.45, 35.74]
**Subtotal (95% CI)**		**8810**		**8812**	**5.9%**	**0.89 [0.05, 16.65]**
Total events	5		6			
Heterogeneity: Tau² = 3.25; Chi² = 3.65, df = 1 (P = 0.06); I² = 73%						
Test for overall effect: Z = 0.08 (P = 0.94)						
**10.20.9 Serious nervous system disorders: Gardasil vs. AAHS (females, age 21–46; follow-up until 48 months)**						
V501-019: Gardasil vs. AAHS (f, 21–46; 48)	3	1911	0	1908	1.6%	6.99 [0.36, 135.21]
**Subtotal (95% CI)**		**1911**		**1908**	**1.6%**	**6.99 [0.36, 135.21]**
Total events	3		0			
Heterogeneity: Not applicable						
Test for overall effect: Z = 1.29 (P = 0.20)						
**10.20.10 Serious nervous system disorders: Gardasil vs. AAHS (males, age 15–27; follow-up until 36 months)**						
V501-020: Gardasil vs. AAHS (m, 15–27; 36)	1	2032	0	2033	1.4%	3.00 [0.12, 73.64]
**Subtotal (95% CI)**		**2032**		**2033**	**1.4%**	**3.00 [0.12, 73.64]**
Total events	1		0			
Heterogeneity: Not applicable						
Test for overall effect: Z = 0.67 (P = 0.50)						
**10.20.11 Serious nervous system disorders: Gardasil 9 vs. saline placebo (females, age 12–26; follow-up until 7 months)**						
V503-006: Gardasil 9 vs. saline (f, 12–26;7)	1	618	1	306	1.8%	0.50 [0.03, 7.89]
**Subtotal (95% CI)**		**618**		**306**	**1.8%**	**0.50 [0.03, 7.89]**
Total events	1		1			
Heterogeneity: Not applicable						
Test for overall effect: Z = 0.50 (P = 0.62)						
**10.20.12 Serious nervous system disorders: HPV 16 vaccine vs. AAHS (females, age 16–25; follow-up until 48 months)**						
V501-005: HPV 16 vaccine vs. AAHS (f, 16–25; 48)	1	1204	1	1205	1.8%	1.00 [0.06, 15.98]
**Subtotal (95% CI)**		**1204**		**1205**	**1.8%**	**1.00 [0.06, 15.98]**
Total events	1		1			
Heterogeneity: Not applicable						
Test for overall effect: Z = 0.00 (P = 1.00)						
**Total (95% CI)**		**31409**		**29922**	**100.0%**	**1.49 [1.02, 2.16]**
Total events	72		46			
Heterogeneity: Tau² = 0.00; Chi² = 8.16, df = 13 (P = 0.83); I² = 0%						
Test for overall effect: Z = 2.08 (P = 0.04)						
Test for subgroup differences: Chi² = 4.07, df = 11 (P = 0.97), I² = 0%						

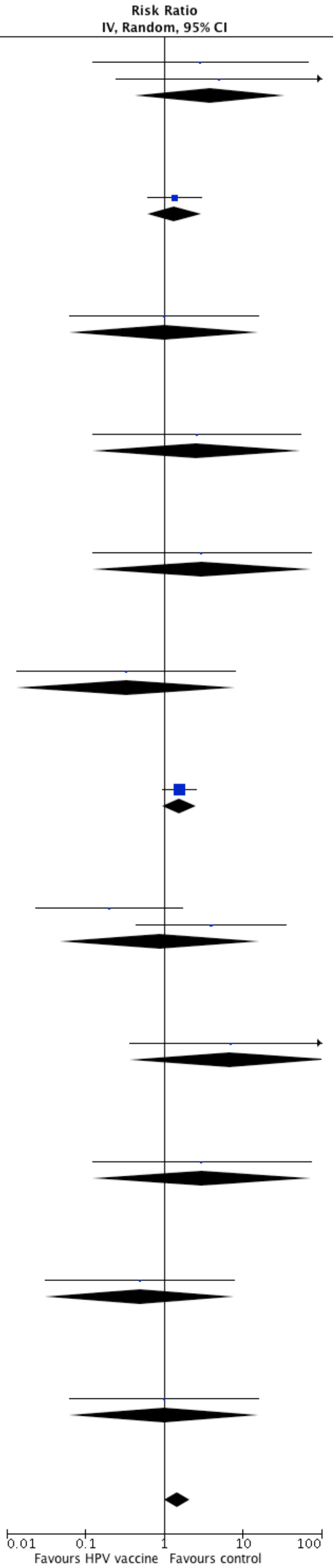

*10.20. Risk ratio for GlaxoSmithKline studies (i.e., HPV-0xx): **1.53 [1.03, 2.28]**; risk ratio for Merck Sharp & Dohme studies (i.e., V50x-xxx): 1.25 [0.39, 3.97].

- Reported MedDRA preferred terms and number of harms per MedDRA term for serious nervous system disorders:

MedDRA system organ class	MedDRA preferred term	HPV vaccine	Comparator
Nervous system disorders (10029205)	Altered state of consciousness	0	1
Nervous system disorders (10029205)	Anoxic encephalopathy (10050750)	1	0
Nervous system disorders (10029205)	Aphasia (10002948)	1	0
Nervous system disorders (10029205)	Basal ganglia haemorrhage	0	1
Nervous system disorders (10029205)	Benign intracranial hypertension (10004277)	0	1
Nervous system disorders (10029205)	Brain injury (10067967)	0	1
Nervous system disorders (10029205)	Cerebral cyst	1	0
Nervous system disorders (10029205)	Cerebral haemorrhage (10008111)	2	2
Nervous system disorders (10029205)	Cerebral ischemia	0	1
Nervous system disorders (10029205)	Cerebrovascular accident (10008190)	2	2
Nervous system disorders (10029205)	Convulsion (10010904)	5	5
Nervous system disorders (10029205)	Cubital tunnel syndrome (10056473)	1	0
Nervous system disorders (10029205)	Diabetic coma	1	0
Nervous system disorders (10029205)	Dizziness (10013573)	5	2
Nervous system disorders (10029205)	Dystonia (10013983)	1	0
Nervous system disorders (10029205)	Epilepsy (10015037)	3	3
Nervous system disorders (10029205)	Facial palsy (10016060)	2	2
Nervous system disorders (10029205)	Facial paresis (10051267)	1	0
Nervous system disorders (10029205)	Grand mal convulsion (10018659)	2	0
Nervous system disorders (10029205)	Haemorrhage intracranial	1	0
Nervous system disorders (10029205)	Headache (10019211)	11	4
Nervous system disorders (10029205)	Hydrocephalus (10020508)	0	1
Nervous system disorders (10029205)	Intracranial hematoma	0	1
Nervous system disorders (10029205)	Ischemic stroke	0	1
Nervous system disorders (10029205)	Intracranial aneurysm (10022758)	1	1
Nervous system disorders (10029205)	Intracranial venous sinus thrombosis (10061251)	1	0
Nervous system disorders (10029205)	Migraine (10027599)	9	3
Nervous system disorders (10029205)	Migraine with aura (10027607)	0	1
Nervous system disorders (10029205)	Monoparesis	0	1
Nervous system disorders (10029205)	Moyamoya disease	0	1
Nervous system disorders (10029205)	Multiple sclerosis (10028245)	4	1
Nervous system disorders (10029205)	Neuropathy peripheral (10029331)	0	1
Nervous system disorders (10029205)	Not specified	1	0
Nervous system disorders (10029205)	Optic neuritis (10030942)	3	1
Nervous system disorders (10029205)	Paraesthesia (10033775)	1	1
Nervous system disorders (10029205)	Peroneal nerve palsy (10034701)	0	1
Nervous system disorders (10029205)	Pleocytosis (10035551)	0	1
Nervous system disorders (10029205)	Polyneuropathy (10036105)	1	0
Nervous system disorders (10029205)	Sciatica (10039674)	0	1
Nervous system disorders (10029205)	Sedation	1	0
Nervous system disorders (10029205)	Subarachnoid haemorrhage (10042316)	1	1
Nervous system disorders (10029205)	Syncope (10042772)	4	3
Nervous system disorders (10029205)	Tension headache (10043269)	3	0
Nervous system disorders (10029205)	Trigeminal neuralgia	1	0
Nervous system disorders (10029205)	Vertebral artery dissection	1	0
**Total**		**72**	**46**

10.21. Serious harms reported within the MedDRA system organ class 'pregnancy, puerperium and perinatal conditions (10036585)'*: intention to treat analysis

Study or Subgroup	HPV vaccine Events	HPV vaccine Total	Control Events	Control Total	Weight	Risk Ratio IV, Random, 95% CI
**10.21.1 Serious pregnancy, puerperium and perinatal conditions: Cervarix vs. Al(OH)3 (females, age 18-35; follow-up until 7 months)**						
HPV-031: Cervarix vs. Al(OH)3 (f, 18-35; 7)	0	176	1	178	0.2%	0.34 [0.01, 8.22]
**Subtotal (95% CI)**		**176**		**178**	**0.2%**	**0.34 [0.01, 8.22]**
Total events	0		1			
Heterogeneity: Not applicable						
Test for overall effect: Z = 0.67 (P = 0.50)						
**10.21.2 Serious pregnancy, puerperium and perinatal conditions: Cervarix vs. Al(OH)3 (females, age 24-72; follow-up until 36 months)**						
HPV-015: Cervarix vs. Al(OH)3 (f, 24-72; 36)	86	2882	88	2871	19.9%	0.97 [0.73, 1.30]
**Subtotal (95% CI)**		**2882**		**2871**	**19.9%**	**0.97 [0.73, 1.30]**
Total events	86		88			
Heterogeneity: Not applicable						
Test for overall effect: Z = 0.18 (P = 0.86)						
**10.21.3 Serious pregnancy, puerperium and perinatal conditions: Cervarix vs. Aimmugen: hepatitis A vaccine (females, age 19-29; follow-up until 12 months)**						
HPV-032: Cervarix vs. Aimmugen (f, 19-25; 7)	5	519	3	521	0.8%	1.67 [0.40, 6.96]
HPV-063: Cervarix vs. Aimmugen (f, 22-29; 12)	15	375	14	377	3.3%	1.08 [0.53, 2.20]
**Subtotal (95% CI)**		**894**		**898**	**4.2%**	**1.18 [0.62, 2.23]**
Total events	20		17			
Heterogeneity: $Tau^2 = 0.00$; $Chi^2 = 0.29$, df = 1 (P = 0.59); $I^2 = 0\%$						
Test for overall effect: Z = 0.50 (P = 0.62)						
**10.21.4 Serious pregnancy, puerperium and perinatal conditions: Cervarix vs. Havrix: hepatitis A vaccine (females, age 15-25; follow-up until 48 months)**						
HPV-008: Cervarix vs. Havrix (f, 15-25; 48)	306	9319	266	9325	65.1%	1.15 [0.98, 1.35]
**Subtotal (95% CI)**		**9319**		**9325**	**65.1%**	**1.15 [0.98, 1.35]**
Total events	306		266			
Heterogeneity: Not applicable						
Test for overall effect: Z = 1.71 (P = 0.09)						
**10.21.5 Serious pregnancy, puerperium and perinatal conditions: Gardasil vs. AAHS (females, age 15-26; follow-up until 45 months)**						
V501-013: Gardasil vs. AAHS (f, 16-24; 45)	17	2723	13	2732	3.3%	1.31 [0.64, 2.70]
V501-015: Gardasil vs. AAHS (f, 15-26; 36)	24	6087	34	6080	6.3%	0.71 [0.42, 1.19]
**Subtotal (95% CI)**		**8810**		**8812**	**9.6%**	**0.91 [0.50, 1.66]**
Total events	41		47			
Heterogeneity: $Tau^2 = 0.09$; $Chi^2 = 1.87$, df = 1 (P = 0.17); $I^2 = 47\%$						
Test for overall effect: Z = 0.30 (P = 0.77)						
**10.21.6 Serious pregnancy, puerperium and perinatal conditions: Gardasil vs. AAHS (females, age 21-46; follow-up until 48 months)**						
V501-019: Gardasil vs. AAHS (f, 21-46; 48)	3	1911	7	1908	0.9%	0.43 [0.11, 1.65]
**Subtotal (95% CI)**		**1911**		**1908**	**0.9%**	**0.43 [0.11, 1.65]**
Total events	3		7			
Heterogeneity: Not applicable						
Test for overall effect: Z = 1.23 (P = 0.22)						
**10.21.7 Serious pregnancy, puerperium and perinatal conditions: HPV 16 vaccine vs. AAHS (females, age 16-25; follow-up until 48 months)**						
V501-005: HPV 16 vaccine vs. AAHS (f, 16-25; 48)	2	1204	0	1205	0.2%	5.00 [0.24, 104.12]
**Subtotal (95% CI)**		**1204**		**1205**	**0.2%**	**5.00 [0.24, 104.12]**
Total events	2		0			
Heterogeneity: Not applicable						
Test for overall effect: Z = 1.04 (P = 0.30)						
**Total (95% CI)**		**25196**		**25197**	**100.0%**	**1.08 [0.94, 1.23]**
Total events	458		426			
Heterogeneity: $Tau^2 = 0.00$; $Chi^2 = 7.58$, df = 8 (P = 0.48); $I^2 = 0\%$						
Test for overall effect: Z = 1.10 (P = 0.27)						
Test for subgroup differences: $Chi^2 = 4.73$, df = 6 (P = 0.58), $I^2 = 0\%$						

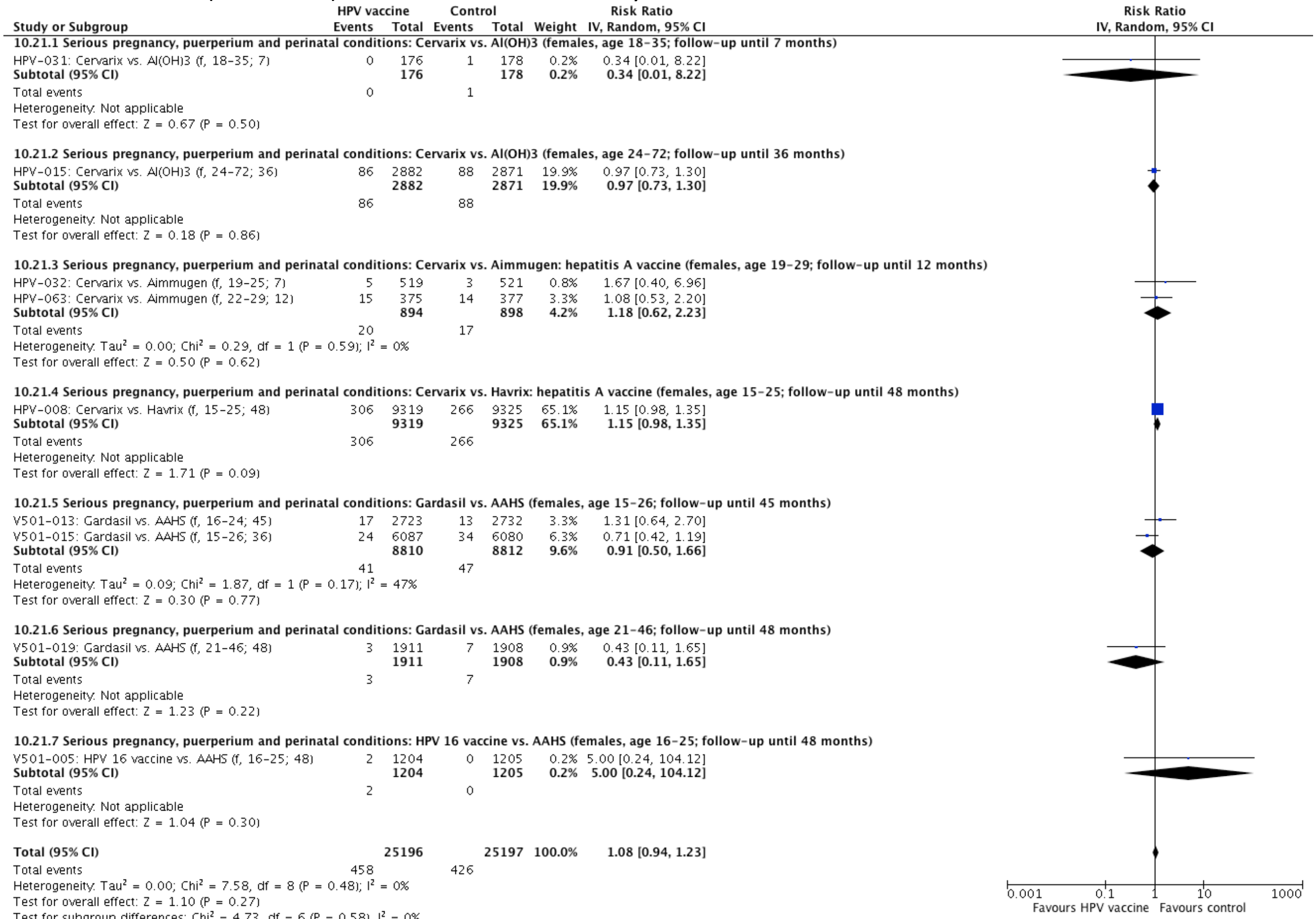

*10.21. Risk ratio for GlaxoSmithKline studies (i.e., HPV-0xx): 1.11 [0.96, 1.27]; risk ratio for Merck Sharp & Dohme studies (i.e., V50x-xxx): 0.87 [0.50, 1.49].

10.22. Serious harms reported within the MedDRA system organ class 'psychiatric disorders (10037175)'*: intention to treat analysis

Study or Subgroup	HPV vaccine Events	HPV vaccine Total	Control Events	Control Total	Weight	Risk Ratio IV, Random, 95% CI
**10.22.1 Serious psychiatric disorders: Cervarix vs. Al(OH)3 (females, age 24–72; follow-up until 36 months)**						
HPV-015: Cervarix vs. Al(OH)3 (f, 24–72; 36)	12	2882	9	2871	12.8%	1.33 [0.56, 3.15]
**Subtotal (95% CI)**		**2882**		**2871**	**12.8%**	**1.33 [0.56, 3.15]**
Total events	12		9			
Heterogeneity: Not applicable						
Test for overall effect: Z = 0.64 (P = 0.52)						
**10.22.2 Serious psychiatric disorders: Cervarix vs. Aimmugen: hepatitis A vaccine (females, age 22–29; follow-up until 12 months)**						
HPV-063: Cervarix vs. Aimmugen (f, 22–29; 12)	4	375	1	377	2.0%	4.02 [0.45, 35.81]
**Subtotal (95% CI)**		**375**		**377**	**2.0%**	**4.02 [0.45, 35.81]**
Total events	4		1			
Heterogeneity: Not applicable						
Test for overall effect: Z = 1.25 (P = 0.21)						
**10.22.3 Serious psychiatric disorders: Cervarix vs. Engerix: hepatitis B vaccine (females and males, age 12–16; follow-up until 72 months)**						
HPV-040: Cervarix vs. Engerix (f & m, 12–16; 72)	4	2436	3	1267	4.3%	0.69 [0.16, 3.09]
**Subtotal (95% CI)**		**2436**		**1267**	**4.3%**	**0.69 [0.16, 3.09]**
Total events	4		3			
Heterogeneity: Not applicable						
Test for overall effect: Z = 0.48 (P = 0.63)						
**10.22.4 Serious psychiatric disorders: Cervarix vs. Havrix: hepatitis A vaccine (females, age 10–14; follow-up until 12 months)**						
HPV-013: Cervarix vs. Havrix (f, 10–14; 12)	0	1035	1	1032	0.9%	0.33 [0.01, 8.15]
**Subtotal (95% CI)**		**1035**		**1032**	**0.9%**	**0.33 [0.01, 8.15]**
Total events	0		1			
Heterogeneity: Not applicable						
Test for overall effect: Z = 0.67 (P = 0.50)						
**10.22.5 Serious psychiatric disorders: Cervarix vs. Havrix: hepatitis A vaccine (females, age 15–25; follow-up until 48 months)**						
HPV-008: Cervarix vs. Havrix (f, 15–25; 48)	56	9319	61	9325	72.9%	0.92 [0.64, 1.32]
**Subtotal (95% CI)**		**9319**		**9325**	**72.9%**	**0.92 [0.64, 1.32]**
Total events	56		61			
Heterogeneity: Not applicable						
Test for overall effect: Z = 0.46 (P = 0.65)						
**10.22.6 Serious psychiatric disorders: Cervarix vs. Twinrix: hepatitis A and B vaccine (females, age 8–15; follow-up until 12 months)**						
HPV-029: Cervarix vs. Twinrix (f, 8–15; 12)	1	270	1	271	1.2%	1.00 [0.06, 15.96]
**Subtotal (95% CI)**		**270**		**271**	**1.2%**	**1.00 [0.06, 15.96]**
Total events	1		1			
Heterogeneity: Not applicable						
Test for overall effect: Z = 0.00 (P = 1.00)						
**10.22.7 Serious psychiatric disorders: Gardasil vs. AAHS (females, age 15–26; follow-up until 45 months)**						
V501-013: Gardasil vs. AAHS (f, 16–24; 45)	1	2723	0	2732	0.9%	3.01 [0.12, 73.85]
V501-015: Gardasil vs. AAHS (f, 15–26; 36)	1	6087	2	6080	1.7%	0.50 [0.05, 5.51]
**Subtotal (95% CI)**		**8810**		**8812**	**2.6%**	**0.95 [0.14, 6.50]**
Total events	2		2			
Heterogeneity: $Tau^2 = 0.00$; $Chi^2 = 0.77$, df = 1 (P = 0.38); $I^2 = 0\%$						
Test for overall effect: Z = 0.05 (P = 0.96)						
**10.22.8 Serious psychiatric disorders: Gardasil vs. AAHS (males, age 15–27; follow-up until 36 months)**						
V501-020: Gardasil vs. AAHS (m, 15–27; 36)	0	2032	2	2033	1.0%	0.20 [0.01, 4.17]
**Subtotal (95% CI)**		**2032**		**2033**	**1.0%**	**0.20 [0.01, 4.17]**
Total events	0		2			
Heterogeneity: Not applicable						
Test for overall effect: Z = 1.04 (P = 0.30)						
**10.22.9 Serious psychiatric disorders: HPV 16 vaccine vs. AAHS (females, age 16–25; follow-up until 48 months)**						
V501-005: HPV 16 vaccine vs. AAHS (f, 16–25; 48)	1	1204	7	1205	2.2%	0.14 [0.02, 1.16]
**Subtotal (95% CI)**		**1204**		**1205**	**2.2%**	**0.14 [0.02, 1.16]**
Total events	1		7			
Heterogeneity: Not applicable						
Test for overall effect: Z = 1.82 (P = 0.07)						
**Total (95% CI)**		**28363**		**27193**	**100.0%**	**0.92 [0.68, 1.25]**
Total events	80		87			
Heterogeneity: $Tau^2 = 0.00$; $Chi^2 = 7.76$, df = 9 (P = 0.56); $I^2 = 0\%$						
Test for overall effect: Z = 0.53 (P = 0.59)						
Test for subgroup differences: $Chi^2 = 6.98$, df = 8 (P = 0.54), $I^2 = 0\%$						

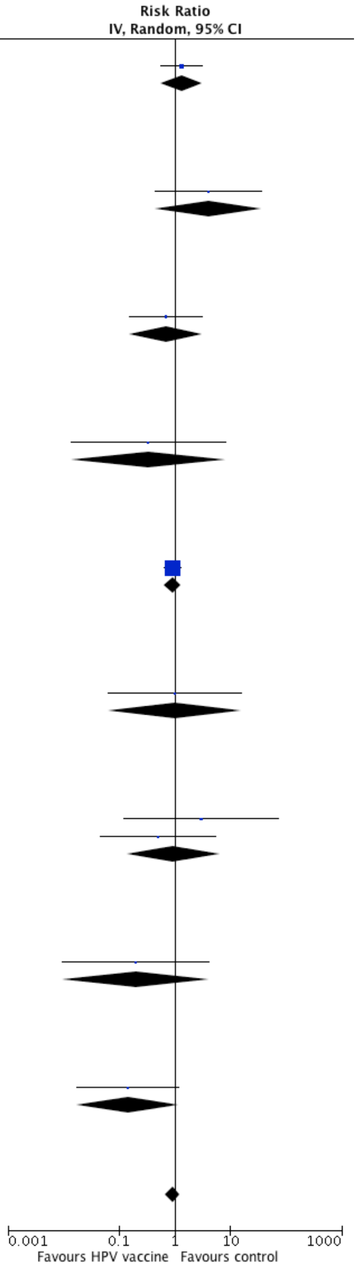

*10.22. Risk ratio for GlaxoSmithKline studies (i.e., HPV-0xx): 0.98 [0.71, 1.34]; risk ratio for Merck Sharp & Dohme studies (i.e., V50x-xxx): 0.35 [0.10, 1.28].

10.23. Serious harms reported within the MedDRA system organ class 'renal and urinary disorders (10038359)'*: intention to treat analysis

Study or Subgroup	HPV vaccine Events	HPV vaccine Total	Control Events	Control Total	Weight	Risk Ratio IV, Random, 95% CI
**10.23.1 Serious renal and urinary disorders: Cervarix vs. Al(OH)3 (females, age 24-72; follow-up until 36 months)**						
HPV-015: Cervarix vs. Al(OH)3 (f, 24-72; 36)	8	2882	7	2871	38.9%	1.14 [0.41, 3.14]
**Subtotal (95% CI)**		**2882**		**2871**	**38.9%**	**1.14 [0.41, 3.14]**
Total events	8		7			
Heterogeneity: Not applicable						
Test for overall effect: Z = 0.25 (P = 0.80)						
**10.23.2 Serious renal and urinary disorders: Cervarix vs. Engerix: hepatitis B vaccine (females and males, age 9-16; follow-up until 12 months)**						
HPV-030: Cervarix vs. Engerix (f, 9-15; 12)	1	247	0	246	3.9%	2.99 [0.12, 72.99]
**Subtotal (95% CI)**		**247**		**246**	**3.9%**	**2.99 [0.12, 72.99]**
Total events	1		0			
Heterogeneity: Not applicable						
Test for overall effect: Z = 0.67 (P = 0.50)						
**10.23.3 Serious renal and urinary disorders: Cervarix vs. Havrix: hepatitis A vaccine (females, age 15-25; follow-up until 48 months)**						
HPV-008: Cervarix vs. Havrix (f, 15-25; 48)	8	9319	8	9325	41.6%	1.00 [0.38, 2.67]
**Subtotal (95% CI)**		**9319**		**9325**	**41.6%**	**1.00 [0.38, 2.67]**
Total events	8		8			
Heterogeneity: Not applicable						
Test for overall effect: Z = 0.00 (P = 1.00)						
**10.23.4 Serious renal and urinary disorders: Gardasil vs. carrier solution (females and males, age 9-16; follow-up until 18 months)**						
V501-018: Gardasil vs. carrier solu. (f&m,9-16;18)	1	1184	0	597	3.9%	1.51 [0.06, 37.11]
**Subtotal (95% CI)**		**1184**		**597**	**3.9%**	**1.51 [0.06, 37.11]**
Total events	1		0			
Heterogeneity: Not applicable						
Test for overall effect: Z = 0.25 (P = 0.80)						
**10.23.5 Serious renal and urinary disorders: Gardasil vs. AAHS (females, age 15-26; follow-up until 45 months)**						
V501-013: Gardasil vs. AAHS (f, 16-24; 45)	0	2723	1	2732	3.9%	0.33 [0.01, 8.21]
V501-015: Gardasil vs. AAHS (f, 15-26; 36)	0	6087	1	6080	3.9%	0.33 [0.01, 8.17]
**Subtotal (95% CI)**		**8810**		**8812**	**7.8%**	**0.33 [0.03, 3.21]**
Total events	0		2			
Heterogeneity: $Tau^2 = 0.00$; $Chi^2 = 0.00$, df = 1 (P = 1.00); $I^2 = 0\%$						
Test for overall effect: Z = 0.95 (P = 0.34)						
**10.23.6 Serious renal and urinary disorders: Gardasil vs. AAHS (females, age 21-46; follow-up until 48 months)**						
V501-019: Gardasil vs. AAHS (f, 21-46; 48)	1	1911	0	1908	3.9%	3.00 [0.12, 73.48]
**Subtotal (95% CI)**		**1911**		**1908**	**3.9%**	**3.00 [0.12, 73.48]**
Total events	1		0			
Heterogeneity: Not applicable						
Test for overall effect: Z = 0.67 (P = 0.50)						
**Total (95% CI)**		**24353**		**23759**	**100.0%**	**1.07 [0.57, 2.01]**
Total events	19		17			
Heterogeneity: $Tau^2 = 0.00$; $Chi^2 = 1.89$, df = 6 (P = 0.93); $I^2 = 0\%$						
Test for overall effect: Z = 0.21 (P = 0.84)						
Test for subgroup differences: $Chi^2 = 1.89$, df = 5 (P = 0.86), $I^2 = 0\%$						

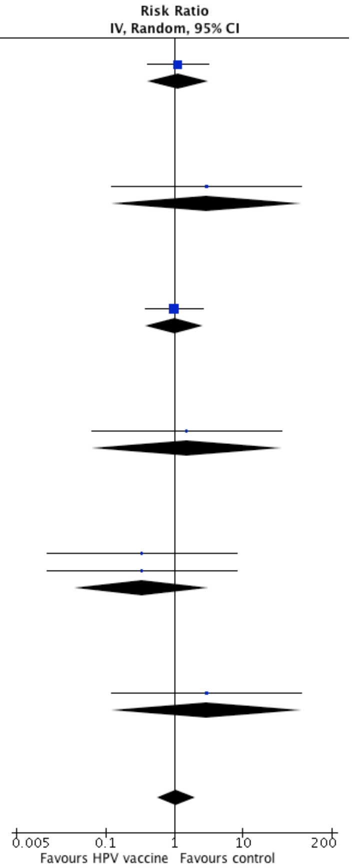

*10.23. Risk ratio for GlaxoSmithKline studies (i.e., HPV-0xx): 1.12 [0.56, 2.22]; risk ratio for Merck Sharp & Dohme studies (i.e., V50x-xxx): 0.83 [0.17, 4.13].

10.24. Serious harms reported within the MedDRA system organ class 'reproductive system and breast disorders (10038604)'*: intention to treat analysis

Study or Subgroup	HPV vaccine Events	HPV vaccine Total	Control Events	Control Total	Weight	Risk Ratio IV, Random, 95% CI
**10.24.1 Serious reproductive system and breast disorders: Cervarix vs. Al(OH)3 (females, age 15-25; follow-up until 7 months)**						
HPV-038: Cervarix vs. Al(OH)3 (f, 15-25; 7)	1	149	0	76	1.0%	1.54 [0.06, 37.36]
**Subtotal (95% CI)**		**149**		**76**	**1.0%**	**1.54 [0.06, 37.36]**
Total events	1		0			
Heterogeneity: Not applicable						
Test for overall effect: Z = 0.27 (P = 0.79)						
**10.24.2 Serious reproductive system and breast disorders: Cervarix vs. Al(OH)3 (females, age 24-72; follow-up until 36 months)**						
HPV-015: Cervarix vs. Al(OH)3 (f, 24-72; 36)	22	2882	27	2871	32.5%	0.81 [0.46, 1.42]
**Subtotal (95% CI)**		**2882**		**2871**	**32.5%**	**0.81 [0.46, 1.42]**
Total events	22		27			
Heterogeneity: Not applicable						
Test for overall effect: Z = 0.73 (P = 0.47)						
**10.24.3 Serious reproductive system and breast disorders: Cervarix vs. Aimmugen: hepatitis A vaccine (females, age 22-29; follow-up until 12 months)**						
HPV-063: Cervarix vs. Aimmugen (f, 22-29; 12)	1	375	1	377	1.3%	1.01 [0.06, 16.01]
**Subtotal (95% CI)**		**375**		**377**	**1.3%**	**1.01 [0.06, 16.01]**
Total events	1		1			
Heterogeneity: Not applicable						
Test for overall effect: Z = 0.00 (P = 1.00)						
**10.24.4 Serious reproductive system and breast disorders: Cervarix vs. Engerix: hepatitis B vaccine (females and males, age 12-16; follow-up until 72 months)**						
HPV-040: Cervarix vs. Engerix (f & m, 12-16; 72)	1	2436	0	1267	1.0%	1.56 [0.06, 38.29]
**Subtotal (95% CI)**		**2436**		**1267**	**1.0%**	**1.56 [0.06, 38.29]**
Total events	1		0			
Heterogeneity: Not applicable						
Test for overall effect: Z = 0.27 (P = 0.79)						
**10.24.5 Serious reproductive system and breast disorders: Cervarix vs. Havrix: hepatitis A vaccine (females, age 10-14; follow-up until 12 months)**						
HPV-013: Cervarix vs. Havrix (f, 10-14; 12)	0	1035	1	1032	1.0%	0.33 [0.01, 8.15]
**Subtotal (95% CI)**		**1035**		**1032**	**1.0%**	**0.33 [0.01, 8.15]**
Total events	0		1			
Heterogeneity: Not applicable						
Test for overall effect: Z = 0.67 (P = 0.50)						
**10.24.6 Serious reproductive system and breast disorders: Cervarix vs. Havrix: hepatitis A vaccine (females, age 15-25; follow-up until 48 months)**						
HPV-008: Cervarix vs. Havrix (f, 15-25; 48)	39	9319	42	9325	54.0%	0.93 [0.60, 1.44]
**Subtotal (95% CI)**		**9319**		**9325**	**54.0%**	**0.93 [0.60, 1.44]**
Total events	39		42			
Heterogeneity: Not applicable						
Test for overall effect: Z = 0.33 (P = 0.74)						
**10.24.7 Serious reproductive system and breast disorders: Gardasil vs. carrier solution (females and males, age 9-16; follow-up until 18 months)**						
V501-018: Gardasil vs. carrier solu. (f&m,9-16;18)	1	1184	0	597	1.0%	1.51 [0.06, 37.11]
**Subtotal (95% CI)**		**1184**		**597**	**1.0%**	**1.51 [0.06, 37.11]**
Total events	1		0			
Heterogeneity: Not applicable						
Test for overall effect: Z = 0.25 (P = 0.80)						
**10.24.8 Serious reproductive system and breast disorders: Gardasil vs. AAHS (females, age 15-26; follow-up until 36 months)**						
V501-015: Gardasil vs. AAHS (f, 15-26; 36)	2	6087	4	6080	3.5%	0.50 [0.09, 2.73]
**Subtotal (95% CI)**		**6087**		**6080**	**3.5%**	**0.50 [0.09, 2.73]**
Total events	2		4			
Heterogeneity: Not applicable						
Test for overall effect: Z = 0.80 (P = 0.42)						
**10.24.9 Serious reproductive system and breast disorders: Gardasil vs. AAHS (females, age 21-46; follow-up until 48 months)**						
V501-019: Gardasil vs. AAHS (f, 21-46; 48)	2	1911	2	1908	2.7%	1.00 [0.14, 7.08]
**Subtotal (95% CI)**		**1911**		**1908**	**2.7%**	**1.00 [0.14, 7.08]**
Total events	2		2			
Heterogeneity: Not applicable						
Test for overall effect: Z = 0.00 (P = 1.00)						
**10.24.10 Serious reproductive system and breast disorders : HPV 16 vaccine vs. AAHS (females, age 16-25; follow-up until 48 months)**						
V501-005: HPV 16 vaccine vs. AAHS (f, 16-25; 48)	3	1204	1	1205	2.0%	3.00 [0.31, 28.82]
**Subtotal (95% CI)**		**1204**		**1205**	**2.0%**	**3.00 [0.31, 28.82]**
Total events	3		1			
Heterogeneity: Not applicable						
Test for overall effect: Z = 0.95 (P = 0.34)						
**Total (95% CI)**		**26582**		**24738**	**100.0%**	**0.90 [0.65, 1.24]**
Total events	72		78			
Heterogeneity: $Tau^2 = 0.00$; $Chi^2 = 2.42$, df = 9 (P = 0.98); $I^2 = 0\%$						
Test for overall effect: Z = 0.66 (P = 0.51)						
Test for subgroup differences: $Chi^2 = 2.42$, df = 9 (P = 0.98), $I^2 = 0\%$						

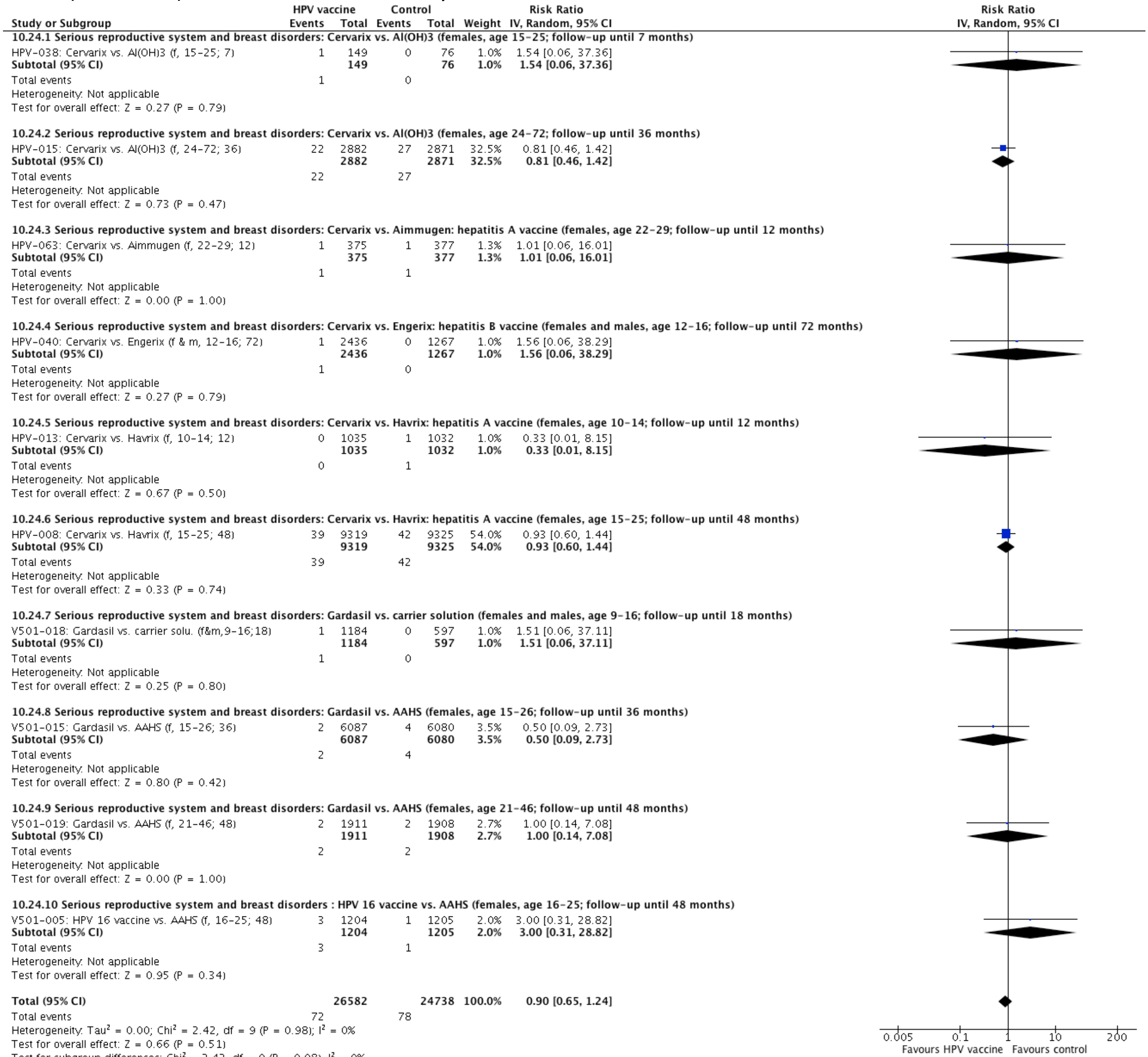

*10.24. Risk ratio for GlaxoSmithKline studies (i.e., HPV-0xx): 0.89 [0.63, 1.24]; risk ratio for Merck Sharp & Dohme studies (i.e., V50x-xxx): 1.02 [0.36, 2.92].

10.25. Serious harms reported within the MedDRA system organ class 'respiratory, thoracic and mediastinal disorders (10038738)'*: intention to treat analysis

Study or Subgroup	HPV vaccine Events	HPV vaccine Total	Control Events	Control Total	Weight	Risk Ratio IV, Random, 95% CI
**10.25.1 Serious respiratory, thoracic and mediastinal disorders: Cervarix vs. Al(OH)3 (females, age 18-35; follow-up until 7 months)**						
HPV-031: Cervarix vs. Al(OH)3 (f, 18-35; 7)	0	176	1	178	2.0%	0.34 [0.01, 8.22]
**Subtotal (95% CI)**		**176**		**178**	**2.0%**	**0.34 [0.01, 8.22]**
Total events	0		1			
Heterogeneity: Not applicable						
Test for overall effect: Z = 0.67 (P = 0.50)						
**10.25.2 Serious respiratory, thoracic and mediastinal disorders: Cervarix vs. Al(OH)3 (females, age 24-72; follow-up until 36 months)**						
HPV-015: Cervarix vs. Al(OH)3 (f, 24-72; 36)	12	2882	8	2871	25.0%	1.49 [0.61, 3.65]
**Subtotal (95% CI)**		**2882**		**2871**	**25.0%**	**1.49 [0.61, 3.65]**
Total events	12		8			
Heterogeneity: Not applicable						
Test for overall effect: Z = 0.88 (P = 0.38)						
**10.25.3 Serious respiratory, thoracic and mediastinal disorders: Cervarix vs. Aimmugen: hepatitis A vaccine (females, age 22-29; follow-up until 12 months)**						
HPV-063: Cervarix vs. Aimmugen (f, 22-29; 12)	2	375	0	377	2.2%	5.03 [0.24, 104.35]
**Subtotal (95% CI)**		**375**		**377**	**2.2%**	**5.03 [0.24, 104.35]**
Total events	2		0			
Heterogeneity: Not applicable						
Test for overall effect: Z = 1.04 (P = 0.30)						
**10.25.4 Serious respiratory, thoracic and mediastinal disorders: Cervarix vs. Engerix: hepatitis B vaccine (females and males, age 12-16; follow-up until 72 months)**						
HPV-040: Cervarix vs. Engerix (f & m, 12-16; 72)	0	2436	1	1267	1.9%	0.17 [0.01, 4.25]
**Subtotal (95% CI)**		**2436**		**1267**	**1.9%**	**0.17 [0.01, 4.25]**
Total events	0		1			
Heterogeneity: Not applicable						
Test for overall effect: Z = 1.07 (P = 0.28)						
**10.25.5 Serious respiratory, thoracic and mediastinal disorders: Cervarix vs. Havrix: hepatitis A vaccine (females, age 15-25; follow-up until 48 months)**						
HPV-008: Cervarix vs. Havrix (f, 15-25; 48)	21	9319	17	9325	48.9%	1.24 [0.65, 2.34]
**Subtotal (95% CI)**		**9319**		**9325**	**48.9%**	**1.24 [0.65, 2.34]**
Total events	21		17			
Heterogeneity: Not applicable						
Test for overall effect: Z = 0.65 (P = 0.52)						
**10.25.6 Serious respiratory, thoracic and mediastinal disorders: Gardasil vs. AAHS (females, age 15-26; follow-up until 45 months)**						
V501-013: Gardasil vs. AAHS (f, 16-24; 45)	3	2723	2	2732	6.2%	1.50 [0.25, 9.00]
V501-015: Gardasil vs. AAHS (f, 15-26; 36)	2	6087	2	6080	5.2%	1.00 [0.14, 7.09]
**Subtotal (95% CI)**		**8810**		**8812**	**11.4%**	**1.25 [0.33, 4.68]**
Total events	5		4			
Heterogeneity: Tau² = 0.00; Chi² = 0.09, df = 1 (P = 0.76); I² = 0%						
Test for overall effect: Z = 0.33 (P = 0.74)						
**10.25.7 Serious respiratory, thoracic and mediastinal disorders: Gardasil vs. AAHS (females, age 21-46; follow-up until 48 months)**						
V501-019: Gardasil vs. AAHS (f, 21-46; 48)	2	1911	2	1908	5.2%	1.00 [0.14, 7.08]
**Subtotal (95% CI)**		**1911**		**1908**	**5.2%**	**1.00 [0.14, 7.08]**
Total events	2		2			
Heterogeneity: Not applicable						
Test for overall effect: Z = 0.00 (P = 1.00)						
**10.25.8 Serious respiratory, thoracic and mediastinal disorders: HPV 16 vaccine vs. AAHS (females, age 16-25; follow-up until 48 months)**						
V501-005: HPV 16 vaccine vs. AAHS (f, 16-25; 48)	2	1204	1	1205	3.5%	2.00 [0.18, 22.05]
**Subtotal (95% CI)**		**1204**		**1205**	**3.5%**	**2.00 [0.18, 22.05]**
Total events	2		1			
Heterogeneity: Not applicable						
Test for overall effect: Z = 0.57 (P = 0.57)						
**Total (95% CI)**		**27113**		**25943**	**100.0%**	**1.26 [0.81, 1.97]**
Total events	44		34			
Heterogeneity: Tau² = 0.00; Chi² = 3.36, df = 8 (P = 0.91); I² = 0%						
Test for overall effect: Z = 1.02 (P = 0.31)						
Test for subgroup differences: Chi² = 3.27, df = 7 (P = 0.86), I² = 0%						

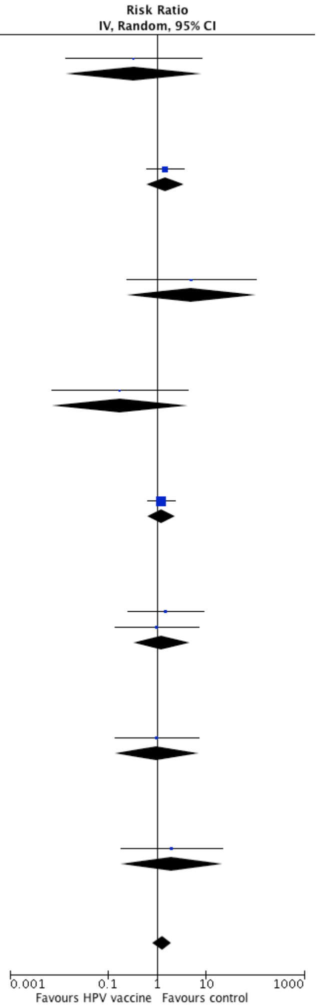

*10.25. Risk ratio for GlaxoSmithKline studies (i.e., HPV-0xx): 1.26 [0.76, 2.07]; risk ratio for Merck Sharp & Dohme studies (i.e., V50x-xxx): 1.28 [0.47, 3.46].

10.26. Serious harms reported within the MedDRA system organ class 'skin and subcutaneous tissue disorders (10040785)'*: intention to treat analysis

Study or Subgroup	HPV vaccine Events	HPV vaccine Total	Control Events	Control Total	Weight	Risk Ratio IV, Random, 95% CI
**10.26.1 Serious skin and subcutaneous tissue disorders: Cervarix vs. Al(OH)3 (females, age 9-17; follow-up until 12 months)**						
HPV-058: Cervarix vs. Al(OH)3 (f, 9-17; 12)	0	374	1	376	7.9%	0.34 [0.01, 8.20]
**Subtotal (95% CI)**		**374**		**376**	**7.9%**	**0.34 [0.01, 8.20]**
Total events	0		1			
Heterogeneity: Not applicable						
Test for overall effect: Z = 0.67 (P = 0.50)						
**10.26.2 Serious skin and subcutaneous tissue disorders: Cervarix vs. Aimmugen: hepatitis A vaccine (females, age 22-29; follow-up until 12 months)**						
HPV-063: Cervarix vs. Aimmugen (f, 22-29; 12)	1	375	0	377	7.9%	3.02 [0.12, 73.80]
**Subtotal (95% CI)**		**375**		**377**	**7.9%**	**3.02 [0.12, 73.80]**
Total events	1		0			
Heterogeneity: Not applicable						
Test for overall effect: Z = 0.68 (P = 0.50)						
**10.26.3 Serious skin and subcutaneous tissue disorders: Cervarix vs. Engerix: hepatitis B vaccine (females and males, age 12-16; follow-up until 72 months)**						
HPV-040: Cervarix vs. Engerix (f & m, 12-16; 72)	2	2436	0	1267	8.8%	2.60 [0.12, 54.15]
**Subtotal (95% CI)**		**2436**		**1267**	**8.8%**	**2.60 [0.12, 54.15]**
Total events	2		0			
Heterogeneity: Not applicable						
Test for overall effect: Z = 0.62 (P = 0.54)						
**10.26.4 Serious skin and subcutaneous tissue disorders: Cervarix vs. Havrix: hepatitis A vaccine (females, age 15-25; follow-up until 48 months)**						
HPV-008: Cervarix vs. Havrix (f, 15-25; 48)	8	9319	5	9325	64.9%	1.60 [0.52, 4.89]
**Subtotal (95% CI)**		**9319**		**9325**	**64.9%**	**1.60 [0.52, 4.89]**
Total events	8		5			
Heterogeneity: Not applicable						
Test for overall effect: Z = 0.83 (P = 0.41)						
**10.26.5 Serious skin and subcutaneous tissue disorders: Gardasil vs. AAHS (females, age 15-26; follow-up until 36 months)**						
V501-015: Gardasil vs. AAHS (f, 15-26; 36)	1	6087	1	6080	10.5%	1.00 [0.06, 15.97]
**Subtotal (95% CI)**		**6087**		**6080**	**10.5%**	**1.00 [0.06, 15.97]**
Total events	1		1			
Heterogeneity: Not applicable						
Test for overall effect: Z = 0.00 (P = 1.00)						
**Total (95% CI)**		**18591**		**17425**	**100.0%**	**1.48 [0.60, 3.63]**
Total events	12		7			
Heterogeneity: $Tau^2 = 0.00$; $Chi^2 = 1.25$, df = 4 (P = 0.87); $I^2 = 0\%$						
Test for overall effect: Z = 0.85 (P = 0.40)						
Test for subgroup differences: $Chi^2 = 1.25$, df = 4 (P = 0.87), $I^2 = 0\%$						

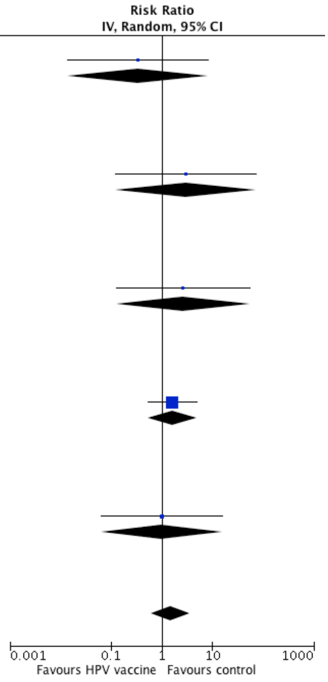

*10.26. Risk ratio for GlaxoSmithKline studies (i.e., HPV-0xx): 1.55 [0.60, 4.00]; risk ratio for Merck Sharp & Dohme studies (i.e., V50x-xxx): 1.48 [0.60, 3.63].

10.27. Serious harms reported within the MedDRA system organ class 'social circumstances (10041244)'*: intention to treat analysis

Study or Subgroup	HPV vaccine Events	HPV vaccine Total	Control Events	Control Total	Weight	Risk Ratio IV, Random, 95% CI
**10.27.1 Serious social circumstances : Cervarix vs. Al(OH)3 (females, age 24-72; follow-up until 36 months)**						
HPV-015: Cervarix vs. Al(OH)3 (f, 24-72; 36)	1	2882	0	2871	36.0%	2.99 [0.12, 73.33]
**Subtotal (95% CI)**		**2882**		**2871**	**36.0%**	**2.99 [0.12, 73.33]**
Total events	1		0			
Heterogeneity: Not applicable						
Test for overall effect: Z = 0.67 (P = 0.50)						
**10.27.2 Serious social circumstances : Cervarix vs. Havrix: hepatitis A vaccine (females, age 15-25; follow-up until 48 months)**						
HPV-008: Cervarix vs. Havrix (f, 15-25; 48)	1	9319	2	9325	64.0%	0.50 [0.05, 5.52]
**Subtotal (95% CI)**		**9319**		**9325**	**64.0%**	**0.50 [0.05, 5.52]**
Total events	1		2			
Heterogeneity: Not applicable						
Test for overall effect: Z = 0.57 (P = 0.57)						
**Total (95% CI)**		**12201**		**12196**	**100.0%**	**0.95 [0.14, 6.50]**
Total events	2		2			
Heterogeneity: Tau² = 0.00; Chi² = 0.77, df = 1 (P = 0.38); I² = 0%						
Test for overall effect: Z = 0.05 (P = 0.96)						
Test for subgroup differences: Chi² = 0.77, df = 1 (P = 0.38), I² = 0%						

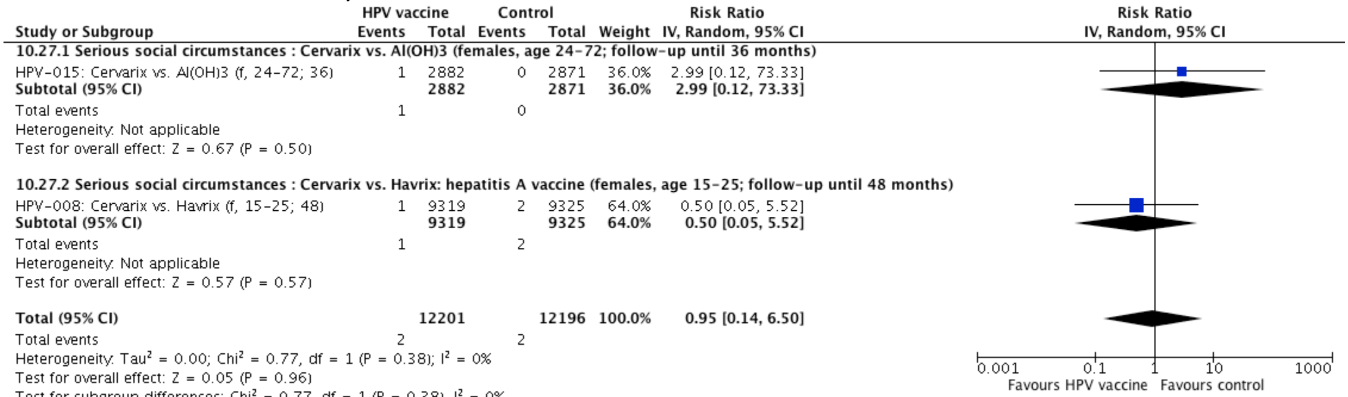

*10.27. Risk ratio for GlaxoSmithKline studies (i.e., HPV-0xx): 0.95 [0.14, 6.50]; risk ratio for Merck Sharp & Dohme studies (i.e., V50x-xxx): not applicable.

10.28. Serious harms reported within the MedDRA system organ class 'surgical and medical procedures (10042613)': intention to treat analysis

Study or Subgroup	HPV vaccine Events	HPV vaccine Total	Control Events	Control Total	Weight	Risk Ratio IV, Random, 95% CI
**10.28.1 Serious surgical and medical procedures : Cervarix vs. Al(OH)3 (females, age 24-72; follow-up until 36 months)**						
HPV-015: Cervarix vs. Al(OH)3 (f, 24-72; 36)	1	2882	0	2871	13.0%	2.99 [0.12, 73.33]
**Subtotal (95% CI)**		**2882**		**2871**	**13.0%**	**2.99 [0.12, 73.33]**
Total events	1		0			
Heterogeneity: Not applicable						
Test for overall effect: Z = 0.67 (P = 0.50)						
**10.28.2 Serious surgical and medical procedures : Cervarix vs. Havrix: hepatitis A vaccine (females, age 15-25; follow-up until 48 months)**						
HPV-008: Cervarix vs. Havrix (f, 15-25; 48)	5	9319	5	9325	87.0%	1.00 [0.29, 3.46]
**Subtotal (95% CI)**		**9319**		**9325**	**87.0%**	**1.00 [0.29, 3.46]**
Total events	5		5			
Heterogeneity: Not applicable						
Test for overall effect: Z = 0.00 (P = 1.00)						
**Total (95% CI)**		**12201**		**12196**	**100.0%**	**1.15 [0.36, 3.67]**
Total events	6		5			
Heterogeneity: $Tau^2 = 0.00$; $Chi^2 = 0.39$, df = 1 (P = 0.53); $I^2 = 0\%$						
Test for overall effect: Z = 0.24 (P = 0.81)						
Test for subgroup differences: $Chi^2 = 0.39$, df = 1 (P = 0.53), $I^2 = 0\%$						

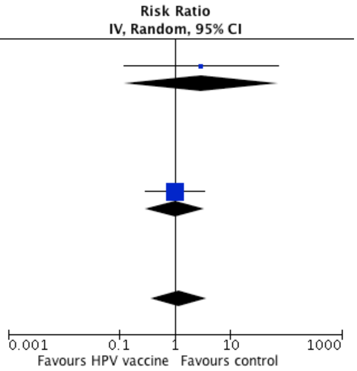

*10.28. Risk ratio for GlaxoSmithKline studies (i.e., HPV-0xx): 1.15 [0.36, 3.67]; risk ratio for Merck Sharp & Dohme studies (i.e., V50x-xxx): not applicable.

10.29. Serious harms reported within the MedDRA system organ class 'vascular disorders (10047065)': intention to treat analysis

Study or Subgroup	HPV vaccine Events	HPV vaccine Total	Control Events	Control Total	Weight	Risk Ratio IV, Random, 95% CI
**10.29.1 Serious vascular disorders: Cervarix vs. Al(OH)3 (females, age 24-72; follow-up until 36 months)**						
HPV-015: Cervarix vs. Al(OH)3 (f, 24-72; 36)	5	2882	3	2871	25.7%	1.66 [0.40, 6.94]
**Subtotal (95% CI)**		**2882**		**2871**	**25.7%**	**1.66 [0.40, 6.94]**
Total events	5		3			
Heterogeneity: Not applicable						
Test for overall effect: Z = 0.69 (P = 0.49)						
**10.29.2 Serious vascular disorders: Cervarix vs. Havrix: hepatitis A vaccine (females, age 15-25; follow-up until 48 months)**						
HPV-008: Cervarix vs. Havrix (f, 15-25; 48)	6	9319	10	9325	51.4%	0.60 [0.22, 1.65]
**Subtotal (95% CI)**		**9319**		**9325**	**51.4%**	**0.60 [0.22, 1.65]**
Total events	6		10			
Heterogeneity: Not applicable						
Test for overall effect: Z = 0.99 (P = 0.32)						
**10.29.3 Serious vascular disorders: Gardasil vs. AAHS (females, age 15-26; follow-up until 45 months)**						
V501-013: Gardasil vs. AAHS (f, 16-24; 45)	1	2723	1	2732	6.8%	1.00 [0.06, 16.03]
V501-015: Gardasil vs. AAHS (f, 15-26; 36)	4	6087	1	6087	11.0%	4.00 [0.45, 35.78]
**Subtotal (95% CI)**		**8810**		**8819**	**17.8%**	**2.35 [0.42, 13.11]**
Total events	5		2			
Heterogeneity: $Tau^2 = 0.00$; $Chi^2 = 0.59$, df = 1 (P = 0.44); $I^2 = 0\%$						
Test for overall effect: Z = 0.97 (P = 0.33)						
**10.29.4 Serious vascular disorders: Gardasil vs. AAHS (females, age 21-46; follow-up until 48 months)**						
V501-019: Gardasil vs. AAHS (f, 21-46; 48)	0	1911	1	1908	5.1%	0.33 [0.01, 8.16]
**Subtotal (95% CI)**		**1911**		**1908**	**5.1%**	**0.33 [0.01, 8.16]**
Total events	0		1			
Heterogeneity: Not applicable						
Test for overall effect: Z = 0.67 (P = 0.50)						
**Total (95% CI)**		**22922**		**22923**	**100.0%**	**0.96 [0.47, 1.99]**
Total events	16		16			
Heterogeneity: $Tau^2 = 0.00$; $Chi^2 = 3.44$, df = 4 (P = 0.49); $I^2 = 0\%$						
Test for overall effect: Z = 0.10 (P = 0.92)						
Test for subgroup differences: $Chi^2 = 2.85$, df = 3 (P = 0.41), $I^2 = 0\%$						

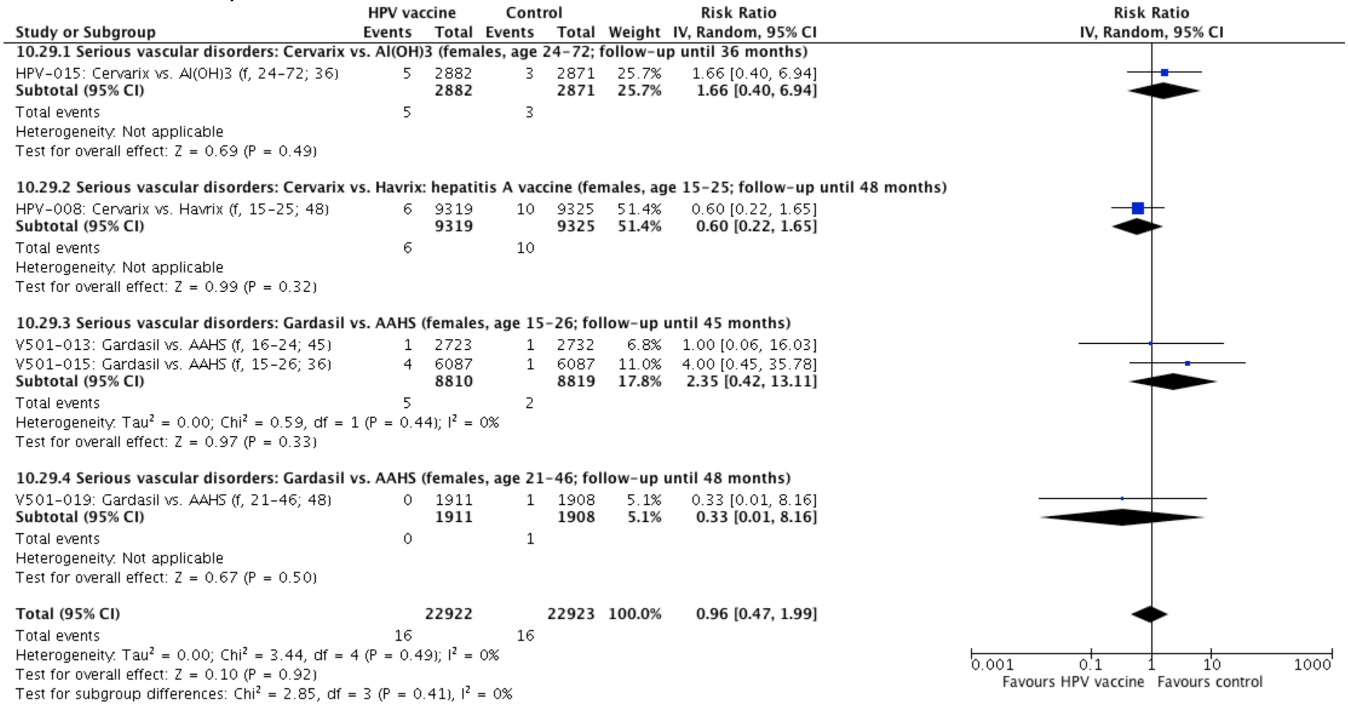

*10.29. Risk ratio for GlaxoSmithKline studies (i.e., HPV-0xx): 0.88 [0.33, 2.30]; risk ratio for Merck Sharp & Dohme studies (i.e., V50x-xxx): 1.52 [0.33, 6.89].

10.30.Most common serious harms - ‘abortion spontaneous’*: intention to treat analysis

Study or Subgroup	HPV vaccine Events	HPV vaccine Total	Control Events	Control Total	Weight	Risk Ratio IV, Random, 95% CI
**10.30.1 Serious abortion spontaneous: Cervarix vs. Al(OH)3 (females, age 18-35; follow-up until 7 months)**						
HPV-031: Cervarix vs. Al(OH)3 (f, 18-35; 7)	0	176	1	178	0.9%	0.34 [0.01, 8.22]
**Subtotal (95% CI)**		**176**		**178**	**0.9%**	**0.34 [0.01, 8.22]**
Total events	0		1			
Heterogeneity: Not applicable						
Test for overall effect: Z = 0.67 (P = 0.50)						
**10.30.2 Serious abortion spontaneous: Cervarix vs. Al(OH)3 (females, age 24-72; follow-up until 36 months)**						
HPV-015: Cervarix vs. Al(OH)3 (f, 24-72; 36)	11	2882	12	2871	13.8%	0.91 [0.40, 2.07]
**Subtotal (95% CI)**		**2882**		**2871**	**13.8%**	**0.91 [0.40, 2.07]**
Total events	11		12			
Heterogeneity: Not applicable						
Test for overall effect: Z = 0.22 (P = 0.83)						
**10.30.3 Serious abortion spontaneous: Cervarix vs. Aimmugen: hepatitis A vaccine (females, age 19-29; follow-up until 12 months)**						
HPV-032: Cervarix vs. Aimmugen (f, 19-25; 7)	5	519	3	521	4.5%	1.67 [0.40, 6.96]
HPV-063: Cervarix vs. Aimmugen (f, 22-29; 12)	3	375	3	377	3.6%	1.01 [0.20, 4.95]
**Subtotal (95% CI)**		**894**		**898**	**8.1%**	**1.33 [0.46, 3.86]**
Total events	8		6			
Heterogeneity: Tau² = 0.00; Chi² = 0.22, df = 1 (P = 0.64); I² = 0%						
Test for overall effect: Z = 0.53 (P = 0.60)						
**10.30.4 Serious abortion spontaneous: Cervarix vs. Havrix: hepatitis A vaccine (females, age 15-25; follow-up until 48 months)**						
HPV-008: Cervarix vs. Havrix (f, 15-25; 48)	70	9319	59	9325	77.2%	1.19 [0.84, 1.68]
**Subtotal (95% CI)**		**9319**		**9325**	**77.2%**	**1.19 [0.84, 1.68]**
Total events	70		59			
Heterogeneity: Not applicable						
Test for overall effect: Z = 0.97 (P = 0.33)						
**Total (95% CI)**		**13271**		**13272**	**100.0%**	**1.14 [0.84, 1.55]**
Total events	89		78			
Heterogeneity: Tau² = 0.00; Chi² = 1.20, df = 4 (P = 0.88); I² = 0%						
Test for overall effect: Z = 0.86 (P = 0.39)						
Test for subgroup differences: Chi² = 0.98, df = 3 (P = 0.81), I² = 0%						

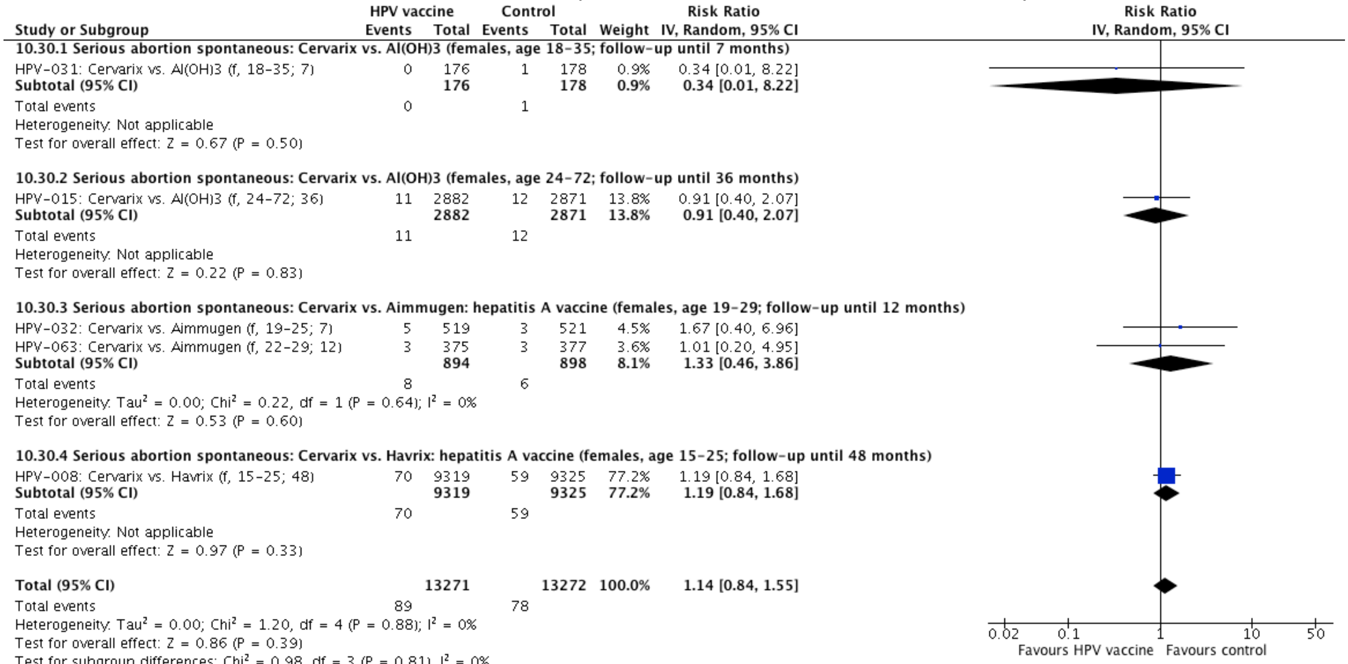

*10.30. Risk ratio for GlaxoSmithKline studies (i.e., HPV-0xx): 1.14 [0.84, 1.55]; risk ratio for Merck Sharp & Dohme studies (i.e., V50x-xxx): not applicable. The total risk ratio for all included serious abortion categories (‘abortion complete,’ ‘abortion incomplete,’ ‘abortion missed,’ ‘abortion spontaneous,’ ‘abortion spontaneous complete,’ ‘abortion spontaneous incomplete’ and ‘abortion threatened’) is 1.08 [0.91, 1.28] (271/13,271 vs. 254/13,272).

10.31. Most common serious harms - ‘appendicitis’*: intention to treat analysis

Study or Subgroup	HPV vaccine Events	HPV vaccine Total	Control Events	Control Total	Weight	Risk Ratio IV, Random, 95% CI
**10.31.1 Serious appendicitis: Cervarix vs. Al(OH)3 (females, age 18-35; follow-up until 7 months)**						
HPV-031: Cervarix vs. Al(OH)3 (f, 18-35; 7)	1	176	0	178	1.0%	3.03 [0.12, 73.97]
**Subtotal (95% CI)**		**176**		**178**	**1.0%**	**3.03 [0.12, 73.97]**
Total events	1		0			
Heterogeneity: Not applicable						
Test for overall effect: Z = 0.68 (P = 0.50)						
**10.31.2 Serious appendicitis: Cervarix vs. Al(OH)3 (females, age 24-72; follow-up until 36 months)**						
HPV-015: Cervarix vs. Al(OH)3 (f, 24-72; 36)	13	2882	12	2871	16.5%	1.08 [0.49, 2.36]
**Subtotal (95% CI)**		**2882**		**2871**	**16.5%**	**1.08 [0.49, 2.36]**
Total events	13		12			
Heterogeneity: Not applicable						
Test for overall effect: Z = 0.19 (P = 0.85)						
**10.31.3 Serious appendicitis: Cervarix vs. Aimmugen: hepatitis A vaccine (females, age 22-29; follow-up until 12 months)**						
HPV-063: Cervarix vs. Aimmugen (f, 22-29; 12)	1	375	2	377	1.8%	0.50 [0.05, 5.52]
**Subtotal (95% CI)**		**375**		**377**	**1.8%**	**0.50 [0.05, 5.52]**
Total events	1		2			
Heterogeneity: Not applicable						
Test for overall effect: Z = 0.56 (P = 0.57)						
**10.31.4 Serious appendicitis: Cervarix vs. Engerix: hepatitis B vaccine (females and males, age 12-16; follow-up until 72 months)**						
HPV-040: Cervarix vs. Engerix (f & m, 12-16; 72)	5	2436	3	1267	5.0%	0.87 [0.21, 3.62]
**Subtotal (95% CI)**		**2436**		**1267**	**5.0%**	**0.87 [0.21, 3.62]**
Total events	5		3			
Heterogeneity: Not applicable						
Test for overall effect: Z = 0.20 (P = 0.84)						
**10.31.5 Serious appendicitis: Cervarix vs. Havrix: hepatitis A vaccine (females, age 10-14; follow-up until 12 months)**						
HPV-013: Cervarix vs. Havrix (f, 10-14; 12)	0	1035	5	1032	1.2%	0.09 [0.01, 1.64]
**Subtotal (95% CI)**		**1035**		**1032**	**1.2%**	**0.09 [0.01, 1.64]**
Total events	0		5			
Heterogeneity: Not applicable						
Test for overall effect: Z = 1.63 (P = 0.10)						
**10.31.6 Serious appendicitis: Cervarix vs. Havrix: hepatitis A vaccine (females, age 15-25; follow-up until 48 months)**						
HPV-008: Cervarix vs. Havrix (f, 15-25; 48)	46	9319	59	9325	68.5%	0.78 [0.53, 1.15]
**Subtotal (95% CI)**		**9319**		**9325**	**68.5%**	**0.78 [0.53, 1.15]**
Total events	46		59			
Heterogeneity: Not applicable						
Test for overall effect: Z = 1.27 (P = 0.21)						
**10.31.7 Serious appendicitis: Gardasil vs. carrier solution (females and males, age 9-16; follow-up until 18 months)**						
V501-018: Gardasil vs. carrier solu. (f&m,9-16;18)	1	1184	0	597	1.0%	1.51 [0.06, 37.11]
**Subtotal (95% CI)**		**1184**		**597**	**1.0%**	**1.51 [0.06, 37.11]**
Total events	1		0			
Heterogeneity: Not applicable						
Test for overall effect: Z = 0.25 (P = 0.80)						
**10.31.8 Serious appendicitis: Gardasil vs. AAHS (females, age 15-26; follow-up until 45 months)**						
V501-013: Gardasil vs. AAHS (f, 16-24; 45)	1	2723	0	2732	1.0%	3.01 [0.12, 73.85]
V501-015: Gardasil vs. AAHS (f, 15-26; 36)	2	6087	0	6080	1.1%	4.99 [0.24, 104.01]
**Subtotal (95% CI)**		**8810**		**8812**	**2.1%**	**3.93 [0.43, 35.55]**
Total events	3		0			
Heterogeneity: $Tau^2 = 0.00$; $Chi^2 = 0.05$, df = 1 (P = 0.82); $I^2 = 0\%$						
Test for overall effect: Z = 1.22 (P = 0.22)						
**10.31.9 Serious appendicitis: Gardasil vs. AAHS (males, age 15-27; follow-up until 36 months)**						
V501-020: Gardasil vs. AAHS (m, 15-27; 36)	1	2032	0	2033	1.0%	3.00 [0.12, 73.64]
**Subtotal (95% CI)**		**2032**		**2033**	**1.0%**	**3.00 [0.12, 73.64]**
Total events	1		0			
Heterogeneity: Not applicable						
Test for overall effect: Z = 0.67 (P = 0.50)						
**10.31.10 Serious appendicitis: Gardasil 9 vs. saline placebo (females, age 12-26; follow-up until 7 months)**						
V503-006: Gardasil 9 vs. saline (f, 12-26;7)	1	618	0	306	1.0%	1.49 [0.06, 36.42]
**Subtotal (95% CI)**		**618**		**306**	**1.0%**	**1.49 [0.06, 36.42]**
Total events	1		0			
Heterogeneity: Not applicable						
Test for overall effect: Z = 0.24 (P = 0.81)						
**10.31.11 Serious appendicitis: HPV 16 vaccine vs. AAHS (females, age 16-25; follow-up until 48 months)**						
V501-005: HPV 16 vaccine vs. AAHS (f, 16-25; 48)	0	1204	1	1205	1.0%	0.33 [0.01, 8.18]
**Subtotal (95% CI)**		**1204**		**1205**	**1.0%**	**0.33 [0.01, 8.18]**
Total events	0		1			
Heterogeneity: Not applicable						
Test for overall effect: Z = 0.67 (P = 0.50)						
**Total (95% CI)**		**30071**		**28003**	**100.0%**	**0.85 [0.62, 1.17]**
Total events	72		82			
Heterogeneity: $Tau^2 = 0.00$; $Chi^2 = 6.72$, df = 11 (P = 0.82); $I^2 = 0\%$						
Test for overall effect: Z = 0.97 (P = 0.33)						
Test for subgroup differences: $Chi^2 = 6.66$, df = 10 (P = 0.76), $I^2 = 0\%$						

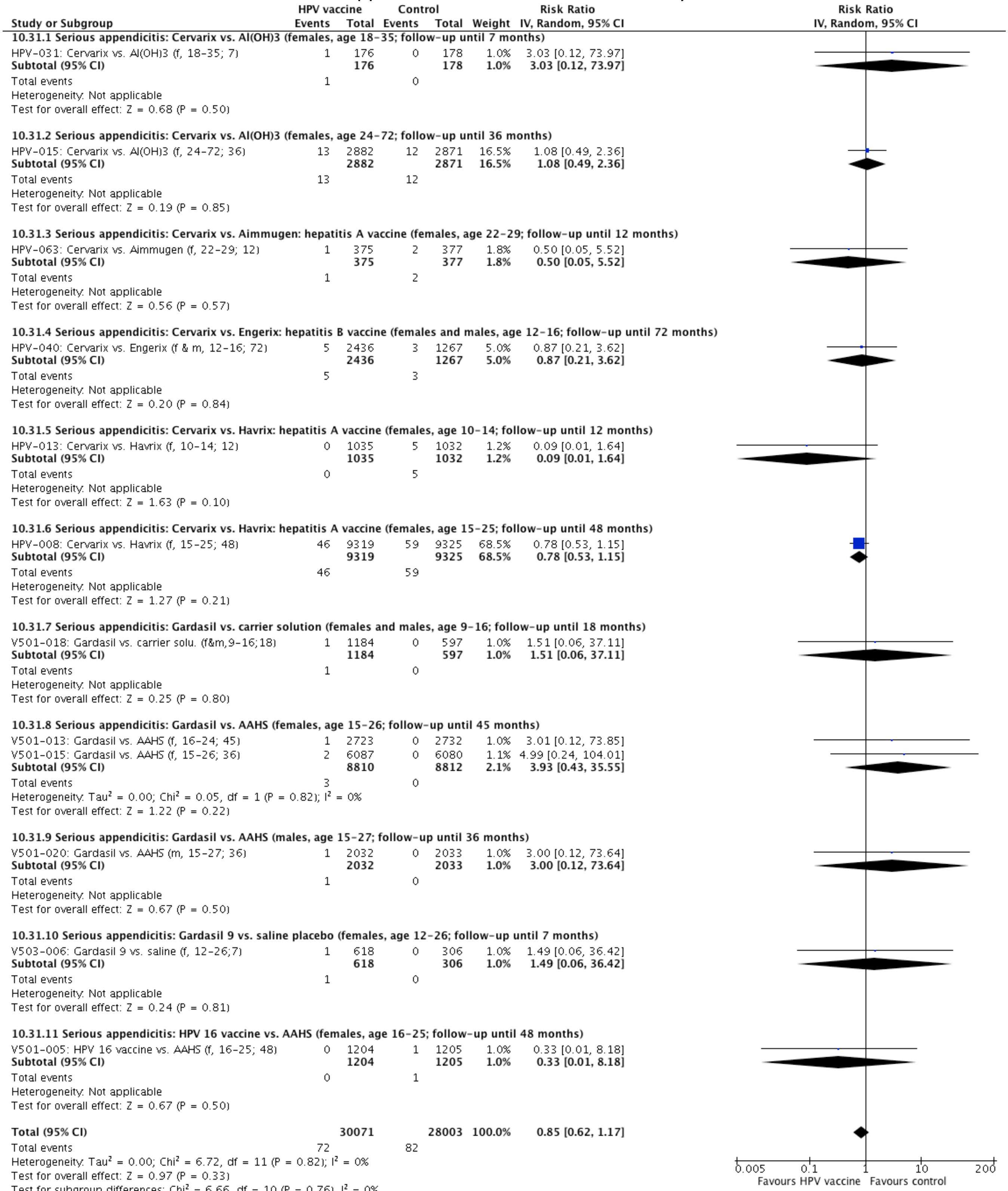

*10.31. Risk ratio for GlaxoSmithKline studies (i.e., HPV-0xx): 0.80 [0.58, 1.11]; risk ratio for Merck Sharp & Dohme studies (i.e., V50x-xxx): 1.83 [0.50, 6.68]. The risk ratio for all serious appendicitis categories (‘appendicitis’ and ‘appendicitis perforated’) is 0.84 [0.61, 1.15] (74/30,071 vs. 85/28,003).

10.32. Most common serious harms - ‘abortion spontaneous incomplete’*: intention to treat analysis

Study or Subgroup	HPV vaccine Events	HPV vaccine Total	Control Events	Control Total	Weight	Risk Ratio IV, Random, 95% CI
**10.32.1 Serious abortion spontaneous incomplete: Cervarix vs. Al(OH)3 (females, age 24-72; follow-up until 36 months)**						
HPV-015: Cervarix vs. Al(OH)3 (f, 24-72; 36)	18	2882	13	2871	24.3%	1.38 [0.68, 2.81]
**Subtotal (95% CI)**		**2882**		**2871**	**24.3%**	**1.38 [0.68, 2.81]**
Total events	18		13			
Heterogeneity: Not applicable						
Test for overall effect: Z = 0.89 (P = 0.38)						
**10.32.2 Serious abortion spontaneous incomplete: Cervarix vs. Aimmugen: hepatitis A vaccine (females, age 22-29; follow-up until 12 months)**						
HPV-063: Cervarix vs. Aimmugen (f, 22-29; 12)	1	375	1	377	1.6%	1.01 [0.06, 16.01]
**Subtotal (95% CI)**		**375**		**377**	**1.6%**	**1.01 [0.06, 16.01]**
Total events	1		1			
Heterogeneity: Not applicable						
Test for overall effect: Z = 0.00 (P = 1.00)						
**10.32.3 Serious abortion spontaneous incomplete: Cervarix vs. Havrix: hepatitis A vaccine (females, age 15-25; follow-up until 48 months)**						
HPV-008: Cervarix vs. Havrix (f, 15-25; 48)	54	9319	40	9325	74.1%	1.35 [0.90, 2.03]
**Subtotal (95% CI)**		**9319**		**9325**	**74.1%**	**1.35 [0.90, 2.03]**
Total events	54		40			
Heterogeneity: Not applicable						
Test for overall effect: Z = 1.45 (P = 0.15)						
**Total (95% CI)**		**12576**		**12573**	**100.0%**	**1.35 [0.95, 1.92]**
Total events	73		54			
Heterogeneity: $Tau^2 = 0.00$; $Chi^2 = 0.05$, df = 2 (P = 0.98); $I^2 = 0\%$						
Test for overall effect: Z = 1.68 (P = 0.09)						
Test for subgroup differences: $Chi^2 = 0.05$, df = 2 (P = 0.98), $I^2 = 0\%$						

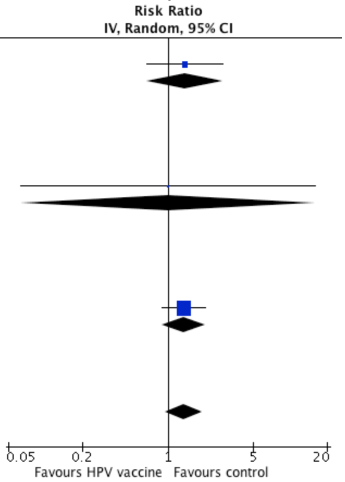

*10.32. Risk ratio for GlaxoSmithKline studies (i.e., HPV-0xx): not applicable; risk ratio for Merck Sharp & Dohme studies (i.e., V50x-xxx): 1.35 [0.95, 1.92]. The total risk ratio for all included serious abortion categories (‘abortion complete,’ ‘abortion incomplete,’ ‘abortion missed,’ ‘abortion spontaneous,’ ‘abortion spontaneous complete,’ ‘abortion spontaneous incomplete’ and ‘abortion threatened’) is 1.08 [0.91, 1.28] (271/13,271 vs. 254/13,272).

10.33. Most common serious harms - ‘abortion spontaneous complete’*: intention to treat analysis

Study or Subgroup	HPV vaccine Events	HPV vaccine Total	Control Events	Control Total	Weight	Risk Ratio IV, Random, 95% CI
**10.33.1 Serious abortion spontaneous complete: Cervarix vs. Al(OH)3 (females, age 24-72; follow-up until 36 months)**						
HPV-015: Cervarix vs. Al(OH)3 (f, 24-72; 36)	19	2882	16	2871	27.8%	1.18 [0.61, 2.30]
**Subtotal (95% CI)**		**2882**		**2871**	**27.8%**	**1.18 [0.61, 2.30]**
Total events	19		16			
Heterogeneity: Not applicable						
Test for overall effect: Z = 0.50 (P = 0.62)						
**10.33.2 Serious abortion spontaneous complete: Cervarix vs. Aimmugen: hepatitis A vaccine (females, age 22-29; follow-up until 12 months)**						
HPV-063: Cervarix vs. Aimmugen (f, 22-29; 12)	1	375	0	377	1.2%	3.02 [0.12, 73.80]
**Subtotal (95% CI)**		**375**		**377**	**1.2%**	**3.02 [0.12, 73.80]**
Total events	1		0			
Heterogeneity: Not applicable						
Test for overall effect: Z = 0.68 (P = 0.50)						
**10.33.3 Serious abortion spontaneous complete: Cervarix vs. Havrix: hepatitis A vaccine (females, age 15-25; follow-up until 48 months)**						
HPV-008: Cervarix vs. Havrix (f, 15-25; 48)	43	9319	46	9325	71.0%	0.94 [0.62, 1.42]
**Subtotal (95% CI)**		**9319**		**9325**	**71.0%**	**0.94 [0.62, 1.42]**
Total events	43		46			
Heterogeneity: Not applicable						
Test for overall effect: Z = 0.32 (P = 0.75)						
**Total (95% CI)**		**12576**		**12573**	**100.0%**	**1.01 [0.71, 1.44]**
Total events	63		62			
Heterogeneity: Tau² = 0.00; Chi² = 0.80, df = 2 (P = 0.67); I² = 0%						
Test for overall effect: Z = 0.07 (P = 0.94)						
Test for subgroup differences: Chi² = 0.80, df = 2 (P = 0.67), I² = 0%						

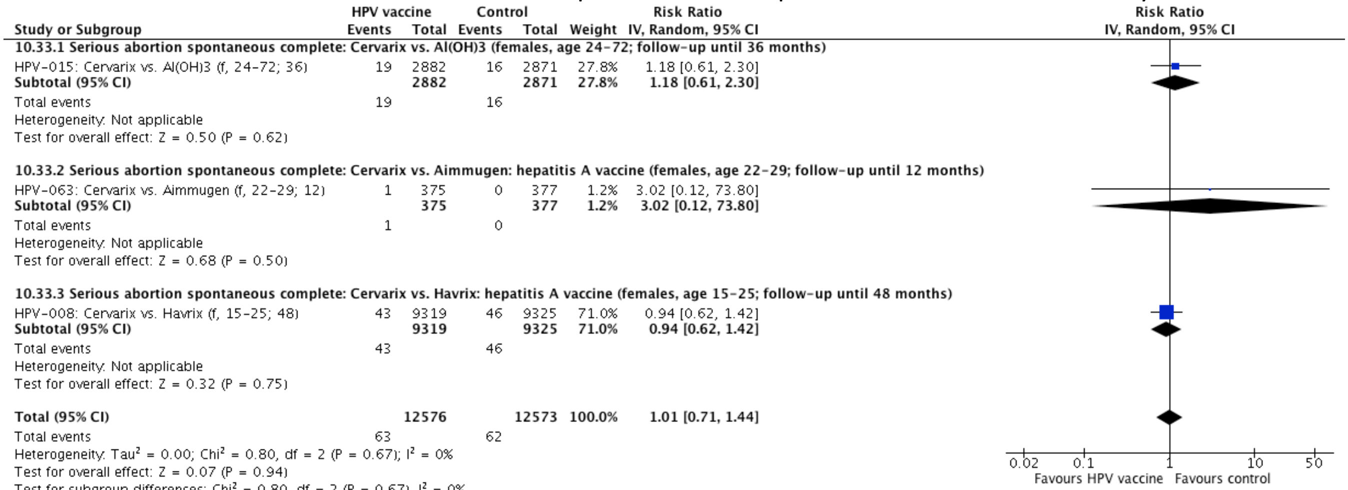

*10.33. Risk ratio for GlaxoSmithKline studies (i.e., HPV-0xx): 1.01 [0.71, 1.44]; risk ratio for Merck Sharp & Dohme studies (i.e., V50x-xxx): not applicable. The total risk ratio for all included serious abortion categories (‘abortion complete,’ ‘abortion incomplete,’ ‘abortion missed,’ ‘abortion spontaneous,’ ‘abortion spontaneous complete,’ ‘abortion spontaneous incomplete’ and ‘abortion threatened’) is 1.08 [0.91, 1.28] (271/13,271 vs. 254/13,272).

10.34. Most common serious harms - ‘abortion missed’*: intention to treat analysis

Study or Subgroup	HPV vaccine Events	HPV vaccine Total	Control Events	Control Total	Weight	Risk Ratio IV, Random, 95% CI
**10.34.1 Serious abortion missed: Cervarix vs. Al(OH)3 (females, age 24-72; follow-up until 36 months)**						
HPV-015: Cervarix vs. Al(OH)3 (f, 24-72; 36)	10	2882	11	2871	28.7%	0.91 [0.39, 2.13]
**Subtotal (95% CI)**		**2882**		**2871**	**28.7%**	**0.91 [0.39, 2.13]**
Total events	10		11			
Heterogeneity: Not applicable						
Test for overall effect: Z = 0.23 (P = 0.82)						
**10.34.2 Serious abortion missed: Cervarix vs. Aimmugen: hepatitis A vaccine (females, age 22-29; follow-up until 12 months)**						
HPV-063: Cervarix vs. Aimmugen (f, 22-29; 12)	3	375	3	377	8.3%	1.01 [0.20, 4.95]
**Subtotal (95% CI)**		**375**		**377**	**8.3%**	**1.01 [0.20, 4.95]**
Total events	3		3			
Heterogeneity: Not applicable						
Test for overall effect: Z = 0.01 (P = 0.99)						
**10.34.3 Serious abortion missed: Cervarix vs. Havrix: hepatitis A vaccine (females, age 15-25; follow-up until 48 months)**						
HPV-008: Cervarix vs. Havrix (f, 15-25; 48)	20	9319	27	9325	63.0%	0.74 [0.42, 1.32]
**Subtotal (95% CI)**		**9319**		**9325**	**63.0%**	**0.74 [0.42, 1.32]**
Total events	20		27			
Heterogeneity: Not applicable						
Test for overall effect: Z = 1.02 (P = 0.31)						
**Total (95% CI)**		**12576**		**12573**	**100.0%**	**0.81 [0.51, 1.27]**
Total events	33		41			
Heterogeneity: Tau^2 = 0.00; Chi^2 = 0.23, df = 2 (P = 0.89); I^2 = 0%						
Test for overall effect: Z = 0.93 (P = 0.35)						
Test for subgroup differences: Chi^2 = 0.23, df = 2 (P = 0.89), I^2 = 0%						

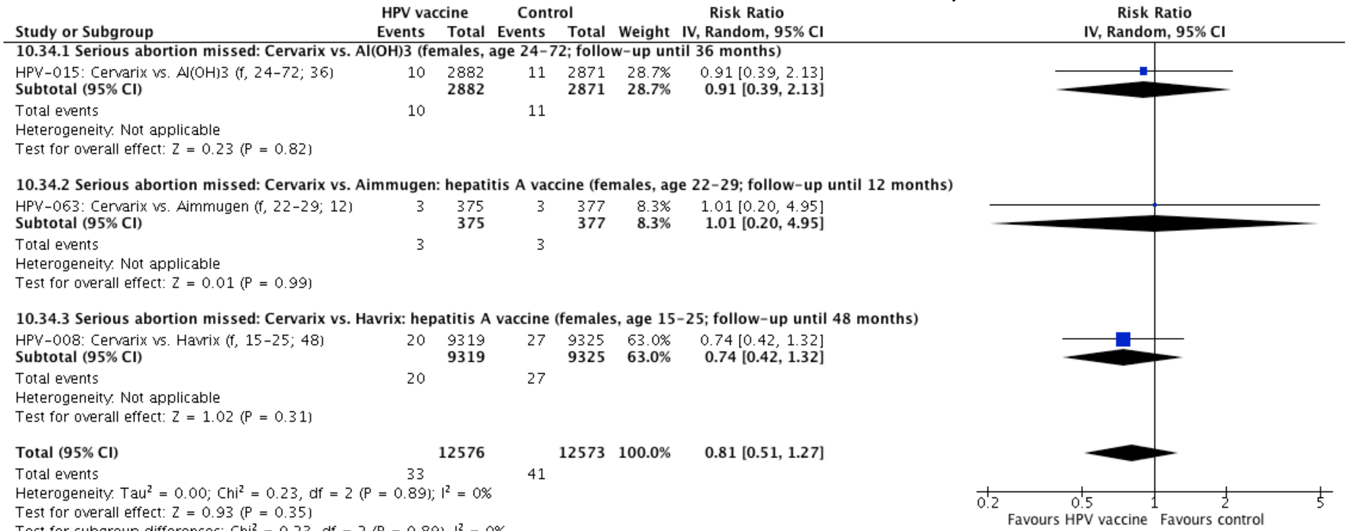

*10.34. Risk ratio for GlaxoSmithKline studies (i.e., HPV-0xx): 0.81 [0.51, 1.27]; risk ratio for Merck Sharp & Dohme studies (i.e., V50x-xxx): not applicable. The total risk ratio for all included serious abortion categories (‘abortion complete,’ ‘abortion incomplete,’ ‘abortion missed,’ ‘abortion spontaneous,’ ‘abortion spontaneous complete,’ ‘abortion spontaneous incomplete’ and ‘abortion threatened’) is 1.08 [0.91, 1.28] (271/13,271 vs. 254/13,272).

10.35. Serious harms most increased by the HPV vaccines - ‘abortion spontaneous incomplete’*: intention to treat analysis

See analysis 10.32.

10.36.Serious harms most increased by the HPV vaccines - 'pyelonephritis'*: intention to treat analysis

Study or Subgroup	HPV vaccine Events	HPV vaccine Total	Control Events	Control Total	Weight	Risk Ratio IV, Random, 95% CI
**10.36.1 Serious pyelonephritis: Cervarix vs. Al(OH)3 (females, age 24-72; follow-up until 36 months)**						
HPV-015: Cervarix vs. Al(OH)3 (f, 24-72; 36)	6	2882	1	2871	8.0%	5.98 [0.72, 49.62]
**Subtotal (95% CI)**		**2882**		**2871**	**8.0%**	**5.98 [0.72, 49.62]**
Total events	6		1			
Heterogeneity: Not applicable						
Test for overall effect: Z = 1.66 (P = 0.10)						
**10.36.2 Serious pyelonephritis: Cervarix vs. Havrix: hepatitis A vaccine (females, age 15-25; follow-up until 48 months)**						
HPV-008: Cervarix vs. Havrix (f, 15-25; 48)	22	9319	13	9325	76.4%	1.69 [0.85, 3.36]
**Subtotal (95% CI)**		**9319**		**9325**	**76.4%**	**1.69 [0.85, 3.36]**
Total events	22		13			
Heterogeneity: Not applicable						
Test for overall effect: Z = 1.51 (P = 0.13)						
**10.36.3 Serious pyelonephritis: Gardasil vs. AAHS (females, age 15-26; follow-up until 45 months)**						
V501-013: Gardasil vs. AAHS (f, 16-24; 45)	2	2732	0	2732	3.9%	5.00 [0.24, 104.10]
V501-015: Gardasil vs. AAHS (f, 15-26; 36)	0	6087	1	6080	3.5%	0.33 [0.01, 8.17]
**Subtotal (95% CI)**		**8819**		**8812**	**7.4%**	**1.36 [0.10, 19.25]**
Total events	2		1			
Heterogeneity: Tau² = 1.14; Chi² = 1.45, df = 1 (P = 0.23); I² = 31%						
Test for overall effect: Z = 0.22 (P = 0.82)						
**10.36.4 Serious pyelonephritis: Gardasil vs. AAHS (females, age 21-46; follow-up until 48 months)**						
V501-019: Gardasil vs. AAHS (f, 21-46; 48)	0	1911	1	1908	3.5%	0.33 [0.01, 8.16]
**Subtotal (95% CI)**		**1911**		**1908**	**3.5%**	**0.33 [0.01, 8.16]**
Total events	0		1			
Heterogeneity: Not applicable						
Test for overall effect: Z = 0.67 (P = 0.50)						
**10.36.5 Serious pyelonephritis: HPV 16 vaccine vs. AAHS (females, age 16-25; follow-up until 48 months)**						
V501-005: HPV 16 vaccine vs. AAHS (f, 16-25; 48)	1	1204	1	1205	4.7%	1.00 [0.06, 15.98]
**Subtotal (95% CI)**		**1204**		**1205**	**4.7%**	**1.00 [0.06, 15.98]**
Total events	1		1			
Heterogeneity: Not applicable						
Test for overall effect: Z = 0.00 (P = 1.00)						
**Total (95% CI)**		**24135**		**24121**	**100.0%**	**1.70 [0.93, 3.10]**
Total events	31		17			
Heterogeneity: Tau² = 0.00; Chi² = 3.98, df = 5 (P = 0.55); I² = 0%						
Test for overall effect: Z = 1.74 (P = 0.08)						
Test for subgroup differences: Chi² = 2.52, df = 4 (P = 0.64), I² = 0%						

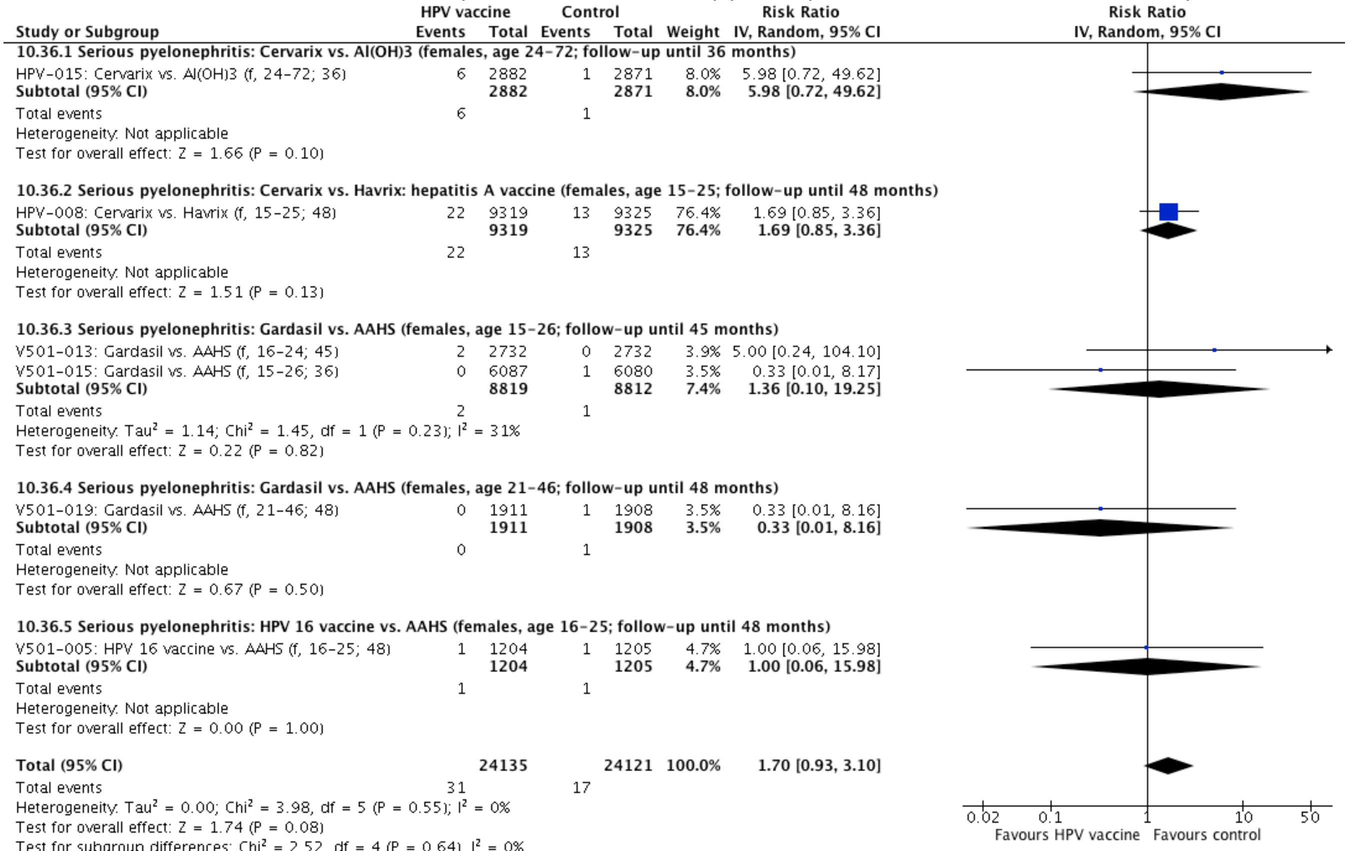

*10.36. Risk ratio for GlaxoSmithKline studies (i.e., HPV-0xx): 2.10 [0.83, 5.34]; risk ratio for Merck Sharp & Dohme studies (i.e., V50x-xxx): 0.91 [0.20, 4.16]. The risk ratio of 'pyelonephritis' together with 'pyelonephritis acute' is 1.56 [0.72, 3.35] (53/21,410 vs. 32/24,498)

10.37. Serious harms most increased by the HPV vaccines - ‘abortion spontaneous’*: intention to treat analysis

See analysis 10.30.

10.38.Serious harms most increased by the HPV vaccines - ‘pneumonia’*: intention to treat analysis

Study or Subgroup	HPV vaccine Events	HPV vaccine Total	Control Events	Control Total	Weight	Risk Ratio IV, Random, 95% CI
**10.38.1 Serious pneumonia: Cervarix vs. Al(OH)3 (females, age 24-72; follow-up until 36 months)**						
HPV-015: Cervarix vs. Al(OH)3 (f, 24-72; 36)	2	2882	1	2871	7.0%	1.99 [0.18, 21.96]
**Subtotal (95% CI)**		**2882**		**2871**	**7.0%**	**1.99 [0.18, 21.96]**
Total events	2		1			
Heterogeneity: Not applicable						
Test for overall effect: Z = 0.56 (P = 0.57)						
**10.38.2 Serious pneumonia: Cervarix vs. Aimmugen: hepatitis A vaccine (females, age 22-29; follow-up until 12 months)**						
HPV-063: Cervarix vs. Aimmugen (f, 22-29; 12)	0	375	1	377	3.9%	0.34 [0.01, 8.20]
**Subtotal (95% CI)**		**375**		**377**	**3.9%**	**0.34 [0.01, 8.20]**
Total events	0		1			
Heterogeneity: Not applicable						
Test for overall effect: Z = 0.67 (P = 0.50)						
**10.38.3 Serious pneumonia: Cervarix vs. Engerix: hepatitis B vaccine (females and males, age 12-16; follow-up until 72 months)**						
HPV-040: Cervarix vs. Engerix (f & m, 12-16; 72)	2	2436	0	1267	4.4%	2.60 [0.12, 54.15]
**Subtotal (95% CI)**		**2436**		**1267**	**4.4%**	**2.60 [0.12, 54.15]**
Total events	2		0			
Heterogeneity: Not applicable						
Test for overall effect: Z = 0.62 (P = 0.54)						
**10.38.4 Serious pneumonia: Cervarix vs. Havrix: hepatitis A vaccine (females, age 15-25; follow-up until 48 months)**						
HPV-008: Cervarix vs. Havrix (f, 15-25; 48)	20	9319	10	9325	69.9%	2.00 [0.94, 4.27]
**Subtotal (95% CI)**		**9319**		**9325**	**69.9%**	**2.00 [0.94, 4.27]**
Total events	20		10			
Heterogeneity: Not applicable						
Test for overall effect: Z = 1.79 (P = 0.07)						
**10.38.5 Serious pneumonia: Gardasil vs. AAHS (females, age 15-26; follow-up until 36 months)**						
V501-015: Gardasil vs. AAHS (f, 15-26; 36)	2	6087	1	6080	7.0%	2.00 [0.18, 22.03]
**Subtotal (95% CI)**		**6087**		**6080**	**7.0%**	**2.00 [0.18, 22.03]**
Total events	2		1			
Heterogeneity: Not applicable						
Test for overall effect: Z = 0.57 (P = 0.57)						
**10.38.6 Serious pneumonia: Gardasil vs. AAHS (females, age 21-46; follow-up until 48 months)**						
V501-019: Gardasil vs. AAHS (f, 21-46; 48)	0	1911	1	1908	3.9%	0.33 [0.01, 8.16]
**Subtotal (95% CI)**		**1911**		**1908**	**3.9%**	**0.33 [0.01, 8.16]**
Total events	0		1			
Heterogeneity: Not applicable						
Test for overall effect: Z = 0.67 (P = 0.50)						
**10.38.7 Serious pneumonia: HPV 16 vaccine vs. AAHS (females, age 16-25; follow-up until 48 months)**						
V501-005: HPV 16 vaccine vs. AAHS (f, 16-25; 48)	0	1204	1	1205	3.9%	0.33 [0.01, 8.18]
**Subtotal (95% CI)**		**1204**		**1205**	**3.9%**	**0.33 [0.01, 8.18]**
Total events	0		1			
Heterogeneity: Not applicable						
Test for overall effect: Z = 0.67 (P = 0.50)						
**Total (95% CI)**		**24214**		**23033**	**100.0%**	**1.64 [0.87, 3.09]**
Total events	26		15			
Heterogeneity: Tau² = 0.00; Chi² = 3.26, df = 6 (P = 0.78); I² = 0%						
Test for overall effect: Z = 1.53 (P = 0.13)						
Test for subgroup differences: Chi² = 3.26, df = 6 (P = 0.78), I² = 0%						

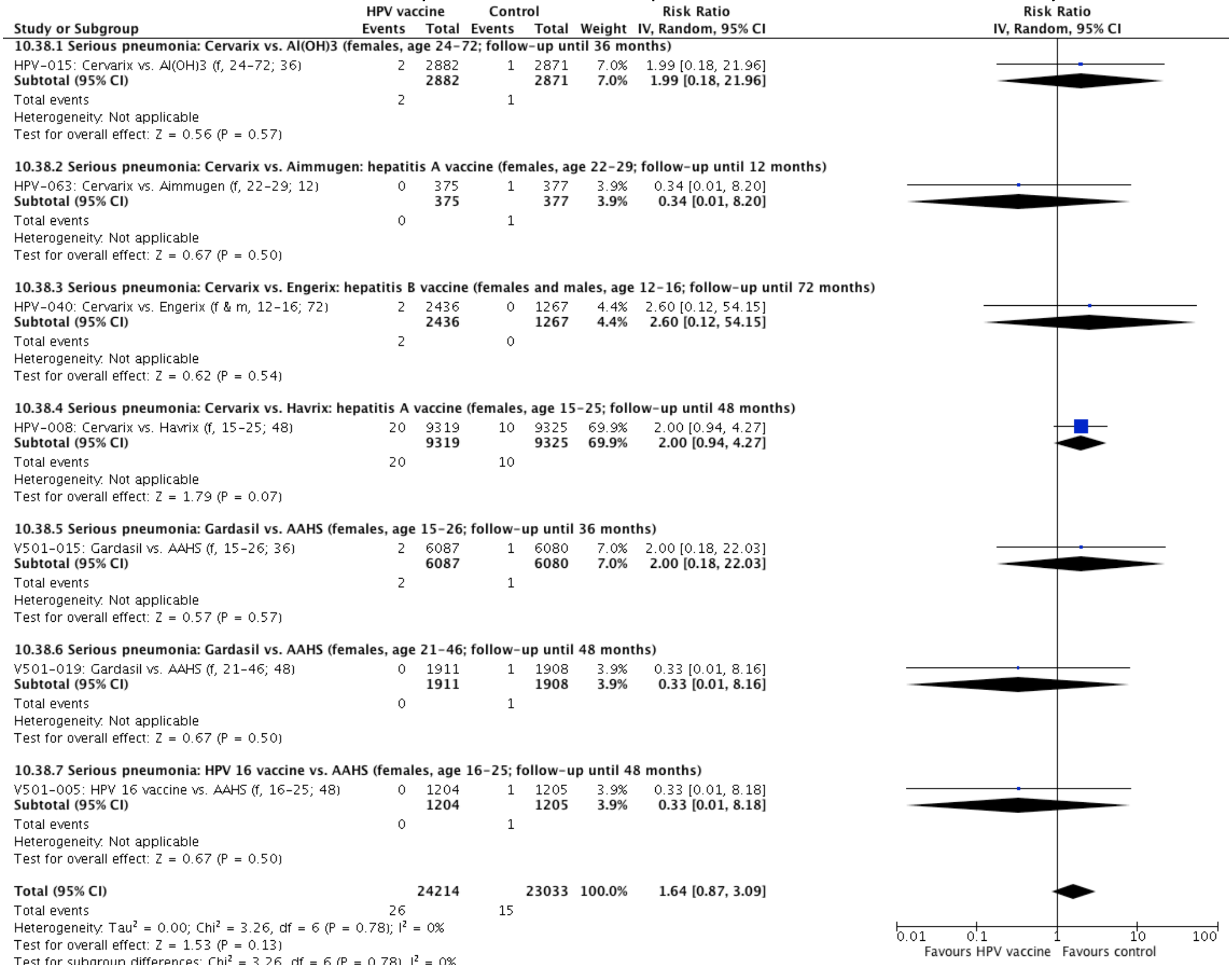

*10.38. Risk ratio for GlaxoSmithKline studies (i.e., HPV-0xx): 1.87 [0.94, 3.71]; risk ratio for Merck Sharp & Dohme studies (i.e., V50x-xxx): 0.77 [0.15, 4.02]. The risk ratio of all serious pneumonia categories (‘pneumonia,’ ‘pneumonia bacterial,’ ‘pneumonia influenza,’ ‘pneumonia mycoplasmal’ and ‘pneumonia streptococcal’) is 1.26 [0.72, 2.23] (29/26,946 vs. 22/25,765).

10.39. Serious harms most increased by the HPV vaccines - ‘tonsillitis’*: intention to treat analysis

	HPV vaccine		Control			Risk Ratio
**Study or Subgroup**	**Events**	**Total**	**Events**	**Total**	**Weight**	**IV, Random, 95% CI**
**10.39.1 Serious tonsillitis: Cervarix vs. Al(OH)3 (females, age 24-72; follow-up until 36 months)**						
HPV-015: Cervarix vs. Al(OH)3 (f, 24-72; 36)	0	2882	1	2871	6.1%	0.33 [0.01, 8.15]
**Subtotal (95% CI)**		**2882**		**2871**	**6.1%**	**0.33 [0.01, 8.15]**
Total events	0		1			
Heterogeneity: Not applicable						
Test for overall effect: Z = 0.68 (P = 0.50)						
**10.39.2 Serious tonsillitis: Cervarix vs. Engerix: hepatitis B vaccine (females and males, age 12-16; follow-up until 72 months)**						
HPV-040: Cervarix vs. Engerix (f & m, 12-16; 72)	4	2436	0	1267	7.3%	4.68 [0.25, 86.91]
**Subtotal (95% CI)**		**2436**		**1267**	**7.3%**	**4.68 [0.25, 86.91]**
Total events	4		0			
Heterogeneity: Not applicable						
Test for overall effect: Z = 1.04 (P = 0.30)						
**10.39.3 Serious tonsillitis: Cervarix vs. Havrix: hepatitis A vaccine (females, age 15-25; follow-up until 48 months)**						
HPV-008: Cervarix vs. Havrix (f, 15-25; 48)	13	9319	8	9325	80.5%	1.63 [0.67, 3.92]
**Subtotal (95% CI)**		**9319**		**9325**	**80.5%**	**1.63 [0.67, 3.92]**
Total events	13		8			
Heterogeneity: Not applicable						
Test for overall effect: Z = 1.08 (P = 0.28)						
**10.39.4 Serious tonsillitis: Gardasil 9 vs. saline placebo (females, age 12-26; follow-up until 7 months)**						
V503-006: Gardasil 9 vs. saline (f, 12-26;7)	1	618	0	306	6.1%	1.49 [0.06, 36.42]
**Subtotal (95% CI)**		**618**		**306**	**6.1%**	**1.49 [0.06, 36.42]**
Total events	1		0			
Heterogeneity: Not applicable						
Test for overall effect: Z = 0.24 (P = 0.81)						
**Total (95% CI)**		**15255**		**13769**	**100.0%**	**1.59 [0.72, 3.49]**
Total events	18		9			
Heterogeneity: $Tau^2 = 0.00; Chi^2 = 1.45, df = 3 (P = 0.69); I^2 = 0\%$						
Test for overall effect: Z = 1.14 (P = 0.25)						
Test for subgroup differences: $Chi^2 = 1.45, df = 3 (P = 0.69), I^2 = 0\%$						

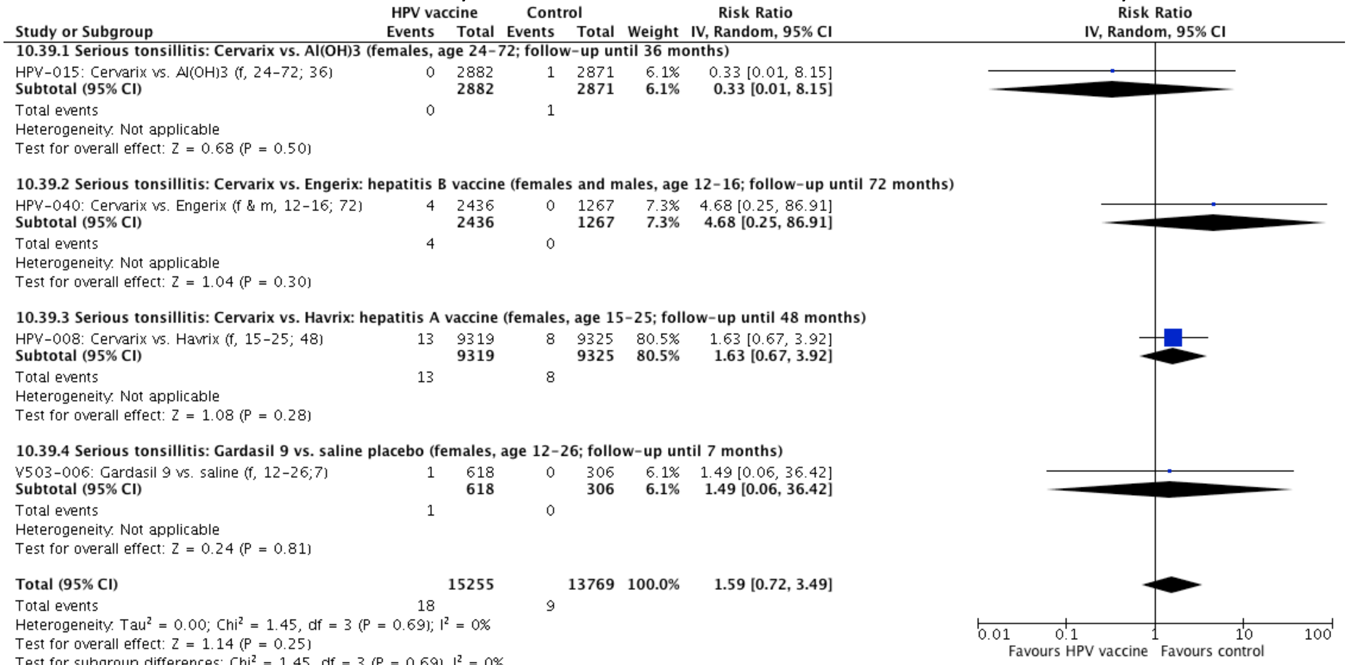

*10.38. Risk ratio for GlaxoSmithKline studies (i.e., HPV-0xx): 1.59 [0.70, 3.60]; risk ratio for Merck Sharp & Dohme studies (i.e., V50x-xxx): 1.49 [0.06, 36.42]. The risk ratio of all serious tonsillitis categories (‘tonsillitis’ and ‘tonsillitis streptococcal’) is 1.54 [0.71, 3.33] (19/17,987 vs. 10/16,501).

10.40. Serious harms most decreased by the HPV vaccines - ‘appendicitis’*: intention to treat analysis

See analysis 10.31.

10.41. Serious harms most decreased by the HPV vaccines - ‘overdose’*: intention to treat analysis

Study or Subgroup	HPV vaccine Events	HPV vaccine Total	Control Events	Control Total	Weight	Risk Ratio IV, Random, 95% CI
**10.41.1 Serious overdose: Cervarix vs. Al(OH)3 (females, age 24-72; follow-up until 36 months)**						
HPV-015: Cervarix vs. Al(OH)3 (f, 24-72; 36)	0	2882	1	2871	2.9%	0.33 [0.01, 8.15]
**Subtotal (95% CI)**		**2882**		**2871**	**2.9%**	**0.33 [0.01, 8.15]**
Total events	0		1			
Heterogeneity: Not applicable						
Test for overall effect: Z = 0.68 (P = 0.50)						
**10.41.2 Serious overdose: Cervarix vs. Havrix: hepatitis A vaccine (females, age 15-25; follow-up until 48 months)**						
HPV-008: Cervarix vs. Havrix (f, 15-25; 48)	3	9319	3	9325	11.4%	1.00 [0.20, 4.96]
**Subtotal (95% CI)**		**9319**		**9325**	**11.4%**	**1.00 [0.20, 4.96]**
Total events	3		3			
Heterogeneity: Not applicable						
Test for overall effect: Z = 0.00 (P = 1.00)						
**10.41.3 Serious overdose: Gardasil vs. AAHS (females, age 15-26; follow-up until 45 months)**						
V501-013: Gardasil vs. AAHS (f, 16-24; 45)	17	2723	26	2732	79.0%	0.66 [0.36, 1.21]
V501-015: Gardasil vs. AAHS (f, 15-26; 36)	1	6087	0	6080	2.9%	3.00 [0.12, 73.54]
**Subtotal (95% CI)**		**8810**		**8812**	**81.9%**	**0.69 [0.38, 1.26]**
Total events	18		26			
Heterogeneity: Tau² = 0.00; Chi² = 0.84, df = 1 (P = 0.36); I² = 0%						
Test for overall effect: Z = 1.21 (P = 0.23)						
**10.41.4 Serious overdose: HPV 16 vaccine vs. AAHS (females, age 16-25; follow-up until 48 months)**						
V501-005: HPV 16 vaccine vs. AAHS (f, 16-25; 48)	1	1204	1	1205	3.8%	1.00 [0.06, 15.98]
**Subtotal (95% CI)**		**1204**		**1205**	**3.8%**	**1.00 [0.06, 15.98]**
Total events	1		1			
Heterogeneity: Not applicable						
Test for overall effect: Z = 0.00 (P = 1.00)						
**Total (95% CI)**		**22215**		**22213**	**100.0%**	**0.72 [0.42, 1.23]**
Total events	22		31			
Heterogeneity: Tau² = 0.00; Chi² = 1.29, df = 4 (P = 0.86); I² = 0%						
Test for overall effect: Z = 1.21 (P = 0.23)						
Test for subgroup differences: Chi² = 0.46, df = 3 (P = 0.93), I² = 0%						

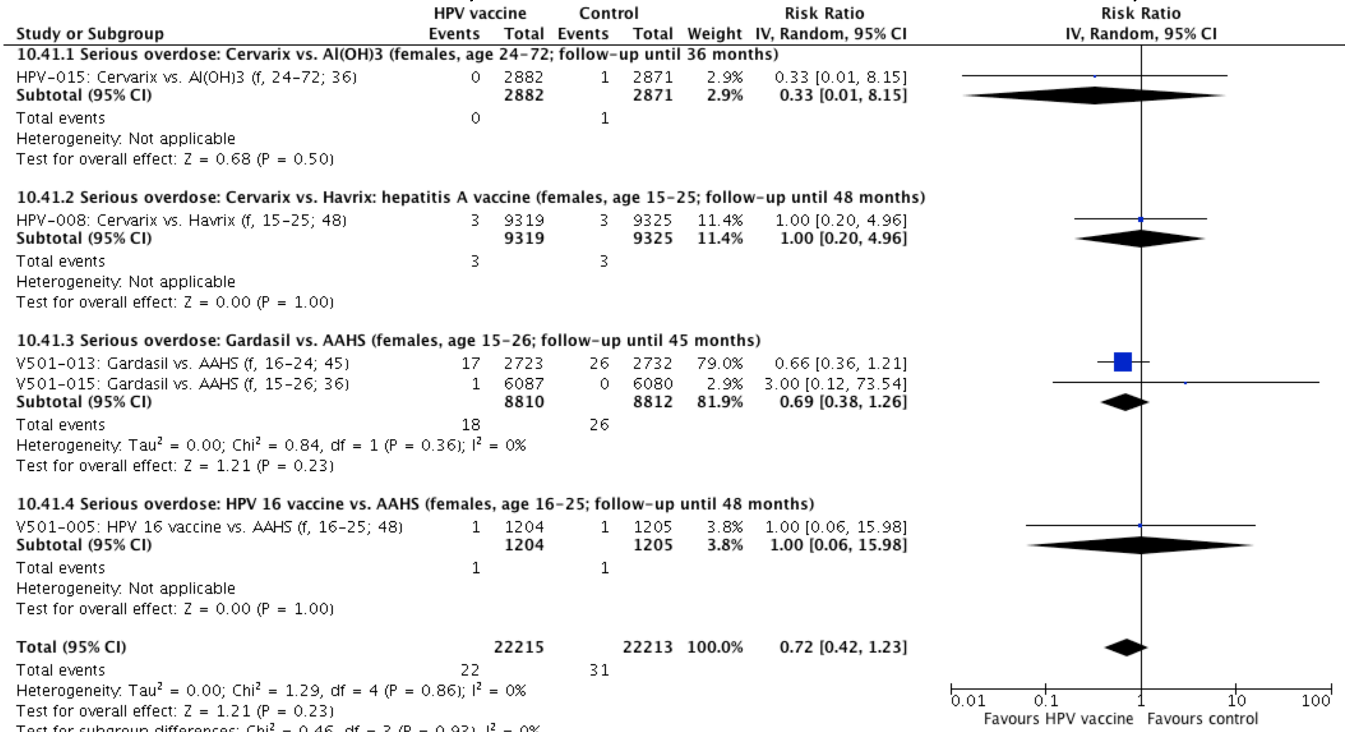

*10.41. Risk ratio for GlaxoSmithKline studies (i.e., HPV-0xx): 0.80 [0.19, 3.36]; risk ratio for Merck Sharp & Dohme studies (i.e., V50x-xxx): 0.70 [0.39, 1.26].

10.42. Serious harms most decreased by the HPV vaccines - ‘abortion missed’*: intention to treat analysis

Study or Subgroup	HPV vaccine Events	HPV vaccine Total	Control Events	Control Total	Weight	Risk Ratio IV, Random, 95% CI
**10.42.1 Serious abortion missed: Cervarix vs. Al(OH)3 (females, age 24-72; follow-up until 36 months)**						
HPV-015: Cervarix vs. Al(OH)3 (f, 24-72; 36)	10	2882	11	2871	28.7%	0.91 [0.39, 2.13]
**Subtotal (95% CI)**		**2882**		**2871**	**28.7%**	**0.91 [0.39, 2.13]**
Total events	10		11			
Heterogeneity: Not applicable						
Test for overall effect: Z = 0.23 (P = 0.82)						
**10.42.2 Serious abortion missed: Cervarix vs. Aimmugen: hepatitis A vaccine (females, age 22-29; follow-up until 12 months)**						
HPV-063: Cervarix vs. Aimmugen (f, 22-29; 12)	3	375	3	377	8.3%	1.01 [0.20, 4.95]
**Subtotal (95% CI)**		**375**		**377**	**8.3%**	**1.01 [0.20, 4.95]**
Total events	3		3			
Heterogeneity: Not applicable						
Test for overall effect: Z = 0.01 (P = 0.99)						
**10.42.3 Serious abortion missed: Cervarix vs. Havrix: hepatitis A vaccine (females, age 15-25; follow-up until 48 months)**						
HPV-008: Cervarix vs. Havrix (f, 15-25; 48)	20	9319	27	9325	63.0%	0.74 [0.42, 1.32]
**Subtotal (95% CI)**		**9319**		**9325**	**63.0%**	**0.74 [0.42, 1.32]**
Total events	20		27			
Heterogeneity: Not applicable						
Test for overall effect: Z = 1.02 (P = 0.31)						
**Total (95% CI)**		**12576**		**12573**	**100.0%**	**0.81 [0.51, 1.27]**
Total events	33		41			
Heterogeneity: $Tau^2 = 0.00$; $Chi^2 = 0.23$, df = 2 (P = 0.89); $I^2 = 0\%$						
Test for overall effect: Z = 0.93 (P = 0.35)						
Test for subgroup differences: $Chi^2 = 0.23$, df = 2 (P = 0.89), $I^2 = 0\%$						

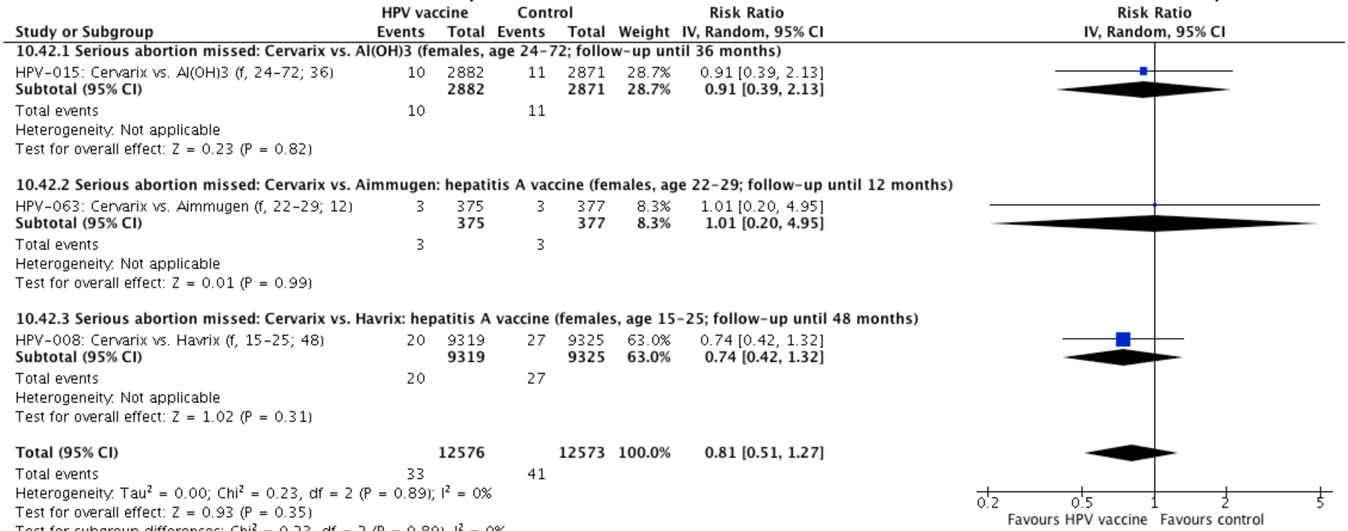

*10.42. Risk ratio for GlaxoSmithKline studies (i.e., HPV-0xx): 0.81 [0.51, 1.27]; risk ratio for Merck Sharp & Dohme studies (i.e., V50x-xxx): not applicable. The total risk ratio for all included serious abortion categories (‘abortion complete,’ ‘abortion incomplete,’ ‘abortion missed,’ ‘abortion spontaneous,’ ‘abortion spontaneous complete,’ ‘abortion spontaneous incomplete’ and ‘abortion threatened’) is 1.08 [0.91, 1.28] (271/13,271 vs. 254/13,272).

10.43. Serious harms most decreased by the HPV vaccines - ‘ligament rupture’*: intention to treat analysis

Study or Subgroup	HPV vaccine Events	HPV vaccine Total	Control Events	Control Total	Weight	Risk Ratio IV, Random, 95% CI
**10.43.1 Serious ligament rupture: Cervarix vs. Al(OH)3 (females, age 24-72; follow-up until 36 months)**						
HPV-015: Cervarix vs. Al(OH)3 (f, 24-72; 36)	2	2882	1	2871	19.9%	1.99 [0.18, 21.96]
**Subtotal (95% CI)**		**2882**		**2871**	**19.9%**	**1.99 [0.18, 21.96]**
Total events	2		1			
Heterogeneity: Not applicable						
Test for overall effect: Z = 0.56 (P = 0.57)						
**10.43.2 Serious ligament rupture: Cervarix vs. Aimmugen: hepatitis A vaccine (females, age 22-29; follow-up until 12 months)**						
HPV-063: Cervarix vs. Aimmugen (f, 22-29; 12)	0	375	1	377	11.2%	0.34 [0.01, 8.20]
**Subtotal (95% CI)**		**375**		**377**	**11.2%**	**0.34 [0.01, 8.20]**
Total events	0		1			
Heterogeneity: Not applicable						
Test for overall effect: Z = 0.67 (P = 0.50)						
**10.43.3 Serious ligament rupture: Cervarix vs. Havrix: hepatitis A vaccine (females, age 15-25; follow-up until 48 months)**						
HPV-008: Cervarix vs. Havrix (f, 15-25; 48)	3	9319	10	9325	68.9%	0.30 [0.08, 1.09]
**Subtotal (95% CI)**		**9319**		**9325**	**68.9%**	**0.30 [0.08, 1.09]**
Total events	3		10			
Heterogeneity: Not applicable						
Test for overall effect: Z = 1.83 (P = 0.07)						
**Total (95% CI)**		**12576**		**12573**	**100.0%**	**0.44 [0.15, 1.29]**
Total events	5		12			
Heterogeneity: $Tau^2 = 0.00; Chi^2 = 1.89, df = 2 (P = 0.39); I^2 = 0\%$						
Test for overall effect: Z = 1.49 (P = 0.14)						
Test for subgroup differences: $Chi^2 = 1.89, df = 2 (P = 0.39), I^2 = 0\%$						

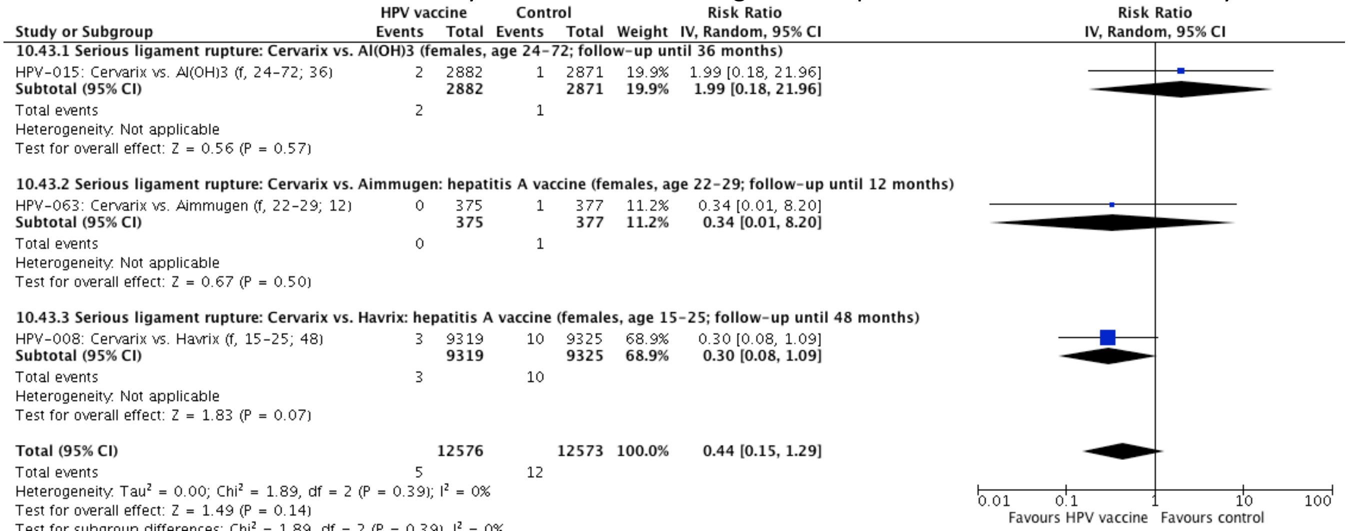

*10.43. Risk ratio for GlaxoSmithKline studies (i.e., HPV-0xx): 0.44 [0.15, 1.29]; risk ratio for Merck Sharp & Dohme studies (i.e., V50x-xxx): not applicable. Risk ratio for all serious ligament injury categories (‘ligament injury,’ ‘ligament rupture’ and ‘ligament sprain’): 0.51 [0.21, 1.29] (7/12,576 vs. 14/12,573).

10.44. Serious harms most decreased by the HPV vaccines - ‘ovarian cyst rupture’*: intention to treat analysis

Study or Subgroup	HPV vaccine Events	HPV vaccine Total	Control Events	Control Total	Weight	Risk Ratio IV, Random, 95% CI
**10.44.1 Serious ovarian cyst ruptured: Cervarix vs. Al(OH)3 (females, age 15-25; follow-up until 7 months)**						
HPV-038: Cervarix vs. Al(OH)3 (f, 15-25; 7)	1	149	0	76	9.0%	1.54 [0.06, 37.36]
**Subtotal (95% CI)**		**149**		**76**	**9.0%**	**1.54 [0.06, 37.36]**
Total events	1		0			
Heterogeneity: Not applicable						
Test for overall effect: Z = 0.27 (P = 0.79)						
**10.44.2 Serious ovarian cyst ruptured: Cervarix vs. Havrix: hepatitis A vaccine (females, age 10-14; follow-up until 12 months)**						
HPV-013: Cervarix vs. Havrix (f, 10-14; 12)	0	1035	1	1032	9.0%	0.33 [0.01, 8.15]
**Subtotal (95% CI)**		**1035**		**1032**	**9.0%**	**0.33 [0.01, 8.15]**
Total events	0		1			
Heterogeneity: Not applicable						
Test for overall effect: Z = 0.67 (P = 0.50)						
**10.44.3 Serious ovarian cyst ruptured: Cervarix vs. Havrix: hepatitis A vaccine (females, age 15-25; follow-up until 48 months)**						
HPV-008: Cervarix vs. Havrix (f, 15-25; 48)	4	9319	11	9325	70.1%	0.36 [0.12, 1.14]
**Subtotal (95% CI)**		**9319**		**9325**	**70.1%**	**0.36 [0.12, 1.14]**
Total events	4		11			
Heterogeneity: Not applicable						
Test for overall effect: Z = 1.73 (P = 0.08)						
**10.44.4 Serious ovarian cyst ruptured: HPV 16 vaccine vs. AAHS (females, age 16-25; follow-up until 48 months)**						
V501-005: HPV 16 vaccine vs. AAHS (f, 16-25; 48)	1	1204	1	1205	11.9%	1.00 [0.06, 15.98]
**Subtotal (95% CI)**		**1204**		**1205**	**11.9%**	**1.00 [0.06, 15.98]**
Total events	1		1			
Heterogeneity: Not applicable						
Test for overall effect: Z = 0.00 (P = 1.00)						
**Total (95% CI)**		**11707**		**11638**	**100.0%**	**0.46 [0.18, 1.21]**
Total events	6		13			
Heterogeneity: $Tau^2 = 0.00$; $Chi^2 = 1.05$, df = 3 (P = 0.79); $I^2 = 0\%$						
Test for overall effect: Z = 1.57 (P = 0.12)						
Test for subgroup differences: $Chi^2 = 1.05$, df = 3 (P = 0.79), $I^2 = 0\%$						

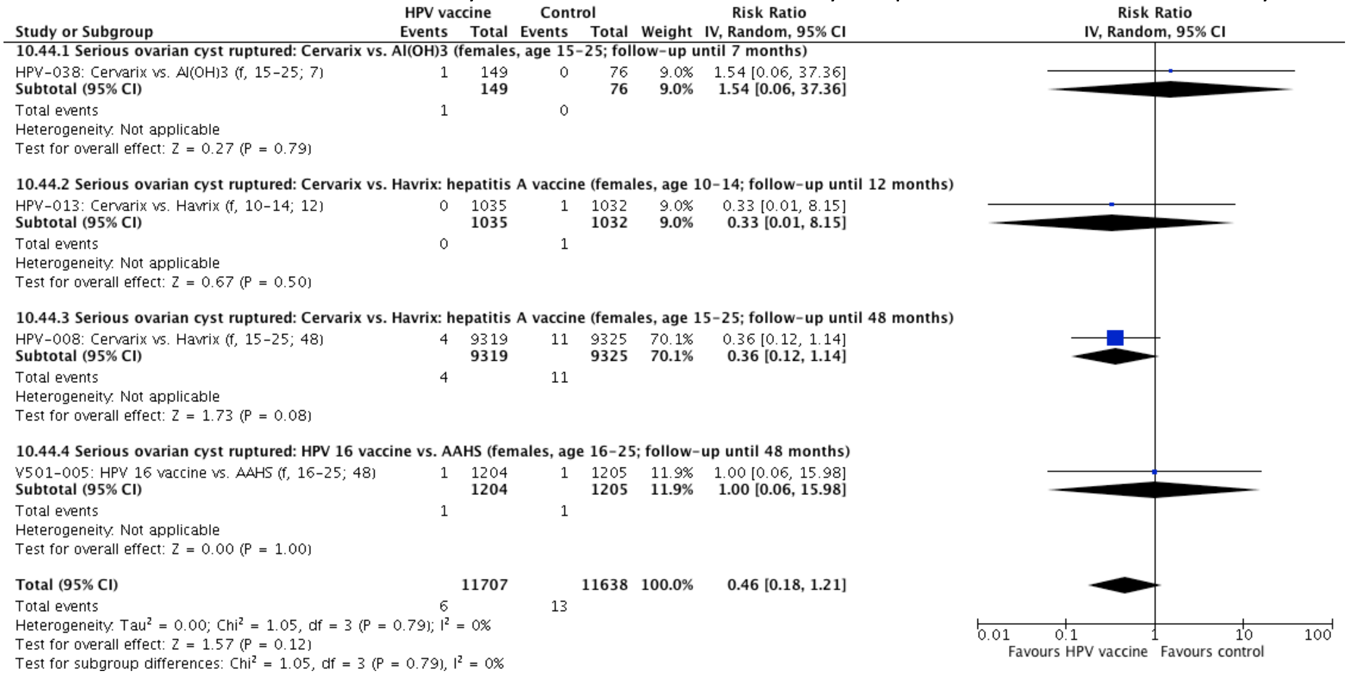

*10.44. Risk ratio for GlaxoSmithKline studies (i.e., HPV-0xx): 0.42 [0.15, 1.16]; risk ratio for Merck Sharp & Dohme studies (i.e., V50x-xxx): 1.00 [0.06, 15.98].

11. <u>New onset diseases</u>

11.1. New onset diseases ('medically significant conditions' and 'new medical history'*): intention to treat analysis

Study or Subgroup	HPV vaccine Events	HPV vaccine Total	Control Events	Control Total	Weight	Risk Ratio IV, Random, 95% CI
**11.1.1 New onset diseases ('medically significant conditions'): Cervarix vs. Al(OH)3 (females, age 9-17; follow-up until 12 months)**						
HPV-058: Cervarix vs. Al(OH)3 (f, 9-17; 12)	10	374	10	376	0.1%	1.01 [0.42, 2.39]
**Subtotal (95% CI)**		**374**		**376**	**0.1%**	**1.01 [0.42, 2.39]**
Total events	10		10			
Heterogeneity: Not applicable						
Test for overall effect: Z = 0.01 (P = 0.99)						
**11.1.2 New onset diseases ('medically significant conditions'): Cervarix vs. Al(OH)3 (females, age 15-25; follow-up until 7 months)**						
HPV-038: Cervarix vs. Al(OH)3 (f, 15-25; 7)	34	149	10	76	0.2%	1.73 [0.91, 3.32]
**Subtotal (95% CI)**		**149**		**76**	**0.2%**	**1.73 [0.91, 3.32]**
Total events	34		10			
Heterogeneity: Not applicable						
Test for overall effect: Z = 1.66 (P = 0.10)						
**11.1.3 New onset diseases ('medically significant conditions'): Cervarix vs. Al(OH)3 (females, age 18-35; follow-up until 7 months)**						
HPV-031: Cervarix vs. Al(OH)3 (f, 18-35; 7)	13	176	24	178	0.2%	0.55 [0.29, 1.04]
HPV-035: Cervarix vs. Al(OH)3 (f, 18-35; 7)	42	150	24	150	0.4%	1.75 [1.12, 2.74]
**Subtotal (95% CI)**		**326**		**328**	**0.6%**	**1.00 [0.32, 3.13]**
Total events	55		48			
Heterogeneity: Tau^2 = 0.59; Chi^2 = 8.46, df = 1 (P = 0.004); I^2 = 88%						
Test for overall effect: Z = 0.00 (P = 1.00)						
**11.1.4 New onset diseases ('medically significant conditions'): Cervarix vs. Al(OH)3 (females, age 24-72; follow-up until 36 months)**						
HPV-015: Cervarix vs. Al(OH)3 (f, 24-72; 36)	1170	2882	1140	2871	10.4%	1.02 [0.96, 1.09]
**Subtotal (95% CI)**		**2882**		**2871**	**10.4%**	**1.02 [0.96, 1.09]**
Total events	1170		1140			
Heterogeneity: Not applicable						
Test for overall effect: Z = 0.69 (P = 0.49)						
**11.1.5 New onset diseases ('medically significant conditions'): Cervarix vs. Aimmugen: hepatitis A vaccine (females, age 22-29; follow-up until 12 months)**						
HPV-032: Cervarix vs. Aimmugen (f, 19-25; 7)	98	519	115	521	1.3%	0.86 [0.67, 1.09]
**Subtotal (95% CI)**		**519**		**521**	**1.3%**	**0.86 [0.67, 1.09]**
Total events	98		115			
Heterogeneity: Not applicable						
Test for overall effect: Z = 1.27 (P = 0.20)						
**11.1.6 New onset diseases ('medically significant conditions'): Cervarix vs. Engerix: hepatitis B vaccine (females and males, age 9-15; follow-up until 12 months)**						
HPV-030: Cervarix vs. Engerix (f, 9-15; 12)	28	247	22	246	0.3%	1.27 [0.75, 2.15]
HPV-040: Cervarix vs. Engerix (f & m, 12-16; 72)	47	643	76	1047	0.6%	1.01 [0.71, 1.43]
**Subtotal (95% CI)**		**890**		**1293**	**0.9%**	**1.08 [0.81, 1.45]**
Total events	75		98			
Heterogeneity: Tau^2 = 0.00; Chi^2 = 0.50, df = 1 (P = 0.48); I^2 = 0%						
Test for overall effect: Z = 0.52 (P = 0.61)						
**11.1.7 New onset diseases ('medically significant conditions'): Cervarix vs. Engerix: hepatitis B vaccine (females, age 26-46; follow-up until 12 months)**						
HPV-069: Cervarix vs. Engerix (f, 26-46; 12)	5	606	7	606	0.1%	0.71 [0.23, 2.24]
**Subtotal (95% CI)**		**606**		**606**	**0.1%**	**0.71 [0.23, 2.24]**
Total events	5		7			
Heterogeneity: Not applicable						
Test for overall effect: Z = 0.58 (P = 0.56)						
**11.1.8 New onset diseases ('medically significant conditions'): Cervarix vs. Havrix: hepatitis A vaccine (females, age 9-15; follow-up until 12 months)**						
HPV-013: Cervarix vs. Havrix (f, 10-14; 12)	130	1035	160	1032	1.6%	0.81 [0.65, 1.00]
HPV-033: Cervarix vs. Havrix (f, 9-15; 7)	11	160	10	161	0.1%	1.11 [0.48, 2.53]
**Subtotal (95% CI)**		**1195**		**1193**	**1.7%**	**0.83 [0.67, 1.02]**
Total events	141		170			
Heterogeneity: Tau^2 = 0.00; Chi^2 = 0.51, df = 1 (P = 0.47); I^2 = 0%						
Test for overall effect: Z = 1.80 (P = 0.07)						
**11.1.9 New onset diseases ('medically significant conditions'): Cervarix vs. Havrix: hepatitis A vaccine (females, age 15-25; follow-up until 48 months)**						
HPV-008: Cervarix vs. Havrix (f, 15-25; 48)	3298	9319	3378	9325	15.6%	0.98 [0.94, 1.02]
**Subtotal (95% CI)**		**9319**		**9325**	**15.6%**	**0.98 [0.94, 1.02]**
Total events	3298		3378			
Heterogeneity: Not applicable						
Test for overall effect: Z = 1.19 (P = 0.23)						
**11.1.10 New onset diseases ('medically significant conditions'): Cervarix vs. Twinrix: hepatitis A and B vaccine (females, age 8-15; follow-up until 12 months)**						
HPV-029: Cervarix vs. Twinrix (f, 8-15; 12)	23	270	31	271	0.3%	0.74 [0.45, 1.24]
**Subtotal (95% CI)**		**270**		**271**	**0.3%**	**0.74 [0.45, 1.24]**
Total events	23		31			
Heterogeneity: Not applicable						
Test for overall effect: Z = 1.13 (P = 0.26)						
**11.1.11 New onset diseases ('new medical history'): Gardasil vs. carrier solution (females and males, age 9-16; follow-up until 18 months)**						
V501-018: Gardasil vs. carrier solu. (f&m,9-16;18)	520	1184	280	597	5.3%	0.94 [0.84, 1.04]
**Subtotal (95% CI)**		**1184**		**597**	**5.3%**	**0.94 [0.84, 1.04]**
Total events	520		280			
Heterogeneity: Not applicable						
Test for overall effect: Z = 1.20 (P = 0.23)						
**11.1.12 New onset diseases ('new medical history'): Gardasil vs. AAHS (females, age 15-26; follow-up until 45 months)**						
V501-013: Gardasil vs. AAHS (f, 16-24; 45)	2328	2723	2311	2732	19.4%	1.01 [0.99, 1.03]
V501-015: Gardasil vs. AAHS (f, 15-26; 36)	4357	6087	4399	6080	19.4%	0.99 [0.97, 1.01]
**Subtotal (95% CI)**		**8810**		**8812**	**38.8%**	**1.00 [0.98, 1.02]**
Total events	6685		6710			
Heterogeneity: Tau^2 = 0.00; Chi^2 = 1.78, df = 1 (P = 0.18); I^2 = 44%						
Test for overall effect: Z = 0.01 (P = 0.99)						
**11.1.13 New onset diseases ('new medical history'): Gardasil vs. AAHS (females, age 21-46; from 0-7 months)**						
V501-019: Gardasil vs. AAHS (f, 21-46; 48)	756	1911	702	1908	7.8%	1.08 [0.99, 1.17]
**Subtotal (95% CI)**		**1911**		**1908**	**7.8%**	**1.08 [0.99, 1.17]**
Total events	756		702			
Heterogeneity: Not applicable						
Test for overall effect: Z = 1.76 (P = 0.08)						
**11.1.14 New onset diseases ('new medical history'): Gardasil vs. AAHS (females, age 21-46; from 7-48 months)**						
V501-019: Gardasil vs. AAHS (f, 21-46; 48)	958	1911	985	1908		Not estimable
**Subtotal (95% CI)**		**0**		**0**		**Not estimable**
Total events	0		0			
Heterogeneity: Not applicable						
Test for overall effect: Not applicable						
**11.1.15 New onset diseases ('new medical history'): Gardasil vs. AAHS (males, age 15-27; from 0-7 months)**						
V501-020: Gardasil vs. AAHS (m, 15-27; 36)	498	2032	463	2032	5.0%	1.08 [0.96, 1.20]
**Subtotal (95% CI)**		**2032**		**2032**	**5.0%**	**1.08 [0.96, 1.20]**
Total events	498		463			
Heterogeneity: Not applicable						
Test for overall effect: Z = 1.29 (P = 0.20)						
**11.1.16 New onset diseases ('new medical history'): Gardasil vs. AAHS (males, age 15-27; from 7-36 months)**						
V501-020: Gardasil vs. AAHS (m, 15-27; 36)	512	2032	546	2033		Not estimable
**Subtotal (95% CI)**		**0**		**0**		**Not estimable**
Total events	0		0			
Heterogeneity: Not applicable						
Test for overall effect: Not applicable						
**11.1.17 New onset diseases ('new medical history'): Gardasil 9 vs. saline placebo (females, age 12-26; follow-up until 7 months)**						
V503-006: Gardasil 9 vs. saline (f, 12-26;7)	175	618	99	306	1.7%	0.88 [0.71, 1.07]
**Subtotal (95% CI)**		**618**		**306**	**1.7%**	**0.88 [0.71, 1.07]**
Total events	175		99			
Heterogeneity: Not applicable						
Test for overall effect: Z = 1.27 (P = 0.20)						
**11.1.18 New onset diseases ('new medical history'): HPV 16 vaccine vs. AAHS (females, age 16-25; from 0-7 months)**						
V501-005: HPV 16 vaccine vs. AAHS (f, 16-25; 48)	715	1204	753	1205	10.3%	0.95 [0.89, 1.01]
**Subtotal (95% CI)**		**1204**		**1205**	**10.3%**	**0.95 [0.89, 1.01]**
Total events	715		753			
Heterogeneity: Not applicable						
Test for overall effect: Z = 1.56 (P = 0.12)						
**11.1.19 New onset diseases ('new medical history'): HPV 16 vaccine vs. AAHS (females, age 16-25; from 7-48 months)**						
V501-005: HPV 16 vaccine vs. AAHS (f, 16-25; 48)	826	1204	834	1205		Not estimable
**Subtotal (95% CI)**		**0**		**0**		**Not estimable**
Total events	0		0			
Heterogeneity: Not applicable						
Test for overall effect: Not applicable						
**Total (95% CI)**		**32289**		**31720**	**100.0%**	**0.99 [0.97, 1.02]**
Total events	14258		14014			
Heterogeneity: Tau^2 = 0.00; Chi^2 = 33.56, df = 19 (P = 0.02); I^2 = 43%						
Test for overall effect: Z = 0.46 (P = 0.64)						
Test for subgroup differences: Chi^2 = 21.22, df = 15 (P = 0.13), I^2 = 29.3%						

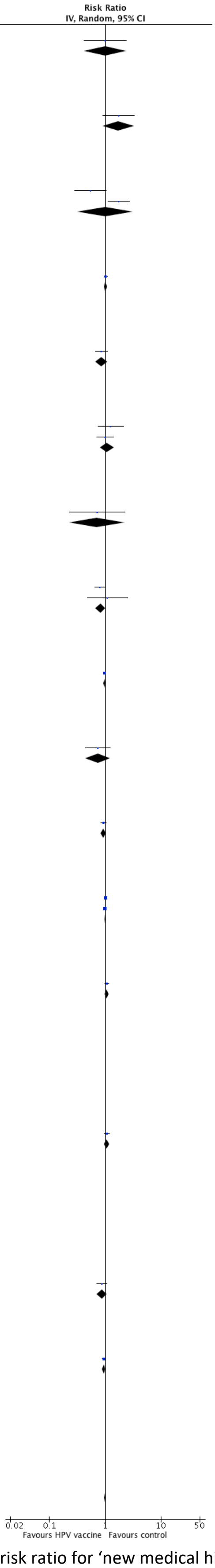

*11.1. Risk ratio for 'medically significant conditions’ (GlaxoSmithKline): 0.98 [0.90, 1.06]; risk ratio for ‘new medical history’ (Merck Sharp & Dohme): 1.00 [0.97, 1.03]; risk ratio for the follow-up periods for the trials V501-005, V501-019 and V501-020 (Merck Sharp & Dohme): 0.98 [0.94, 1.01] (2,296 participants with new medical history in the HPV vaccine group vs. 2,365 participants with new medical history in the control group. The trials V501-005, V501-019 and V501-020 split the reporting of new onset diseases into the vaccination period and the follow-up period. To avoid double counting of participants in the total risk ratio estimate, we only included the new onset diseases reported in the vaccination period for the trials V501-005, V501-019 and V501-020).

11.2. New onset diseases ('medically significant conditions' and 'new medical history'*) reported within the MedDRA system organ class ‘blood and lymphatic system disorders (10005329)': intention to treat analysis

Study or Subgroup	HPV vaccine Events	HPV vaccine Total	Control Events	Control Total	Weight	Risk Ratio IV, Random, 95% CI
**11.2.1 New onset blood and lymphatic system disorders ('medically significant conditions'): Cervarix vs. Al(OH)3 (females, age 24-72; follow-up until 36 months)**						
HPV-015: Cervarix vs. Al(OH)3 (f, 24-72; 36)	37	2882	31	2871	7.9%	1.19 [0.74, 1.91]
**Subtotal (95% CI)**		**2882**		**2871**	**7.9%**	**1.19 [0.74, 1.91]**
Total events	37		31			
Heterogeneity: Not applicable						
Test for overall effect: Z = 0.72 (P = 0.47)						
**11.2.2 New onset blood and lymphatic system disorders ('medically significant conditions'): Cervarix vs. Aimmugen: hepatitis A vaccine (females, age 22-29; follow-up until 12 months)**						
HPV-063: Cervarix vs. Aimmugen (f, 22-29; 12)	1	375	1	377	0.2%	1.01 [0.06, 16.01]
**Subtotal (95% CI)**		**375**		**377**	**0.2%**	**1.01 [0.06, 16.01]**
Total events	1		1			
Heterogeneity: Not applicable						
Test for overall effect: Z = 0.00 (P = 1.00)						
**11.2.3 New onset blood and lymphatic system disorders ('medically significant conditions'): Cervarix vs. Engerix: hepatitis B vaccine (females and males, age 9-15; follow-up until 12 months)**						
HPV-030: Cervarix vs. Engerix (f, 9-15; 12)	1	247	0	246	0.2%	2.99 [0.12, 72.99]
**Subtotal (95% CI)**		**247**		**246**	**0.2%**	**2.99 [0.12, 72.99]**
Total events	1		0			
Heterogeneity: Not applicable						
Test for overall effect: Z = 0.67 (P = 0.50)						
**11.2.4 New onset blood and lymphatic system disorders ('medically significant conditions'): Cervarix vs. Havrix: hepatitis A vaccine (females, age 9-15; follow-up until 12 months)**						
HPV-033: Cervarix vs. Havrix (f, 9-15; 7)	1	160	0	161	0.2%	3.02 [0.12, 73.55]
HPV-013: Cervarix vs. Havrix (f, 10-14; 12)	3	1035	6	1032	0.9%	0.50 [0.13, 1.99]
**Subtotal (95% CI)**		**1195**		**1193**	**1.1%**	**0.67 [0.18, 2.52]**
Total events	4		6			
Heterogeneity: Tau² = 0.05; Chi² = 1.03, df = 1 (P = 0.31); I² = 3%						
Test for overall effect: Z = 0.59 (P = 0.56)						
**11.2.5 New onset blood and lymphatic system disorders ('medically significant conditions'): Cervarix vs. Havrix: hepatitis A vaccine (females, age 15-25; follow-up until 48 months)**						
HPV-008: Cervarix vs. Havrix (f, 15-25; 48)	53	9319	46	9325	11.4%	1.15 [0.78, 1.71]
**Subtotal (95% CI)**		**9319**		**9325**	**11.4%**	**1.15 [0.78, 1.71]**
Total events	53		46			
Heterogeneity: Not applicable						
Test for overall effect: Z = 0.71 (P = 0.48)						
**11.2.6 New onset blood and lymphatic system disorders ('medically significant conditions'): Cervarix vs. Twinrix: hepatitis A and B vaccine (females, age 8-15; follow-up until 12 months)**						
HPV-029: Cervarix vs. Twinrix (f, 8-15; 12)	0	270	1	271	0.2%	0.33 [0.01, 8.18]
**Subtotal (95% CI)**		**270**		**271**	**0.2%**	**0.33 [0.01, 8.18]**
Total events	0		1			
Heterogeneity: Not applicable						
Test for overall effect: Z = 0.67 (P = 0.50)						
**11.2.7 New onset blood and lymphatic system disorders ('new medical history'): Gardasil vs. carrier solution (females and males, age 9-16; follow-up until 18 months)**						
V501-018: Gardasil vs. carrier solu. (f&m,9-16;18)	2	1184	2	597	0.5%	0.50 [0.07, 3.57]
**Subtotal (95% CI)**		**1184**		**597**	**0.5%**	**0.50 [0.07, 3.57]**
Total events	2		2			
Heterogeneity: Not applicable						
Test for overall effect: Z = 0.69 (P = 0.49)						
**11.2.8 New onset blood and lymphatic system disorders ('new medical history'): Gardasil vs. AAHS (females, age 15-26; follow-up until 45 months)**						
V501-015: Gardasil vs. AAHS (f, 15-26; 36)	239	6087	246	6080	58.3%	0.97 [0.82, 1.16]
V501-013: Gardasil vs. AAHS (f, 16-24; 45)	67	2723	61	2732	15.1%	1.10 [0.78, 1.55]
**Subtotal (95% CI)**		**8810**		**8812**	**73.4%**	**1.00 [0.85, 1.16]**
Total events	306		307			
Heterogeneity: Tau² = 0.00; Chi² = 0.42, df = 1 (P = 0.52); I² = 0%						
Test for overall effect: Z = 0.05 (P = 0.96)						
**11.2.9 New onset blood and lymphatic system disorders ('new medical history'): Gardasil vs. AAHS (females, age 21-46; from 0-7 months)**						
V501-019: Gardasil vs. AAHS (f, 21-46; 48)	7	1911	12	1908	2.0%	0.58 [0.23, 1.48]
**Subtotal (95% CI)**		**1911**		**1908**	**2.0%**	**0.58 [0.23, 1.48]**
Total events	7		12			
Heterogeneity: Not applicable						
Test for overall effect: Z = 1.14 (P = 0.25)						
**11.2.10 New onset blood and lymphatic system disorders ('new medical history'): Gardasil vs. AAHS (females, age 21-46; from 7-48 months)**						
V501-019: Gardasil vs. AAHS (f, 21-46; 48)	37	1911	33	1908	0.0%	1.12 [0.70, 1.78]
**Subtotal (95% CI)**		**0**		**0**		**Not estimable**
Total events	0		0			
Heterogeneity: Not applicable						
Test for overall effect: Not applicable						
**11.2.11 New onset blood and lymphatic system disorders ('new medical history'): Gardasil vs. AAHS (males, age 15-27; from 0-7 months)**						
V501-020: Gardasil vs. AAHS (m, 15-27; 36)	1	2032	3	2032	0.3%	0.33 [0.03, 3.20]
**Subtotal (95% CI)**		**2032**		**2032**	**0.3%**	**0.33 [0.03, 3.20]**
Total events	1		3			
Heterogeneity: Not applicable						
Test for overall effect: Z = 0.95 (P = 0.34)						
**11.2.12 New onset blood and lymphatic system disorders ('new medical history'): Gardasil vs. AAHS (males, age 15-27; from 7-36 months)**						
V501-020: Gardasil vs. AAHS (m, 15-27; 36)	4	2032	3	2033	0.0%	1.33 [0.30, 5.95]
**Subtotal (95% CI)**		**0**		**0**		**Not estimable**
Total events	0		0			
Heterogeneity: Not applicable						
Test for overall effect: Not applicable						
**11.2.13 New onset blood and lymphatic system disorders ('new medical history'): HPV 16 vaccine vs. AAHS (females, age 16-25; from 7-48 months)**						
V501-005: HPV 16 vaccine vs. AAHS (f, 16-25; 48)	12	1204	12	1205	2.8%	1.00 [0.45, 2.22]
**Subtotal (95% CI)**		**1204**		**1205**	**2.8%**	**1.00 [0.45, 2.22]**
Total events	12		12			
Heterogeneity: Not applicable						
Test for overall effect: Z = 0.00 (P = 1.00)						
**Total (95% CI)**		**29429**		**28837**	**100.0%**	**1.00 [0.88, 1.15]**
Total events	424		421			
Heterogeneity: Tau² = 0.00; Chi² = 6.44, df = 12 (P = 0.89); I² = 0%						
Test for overall effect: Z = 0.07 (P = 0.95)						
Test for subgroup differences: Chi² = 4.93, df = 10 (P = 0.90), I² = 0%						

Risk Ratio
IV, Random, 95% CI

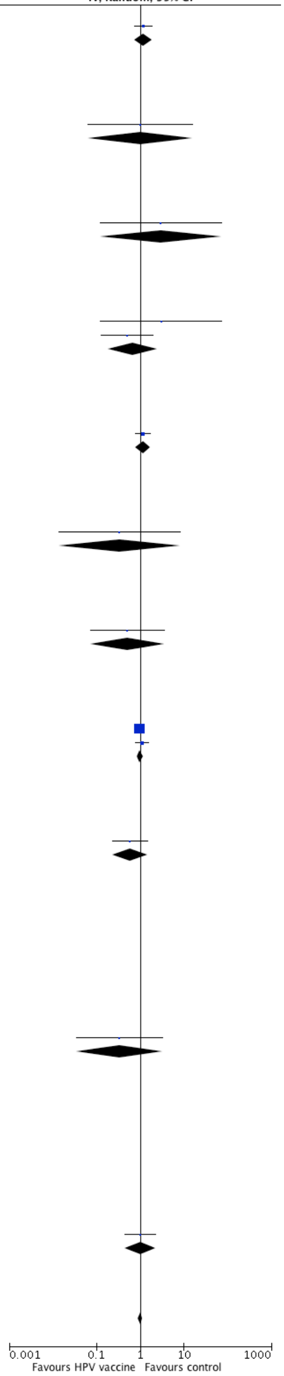

*11.2. Risk ratio for 'medically significant conditions’ (GlaxoSmithKline): 1.13 [0.84, 1.51]; risk ratio for ‘new medical history’ (Merck Sharp & Dohme): 0.98 [0.86, 1.13]; risk ratio for the follow-up periods for the trials V501-019 and V501-020: 1.14 [0.73, 1.77]. The trials V501-005, V501-019 and V501-020 split the reporting of new onset diseases into the vaccination period and the follow-up period. To avoid double counting of participants in the total risk ratio estimate, we only included the new onset diseases reported in the vaccination period for the trials V501-019 and V501-020.

11.3. New onset diseases ('medically significant conditions' and 'new medical history'*) reported within the MedDRA system organ class 'cardiac disorders (10007541)': intention to treat analysis

Study or Subgroup	HPV vaccine Events	HPV vaccine Total	Control Events	Control Total	Weight	Risk Ratio IV, Random, 95% CI
**11.3.1 New onset cardiac disorders ('medically significant conditions'): Cervarix vs. Al(OH)3 (females, age 24-72; follow-up until 36 months)**						
HPV-015: Cervarix vs. Al(OH)3 (f, 24-72; 36)	18	2882	11	2871	17.8%	1.63 [0.77, 3.45]
**Subtotal (95% CI)**		**2882**		**2871**	**17.8%**	**1.63 [0.77, 3.45]**
Total events	18		11			
Heterogeneity: Not applicable						
Test for overall effect: Z = 1.28 (P = 0.20)						
**11.3.2 New onset cardiac disorders ('medically significant conditions'): Cervarix vs. Engerix: hepatitis B vaccine (females, age 26-46; follow-up until 12 months)**						
HPV-069: Cervarix vs. Engerix (f, 26-46; 12)	0	606	3	606	2.5%	0.14 [0.01, 2.76]
**Subtotal (95% CI)**		**606**		**606**	**2.5%**	**0.14 [0.01, 2.76]**
Total events	0		3			
Heterogeneity: Not applicable						
Test for overall effect: Z = 1.29 (P = 0.20)						
**11.3.3 New onset cardiac disorders ('medically significant conditions'): Cervarix vs. Havrix: hepatitis A vaccine (females, age 10-14; follow-up until 12 months)**						
HPV-013: Cervarix vs. Havrix (f, 10-14; 12)	1	1035	2	1032	3.7%	0.50 [0.05, 5.49]
**Subtotal (95% CI)**		**1035**		**1032**	**3.7%**	**0.50 [0.05, 5.49]**
Total events	1		2			
Heterogeneity: Not applicable						
Test for overall effect: Z = 0.57 (P = 0.57)						
**11.3.4 New onset cardiac disorders ('medically significant conditions'): Cervarix vs. Havrix: hepatitis A vaccine (females, age 15-25; follow-up until 48 months)**						
HPV-008: Cervarix vs. Havrix (f, 15-25; 48)	43	9319	26	9325	23.5%	1.65 [1.02, 2.69]
**Subtotal (95% CI)**		**9319**		**9325**	**23.5%**	**1.65 [1.02, 2.69]**
Total events	43		26			
Heterogeneity: Not applicable						
Test for overall effect: Z = 2.03 (P = 0.04)						
**11.3.5 New onset cardiac disorders ('medically significant conditions'): Cervarix vs. Twinrix: hepatitis A and B vaccine (females, age 8-15; follow-up until 12 months)**						
HPV-029: Cervarix vs. Twinrix (f, 8-15; 12)	1	270	0	271	2.2%	3.01 [0.12, 73.59]
**Subtotal (95% CI)**		**270**		**271**	**2.2%**	**3.01 [0.12, 73.59]**
Total events	1		0			
Heterogeneity: Not applicable						
Test for overall effect: Z = 0.68 (P = 0.50)						
**11.3.6 New onset cardiac disorders ('new medical history'): Gardasil vs. carrier solution (females and males, age 9-16; follow-up until 18 months)**						
V501-018: Gardasil vs. carrier solu. (f&m,9-16;18)	1	1184	3	597	4.1%	0.17 [0.02, 1.61]
**Subtotal (95% CI)**		**1184**		**597**	**4.1%**	**0.17 [0.02, 1.61]**
Total events	1		3			
Heterogeneity: Not applicable						
Test for overall effect: Z = 1.55 (P = 0.12)						
**11.3.7 New onset cardiac disorders ('new medical history'): Gardasil vs. AAHS (females, age 15-26; follow-up until 45 months)**						
V501-013: Gardasil vs. AAHS (f, 16-24; 45)	13	2723	21	2732	19.0%	0.62 [0.31, 1.24]
V501-015: Gardasil vs. AAHS (f, 15-26; 36)	27	6087	16	6080	20.5%	1.69 [0.91, 3.12]
**Subtotal (95% CI)**		**8810**		**8812**	**39.5%**	**1.04 [0.39, 2.75]**
Total events	40		37			
Heterogeneity: $Tau^2 = 0.39$; $Chi^2 = 4.47$, df = 1 (P = 0.03); $I^2 = 78\%$						
Test for overall effect: Z = 0.07 (P = 0.94)						
**11.3.8 New onset ('new medical history'): Gardasil vs. AAHS (females, age 21-46; from 0-7 months)**						
V501-019: Gardasil vs. AAHS (f, 21-46; 48)	1	1911	4	1908	4.3%	0.25 [0.03, 2.23]
**Subtotal (95% CI)**		**1911**		**1908**	**4.3%**	**0.25 [0.03, 2.23]**
Total events	1		4			
Heterogeneity: Not applicable						
Test for overall effect: Z = 1.24 (P = 0.21)						
**11.3.9 New onset ('new medical history'): Gardasil vs. AAHS (females, age 21-46; from 7-48 months)**						
V501-019: Gardasil vs. AAHS (f, 21-46; 48)	7	1911	8	1908	0.0%	0.87 [0.32, 2.40]
**Subtotal (95% CI)**		**0**		**0**		**Not estimable**
Total events	0		0			
Heterogeneity: Not applicable						
Test for overall effect: Not applicable						
**11.3.10 New onset ('new medical history'): Gardasil vs. AAHS (males, age 15-27; from 0-7 months)**						
V501-020: Gardasil vs. AAHS (m, 15-27; 36)	3	2032	0	2032	2.5%	7.00 [0.36, 135.43]
**Subtotal (95% CI)**		**2032**		**2032**	**2.5%**	**7.00 [0.36, 135.43]**
Total events	3		0			
Heterogeneity: Not applicable						
Test for overall effect: Z = 1.29 (P = 0.20)						
**11.3.11 New onset ('new medical history'): Gardasil vs. AAHS (males, age 15-27; from 7-36 months)**						
V501-020: Gardasil vs. AAHS (m, 15-27; 36)	2	2032	5	2033	0.0%	0.40 [0.08, 2.06]
**Subtotal (95% CI)**		**0**		**0**		**Not estimable**
Total events	0		0			
Heterogeneity: Not applicable						
Test for overall effect: Not applicable						
**Total (95% CI)**		**28049**		**27454**	**100.0%**	**1.09 [0.67, 1.78]**
Total events	108		86			
Heterogeneity: $Tau^2 = 0.20$; $Chi^2 = 15.93$, df = 9 (P = 0.07); $I^2 = 43\%$						
Test for overall effect: Z = 0.36 (P = 0.72)						
Test for subgroup differences: $Chi^2 = 11.04$, df = 8 (P = 0.20), $I^2 = 27.6\%$						

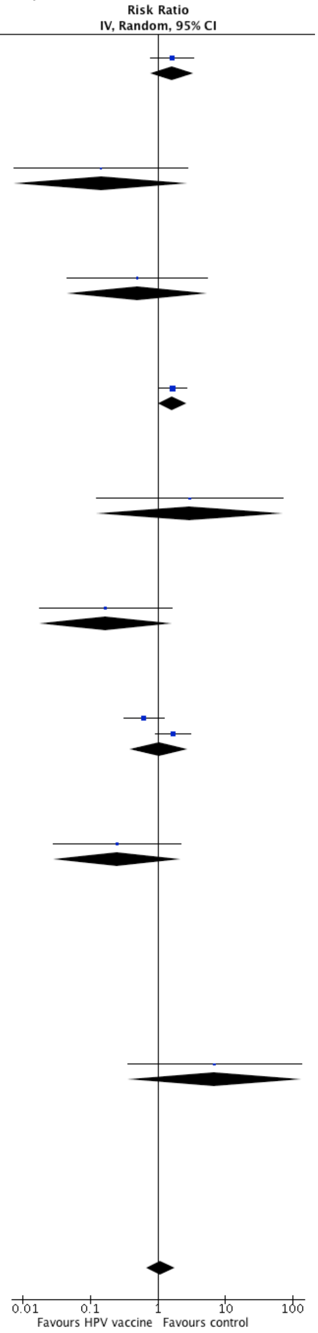

*11.3. Risk ratio for 'medically significant conditions' (GlaxoSmithKline): **1.54 [1.04, 2.29]**; risk ratio for 'new medical history' (Merck Sharp & Dohme): 0.81 [0.33, 2.03]; risk ratio for the follow-up periods for the trials V501-019 and V501-020: 0.70 [0.30, 1.67]. The trials V501-019 and V501-020 split the reporting of new onset diseases into the vaccination period and the follow-up period. To avoid double counting of participants in the total risk ratio estimate, we only included the new onset diseases reported in the vaccination period for the trials V501-019 and V501-020.

11.4. New onset diseases ('medically significant conditions' and 'new medical history'*) reported within the MedDRA system organ class ‘congenital familial and genetic disorders (10010331)': intention to treat analysis

Study or Subgroup	HPV vaccine Events	HPV vaccine Total	Control Events	Control Total	Weight	Risk Ratio IV, Random, 95% CI
**11.4.1 New onset congenital familial and genetic disorders ('medically significant conditions'): Cervarix vs. Al(OH)3 (females, age 18-35; follow-up until 7 months)**						
HPV-031: Cervarix vs. Al(OH)3 (f, 18-35; 7)	0	176	1	178	1.3%	0.34 [0.01, 8.22]
**Subtotal (95% CI)**		**176**		**178**	**1.3%**	**0.34 [0.01, 8.22]**
Total events	0		1			
Heterogeneity: Not applicable						
Test for overall effect: Z = 0.67 (P = 0.50)						
**11.4.2 New onset congenital familial and genetic disorders ('medically significant conditions'): Cervarix vs. Al(OH)3 (females, age 24-72; follow-up until 36 months)**						
HPV-015: Cervarix vs. Al(OH)3 (f, 24-72; 36)	4	2882	3	2871	5.9%	1.33 [0.30, 5.93]
**Subtotal (95% CI)**		**2882**		**2871**	**5.9%**	**1.33 [0.30, 5.93]**
Total events	4		3			
Heterogeneity: Not applicable						
Test for overall effect: Z = 0.37 (P = 0.71)						
**11.4.3 New onset congenital familial and genetic disorders ('medically significant conditions'): Cervarix vs. Aimmugen: hepatitis A vaccine (females, age 22-29; follow-up until 12 months)**						
HPV-063: Cervarix vs. Aimmugen (f, 22-29; 12)	0	375	1	377	1.3%	0.34 [0.01, 8.20]
**Subtotal (95% CI)**		**375**		**377**	**1.3%**	**0.34 [0.01, 8.20]**
Total events	0		1			
Heterogeneity: Not applicable						
Test for overall effect: Z = 0.67 (P = 0.50)						
**11.4.4 New onset congenital familial and genetic disorders ('medically significant conditions'): Cervarix vs. Engerix: hepatitis B vaccine (females, age 26-46; follow-up until 12 months)**						
HPV-069: Cervarix vs. Engerix (f, 26-46; 12)	0	606	1	606	1.3%	0.33 [0.01, 8.17]
**Subtotal (95% CI)**		**606**		**606**	**1.3%**	**0.33 [0.01, 8.17]**
Total events	0		1			
Heterogeneity: Not applicable						
Test for overall effect: Z = 0.67 (P = 0.50)						
**11.4.5 New onset congenital familial and genetic disorders ('medically significant conditions'): Cervarix vs. Havrix: hepatitis A vaccine (females, age 10-14; follow-up until 12 months)**						
HPV-013: Cervarix vs. Havrix (f, 10-14; 12)	0	1035	1	1032	1.3%	0.33 [0.01, 8.15]
**Subtotal (95% CI)**		**1035**		**1032**	**1.3%**	**0.33 [0.01, 8.15]**
Total events	0		1			
Heterogeneity: Not applicable						
Test for overall effect: Z = 0.67 (P = 0.50)						
**11.4.6 New onset congenital familial and genetic disorders ('medically significant conditions'): Cervarix vs. Havrix: hepatitis A vaccine (females, age 15-25; follow-up until 48 months)**						
HPV-008: Cervarix vs. Havrix (f, 15-25; 48)	8	9319	13	9325	17.1%	0.62 [0.26, 1.48]
**Subtotal (95% CI)**		**9319**		**9325**	**17.1%**	**0.62 [0.26, 1.48]**
Total events	8		13			
Heterogeneity: Not applicable						
Test for overall effect: Z = 1.08 (P = 0.28)						
**11.4.7 New onset congenital familial and genetic disorders ('new medical history'): Gardasil vs. carrier solution (females and males, age 9-16; follow-up until 18 months)**						
V501-018: Gardasil vs. carrier solu. (f&m,9-16;18)	2	1184	3	597	4.2%	0.34 [0.06, 2.01]
**Subtotal (95% CI)**		**1184**		**597**	**4.2%**	**0.34 [0.06, 2.01]**
Total events	2		3			
Heterogeneity: Not applicable						
Test for overall effect: Z = 1.20 (P = 0.23)						
**11.4.8 New onset congenital familial and genetic disorders ('new medical history'): Gardasil vs. AAHS (females, age 15-26; follow-up until 45 months)**						
V501-013: Gardasil vs. AAHS (f, 16-24; 45)	5	2723	8	2732	10.6%	0.63 [0.21, 1.91]
V501-015: Gardasil vs. AAHS (f, 15-26; 36)	18	6087	19	6080	32.0%	0.95 [0.50, 1.80]
**Subtotal (95% CI)**		**8810**		**8812**	**42.7%**	**0.85 [0.49, 1.49]**
Total events	23		27			
Heterogeneity: $Tau^2 = 0.00$; $Chi^2 = 0.39$, df = 1 (P = 0.53); $I^2 = 0\%$						
Test for overall effect: Z = 0.56 (P = 0.58)						
**11.4.9 New onset congenital familial and genetic disorders ('new medical history'): Gardasil vs. AAHS (females, age 21-46; from 0-7 months)**						
V501-019: Gardasil vs. AAHS (f, 21-46; 48)	2	1911	1	1908	2.3%	2.00 [0.18, 22.00]
**Subtotal (95% CI)**		**1911**		**1908**	**2.3%**	**2.00 [0.18, 22.00]**
Total events	2		1			
Heterogeneity: Not applicable						
Test for overall effect: Z = 0.56 (P = 0.57)						
**11.4.10 New onset congenital familial and genetic disorders ('new medical history'): Gardasil vs. AAHS (females, age 21-46; from 7-48 months)**						
V501-019: Gardasil vs. AAHS (f, 21-46; 48)	8	1911	8	1908	0.0%	1.00 [0.38, 2.65]
**Subtotal (95% CI)**		**0**		**0**		**Not estimable**
Total events	0		0			
Heterogeneity: Not applicable						
Test for overall effect: Not applicable						
**11.4.11 New onset congenital familial and genetic disorders ('new medical history'): Gardasil vs. AAHS (males, age 15-27; from 0-7 months)**						
V501-020: Gardasil vs. AAHS (m, 15-27; 36)	1	2032	1	2032	1.7%	1.00 [0.06, 15.98]
**Subtotal (95% CI)**		**2032**		**2032**	**1.7%**	**1.00 [0.06, 15.98]**
Total events	1		1			
Heterogeneity: Not applicable						
Test for overall effect: Z = 0.00 (P = 1.00)						
**11.4.12 New onset congenital familial and genetic disorders ('new medical history'): Gardasil vs. AAHS (males, age 15-27; from 7-36 months)**						
V501-020: Gardasil vs. AAHS (m, 15-27; 36)	3	2032	5	2033	0.0%	0.60 [0.14, 2.51]
**Subtotal (95% CI)**		**0**		**0**		**Not estimable**
Total events	0		0			
Heterogeneity: Not applicable						
Test for overall effect: Not applicable						
**11.4.13 New onset congenital familial and genetic disorders ('new medical history'): HPV 16 vaccine vs. AAHS (females, age 16-25; from 7-48 months)**						
V501-005: HPV 16 vaccine vs. AAHS (f, 16-25; 48)	10	1204	15	1205	20.9%	0.67 [0.30, 1.48]
**Subtotal (95% CI)**		**1204**		**1205**	**20.9%**	**0.67 [0.30, 1.48]**
Total events	10		15			
Heterogeneity: Not applicable						
Test for overall effect: Z = 1.00 (P = 0.32)						
**Total (95% CI)**		**29534**		**28943**	**100.0%**	**0.74 [0.51, 1.06]**
Total events	50		67			
Heterogeneity: $Tau^2 = 0.00$; $Chi^2 = 3.86$, df = 11 (P = 0.97); $I^2 = 0\%$						
Test for overall effect: Z = 1.64 (P = 0.10)						
Test for subgroup differences: $Chi^2 = 3.47$, df = 10 (P = 0.97), $I^2 = 0\%$						

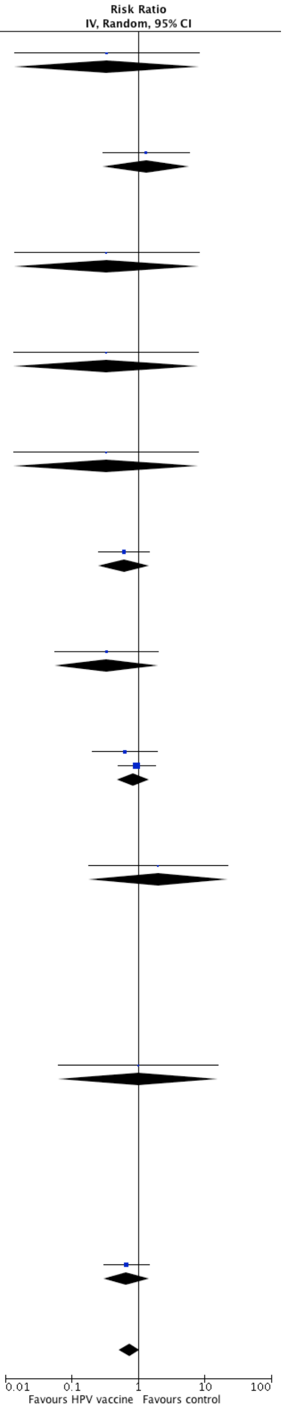

*11.4. Risk ratio for 'medically significant conditions’ (GlaxoSmithKline): 0.65 [0.33, 1.28]; risk ratio for ‘new medical history’ (Merck Sharp & Dohme): 0.78 [0.51, 1.19]; risk ratio for the follow-up periods for the trials V501-019 and V501-020: 0.85 [0.38, 1.90]. The trials V501-019 and V501-020 split the reporting of new onset diseases into the vaccination period and the follow-up period. To avoid double counting of participants in the total risk ratio estimate, we only included the new onset diseases reported in the vaccination period for the trials V501-019 and V501-020.

11.5. New onset diseases ('medically significant conditions' and 'new medical history'*) reported within the MedDRA system organ class ‘ear and labyrinth disorders (10013993)': intention to treat analysis

Study or Subgroup	HPV vaccine Events	HPV vaccine Total	Control Events	Control Total	Weight	Risk Ratio IV, Random, 95% CI
**11.5.1 New onset ear and labyrinth disorders ('medically significant conditions'): Cervarix vs. Al(OH)3 (females, age 15-25; follow-up until 7 months)**						
HPV-038: Cervarix vs. Al(OH)3 (f, 15-25; 7)	0	149	1	76	0.8%	0.17 [0.01, 4.15]
**Subtotal (95% CI)**		**149**		**76**	**0.8%**	**0.17 [0.01, 4.15]**
Total events	0		1			
Heterogeneity: Not applicable						
Test for overall effect: Z = 1.09 (P = 0.28)						
**11.5.2 New onset ear and labyrinth disorders ('medically significant conditions'): Cervarix vs. Al(OH)3 (females, age 24-72; follow-up until 36 months)**						
HPV-015: Cervarix vs. Al(OH)3 (f, 24-72; 36)	27	2882	23	2871	16.2%	1.17 [0.67, 2.03]
**Subtotal (95% CI)**		**2882**		**2871**	**16.2%**	**1.17 [0.67, 2.03]**
Total events	27		23			
Heterogeneity: Not applicable						
Test for overall effect: Z = 0.55 (P = 0.58)						
**11.5.3 New onset ear and labyrinth disorders ('medically significant conditions'): Cervarix vs. Aimmugen: hepatitis A vaccine (females, age 22-29; follow-up until 12 months)**						
HPV-063: Cervarix vs. Aimmugen (f, 22-29; 12)	1	375	1	377	1.1%	1.01 [0.06, 16.01]
**Subtotal (95% CI)**		**375**		**377**	**1.1%**	**1.01 [0.06, 16.01]**
Total events	1		1			
Heterogeneity: Not applicable						
Test for overall effect: Z = 0.00 (P = 1.00)						
**11.5.4 New onset ear and labyrinth disorders ('medically significant conditions'): Cervarix vs. Engerix: hepatitis B vaccine (females and males, age 9-15; follow-up until 12 months)**						
HPV-030: Cervarix vs. Engerix (f, 9-15; 12)	0	247	2	246	0.9%	0.20 [0.01, 4.13]
**Subtotal (95% CI)**		**247**		**246**	**0.9%**	**0.20 [0.01, 4.13]**
Total events	0		2			
Heterogeneity: Not applicable						
Test for overall effect: Z = 1.04 (P = 0.30)						
**11.5.5 New onset ear and labyrinth disorders ('medically significant conditions'): Cervarix vs. Havrix: hepatitis A vaccine (females, age 9-15; follow-up until 12 months)**						
HPV-013: Cervarix vs. Havrix (f, 10-14; 12)	5	1035	4	1032	4.4%	1.25 [0.34, 4.63]
HPV-033: Cervarix vs. Havrix (f, 9-15; 7)	0	160	1	161	0.8%	0.34 [0.01, 8.17]
**Subtotal (95% CI)**		**1195**		**1193**	**5.2%**	**1.03 [0.31, 3.47]**
Total events	5		5			
Heterogeneity: $Tau^2 = 0.00$; $Chi^2 = 0.56$, df = 1 (P = 0.46); $I^2 = 0\%$						
Test for overall effect: Z = 0.05 (P = 0.96)						
**11.5.6 New onset ear and labyrinth disorders ('medically significant conditions'): Cervarix vs. Havrix: hepatitis A vaccine (females, age 15-25; follow-up until 48 months)**						
HPV-008: Cervarix vs. Havrix (f, 15-25; 48)	14	9319	29	9325	13.6%	0.48 [0.26, 0.91]
**Subtotal (95% CI)**		**9319**		**9325**	**13.6%**	**0.48 [0.26, 0.91]**
Total events	14		29			
Heterogeneity: Not applicable						
Test for overall effect: Z = 2.24 (P = 0.03)						
**11.5.7 New onset ear and labyrinth disorders ('medically significant conditions'): Cervarix vs. Twinrix: hepatitis A and B vaccine (females, age 8-15; follow-up until 12 months)**						
HPV-029: Cervarix vs. Twinrix (f, 8-15; 12)	3	270	0	271	1.0%	7.03 [0.36, 135.37]
**Subtotal (95% CI)**		**270**		**271**	**1.0%**	**7.03 [0.36, 135.37]**
Total events	3		0			
Heterogeneity: Not applicable						
Test for overall effect: Z = 1.29 (P = 0.20)						
**11.5.8 New onset ear and labyrinth disorders ('new medical history'): Gardasil vs. carrier solution (females and males, age 9-16; follow-up until 18 months)**						
V501-018: Gardasil vs. carrier solu. (f&m,9-16;18)	7	1184	7	597	6.5%	0.50 [0.18, 1.43]
**Subtotal (95% CI)**		**1184**		**597**	**6.5%**	**0.50 [0.18, 1.43]**
Total events	7		7			
Heterogeneity: Not applicable						
Test for overall effect: Z = 1.29 (P = 0.20)						
**11.5.9 New onset ear and labyrinth disorders ('new medical history'): Gardasil vs. AAHS (females, age 15-26; follow-up until 45 months)**						
V501-013: Gardasil vs. AAHS (f, 16-24; 45)	32	2723	23	2732	16.9%	1.40 [0.82, 2.38]
V501-015: Gardasil vs. AAHS (f, 15-26; 36)	40	6087	31	6080	19.4%	1.29 [0.81, 2.06]
**Subtotal (95% CI)**		**8810**		**8812**	**36.3%**	**1.33 [0.94, 1.90]**
Total events	72		54			
Heterogeneity: $Tau^2 = 0.00$; $Chi^2 = 0.05$, df = 1 (P = 0.83); $I^2 = 0\%$						
Test for overall effect: Z = 1.61 (P = 0.11)						
**11.5.10 New onset ear and labyrinth disorders ('new medical history'): Gardasil vs. AAHS (females, age 21-46; from 0-7 months)**						
V501-019: Gardasil vs. AAHS (f, 21-46; 48)	9	1911	6	1908	6.6%	1.50 [0.53, 4.20]
**Subtotal (95% CI)**		**1911**		**1908**	**6.6%**	**1.50 [0.53, 4.20]**
Total events	9		6			
Heterogeneity: Not applicable						
Test for overall effect: Z = 0.77 (P = 0.44)						
**11.5.11 New onset ear and labyrinth disorders ('new medical history'): Gardasil vs. AAHS (females, age 21-46; from 7-48 months)**						
V501-019: Gardasil vs. AAHS (f, 21-46; 48)	32	1911	26	1908	0.0%	1.23 [0.74, 2.05]
**Subtotal (95% CI)**		**0**		**0**		**Not estimable**
Total events	0		0			
Heterogeneity: Not applicable						
Test for overall effect: Not applicable						
**11.5.12 New onset ear and labyrinth disorders ('new medical history'): Gardasil vs. AAHS (males, age 15-27; from 0-7 months)**						
V501-020: Gardasil vs. AAHS (m, 15-27; 36)	5	2032	2	2032	2.9%	2.50 [0.49, 12.87]
**Subtotal (95% CI)**		**2032**		**2032**	**2.9%**	**2.50 [0.49, 12.87]**
Total events	5		2			
Heterogeneity: Not applicable						
Test for overall effect: Z = 1.10 (P = 0.27)						
**11.5.13 New onset ear and labyrinth disorders ('new medical history'): Gardasil vs. AAHS (males, age 15-27; from 7-36 months)**						
V501-020: Gardasil vs. AAHS (m, 15-27; 36)	7	2032	5	2033	0.0%	1.40 [0.45, 4.41]
**Subtotal (95% CI)**		**0**		**0**		**Not estimable**
Total events	0		0			
Heterogeneity: Not applicable						
Test for overall effect: Not applicable						
**11.5.14 New onset ear and labyrinth disorders ('new medical history'): HPV 16 vaccine vs. AAHS (females, age 16-25; from 7-48 months)**						
V501-005: HPV 16 vaccine vs. AAHS (f, 16-25; 48)	10	1204	7	1205	7.4%	1.43 [0.55, 3.74]
**Subtotal (95% CI)**		**1204**		**1205**	**7.4%**	**1.43 [0.55, 3.74]**
Total events	10		7			
Heterogeneity: Not applicable						
Test for overall effect: Z = 0.73 (P = 0.47)						
**11.5.15 New onset ear and labyrinth disorders ('new medical history'): Gardasil 9 vs. saline placebo (females, age 12-26; follow-up until 7 months)**						
V503-006: Gardasil 9 vs. saline (f, 12-26;7)	1	618	2	306	1.4%	0.25 [0.02, 2.72]
**Subtotal (95% CI)**		**618**		**306**	**1.4%**	**0.25 [0.02, 2.72]**
Total events	1		2			
Heterogeneity: Not applicable						
Test for overall effect: Z = 1.14 (P = 0.25)						
**Total (95% CI)**		**30196**		**29219**	**100.0%**	**1.04 [0.78, 1.39]**
Total events	154		139			
Heterogeneity: $Tau^2 = 0.06$; $Chi^2 = 17.43$, df = 14 (P = 0.23); $I^2 = 20\%$						
Test for overall effect: Z = 0.25 (P = 0.80)						
Test for subgroup differences: $Chi^2 = 16.83$, df = 12 (P = 0.16), $I^2 = 28.7\%$						

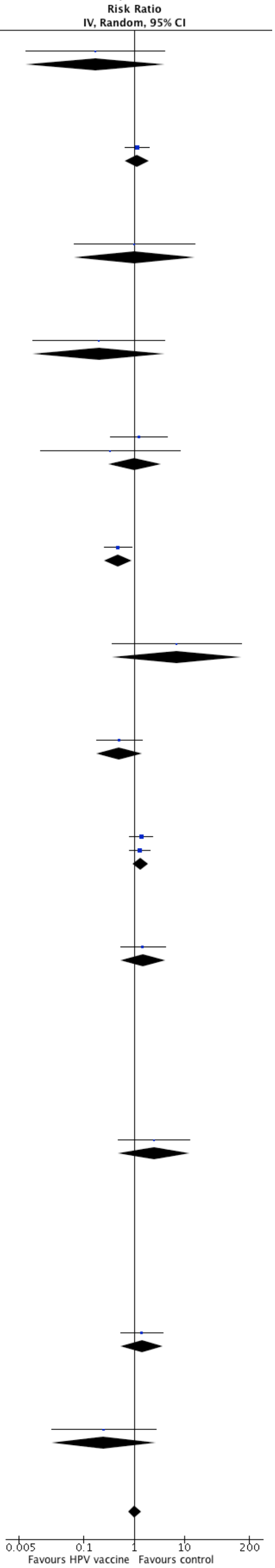

*11.5. Risk ratio for 'medically significant conditions’ (GlaxoSmithKline): 0.80 [0.48, 1.35]; risk ratio for ‘new medical history’ (Merck Sharp & Dohme): 1.25 [0.93, 1.67]; risk ratio for the follow-up periods for the trials V501-019 and V501-020: 1.26 [0.79, 2.01]. The trials V501-019 and V501-020 split the reporting of new onset diseases into the vaccination period and the follow-up period. To avoid double counting of participants in the total risk ratio estimate, we only included the new onset diseases reported in the vaccination period for the trials V501-019 and V501-020.

11.6. New onset diseases ('medically significant conditions' and 'new medical history'*) reported within the MedDRA system organ class 'endocrine disorders (10014698)': intention to treat analysis

Study or Subgroup	HPV vaccine Events	HPV vaccine Total	Control Events	Control Total	Weight	Risk Ratio IV, Random, 95% CI
**11.6.1 New onset endocrine disorders ('medically significant conditions'): Cervarix vs. Al(OH)3 (females, age 15-25; follow-up until 7 months)**						
HPV-038: Cervarix vs. Al(OH)3 (f, 15-25; 7)	0	149	1	76	0.4%	0.17 [0.01, 4.15]
**Subtotal (95% CI)**		**149**		**76**	**0.4%**	**0.17 [0.01, 4.15]**
Total events	0		1			
Heterogeneity: Not applicable						
Test for overall effect: Z = 1.09 (P = 0.28)						
**11.6.2 New onset endocrine disorders ('medically significant conditions'): Cervarix vs. Al(OH)3 (females, age 18-35; follow-up until 7 months)**						
HPV-035: Cervarix vs. Al(OH)3 (f, 18-35; 7)	1	150	0	150	0.4%	3.00 [0.12, 73.06]
**Subtotal (95% CI)**		**150**		**150**	**0.4%**	**3.00 [0.12, 73.06]**
Total events	1		0			
Heterogeneity: Not applicable						
Test for overall effect: Z = 0.67 (P = 0.50)						
**11.6.3 New onset endocrine disorders ('medically significant conditions'): Cervarix vs. Al(OH)3 (females, age 24-72; follow-up until 36 months)**						
HPV-015: Cervarix vs. Al(OH)3 (f, 24-72; 36)	37	2882	33	2871	17.9%	1.12 [0.70, 1.78]
**Subtotal (95% CI)**		**2882**		**2871**	**17.9%**	**1.12 [0.70, 1.78]**
Total events	37		33			
Heterogeneity: Not applicable						
Test for overall effect: Z = 0.46 (P = 0.64)						
**11.6.4 New onset endocrine disorders ('medically significant conditions'): Cervarix vs. Engerix: hepatitis B vaccine (females, age 26-46; follow-up until 12 months)**						
HPV-069: Cervarix vs. Engerix (f, 26-46; 12)	1	606	0	606	0.4%	3.00 [0.12, 73.50]
**Subtotal (95% CI)**		**606**		**606**	**0.4%**	**3.00 [0.12, 73.50]**
Total events	1		0			
Heterogeneity: Not applicable						
Test for overall effect: Z = 0.67 (P = 0.50)						
**11.6.5 New onset endocrine disorders ('medically significant conditions'): Cervarix vs. Havrix: hepatitis A vaccine (females, age 10-14; follow-up until 12 months)**						
HPV-013: Cervarix vs. Havrix (f, 10-14; 12)	1	1035	3	1032	0.8%	0.33 [0.03, 3.19]
**Subtotal (95% CI)**		**1035**		**1032**	**0.8%**	**0.33 [0.03, 3.19]**
Total events	1		3			
Heterogeneity: Not applicable						
Test for overall effect: Z = 0.95 (P = 0.34)						
**11.6.6 New onset endocrine disorders ('medically significant conditions'): Cervarix vs. Havrix: hepatitis A vaccine (females, age 15-25; follow-up until 48 months)**						
HPV-008: Cervarix vs. Havrix (f, 15-25; 48)	55	9319	53	9325	27.5%	1.04 [0.71, 1.51]
**Subtotal (95% CI)**		**9319**		**9325**	**27.5%**	**1.04 [0.71, 1.51]**
Total events	55		53			
Heterogeneity: Not applicable						
Test for overall effect: Z = 0.20 (P = 0.84)						
**11.6.7 New onset endocrine disorders ('new medical history'): Gardasil vs. carrier solution (females and males, age 9-16; follow-up until 18 months)**						
V501-018: Gardasil vs. carrier solu. (f&m,9-16;18)	1	1184	0	597	0.4%	1.51 [0.06, 37.11]
**Subtotal (95% CI)**		**1184**		**597**	**0.4%**	**1.51 [0.06, 37.11]**
Total events	1		0			
Heterogeneity: Not applicable						
Test for overall effect: Z = 0.25 (P = 0.80)						
**11.6.8 New onset endocrine disorders ('new medical history'): Gardasil vs. AAHS (females, age 15-26; follow-up until 45 months)**						
V501-013: Gardasil vs. AAHS (f, 16-24; 45)	23	2723	23	2732	11.8%	1.00 [0.56, 1.78]
V501-015: Gardasil vs. AAHS (f, 15-26; 36)	42	6087	50	6080	23.3%	0.84 [0.56, 1.26]
**Subtotal (95% CI)**		**8810**		**8812**	**35.1%**	**0.89 [0.64, 1.24]**
Total events	65		73			
Heterogeneity: $Tau^2 = 0.00$; $Chi^2 = 0.25$, df = 1 (P = 0.62); $I^2 = 0\%$						
Test for overall effect: Z = 0.68 (P = 0.50)						
**11.6.9 New onset endocrine disorders ('new medical history'): Gardasil vs. AAHS (females, age 21-46; from 0-7 months)**						
V501-019: Gardasil vs. AAHS (f, 21-46; 48)	9	1911	9	1908	4.6%	1.00 [0.40, 2.51]
**Subtotal (95% CI)**		**1911**		**1908**	**4.6%**	**1.00 [0.40, 2.51]**
Total events	9		9			
Heterogeneity: Not applicable						
Test for overall effect: Z = 0.00 (P = 1.00)						
**11.6.10 New onset endocrine disorders ('new medical history'): Gardasil vs. AAHS (females, age 21-46; from 7-48 months)**						
V501-019: Gardasil vs. AAHS (f, 21-46; 48)	18	1911	22	1908	0.0%	0.82 [0.44, 1.52]
**Subtotal (95% CI)**		**0**		**0**		**Not estimable**
Total events	0		0			
Heterogeneity: Not applicable						
Test for overall effect: Not applicable						
**11.6.11 New onset endocrine disorders ('new medical history'): Gardasil vs. AAHS (males, age 15-27; from 0-7 months)**						
V501-020: Gardasil vs. AAHS (m, 15-27; 36)	1	2032	0	2032	0.4%	3.00 [0.12, 73.60]
**Subtotal (95% CI)**		**2032**		**2032**	**0.4%**	**3.00 [0.12, 73.60]**
Total events	1		0			
Heterogeneity: Not applicable						
Test for overall effect: Z = 0.67 (P = 0.50)						
**11.6.12 New onset endocrine disorders ('new medical history'): Gardasil vs. AAHS (males, age 15-27; from 7-36 months)**						
V501-020: Gardasil vs. AAHS (m, 15-27; 36)	0	2032	1	2033	0.0%	0.33 [0.01, 8.18]
**Subtotal (95% CI)**		**0**		**0**		**Not estimable**
Total events	0		0			
Heterogeneity: Not applicable						
Test for overall effect: Not applicable						
**11.6.13 New onset endocrine disorders ('new medical history'): HPV 16 vaccine vs. AAHS (females, age 16-25; from 7-48 months)**						
V501-005: HPV 16 vaccine vs. AAHS (f, 16-25; 48)	17	1204	32	1205	11.5%	0.53 [0.30, 0.95]
**Subtotal (95% CI)**		**1204**		**1205**	**11.5%**	**0.53 [0.30, 0.95]**
Total events	17		32			
Heterogeneity: Not applicable						
Test for overall effect: Z = 2.12 (P = 0.03)						
**11.6.14 New onset endocrine disorders ('new medical history'): Gardasil 9 vs. saline placebo (females, age 12-26; follow-up until 7 months)**						
V503-006: Gardasil 9 vs. saline (f, 12-26;7)	1	618	3	306	0.8%	0.17 [0.02, 1.58]
**Subtotal (95% CI)**		**618**		**306**	**0.8%**	**0.17 [0.02, 1.58]**
Total events	1		3			
Heterogeneity: Not applicable						
Test for overall effect: Z = 1.56 (P = 0.12)						
**Total (95% CI)**		**29900**		**28920**	**100.0%**	**0.91 [0.74, 1.10]**
Total events	189		207			
Heterogeneity: $Tau^2 = 0.00$; $Chi^2 = 10.49$, df = 12 (P = 0.57); $I^2 = 0\%$						
Test for overall effect: Z = 0.97 (P = 0.33)						
Test for subgroup differences: $Chi^2 = 10.25$, df = 11 (P = 0.51), $I^2 = 0\%$						

Risk Ratio
IV, Random, 95% CI

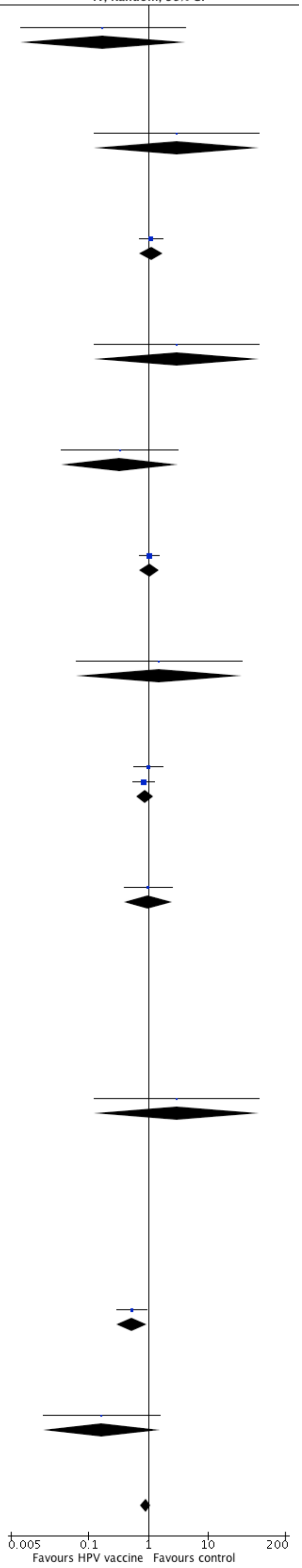

*11.6. Risk ratio for 'medically significant conditions' (GlaxoSmithKline): 1.05 [0.79, 1.40]; risk ratio for 'new medical history' (Merck Sharp & Dohme): 0.79 [0.61, 1.04]; risk ratio for the follow-up periods for the trials V501-019 and V501-020: 0.79 [0.43, 1.45]. The trials V501-019 and V501-020 split the reporting of new onset diseases into the vaccination period and the follow-up period. To avoid double counting of participants in the total risk ratio estimate, we only included the new onset diseases reported in the vaccination period for the trials V501-019 and V501-020.

11.7. New onset diseases ('medically significant conditions' and 'new medical history'*) reported within the MedDRA system organ class ‘eye disorders (10015919)': intention to treat analysis

Study or Subgroup	HPV vaccine Events	HPV vaccine Total	Control Events	Control Total	Weight	Risk Ratio IV, Random, 95% CI
**11.7.1 New onset eye disorders ('medically significant conditions'): Cervarix vs. Al(OH)3 (females, age 15-25; follow-up until 7 months)**						
HPV-038: Cervarix vs. Al(OH)3 (f, 15-25; 7)	1	149	1	76	0.3%	0.51 [0.03, 8.04]
**Subtotal (95% CI)**		**149**		**76**	**0.3%**	**0.51 [0.03, 8.04]**
Total events	1		1			
Heterogeneity: Not applicable						
Test for overall effect: Z = 0.48 (P = 0.63)						
**11.7.2 New onset eye disorders ('medically significant conditions'): Cervarix vs. Al(OH)3 (females, age 18-35; follow-up until 7 months)**						
HPV-031: Cervarix vs. Al(OH)3 (f, 18-35; 7)	0	176	2	178	0.3%	0.20 [0.01, 4.18]
HPV-035: Cervarix vs. Al(OH)3 (f, 18-35; 7)	1	150	0	150	0.2%	3.00 [0.12, 73.06]
**Subtotal (95% CI)**		**326**		**328**	**0.5%**	**0.74 [0.05, 10.40]**
Total events	1		2			
Heterogeneity: Tau^2 = 1.12; Chi^2 = 1.44, df = 1 (P = 0.23); I^2 = 31%						
Test for overall effect: Z = 0.22 (P = 0.82)						
**11.7.3 New onset eye disorders ('medically significant conditions'): Cervarix vs. Al(OH)3 (females, age 24-72; follow-up until 36 months)**						
HPV-015: Cervarix vs. Al(OH)3 (f, 24-72; 36)	17	2882	11	2871	4.3%	1.54 [0.72, 3.28]
**Subtotal (95% CI)**		**2882**		**2871**	**4.3%**	**1.54 [0.72, 3.28]**
Total events	17		11			
Heterogeneity: Not applicable						
Test for overall effect: Z = 1.12 (P = 0.26)						
**11.7.4 New onset eye disorders ('medically significant conditions'): Cervarix vs. Aimmugen: hepatitis A vaccine (females, age 22-29; follow-up until 12 months)**						
HPV-063: Cervarix vs. Aimmugen (f, 22-29; 12)	3	375	6	377	1.3%	0.50 [0.13, 2.00]
**Subtotal (95% CI)**		**375**		**377**	**1.3%**	**0.50 [0.13, 2.00]**
Total events	3		6			
Heterogeneity: Not applicable						
Test for overall effect: Z = 0.98 (P = 0.33)						
**11.7.5 New onset eye disorders ('medically significant conditions'): Cervarix vs. Engerix: hepatitis B vaccine (females and males, age 9-15; follow-up until 12 months)**						
HPV-030: Cervarix vs. Engerix (f, 9-15; 12)	0	247	1	246	0.2%	0.33 [0.01, 8.11]
**Subtotal (95% CI)**		**247**		**246**	**0.2%**	**0.33 [0.01, 8.11]**
Total events	0		1			
Heterogeneity: Not applicable						
Test for overall effect: Z = 0.68 (P = 0.50)						
**11.7.6 New onset eye disorders ('medically significant conditions'): Cervarix vs. Havrix: hepatitis A vaccine (females, age 9-15; follow-up until 12 months)**						
HPV-013: Cervarix vs. Havrix (f, 10-14; 12)	5	1035	9	1032	2.1%	0.55 [0.19, 1.65]
HPV-033: Cervarix vs. Havrix (f, 9-15; 7)	1	160	0	161	0.2%	3.02 [0.12, 73.55]
**Subtotal (95% CI)**		**1195**		**1193**	**2.3%**	**0.66 [0.24, 1.85]**
Total events	6		9			
Heterogeneity: Tau^2 = 0.00; Chi^2 = 0.97, df = 1 (P = 0.32); I^2 = 0%						
Test for overall effect: Z = 0.79 (P = 0.43)						
**11.7.7 New onset eye disorders ('medically significant conditions'): Cervarix vs. Havrix: hepatitis A vaccine (females, age 15-25; follow-up until 48 months)**						
HPV-008: Cervarix vs. Havrix (f, 15-25; 48)	63	9319	73	9325	21.9%	0.86 [0.62, 1.21]
**Subtotal (95% CI)**		**9319**		**9325**	**21.9%**	**0.86 [0.62, 1.21]**
Total events	63		73			
Heterogeneity: Not applicable						
Test for overall effect: Z = 0.86 (P = 0.39)						
**11.7.8 New onset eye disorders ('new medical history'): Gardasil vs. carrier solution (females and males, age 9-16; follow-up until 18 months)**						
V501-018: Gardasil vs. carrier solu. (f&m,9-16;18)	10	1184	5	597	2.2%	1.01 [0.35, 2.94]
**Subtotal (95% CI)**		**1184**		**597**	**2.2%**	**1.01 [0.35, 2.94]**
Total events	10		5			
Heterogeneity: Not applicable						
Test for overall effect: Z = 0.02 (P = 0.99)						
**11.7.9 New onset eye disorders ('new medical history'): Gardasil vs. AAHS (females, age 15-26; follow-up until 45 months)**						
V501-013: Gardasil vs. AAHS (f, 16-24; 45)	56	2723	56	2732	18.4%	1.00 [0.70, 1.45]
V501-015: Gardasil vs. AAHS (f, 15-26; 36)	101	6087	121	6080	36.0%	0.83 [0.64, 1.08]
**Subtotal (95% CI)**		**8810**		**8812**	**54.4%**	**0.89 [0.72, 1.10]**
Total events	157		177			
Heterogeneity: Tau^2 = 0.00; Chi^2 = 0.65, df = 1 (P = 0.42); I^2 = 0%						
Test for overall effect: Z = 1.10 (P = 0.27)						
**11.7.10 New onset eye disorders ('new medical history'): Gardasil vs. AAHS (females, age 21-46; from 0-7 months)**						
V501-019: Gardasil vs. AAHS (f, 21-46; 48)	16	1911	22	1908	6.0%	0.73 [0.38, 1.38]
**Subtotal (95% CI)**		**1911**		**1908**	**6.0%**	**0.73 [0.38, 1.38]**
Total events	16		22			
Heterogeneity: Not applicable						
Test for overall effect: Z = 0.98 (P = 0.33)						
**11.7.11 New onset eye disorders ('new medical history'): Gardasil vs. AAHS (females, age 21-46; from 7-48 months)**						
V501-019: Gardasil vs. AAHS (f, 21-46; 48)	50	1911	43	1908	0.0%	1.16 [0.78, 1.74]
**Subtotal (95% CI)**		**0**		**0**		**Not estimable**
Total events	0		0			
Heterogeneity: Not applicable						
Test for overall effect: Not applicable						
**11.7.12 New onset eye disorders ('new medical history'): Gardasil vs. AAHS (males, age 15-27; from 0-7 months)**						
V501-020: Gardasil vs. AAHS (m, 15-27; 36)	6	2032	5	2032	1.8%	1.20 [0.37, 3.93]
**Subtotal (95% CI)**		**2032**		**2032**	**1.8%**	**1.20 [0.37, 3.93]**
Total events	6		5			
Heterogeneity: Not applicable						
Test for overall effect: Z = 0.30 (P = 0.76)						
**11.7.13 New onset eye disorders ('new medical history'): Gardasil vs. AAHS (males, age 15-27; from 7-36 months)**						
V501-020: Gardasil vs. AAHS (m, 15-27; 36)	16	2032	18	2033	0.0%	0.89 [0.45, 1.74]
**Subtotal (95% CI)**		**0**		**0**		**Not estimable**
Total events	0		0			
Heterogeneity: Not applicable						
Test for overall effect: Not applicable						
**11.7.14 New onset eye disorders ('new medical history'): HPV 16 vaccine vs. AAHS (females, age 16-25; from 0-7 months)**						
V501-005: HPV 16 vaccine vs. AAHS (f, 16-25; 48)	13	1204	12	1205	4.0%	1.08 [0.50, 2.37]
**Subtotal (95% CI)**		**1204**		**1205**	**4.0%**	**1.08 [0.50, 2.37]**
Total events	13		12			
Heterogeneity: Not applicable						
Test for overall effect: Z = 0.20 (P = 0.84)						
**11.7.15 New onset eye disorders ('new medical history'): HPV 16 vaccine vs. AAHS (females, age 16-25; from 7-48 months)**						
V501-005: HPV 16 vaccine vs. AAHS (f, 16-25; 48)	12	1204	15	1205	0.0%	0.80 [0.38, 1.70]
**Subtotal (95% CI)**		**0**		**0**		**Not estimable**
Total events	0		0			
Heterogeneity: Not applicable						
Test for overall effect: Not applicable						
**11.7.16 New onset eye disorders ('new medical history'): Gardasil 9 vs. saline placebo (females, age 12-26; follow-up until 7 months)**						
V503-006: Gardasil 9 vs. saline (f, 12-26;7)	3	618	2	306	0.8%	0.74 [0.12, 4.42]
**Subtotal (95% CI)**		**618**		**306**	**0.8%**	**0.74 [0.12, 4.42]**
Total events	3		2			
Heterogeneity: Not applicable						
Test for overall effect: Z = 0.33 (P = 0.74)						
**Total (95% CI)**		**30252**		**29276**	**100.0%**	**0.89 [0.76, 1.04]**
Total events	296		326			
Heterogeneity: Tau^2 = 0.00; Chi^2 = 7.61, df = 15 (P = 0.94); I^2 = 0%						
Test for overall effect: Z = 1.48 (P = 0.14)						
Test for subgroup differences: Chi^2 = 4.54, df = 12 (P = 0.97), I^2 = 0%						

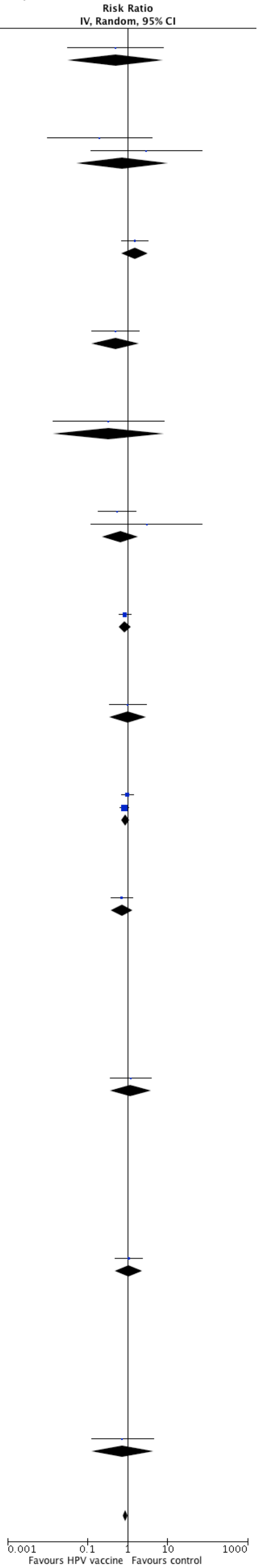

*11.7. Risk ratio for 'medically significant conditions’ (GlaxoSmithKline): 0.88 [0.67, 1.17]; risk ratio for ‘new medical history’ (Merck Sharp & Dohme): 0.89 [0.74, 1.08]; risk ratio for the follow-up periods for the trials V501-005, V501-019 and V501-020: 1.03 [0.75, 1.41]. The trials V501-005, V501-019 and V501-020 split the reporting of new onset diseases into the vaccination period and the follow-up period. To avoid double counting of participants in the total risk ratio estimate, we only included the new onset diseases reported in the vaccination period for the trials V501-005, V501-019 and V501-020.

11.8. New onset diseases ('medically significant conditions' and 'new medical history'*) reported within the MedDRA system organ class ‘gastrointestinal disorders (10017947)': intention to treat analysis

Study or Subgroup	HPV vaccine Events	HPV vaccine Total	Control Events	Control Total	Weight	Risk Ratio IV, Random, 95% CI
**11.8.1 New onset gastrointestinal disorders ('medically significant conditions'): Cervarix vs. Al(OH)3 (females, age 9-17; follow-up until 12 months)**						
HPV-058: Cervarix vs. Al(OH)3 (f, 9-17; 12)	3	374	1	376	0.2%	3.02 [0.32, 28.86]
**Subtotal (95% CI)**		**374**		**376**	**0.2%**	**3.02 [0.32, 28.86]**
Total events	3		1			
Heterogeneity: Not applicable						
Test for overall effect: Z = 0.96 (P = 0.34)						
**11.8.2 New onset gastrointestinal disorders ('medically significant conditions'): Cervarix vs. Al(OH)3 (females, age 15-25; follow-up until 7 months)**						
HPV-038: Cervarix vs. Al(OH)3 (f, 15-25; 7)	10	149	3	76	0.6%	1.70 [0.48, 6.00]
**Subtotal (95% CI)**		**149**		**76**	**0.6%**	**1.70 [0.48, 6.00]**
Total events	10		3			
Heterogeneity: Not applicable						
Test for overall effect: Z = 0.83 (P = 0.41)						
**11.8.3 New onset gastrointestinal disorders ('medically significant conditions'): Cervarix vs. Al(OH)3 (females, age 18-35; follow-up until 7 months)**						
HPV-031: Cervarix vs. Al(OH)3 (f, 18-35; 7)	2	176	2	178	0.2%	1.01 [0.14, 7.10]
HPV-035: Cervarix vs. Al(OH)3 (f, 18-35; 7)	9	150	3	150	0.6%	3.00 [0.83, 10.86]
**Subtotal (95% CI)**		**326**		**328**	**0.8%**	**2.16 [0.74, 6.31]**
Total events	11		5			
Heterogeneity: $Tau^2 = 0.00$; $Chi^2 = 0.83$, df = 1 (P = 0.36); $I^2 = 0\%$						
Test for overall effect: Z = 1.40 (P = 0.16)						
**11.8.4 New onset gastrointestinal disorders ('medically significant conditions'): Cervarix vs. Al(OH)3 (females, age 24-72; follow-up until 36 months)**						
HPV-015: Cervarix vs. Al(OH)3 (f, 24-72; 36)	250	2882	203	2871	13.5%	1.23 [1.03, 1.47]
**Subtotal (95% CI)**		**2882**		**2871**	**13.5%**	**1.23 [1.03, 1.47]**
Total events	250		203			
Heterogeneity: Not applicable						
Test for overall effect: Z = 2.25 (P = 0.02)						
**11.8.5 New onset gastrointestinal disorders ('medically significant conditions'): Cervarix vs. Aimmugen: hepatitis A vaccine (females, age 22-29; follow-up until 12 months)**						
HPV-063: Cervarix vs. Aimmugen (f, 22-29; 12)	12	375	33	377	2.1%	0.37 [0.19, 0.70]
**Subtotal (95% CI)**		**375**		**377**	**2.1%**	**0.37 [0.19, 0.70]**
Total events	12		33			
Heterogeneity: Not applicable						
Test for overall effect: Z = 3.06 (P = 0.002)						
**11.8.6 New onset gastrointestinal disorders ('medically significant conditions'): Cervarix vs. Engerix: hepatitis B vaccine (females and males, age 9-15; follow-up until 12 months)**						
HPV-030: Cervarix vs. Engerix (f, 9-15; 12)	2	247	5	246	0.4%	0.40 [0.08, 2.03]
**Subtotal (95% CI)**		**247**		**246**	**0.4%**	**0.40 [0.08, 2.03]**
Total events	2		5			
Heterogeneity: Not applicable						
Test for overall effect: Z = 1.11 (P = 0.27)						
**11.8.7 New onset gastrointestinal disorders ('medically significant conditions'): Cervarix vs. Havrix: hepatitis A vaccine (females, age 9-15; follow-up until 12 months)**						
HPV-013: Cervarix vs. Havrix (f, 10-14; 12)	22	1035	16	1032	2.1%	1.37 [0.72, 2.60]
HPV-033: Cervarix vs. Havrix (f, 9-15; 7)	3	160	3	161	0.4%	1.01 [0.21, 4.91]
**Subtotal (95% CI)**		**1195**		**1193**	**2.5%**	**1.31 [0.73, 2.37]**
Total events	25		19			
Heterogeneity: $Tau^2 = 0.00$; $Chi^2 = 0.13$, df = 1 (P = 0.72); $I^2 = 0\%$						
Test for overall effect: Z = 0.90 (P = 0.37)						
**11.8.8 New onset gastrointestinal disorders ('medically significant conditions'): Cervarix vs. Havrix: hepatitis A vaccine (females, age 15-25; follow-up until 48 months)**						
HPV-008: Cervarix vs. Havrix (f, 15-25; 48)	346	9319	327	9325	15.6%	1.06 [0.91, 1.23]
**Subtotal (95% CI)**		**9319**		**9325**	**15.6%**	**1.06 [0.91, 1.23]**
Total events	346		327			
Heterogeneity: Not applicable						
Test for overall effect: Z = 0.75 (P = 0.45)						
**11.8.9 New onset gastrointestinal disorders ('medically significant conditions'): Cervarix vs. Twinrix: hepatitis A and B vaccine (females, age 8-15; follow-up until 12 months)**						
HPV-029: Cervarix vs. Twinrix (f, 8-15; 12)	1	270	2	271	0.2%	0.50 [0.05, 5.50]
**Subtotal (95% CI)**		**270**		**271**	**0.2%**	**0.50 [0.05, 5.50]**
Total events	1		2			
Heterogeneity: Not applicable						
Test for overall effect: Z = 0.56 (P = 0.57)						
**11.8.10 New onset gastrointestinal disorders ('new medical history'): Gardasil vs. carrier solution (females and males, age 9-16; follow-up until 18 months)**						
V501-018: Gardasil vs. carrier solu. (f&m,9-16;18)	44	1184	19	597	3.0%	1.17 [0.69, 1.98]
**Subtotal (95% CI)**		**1184**		**597**	**3.0%**	**1.17 [0.69, 1.98]**
Total events	44		19			
Heterogeneity: Not applicable						
Test for overall effect: Z = 0.57 (P = 0.57)						
**11.8.11 New onset gastrointestinal disorders ('new medical history'): Gardasil vs. AAHS (females, age 15-26; follow-up until 45 months)**						
V501-013: Gardasil vs. AAHS (f, 16-24; 45)	469	2723	457	2732	18.1%	1.03 [0.92, 1.16]
V501-015: Gardasil vs. AAHS (f, 15-26; 36)	785	6087	834	6080	20.2%	0.94 [0.86, 1.03]
**Subtotal (95% CI)**		**8810**		**8812**	**38.3%**	**0.98 [0.89, 1.07]**
Total events	1254		1291			
Heterogeneity: $Tau^2 = 0.00$; $Chi^2 = 1.44$, df = 1 (P = 0.23); $I^2 = 31\%$						
Test for overall effect: Z = 0.54 (P = 0.59)						
**11.8.12 New onset gastrointestinal disorders ('new medical history'): Gardasil vs. AAHS (females, age 21-46; from 0-7 months)**						
V501-019: Gardasil vs. AAHS (f, 21-46; 48)	92	1911	84	1908	7.8%	1.09 [0.82, 1.46]
**Subtotal (95% CI)**		**1911**		**1908**	**7.8%**	**1.09 [0.82, 1.46]**
Total events	92		84			
Heterogeneity: Not applicable						
Test for overall effect: Z = 0.61 (P = 0.54)						
**11.8.13 New onset gastrointestinal disorders ('new medical history'): Gardasil vs. AAHS (females, age 21-46; from 7-48 months)**						
V501-019: Gardasil vs. AAHS (f, 21-46; 48)	176	1911	180	1908	0.0%	0.98 [0.80, 1.19]
**Subtotal (95% CI)**		**0**		**0**		**Not estimable**
Total events	0		0			
Heterogeneity: Not applicable						
Test for overall effect: Not applicable						
**11.8.14 New onset gastrointestinal disorders ('new medical history'): Gardasil vs. AAHS (males, age 15-27; from 0-7 months)**						
V501-020: Gardasil vs. AAHS (m, 15-27; 36)	73	2032	57	2032	6.1%	1.28 [0.91, 1.80]
**Subtotal (95% CI)**		**2032**		**2032**	**6.1%**	**1.28 [0.91, 1.80]**
Total events	73		57			
Heterogeneity: Not applicable						
Test for overall effect: Z = 1.42 (P = 0.15)						
**11.8.15 New onset gastrointestinal disorders ('new medical history'): Gardasil vs. AAHS (males, age 15-27; from 7-36 months)**						
V501-020: Gardasil vs. AAHS (m, 15-27; 36)	84	2032	93	2033	0.0%	0.90 [0.68, 1.21]
**Subtotal (95% CI)**		**0**		**0**		**Not estimable**
Total events	0		0			
Heterogeneity: Not applicable						
Test for overall effect: Not applicable						
**11.8.16 New onset gastrointestinal disorders ('new medical history'): HPV 16 vaccine vs. AAHS (females, age 16-25; from 0-7 months)**						
V501-005: HPV 16 vaccine vs. AAHS (f, 16-25; 48)	81	1204	83	1205	7.5%	0.98 [0.73, 1.31]
**Subtotal (95% CI)**		**1204**		**1205**	**7.5%**	**0.98 [0.73, 1.31]**
Total events	81		83			
Heterogeneity: Not applicable						
Test for overall effect: Z = 0.16 (P = 0.88)						
**11.8.17 New onset gastrointestinal disorders ('new medical history'): HPV 16 vaccine vs. AAHS (females, age 16-25; from 7-48 months)**						
V501-005: HPV 16 vaccine vs. AAHS (f, 16-25; 48)	186	1204	187	1205	0.0%	1.00 [0.83, 1.20]
**Subtotal (95% CI)**		**0**		**0**		**Not estimable**
Total events	0		0			
Heterogeneity: Not applicable						
Test for overall effect: Not applicable						
**11.8.18 New onset gastrointestinal disorders ('new medical history'): Gardasil 9 vs. saline placebo (females, age 12-26; follow-up until 7 months)**						
V503-006: Gardasil 9 vs. saline (f, 12-26;7)	22	618	9	306	1.5%	1.21 [0.56, 2.60]
**Subtotal (95% CI)**		**618**		**306**	**1.5%**	**1.21 [0.56, 2.60]**
Total events	22		9			
Heterogeneity: Not applicable						
Test for overall effect: Z = 0.49 (P = 0.62)						
**Total (95% CI)**		**30896**		**29923**	**100.0%**	**1.05 [0.95, 1.16]**
Total events	2226		2141			
Heterogeneity: $Tau^2 = 0.01$; $Chi^2 = 26.34$, df = 17 (P = 0.07); $I^2 = 35\%$						
Test for overall effect: Z = 1.00 (P = 0.32)						
Test for subgroup differences: $Chi^2 = 23.00$, df = 14 (P = 0.06), $I^2 = 39.1\%$						

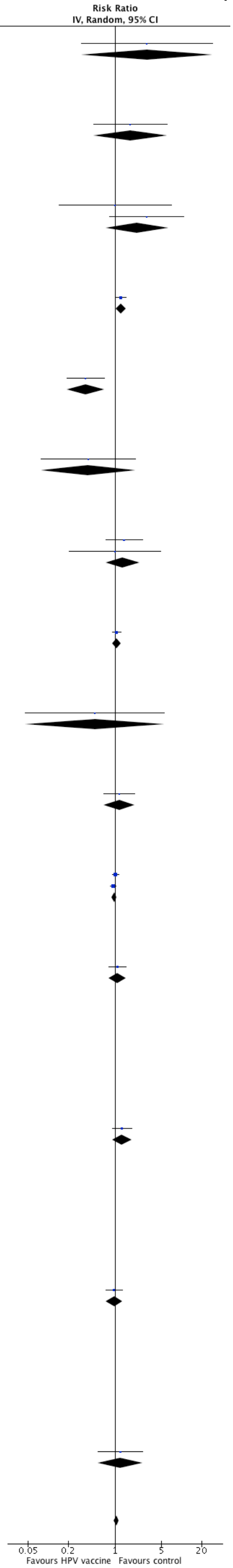

*11.8. Risk ratio for 'medically significant conditions’ (GlaxoSmithKline): 1.05 [0.81, 1.36]; risk ratio for ‘new medical history’ (Merck Sharp & Dohme): 0.99 [0.93, 1.06]; risk ratio for the follow-up periods for the trials V501-005, V501-019 and V501-020: 0.97 [0.86, 1.10]. The trials V501-005, V501-019 and V501-020 split the reporting of new onset diseases into the vaccination period and the follow-up period. To avoid double counting of participants in the total risk ratio estimate, we only included the new onset diseases reported in the vaccination period for the trials V501-005, V501-019 and V501-020.

11.9. New onset diseases ('medically significant conditions' and 'new medical history'*) reported within the MedDRA system organ class ‘general disorders and administration site conditions (10018065)': intention to treat analysis

Study or Subgroup	HPV vaccine Events	HPV vaccine Total	Control Events	Control Total	Weight	Risk Ratio IV, Random, 95% CI
**11.9.1 New onset general disorders and administration site conditions ('medically significant conditions'): Cervarix vs. Al(OH)3 (females, age 9-17; follow-up until 12 months)**						
HPV-058: Cervarix vs. Al(OH)3 (f, 9-17; 12)	1	374	2	376	0.3%	0.50 [0.05, 5.52]
**Subtotal (95% CI)**		**374**		**376**	**0.3%**	**0.50 [0.05, 5.52]**
Total events	1		2			
Heterogeneity: Not applicable						
Test for overall effect: Z = 0.56 (P = 0.57)						
**11.9.2 New onset general disorders and administration site conditions ('medically significant conditions'): Cervarix vs. Al(OH)3 (females, age 18-35; follow-up until 7 months)**						
HPV-031: Cervarix vs. Al(OH)3 (f, 18-35; 7)	2	176	0	178	0.2%	5.06 [0.24, 104.58]
HPV-035: Cervarix vs. Al(OH)3 (f, 18-35; 7)	2	150	1	150	0.3%	2.00 [0.18, 21.82]
**Subtotal (95% CI)**		**326**		**328**	**0.4%**	**2.85 [0.44, 18.64]**
Total events	4		1			
Heterogeneity: Tau^2 = 0.00; Chi^2 = 0.22, df = 1 (P = 0.64); I^2 = 0%						
Test for overall effect: Z = 1.10 (P = 0.27)						
**11.9.3 New onset general disorders and administration site conditions ('medically significant conditions'): Cervarix vs. Al(OH)3 (females, age 24-72; follow-up until 36 months)**						
HPV-015: Cervarix vs. Al(OH)3 (f, 24-72; 36)	63	2882	50	2871	10.8%	1.26 [0.87, 1.81]
**Subtotal (95% CI)**		**2882**		**2871**	**10.8%**	**1.26 [0.87, 1.81]**
Total events	63		50			
Heterogeneity: Not applicable						
Test for overall effect: Z = 1.21 (P = 0.23)						
**11.9.4 New onset general disorders and administration site conditions ('medically significant conditions'): Cervarix vs. Aimmugen: hepatitis A vaccine (females, age 22-29; follow-up until 12 months)**						
HPV-063: Cervarix vs. Aimmugen (f, 22-29; 12)	3	375	7	377	0.8%	0.43 [0.11, 1.65]
**Subtotal (95% CI)**		**375**		**377**	**0.8%**	**0.43 [0.11, 1.65]**
Total events	3		7			
Heterogeneity: Not applicable						
Test for overall effect: Z = 1.23 (P = 0.22)						
**11.9.5 New onset general disorders and administration site conditions ('medically significant conditions'): Cervarix vs. Engerix: hepatitis B vaccine (females and males, age 9-15; follow-up until 12 months)**						
HPV-030: Cervarix vs. Engerix (f, 9-15; 12)	3	247	4	246	0.7%	0.75 [0.17, 3.30]
**Subtotal (95% CI)**		**247**		**246**	**0.7%**	**0.75 [0.17, 3.30]**
Total events	3		4			
Heterogeneity: Not applicable						
Test for overall effect: Z = 0.38 (P = 0.70)						
**11.9.6 New onset general disorders and administration site conditions ('medically significant conditions'): Cervarix vs. Havrix: hepatitis A vaccine (females, age 9-15; follow-up until 12 months)**						
HPV-013: Cervarix vs. Havrix (f, 10-14; 12)	10	1035	8	1032	1.7%	1.25 [0.49, 3.15]
HPV-033: Cervarix vs. Havrix (f, 9-15; 7)	2	160	0	161	0.2%	5.03 [0.24, 103.97]
**Subtotal (95% CI)**		**1195**		**1193**	**1.9%**	**1.40 [0.58, 3.40]**
Total events	12		8			
Heterogeneity: Tau^2 = 0.00; Chi^2 = 0.75, df = 1 (P = 0.39); I^2 = 0%						
Test for overall effect: Z = 0.75 (P = 0.45)						
**11.9.7 New onset general disorders and administration site conditions ('medically significant conditions'): Cervarix vs. Havrix: hepatitis A vaccine (females, age 15-25; follow-up until 48 months)**						
HPV-008: Cervarix vs. Havrix (f, 15-25; 48)	63	9319	67	9325	12.4%	0.94 [0.67, 1.33]
**Subtotal (95% CI)**		**9319**		**9325**	**12.4%**	**0.94 [0.67, 1.33]**
Total events	63		67			
Heterogeneity: Not applicable						
Test for overall effect: Z = 0.35 (P = 0.73)						
**11.9.8 New onset general disorders and administration site conditions ('medically significant conditions'): Cervarix vs. Twinrix: hepatitis A and B vaccine (females, age 8-15; follow-up until 12 months)**						
HPV-029: Cervarix vs. Twinrix (f, 8-15; 12)	3	270	0	271	0.2%	7.03 [0.36, 135.37]
**Subtotal (95% CI)**		**270**		**271**	**0.2%**	**7.03 [0.36, 135.37]**
Total events	3		0			
Heterogeneity: Not applicable						
Test for overall effect: Z = 1.29 (P = 0.20)						
**11.9.9 New onset general disorders and administration site conditions ('new medical history'): Gardasil vs. carrier solution (females and males, age 9-16; follow-up until 18 months)**						
V501-018: Gardasil vs. carrier solu. (f&m,9-16;18)	10	1184	6	597	1.4%	0.84 [0.31, 2.30]
**Subtotal (95% CI)**		**1184**		**597**	**1.4%**	**0.84 [0.31, 2.30]**
Total events	10		6			
Heterogeneity: Not applicable						
Test for overall effect: Z = 0.34 (P = 0.74)						
**11.9.10 New onset general disorders and administration site conditions ('new medical history'): Gardasil vs. AAHS (females, age 15-26; follow-up until 45 months)**						
V501-013: Gardasil vs. AAHS (f, 16-24; 45)	142	2723	121	2732	26.0%	1.18 [0.93, 1.49]
V501-015: Gardasil vs. AAHS (f, 15-26; 36)	197	6087	170	6080	35.6%	1.16 [0.95, 1.42]
**Subtotal (95% CI)**		**8810**		**8812**	**61.6%**	**1.17 [1.00, 1.36]**
Total events	339		291			
Heterogeneity: Tau^2 = 0.00; Chi^2 = 0.01, df = 1 (P = 0.91); I^2 = 0%						
Test for overall effect: Z = 1.96 (P = 0.05)						
**11.9.11 New onset general disorders and administration site conditions ('new medical history'): Gardasil vs. AAHS (females, age 21-46; from 0-7 months)**						
V501-019: Gardasil vs. AAHS (f, 21-46; 48)	26	1911	24	1908	4.8%	1.08 [0.62, 1.88]
**Subtotal (95% CI)**		**1911**		**1908**	**4.8%**	**1.08 [0.62, 1.88]**
Total events	26		24			
Heterogeneity: Not applicable						
Test for overall effect: Z = 0.28 (P = 0.78)						
**11.9.12 New onset general disorders and administration site conditions ('new medical history'): Gardasil vs. AAHS (females, age 21-46; from 7-48 months)**						
V501-019: Gardasil vs. AAHS (f, 21-46; 48)	23	1911	28	1908	0.0%	0.82 [0.47, 1.42]
**Subtotal (95% CI)**		**0**		**0**		**Not estimable**
Total events	0		0			
Heterogeneity: Not applicable						
Test for overall effect: Not applicable						
**11.9.13 New onset general disorders and administration site conditions ('new medical history'): Gardasil vs. AAHS (males, age 15-27; from 0-7 months)**						
V501-020: Gardasil vs. AAHS (m, 15-27; 36)	12	2032	6	2032	1.5%	2.00 [0.75, 5.32]
**Subtotal (95% CI)**		**2032**		**2032**	**1.5%**	**2.00 [0.75, 5.32]**
Total events	12		6			
Heterogeneity: Not applicable						
Test for overall effect: Z = 1.39 (P = 0.16)						
**11.9.14 New onset general disorders and administration site conditions ('new medical history'): Gardasil vs. AAHS (males, age 15-27; from 7-36 months)**						
V501-020: Gardasil vs. AAHS (m, 15-27; 36)	7	2032	9	2033	0.0%	0.78 [0.29, 2.09]
**Subtotal (95% CI)**		**0**		**0**		**Not estimable**
Total events	0		0			
Heterogeneity: Not applicable						
Test for overall effect: Not applicable						
**11.9.15 New onset general disorders and administration site conditions ('new medical history'): HPV 16 vaccine vs. AAHS (females, age 16-25; from 0-7 months)**						
V501-005: HPV 16 vaccine vs. AAHS (f, 16-25; 48)	13	1204	15	1205	2.7%	0.87 [0.41, 1.82]
**Subtotal (95% CI)**		**1204**		**1205**	**2.7%**	**0.87 [0.41, 1.82]**
Total events	13		15			
Heterogeneity: Not applicable						
Test for overall effect: Z = 0.38 (P = 0.71)						
**11.9.16 New onset general disorders and administration site conditions ('new medical history'): HPV 16 vaccine vs. AAHS (females, age 16-25; from 7-48 months)**						
V501-005: HPV 16 vaccine vs. AAHS (f, 16-25; 48)	18	1204	23	1205	0.0%	0.78 [0.42, 1.44]
**Subtotal (95% CI)**		**0**		**0**		**Not estimable**
Total events	0		0			
Heterogeneity: Not applicable						
Test for overall effect: Not applicable						
**11.9.17 New onset general disorders and administration site conditions ('new medical history'): Gardasil 9 vs. saline placebo (females, age 12-26; follow-up until 7 months)**						
V503-006: Gardasil 9 vs. saline (f, 12-26;7)	11	618	2	306	0.6%	2.72 [0.61, 12.21]
**Subtotal (95% CI)**		**618**		**306**	**0.6%**	**2.72 [0.61, 12.21]**
Total events	11		2			
Heterogeneity: Not applicable						
Test for overall effect: Z = 1.31 (P = 0.19)						
**Total (95% CI)**		**30747**		**29847**	**100.0%**	**1.14 [1.01, 1.28]**
Total events	563		483			
Heterogeneity: Tau^2 = 0.00; Chi^2 = 11.36, df = 16 (P = 0.79); I^2 = 0%						
Test for overall effect: Z = 2.10 (P = 0.04)						
Test for subgroup differences: Chi^2 = 10.38, df = 13 (P = 0.66), I^2 = 0%						

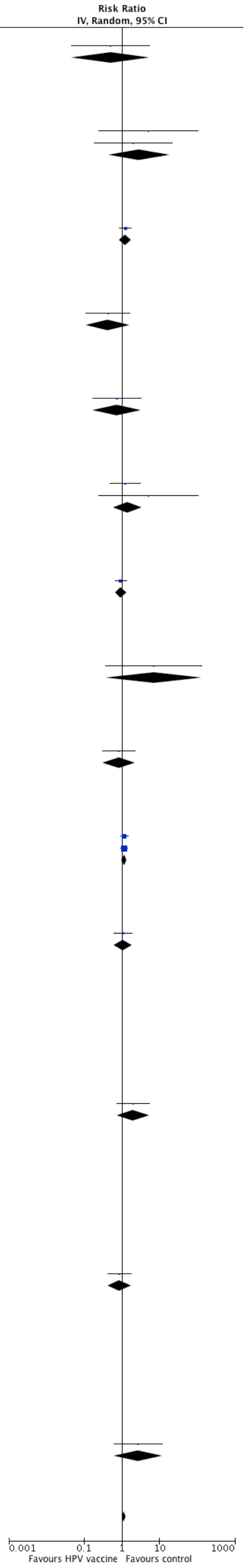

*11.9. Risk ratio for 'medically significant conditions’ (GlaxoSmithKline): 1.08 [0.86, 1.36]; risk ratio for ‘new medical history’ (Merck Sharp & Dohme): **1.16 [1.01, 1.34]**; risk ratio for the follow-up periods for the trials V501-005, V501-019 and V501-020: 0.80 [0.55, 1.17]. The trials V501-005, V501-019 and V501-020 split the reporting of new onset diseases into the vaccination period and the follow-up period. To avoid double counting of participants in the total risk ratio estimate, we only included the new onset diseases reported in the vaccination period for the trials V501-005, V501-019 and V501-020.

11.10. New onset diseases ('medically significant conditions' and 'new medical history'*) reported within the MedDRA system organ class ‘hepatobiliary disorders (10019805)': intention to treat analysis

Study or Subgroup	HPV vaccine Events	HPV vaccine Total	Control Events	Control Total	Weight	Risk Ratio IV, Random, 95% CI
**11.10.1 New onset hepatobiliary disorders ('medically significant conditions'): Cervarix vs. Al(OH)3 (females, age 24-72; follow-up until 36 months)**						
HPV-015: Cervarix vs. Al(OH)3 (f, 24-72; 36)	38	2882	44	2871	26.6%	0.86 [0.56, 1.32]
**Subtotal (95% CI)**		**2882**		**2871**	**26.6%**	**0.86 [0.56, 1.32]**
Total events	38		44			
Heterogeneity: Not applicable						
Test for overall effect: Z = 0.68 (P = 0.49)						
**11.10.2 New onset hepatobiliary disorders ('medically significant conditions'): Cervarix vs. Aimmugen: hepatitis A vaccine (females, age 22-29; follow-up until 12 months)**						
HPV-063: Cervarix vs. Aimmugen (f, 22-29; 12)	1	375	2	377	2.0%	0.50 [0.05, 5.52]
**Subtotal (95% CI)**		**375**		**377**	**2.0%**	**0.50 [0.05, 5.52]**
Total events	1		2			
Heterogeneity: Not applicable						
Test for overall effect: Z = 0.56 (P = 0.57)						
**11.10.3 New onset hepatobiliary disorders ('medically significant conditions'): Cervarix vs. Engerix: hepatitis B vaccine (females, age 26-46; follow-up until 12 months)**						
HPV-069: Cervarix vs. Engerix (f, 26-46; 12)	2	606	1	606	2.0%	2.00 [0.18, 22.00]
**Subtotal (95% CI)**		**606**		**606**	**2.0%**	**2.00 [0.18, 22.00]**
Total events	2		1			
Heterogeneity: Not applicable						
Test for overall effect: Z = 0.57 (P = 0.57)						
**11.10.4 New onset hepatobiliary disorders ('medically significant conditions'): Cervarix vs. Havrix: hepatitis A vaccine (females, age 10-14; follow-up until 12 months)**						
HPV-013: Cervarix vs. Havrix (f, 10-14; 12)	0	1035	1	1032	1.2%	0.33 [0.01, 8.15]
**Subtotal (95% CI)**		**1035**		**1032**	**1.2%**	**0.33 [0.01, 8.15]**
Total events	0		1			
Heterogeneity: Not applicable						
Test for overall effect: Z = 0.67 (P = 0.50)						
**11.10.5 New onset hepatobiliary disorders ('medically significant conditions'): Cervarix vs. Havrix: hepatitis A vaccine (females, age 15-25; follow-up until 48 months)**						
HPV-008: Cervarix vs. Havrix (f, 15-25; 48)	41	9319	40	9325	26.4%	1.03 [0.66, 1.58]
**Subtotal (95% CI)**		**9319**		**9325**	**26.4%**	**1.03 [0.66, 1.58]**
Total events	41		40			
Heterogeneity: Not applicable						
Test for overall effect: Z = 0.11 (P = 0.91)						
**11.10.6 New onset hepatobiliary disorders ('new medical history'): Gardasil vs. AAHS (females, age 15-26; follow-up until 45 months)**						
V501-013: Gardasil vs. AAHS (f, 16-24; 45)	10	2723	29	2732	15.5%	0.35 [0.17, 0.71]
V501-015: Gardasil vs. AAHS (f, 15-26; 36)	30	6087	24	6080	21.8%	1.25 [0.73, 2.13]
**Subtotal (95% CI)**		**8810**		**8812**	**37.3%**	**0.67 [0.19, 2.36]**
Total events	40		53			
Heterogeneity: Tau^2 = 0.72; Chi^2 = 7.90, df = 1 (P = 0.005); I^2 = 87%						
Test for overall effect: Z = 0.62 (P = 0.54)						
**11.10.7 New onset hepatobiliary disorders ('new medical history'): Gardasil vs. AAHS (females, age 21-46; from 0-7 months)**						
V501-019: Gardasil vs. AAHS (f, 21-46; 48)	2	1911	0	1908	1.3%	4.99 [0.24, 103.91]
**Subtotal (95% CI)**		**1911**		**1908**	**1.3%**	**4.99 [0.24, 103.91]**
Total events	2		0			
Heterogeneity: Not applicable						
Test for overall effect: Z = 1.04 (P = 0.30)						
**11.10.8 New onset hepatobiliary disorders ('new medical history'): Gardasil vs. AAHS (females, age 21-46; from 7-48 months)**						
V501-019: Gardasil vs. AAHS (f, 21-46; 48)	12	1911	10	1908	0.0%	1.20 [0.52, 2.77]
**Subtotal (95% CI)**		**0**		**0**		**Not estimable**
Total events	0		0			
Heterogeneity: Not applicable						
Test for overall effect: Not applicable						
**11.10.9 New onset hepatobiliary disorders ('new medical history'): Gardasil vs. AAHS (males, age 15-27; from 7-36 months)**						
V501-020: Gardasil vs. AAHS (m, 15-27; 36)	2	2032	1	2033	2.0%	2.00 [0.18, 22.05]
**Subtotal (95% CI)**		**2032**		**2033**	**2.0%**	**2.00 [0.18, 22.05]**
Total events	2		1			
Heterogeneity: Not applicable						
Test for overall effect: Z = 0.57 (P = 0.57)						
**11.10.10 New onset hepatobiliary disorders ('new medical history'): Gardasil 9 vs. saline placebo (females, age 12-26; follow-up until 7 months)**						
V503-006: Gardasil 9 vs. saline (f, 12-26;7)	0	618	1	306	1.2%	0.17 [0.01, 4.05]
**Subtotal (95% CI)**		**618**		**306**	**1.2%**	**0.17 [0.01, 4.05]**
Total events	0		1			
Heterogeneity: Not applicable						
Test for overall effect: Z = 1.10 (P = 0.27)						
**Total (95% CI)**		**27588**		**27270**	**100.0%**	**0.86 [0.61, 1.22]**
Total events	126		143			
Heterogeneity: Tau^2 = 0.07; Chi^2 = 12.41, df = 9 (P = 0.19); I^2 = 27%						
Test for overall effect: Z = 0.83 (P = 0.40)						
Test for subgroup differences: Chi^2 = 4.31, df = 8 (P = 0.83), I^2 = 0%						

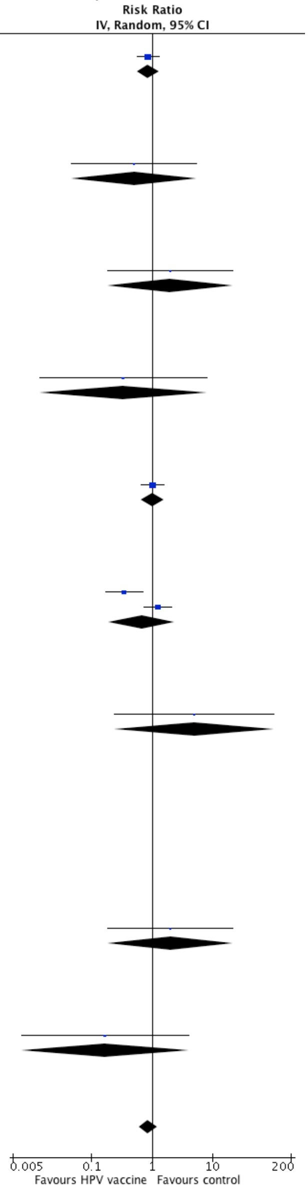

*11.10. Risk ratio for 'medically significant conditions’ (GlaxoSmithKline): 0.93 [0.69, 1.26]; risk ratio for ‘new medical history’ (Merck Sharp & Dohme): 0.81 [0.31, 2.15]; risk ratio for the follow-up period for the trial V501-019: 1.20 [0.52, 2.77]. The trial V501-019 split the reporting of new onset diseases into the vaccination period and the follow-up period. To avoid double counting of participants in the total risk ratio estimate, we only included the new onset diseases reported in the vaccination period for the trial V501-019.

11.11. New onset diseases ('medically significant conditions' and 'new medical history'*) reported within the MedDRA system organ class ‘immune system disorders (10021428)': intention to treat analysis

Study or Subgroup	HPV vaccine Events	HPV vaccine Total	Control Events	Control Total	Weight	Risk Ratio IV, Random, 95% CI
**11.11.1 New onset immune system disorders ('medically significant conditions'): Cervarix vs. Al(OH)3 (females, age 18-35; follow-up until 7 months)**						
HPV-035: Cervarix vs. Al(OH)3 (f, 18-35; 7)	2	150	1	150	0.5%	2.00 [0.18, 21.82]
**Subtotal (95% CI)**		**150**		**150**	**0.5%**	**2.00 [0.18, 21.82]**
Total events	2		1			
Heterogeneity: Not applicable						
Test for overall effect: Z = 0.57 (P = 0.57)						
**11.11.2 New onset immune system disorders ('medically significant conditions'): Cervarix vs. Al(OH)3 (females, age 24-72; follow-up until 36 months)**						
HPV-015: Cervarix vs. Al(OH)3 (f, 24-72; 36)	18	2882	29	2871	7.0%	0.62 [0.34, 1.11]
**Subtotal (95% CI)**		**2882**		**2871**	**7.0%**	**0.62 [0.34, 1.11]**
Total events	18		29			
Heterogeneity: Not applicable						
Test for overall effect: Z = 1.61 (P = 0.11)						
**11.11.3 New onset immune system disorders ('medically significant conditions'): Cervarix vs. Engerix: hepatitis B vaccine (females and males, age 9-15; follow-up until 12 months)**						
HPV-030: Cervarix vs. Engerix (f, 9-15; 12)	1	247	0	246	0.3%	2.99 [0.12, 72.99]
**Subtotal (95% CI)**		**247**		**246**	**0.3%**	**2.99 [0.12, 72.99]**
Total events	1		0			
Heterogeneity: Not applicable						
Test for overall effect: Z = 0.67 (P = 0.50)						
**11.11.4 New onset immune system disorders ('medically significant conditions'): Cervarix vs. Havrix: hepatitis A vaccine (females, age 10-14; follow-up until 12 months)**						
HPV-013: Cervarix vs. Havrix (f, 10-14; 12)	4	1035	4	1032	1.3%	1.00 [0.25, 3.98]
**Subtotal (95% CI)**		**1035**		**1032**	**1.3%**	**1.00 [0.25, 3.98]**
Total events	4		4			
Heterogeneity: Not applicable						
Test for overall effect: Z = 0.00 (P = 1.00)						
**11.11.5 New onset immune system disorders ('medically significant conditions'): Cervarix vs. Havrix: hepatitis A vaccine (females, age 15-25; follow-up until 48 months)**						
HPV-008: Cervarix vs. Havrix (f, 15-25; 48)	46	9319	77	9325	16.0%	0.60 [0.42, 0.86]
**Subtotal (95% CI)**		**9319**		**9325**	**16.0%**	**0.60 [0.42, 0.86]**
Total events	46		77			
Heterogeneity: Not applicable						
Test for overall effect: Z = 2.77 (P = 0.006)						
**11.11.6 New onset immune system disorders ('medically significant conditions'): Cervarix vs. Twinrix: hepatitis A and B vaccine (females, age 8-15; follow-up until 12 months)**						
HPV-029: Cervarix vs. Twinrix (f, 8-15; 12)	0	270	1	271	0.3%	0.33 [0.01, 8.18]
**Subtotal (95% CI)**		**270**		**271**	**0.3%**	**0.33 [0.01, 8.18]**
Total events	0		1			
Heterogeneity: Not applicable						
Test for overall effect: Z = 0.67 (P = 0.50)						
**11.11.7 New onset immune system disorders ('new medical history'): Gardasil vs. carrier solution (females and males, age 9-16; follow-up until 18 months)**						
V501-018: Gardasil vs. carrier solu. (f&m,9-16;18)	3	1184	4	597	1.1%	0.38 [0.08, 1.68]
**Subtotal (95% CI)**		**1184**		**597**	**1.1%**	**0.38 [0.08, 1.68]**
Total events	3		4			
Heterogeneity: Not applicable						
Test for overall effect: Z = 1.28 (P = 0.20)						
**11.11.8 New onset immune system disorders ('new medical history'): Gardasil vs. AAHS (females, age 15-26; follow-up until 45 months)**						
V501-013: Gardasil vs. AAHS (f, 16-24; 45)	94	2723	94	2732	23.9%	1.00 [0.76, 1.33]
V501-015: Gardasil vs. AAHS (f, 15-26; 36)	132	6087	123	6080	29.2%	1.07 [0.84, 1.37]
**Subtotal (95% CI)**		**8810**		**8812**	**53.1%**	**1.04 [0.87, 1.25]**
Total events	226		217			
Heterogeneity: $Tau^2 = 0.00$; $Chi^2 = 0.12$, df = 1 (P = 0.73); $I^2 = 0\%$						
Test for overall effect: Z = 0.44 (P = 0.66)						
**11.11.9 New onset immune system disorders ('new medical history'): Gardasil vs. AAHS (females, age 21-46; from 0-7 months)**						
V501-019: Gardasil vs. AAHS (f, 21-46; 48)	9	1911	9	1908	3.0%	1.00 [0.40, 2.51]
**Subtotal (95% CI)**		**1911**		**1908**	**3.0%**	**1.00 [0.40, 2.51]**
Total events	9		9			
Heterogeneity: Not applicable						
Test for overall effect: Z = 0.00 (P = 1.00)						
**11.11.10 New onset immune system disorders ('new medical history'): Gardasil vs. AAHS (females, age 21-46; from 7-48 months)**						
V501-019: Gardasil vs. AAHS (f, 21-46; 48)	12	1911	8	1908	0.0%	1.50 [0.61, 3.66]
**Subtotal (95% CI)**		**0**		**0**		**Not estimable**
Total events	0		0			
Heterogeneity: Not applicable						
Test for overall effect: Not applicable						
**11.11.11 New onset immune system disorders ('new medical history'): Gardasil vs. AAHS (males, age 15-27; from 0-7 months)**						
V501-020: Gardasil vs. AAHS (m, 15-27; 36)	11	2032	8	2032	3.0%	1.38 [0.55, 3.41]
**Subtotal (95% CI)**		**2032**		**2032**	**3.0%**	**1.38 [0.55, 3.41]**
Total events	11		8			
Heterogeneity: Not applicable						
Test for overall effect: Z = 0.69 (P = 0.49)						
**11.11.12 New onset immune system disorders ('new medical history'): Gardasil vs. AAHS (males, age 15-27; from 7-36 months)**						
V501-020: Gardasil vs. AAHS (m, 15-27; 36)	3	2032	4	2033	0.0%	0.75 [0.17, 3.35]
**Subtotal (95% CI)**		**0**		**0**		**Not estimable**
Total events	0		0			
Heterogeneity: Not applicable						
Test for overall effect: Not applicable						
**11.11.13 New onset immune system disorders ('new medical history'): HPV 16 vaccine vs. AAHS (females, age 16-25; from 0-7 months)**						
V501-005: HPV 16 vaccine vs. AAHS (f, 16-25; 48)	49	1204	48	1205	14.3%	1.02 [0.69, 1.51]
**Subtotal (95% CI)**		**1204**		**1205**	**14.3%**	**1.02 [0.69, 1.51]**
Total events	49		48			
Heterogeneity: Not applicable						
Test for overall effect: Z = 0.11 (P = 0.91)						
**11.11.14 New onset immune system disorders ('new medical history'): HPV 16 vaccine vs. AAHS (females, age 16-25; from 7-48 months)**						
V501-005: HPV 16 vaccine vs. AAHS (f, 16-25; 48)	48	1204	54	1205	0.0%	0.89 [0.61, 1.30]
**Subtotal (95% CI)**		**0**		**0**		**Not estimable**
Total events	0		0			
Heterogeneity: Not applicable						
Test for overall effect: Not applicable						
**11.11.15 New onset immune system disorders ('new medical history'): Gardasil 9 vs. saline placebo (females, age 12-26; follow-up until 7 months)**						
V503-006: Gardasil 9 vs. saline (f, 12-26;7)	2	618	0	306	0.3%	2.48 [0.12, 51.49]
**Subtotal (95% CI)**		**618**		**306**	**0.3%**	**2.48 [0.12, 51.49]**
Total events	2		0			
Heterogeneity: Not applicable						
Test for overall effect: Z = 0.59 (P = 0.56)						
**Total (95% CI)**		**29662**		**28755**	**100.0%**	**0.92 [0.78, 1.08]**
Total events	371		398			
Heterogeneity: $Tau^2 = 0.01$; $Chi^2 = 13.18$, df = 12 (P = 0.36); $I^2 = 9\%$						
Test for overall effect: Z = 1.06 (P = 0.29)						
Test for subgroup differences: $Chi^2 = 13.06$, df = 11 (P = 0.29), $I^2 = 15.8\%$						

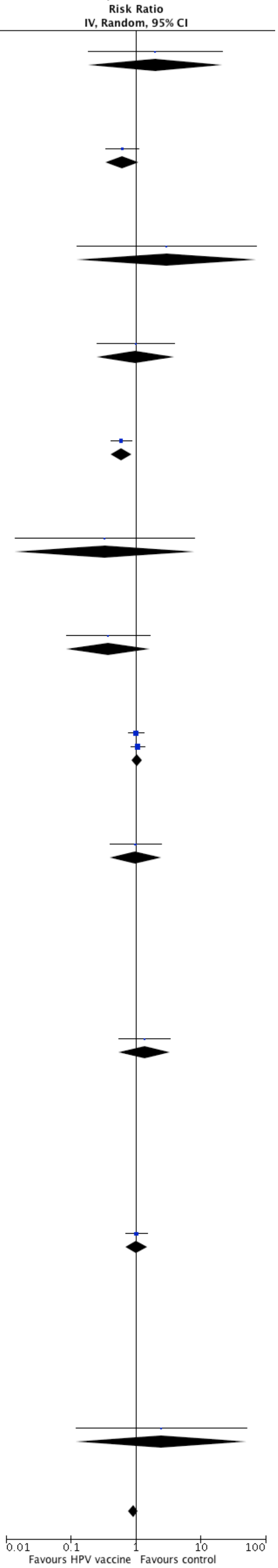

*11.11. Risk ratio for 'medically significant conditions' (GlaxoSmithKline): **0.64 [0.48, 0.86]**; risk ratio for ‘new medical history’ (Merck Sharp & Dohme): 1.04 [0.88, 1.22]; risk ratio for the follow-up periods for the trials V501-005, V501-019 and V501-020: 0.95 [0.68, 1.34]. The trials V501-005, V501-019 and V501-020 split the reporting of new onset diseases into the vaccination period and the follow-up period. To avoid double counting of participants in the total risk ratio estimate, we only included the new onset diseases reported in the vaccination period for the trials V501-005, V501-019 and V501-020.

11.12. New onset diseases ('medically significant conditions' and 'new medical history'*) reported within the MedDRA system organ class ‘infections and infestations (10021881)': intention to treat analysis

Study or Subgroup	HPV vaccine Events	HPV vaccine Total	Control Events	Control Total	Weight	Risk Ratio IV, Random, 95% CI
**11.12.1 New onset infections and infestations ('medically significant conditions'): Cervarix vs. Al(OH)3 (females, age 9-17; follow-up until 12 months)**						
HPV-058: Cervarix vs. Al(OH)3 (f, 9-17; 12)	1	374	3	376	0.0%	0.34 [0.04, 3.21]
**Subtotal (95% CI)**		**374**		**376**	**0.0%**	**0.34 [0.04, 3.21]**
Total events	1		3			
Heterogeneity: Not applicable						
Test for overall effect: Z = 0.95 (P = 0.34)						
**11.12.2 New onset infections and infestations ('medically significant conditions'): Cervarix vs. Al(OH)3 (females, age 15-25; follow-up until 7 months)**						
HPV-038: Cervarix vs. Al(OH)3 (f, 15-25; 7)	4	149	2	76	0.0%	1.02 [0.19, 5.44]
**Subtotal (95% CI)**		**149**		**76**	**0.0%**	**1.02 [0.19, 5.44]**
Total events	4		2			
Heterogeneity: Not applicable						
Test for overall effect: Z = 0.02 (P = 0.98)						
**11.12.3 New onset infections and infestations ('medically significant conditions'): Cervarix vs. Al(OH)3 (females, age 18-35; follow-up until 7 months)**						
HPV-031: Cervarix vs. Al(OH)3 (f, 18-35; 7)	4	176	2	178	0.0%	2.02 [0.38, 10.90]
HPV-035: Cervarix vs. Al(OH)3 (f, 18-35; 7)	15	150	11	150	0.1%	1.36 [0.65, 2.87]
**Subtotal (95% CI)**		**326**		**328**	**0.1%**	**1.45 [0.74, 2.87]**
Total events	19		13			
Heterogeneity: $Tau^2 = 0.00$; $Chi^2 = 0.18$, df = 1 (P = 0.67); $I^2 = 0\%$						
Test for overall effect: Z = 1.08 (P = 0.28)						
**11.12.4 New onset infections and infestations ('medically significant conditions'): Cervarix vs. Al(OH)3 (females, age 24-72; follow-up until 36 months)**						
HPV-015: Cervarix vs. Al(OH)3 (f, 24-72; 36)	381	2882	389	2871	2.5%	0.98 [0.86, 1.11]
**Subtotal (95% CI)**		**2882**		**2871**	**2.5%**	**0.98 [0.86, 1.11]**
Total events	381		389			
Heterogeneity: Not applicable						
Test for overall effect: Z = 0.37 (P = 0.71)						
**11.12.5 New onset infections and infestations ('medically significant conditions'): Cervarix vs. Aimmugen: hepatitis A vaccine (females, age 22-29; follow-up until 12 months)**						
HPV-063: Cervarix vs. Aimmugen (f, 22-29; 12)	20	375	36	377	0.2%	0.56 [0.33, 0.95]
**Subtotal (95% CI)**		**375**		**377**	**0.2%**	**0.56 [0.33, 0.95]**
Total events	20		36			
Heterogeneity: Not applicable						
Test for overall effect: Z = 2.16 (P = 0.03)						
**11.12.6 New onset infections and infestations ('medically significant conditions'): Cervarix vs. Engerix: hepatitis B vaccine (females and males, age 9-15; follow-up until 12 months)**						
HPV-030: Cervarix vs. Engerix (f, 9-15; 12)	9	247	6	246	0.0%	1.49 [0.54, 4.13]
**Subtotal (95% CI)**		**247**		**246**	**0.0%**	**1.49 [0.54, 4.13]**
Total events	9		6			
Heterogeneity: Not applicable						
Test for overall effect: Z = 0.77 (P = 0.44)						
**11.12.7 New onset infections and infestations ('medically significant conditions'): Cervarix vs. Engerix: hepatitis B vaccine (females, age 26-46; follow-up until 12 months)**						
HPV-069: Cervarix vs. Engerix (f, 26-46; 12)	1	606	3	606	0.0%	0.33 [0.03, 3.20]
**Subtotal (95% CI)**		**606**		**606**	**0.0%**	**0.33 [0.03, 3.20]**
Total events	1		3			
Heterogeneity: Not applicable						
Test for overall effect: Z = 0.95 (P = 0.34)						
**11.12.8 New onset infections and infestations ('medically significant conditions'): Cervarix vs. Havrix: hepatitis A vaccine (females, age 9-15; follow-up until 12 months)**						
HPV-013: Cervarix vs. Havrix (f, 10-14; 12)	79	1035	105	1032	0.6%	0.75 [0.57, 0.99]
HPV-033: Cervarix vs. Havrix (f, 9-15; 7)	0	160	1	161	0.0%	0.34 [0.01, 8.17]
**Subtotal (95% CI)**		**1195**		**1193**	**0.6%**	**0.75 [0.56, 0.98]**
Total events	79		106			
Heterogeneity: $Tau^2 = 0.00$; $Chi^2 = 0.24$, df = 1 (P = 0.62); $I^2 = 0\%$						
Test for overall effect: Z = 2.07 (P = 0.04)						
**11.12.9 New onset infections and infestations ('medically significant conditions'): Cervarix vs. Havrix: hepatitis A vaccine (females, age 15-25; follow-up until 48 months)**						
HPV-008: Cervarix vs. Havrix (f, 15-25; 48)	2100	9319	2149	9325	15.7%	0.98 [0.93, 1.03]
**Subtotal (95% CI)**		**9319**		**9325**	**15.7%**	**0.98 [0.93, 1.03]**
Total events	2100		2149			
Heterogeneity: Not applicable						
Test for overall effect: Z = 0.83 (P = 0.41)						
**11.12.10 New onset infections and infestations ('medically significant conditions'): Cervarix vs. Twinrix: hepatitis A and B vaccine (females, age 8-15; follow-up until 12 months)**						
HPV-029: Cervarix vs. Twinrix (f, 8-15; 12)	9	270	9	271	0.1%	1.00 [0.40, 2.49]
**Subtotal (95% CI)**		**270**		**271**	**0.1%**	**1.00 [0.40, 2.49]**
Total events	9		9			
Heterogeneity: Not applicable						
Test for overall effect: Z = 0.01 (P = 0.99)						
**11.12.11 New onset infections and infestations ('new medical history'): Gardasil vs. carrier solution (females and males, age 9-16; follow-up until 18 months)**						
V501-018: Gardasil vs. carrier solu. (f&m,9-16;18)	165	1184	75	597	0.7%	1.11 [0.86, 1.43]
**Subtotal (95% CI)**		**1184**		**597**	**0.7%**	**1.11 [0.86, 1.43]**
Total events	165		75			
Heterogeneity: Not applicable						
Test for overall effect: Z = 0.80 (P = 0.42)						
**11.12.12 New onset infections and infestations ('new medical history'): Gardasil vs. AAHS (females, age 15-26; follow-up until 45 months)**						
V501-013: Gardasil vs. AAHS (f, 16-24; 45)	1828	2723	1841	2732	31.9%	1.00 [0.96, 1.03]
V501-015: Gardasil vs. AAHS (f, 15-26; 36)	3207	6087	3302	6080	39.8%	0.97 [0.94, 1.00]
**Subtotal (95% CI)**		**8810**		**8812**	**71.7%**	**0.98 [0.96, 1.01]**
Total events	5035		5143			
Heterogeneity: $Tau^2 = 0.00$; $Chi^2 = 1.10$, df = 1 (P = 0.29); $I^2 = 9\%$						
Test for overall effect: Z = 1.39 (P = 0.16)						
**11.12.13 New onset infections and infestations ('new medical history'): Gardasil vs. AAHS (females, age 21-46; from 0-7 months)**						
V501-019: Gardasil vs. AAHS (f, 21-46; 48)	344	1911	318	1908	2.3%	1.08 [0.94, 1.24]
**Subtotal (95% CI)**		**1911**		**1908**	**2.3%**	**1.08 [0.94, 1.24]**
Total events	344		318			
Heterogeneity: Not applicable						
Test for overall effect: Z = 1.09 (P = 0.28)						
**11.12.14 New onset infections and infestations ('new medical history'): Gardasil vs. AAHS (females, age 21-46; from 7-48 months)**						
V501-019: Gardasil vs. AAHS (f, 21-46; 48)	519	1911	510	1908	0.0%	1.02 [0.92, 1.13]
**Subtotal (95% CI)**		**0**		**0**		**Not estimable**
Total events	0		0			
Heterogeneity: Not applicable						
Test for overall effect: Not applicable						
**11.12.15 New onset infections and infestations ('new medical history'): Gardasil vs. AAHS (males, age 15-27; from 0-7 months)**						
V501-020: Gardasil vs. AAHS (m, 15-27; 36)	267	2032	265	2032	1.7%	1.01 [0.86, 1.18]
**Subtotal (95% CI)**		**2032**		**2032**	**1.7%**	**1.01 [0.86, 1.18]**
Total events	267		265			
Heterogeneity: Not applicable						
Test for overall effect: Z = 0.09 (P = 0.93)						
**11.12.16 New onset infections and infestations ('new medical history'): Gardasil vs. AAHS (males, age 15-27; from 7-36 months)**						
V501-020: Gardasil vs. AAHS (m, 15-27; 36)	313	2032	341	2033	0.0%	0.92 [0.80, 1.06]
**Subtotal (95% CI)**		**0**		**0**		**Not estimable**
Total events	0		0			
Heterogeneity: Not applicable						
Test for overall effect: Not applicable						
**11.12.17 New onset infections and infestations ('new medical history'): HPV 16 vaccine vs. AAHS (females, age 16-25; from 0-7 months)**						
V501-005: HPV 16 vaccine vs. AAHS (f, 16-25; 48)	422	1204	446	1205	3.9%	0.95 [0.85, 1.05]
**Subtotal (95% CI)**		**1204**		**1205**	**3.9%**	**0.95 [0.85, 1.05]**
Total events	422		446			
Heterogeneity: Not applicable						
Test for overall effect: Z = 1.00 (P = 0.32)						
**11.12.18 New onset infections and infestations ('new medical history'): HPV 16 vaccine vs. AAHS (females, age 16-25; from 7-48 months)**						
V501-005: HPV 16 vaccine vs. AAHS (f, 16-25; 48)	442	1204	469	1205	0.0%	0.94 [0.85, 1.05]
**Subtotal (95% CI)**		**0**		**0**		**Not estimable**
Total events	0		0			
Heterogeneity: Not applicable						
Test for overall effect: Not applicable						
**11.12.19 New onset infections and infestations ('new medical history'): Gardasil 9 vs. saline placebo (females, age 12-26; follow-up until 7 months)**						
V503-006: Gardasil 9 vs. saline (f, 12-26;7)	114	618	62	306	0.6%	0.91 [0.69, 1.20]
**Subtotal (95% CI)**		**618**		**306**	**0.6%**	**0.91 [0.69, 1.20]**
Total events	114		62			
Heterogeneity: Not applicable						
Test for overall effect: Z = 0.66 (P = 0.51)						
**Total (95% CI)**		**31502**		**30529**	**100.0%**	**0.98 [0.96, 1.00]**
Total events	8970		9025			
Heterogeneity: $Tau^2 = 0.00$; $Chi^2 = 16.91$, df = 18 (P = 0.53); $I^2 = 0\%$						
Test for overall effect: Z = 1.85 (P = 0.06)						
Test for subgroup differences: $Chi^2 = 15.39$, df = 15 (P = 0.42), $I^2 = 2.5\%$						

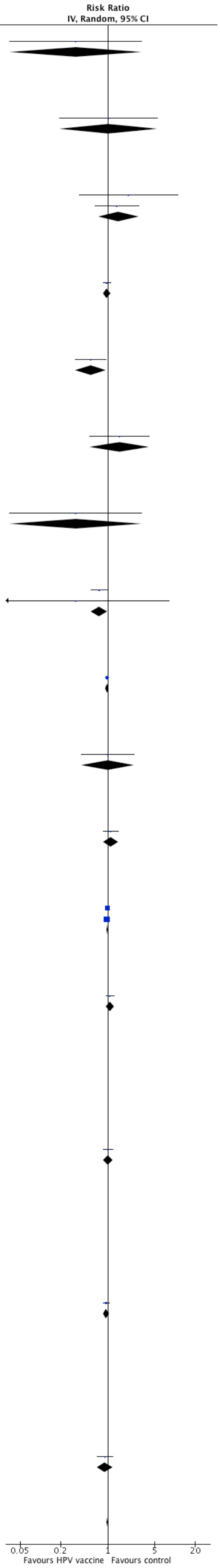

*11.12. Risk ratio for 'medically significant conditions’ (GlaxoSmithKline): 0.97 [0.80, 1.19]; risk ratio for ‘new medical history’ (Merck Sharp & Dohme): 0.98 [0.96, 1.01]; risk ratio for the follow-up periods for the trials V501-005, V501-019 and V501-020: 0.97 [0.90, 1.03]. The trials V501-005, V501-019 and V501-020 split the reporting of new onset diseases into the vaccination period and the follow-up period. To avoid double counting of participants in the total risk ratio estimate, we only included the new onset diseases reported in the vaccination fperiod for the trials V501-005, V501-019 and V501-020.

11.13. New onset diseases ('medically significant conditions' and 'new medical history'*) reported within the MedDRA system organ class ‘injury poisoning and procedural complications (10022117)': intention to treat analysis

Study or Subgroup	HPV vaccine Events	HPV vaccine Total	Control Events	Control Total	Weight	Risk Ratio IV, Random, 95% CI
**11.13.1 New onset injury, poisoning and procedural complications ('medically significant conditions'): Cervarix vs. Al(OH)3 (females, age 9-17; follow-up until 12 months)**						
HPV-058: Cervarix vs. Al(OH)3 (f, 9-17; 12)	0	374	2	376	0.2%	0.20 [0.01, 4.17]
**Subtotal (95% CI)**		**374**		**376**	**0.2%**	**0.20 [0.01, 4.17]**
Total events	0		2			
Heterogeneity: Not applicable						
Test for overall effect: Z = 1.04 (P = 0.30)						
**11.13.2 New onset injury, poisoning and procedural complications ('medically significant conditions'): Cervarix vs. Al(OH)3 (females, age 15-25; follow-up until 7 months)**						
HPV-038: Cervarix vs. Al(OH)3 (f, 15-25; 7)	5	149	0	76	0.2%	5.65 [0.32, 100.79]
**Subtotal (95% CI)**		**149**		**76**	**0.2%**	**5.65 [0.32, 100.79]**
Total events	5		0			
Heterogeneity: Not applicable						
Test for overall effect: Z = 1.18 (P = 0.24)						
**11.13.3 New onset injury, poisoning and procedural complications ('medically significant conditions'): Cervarix vs. Al(OH)3 (females, age 18-35; follow-up until 7 months)**						
HPV-031: Cervarix vs. Al(OH)3 (f, 18-35; 7)	1	176	4	178	0.3%	0.25 [0.03, 2.24]
HPV-035: Cervarix vs. Al(OH)3 (f, 18-35; 7)	1	150	2	150	0.3%	0.50 [0.05, 5.46]
**Subtotal (95% CI)**		**326**		**328**	**0.6%**	**0.34 [0.07, 1.73]**
Total events	2		6			
Heterogeneity: $Tau^2 = 0.00$; $Chi^2 = 0.17$, df = 1 (P = 0.68); $I^2 = 0\%$						
Test for overall effect: Z = 1.30 (P = 0.20)						
**11.13.4 New onset injury, poisoning and procedural complications ('medically significant conditions'): Cervarix vs. Al(OH)3 (females, age 24-72; follow-up until 36 months)**						
HPV-015: Cervarix vs. Al(OH)3 (f, 24-72; 36)	139	2882	154	2871	12.8%	0.90 [0.72, 1.12]
**Subtotal (95% CI)**		**2882**		**2871**	**12.8%**	**0.90 [0.72, 1.12]**
Total events	139		154			
Heterogeneity: Not applicable						
Test for overall effect: Z = 0.93 (P = 0.35)						
**11.13.5 New onset injury, poisoning and procedural complications ('medically significant conditions'): Cervarix vs. Aimmugen: hepatitis A vaccine (females, age 22-29; follow-up until 12 months)**						
HPV-063: Cervarix vs. Aimmugen (f, 22-29; 12)	11	375	14	377	2.4%	0.79 [0.36, 1.72]
**Subtotal (95% CI)**		**375**		**377**	**2.4%**	**0.79 [0.36, 1.72]**
Total events	11		14			
Heterogeneity: Not applicable						
Test for overall effect: Z = 0.60 (P = 0.55)						
**11.13.6 New onset injury, poisoning and procedural complications ('medically significant conditions'): Cervarix vs. Engerix: hepatitis B vaccine (females and males, age 9-15; follow-up until 12 months)**						
HPV-030: Cervarix vs. Engerix (f, 9-15; 12)	10	247	1	246	0.4%	9.96 [1.28, 77.21]
**Subtotal (95% CI)**		**247**		**246**	**0.4%**	**9.96 [1.28, 77.21]**
Total events	10		1			
Heterogeneity: Not applicable						
Test for overall effect: Z = 2.20 (P = 0.03)						
**11.13.7 New onset injury, poisoning and procedural complications ('medically significant conditions'): Cervarix vs. Havrix: hepatitis A vaccine (females, age 9-15; follow-up until 12 months)**						
HPV-013: Cervarix vs. Havrix (f, 10-14; 12)	15	1035	22	1032	3.3%	0.68 [0.35, 1.30]
HPV-033: Cervarix vs. Havrix (f, 9-15; 7)	1	160	2	161	0.3%	0.50 [0.05, 5.49]
**Subtotal (95% CI)**		**1195**		**1193**	**3.6%**	**0.67 [0.36, 1.25]**
Total events	16		24			
Heterogeneity: $Tau^2 = 0.00$; $Chi^2 = 0.06$, df = 1 (P = 0.81); $I^2 = 0\%$						
Test for overall effect: Z = 1.27 (P = 0.20)						
**11.13.8 New onset injury, poisoning and procedural complications ('medically significant conditions'): Cervarix vs. Havrix: hepatitis A vaccine (females, age 15-25; follow-up until 48 months)**						
HPV-008: Cervarix vs. Havrix (f, 15-25; 48)	245	9319	275	9325	15.3%	0.89 [0.75, 1.06]
**Subtotal (95% CI)**		**9319**		**9325**	**15.3%**	**0.89 [0.75, 1.06]**
Total events	245		275			
Heterogeneity: Not applicable						
Test for overall effect: Z = 1.33 (P = 0.18)						
**11.13.9 New onset injury, poisoning and procedural complications ('medically significant conditions'): Cervarix vs. Twinrix: hepatitis A and B vaccine (females, age 8-15; follow-up until 12 months)**						
HPV-029: Cervarix vs. Twinrix (f, 8-15; 12)	6	270	7	271	1.3%	0.86 [0.29, 2.53]
**Subtotal (95% CI)**		**270**		**271**	**1.3%**	**0.86 [0.29, 2.53]**
Total events	6		7			
Heterogeneity: Not applicable						
Test for overall effect: Z = 0.27 (P = 0.78)						
**11.13.10 New onset injury, poisoning and procedural complications ('new medical history'): Gardasil vs. carrier solution (females and males, age 9-16; follow-up until 18 months)**						
V501-018: Gardasil vs. carrier solu. (f&m,9-16;18)	65	1184	47	597	7.8%	0.70 [0.49, 1.00]
**Subtotal (95% CI)**		**1184**		**597**	**7.8%**	**0.70 [0.49, 1.00]**
Total events	65		47			
Heterogeneity: Not applicable						
Test for overall effect: Z = 1.95 (P = 0.05)						
**11.13.11 New onset injury, poisoning and procedural complications ('new medical history'): Gardasil vs. AAHS (females, age 15-26; follow-up until 45 months)**						
V501-013: Gardasil vs. AAHS (f, 16-24; 45)	222	2723	204	2732	14.7%	1.09 [0.91, 1.31]
V501-015: Gardasil vs. AAHS (f, 15-26; 36)	304	6087	297	6080	16.0%	1.02 [0.87, 1.19]
**Subtotal (95% CI)**		**8810**		**8812**	**30.7%**	**1.05 [0.93, 1.18]**
Total events	526		501			
Heterogeneity: $Tau^2 = 0.00$; $Chi^2 = 0.29$, df = 1 (P = 0.59); $I^2 = 0\%$						
Test for overall effect: Z = 0.82 (P = 0.41)						
**11.13.12 New onset injury, poisoning and procedural complications ('new medical history'): Gardasil vs. AAHS (females, age 21-46; from 0-7 months)**						
V501-019: Gardasil vs. AAHS (f, 21-46; 48)	62	1911	40	1908	7.1%	1.55 [1.05, 2.29]
**Subtotal (95% CI)**		**1911**		**1908**	**7.1%**	**1.55 [1.05, 2.29]**
Total events	62		40			
Heterogeneity: Not applicable						
Test for overall effect: Z = 2.18 (P = 0.03)						
**11.13.13 New onset injury, poisoning and procedural complications ('new medical history'): Gardasil vs. AAHS (females, age 21-46; from 7-48 months)**						
V501-019: Gardasil vs. AAHS (f, 21-46; 48)	130	1911	96	1908	0.0%	1.35 [1.05, 1.75]
**Subtotal (95% CI)**		**0**		**0**		**Not estimable**
Total events	0		0			
Heterogeneity: Not applicable						
Test for overall effect: Not applicable						
**11.13.14 New onset injury, poisoning and procedural complications ('new medical history'): Gardasil vs. AAHS (males, age 15-27; from 0-7 months)**						
V501-020: Gardasil vs. AAHS (m, 15-27; 36)	48	2032	45	2032	6.8%	1.07 [0.71, 1.59]
**Subtotal (95% CI)**		**2032**		**2032**	**6.8%**	**1.07 [0.71, 1.59]**
Total events	48		45			
Heterogeneity: Not applicable						
Test for overall effect: Z = 0.31 (P = 0.75)						
**11.13.15 New onset injury, poisoning and procedural complications ('new medical history'): Gardasil vs. AAHS (males, age 15-27; from 7-36 months)**						
V501-020: Gardasil vs. AAHS (m, 15-27; 36)	95	2032	83	2033	0.0%	1.15 [0.86, 1.53]
**Subtotal (95% CI)**		**0**		**0**		**Not estimable**
Total events	0		0			
Heterogeneity: Not applicable						
Test for overall effect: Not applicable						
**11.13.16 New onset injury, poisoning and procedural complications ('new medical history'): HPV 16 vaccine vs. AAHS (females, age 16-25; from 0-7 months)**						
V501-005: HPV 16 vaccine vs. AAHS (f, 16-25; 48)	60	1204	86	1205	9.1%	0.70 [0.51, 0.96]
**Subtotal (95% CI)**		**1204**		**1205**	**9.1%**	**0.70 [0.51, 0.96]**
Total events	60		86			
Heterogeneity: Not applicable						
Test for overall effect: Z = 2.20 (P = 0.03)						
**11.13.17 New onset injury, poisoning and procedural complications ('new medical history'): HPV 16 vaccine vs. AAHS (females, age 16-25; from 7-48 months)**						
V501-005: HPV 16 vaccine vs. AAHS (f, 16-25; 48)	93	1204	93	1205	0.0%	1.00 [0.76, 1.32]
**Subtotal (95% CI)**		**0**		**0**		**Not estimable**
Total events	0		0			
Heterogeneity: Not applicable						
Test for overall effect: Not applicable						
**11.13.18 New onset injury, poisoning and procedural complications ('new medical history'): Gardasil 9 vs. saline placebo (females, age 12-26; follow-up until 7 months)**						
V503-006: Gardasil 9 vs. saline (f, 12-26;7)	10	618	7	306	1.7%	0.71 [0.27, 1.84]
**Subtotal (95% CI)**		**618**		**306**	**1.7%**	**0.71 [0.27, 1.84]**
Total events	10		7			
Heterogeneity: Not applicable						
Test for overall effect: Z = 0.71 (P = 0.48)						
**Total (95% CI)**		**30896**		**29923**	**100.0%**	**0.94 [0.82, 1.06]**
Total events	1205		1209			
Heterogeneity: $Tau^2 = 0.02$; $Chi^2 = 27.58$, df = 17 (P = 0.05); $I^2 = 38\%$						
Test for overall effect: Z = 1.00 (P = 0.32)						
Test for subgroup differences: $Chi^2 = 27.06$, df = 14 (P = 0.02), $I^2 = 48.3\%$						

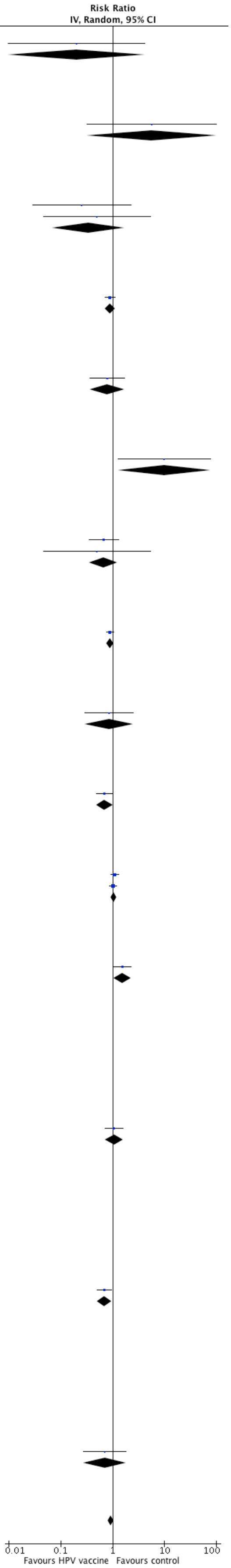

*11.13. Risk ratio for 'medically significant conditions’ (GlaxoSmithKline): 0.88 [0.76, 1.02]; risk ratio for ‘new medical history’ (Merck Sharp & Dohme): 0.97 [0.81, 1.17]; risk ratio for the follow-up periods for the trials V501-005, V501-019 and V501-020: 1.17 [0.98, 1.39]. The trials V501-005, V501-019 and V501-020 split the reporting of new onset diseases into the vaccination period and the follow-up period. To avoid double counting of participants in the total risk ratio estimate, we only included the new onset diseases reported in the vaccination period for the trials V501-005, V501-019 and V501-020.

11.14. New onset diseases ('medically significant conditions' and 'new medical history'*) reported within the MedDRA system organ class 'investigations (10022891)': intention to treat analysis

Study or Subgroup	HPV vaccine Events	HPV vaccine Total	Control Events	Control Total	Weight	Risk Ratio IV, Random, 95% CI
**11.14.1 New onset investigations ('medically significant conditions'): Cervarix vs. Al(OH)3 (females, age 24-72; follow-up until 36 months)**						
HPV-015: Cervarix vs. Al(OH)3 (f, 24-72; 36)	11	2882	19	2891	0.8%	0.58 [0.28, 1.22]
**Subtotal (95% CI)**		**2882**		**2891**	**0.8%**	**0.58 [0.28, 1.22]**
Total events	11		19			
Heterogeneity: Not applicable						
Test for overall effect: Z = 1.44 (P = 0.15)						
**11.14.2 New onset investigations ('medically significant conditions'): Cervarix vs. Aimmugen: hepatitis A vaccine (females, age 22-29; follow-up until 12 months)**						
HPV-063: Cervarix vs. Aimmugen (f, 22-29; 12)	1	375	0	377	0.0%	3.02 [0.12, 73.80]
**Subtotal (95% CI)**		**375**		**377**	**0.0%**	**3.02 [0.12, 73.80]**
Total events	1		0			
Heterogeneity: Not applicable						
Test for overall effect: Z = 0.68 (P = 0.50)						
**11.14.3 New onset investigations ('medically significant conditions'): Cervarix vs. Havrix: hepatitis A vaccine (females, age 10-14; follow-up until 12 months)**						
HPV-013: Cervarix vs. Havrix (f, 10-14; 12)	0	1035	2	1032	0.1%	0.20 [0.01, 4.15]
**Subtotal (95% CI)**		**1035**		**1032**	**0.1%**	**0.20 [0.01, 4.15]**
Total events	0		2			
Heterogeneity: Not applicable						
Test for overall effect: Z = 1.04 (P = 0.30)						
**11.14.4 New onset investigations ('medically significant conditions'): Cervarix vs. Havrix: hepatitis A vaccine (females, age 15-25; follow-up until 48 months)**						
HPV-008: Cervarix vs. Havrix (f, 15-25; 48)	14	9319	14	9325	0.8%	1.00 [0.48, 2.10]
**Subtotal (95% CI)**		**9319**		**9325**	**0.8%**	**1.00 [0.48, 2.10]**
Total events	14		14			
Heterogeneity: Not applicable						
Test for overall effect: Z = 0.00 (P = 1.00)						
**11.14.5 New onset investigations ('new medical history'): Gardasil vs. carrier solution (females and males, age 9-16; follow-up until 18 months)**						
V501-018: Gardasil vs. carrier solu. (f&m,9-16;18)	2	1184	4	597	0.2%	0.25 [0.05, 1.37]
**Subtotal (95% CI)**		**1184**		**597**	**0.2%**	**0.25 [0.05, 1.37]**
Total events	2		4			
Heterogeneity: Not applicable						
Test for overall effect: Z = 1.59 (P = 0.11)						
**11.14.6 New onset investigations ('new medical history'): Gardasil vs. AAHS (females, age 15-26; follow-up until 45 months)**						
V501-013: Gardasil vs. AAHS (f, 16-24; 45)	457	2723	461	2732	33.3%	0.99 [0.88, 1.12]
V501-015: Gardasil vs. AAHS (f, 15-26; 36)	820	6087	815	6080	57.1%	1.00 [0.92, 1.10]
**Subtotal (95% CI)**		**8810**		**8812**	**90.4%**	**1.00 [0.93, 1.08]**
Total events	1277		1276			
Heterogeneity: Tau² = 0.00; Chi² = 0.02, df = 1 (P = 0.89); I² = 0%						
Test for overall effect: Z = 0.03 (P = 0.98)						
**11.14.7 New onset investigations ('new medical history'): Gardasil vs. AAHS (females, age 21-46; from 0-7 months)**						
V501-019: Gardasil vs. AAHS (f, 21-46; 48)	43	1911	34	1908	2.3%	1.26 [0.81, 1.97]
**Subtotal (95% CI)**		**1911**		**1908**	**2.3%**	**1.26 [0.81, 1.97]**
Total events	43		34			
Heterogeneity: Not applicable						
Test for overall effect: Z = 1.03 (P = 0.30)						
**11.14.8 New onset investigations ('new medical history'): Gardasil vs. AAHS (females, age 21-46; from 7-48 months)**						
V501-019: Gardasil vs. AAHS (f, 21-46; 48)	150	1911	156	1908	0.0%	0.96 [0.77, 1.19]
**Subtotal (95% CI)**		**0**		**0**		**Not estimable**
Total events	0		0			
Heterogeneity: Not applicable						
Test for overall effect: Not applicable						
**11.14.9 New onset investigations ('new medical history'): Gardasil vs. AAHS (males, age 15-27; from 0-7 months)**						
V501-020: Gardasil vs. AAHS (m, 15-27; 36)	6	2032	3	2032	0.2%	2.00 [0.50, 7.99]
**Subtotal (95% CI)**		**2032**		**2032**	**0.2%**	**2.00 [0.50, 7.99]**
Total events	6		3			
Heterogeneity: Not applicable						
Test for overall effect: Z = 0.98 (P = 0.33)						
**11.14.10 New onset investigations ('new medical history'): Gardasil vs. AAHS (males, age 15-27; from 7-36 months)**						
V501-020: Gardasil vs. AAHS (m, 15-27; 36)	28	2032	29	2033	0.0%	0.97 [0.58, 1.62]
**Subtotal (95% CI)**		**0**		**0**		**Not estimable**
Total events	0		0			
Heterogeneity: Not applicable						
Test for overall effect: Not applicable						
**11.14.11 New onset investigations ('new medical history'): HPV 16 vaccine vs. AAHS (females, age 16-25; from 0-7 months)**						
V501-005: HPV 16 vaccine vs. AAHS (f, 16-25; 48)	75	1204	74	1205	4.8%	1.01 [0.74, 1.38]
**Subtotal (95% CI)**		**1204**		**1205**	**4.8%**	**1.01 [0.74, 1.38]**
Total events	75		74			
Heterogeneity: Not applicable						
Test for overall effect: Z = 0.09 (P = 0.93)						
**11.14.12 New onset investigations ('new medical history'): HPV 16 vaccine vs. AAHS (females, age 16-25; from 7-48 months)**						
V501-005: HPV 16 vaccine vs. AAHS (f, 16-25; 48)	217	1204	234	1205	0.0%	0.93 [0.79, 1.10]
**Subtotal (95% CI)**		**0**		**0**		**Not estimable**
Total events	0		0			
Heterogeneity: Not applicable						
Test for overall effect: Not applicable						
**11.14.13 New onset investigations ('new medical history'): Gardasil 9 vs. saline placebo (females, age 12-26; follow-up until 7 months)**						
V503-006: Gardasil 9 vs. saline (f, 12-26;7)	9	618	3	306	0.3%	1.49 [0.41, 5.45]
**Subtotal (95% CI)**		**618**		**306**	**0.3%**	**1.49 [0.41, 5.45]**
Total events	9		3			
Heterogeneity: Not applicable						
Test for overall effect: Z = 0.60 (P = 0.55)						
**Total (95% CI)**		**29370**		**28485**	**100.0%**	**1.00 [0.94, 1.07]**
Total events	1438		1429			
Heterogeneity: Tau² = 0.00; Chi² = 8.54, df = 10 (P = 0.58); I² = 0%						
Test for overall effect: Z = 0.08 (P = 0.94)						
Test for subgroup differences: Chi² = 8.52, df = 9 (P = 0.48), I² = 0%						

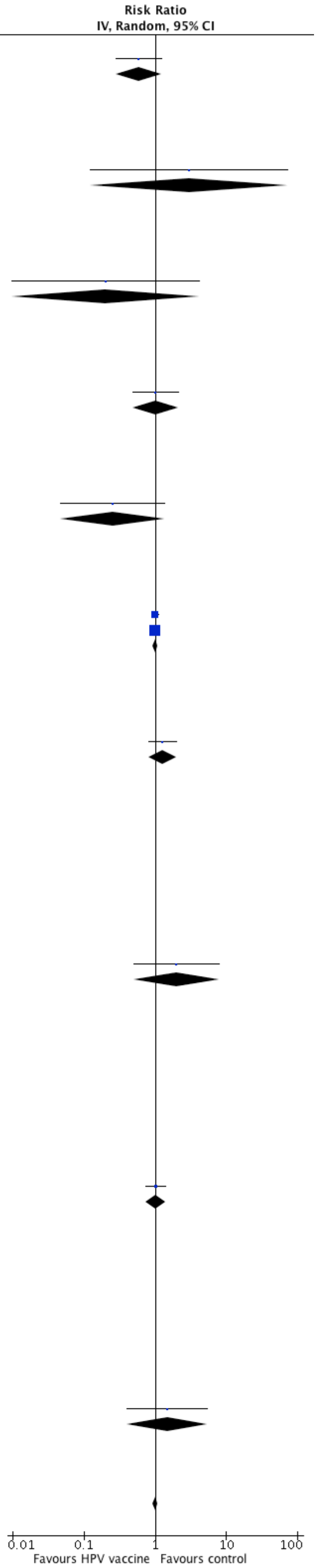

*11.14. Risk ratio for 'medically significant conditions' (GlaxoSmithKline): 0.76 [0.46, 1.27]; risk ratio for 'new medical history' (Merck Sharp & Dohme): 1.01 [0.94, 1.08]; risk ratio for the follow-up periods for the trials V501-005, V501-019 and V501-020: 0.94 [0.83, 1.07]. The trials V501-005, V501-019 and V501-020 split the reporting of new onset diseases into the vaccination period and the follow-up period. To avoid double counting of participants in the total risk ratio estimate, we only included the new onset diseases reported in the vaccination period for the trials V501-005, V501-019 and V501-020.

11.15. New onset diseases ('medically significant conditions' and 'new medical history'*) reported within the MedDRA system organ class ‘metabolism and nutrition disorders (10027433)': intention to treat analysis

Study or Subgroup	HPV vaccine Events	HPV vaccine Total	Control Events	Control Total	Weight	Risk Ratio IV, Random, 95% CI
**11.15.1 New onset metabolism and nutrition disorders ('medically significant conditions'): Cervarix vs. Al(OH)3 (females, age 24-72; follow-up until 36 months)**						
HPV-015: Cervarix vs. Al(OH)3 (f, 24-72; 36)	101	2882	137	2871	34.6%	0.73 [0.57, 0.94]
**Subtotal (95% CI)**		**2882**		**2871**	**34.6%**	**0.73 [0.57, 0.94]**
Total events	101		137			
Heterogeneity: Not applicable						
Test for overall effect: Z = 2.40 (P = 0.02)						
**11.15.2 New onset metabolism and nutrition disorders ('medically significant conditions'): Cervarix vs. Havrix: hepatitis A vaccine (females, age 10-14; follow-up until 12 months)**						
HPV-013: Cervarix vs. Havrix (f, 10-14; 12)	3	1035	2	1032	0.7%	1.50 [0.25, 8.93]
**Subtotal (95% CI)**		**1035**		**1032**	**0.7%**	**1.50 [0.25, 8.93]**
Total events	3		2			
Heterogeneity: Not applicable						
Test for overall effect: Z = 0.44 (P = 0.66)						
**11.15.3 New onset metabolism and nutrition disorders ('medically significant conditions'): Cervarix vs. Havrix: hepatitis A vaccine (females, age 15-25; follow-up until 48 months)**						
HPV-008: Cervarix vs. Havrix (f, 15-25; 48)	37	9319	41	9325	11.2%	0.90 [0.58, 1.41]
**Subtotal (95% CI)**		**9319**		**9325**	**11.2%**	**0.90 [0.58, 1.41]**
Total events	37		41			
Heterogeneity: Not applicable						
Test for overall effect: Z = 0.45 (P = 0.65)						
**11.15.4 New onset metabolism and nutrition disorders ('new medical history'): Gardasil vs. carrier solution (females and males, age 9-16; follow-up until 18 months)**						
V501-018: Gardasil vs. carrier solu. (f&m,9-16;18)	9	1184	2	597	0.9%	2.27 [0.49, 10.47]
**Subtotal (95% CI)**		**1184**		**597**	**0.9%**	**2.27 [0.49, 10.47]**
Total events	9		2			
Heterogeneity: Not applicable						
Test for overall effect: Z = 1.05 (P = 0.29)						
**11.15.5 New onset metabolism and nutrition disorders ('new medical history'): Gardasil vs. AAHS (females, age 15-26; follow-up until 45 months)**						
V501-013: Gardasil vs. AAHS (f, 16-24; 45)	63	2723	53	2732	16.8%	1.19 [0.83, 1.71]
V501-015: Gardasil vs. AAHS (f, 15-26; 36)	96	6087	91	6080	27.1%	1.05 [0.79, 1.40]
**Subtotal (95% CI)**		**8810**		**8812**	**43.9%**	**1.10 [0.88, 1.38]**
Total events	159		144			
Heterogeneity: Tau² = 0.00; Chi² = 0.28, df = 1 (P = 0.60); I² = 0%						
Test for overall effect: Z = 0.87 (P = 0.38)						
**11.15.6 New onset metabolism and nutrition disorders ('new medical history'): Gardasil vs. AAHS (females, age 21-46; from 0-7 months)**						
V501-019: Gardasil vs. AAHS (f, 21-46; 48)	15	1911	11	1908	3.6%	1.36 [0.63, 2.96]
**Subtotal (95% CI)**		**1911**		**1908**	**3.6%**	**1.36 [0.63, 2.96]**
Total events	15		11			
Heterogeneity: Not applicable						
Test for overall effect: Z = 0.78 (P = 0.44)						
**11.15.7 New onset metabolism and nutrition disorders ('new medical history'): Gardasil vs. AAHS (females, age 21-46; from 7-48 months)**						
V501-019: Gardasil vs. AAHS (f, 21-46; 48)	87	1911	75	1908	0.0%	1.16 [0.86, 1.57]
**Subtotal (95% CI)**		**0**		**0**		**Not estimable**
Total events	0		0			
Heterogeneity: Not applicable						
Test for overall effect: Not applicable						
**11.15.8 New onset metabolism and nutrition disorders ('new medical history'): Gardasil vs. AAHS (males, age 15-27; from 0-7 months)**						
V501-020: Gardasil vs. AAHS (m, 15-27; 36)	1	2032	2	2032	0.4%	0.50 [0.05, 5.51]
**Subtotal (95% CI)**		**2032**		**2032**	**0.4%**	**0.50 [0.05, 5.51]**
Total events	1		2			
Heterogeneity: Not applicable						
Test for overall effect: Z = 0.57 (P = 0.57)						
**11.15.9 New onset metabolism and nutrition disorders ('new medical history'): Gardasil vs. AAHS (males, age 15-27; from 7-36 months)**						
V501-020: Gardasil vs. AAHS (m, 15-27; 36)	9	2032	12	2033	0.0%	0.75 [0.32, 1.78]
**Subtotal (95% CI)**		**0**		**0**		**Not estimable**
Total events	0		0			
Heterogeneity: Not applicable						
Test for overall effect: Not applicable						
**11.15.10 New onset metabolism and nutrition disorders ('new medical history'): HPV 16 vaccine vs. AAHS (females, age 16-25; from 7-48 months)**						
V501-005: HPV 16 vaccine vs. AAHS (f, 16-25; 48)	15	1204	15	1205	4.3%	1.00 [0.49, 2.04]
**Subtotal (95% CI)**		**1204**		**1205**	**4.3%**	**1.00 [0.49, 2.04]**
Total events	15		15			
Heterogeneity: Not applicable						
Test for overall effect: Z = 0.00 (P = 1.00)						
**11.15.11 New onset metabolism and nutrition disorders ('new medical history'): Gardasil 9 vs. saline placebo (females, age 12-26; follow-up until 7 months)**						
V503-006: Gardasil 9 vs. saline (f, 12-26;7)	2	618	1	306	0.4%	0.99 [0.09, 10.88]
**Subtotal (95% CI)**		**618**		**306**	**0.4%**	**0.99 [0.09, 10.88]**
Total events	2		1			
Heterogeneity: Not applicable						
Test for overall effect: Z = 0.01 (P = 0.99)						
**Total (95% CI)**		**28995**		**28088**	**100.0%**	**0.95 [0.82, 1.10]**
Total events	342		355			
Heterogeneity: Tau² = 0.00; Chi² = 8.71, df = 9 (P = 0.46); I² = 0%						
Test for overall effect: Z = 0.73 (P = 0.46)						
Test for subgroup differences: Chi² = 8.43, df = 8 (P = 0.39), I² = 5.1%						

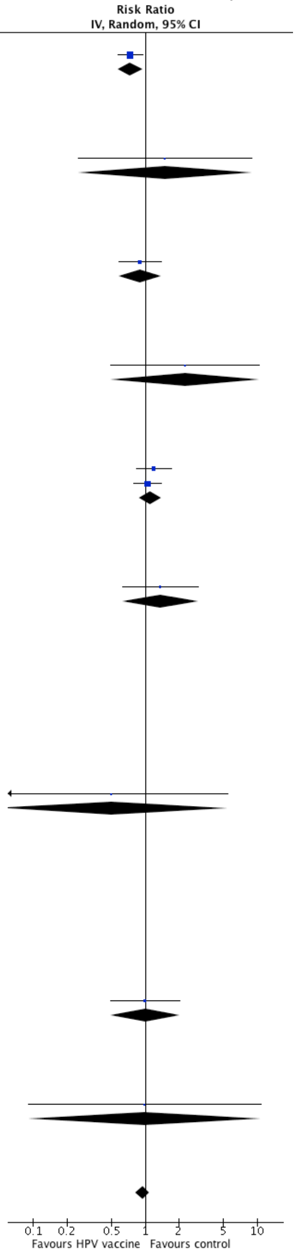

*11.15. Risk ratio for 'medically significant conditions’ (GlaxoSmithKline): **0.78 [0.63, 0.97]**; risk ratio for ‘new medical history’ (Merck Sharp & Dohme): 1.12 [0.91, 1.37]; risk ratio for the follow-up periods for the trials V501-019 and V501-020: 1.10 [0.83, 1.47]. The trials V501-019 and V501-020 split the reporting of new onset diseases into the vaccination period and the follow-up period. To avoid double counting of participants in the total risk ratio estimate, we only included the new onset diseases reported in the vaccination period for the trials V501-019 and V501-020.

11.16. New onset diseases ('medically significant conditions' and 'new medical history'*) reported within the MedDRA system organ class ‘musculoskeletal and connective tissue disorders (10028395)': intention to treat analysis

Study or Subgroup	HPV vaccine Events	HPV vaccine Total	Control Events	Control Total	Weight	Risk Ratio IV, Random, 95% CI
**11.16.1 New onset musculoskeletal and connective tissue disorders ('medically significant conditions'): Cervarix vs. Al(OH)3 (females, age 9-17; follow-up until 12 months)**						
HPV-058: Cervarix vs. Al(OH)3 (f, 9-17; 12)	1	374	0	376	0.1%	3.02 [0.12, 73.80]
**Subtotal (95% CI)**		**374**		**376**	**0.1%**	**3.02 [0.12, 73.80]**
Total events	1		0			
Heterogeneity: Not applicable						
Test for overall effect: Z = 0.68 (P = 0.50)						
**11.16.2 New onset musculoskeletal and connective tissue disorders ('medically significant conditions'): Cervarix vs. Al(OH)3 (females, age 15-25; follow-up until 7 months)**						
HPV-038: Cervarix vs. Al(OH)3 (f, 15-25; 7)	3	149	0	76	0.1%	3.59 [0.19, 68.68]
**Subtotal (95% CI)**		**149**		**76**	**0.1%**	**3.59 [0.19, 68.68]**
Total events	3		0			
Heterogeneity: Not applicable						
Test for overall effect: Z = 0.85 (P = 0.40)						
**11.16.3 New onset musculoskeletal and connective tissue disorders ('medically significant conditions'): Cervarix vs. Al(OH)3 (females, age 18-35; follow-up until 7 months)**						
HPV-031: Cervarix vs. Al(OH)3 (f, 18-35; 7)	5	176	2	178	0.2%	2.53 [0.50, 12.86]
HPV-035: Cervarix vs. Al(OH)3 (f, 18-35; 7)	4	150	1	150	0.1%	4.00 [0.45, 35.37]
**Subtotal (95% CI)**		**326**		**328**	**0.3%**	**2.98 [0.81, 10.97]**
Total events	9		3			
Heterogeneity: Tau² = 0.00; Chi² = 0.11, df = 1 (P = 0.74); I² = 0%						
Test for overall effect: Z = 1.64 (P = 0.10)						
**11.16.4 New onset musculoskeletal and connective tissue disorders ('medically significant conditions'): Cervarix vs. Al(OH)3 (females, age 24-72; follow-up until 36 months)**						
HPV-015: Cervarix vs. Al(OH)3 (f, 24-72; 36)	200	2882	213	2871	17.1%	0.94 [0.78, 1.13]
**Subtotal (95% CI)**		**2882**		**2871**	**17.1%**	**0.94 [0.78, 1.13]**
Total events	200		213			
Heterogeneity: Not applicable						
Test for overall effect: Z = 0.70 (P = 0.48)						
**11.16.5 New onset musculoskeletal and connective tissue disorders ('medically significant conditions'): Cervarix vs. Aimmugen: hepatitis A vaccine (females, age 22-29; follow-up until 12 months)**						
HPV-063: Cervarix vs. Aimmugen (f, 22-29; 12)	3	375	4	377	0.3%	0.75 [0.17, 3.35]
**Subtotal (95% CI)**		**375**		**377**	**0.3%**	**0.75 [0.17, 3.35]**
Total events	3		4			
Heterogeneity: Not applicable						
Test for overall effect: Z = 0.37 (P = 0.71)						
**11.16.6 New onset musculoskeletal and connective tissue disorders ('medically significant conditions'): Cervarix vs. Engerix: hepatitis B vaccine (females and males, age 9-15; follow-up until 12 months)**						
HPV-030: Cervarix vs. Engerix (f, 9-15; 12)	2	247	2	246	0.2%	1.00 [0.14, 7.01]
**Subtotal (95% CI)**		**247**		**246**	**0.2%**	**1.00 [0.14, 7.01]**
Total events	2		2			
Heterogeneity: Not applicable						
Test for overall effect: Z = 0.00 (P = 1.00)						
**11.16.7 New onset musculoskeletal and connective tissue disorders ('medically significant conditions'): Cervarix vs. Havrix: hepatitis A vaccine (females, age 10-14; follow-up until 12 months)**						
HPV-013: Cervarix vs. Havrix (f, 10-14; 12)	16	1035	6	1032	0.7%	2.66 [1.04, 6.77]
**Subtotal (95% CI)**		**1035**		**1032**	**0.7%**	**2.66 [1.04, 6.77]**
Total events	16		6			
Heterogeneity: Not applicable						
Test for overall effect: Z = 2.05 (P = 0.04)						
**11.16.8 New onset musculoskeletal and connective tissue disorders ('medically significant conditions'): Cervarix vs. Havrix: hepatitis A vaccine (females, age 15-25; follow-up until 48 months)**						
HPV-008: Cervarix vs. Havrix (f, 15-25; 48)	210	9319	200	9325	16.1%	1.05 [0.87, 1.27]
**Subtotal (95% CI)**		**9319**		**9325**	**16.1%**	**1.05 [0.87, 1.27]**
Total events	210		200			
Heterogeneity: Not applicable						
Test for overall effect: Z = 0.51 (P = 0.61)						
**11.16.9 New onset musculoskeletal and connective tissue disorders ('medically significant conditions'): Cervarix vs. Twinrix: hepatitis A and B vaccine (females, age 8-15; follow-up until 12 months)**						
HPV-029: Cervarix vs. Twinrix (f, 8-15; 12)	1	270	0	271	0.1%	3.01 [0.12, 73.59]
**Subtotal (95% CI)**		**270**		**271**	**0.1%**	**3.01 [0.12, 73.59]**
Total events	1		0			
Heterogeneity: Not applicable						
Test for overall effect: Z = 0.68 (P = 0.50)						
**11.16.10 New onset musculoskeletal and connective tissue disorders ('new medical history'): Gardasil vs. carrier solution (females and males, age 9-16; follow-up until 18 months)**						
V501-018: Gardasil vs. carrier solu. (f&m,9-16;18)	31	1184	24	597	2.2%	0.65 [0.39, 1.10]
**Subtotal (95% CI)**		**1184**		**597**	**2.2%**	**0.65 [0.39, 1.10]**
Total events	31		24			
Heterogeneity: Not applicable						
Test for overall effect: Z = 1.60 (P = 0.11)						
**11.16.11 New onset musculoskeletal and connective tissue disorders ('new medical history'): Gardasil vs. AAHS (females, age 15-26; follow-up until 45 months)**						
V501-013: Gardasil vs. AAHS (f, 16-24; 45)	188	2723	185	2732	15.4%	1.02 [0.84, 1.24]
V501-015: Gardasil vs. AAHS (f, 15-26; 36)	362	6087	351	6080	29.1%	1.03 [0.89, 1.19]
**Subtotal (95% CI)**		**8810**		**8812**	**44.5%**	**1.03 [0.91, 1.15]**
Total events	550		536			
Heterogeneity: Tau² = 0.00; Chi² = 0.01, df = 1 (P = 0.93); I² = 0%						
Test for overall effect: Z = 0.44 (P = 0.66)						
**11.16.12 New onset musculoskeletal and connective tissue disorders ('new medical history'): Gardasil vs. AAHS (females, age 21-46; from 7-48 months)**						
V501-019: Gardasil vs. AAHS (f, 21-46; 48)	136	1911	128	1908	10.9%	1.06 [0.84, 1.34]
**Subtotal (95% CI)**		**1911**		**1908**	**10.9%**	**1.06 [0.84, 1.34]**
Total events	136		128			
Heterogeneity: Not applicable						
Test for overall effect: Z = 0.50 (P = 0.62)						
**11.16.13 New onset musculoskeletal and connective tissue disorders ('new medical history'): Gardasil vs. AAHS (males, age 15-27; from 0-7 months)**						
V501-020: Gardasil vs. AAHS (m, 15-27; 36)	29	2032	38	2032	2.6%	0.76 [0.47, 1.23]
**Subtotal (95% CI)**		**2032**		**2032**	**2.6%**	**0.76 [0.47, 1.23]**
Total events	29		38			
Heterogeneity: Not applicable						
Test for overall effect: Z = 1.11 (P = 0.27)						
**11.16.14 New onset musculoskeletal and connective tissue disorders ('new medical history'): Gardasil vs. AAHS (males, age 15-27; from 7-36 months)**						
V501-020: Gardasil vs. AAHS (m, 15-27; 36)	36	2032	40	2033	0.0%	0.90 [0.58, 1.41]
**Subtotal (95% CI)**		**0**		**0**		**Not estimable**
Total events	0		0			
Heterogeneity: Not applicable						
Test for overall effect: Not applicable						
**11.16.15 New onset musculoskeletal and connective tissue disorders ('new medical history'): HPV 16 vaccine vs. AAHS (females, age 16-25; from 0-7 months)**						
V501-005: HPV 16 vaccine vs. AAHS (f, 16-25; 48)	60	1204	57	1205	4.7%	1.05 [0.74, 1.50]
**Subtotal (95% CI)**		**1204**		**1205**	**4.7%**	**1.05 [0.74, 1.50]**
Total events	60		57			
Heterogeneity: Not applicable						
Test for overall effect: Z = 0.29 (P = 0.77)						
**11.16.16 New onset musculoskeletal and connective tissue disorders ('new medical history'): HPV 16 vaccine vs. AAHS (females, age 16-25; from 7-48 months)**						
V501-005: HPV 16 vaccine vs. AAHS (f, 16-25; 48)	60	1204	75	1205	0.0%	0.80 [0.58, 1.11]
**Subtotal (95% CI)**		**0**		**0**		**Not estimable**
Total events	0		0			
Heterogeneity: Not applicable						
Test for overall effect: Not applicable						
**11.16.17 New onset musculoskeletal and connective tissue disorders ('new medical history'): Gardasil 9 vs. saline placebo (females, age 12-26; follow-up until 7 months)**						
V503-006: Gardasil 9 vs. saline (f, 12-26;7)	12	618	2	306	0.3%	2.97 [0.67, 13.19]
**Subtotal (95% CI)**		**618**		**306**	**0.3%**	**2.97 [0.67, 13.19]**
Total events	12		2			
Heterogeneity: Not applicable						
Test for overall effect: Z = 1.43 (P = 0.15)						
**Total (95% CI)**		**30736**		**29762**	**100.0%**	**1.02 [0.94, 1.10]**
Total events	1263		1213			
Heterogeneity: Tau² = 0.00; Chi² = 15.76, df = 16 (P = 0.47); I² = 0%						
Test for overall effect: Z = 0.40 (P = 0.69)						
Test for subgroup differences: Chi² = 15.65, df = 14 (P = 0.34), I² = 10.5%						

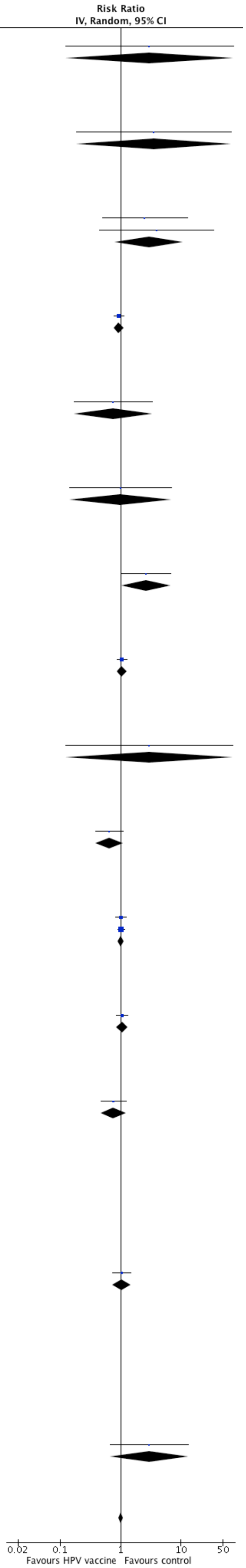

*11.16. Risk ratio for 'medically significant conditions’ (GlaxoSmithKline): 1.05 [0.88, 1.26]; risk ratio for ‘new medical history’ (Merck Sharp & Dohme): 1.01 [0.91, 1.12]; risk ratio for the follow-up periods for the trials V501-005 and V501-020: 0.83 [0.64, 1.09]. The trials V501-005 and V501-020 split the reporting of new onset diseases into the vaccination period and the follow-up period. To avoid double counting of participants in the total risk ratio estimate, we only included the new onset diseases reported in the vaccination period for the trials V501-005 and V501-020.

11.17. New onset diseases ('medically significant conditions' and 'new medical history'*) reported within the MedDRA system organ class ‘neoplasms benign, malignant and unspecified (incl. cysts and polyps) (10029104)': intention to treat analysis

Study or Subgroup	HPV vaccine Events	HPV vaccine Total	Control Events	Control Total	Weight	Risk Ratio IV, Random, 95% CI
**11.17.1 New onset neoplasms ('medically significant conditions'): Cervarix vs. Al(OH)3 (females, age 9-17; follow-up until 12 months)**						
HPV-058: Cervarix vs. Al(OH)3 (f, 9-17; 12)	1	374	0	376	0.2%	3.02 [0.12, 73.80]
**Subtotal (95% CI)**		**374**		**376**	**0.2%**	**3.02 [0.12, 73.80]**
Total events	1		0			
Heterogeneity: Not applicable						
Test for overall effect: Z = 0.68 (P = 0.50)						
**11.17.2 New onset neoplasms ('medically significant conditions'): Cervarix vs. Al(OH)3 (females, age 15-25; follow-up until 7 months)**						
HPV-038: Cervarix vs. Al(OH)3 (f, 15-25; 7)	1	149	0	76	0.2%	1.54 [0.06, 37.36]
**Subtotal (95% CI)**		**149**		**76**	**0.2%**	**1.54 [0.06, 37.36]**
Total events	1		0			
Heterogeneity: Not applicable						
Test for overall effect: Z = 0.27 (P = 0.79)						
**11.17.3 New onset neoplasms ('medically significant conditions'): Cervarix vs. Al(OH)3 (females, age 18-35; follow-up until 7 months)**						
HPV-031: Cervarix vs. Al(OH)3 (f, 18-35; 7)	0	176	1	178	0.2%	0.34 [0.01, 8.22]
**Subtotal (95% CI)**		**176**		**178**	**0.2%**	**0.34 [0.01, 8.22]**
Total events	0		1			
Heterogeneity: Not applicable						
Test for overall effect: Z = 0.67 (P = 0.50)						
**11.17.4 New onset neoplasms ('medically significant conditions'): Cervarix vs. Al(OH)3 (females, age 24-72; follow-up until 36 months)**						
HPV-015: Cervarix vs. Al(OH)3 (f, 24-72; 36)	147	2882	122	2871	30.8%	1.20 [0.95, 1.52]
**Subtotal (95% CI)**		**2882**		**2871**	**30.8%**	**1.20 [0.95, 1.52]**
Total events	147		122			
Heterogeneity: Not applicable						
Test for overall effect: Z = 1.53 (P = 0.13)						
**11.17.5 New onset neoplasms ('medically significant conditions'): Cervarix vs. Aimmugen: hepatitis A vaccine (females, age 22-29; follow-up until 12 months)**						
HPV-063: Cervarix vs. Aimmugen (f, 22-29; 12)	0	375	1	377	0.2%	0.34 [0.01, 8.20]
**Subtotal (95% CI)**		**375**		**377**	**0.2%**	**0.34 [0.01, 8.20]**
Total events	0		1			
Heterogeneity: Not applicable						
Test for overall effect: Z = 0.67 (P = 0.50)						
**11.17.6 New onset neoplasms ('medically significant conditions'): Cervarix vs. Engerix: hepatitis B vaccine (females, age 26-46; follow-up until 12 months)**						
HPV-069: Cervarix vs. Engerix (f, 26-46; 12)	1	606	1	606	0.2%	1.00 [0.06, 15.95]
**Subtotal (95% CI)**		**606**		**606**	**0.2%**	**1.00 [0.06, 15.95]**
Total events	1		1			
Heterogeneity: Not applicable						
Test for overall effect: Z = 0.00 (P = 1.00)						
**11.17.7 New onset neoplasms ('medically significant conditions'): Cervarix vs. Havrix: hepatitis A vaccine (females, age 9-15; follow-up until 12 months)**						
HPV-013: Cervarix vs. Havrix (f, 10-14; 12)	2	1035	4	1032	0.6%	0.50 [0.09, 2.72]
HPV-033: Cervarix vs. Havrix (f, 9-15; 7)	0	160	1	161	0.2%	0.34 [0.01, 8.17]
**Subtotal (95% CI)**		**1195**		**1193**	**0.8%**	**0.46 [0.10, 2.04]**
Total events	2		5			
Heterogeneity: Tau^2 = 0.00; Chi^2 = 0.05, df = 1 (P = 0.83); I^2 = 0%						
Test for overall effect: Z = 1.03 (P = 0.31)						
**11.17.8 New onset neoplasms ('medically significant conditions'): Cervarix vs. Havrix: hepatitis A vaccine (females, age 15-25; follow-up until 48 months)**						
HPV-008: Cervarix vs. Havrix (f, 15-25; 48)	81	9319	84	9325	18.3%	0.96 [0.71, 1.31]
**Subtotal (95% CI)**		**9319**		**9325**	**18.3%**	**0.96 [0.71, 1.31]**
Total events	81		84			
Heterogeneity: Not applicable						
Test for overall effect: Z = 0.23 (P = 0.82)						
**11.17.9 New onset neoplasms ('new medical history'): Gardasil vs. carrier solution (females and males, age 9-16; follow-up until 18 months)**						
V501-018: Gardasil vs. carrier solu. (f&m,9-16;18)	8	1184	6	597	1.5%	0.67 [0.23, 1.93]
**Subtotal (95% CI)**		**1184**		**597**	**1.5%**	**0.67 [0.23, 1.93]**
Total events	8		6			
Heterogeneity: Not applicable						
Test for overall effect: Z = 0.74 (P = 0.46)						
**11.17.10 New onset neoplasms ('new medical history'): Gardasil vs. AAHS (females, age 15-26; follow-up until 45 months)**						
V501-013: Gardasil vs. AAHS (f, 16-24; 45)	75	2723	65	2732	15.8%	1.16 [0.83, 1.61]
V501-015: Gardasil vs. AAHS (f, 15-26; 36)	101	6087	92	6080	21.6%	1.10 [0.83, 1.45]
**Subtotal (95% CI)**		**8810**		**8812**	**37.3%**	**1.12 [0.91, 1.39]**
Total events	176		157			
Heterogeneity: Tau^2 = 0.00; Chi^2 = 0.06, df = 1 (P = 0.81); I^2 = 0%						
Test for overall effect: Z = 1.06 (P = 0.29)						
**11.17.11 New onset neoplasms ('new medical history'): Gardasil vs. AAHS (females, age 21-46; from 0-7 months)**						
V501-019: Gardasil vs. AAHS (f, 21-46; 48)	17	1911	17	1908	3.8%	1.00 [0.51, 1.95]
**Subtotal (95% CI)**		**1911**		**1908**	**3.8%**	**1.00 [0.51, 1.95]**
Total events	17		17			
Heterogeneity: Not applicable						
Test for overall effect: Z = 0.00 (P = 1.00)						
**11.17.12 New onset neoplasms ('new medical history'): Gardasil vs. AAHS (females, age 21-46; from 7-48 months)**						
V501-019: Gardasil vs. AAHS (f, 21-46; 48)	67	1911	80	1908	0.0%	0.84 [0.61, 1.15]
**Subtotal (95% CI)**		**0**		**0**		**Not estimable**
Total events	0		0			
Heterogeneity: Not applicable						
Test for overall effect: Not applicable						
**11.17.13 New onset neoplasms ('new medical history'): Gardasil vs. AAHS (males, age 15-27; from 0-7 months)**						
V501-020: Gardasil vs. AAHS (m, 15-27; 36)	8	2032	9	2032	1.9%	0.89 [0.34, 2.30]
**Subtotal (95% CI)**		**2032**		**2032**	**1.9%**	**0.89 [0.34, 2.30]**
Total events	8		9			
Heterogeneity: Not applicable						
Test for overall effect: Z = 0.24 (P = 0.81)						
**11.17.14 New onset neoplasms ('new medical history'): Gardasil vs. AAHS (males, age 15-27; from 7-36 months)**						
V501-020: Gardasil vs. AAHS (m, 15-27; 36)	11	2032	15	2033	0.0%	0.73 [0.34, 1.59]
**Subtotal (95% CI)**		**0**		**0**		**Not estimable**
Total events	0		0			
Heterogeneity: Not applicable						
Test for overall effect: Not applicable						
**11.17.15 New onset neoplasms ('new medical history'): HPV 16 vaccine vs. AAHS (females, age 16-25; from 7-48 months)**						
V501-005: HPV 16 vaccine vs. AAHS (f, 16-25; 48)	23	1204	18	1205	4.5%	1.28 [0.69, 2.36]
**Subtotal (95% CI)**		**1204**		**1205**	**4.5%**	**1.28 [0.69, 2.36]**
Total events	23		18			
Heterogeneity: Not applicable						
Test for overall effect: Z = 0.79 (P = 0.43)						
**11.17.16 New onset neoplasms ('new medical history'): Gardasil 9 vs. saline placebo (females, age 12-26; follow-up until 7 months)**						
V503-006: Gardasil 9 vs. saline (f, 12-26;7)	1	618	0	306	0.2%	1.49 [0.06, 36.42]
**Subtotal (95% CI)**		**618**		**306**	**0.2%**	**1.49 [0.06, 36.42]**
Total events	1		0			
Heterogeneity: Not applicable						
Test for overall effect: Z = 0.24 (P = 0.81)						
**Total (95% CI)**		**30835**		**29862**	**100.0%**	**1.09 [0.96, 1.25]**
Total events	466		421			
Heterogeneity: Tau^2 = 0.00; Chi^2 = 5.57, df = 15 (P = 0.99); I^2 = 0%						
Test for overall effect: Z = 1.34 (P = 0.18)						
Test for subgroup differences: Chi^2 = 5.46, df = 13 (P = 0.96), I^2 = 0%						

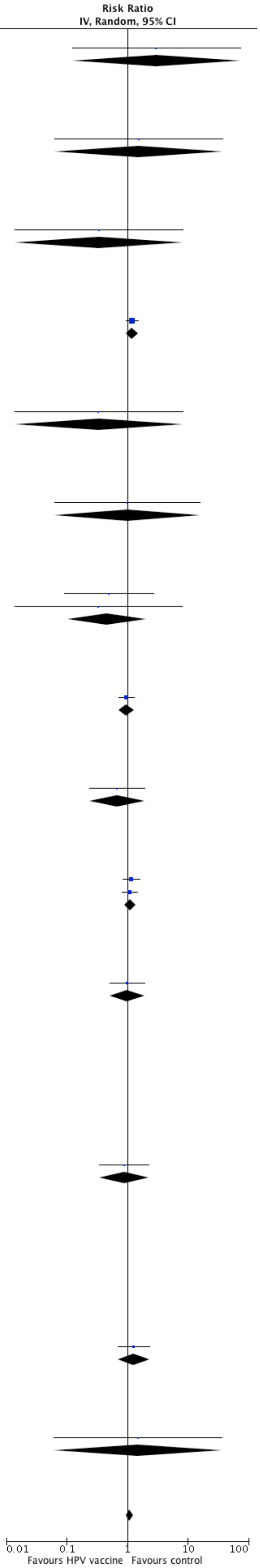

*11.17. Risk ratio for 'medically significant conditions’ (GlaxoSmithKline): 1.09 [0.91, 1.31]; risk ratio for ‘new medical history’ (Merck Sharp & Dohme): 1.10 [0.91, 1.32]; risk ratio for the follow-up periods for the trials V501-019 and V501-020: 0.82 [0.61, 1.10]. The trials V501-019 and V501-020 split the reporting of new onset diseases into the vaccination period and the follow-up period. To avoid double counting of participants in the total risk ratio estimate, we only included the new onset diseases reported in the vaccination period for the trials V501-019 and V501-020.

11.18. New onset diseases ('medically significant conditions' and 'new medical history'*) reported within the MedDRA system organ class ‘nervous system disorders (10029205)': intention to treat analysis

Study or Subgroup	HPV vaccine Events	HPV vaccine Total	Control Events	Control Total	Weight	Risk Ratio IV, Random, 95% CI
**11.18.1 New onset nervous system disorders ('medically significant conditions'): Cervarix vs. Al(OH)3 (females, age 9-17; follow-up until 12 months)**						
HPV-058: Cervarix vs. Al(OH)3 (f, 9-17; 12)	1	374	0	376	0.1%	3.02 [0.12, 73.80]
**Subtotal (95% CI)**		**374**		**376**	**0.1%**	**3.02 [0.12, 73.80]**
Total events	1		0			
Heterogeneity: Not applicable						
Test for overall effect: Z = 0.68 (P = 0.50)						
**11.18.2 New onset nervous system disorders ('medically significant conditions'): Cervarix vs. Al(OH)3 (females, age 18-35; follow-up until 7 months)**						
HPV-031: Cervarix vs. Al(OH)3 (f, 18-35; 7)	0	176	1	178	0.1%	0.34 [0.01, 8.22]
HPV-035: Cervarix vs. Al(OH)3 (f, 18-35; 7)	7	150	1	150	0.2%	7.00 [0.87, 56.20]
**Subtotal (95% CI)**		**326**		**328**	**0.3%**	**1.98 [0.11, 37.05]**
Total events	7		2			
Heterogeneity: Tau^2 = 2.71; Chi^2 = 2.43, df = 1 (P = 0.12); I^2 = 59%						
Test for overall effect: Z = 0.46 (P = 0.65)						
**11.18.3 New onset nervous system disorders ('medically significant conditions'): Cervarix vs. Al(OH)3 (females, age 24-72; follow-up until 36 months)**						
HPV-015: Cervarix vs. Al(OH)3 (f, 24-72; 36)	120	2882	94	2871	9.4%	1.27 [0.98, 1.66]
**Subtotal (95% CI)**		**2882**		**2871**	**9.4%**	**1.27 [0.98, 1.66]**
Total events	120		94			
Heterogeneity: Not applicable						
Test for overall effect: Z = 1.78 (P = 0.08)						
**11.18.4 New onset nervous system disorders ('medically significant conditions'): Cervarix vs. Aimmugen: hepatitis A vaccine (females, age 22-29; follow-up until 12 months)**						
HPV-063: Cervarix vs. Aimmugen (f, 22-29; 12)	5	375	6	377	0.6%	0.84 [0.26, 2.72]
**Subtotal (95% CI)**		**375**		**377**	**0.6%**	**0.84 [0.26, 2.72]**
Total events	5		6			
Heterogeneity: Not applicable						
Test for overall effect: Z = 0.29 (P = 0.77)						
**11.18.5 New onset nervous system disorders ('medically significant conditions'): Cervarix vs. Engerix: hepatitis B vaccine (females and males, age 9-15; follow-up until 12 months)**						
HPV-030: Cervarix vs. Engerix (f, 9-15; 12)	2	247	0	246	0.1%	4.98 [0.24, 103.20]
**Subtotal (95% CI)**		**247**		**246**	**0.1%**	**4.98 [0.24, 103.20]**
Total events	2		0			
Heterogeneity: Not applicable						
Test for overall effect: Z = 1.04 (P = 0.30)						
**11.18.6 New onset nervous system disorders ('medically significant conditions'): Cervarix vs. Engerix: hepatitis B vaccine (females, age 26-46; follow-up until 12 months)**						
HPV-069: Cervarix vs. Engerix (f, 26-46; 12)	1	606	0	606	0.1%	3.00 [0.12, 73.50]
**Subtotal (95% CI)**		**606**		**606**	**0.1%**	**3.00 [0.12, 73.50]**
Total events	1		0			
Heterogeneity: Not applicable						
Test for overall effect: Z = 0.67 (P = 0.50)						
**11.18.7 New onset nervous system disorders ('medically significant conditions'): Cervarix vs. Havrix: hepatitis A vaccine (females, age 10-14; follow-up until 12 months)**						
HPV-013: Cervarix vs. Havrix (f, 10-14; 12)	22	1035	16	1032	1.8%	1.37 [0.72, 2.60]
**Subtotal (95% CI)**		**1035**		**1032**	**1.8%**	**1.37 [0.72, 2.60]**
Total events	22		16			
Heterogeneity: Not applicable						
Test for overall effect: Z = 0.97 (P = 0.33)						
**11.18.8 New onset nervous system disorders ('medically significant conditions'): Cervarix vs. Havrix: hepatitis A vaccine (females, age 15-25; follow-up until 48 months)**						
HPV-008: Cervarix vs. Havrix (f, 15-25; 48)	220	9319	208	9325	16.4%	1.06 [0.88, 1.28]
**Subtotal (95% CI)**		**9319**		**9325**	**16.4%**	**1.06 [0.88, 1.28]**
Total events	220		208			
Heterogeneity: Not applicable						
Test for overall effect: Z = 0.59 (P = 0.55)						
**11.18.9 New onset nervous system disorders ('medically significant conditions'): Cervarix vs. Twinrix: hepatitis A and B vaccine (females, age 8-15; follow-up until 12 months)**						
HPV-029: Cervarix vs. Twinrix (f, 8-15; 12)	0	270	2	271	0.1%	0.20 [0.01, 4.16]
**Subtotal (95% CI)**		**270**		**271**	**0.1%**	**0.20 [0.01, 4.16]**
Total events	0		2			
Heterogeneity: Not applicable						
Test for overall effect: Z = 1.04 (P = 0.30)						
**11.18.10 New onset nervous system disorders ('new medical history'): Gardasil vs. carrier solution (females and males, age 9-16; follow-up until 18 months)**						
V501-018: Gardasil vs. carrier solu. (f&m,9-16;18)	29	1184	8	597	1.3%	1.83 [0.84, 3.97]
**Subtotal (95% CI)**		**1184**		**597**	**1.3%**	**1.83 [0.84, 3.97]**
Total events	29		8			
Heterogeneity: Not applicable						
Test for overall effect: Z = 1.52 (P = 0.13)						
**11.18.11 New onset nervous system disorders ('new medical history'): Gardasil vs. AAHS (females, age 15-26; follow-up until 45 months)**						
V501-013: Gardasil vs. AAHS (f, 16-24; 45)	363	2723	310	2732	24.0%	1.17 [1.02, 1.35]
V501-015: Gardasil vs. AAHS (f, 15-26; 36)	456	6087	461	6080	28.1%	0.99 [0.87, 1.12]
**Subtotal (95% CI)**		**8810**		**8812**	**52.1%**	**1.07 [0.91, 1.27]**
Total events	819		771			
Heterogeneity: Tau^2 = 0.01; Chi^2 = 3.23, df = 1 (P = 0.07); I^2 = 69%						
Test for overall effect: Z = 0.82 (P = 0.41)						
**11.18.12 New onset nervous system disorders ('new medical history'): Gardasil vs. AAHS (females, age 21-46; from 0-7 months)**						
V501-019: Gardasil vs. AAHS (f, 21-46; 48)	93	1911	100	1908	8.8%	0.93 [0.71, 1.22]
**Subtotal (95% CI)**		**1911**		**1908**	**8.8%**	**0.93 [0.71, 1.22]**
Total events	93		100			
Heterogeneity: Not applicable						
Test for overall effect: Z = 0.53 (P = 0.60)						
**11.18.13 New onset nervous system disorders ('new medical history'): Gardasil vs. AAHS (females, age 21-46; from 7-48 months)**						
V501-019: Gardasil vs. AAHS (f, 21-46; 48)	90	1911	85	1908	0.0%	1.06 [0.79, 1.41]
**Subtotal (95% CI)**		**0**		**0**		**Not estimable**
Total events	0		0			
Heterogeneity: Not applicable						
Test for overall effect: Not applicable						
**11.18.14 New onset nervous system disorders ('new medical history'): Gardasil vs. AAHS (males, age 15-27; from 0-7 months)**						
V501-020: Gardasil vs. AAHS (m, 15-27; 36)	28	2032	31	2032	2.9%	0.90 [0.54, 1.50]
**Subtotal (95% CI)**		**2032**		**2032**	**2.9%**	**0.90 [0.54, 1.50]**
Total events	28		31			
Heterogeneity: Not applicable						
Test for overall effect: Z = 0.39 (P = 0.69)						
**11.18.15 New onset nervous system disorders ('new medical history'): Gardasil vs. AAHS (males, age 15-27; from 7-36 months)**						
V501-020: Gardasil vs. AAHS (m, 15-27; 36)	19	2032	14	2033	0.0%	1.36 [0.68, 2.70]
**Subtotal (95% CI)**		**0**		**0**		**Not estimable**
Total events	0		0			
Heterogeneity: Not applicable						
Test for overall effect: Not applicable						
**11.18.16 New onset nervous system disorders ('new medical history'): HPV 16 vaccine vs. AAHS (females, age 16-25; from 0-7 months)**						
V501-005: HPV 16 vaccine vs. AAHS (f, 16-25; 48)	47	1204	58	1205	5.0%	0.81 [0.56, 1.18]
**Subtotal (95% CI)**		**1204**		**1205**	**5.0%**	**0.81 [0.56, 1.18]**
Total events	47		58			
Heterogeneity: Not applicable						
Test for overall effect: Z = 1.09 (P = 0.28)						
**11.18.17 New onset nervous system disorders ('new medical history'): HPV 16 vaccine vs. AAHS (females, age 16-25; from 7-48 months)**						
V501-005: HPV 16 vaccine vs. AAHS (f, 16-25; 48)	58	1204	71	1205	0.0%	0.82 [0.58, 1.15]
**Subtotal (95% CI)**		**0**		**0**		**Not estimable**
Total events	0		0			
Heterogeneity: Not applicable						
Test for overall effect: Not applicable						
**11.18.18 New onset nervous system disorders ('new medical history'): Gardasil 9 vs. saline placebo (females, age 12-26; follow-up until 7 months)**						
V503-006: Gardasil 9 vs. saline (f, 12-26;7)	16	618	9	306	1.2%	0.88 [0.39, 1.97]
**Subtotal (95% CI)**		**618**		**306**	**1.2%**	**0.88 [0.39, 1.97]**
Total events	16		9			
Heterogeneity: Not applicable						
Test for overall effect: Z = 0.31 (P = 0.76)						
**Total (95% CI)**		**31193**		**30292**	**100.0%**	**1.06 [0.97, 1.16]**
Total events	1410		1305			
Heterogeneity: Tau^2 = 0.00; Chi^2 = 17.77, df = 16 (P = 0.34); I^2 = 10%						
Test for overall effect: Z = 1.39 (P = 0.16)						
Test for subgroup differences: Chi^2 = 11.08, df = 14 (P = 0.68), I^2 = 0%						

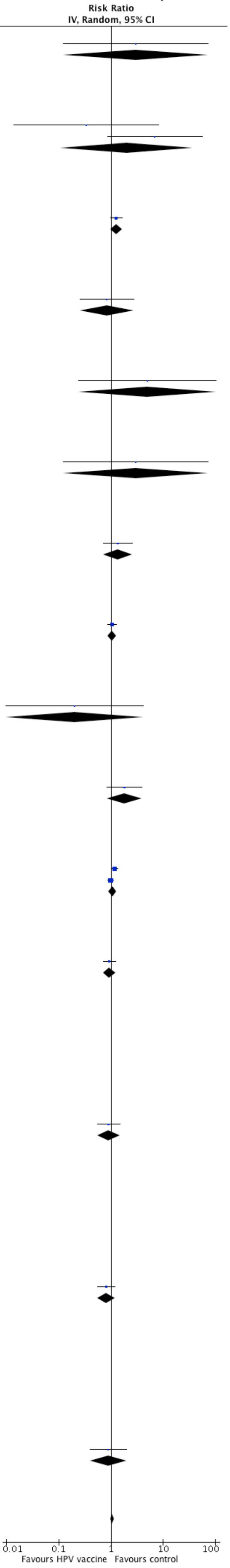

*11.18. Risk ratio for 'medically significant conditions’ (GlaxoSmithKline): 1.14 [0.99, 1.32]; risk ratio for ‘new medical history’ (Merck Sharp & Dohme): 1.02 [0.91, 1.16]; risk ratio for the follow-up periods for the trials V501-005, V501-019 and V501-020: 0.98 [0.78, 1.23]. The trials V501-005, V501-019 and V501-020 split the reporting of new onset diseases into the vaccination period and the follow-up period. To avoid double counting of participants in the total risk ratio estimate, we only included the new onset diseases reported in the vaccination period for the trials V501-005, V501-019 and V501-020.

11.19. New onset diseases ('medically significant conditions' and 'new medical history'*) reported within the MedDRA system organ class ‘pregnancy, puerperium and perinatal conditions (10036585)': intention to treat analysis

Study or Subgroup	HPV vaccine Events	HPV vaccine Total	Control Events	Control Total	Weight	Risk Ratio IV, Random, 95% CI
**11.19.1 New onset pregnancy, puerperium and perinatal conditions ('medically significant conditions'): Cervarix vs. Al(OH)3 (females, age 18-35; follow-up until 7 months)**						
HPV-031: Cervarix vs. Al(OH)3 (f, 18-35; 7)	0	176	1	178	0.1%	0.34 [0.01, 8.22]
**Subtotal (95% CI)**		**176**		**178**	**0.1%**	**0.34 [0.01, 8.22]**
Total events	0		1			
Heterogeneity: Not applicable						
Test for overall effect: Z = 0.67 (P = 0.50)						
**11.19.2 New onset pregnancy, puerperium and perinatal conditions ('medically significant conditions'): Cervarix vs. Al(OH)3 (females, age 24-72; follow-up until 36 months)**						
HPV-015: Cervarix vs. Al(OH)3 (f, 24-72; 36)	92	2882	97	2871	13.1%	0.94 [0.71, 1.25]
**Subtotal (95% CI)**		**2882**		**2871**	**13.1%**	**0.94 [0.71, 1.25]**
Total events	92		97			
Heterogeneity: Not applicable						
Test for overall effect: Z = 0.40 (P = 0.69)						
**11.19.3 New onset pregnancy, puerperium and perinatal conditions ('medically significant conditions'): Cervarix vs. Aimmugen: hepatitis A vaccine (females, age 22-29; follow-up until 12 months)**						
HPV-063: Cervarix vs. Aimmugen (f, 22-29; 12)	15	375	14	377	2.0%	1.08 [0.53, 2.20]
**Subtotal (95% CI)**		**375**		**377**	**2.0%**	**1.08 [0.53, 2.20]**
Total events	15		14			
Heterogeneity: Not applicable						
Test for overall effect: Z = 0.20 (P = 0.84)						
**11.19.4 New onset pregnancy, puerperium and perinatal conditions ('medically significant conditions'): Cervarix vs. Havrix: hepatitis A vaccine (females, age 15-25; follow-up until 48 months)**						
HPV-008: Cervarix vs. Havrix (f, 15-25; 48)	311	9319	273	9325	40.3%	1.14 [0.97, 1.34]
**Subtotal (95% CI)**		**9319**		**9325**	**40.3%**	**1.14 [0.97, 1.34]**
Total events	311		273			
Heterogeneity: Not applicable						
Test for overall effect: Z = 1.60 (P = 0.11)						
**11.19.5 New onset pregnancy, puerperium and perinatal conditions ('new medical history'): Gardasil vs. carrier solution (females and males, age 9-16; follow-up until 18 months)**						
V501-018: Gardasil vs. carrier solu. (f&m,9-16;18)	1	1184	0	597	0.1%	1.51 [0.06, 37.11]
**Subtotal (95% CI)**		**1184**		**597**	**0.1%**	**1.51 [0.06, 37.11]**
Total events	1		0			
Heterogeneity: Not applicable						
Test for overall effect: Z = 0.25 (P = 0.80)						
**11.19.6 New onset pregnancy, puerperium and perinatal conditions ('new medical history'): Gardasil vs. AAHS (females, age 15-26; follow-up until 45 months)**						
V501-013: Gardasil vs. AAHS (f, 16-24; 45)	47	2723	57	2732	7.0%	0.83 [0.56, 1.21]
V501-015: Gardasil vs. AAHS (f, 15-26; 36)	163	6087	170	6080	22.9%	0.96 [0.77, 1.18]
**Subtotal (95% CI)**		**8810**		**8812**	**30.0%**	**0.93 [0.77, 1.11]**
Total events	210		227			
Heterogeneity: $Tau^2 = 0.00$; $Chi^2 = 0.43$, df = 1 (P = 0.51); $I^2 = 0\%$						
Test for overall effect: Z = 0.82 (P = 0.41)						
**11.19.7 New onset pregnancy, puerperium and perinatal conditions ('new medical history'): Gardasil vs. AAHS (females, age 21-46; from 0-7 months)**						
V501-019: Gardasil vs. AAHS (f, 21-46; 48)	4	1911	2	1908	0.4%	2.00 [0.37, 10.89]
**Subtotal (95% CI)**		**1911**		**1908**	**0.4%**	**2.00 [0.37, 10.89]**
Total events	4		2			
Heterogeneity: Not applicable						
Test for overall effect: Z = 0.80 (P = 0.42)						
**11.19.8 New onset pregnancy, puerperium and perinatal conditions ('new medical history'): Gardasil vs. AAHS (females, age 21-46; from 7-48 months)**						
V501-019: Gardasil vs. AAHS (f, 21-46; 48)	32	1911	41	1908	0.0%	0.78 [0.49, 1.23]
**Subtotal (95% CI)**		**0**		**0**		**Not estimable**
Total events	0		0			
Heterogeneity: Not applicable						
Test for overall effect: Not applicable						
**11.19.9 New onset pregnancy, puerperium and perinatal conditions ('new medical history'): Gardasil vs. AAHS (males, age 15-27; from 0-7 months)**						
V501-020: Gardasil vs. AAHS (m, 15-27; 36)	0	2032	1	2032	0.1%	0.33 [0.01, 8.18]
**Subtotal (95% CI)**		**2032**		**2032**	**0.1%**	**0.33 [0.01, 8.18]**
Total events	0		1			
Heterogeneity: Not applicable						
Test for overall effect: Z = 0.67 (P = 0.50)						
**11.19.10 New onset pregnancy, puerperium and perinatal conditions ('new medical history'): HPV 16 vaccine vs. AAHS (females, age 16-25; from 7-48 months)**						
V501-005: HPV 16 vaccine vs. AAHS (f, 16-25; 48)	93	1204	99	1205	14.0%	0.94 [0.72, 1.23]
**Subtotal (95% CI)**		**1204**		**1205**	**14.0%**	**0.94 [0.72, 1.23]**
Total events	93		99			
Heterogeneity: Not applicable						
Test for overall effect: Z = 0.45 (P = 0.66)						
**Total (95% CI)**		**27893**		**27305**	**100.0%**	**1.02 [0.92, 1.12]**
Total events	726		714			
Heterogeneity: $Tau^2 = 0.00$; $Chi^2 = 5.59$, df = 9 (P = 0.78); $I^2 = 0\%$						
Test for overall effect: Z = 0.30 (P = 0.76)						
Test for subgroup differences: $Chi^2 = 5.15$, df = 8 (P = 0.74), $I^2 = 0\%$						

Risk Ratio
IV, Random, 95% CI

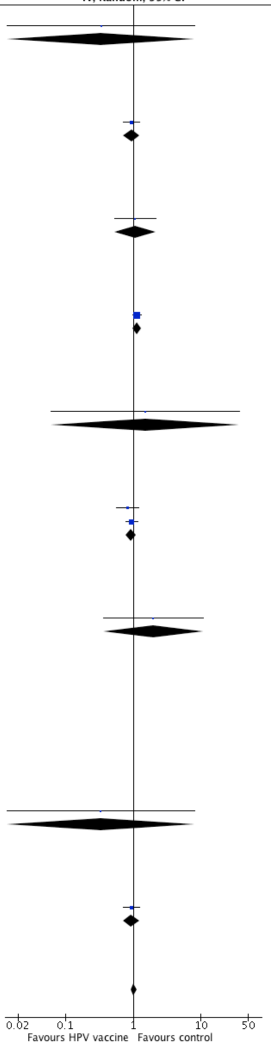

*11.19. Risk ratio for 'medically significant conditions’ (GlaxoSmithKline): 1.09 [0.95, 1.24]; risk ratio for ‘new medical history’ (Merck Sharp & Dohme): 0.93 [0.80, 1.09]; risk ratio for the follow-up period for the trial V501-019: 0.78 [0.49, 1.23]. The trial V501-019 split the reporting of new onset diseases into the vaccination period and the follow-up period. To avoid double counting of participants in the total risk ratio estimate, we only included the new onset diseases reported in the vaccination period for the trial V501-019.

11.20. New onset diseases ('medically significant conditions' and 'new medical history'*) reported within the MedDRA system organ class ‘psychiatric disorders (10037175)': intention to treat analysis

Study or Subgroup	HPV vaccine Events	HPV vaccine Total	Control Events	Control Total	Weight	Risk Ratio IV, Random, 95% CI
**11.20.1 New onset psychiatric disorders ('medically significant conditions'): Cervarix vs. Al(OH)3 (females, age 15-25; follow-up until 7 months)**						
HPV-038: Cervarix vs. Al(OH)3 (f, 15-25; 7)	0	149	1	76	0.1%	0.17 [0.01, 4.15]
**Subtotal (95% CI)**		**149**		**76**	**0.1%**	**0.17 [0.01, 4.15]**
Total events	0		1			
Heterogeneity: Not applicable						
Test for overall effect: Z = 1.09 (P = 0.28)						
**11.20.2 New onset psychiatric disorders ('medically significant conditions'): Cervarix vs. Al(OH)3 (females, age 18-35; follow-up until 7 months)**						
HPV-031: Cervarix vs. Al(OH)3 (f, 18-35; 7)	1	176	0	178	0.1%	3.03 [0.12, 73.97]
**Subtotal (95% CI)**		**176**		**178**	**0.1%**	**3.03 [0.12, 73.97]**
Total events	1		0			
Heterogeneity: Not applicable						
Test for overall effect: Z = 0.68 (P = 0.50)						
**11.20.3 New onset psychiatric disorders ('medically significant conditions'): Cervarix vs. Al(OH)3 (females, age 24-72; follow-up until 36 months)**						
HPV-015: Cervarix vs. Al(OH)3 (f, 24-72; 36)	101	2882	115	2871	11.2%	0.87 [0.67, 1.14]
**Subtotal (95% CI)**		**2882**		**2871**	**11.2%**	**0.87 [0.67, 1.14]**
Total events	101		115			
Heterogeneity: Not applicable						
Test for overall effect: Z = 1.00 (P = 0.32)						
**11.20.4 New onset psychiatric disorders ('medically significant conditions'): Cervarix vs. Aimmugen: hepatitis A vaccine (females, age 22-29; follow-up until 12 months)**						
HPV-063: Cervarix vs. Aimmugen (f, 22-29; 12)	8	375	3	377	0.4%	2.68 [0.72, 10.03]
**Subtotal (95% CI)**		**375**		**377**	**0.4%**	**2.68 [0.72, 10.03]**
Total events	8		3			
Heterogeneity: Not applicable						
Test for overall effect: Z = 1.47 (P = 0.14)						
**11.20.5 New onset psychiatric disorders ('medically significant conditions'): Cervarix vs. Engerix: hepatitis B vaccine (females and males, age 9-15; follow-up until 12 months)**						
HPV-030: Cervarix vs. Engerix (f, 9-15; 12)	0	247	2	246	0.1%	0.20 [0.01, 4.13]
**Subtotal (95% CI)**		**247**		**246**	**0.1%**	**0.20 [0.01, 4.13]**
Total events	0		2			
Heterogeneity: Not applicable						
Test for overall effect: Z = 1.04 (P = 0.30)						
**11.20.6 New onset psychiatric disorders ('medically significant conditions'): Cervarix vs. Havrix: hepatitis A vaccine (females, age 9-15; follow-up until 12 months)**						
HPV-013: Cervarix vs. Havrix (f, 10-14; 12)	0	1035	3	1032	0.1%	0.14 [0.01, 2.75]
HPV-033: Cervarix vs. Havrix (f, 9-15; 7)	2	160	0	161	0.1%	5.03 [0.24, 103.97]
**Subtotal (95% CI)**		**1195**		**1193**	**0.2%**	**0.83 [0.03, 27.43]**
Total events	2		3			
Heterogeneity: $Tau^2 = 4.02$; $Chi^2 = 2.72$, df = 1 (P = 0.10); $I^2 = 63\%$						
Test for overall effect: Z = 0.10 (P = 0.92)						
**11.20.7 New onset psychiatric disorders ('medically significant conditions'): Cervarix vs. Havrix: hepatitis A vaccine (females, age 15-25; follow-up until 48 months)**						
HPV-008: Cervarix vs. Havrix (f, 15-25; 48)	335	9319	327	9325	34.5%	1.03 [0.88, 1.19]
**Subtotal (95% CI)**		**9319**		**9325**	**34.5%**	**1.03 [0.88, 1.19]**
Total events	335		327			
Heterogeneity: Not applicable						
Test for overall effect: Z = 0.33 (P = 0.75)						
**11.20.8 New onset psychiatric disorders ('medically significant conditions'): Cervarix vs. Twinrix: hepatitis A and B vaccine (females, age 8-15; follow-up until 12 months)**						
HPV-029: Cervarix vs. Twinrix (f, 8-15; 12)	1	271	1	271	0.1%	1.00 [0.06, 15.91]
**Subtotal (95% CI)**		**271**		**271**	**0.1%**	**1.00 [0.06, 15.91]**
Total events	1		1			
Heterogeneity: Not applicable						
Test for overall effect: Z = 0.00 (P = 1.00)						
**11.20.9 New onset psychiatric disorders ('new medical history'): Gardasil vs. carrier solution (females and males, age 9-16; follow-up until 18 months)**						
V501-018: Gardasil vs. carrier solu. (f&m,9-16;18)	17	1184	10	597	1.3%	0.86 [0.39, 1.86]
**Subtotal (95% CI)**		**1184**		**597**	**1.3%**	**0.86 [0.39, 1.86]**
Total events	17		10			
Heterogeneity: Not applicable						
Test for overall effect: Z = 0.39 (P = 0.70)						
**11.20.10 New onset psychiatric disorders ('new medical history'): Gardasil vs. AAHS (females, age 15-26; follow-up until 45 months)**						
V501-013: Gardasil vs. AAHS (f, 16-24; 45)	158	2723	161	2732	17.0%	0.98 [0.80, 1.22]
V501-015: Gardasil vs. AAHS (f, 15-26; 36)	256	6087	258	6080	27.0%	0.99 [0.84, 1.17]
**Subtotal (95% CI)**		**8810**		**8812**	**44.0%**	**0.99 [0.87, 1.13]**
Total events	414		419			
Heterogeneity: $Tau^2 = 0.00$; $Chi^2 = 0.00$, df = 1 (P = 0.96); $I^2 = 0\%$						
Test for overall effect: Z = 0.17 (P = 0.87)						
**11.20.11 New onset psychiatric disorders ('new medical history'): Gardasil vs. AAHS (females, age 21-46; from 0-7 months)**						
V501-019: Gardasil vs. AAHS (f, 21-46; 48)	31	1911	30	1908	3.1%	1.03 [0.63, 1.70]
**Subtotal (95% CI)**		**1911**		**1908**	**3.1%**	**1.03 [0.63, 1.70]**
Total events	31		30			
Heterogeneity: Not applicable						
Test for overall effect: Z = 0.12 (P = 0.90)						
**11.20.12 New onset psychiatric disorders ('new medical history'): Gardasil vs. AAHS (females, age 21-46; from 7-48 months)**						
V501-019: Gardasil vs. AAHS (f, 21-46; 48)	31	1911	27	1908	0.0%	1.15 [0.69, 1.91]
**Subtotal (95% CI)**		**0**		**0**		**Not estimable**
Total events	0		0			
Heterogeneity: Not applicable						
Test for overall effect: Not applicable						
**11.20.13 New onset psychiatric disorders ('new medical history'): Gardasil vs. AAHS (males, age 15-27; from 0-7 months)**						
V501-020: Gardasil vs. AAHS (m, 15-27; 36)	14	2032	13	2032	1.4%	1.08 [0.51, 2.29]
**Subtotal (95% CI)**		**2032**		**2032**	**1.4%**	**1.08 [0.51, 2.29]**
Total events	14		13			
Heterogeneity: Not applicable						
Test for overall effect: Z = 0.19 (P = 0.85)						
**11.20.14 New onset psychiatric disorders ('new medical history'): Gardasil vs. AAHS (males, age 15-27; from 7-36 months)**						
V501-020: Gardasil vs. AAHS (m, 15-27; 36)	15	2032	25	2033	0.0%	0.60 [0.32, 1.14]
**Subtotal (95% CI)**		**0**		**0**		**Not estimable**
Total events	0		0			
Heterogeneity: Not applicable						
Test for overall effect: Not applicable						
**11.20.15 New onset psychiatric disorders ('new medical history'): HPV 16 vaccine vs. AAHS (females, age 16-25; from 0-7 months)**						
V501-005: HPV 16 vaccine vs. AAHS (f, 16-25; 48)	35	1204	30	1205	3.3%	1.17 [0.72, 1.89]
**Subtotal (95% CI)**		**1204**		**1205**	**3.3%**	**1.17 [0.72, 1.89]**
Total events	35		30			
Heterogeneity: Not applicable						
Test for overall effect: Z = 0.63 (P = 0.53)						
**11.20.16 New onset psychiatric disorders ('new medical history'): HPV 16 vaccine vs. AAHS (females, age 16-25; from 7-48 months)**						
V501-005: HPV 16 vaccine vs. AAHS (f, 16-25; 48)	83	1204	106	1205	0.0%	0.78 [0.59, 1.03]
**Subtotal (95% CI)**		**0**		**0**		**Not estimable**
Total events	0		0			
Heterogeneity: Not applicable						
Test for overall effect: Not applicable						
**11.20.17 New onset psychiatric disorders ('new medical history'): Gardasil 9 vs. saline placebo (females, age 12-26; follow-up until 7 months)**						
V503-006: Gardasil 9 vs. saline (f, 12-26;7)	2	618	5	306	0.3%	0.20 [0.04, 1.02]
**Subtotal (95% CI)**		**618**		**306**	**0.3%**	**0.20 [0.04, 1.02]**
Total events	2		5			
Heterogeneity: Not applicable						
Test for overall effect: Z = 1.94 (P = 0.05)						
**Total (95% CI)**		**30373**		**29397**	**100.0%**	**0.99 [0.91, 1.08]**
Total events	961		959			
Heterogeneity: $Tau^2 = 0.00$; $Chi^2 = 13.10$, df = 15 (P = 0.59); $I^2 = 0\%$						
Test for overall effect: Z = 0.20 (P = 0.84)						
Test for subgroup differences: $Chi^2 = 10.36$, df = 13 (P = 0.66), $I^2 = 0\%$						

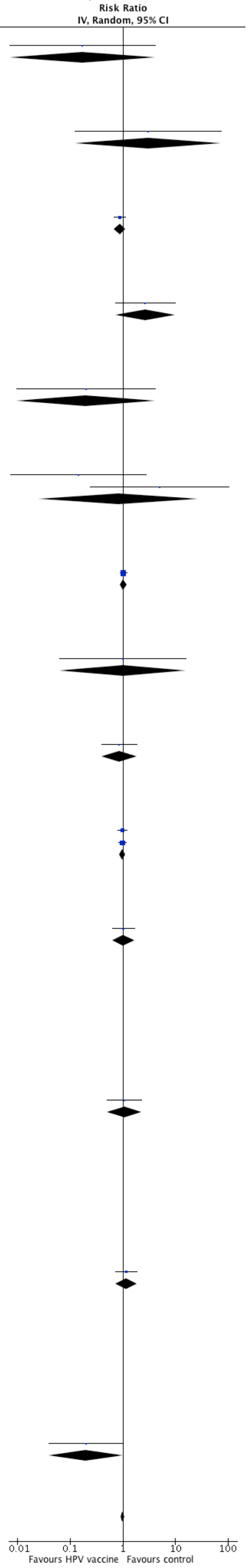

*11.20. Risk ratio for 'medically significant conditions’ (GlaxoSmithKline): 0.98 [0.82, 1.17]; risk ratio for ‘new medical history’ (Merck Sharp & Dohme): 0.99 [0.88, 1.12]; risk ratio for the follow-up periods for the trials V501-005, V501-019 and V501-020: 0.82 [0.62, 1.10]. The trials V501-005, V501-019 and V501-020 split the reporting of new onset diseases into the vaccination period and the follow-up period. To avoid double counting of participants in the total risk ratio estimate, we only included the new onset diseases reported in the vaccination period for the trials V501-005, V501-019 and V501-020.

11.21. New onset diseases ('medically significant conditions' and 'new medical history'*) reported within the MedDRA system organ class ‘renal and urinary disorders (10038359)': intention to treat analysis

Study or Subgroup	HPV vaccine Events	HPV vaccine Total	Control Events	Control Total	Weight	Risk Ratio IV, Random, 95% CI
**11.21.1 New onset renal and urinary disorders ('medically significant conditions'): Cervarix vs. Al(OH)3 (females, age 18-35; follow-up until 7 months)**						
HPV-031: Cervarix vs. Al(OH)3 (f, 18-35; 7)	0	176	1	178	0.2%	0.34 [0.01, 8.22]
**Subtotal (95% CI)**		**176**		**178**	**0.2%**	**0.34 [0.01, 8.22]**
Total events	0		1			
Heterogeneity: Not applicable						
Test for overall effect: Z = 0.67 (P = 0.50)						
**11.21.2 New onset renal and urinary disorders ('medically significant conditions'): Cervarix vs. Al(OH)3 (females, age 24-72; follow-up until 36 months)**						
HPV-015: Cervarix vs. Al(OH)3 (f, 24-72; 36)	44	2882	44	2871	11.0%	1.00 [0.66, 1.51]
**Subtotal (95% CI)**		**2882**		**2871**	**11.0%**	**1.00 [0.66, 1.51]**
Total events	44		44			
Heterogeneity: Not applicable						
Test for overall effect: Z = 0.02 (P = 0.99)						
**11.21.3 New onset renal and urinary disorders ('medically significant conditions'): Cervarix vs. Engerix: hepatitis B vaccine (females and males, age 9-15; follow-up until 12 months)**						
HPV-030: Cervarix vs. Engerix (f, 9-15; 12)	1	247	0	246	0.2%	2.99 [0.12, 72.99]
**Subtotal (95% CI)**		**247**		**246**	**0.2%**	**2.99 [0.12, 72.99]**
Total events	1		0			
Heterogeneity: Not applicable						
Test for overall effect: Z = 0.67 (P = 0.50)						
**11.21.4 New onset renal and urinary disorders ('medically significant conditions'): Cervarix vs. Havrix: hepatitis A vaccine (females, age 10-14; follow-up until 12 months)**						
HPV-013: Cervarix vs. Havrix (f, 10-14; 12)	0	1035	1	1032	0.2%	0.33 [0.01, 8.15]
**Subtotal (95% CI)**		**1035**		**1032**	**0.2%**	**0.33 [0.01, 8.15]**
Total events	0		1			
Heterogeneity: Not applicable						
Test for overall effect: Z = 0.67 (P = 0.50)						
**11.21.5 New onset renal and urinary disorders ('medically significant conditions'): Cervarix vs. Havrix: hepatitis A vaccine (females, age 15-25; follow-up until 48 months)**						
HPV-008: Cervarix vs. Havrix (f, 15-25; 48)	41	9319	43	9325	10.4%	0.95 [0.62, 1.46]
**Subtotal (95% CI)**		**9319**		**9325**	**10.4%**	**0.95 [0.62, 1.46]**
Total events	41		43			
Heterogeneity: Not applicable						
Test for overall effect: Z = 0.22 (P = 0.83)						
**11.21.6 New onset renal and urinary disorders ('new medical history'): Gardasil vs. carrier solution (females and males, age 9-16; follow-up until 18 months)**						
V501-018: Gardasil vs. carrier solu. (f&m,9-16;18)	7	1184	0	597	0.2%	7.57 [0.43, 132.31]
**Subtotal (95% CI)**		**1184**		**597**	**0.2%**	**7.57 [0.43, 132.31]**
Total events	7		0			
Heterogeneity: Not applicable						
Test for overall effect: Z = 1.39 (P = 0.17)						
**11.21.7 New onset renal and urinary disorders ('new medical history'): Gardasil vs. AAHS (females, age 15-26; follow-up until 45 months)**						
V501-013: Gardasil vs. AAHS (f, 16-24; 45)	75	2723	73	2732	18.7%	1.03 [0.75, 1.42]
V501-015: Gardasil vs. AAHS (f, 15-26; 36)	178	6087	175	6080	44.6%	1.02 [0.83, 1.25]
**Subtotal (95% CI)**		**8810**		**8812**	**63.3%**	**1.02 [0.86, 1.21]**
Total events	253		248			
Heterogeneity: $Tau^2 = 0.00$; $Chi^2 = 0.01$, df = 1 (P = 0.94); $I^2 = 0\%$						
Test for overall effect: Z = 0.23 (P = 0.82)						
**11.21.8 New onset renal and urinary disorders ('new medical history'): Gardasil vs. AAHS (females, age 21-46; from 0-7 months)**						
V501-019: Gardasil vs. AAHS (f, 21-46; 48)	10	1911	6	1908	1.8%	1.66 [0.61, 4.57]
**Subtotal (95% CI)**		**1911**		**1908**	**1.8%**	**1.66 [0.61, 4.57]**
Total events	10		6			
Heterogeneity: Not applicable						
Test for overall effect: Z = 0.99 (P = 0.32)						
**11.21.9 New onset renal and urinary disorders ('new medical history'): Gardasil vs. AAHS (females, age 21-46; from 7-48 months)**						
V501-019: Gardasil vs. AAHS (f, 21-46; 48)	16	1911	26	1908	0.0%	0.61 [0.33, 1.14]
**Subtotal (95% CI)**		**0**		**0**		**Not estimable**
Total events	0		0			
Heterogeneity: Not applicable						
Test for overall effect: Not applicable						
**11.21.10 New onset renal and urinary disorders ('new medical history'): Gardasil vs. AAHS (males, age 15-27; from 0-7 months)**						
V501-020: Gardasil vs. AAHS (m, 15-27; 36)	4	2032	4	2032	1.0%	1.00 [0.25, 3.99]
**Subtotal (95% CI)**		**2032**		**2032**	**1.0%**	**1.00 [0.25, 3.99]**
Total events	4		4			
Heterogeneity: Not applicable						
Test for overall effect: Z = 0.00 (P = 1.00)						
**11.21.11 New onset renal and urinary disorders ('new medical history'): Gardasil vs. AAHS (males, age 15-27; from 7-36 months)**						
V501-020: Gardasil vs. AAHS (m, 15-27; 36)	10	2032	19	2033	0.0%	0.53 [0.25, 1.13]
**Subtotal (95% CI)**		**0**		**0**		**Not estimable**
Total events	0		0			
Heterogeneity: Not applicable						
Test for overall effect: Not applicable						
**11.21.12 New onset renal and urinary disorders ('new medical history'): HPV 16 vaccine vs. AAHS (females, age 16-25; from 0-7 months)**						
V501-005: HPV 16 vaccine vs. AAHS (f, 16-25; 48)	44	1204	47	1205	11.6%	0.94 [0.63, 1.40]
**Subtotal (95% CI)**		**1204**		**1205**	**11.6%**	**0.94 [0.63, 1.40]**
Total events	44		47			
Heterogeneity: Not applicable						
Test for overall effect: Z = 0.32 (P = 0.75)						
**11.21.13 New onset renal and urinary disorders ('new medical history'): HPV 16 vaccine vs. AAHS (females, age 16-25; from 7-48 months)**						
V501-005: HPV 16 vaccine vs. AAHS (f, 16-25; 48)	103	1204	110	1205	0.0%	0.94 [0.73, 1.21]
**Subtotal (95% CI)**		**0**		**0**		**Not estimable**
Total events	0		0			
Heterogeneity: Not applicable						
Test for overall effect: Not applicable						
**11.21.14 New onset renal and urinary disorders ('new medical history'): Gardasil 9 vs. saline placebo (females, age 12-26; follow-up until 7 months)**						
V503-006: Gardasil 9 vs. saline (f, 12-26;7)	0	618	1	306	0.2%	0.17 [0.01, 4.05]
**Subtotal (95% CI)**		**618**		**306**	**0.2%**	**0.17 [0.01, 4.05]**
Total events	0		1			
Heterogeneity: Not applicable						
Test for overall effect: Z = 1.10 (P = 0.27)						
**Total (95% CI)**		**29418**		**28512**	**100.0%**	**1.01 [0.88, 1.16]**
Total events	404		395			
Heterogeneity: $Tau^2 = 0.00$; $Chi^2 = 5.66$, df = 11 (P = 0.90); $I^2 = 0\%$						
Test for overall effect: Z = 0.12 (P = 0.90)						
Test for subgroup differences: $Chi^2 = 5.65$, df = 10 (P = 0.84), $I^2 = 0\%$						

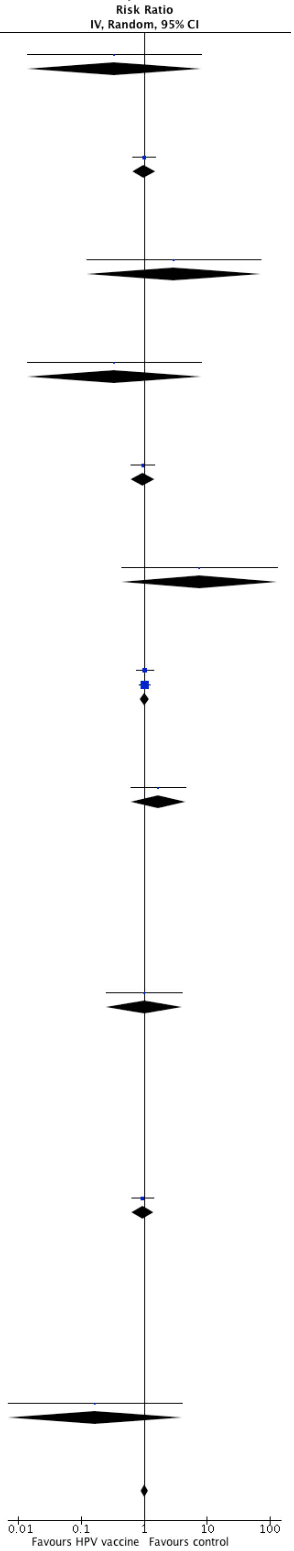

*11.21. Risk ratio for 'medically significant conditions’ (GlaxoSmithKline): 0.97 [0.72, 1.30]; risk ratio for ‘new medical history’ (Merck Sharp & Dohme): 1.02 [0.87, 1.19] risk ratio for the follow-up periods for the trials V501-005, V501-019 and V501-020: 0.77 [0.53, 1.10]. The trials V501-005, V501-019 and V501-020 split the reporting of new onset diseases into the vaccination period and the follow-up period. To avoid double counting of participants in the total risk ratio estimate, we only included the new onset diseases reported in the vaccination period for the trials V501-005, V501-019 and V501-020.

11.22. New onset diseases ('medically significant conditions' and 'new medical history'*) reported within the MedDRA system organ class ‘reproductive system and breast disorders (10038604)': intention to treat analysis

Study or Subgroup	HPV vaccine Events	HPV vaccine Total	Control Events	Control Total	Weight	Risk Ratio IV, Random, 95% CI
**11.22.1 New onset reproductive system and breast disorders ('medically significant conditions'): Cervarix vs. Al(OH)3 (females, age 9-17; follow-up until 12 months)**						
HPV-058: Cervarix vs. Al(OH)3 (f, 9-17; 12)	0	374	1	376	0.0%	0.34 [0.01, 8.20]
**Subtotal (95% CI)**		**374**		**376**	**0.0%**	**0.34 [0.01, 8.20]**
Total events	0		1			
Heterogeneity: Not applicable						
Test for overall effect: Z = 0.67 (P = 0.50)						
**11.22.2 New onset reproductive system and breast disorders ('medically significant conditions'): Cervarix vs. Al(OH)3 (females, age 15-25; follow-up until 7 months)**						
HPV-038: Cervarix vs. Al(OH)3 (f, 15-25; 7)	2	149	0	76	0.0%	2.57 [0.12, 52.80]
**Subtotal (95% CI)**		**149**		**76**	**0.0%**	**2.57 [0.12, 52.80]**
Total events	2		0			
Heterogeneity: Not applicable						
Test for overall effect: Z = 0.61 (P = 0.54)						
**11.22.3 New onset reproductive system and breast disorders ('medically significant conditions'): Cervarix vs. Al(OH)3 (females, age 18-35; follow-up until 7 months)**						
HPV-031: Cervarix vs. Al(OH)3 (f, 18-35; 7)	2	176	1	78	0.0%	0.89 [0.08, 9.63]
HPV-035: Cervarix vs. Al(OH)3 (f, 18-35; 7)	3	150	2	150	0.1%	1.50 [0.25, 8.85]
**Subtotal (95% CI)**		**326**		**228**	**0.1%**	**1.24 [0.30, 5.17]**
Total events	5		3			
Heterogeneity: Tau^2 = 0.00; Chi^2 = 0.12, df = 1 (P = 0.73); I^2 = 0%						
Test for overall effect: Z = 0.30 (P = 0.76)						
**11.22.4 New onset reproductive system and breast disorders ('medically significant conditions'): Cervarix vs. Al(OH)3 (females, age 24-72; follow-up until 36 months)**						
HPV-015: Cervarix vs. Al(OH)3 (f, 24-72; 36)	175	2882	186	2871	4.1%	0.94 [0.77, 1.14]
**Subtotal (95% CI)**		**2882**		**2871**	**4.1%**	**0.94 [0.77, 1.14]**
Total events	175		186			
Heterogeneity: Not applicable						
Test for overall effect: Z = 0.64 (P = 0.53)						
**11.22.5 New onset reproductive system and breast disorders ('medically significant conditions'): Cervarix vs. Aimmugen: hepatitis A vaccine (females, age 22-29; follow-up until 12 months)**						
HPV-063: Cervarix vs. Aimmugen (f, 22-29; 12)	4	375	4	377	0.1%	1.01 [0.25, 3.99]
**Subtotal (95% CI)**		**375**		**377**	**0.1%**	**1.01 [0.25, 3.99]**
Total events	4		4			
Heterogeneity: Not applicable						
Test for overall effect: Z = 0.01 (P = 0.99)						
**11.22.6 New onset reproductive system and breast disorders ('medically significant conditions'): Cervarix vs. Engerix: hepatitis B vaccine (females and males, age 9-15; follow-up until 12 months)**						
HPV-030: Cervarix vs. Engerix (f, 9-15; 12)	0	247	1	246	0.0%	0.33 [0.01, 8.11]
**Subtotal (95% CI)**		**247**		**246**	**0.0%**	**0.33 [0.01, 8.11]**
Total events	0		1			
Heterogeneity: Not applicable						
Test for overall effect: Z = 0.68 (P = 0.50)						
**11.22.7 New onset reproductive system and breast disorders ('medically significant conditions'): Cervarix vs. Havrix: hepatitis A vaccine (females, age 9-15; follow-up until 12 months)**						
HPV-013: Cervarix vs. Havrix (f, 10-14; 12)	3	1035	7	1032	0.1%	0.43 [0.11, 1.65]
HPV-033: Cervarix vs. Havrix (f, 9-15; 7)	1	160	0	161	0.0%	3.02 [0.12, 73.55]
**Subtotal (95% CI)**		**1195**		**1193**	**0.1%**	**0.65 [0.13, 3.14]**
Total events	4		7			
Heterogeneity: Tau^2 = 0.35; Chi^2 = 1.22, df = 1 (P = 0.27); I^2 = 18%						
Test for overall effect: Z = 0.54 (P = 0.59)						
**11.22.8 New onset reproductive system and breast disorders ('medically significant conditions'): Cervarix vs. Havrix: hepatitis A vaccine (females, age 15-25; follow-up until 48 months)**						
HPV-008: Cervarix vs. Havrix (f, 15-25; 48)	283	9319	307	9325	6.5%	0.92 [0.79, 1.08]
**Subtotal (95% CI)**		**9319**		**9325**	**6.5%**	**0.92 [0.79, 1.08]**
Total events	283		307			
Heterogeneity: Not applicable						
Test for overall effect: Z = 1.00 (P = 0.32)						
**11.22.9 New onset reproductive system and breast disorders ('new medical history'): Gardasil vs. carrier solution (females and males, age 9-16; follow-up until 18 months)**						
V501-018: Gardasil vs. carrier solu. (f&m,9-16;18)	21	1184	9	597	0.3%	1.18 [0.54, 2.55]
**Subtotal (95% CI)**		**1184**		**597**	**0.3%**	**1.18 [0.54, 2.55]**
Total events	21		9			
Heterogeneity: Not applicable						
Test for overall effect: Z = 0.41 (P = 0.68)						
**11.22.10 New onset reproductive system and breast disorders ('new medical history'): Gardasil vs. AAHS (females, age 15-26; follow-up until 45 months)**						
V501-013: Gardasil vs. AAHS (f, 16-24; 45)	964	2723	997	2732	32.5%	0.97 [0.90, 1.04]
V501-015: Gardasil vs. AAHS (f, 15-26; 36)	1703	6087	1668	6080	49.6%	1.02 [0.96, 1.08]
**Subtotal (95% CI)**		**8810**		**8812**	**82.1%**	**1.00 [0.95, 1.05]**
Total events	2667		2665			
Heterogeneity: Tau^2 = 0.00; Chi^2 = 1.15, df = 1 (P = 0.28); I^2 = 13%						
Test for overall effect: Z = 0.04 (P = 0.97)						
**11.22.11 New onset reproductive system and breast disorders ('new medical history'): Gardasil vs. AAHS (females, age 21-46; from 0-7 months)**						
V501-019: Gardasil vs. AAHS (f, 21-46; 48)	117	1911	102	1908	2.5%	1.15 [0.88, 1.48]
**Subtotal (95% CI)**		**1911**		**1908**	**2.5%**	**1.15 [0.88, 1.48]**
Total events	117		102			
Heterogeneity: Not applicable						
Test for overall effect: Z = 1.03 (P = 0.30)						
**11.22.12 New onset reproductive system and breast disorders ('new medical history'): Gardasil vs. AAHS (females, age 21-46; from 7-48 months)**						
V501-019: Gardasil vs. AAHS (f, 21-46; 48)	268	1911	277	1908	0.0%	0.97 [0.83, 1.13]
**Subtotal (95% CI)**		**0**		**0**		**Not estimable**
Total events	0		0			
Heterogeneity: Not applicable						
Test for overall effect: Not applicable						
**11.22.13 New onset reproductive system and breast disorders ('new medical history'): Gardasil vs. AAHS (males, age 15-27; from 0-7 months)**						
V501-020: Gardasil vs. AAHS (m, 15-27; 36)	17	2032	14	2032	0.3%	1.21 [0.60, 2.46]
**Subtotal (95% CI)**		**2032**		**2032**	**0.3%**	**1.21 [0.60, 2.46]**
Total events	17		14			
Heterogeneity: Not applicable						
Test for overall effect: Z = 0.54 (P = 0.59)						
**11.22.14 New onset reproductive system and breast disorders ('new medical history'): Gardasil vs. AAHS (males, age 15-27; from 7-36 months)**						
V501-020: Gardasil vs. AAHS (m, 15-27; 36)	19	2032	34	2033	0.0%	0.56 [0.32, 0.98]
**Subtotal (95% CI)**		**0**		**0**		**Not estimable**
Total events	0		0			
Heterogeneity: Not applicable						
Test for overall effect: Not applicable						
**11.22.15 New onset reproductive system and breast disorders ('new medical history'): HPV 16 vaccine vs. AAHS (females, age 16-25; from 0-7 months)**						
V501-005: HPV 16 vaccine vs. AAHS (f, 16-25; 48)	155	1204	156	1205	3.8%	0.99 [0.81, 1.22]
**Subtotal (95% CI)**		**1204**		**1205**	**3.8%**	**0.99 [0.81, 1.22]**
Total events	155		156			
Heterogeneity: Not applicable						
Test for overall effect: Z = 0.05 (P = 0.96)						
**11.22.16 New onset reproductive system and breast disorders ('new medical history'): HPV 16 vaccine vs. AAHS (females, age 16-25; from 7-48 months)**						
V501-005: HPV 16 vaccine vs. AAHS (f, 16-25; 48)	361	1204	339	1205	0.0%	1.07 [0.94, 1.21]
**Subtotal (95% CI)**		**0**		**0**		**Not estimable**
Total events	0		0			
Heterogeneity: Not applicable						
Test for overall effect: Not applicable						
**11.22.17 New onset reproductive system and breast disorders ('new medical history'): Gardasil 9 vs. saline placebo (females, age 12-26; follow-up until 7 months)**						
V503-006: Gardasil 9 vs. saline (f, 12-26;7)	8	618	8	306	0.2%	0.50 [0.19, 1.31]
**Subtotal (95% CI)**		**618**		**306**	**0.2%**	**0.50 [0.19, 1.31]**
Total events	8		8			
Heterogeneity: Not applicable						
Test for overall effect: Z = 1.42 (P = 0.16)						
**Total (95% CI)**		**30626**		**29552**	**100.0%**	**0.99 [0.95, 1.04]**
Total events	3458		3463			
Heterogeneity: Tau^2 = 0.00; Chi^2 = 9.49, df = 16 (P = 0.89); I^2 = 0%						
Test for overall effect: Z = 0.27 (P = 0.78)						
Test for subgroup differences: Chi^2 = 6.51, df = 13 (P = 0.93), I^2 = 0%						

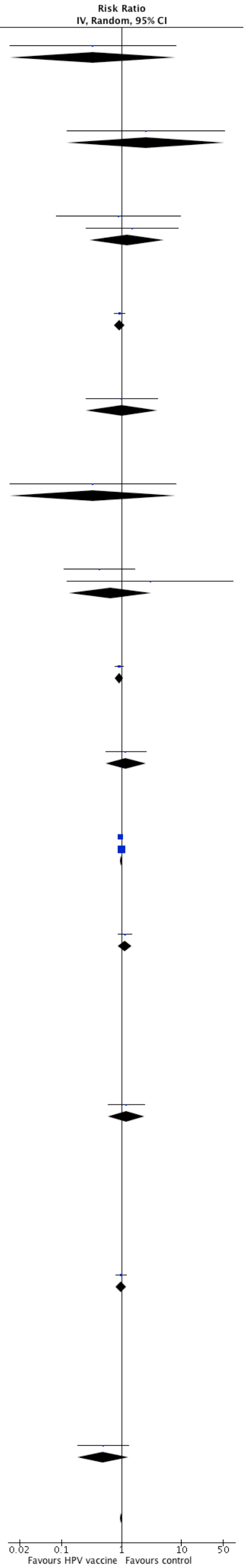

*11.22. Risk ratio for 'medically significant conditions’ (GlaxoSmithKline): 0.93 [0.82, 1.05]; risk ratio for ‘new medical history’ (Merck Sharp & Dohme): 1.00 [0.96, 1.05]; risk ratio for the follow-up periods for the trials V501-005, V501-019 and V501-020: 0.96 [0.79, 1.16]. The trials V501-005, V501-019 and V501-020 split the reporting of new onset diseases into the vaccination period and the follow-up period. To avoid double counting of participants in the total risk ratio estimate, we only included the new onset diseases reported in the vaccination period for the trials V501-005, V501-019 and V501-020.

11.23. New onset diseases ('medically significant conditions' and 'new medical history'*) reported within the MedDRA system organ class ‘respiratory, thoracic and mediastinal disorders (10038738)': intention to treat analysis

Study or Subgroup	HPV vaccine Events	HPV vaccine Total	Control Events	Control Total	Weight	Risk Ratio IV, Random, 95% CI
**11.23.1 New onset respiratory, thoracic and mediastinal disorders ('medically significant conditions'): Cervarix vs. Al(OH)3 (females, age 9-17; follow-up until 12 months)**						
HPV-058: Cervarix vs. Al(OH)3 (f, 9-17; 12)	2	374	0	376	0.1%	5.03 [0.24, 104.35]
**Subtotal (95% CI)**		**374**		**376**	**0.1%**	**5.03 [0.24, 104.35]**
Total events	2		0			
Heterogeneity: Not applicable						
Test for overall effect: Z = 1.04 (P = 0.30)						
**11.23.2 New onset respiratory, thoracic and mediastinal disorders ('medically significant conditions'): Cervarix vs. Al(OH)3 (females, age 15-25; follow-up until 7 months)**						
HPV-038: Cervarix vs. Al(OH)3 (f, 15-25; 7)	1	149	1	76	0.1%	0.51 [0.03, 8.04]
**Subtotal (95% CI)**		**149**		**76**	**0.1%**	**0.51 [0.03, 8.04]**
Total events	1		1			
Heterogeneity: Not applicable						
Test for overall effect: Z = 0.48 (P = 0.63)						
**11.23.3 New onset respiratory, thoracic and mediastinal disorders ('medically significant conditions'): Cervarix vs. Al(OH)3 (females, age 18-35; follow-up until 7 months)**						
HPV-031: Cervarix vs. Al(OH)3 (f, 18-35; 7)	0	176	3	178	0.1%	0.14 [0.01, 2.78]
HPV-035: Cervarix vs. Al(OH)3 (f, 18-35; 7)	11	150	6	150	1.0%	1.83 [0.70, 4.83]
**Subtotal (95% CI)**		**326**		**328**	**1.1%**	**0.77 [0.07, 8.16]**
Total events	11		9			
Heterogeneity: Tau^2 = 1.97; Chi^2 = 2.56, df = 1 (P = 0.11); I^2 = 61%						
Test for overall effect: Z = 0.22 (P = 0.83)						
**11.23.4 New onset respiratory, thoracic and mediastinal disorders ('medically significant conditions'): Cervarix vs. Al(OH)3 (females, age 24-72; follow-up until 36 months)**						
HPV-015: Cervarix vs. Al(OH)3 (f, 24-72; 36)	84	2882	70	2871	9.7%	1.20 [0.87, 1.63]
**Subtotal (95% CI)**		**2882**		**2871**	**9.7%**	**1.20 [0.87, 1.63]**
Total events	84		70			
Heterogeneity: Not applicable						
Test for overall effect: Z = 1.12 (P = 0.26)						
**11.23.5 New onset respiratory, thoracic and mediastinal disorders ('medically significant conditions'): Cervarix vs. Aimmugen: hepatitis A vaccine (females, age 22-29; follow-up until 12 months)**						
HPV-063: Cervarix vs. Aimmugen (f, 22-29; 12)	13	375	8	377	1.3%	1.63 [0.69, 3.90]
**Subtotal (95% CI)**		**375**		**377**	**1.3%**	**1.63 [0.69, 3.90]**
Total events	13		8			
Heterogeneity: Not applicable						
Test for overall effect: Z = 1.11 (P = 0.27)						
**11.23.6 New onset respiratory, thoracic and mediastinal disorders ('medically significant conditions'): Cervarix vs. Engerix: hepatitis B vaccine (females and males, age 9-15; follow-up until 12 months)**						
HPV-030: Cervarix vs. Engerix (f, 9-15; 12)	3	247	2	246	0.3%	1.49 [0.25, 8.86]
**Subtotal (95% CI)**		**247**		**246**	**0.3%**	**1.49 [0.25, 8.86]**
Total events	3		2			
Heterogeneity: Not applicable						
Test for overall effect: Z = 0.44 (P = 0.66)						
**11.23.7 New onset respiratory, thoracic and mediastinal disorders ('medically significant conditions'): Cervarix vs. Havrix: hepatitis A vaccine (females, age 9-15; follow-up until 12 months)**						
HPV-013: Cervarix vs. Havrix (f, 10-14; 12)	35	1035	28	1032	4.0%	1.25 [0.76, 2.03]
HPV-033: Cervarix vs. Havrix (f, 9-15; 7)	1	160	1	161	0.1%	1.01 [0.06, 15.95]
**Subtotal (95% CI)**		**1195**		**1193**	**4.1%**	**1.24 [0.76, 2.00]**
Total events	36		29			
Heterogeneity: Tau^2 = 0.00; Chi^2 = 0.02, df = 1 (P = 0.88); I^2 = 0%						
Test for overall effect: Z = 0.87 (P = 0.38)						
**11.23.8 New onset respiratory, thoracic and mediastinal disorders ('medically significant conditions'): Cervarix vs. Havrix: hepatitis A vaccine (females, age 15-25; follow-up until 48 months)**						
HPV-008: Cervarix vs. Havrix (f, 15-25; 48)	126	9319	147	9325	17.1%	0.86 [0.68, 1.09]
**Subtotal (95% CI)**		**9319**		**9325**	**17.1%**	**0.86 [0.68, 1.09]**
Total events	126		147			
Heterogeneity: Not applicable						
Test for overall effect: Z = 1.27 (P = 0.20)						
**11.23.9 New onset respiratory, thoracic and mediastinal disorders ('medically significant conditions'): Cervarix vs. Twinrix: hepatitis A and B vaccine (females, age 8-15; follow-up until 12 months)**						
HPV-029: Cervarix vs. Twinrix (f, 8-15; 12)	3	270	6	271	0.5%	0.50 [0.13, 1.99]
**Subtotal (95% CI)**		**270**		**271**	**0.5%**	**0.50 [0.13, 1.99]**
Total events	3		6			
Heterogeneity: Not applicable						
Test for overall effect: Z = 0.98 (P = 0.33)						
**11.23.10 New onset respiratory, thoracic and mediastinal disorders ('new medical history'): Gardasil vs. carrier solution (females and males, age 9-16; follow-up until 18 months)**						
V501-018: Gardasil vs. carrier solu. (f&m,9-16;18)	31	1184	22	597	3.3%	0.71 [0.42, 1.22]
**Subtotal (95% CI)**		**1184**		**597**	**3.3%**	**0.71 [0.42, 1.22]**
Total events	31		22			
Heterogeneity: Not applicable						
Test for overall effect: Z = 1.25 (P = 0.21)						
**11.23.11 New onset respiratory, thoracic and mediastinal disorders ('new medical history'): Gardasil vs. AAHS (females, age 15-26; follow-up until 45 months)**						
V501-013: Gardasil vs. AAHS (f, 16-24; 45)	167	2723	165	2732	22.0%	1.02 [0.82, 1.25]
V501-015: Gardasil vs. AAHS (f, 15-26; 36)	208	6087	208	6080	26.8%	1.00 [0.83, 1.21]
**Subtotal (95% CI)**		**8810**		**8812**	**48.7%**	**1.01 [0.87, 1.16]**
Total events	375		373			
Heterogeneity: Tau^2 = 0.00; Chi^2 = 0.01, df = 1 (P = 0.91); I^2 = 0%						
Test for overall effect: Z = 0.09 (P = 0.93)						
**11.23.12 New onset respiratory, thoracic and mediastinal disorders ('new medical history'): Gardasil vs. AAHS (females, age 21-46; from 0-7 months)**						
V501-019: Gardasil vs. AAHS (f, 21-46; 48)	32	1911	33	1908	4.1%	0.97 [0.60, 1.57]
**Subtotal (95% CI)**		**1911**		**1908**	**4.1%**	**0.97 [0.60, 1.57]**
Total events	32		33			
Heterogeneity: Not applicable						
Test for overall effect: Z = 0.13 (P = 0.90)						
**11.23.13 New onset respiratory, thoracic and mediastinal disorders ('new medical history'): Gardasil vs. AAHS (females, age 21-46; from 7-48 months)**						
V501-019: Gardasil vs. AAHS (f, 21-46; 48)	40	1911	41	1908	0.0%	0.97 [0.63, 1.50]
**Subtotal (95% CI)**		**0**		**0**		**Not estimable**
Total events	0		0			
Heterogeneity: Not applicable						
Test for overall effect: Not applicable						
**11.23.14 New onset respiratory, thoracic and mediastinal disorders ('new medical history'): Gardasil vs. AAHS (males, age 15-27; from 0-7 months)**						
V501-020: Gardasil vs. AAHS (m, 15-27; 36)	23	2032	24	2032	3.0%	0.96 [0.54, 1.69]
**Subtotal (95% CI)**		**2032**		**2032**	**3.0%**	**0.96 [0.54, 1.69]**
Total events	23		24			
Heterogeneity: Not applicable						
Test for overall effect: Z = 0.15 (P = 0.88)						
**11.23.15 New onset respiratory, thoracic and mediastinal disorders ('new medical history'): Gardasil vs. AAHS (males, age 15-27; from 7-36 months)**						
V501-020: Gardasil vs. AAHS (m, 15-27; 36)	18	2032	17	2033	0.0%	1.06 [0.55, 2.05]
**Subtotal (95% CI)**		**0**		**0**		**Not estimable**
Total events	0		0			
Heterogeneity: Not applicable						
Test for overall effect: Not applicable						
**11.23.16 New onset respiratory, thoracic and mediastinal disorders ('new medical history'): HPV 16 vaccine vs. AAHS (females, age 16-25; from 0-7 months)**						
V501-005: HPV 16 vaccine vs. AAHS (f, 16-25; 48)	47	1204	42	1205	5.7%	1.12 [0.74, 1.68]
**Subtotal (95% CI)**		**1204**		**1205**	**5.7%**	**1.12 [0.74, 1.68]**
Total events	47		42			
Heterogeneity: Not applicable						
Test for overall effect: Z = 0.54 (P = 0.59)						
**11.23.17 New onset respiratory, thoracic and mediastinal disorders ('new medical history'): HPV 16 vaccine vs. AAHS (females, age 16-25; from 7-48 months)**						
V501-005: HPV 16 vaccine vs. AAHS (f, 16-25; 48)	38	1204	42	1205	0.0%	0.91 [0.59, 1.39]
**Subtotal (95% CI)**		**0**		**0**		**Not estimable**
Total events	0		0			
Heterogeneity: Not applicable						
Test for overall effect: Not applicable						
**11.23.18 New onset respiratory, thoracic and mediastinal disorders ('new medical history'): Gardasil 9 vs. saline placebo (females, age 12-26; follow-up until 7 months)**						
V503-006: Gardasil 9 vs. saline (f, 12-26;7)	8	618	5	306	0.8%	0.79 [0.26, 2.40]
**Subtotal (95% CI)**		**618**		**306**	**0.8%**	**0.79 [0.26, 2.40]**
Total events	8		5			
Heterogeneity: Not applicable						
Test for overall effect: Z = 0.41 (P = 0.68)						
**Total (95% CI)**		**30896**		**29923**	**100.0%**	**1.00 [0.91, 1.11]**
Total events	795		771			
Heterogeneity: Tau^2 = 0.00; Chi^2 = 12.58, df = 17 (P = 0.76); I^2 = 0%						
Test for overall effect: Z = 0.05 (P = 0.96)						
Test for subgroup differences: Chi^2 = 9.44, df = 14 (P = 0.80), I^2 = 0%						

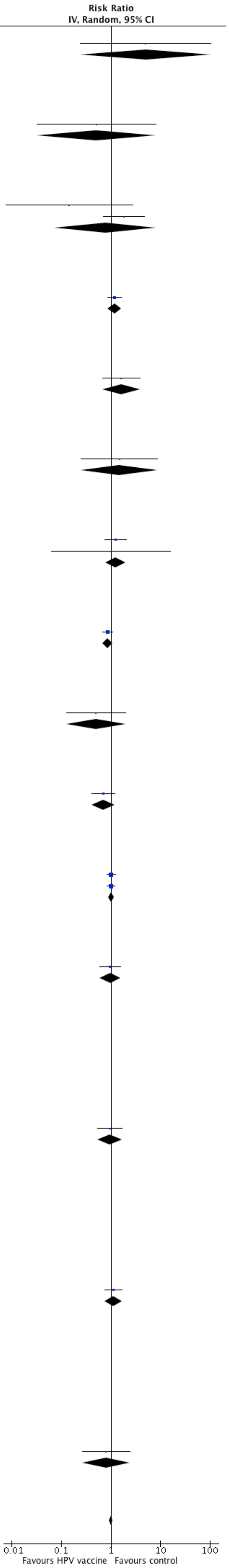

*11.23. Risk ratio for 'medically significant conditions’ (GlaxoSmithKline): 1.04 [0.87, 1.25]; risk ratio for ‘new medical history’ (Merck Sharp & Dohme): 0.99 [0.88, 1.12]; risk ratio for the follow-up periods for the trials V501-005, V501-019 and V501-020: 0.96 [0.73, 1.27]. The trials V501-005, V501-019 and V501-020 split the reporting of new onset diseases into the vaccination period and the follow-up period. To avoid double counting of participants in the total risk ratio estimate, we only included the new onset diseases reported in the vaccination period for the trials V501-005, V501-019 and V501-020.

11.24. New onset diseases ('medically significant conditions' and 'new medical history'*) reported within the MedDRA system organ class ‘skin and subcutaneous tissue disorders (10040785)': intention to treat analysis

Study or Subgroup	HPV vaccine Events	HPV vaccine Total	Control Events	Control Total	Weight	Risk Ratio IV, Random, 95% CI
**11.24.1 New onset skin and subcutaneous tissue disorders ('medically significant conditions'): Cervarix vs. Al(OH)3 (females, age 9-17; follow-up until 12 months)**						
HPV-058: Cervarix vs. Al(OH)3 (f, 9-17; 12)	0	374	2	376	0.1%	0.20 [0.01, 4.17]
**Subtotal (95% CI)**		**374**		**376**	**0.1%**	**0.20 [0.01, 4.17]**
Total events	0		2			
Heterogeneity: Not applicable						
Test for overall effect: Z = 1.04 (P = 0.30)						
**11.24.2 New onset skin and subcutaneous tissue disorders ('medically significant conditions'): Cervarix vs. Al(OH)3 (females, age 15-25; follow-up until 7 months)**						
HPV-038: Cervarix vs. Al(OH)3 (f, 15-25; 7)	10	149	2	76	0.6%	2.55 [0.57, 11.35]
**Subtotal (95% CI)**		**149**		**76**	**0.6%**	**2.55 [0.57, 11.35]**
Total events	10		2			
Heterogeneity: Not applicable						
Test for overall effect: Z = 1.23 (P = 0.22)						
**11.24.3 New onset skin and subcutaneous tissue disorders ('medically significant conditions'): Cervarix vs. Al(OH)3 (females, age 18-35; follow-up until 7 months)**						
HPV-031: Cervarix vs. Al(OH)3 (f, 18-35; 7)	0	176	4	178	0.2%	0.11 [0.01, 2.07]
HPV-035: Cervarix vs. Al(OH)3 (f, 18-35; 7)	9	150	2	150	0.6%	4.50 [0.99, 20.48]
**Subtotal (95% CI)**		**326**		**328**	**0.8%**	**0.88 [0.02, 32.08]**
Total events	9		6			
Heterogeneity: $Tau^2 = 5.40$; $Chi^2 = 4.85$, df = 1 (P = 0.03); $I^2 = 79\%$						
Test for overall effect: Z = 0.07 (P = 0.95)						
**11.24.4 New onset skin and subcutaneous tissue disorders ('medically significant conditions'): Cervarix vs. Al(OH)3 (females, age 24-72; follow-up until 36 months)**						
HPV-015: Cervarix vs. Al(OH)3 (f, 24-72; 36)	65	2882	77	2871	8.9%	0.84 [0.61, 1.17]
**Subtotal (95% CI)**		**2882**		**2871**	**8.9%**	**0.84 [0.61, 1.17]**
Total events	65		77			
Heterogeneity: Not applicable						
Test for overall effect: Z = 1.04 (P = 0.30)						
**11.24.5 New onset skin and subcutaneous tissue disorders ('medically significant conditions'): Cervarix vs. Aimmugen: hepatitis A vaccine (females, age 22-29; follow-up until 12 months)**						
HPV-063: Cervarix vs. Aimmugen (f, 22-29; 12)	18	375	18	377	3.0%	1.01 [0.53, 1.90]
**Subtotal (95% CI)**		**375**		**377**	**3.0%**	**1.01 [0.53, 1.90]**
Total events	18		18			
Heterogeneity: Not applicable						
Test for overall effect: Z = 0.02 (P = 0.99)						
**11.24.6 New onset skin and subcutaneous tissue disorders ('medically significant conditions'): Cervarix vs. Engerix: hepatitis B vaccine (females and males, age 9-15; follow-up until 12 months)**						
HPV-030: Cervarix vs. Engerix (f, 9-15; 12)	1	247	1	246	0.2%	1.00 [0.06, 15.83]
**Subtotal (95% CI)**		**247**		**246**	**0.2%**	**1.00 [0.06, 15.83]**
Total events	1		1			
Heterogeneity: Not applicable						
Test for overall effect: Z = 0.00 (P = 1.00)						
**11.24.7 New onset skin and subcutaneous tissue disorders ('medically significant conditions'): Cervarix vs. Engerix: hepatitis B vaccine (females, age 26-46; follow-up until 12 months)**						
HPV-069: Cervarix vs. Engerix (f, 26-46; 12)	1	606	1	606	0.2%	1.00 [0.06, 15.95]
**Subtotal (95% CI)**		**606**		**606**	**0.2%**	**1.00 [0.06, 15.95]**
Total events	1		1			
Heterogeneity: Not applicable						
Test for overall effect: Z = 0.00 (P = 1.00)						
**11.24.8 New onset skin and subcutaneous tissue disorders ('medically significant conditions'): Cervarix vs. Havrix: hepatitis A vaccine (females, age 9-15; follow-up until 12 months)**						
HPV-013: Cervarix vs. Havrix (f, 10-14; 12)	23	1035	27	1032	4.0%	0.85 [0.49, 1.47]
HPV-033: Cervarix vs. Havrix (f, 9-15; 7)	4	160	1	161	0.3%	4.03 [0.45, 35.62]
**Subtotal (95% CI)**		**1195**		**1193**	**4.2%**	**1.27 [0.33, 4.86]**
Total events	27		28			
Heterogeneity: $Tau^2 = 0.55$; $Chi^2 = 1.84$, df = 1 (P = 0.18); $I^2 = 46\%$						
Test for overall effect: Z = 0.35 (P = 0.72)						
**11.24.9 New onset skin and subcutaneous tissue disorders ('medically significant conditions'): Cervarix vs. Havrix: hepatitis A vaccine (females, age 15-25; follow-up until 48 months)**						
HPV-008: Cervarix vs. Havrix (f, 15-25; 48)	204	9319	205	9325	16.3%	1.00 [0.82, 1.21]
**Subtotal (95% CI)**		**9319**		**9325**	**16.3%**	**1.00 [0.82, 1.21]**
Total events	204		205			
Heterogeneity: Not applicable						
Test for overall effect: Z = 0.04 (P = 0.97)						
**11.24.10 New onset skin and subcutaneous tissue disorders ('medically significant conditions'): Cervarix vs. Twinrix: hepatitis A and B vaccine (females, age 8-15; follow-up until 12 months)**						
HPV-029: Cervarix vs. Twinrix (f, 8-15; 12)	1	270	3	271	0.3%	0.33 [0.04, 3.20]
**Subtotal (95% CI)**		**270**		**271**	**0.3%**	**0.33 [0.04, 3.20]**
Total events	1		3			
Heterogeneity: Not applicable						
Test for overall effect: Z = 0.95 (P = 0.34)						
**11.24.11 New onset skin and subcutaneous tissue disorders ('new medical history'): Gardasil vs. carrier solution (females and males, age 9-16; follow-up until 18 months)**						
V501-018: Gardasil vs. carrier solu. (f&m,9-16;18)	45	1184	23	597	4.8%	0.99 [0.60, 1.61]
**Subtotal (95% CI)**		**1184**		**597**	**4.8%**	**0.99 [0.60, 1.61]**
Total events	45		23			
Heterogeneity: Not applicable						
Test for overall effect: Z = 0.05 (P = 0.96)						
**11.24.12 New onset skin and subcutaneous tissue disorders ('new medical history'): Gardasil vs. AAHS (females, age 15-26; follow-up until 45 months)**						
V501-013: Gardasil vs. AAHS (f, 16-24; 45)	200	2723	210	2732	16.7%	0.96 [0.79, 1.15]
V501-015: Gardasil vs. AAHS (f, 15-26; 36)	410	6087	452	6080	21.3%	0.91 [0.80, 1.03]
**Subtotal (95% CI)**		**8810**		**8812**	**38.0%**	**0.92 [0.83, 1.02]**
Total events	610		662			
Heterogeneity: $Tau^2 = 0.00$; $Chi^2 = 0.21$, df = 1 (P = 0.65); $I^2 = 0\%$						
Test for overall effect: Z = 1.51 (P = 0.13)						
**11.24.13 New onset skin and subcutaneous tissue disorders ('new medical history'): Gardasil vs. AAHS (females, age 21-46; from 0-7 months)**						
V501-019: Gardasil vs. AAHS (f, 21-46; 48)	60	1911	33	1908	6.2%	1.82 [1.19, 2.76]
**Subtotal (95% CI)**		**1911**		**1908**	**6.2%**	**1.82 [1.19, 2.76]**
Total events	60		33			
Heterogeneity: Not applicable						
Test for overall effect: Z = 2.78 (P = 0.005)						
**11.24.14 New onset skin and subcutaneous tissue disorders ('new medical history'): Gardasil vs. AAHS (females, age 21-46; from 7-48 months)**						
V501-019: Gardasil vs. AAHS (f, 21-46; 48)	87	1911	86	1908	0.0%	1.01 [0.75, 1.35]
**Subtotal (95% CI)**		**0**		**0**		**Not estimable**
Total events	0		0			
Heterogeneity: Not applicable						
Test for overall effect: Not applicable						
**11.24.15 New onset skin and subcutaneous tissue disorders ('new medical history'): Gardasil vs. AAHS (males, age 15-27; from 0-7 months)**						
V501-020: Gardasil vs. AAHS (m, 15-27; 36)	43	2032	45	2032	6.3%	0.96 [0.63, 1.44]
**Subtotal (95% CI)**		**2032**		**2032**	**6.3%**	**0.96 [0.63, 1.44]**
Total events	43		45			
Heterogeneity: Not applicable						
Test for overall effect: Z = 0.22 (P = 0.83)						
**11.24.16 New onset skin and subcutaneous tissue disorders ('new medical history'): Gardasil vs. AAHS (males, age 15-27; from 7-36 months)**						
V501-020: Gardasil vs. AAHS (m, 15-27; 36)	69	2032	67	2033	0.0%	1.03 [0.74, 1.43]
**Subtotal (95% CI)**		**0**		**0**		**Not estimable**
Total events	0		0			
Heterogeneity: Not applicable						
Test for overall effect: Not applicable						
**11.24.17 New onset skin and subcutaneous tissue disorders ('new medical history'): HPV 16 vaccine vs. AAHS (females, age 16-25; from 0-7 months)**						
V501-005: HPV 16 vaccine vs. AAHS (f, 16-25; 48)	70	1204	62	1205	8.7%	1.13 [0.81, 1.58]
**Subtotal (95% CI)**		**1204**		**1205**	**8.7%**	**1.13 [0.81, 1.58]**
Total events	70		62			
Heterogeneity: Not applicable						
Test for overall effect: Z = 0.72 (P = 0.47)						
**11.24.18 New onset skin and subcutaneous tissue disorders ('new medical history'): HPV 16 vaccine vs. AAHS (females, age 16-25; from 7-48 months)**						
V501-005: HPV 16 vaccine vs. AAHS (f, 16-25; 48)	94	1204	103	1205	0.0%	0.91 [0.70, 1.19]
**Subtotal (95% CI)**		**0**		**0**		**Not estimable**
Total events	0		0			
Heterogeneity: Not applicable						
Test for overall effect: Not applicable						
**11.24.19 New onset skin and subcutaneous tissue disorders ('new medical history'): Gardasil 9 vs. saline placebo (females, age 12-26; follow-up until 7 months)**						
V503-006: Gardasil 9 vs. saline (f, 12-26;7)	9	618	8	306	1.5%	0.56 [0.22, 1.43]
**Subtotal (95% CI)**		**618**		**306**	**1.5%**	**0.56 [0.22, 1.43]**
Total events	9		8			
Heterogeneity: Not applicable						
Test for overall effect: Z = 1.22 (P = 0.22)						
**Total (95% CI)**		**31502**		**30529**	**100.0%**	**0.99 [0.88, 1.12]**
Total events	1173		1176			
Heterogeneity: $Tau^2 = 0.01$; $Chi^2 = 24.02$, df = 18 (P = 0.15); $I^2 = 25\%$						
Test for overall effect: Z = 0.10 (P = 0.92)						
Test for subgroup differences: $Chi^2 = 16.07$, df = 15 (P = 0.38), $I^2 = 6.6\%$						

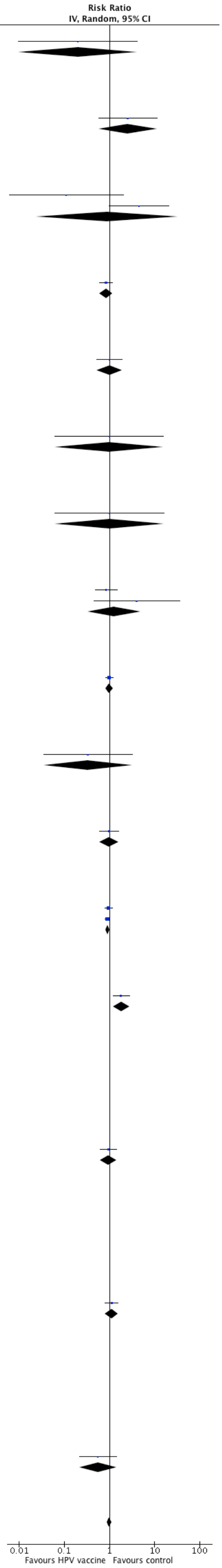

*11.24. Risk ratio for 'medically significant conditions’ (GlaxoSmithKline): 0.96 [0.79, 1.18]; risk ratio for ‘new medical history’ (Merck Sharp & Dohme): 1.02 [0.86, 1.20]; risk ratio for the follow-up periods for the trials V501-005, V501-019 and V501-020: 0.98 [0.82, 1.16]. The trials V501-005, V501-019 and V501-020 split the reporting of new onset diseases into the vaccination period and the follow-up period. To avoid double counting of participants in the total risk ratio estimate, we only included the new onset diseases reported in the vaccination period for the trials V501-005, V501-019 and V501-020.

11.25. New onset diseases ('medically significant conditions' and 'new medical history'*) reported within the MedDRA system organ class ‘social circumstances (10041244)': intention to treat analysis

Study or Subgroup	HPV vaccine Events	HPV vaccine Total	Control Events	Control Total	Weight	Risk Ratio IV, Random, 95% CI
**11.25.1 New onset social circumstances ('medically significant conditions'): Cervarix vs. Al(OH)3 (females, age 24-72; follow-up until 36 months)**						
HPV-015: Cervarix vs. Al(OH)3 (f, 24-72; 36)	2	2882	1	2871	3.2%	1.99 [0.18, 21.96]
**Subtotal (95% CI)**		**2882**		**2871**	**3.2%**	**1.99 [0.18, 21.96]**
Total events	2		1			
Heterogeneity: Not applicable						
Test for overall effect: Z = 0.56 (P = 0.57)						
**11.25.2 New onset social circumstances ('medically significant conditions'): Cervarix vs. Havrix: hepatitis A vaccine (females, age 15-25; follow-up until 48 months)**						
HPV-008: Cervarix vs. Havrix (f, 15-25; 48)	5	9319	8	9325	14.9%	0.63 [0.20, 1.91]
**Subtotal (95% CI)**		**9319**		**9325**	**14.9%**	**0.63 [0.20, 1.91]**
Total events	5		8			
Heterogeneity: Not applicable						
Test for overall effect: Z = 0.82 (P = 0.41)						
**11.25.3 New onset social circumstances ('new medical history'): Gardasil vs. carrier solution (females and males, age 9-16; follow-up until 18 months)**						
V501-018: Gardasil vs. carrier solu. (f&m,9-16;18)	5	1184	5	597	12.2%	0.50 [0.15, 1.73]
**Subtotal (95% CI)**		**1184**		**597**	**12.2%**	**0.50 [0.15, 1.73]**
Total events	5		5			
Heterogeneity: Not applicable						
Test for overall effect: Z = 1.09 (P = 0.28)						
**11.25.4 New onset social circumstances ('new medical history'): Gardasil vs. AAHS (females, age 15-26; follow-up until 45 months)**						
V501-013: Gardasil vs. AAHS (f, 16-24; 45)	17	2723	14	2732	37.4%	1.22 [0.60, 2.47]
V501-015: Gardasil vs. AAHS (f, 15-26; 36)	7	6087	10	6080	20.0%	0.70 [0.27, 1.84]
**Subtotal (95% CI)**		**8810**		**8812**	**57.3%**	**1.00 [0.57, 1.77]**
Total events	24		24			
Heterogeneity: Tau² = 0.00; Chi² = 0.83, df = 1 (P = 0.36); I² = 0%						
Test for overall effect: Z = 0.01 (P = 0.99)						
**11.25.5 New onset social circumstances ('new medical history'): Gardasil vs. AAHS (females, age 21-46; from 0-7 months)**						
V501-019: Gardasil vs. AAHS (f, 21-46; 48)	6	1911	1	1908	4.2%	5.99 [0.72, 49.71]
**Subtotal (95% CI)**		**1911**		**1908**	**4.2%**	**5.99 [0.72, 49.71]**
Total events	6		1			
Heterogeneity: Not applicable						
Test for overall effect: Z = 1.66 (P = 0.10)						
**11.25.6 New onset social circumstances ('new medical history'): Gardasil vs. AAHS (females, age 21-46; from 7-48 months)**						
V501-019: Gardasil vs. AAHS (f, 21-46; 48)	11	1911	13	1908	0.0%	0.84 [0.38, 1.88]
**Subtotal (95% CI)**		**0**		**0**		**Not estimable**
Total events	0		0			
Heterogeneity: Not applicable						
Test for overall effect: Not applicable						
**11.25.7 New onset social circumstances ('new medical history'): Gardasil vs. AAHS (males, age 15-27; from 0-7 months)**						
V501-020: Gardasil vs. AAHS (m, 15-27; 36)	3	2032	2	2032	5.8%	1.50 [0.25, 8.97]
**Subtotal (95% CI)**		**2032**		**2032**	**5.8%**	**1.50 [0.25, 8.97]**
Total events	3		2			
Heterogeneity: Not applicable						
Test for overall effect: Z = 0.44 (P = 0.66)						
**11.25.8 New onset social circumstances ('new medical history'): Gardasil vs. AAHS (males, age 15-27; from 7-36 months)**						
V501-020: Gardasil vs. AAHS (m, 15-27; 36)	0	2032	2	2033	0.0%	0.20 [0.01, 4.17]
**Subtotal (95% CI)**		**0**		**0**		**Not estimable**
Total events	0		0			
Heterogeneity: Not applicable						
Test for overall effect: Not applicable						
**11.25.9 New onset social circumstances ('new medical history'): Gardasil 9 vs. saline placebo (females, age 12-26; follow-up until 7 months)**						
V503-006: Gardasil 9 vs. saline (f, 12-26;7)	1	618	1	306	2.4%	0.50 [0.03, 7.89]
**Subtotal (95% CI)**		**618**		**306**	**2.4%**	**0.50 [0.03, 7.89]**
Total events	1		1			
Heterogeneity: Not applicable						
Test for overall effect: Z = 0.50 (P = 0.62)						
**Total (95% CI)**		**26756**		**25851**	**100.0%**	**0.95 [0.62, 1.47]**
Total events	46		42			
Heterogeneity: Tau² = 0.00; Chi² = 6.15, df = 7 (P = 0.52); I² = 0%						
Test for overall effect: Z = 0.22 (P = 0.83)						
Test for subgroup differences: Chi² = 5.32, df = 6 (P = 0.50), I² = 0%						

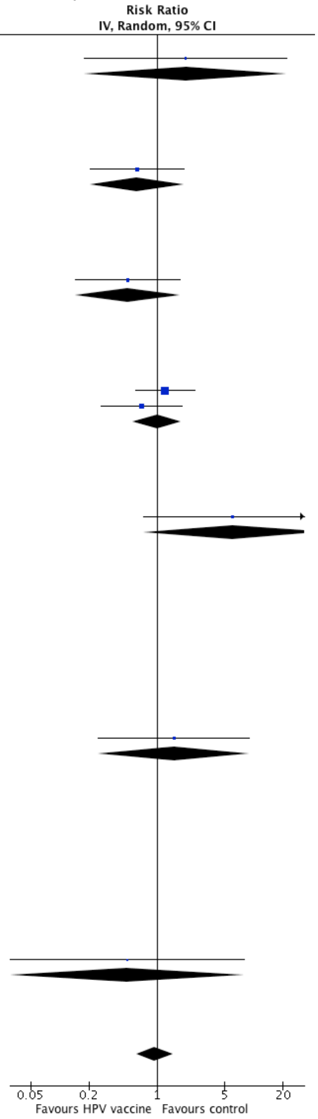

*11.25. Risk ratio for 'medically significant conditions’ (GlaxoSmithKline): 0.77 [0.28, 2.12]; risk ratio for ‘new medical history’ (Merck Sharp & Dohme): 1.00 [0.61, 1.64]; risk ratio for the follow-up periods for the trials V501-019 and V501-020: 0.77 [0.35, 1.67]. The trials V501-019 and V501-020 split the reporting of new onset diseases into the vaccination period and the follow-up period. To avoid double counting of participants in the total risk ratio estimate, we only included the new onset diseases reported in the vaccination period for the trials V501-019 and V501-020.

11.26. New onset diseases ('medically significant conditions' and 'new medical history'*) reported within the MedDRA system organ class ‘surgical and medical procedures (10042613)': intention to treat analysis

Study or Subgroup	HPV vaccine Events	HPV vaccine Total	Control Events	Control Total	Weight	Risk Ratio IV, Random, 95% CI
**11.26.1 New onset surgical and medical procedures ('medically significant conditions'): Cervarix vs. Al(OH)3 (females, age 24-72; follow-up until 36 months)**						
HPV-015: Cervarix vs. Al(OH)3 (f, 24-72; 36)	32	2882	28	2871	2.0%	1.14 [0.69, 1.89]
**Subtotal (95% CI)**		**2882**		**2871**	**2.0%**	**1.14 [0.69, 1.89]**
Total events	32		28			
Heterogeneity: Not applicable						
Test for overall effect: Z = 0.50 (P = 0.61)						
**11.26.2 New onset surgical and medical procedures ('medically significant conditions'): Cervarix vs. Havrix: hepatitis A vaccine (females, age 10-14; follow-up until 12 months)**						
HPV-013: Cervarix vs. Havrix (f, 10-14; 12)	5	1035	6	1032	0.4%	0.83 [0.25, 2.71]
**Subtotal (95% CI)**		**1035**		**1032**	**0.4%**	**0.83 [0.25, 2.71]**
Total events	5		6			
Heterogeneity: Not applicable						
Test for overall effect: Z = 0.31 (P = 0.76)						
**11.26.3 New onset surgical and medical procedures ('medically significant conditions'): Cervarix vs. Havrix: hepatitis A vaccine (females, age 15-25; follow-up until 48 months)**						
HPV-008: Cervarix vs. Havrix (f, 15-25; 48)	35	9319	40	9325	2.5%	0.88 [0.56, 1.38]
**Subtotal (95% CI)**		**9319**		**9325**	**2.5%**	**0.88 [0.56, 1.38]**
Total events	35		40			
Heterogeneity: Not applicable						
Test for overall effect: Z = 0.58 (P = 0.57)						
**11.26.4 New onset surgical and medical procedures ('new medical history'): Gardasil vs. carrier solution (females and males, age 9-16; follow-up until 18 months)**						
V501-018: Gardasil vs. carrier solu. (f&m,9-16;18)	19	1184	9	597	0.8%	1.06 [0.48, 2.34]
**Subtotal (95% CI)**		**1184**		**597**	**0.8%**	**1.06 [0.48, 2.34]**
Total events	19		9			
Heterogeneity: Not applicable						
Test for overall effect: Z = 0.16 (P = 0.88)						
**11.26.5 New onset surgical and medical procedures ('new medical history'): Gardasil vs. AAHS (females, age 15-26; follow-up until 45 months)**						
V501-013: Gardasil vs. AAHS (f, 16-24; 45)	435	2723	456	2732	35.7%	0.96 [0.85, 1.08]
V501-015: Gardasil vs. AAHS (f, 15-26; 36)	609	6087	633	6080	46.4%	0.96 [0.86, 1.07]
**Subtotal (95% CI)**		**8810**		**8812**	**82.2%**	**0.96 [0.89, 1.04]**
Total events	1044		1089			
Heterogeneity: Tau² = 0.00; Chi² = 0.00, df = 1 (P = 0.96); I² = 0%						
Test for overall effect: Z = 1.03 (P = 0.30)						
**11.26.6 New onset surgical and medical procedures ('new medical history'): Gardasil vs. AAHS (females, age 21-46; from 0-7 months)**						
V501-019: Gardasil vs. AAHS (f, 21-46; 48)	92	1911	79	1908	6.0%	1.16 [0.87, 1.56]
**Subtotal (95% CI)**		**1911**		**1908**	**6.0%**	**1.16 [0.87, 1.56]**
Total events	92		79			
Heterogeneity: Not applicable						
Test for overall effect: Z = 1.01 (P = 0.31)						
**11.26.7 New onset surgical and medical procedures ('new medical history'): Gardasil vs. AAHS (females, age 21-46; from 7-48 months)**						
V501-019: Gardasil vs. AAHS (f, 21-46; 48)	252	1911	266	1908	0.0%	0.95 [0.81, 1.11]
**Subtotal (95% CI)**		**0**		**0**		**Not estimable**
Total events	0		0			
Heterogeneity: Not applicable						
Test for overall effect: Not applicable						
**11.26.8 New onset surgical and medical procedures ('new medical history'): Gardasil vs. AAHS (males, age 15-27; from 0-7 months)**						
V501-020: Gardasil vs. AAHS (m, 15-27; 36)	38	2032	27	2032	2.2%	1.41 [0.86, 2.30]
**Subtotal (95% CI)**		**2032**		**2032**	**2.2%**	**1.41 [0.86, 2.30]**
Total events	38		27			
Heterogeneity: Not applicable						
Test for overall effect: Z = 1.37 (P = 0.17)						
**11.26.9 New onset surgical and medical procedures ('new medical history'): Gardasil vs. AAHS (males, age 15-27; from 7-36 months)**						
V501-020: Gardasil vs. AAHS (m, 15-27; 36)	55	2032	36	2033	0.0%	1.53 [1.01, 2.32]
**Subtotal (95% CI)**		**0**		**0**		**Not estimable**
Total events	0		0			
Heterogeneity: Not applicable						
Test for overall effect: Not applicable						
**11.26.10 New onset surgical and medical procedures ('new medical history'): HPV 16 vaccine vs. AAHS (females, age 16-25; from 0-7 months)**						
V501-005: HPV 16 vaccine vs. AAHS (f, 16-25; 48)	40	1204	51	1205	3.1%	0.78 [0.52, 1.18]
**Subtotal (95% CI)**		**1204**		**1205**	**3.1%**	**0.78 [0.52, 1.18]**
Total events	40		51			
Heterogeneity: Not applicable						
Test for overall effect: Z = 1.17 (P = 0.24)						
**11.26.11 New onset surgical and medical procedures ('new medical history'): HPV 16 vaccine vs. AAHS (females, age 16-25; from 7-48 months)**						
V501-005: HPV 16 vaccine vs. AAHS (f, 16-25; 48)	164	1204	148	1205	0.0%	1.11 [0.90, 1.36]
**Subtotal (95% CI)**		**0**		**0**		**Not estimable**
Total events	0		0			
Heterogeneity: Not applicable						
Test for overall effect: Not applicable						
**11.26.12 New onset surgical and medical procedures ('new medical history'): Gardasil 9 vs. saline placebo (females, age 12-26; follow-up until 7 months)**						
V503-006: Gardasil 9 vs. saline (f, 12-26;7)	13	618	11	306	0.8%	0.59 [0.27, 1.29]
**Subtotal (95% CI)**		**618**		**306**	**0.8%**	**0.59 [0.27, 1.29]**
Total events	13		11			
Heterogeneity: Not applicable						
Test for overall effect: Z = 1.33 (P = 0.18)						
**Total (95% CI)**		**28995**		**28088**	**100.0%**	**0.97 [0.90, 1.04]**
Total events	1318		1340			
Heterogeneity: Tau² = 0.00; Chi² = 7.07, df = 9 (P = 0.63); I² = 0%						
Test for overall effect: Z = 0.84 (P = 0.40)						
Test for subgroup differences: Chi² = 7.07, df = 8 (P = 0.53), I² = 0%						

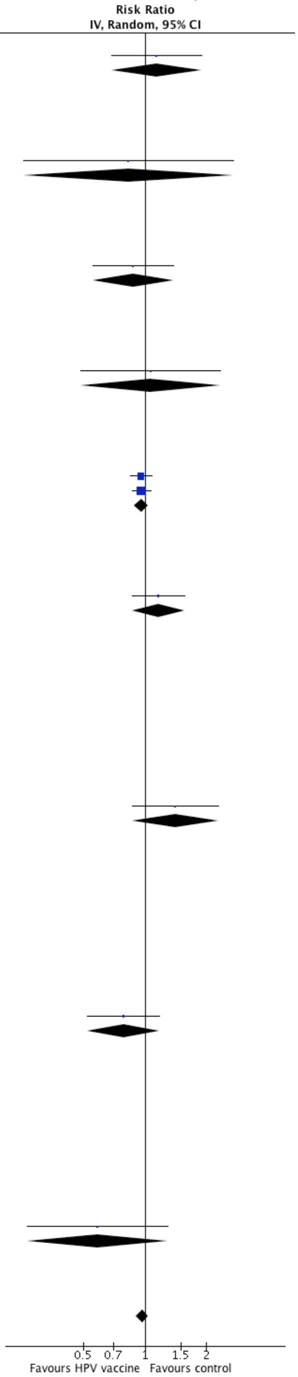

*11.26. Risk ratio for 'medically significant conditions’ (GlaxoSmithKline): 0.99 [0.71, 1.36]; risk ratio for ‘new medical history’ (Merck Sharp & Dohme): 0.97 [0.89, 1.06]; risk ratio for the follow-up periods for the trials V501-005, V501-019 and V501-020: 1.10 [0.88, 1.36]. The trials V501-005, V501-019 and V501-020 split the reporting of new onset diseases into the vaccination period and the follow-up period. To avoid double counting of participants in the total risk ratio estimate, we only included the new onset diseases reported in the vaccination period for the trials V501-005, V501-019 and V501-020.

11.27. New onset diseases ('medically significant conditions' and 'new medical history'*) reported within the MedDRA system organ class 'vascular disorders (10047065)': intention to treat analysis

Study or Subgroup	HPV vaccine Events	HPV vaccine Total	Control Events	Control Total	Weight	Risk Ratio IV, Random, 95% CI
**11.27.1 New onset vascular disorders ('medically significant conditions'): Cervarix vs. Al(OH)3 (females, age 24-72; follow-up until 36 months)**						
HPV-015: Cervarix vs. Al(OH)3 (f, 24-72; 36)	65	2882	95	2871	30.4%	0.68 [0.50, 0.93]
**Subtotal (95% CI)**		**2882**		**2871**	**30.4%**	**0.68 [0.50, 0.93]**
Total events	65		95			
Heterogeneity: Not applicable						
Test for overall effect: Z = 2.41 (P = 0.02)						
**11.27.2 New onset vascular disorders ('medically significant conditions'): Cervarix vs. Aimmugen: hepatitis A vaccine (females, age 22-29; follow-up until 12 months)**						
HPV-063: Cervarix vs. Aimmugen (f, 22-29; 12)	1	375	1	377	0.4%	1.01 [0.06, 16.01]
**Subtotal (95% CI)**		**375**		**377**	**0.4%**	**1.01 [0.06, 16.01]**
Total events	1		1			
Heterogeneity: Not applicable						
Test for overall effect: Z = 0.00 (P = 1.00)						
**11.27.3 New onset vascular disorders ('medically significant conditions'): Cervarix vs. Havrix: hepatitis A vaccine (females, age 10-14; follow-up until 12 months)**						
HPV-013: Cervarix vs. Havrix (f, 10-14; 12)	4	1035	2	1032	1.0%	1.99 [0.37, 10.86]
**Subtotal (95% CI)**		**1035**		**1032**	**1.0%**	**1.99 [0.37, 10.86]**
Total events	4		2			
Heterogeneity: Not applicable						
Test for overall effect: Z = 0.80 (P = 0.42)						
**11.27.4 New onset vascular disorders ('medically significant conditions'): Cervarix vs. Havrix: hepatitis A vaccine (females, age 15-25; follow-up until 48 months)**						
HPV-008: Cervarix vs. Havrix (f, 15-25; 48)	41	9319	39	9325	15.4%	1.05 [0.68, 1.63]
**Subtotal (95% CI)**		**9319**		**9325**	**15.4%**	**1.05 [0.68, 1.63]**
Total events	41		39			
Heterogeneity: Not applicable						
Test for overall effect: Z = 0.23 (P = 0.82)						
**11.27.5 New onset vascular disorders ('medically significant conditions'): Cervarix vs. Twinrix: hepatitis A and B vaccine (females, age 8-15; follow-up until 12 months)**						
HPV-029: Cervarix vs. Twinrix (f, 8-15; 12)	0	270	1	271	0.3%	0.33 [0.01, 8.18]
**Subtotal (95% CI)**		**270**		**271**	**0.3%**	**0.33 [0.01, 8.18]**
Total events	0		1			
Heterogeneity: Not applicable						
Test for overall effect: Z = 0.67 (P = 0.50)						
**11.27.6 New onset vascular disorders ('new medical history'): Gardasil vs. carrier solution (females and males, age 9-16; follow-up until 18 months)**						
V501-018: Gardasil vs. carrier solu. (f&m,9-16;18)	1	1184	2	597	0.5%	0.25 [0.02, 2.77]
**Subtotal (95% CI)**		**1184**		**597**	**0.5%**	**0.25 [0.02, 2.77]**
Total events	1		2			
Heterogeneity: Not applicable						
Test for overall effect: Z = 1.13 (P = 0.26)						
**11.27.7 New onset vascular disorders ('new medical history'): Gardasil vs. AAHS (females, age 15-26; follow-up until 45 months)**						
V501-013: Gardasil vs. AAHS (f, 16-24; 45)	36	2723	34	2732	13.6%	1.06 [0.67, 1.69]
V501-015: Gardasil vs. AAHS (f, 15-26; 36)	65	6087	85	6080	28.6%	0.76 [0.55, 1.05]
**Subtotal (95% CI)**		**8810**		**8812**	**42.2%**	**0.86 [0.63, 1.18]**
Total events	101		119			
Heterogeneity: $Tau^2 = 0.01$; $Chi^2 = 1.31$, df = 1 (P = 0.25); $I^2 = 23\%$						
Test for overall effect: Z = 0.94 (P = 0.35)						
**11.27.8 New onset vascular disorders ('new medical history'): Gardasil vs. AAHS (females, age 21-46; from 0-7 months)**						
V501-019: Gardasil vs. AAHS (f, 21-46; 48)	9	1911	22	1908	4.9%	0.41 [0.19, 0.88]
**Subtotal (95% CI)**		**1911**		**1908**	**4.9%**	**0.41 [0.19, 0.88]**
Total events	9		22			
Heterogeneity: Not applicable						
Test for overall effect: Z = 2.27 (P = 0.02)						
**11.27.9 New onset vascular disorders ('new medical history'): Gardasil vs. AAHS (females, age 21-46; from 7-48 months)**						
V501-019: Gardasil vs. AAHS (f, 21-46; 48)	45	1911	39	1908	0.0%	1.15 [0.75, 1.76]
**Subtotal (95% CI)**		**0**		**0**		**Not estimable**
Total events	0		0			
Heterogeneity: Not applicable						
Test for overall effect: Not applicable						
**11.27.10 New onset vascular disorders ('new medical history'): Gardasil vs. AAHS (males, age 15-27; from 0-7 months)**						
V501-020: Gardasil vs. AAHS (m, 15-27; 36)	2	2032	3	2032	0.9%	0.67 [0.11, 3.99]
**Subtotal (95% CI)**		**2032**		**2032**	**0.9%**	**0.67 [0.11, 3.99]**
Total events	2		3			
Heterogeneity: Not applicable						
Test for overall effect: Z = 0.44 (P = 0.66)						
**11.27.11 New onset vascular disorders ('new medical history'): Gardasil vs. AAHS (males, age 15-27; from 7-36 months)**						
V501-020: Gardasil vs. AAHS (m, 15-27; 36)	7	2032	12	2033	0.0%	0.58 [0.23, 1.48]
**Subtotal (95% CI)**		**0**		**0**		**Not estimable**
Total events	0		0			
Heterogeneity: Not applicable						
Test for overall effect: Not applicable						
**11.27.12 New onset vascular disorders ('new medical history'): HPV 16 vaccine vs. AAHS (females, age 16-25; from 7-48 months)**						
V501-005: HPV 16 vaccine vs. AAHS (f, 16-25; 48)	10	1204	10	1205	3.9%	1.00 [0.42, 2.40]
**Subtotal (95% CI)**		**1204**		**1205**	**3.9%**	**1.00 [0.42, 2.40]**
Total events	10		10			
Heterogeneity: Not applicable						
Test for overall effect: Z = 0.00 (P = 1.00)						
**Total (95% CI)**		**29022**		**28430**	**100.0%**	**0.80 [0.67, 0.94]**
Total events	234		294			
Heterogeneity: $Tau^2 = 0.00$; $Chi^2 = 9.54$, df = 10 (P = 0.48); $I^2 = 0\%$						
Test for overall effect: Z = 2.61 (P = 0.009)						
Test for subgroup differences: $Chi^2 = 8.24$, df = 9 (P = 0.51), $I^2 = 0\%$						

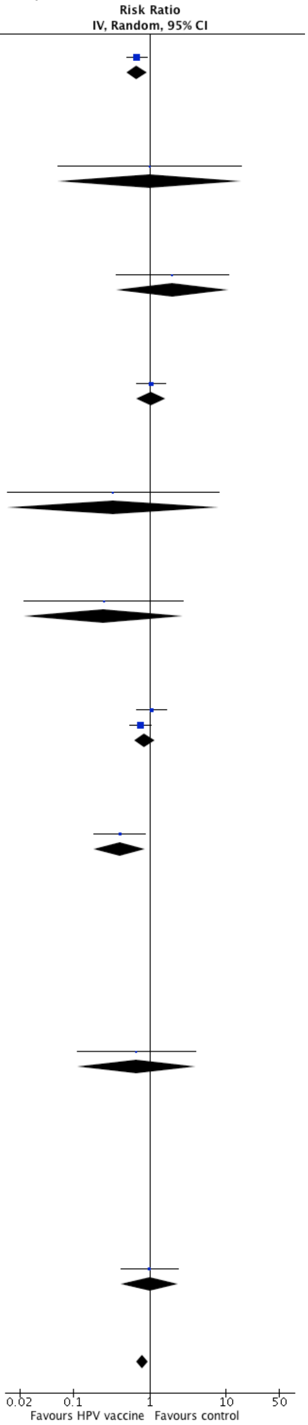

*11.27. Risk ratio for 'medically significant conditions' (GlaxoSmithKline): 0.80 [0.63, 1.03]; risk ratio for 'new medical history' (Merck Sharp & Dohme): 0.78 [0.60, 1.03]; risk ratio for the follow-up periods for the trials V501-019 and V501-020: 0.93 [0.51, 1.73]. The trials V501-019 and V501-020 split the reporting of new onset diseases into the vaccination period and the follow-up period. To avoid double counting of participants in the total risk ratio estimate, we only included the new onset diseases reported in the vaccination period for the trials V501-019 and V501-020.

11.28. Most common new onset diseases (‘medically significant conditions’*) - ‘gynaecological chlamydia infection’: intention to treat analysis

Study or Subgroup	HPV vaccine Events	HPV vaccine Total	Control Events	Control Total	Weight	Risk Ratio IV, Random, 95% CI
**11.28.1 New onset gynaecological chlamydia infection ('medically significant conditions'): Cervarix vs. Havrix: hepatitis A vaccine (females, age 15-25; follow-up until 48 months)**						
HPV-008: Cervarix vs. Havrix (f, 15-25; 48)	1035	9319	1085	9325	74.2%	0.95 [0.88, 1.03]
**Subtotal (95% CI)**		**9319**		**9325**	**74.2%**	**0.95 [0.88, 1.03]**
Total events	1035		1085			
Heterogeneity: Not applicable						
Test for overall effect: Z = 1.14 (P = 0.26)						
**11.28.2 New onset gynaecological chlamydia infection ('new medical history'): Gardasil vs. AAHS (females, age 15-26; follow-up until 45 months)**						
V501-013: Gardasil vs. AAHS (f, 16-24; 45)	51	2723	58	2732	3.4%	0.88 [0.61, 1.28]
V501-015: Gardasil vs. AAHS (f, 15-26; 36)	296	6087	342	6080	20.8%	0.86 [0.74, 1.01]
**Subtotal (95% CI)**		**8810**		**8812**	**24.2%**	**0.87 [0.75, 1.00]**
Total events	347		400			
Heterogeneity: $Tau^2 = 0.00$; $Chi^2 = 0.01$, df = 1 (P = 0.92); $I^2 = 0\%$						
Test for overall effect: Z = 1.99 (P = 0.05)						
**11.28.3 New onset gynaecological chlamydia infection ('new medical history'): Gardasil vs. AAHS (females, age 21-46; from 7-48 months)**						
V501-019: Gardasil vs. AAHS (f, 21-46; 48)	2	1911	6	1908	0.2%	0.33 [0.07, 1.65]
**Subtotal (95% CI)**		**1911**		**1908**	**0.2%**	**0.33 [0.07, 1.65]**
Total events	2		6			
Heterogeneity: Not applicable						
Test for overall effect: Z = 1.35 (P = 0.18)						
**11.28.4 New onset gynaecological chlamydia infection ('new medical history'): HPV 16 vaccine vs. AAHS (females, age 16-25; from 0-7 months)**						
V501-005: HPV 16 vaccine vs. AAHS (f, 16-25; 48)	23	1204	21	1205	1.4%	1.10 [0.61, 1.97]
**Subtotal (95% CI)**		**1204**		**1205**	**1.4%**	**1.10 [0.61, 1.97]**
Total events	23		21			
Heterogeneity: Not applicable						
Test for overall effect: Z = 0.31 (P = 0.76)						
**11.28.5 New onset gynaecological chlamydia infection ('new medical history'): HPV 16 vaccine vs. AAHS (females, age 16-25; from 7-48 months)**						
V501-005: HPV 16 vaccine vs. AAHS (f, 16-25; 48)	32	1204	23	1205	0.0%	1.39 [0.82, 2.37]
**Subtotal (95% CI)**		**0**		**0**		**Not estimable**
Total events	0		0			
Heterogeneity: Not applicable						
Test for overall effect: Not applicable						
**11.28.6 New onset gynaecological chlamydia infection ('new medical history'): Gardasil 9 vs. saline placebo (females, age 12-26; follow-up until 7 months)**						
V503-006: Gardasil 9 vs. saline (f, 12-26;7)	2	618	0	306	0.1%	2.48 [0.12, 51.49]
**Subtotal (95% CI)**		**618**		**306**	**0.1%**	**2.48 [0.12, 51.49]**
Total events	2		0			
Heterogeneity: Not applicable						
Test for overall effect: Z = 0.59 (P = 0.56)						
**Total (95% CI)**		**21862**		**21556**	**100.0%**	**0.93 [0.87, 1.00]**
Total events	1409		1512			
Heterogeneity: $Tau^2 = 0.00$; $Chi^2 = 3.66$, df = 5 (P = 0.60); $I^2 = 0\%$						
Test for overall effect: Z = 1.97 (P = 0.05)						
Test for subgroup differences: $Chi^2 = 3.65$, df = 4 (P = 0.46), $I^2 = 0\%$						

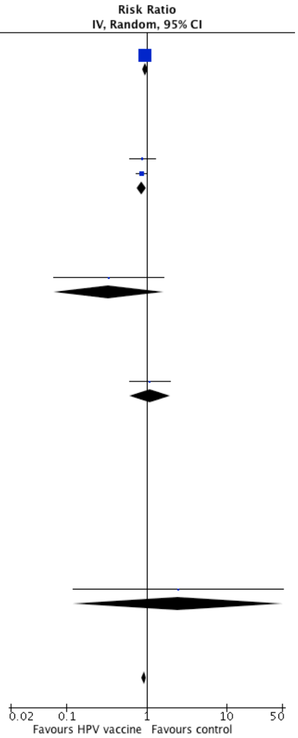

*11.28. Risk ratio for 'medically significant conditions’ (GlaxoSmithKline): 0.95 [0.88, 1.03]; risk ratio for ‘new medical history’ (Merck Sharp & Dohme): 0.87 [0.76, 1.00]; risk ratio for the follow-up period for the trial V501-005: 1.39 [0.82, 2.37]. The trial V501-005 split the reporting of new onset diseases into the vaccination period and the follow-up period. To avoid double counting of participants in the total risk ratio estimate, we only included the new onset diseases reported in the vaccination period for the trial V501-005.

11.29. Most common new onset diseases (‘medically significant conditions’*) - ‘depression’: intention to treat analysis

Study or Subgroup	HPV vaccine Events	HPV vaccine Total	Control Events	Control Total	Weight	Risk Ratio IV, Random, 95% CI
**11.29.1 New onset depression ('medically significant conditions'): Cervarix vs. Al(OH)3 (females, age 24-72; follow-up until 36 months)**						
HPV-015: Cervarix vs. Al(OH)3 (f, 24-72; 36)	43	2882	44	2871	10.0%	0.97 [0.64, 1.48]
**Subtotal (95% CI)**		**2882**		**2871**	**10.0%**	**0.97 [0.64, 1.48]**
Total events	43		44			
Heterogeneity: Not applicable						
Test for overall effect: Z = 0.13 (P = 0.90)						
**11.29.2 New onset depression ('medically significant conditions'): Cervarix vs. Aimmugen: hepatitis A vaccine (females, age 22-29; follow-up until 12 months)**						
HPV-063: Cervarix vs. Aimmugen (f, 22-29; 12)	3	375	2	377	0.5%	1.51 [0.25, 8.97]
**Subtotal (95% CI)**		**375**		**377**	**0.5%**	**1.51 [0.25, 8.97]**
Total events	3		2			
Heterogeneity: Not applicable						
Test for overall effect: Z = 0.45 (P = 0.65)						
**11.29.3 New onset depression ('medically significant conditions'): Cervarix vs. Havrix: hepatitis A vaccine (females, age 15-25; follow-up until 48 months)**						
HPV-008: Cervarix vs. Havrix (f, 15-25; 48)	177	9319	171	9325	40.0%	1.04 [0.84, 1.28]
**Subtotal (95% CI)**		**9319**		**9325**	**40.0%**	**1.04 [0.84, 1.28]**
Total events	177		171			
Heterogeneity: Not applicable						
Test for overall effect: Z = 0.33 (P = 0.74)						
**11.29.4 New onset depression ('medically significant conditions'): Cervarix vs. Twinrix: hepatitis A and B vaccine (females, age 8-15; follow-up until 12 months)**						
HPV-029: Cervarix vs. Twinrix (f, 8-15; 12)	0	270	1	271	0.2%	0.33 [0.01, 8.18]
**Subtotal (95% CI)**		**270**		**271**	**0.2%**	**0.33 [0.01, 8.18]**
Total events	0		1			
Heterogeneity: Not applicable						
Test for overall effect: Z = 0.67 (P = 0.50)						
**11.29.5 New onset depression ('new medical history'): Gardasil vs. carrier solution (females and males, age 9-16; follow-up until 18 months)**						
V501-018: Gardasil vs. carrier solu. (f&m,9-16;18)	6	1184	2	597	0.7%	1.51 [0.31, 7.47]
**Subtotal (95% CI)**		**1184**		**597**	**0.7%**	**1.51 [0.31, 7.47]**
Total events	6		2			
Heterogeneity: Not applicable						
Test for overall effect: Z = 0.51 (P = 0.61)						
**11.29.6 New onset depression ('new medical history'): Gardasil vs. AAHS (females, age 15-26; follow-up until 45 months)**						
V501-013: Gardasil vs. AAHS (f, 16-24; 45)	70	2723	54	2732	14.1%	1.30 [0.92, 1.85]
V501-015: Gardasil vs. AAHS (f, 15-26; 36)	116	6087	131	6080	28.3%	0.88 [0.69, 1.13]
**Subtotal (95% CI)**		**8810**		**8812**	**42.4%**	**1.05 [0.72, 1.53]**
Total events	186		185			
Heterogeneity: $Tau^2 = 0.05$; $Chi^2 = 3.10$, df = 1 (P = 0.08); $I^2 = 68\%$						
Test for overall effect: Z = 0.26 (P = 0.80)						
**11.29.7 New onset depression ('new medical history'): Gardasil vs. AAHS (females, age 21-46; from 0-7 months)**						
V501-019: Gardasil vs. AAHS (f, 21-46; 48)	7	1911	5	1908	1.3%	1.40 [0.44, 4.40]
**Subtotal (95% CI)**		**1911**		**1908**	**1.3%**	**1.40 [0.44, 4.40]**
Total events	7		5			
Heterogeneity: Not applicable						
Test for overall effect: Z = 0.57 (P = 0.57)						
**11.29.8 New onset depression ('new medical history'): Gardasil vs. AAHS (females, age 21-46; from 7-48 months)**						
V501-019: Gardasil vs. AAHS (f, 21-46; 48)	12	1911	13	1908	0.0%	0.92 [0.42, 2.01]
**Subtotal (95% CI)**		**0**		**0**		**Not estimable**
Total events	0		0			
Heterogeneity: Not applicable						
Test for overall effect: Not applicable						
**11.29.9 New onset depression ('new medical history'): Gardasil vs. AAHS (males, age 15-27; from 0-7 months)**						
V501-020: Gardasil vs. AAHS (m, 15-27; 36)	4	2032	4	2032	0.9%	1.00 [0.25, 3.99]
**Subtotal (95% CI)**		**2032**		**2032**	**0.9%**	**1.00 [0.25, 3.99]**
Total events	4		4			
Heterogeneity: Not applicable						
Test for overall effect: Z = 0.00 (P = 1.00)						
**11.29.10 New onset depression ('new medical history'): Gardasil vs. AAHS (males, age 15-27; from 7-36 months)**						
V501-020: Gardasil vs. AAHS (m, 15-27; 36)	5	2032	9	2033	0.0%	0.56 [0.19, 1.66]
**Subtotal (95% CI)**		**0**		**0**		**Not estimable**
Total events	0		0			
Heterogeneity: Not applicable						
Test for overall effect: Not applicable						
**11.29.11 New onset depression ('new medical history'): HPV 16 vaccine vs. AAHS (females, age 16-25; from 0-7 months)**						
V501-005: HPV 16 vaccine vs. AAHS (f, 16-25; 48)	17	1204	18	1205	4.0%	0.95 [0.49, 1.83]
**Subtotal (95% CI)**		**1204**		**1205**	**4.0%**	**0.95 [0.49, 1.83]**
Total events	17		18			
Heterogeneity: Not applicable						
Test for overall effect: Z = 0.17 (P = 0.87)						
**11.29.12 New onset depression ('new medical history'): HPV 16 vaccine vs. AAHS (females, age 16-25; from 7-48 months)**						
V501-005: HPV 16 vaccine vs. AAHS (f, 16-25; 48)	45	1204	48	1205	0.0%	0.94 [0.63, 1.40]
**Subtotal (95% CI)**		**0**		**0**		**Not estimable**
Total events	0		0			
Heterogeneity: Not applicable						
Test for overall effect: Not applicable						
**Total (95% CI)**		**27987**		**27398**	**100.0%**	**1.02 [0.89, 1.16]**
Total events	443		432			
Heterogeneity: $Tau^2 = 0.00$; $Chi^2 = 4.41$, df = 9 (P = 0.88); $I^2 = 0\%$						
Test for overall effect: Z = 0.28 (P = 0.78)						
Test for subgroup differences: $Chi^2 = 1.30$, df = 8 (P = 1.00), $I^2 = 0\%$						

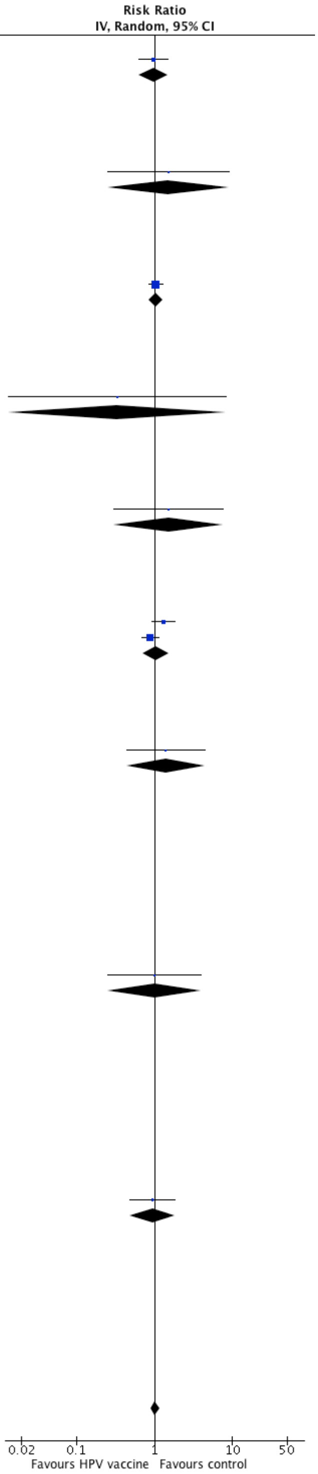

*11.29. Risk ratio for 'medically significant conditions’ (GlaxoSmithKline): 1.02 [0.85, 1.23]; risk ratio for ‘new medical history’ (Merck Sharp & Dohme): 1.01 [0.84, 1.22]; risk ratio for the follow-up periods for the trials V501-005, V501-019 and V501-020: 0.89 [0.63, 1.25]. The trials V501-005, V501-019 and V501-020 split the reporting of new onset diseases into the vaccination period and the follow-up period. To avoid double counting of participants in the total risk ratio estimate, we only included the new onset diseases reported in the vaccination period for the trials V501-005, V501-019 and V501-020.

11.30. Most common new onset diseases (‘medically significant conditions’*) - ‘genitourinary tract gonococcal infection’: intention to treat analysis

Study or Subgroup	HPV vaccine Events	HPV vaccine Total	Control Events	Control Total	Weight	Risk Ratio IV, Random, 95% CI
**11.30.1 New onset genitourinary tract gonococcal infection ('medically significant conditions'): Cervarix vs. Al(OH)3 (females, age 24-72; follow-up until 36 months)**						
HPV-015: Cervarix vs. Al(OH)3 (f, 24-72; 36)	1	2882	1	2871	0.6%	1.00 [0.06, 15.92]
**Subtotal (95% CI)**		**2882**		**2871**	**0.6%**	**1.00 [0.06, 15.92]**
Total events	1		1			
Heterogeneity: Not applicable						
Test for overall effect: Z = 0.00 (P = 1.00)						
**11.30.2 New onset genitourinary tract gonococcal infection ('medically significant conditions'): Cervarix vs. Havrix: hepatitis A vaccine (females, age 15-25; follow-up until 48 months)**						
HPV-008: Cervarix vs. Havrix (f, 15-25; 48)	141	9319	155	9325	95.5%	0.91 [0.73, 1.14]
**Subtotal (95% CI)**		**9319**		**9325**	**95.5%**	**0.91 [0.73, 1.14]**
Total events	141		155			
Heterogeneity: Not applicable						
Test for overall effect: Z = 0.81 (P = 0.42)						
**11.30.3 New onset genitourinary tract gonococcal infection ('new medical history'): Gardasil vs. AAHS (females, age 15-26; follow-up until 45 months)**						
V501-013: Gardasil vs. AAHS (f, 16-24; 45)	2	2723	4	2732	1.7%	0.50 [0.09, 2.74]
V501-015: Gardasil vs. AAHS (f, 15-26; 36)	4	6087	2	6080	1.7%	2.00 [0.37, 10.90]
**Subtotal (95% CI)**		**8810**		**8812**	**3.4%**	**1.00 [0.26, 3.88]**
Total events	6		6			
Heterogeneity: $Tau^2 = 0.21$; $Chi^2 = 1.27$, df = 1 (P = 0.26); $I^2 = 22\%$						
Test for overall effect: Z = 0.00 (P = 1.00)						
**11.30.4 New onset genitourinary tract gonococcal infection ('new medical history'): Gardasil vs. AAHS (males, age 15-27; from 0-7 months)**						
V501-020: Gardasil vs. AAHS (m, 15-27; 36)	1	2032	0	2032	0.5%	3.00 [0.12, 73.60]
**Subtotal (95% CI)**		**2032**		**2032**	**0.5%**	**3.00 [0.12, 73.60]**
Total events	1		0			
Heterogeneity: Not applicable						
Test for overall effect: Z = 0.67 (P = 0.50)						
**11.30.5 New onset genitourinary tract gonococcal infection ('new medical history'): Gardasil vs. AAHS (males, age 15-27; from 7-36 months)**						
V501-020: Gardasil vs. AAHS (m, 15-27; 36)	1	2032	2	2033	0.0%	0.50 [0.05, 5.51]
**Subtotal (95% CI)**		**0**		**0**		**Not estimable**
Total events	0		0			
Heterogeneity: Not applicable						
Test for overall effect: Not applicable						
**Total (95% CI)**		**23043**		**23040**	**100.0%**	**0.92 [0.74, 1.15]**
Total events	149		162			
Heterogeneity: $Tau^2 = 0.00$; $Chi^2 = 1.83$, df = 4 (P = 0.77); $I^2 = 0\%$						
Test for overall effect: Z = 0.75 (P = 0.45)						
Test for subgroup differences: $Chi^2 = 0.55$, df = 3 (P = 0.91), $I^2 = 0\%$						

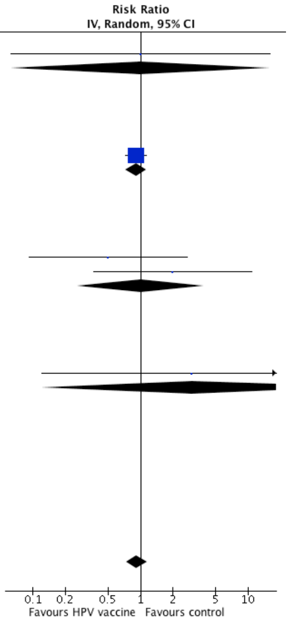

*11.30. Risk ratio for 'medically significant conditions’ (GlaxoSmithKline): 0.91 [0.73, 1.14]; risk ratio for ‘new medical history’ (Merck Sharp & Dohme): 1.15 [0.37, 3.52]; risk ratio for the follow-up period for the trial V501-020: 0.50 [0.05, 5.51]. The trial V501-020 split the reporting of new onset diseases into the vaccination period and the follow-up period. To avoid double counting of participants in the total risk ratio estimate, we only included the new onset diseases reported in the vaccination period for the trial V501-020.

11.31. Most common new onset diseases (‘new medical history’*) - ‘vaginal candidiasis’: intention to treat analysis

Study or Subgroup	HPV vaccine Events	HPV vaccine Total	Control Events	Control Total	Weight	Risk Ratio IV, Random, 95% CI
**11.31.1 New onset vaginal candidiasis ('new medical history'): Gardasil vs. carrier solution (females and males, age 9-16; follow-up until 18 months)**						
V501-018: Gardasil vs. carrier solu. (f&m,9-16;18)	5	1184	3	597	0.2%	0.84 [0.20, 3.50]
**Subtotal (95% CI)**		**1184**		**597**	**0.2%**	**0.84 [0.20, 3.50]**
Total events	5		3			
Heterogeneity: Not applicable						
Test for overall effect: Z = 0.24 (P = 0.81)						
**11.31.2 New onset vaginal candidiasis ('new medical history'): Gardasil vs. AAHS (females, age 15-26; follow-up until 45 months)**						
V501-013: Gardasil vs. AAHS (f, 16-24; 45)	417	2723	453	2732	33.3%	0.92 [0.82, 1.04]
V501-015: Gardasil vs. AAHS (f, 15-26; 36)	841	6087	877	6080	64.5%	0.96 [0.88, 1.05]
**Subtotal (95% CI)**		**8810**		**8812**	**97.8%**	**0.95 [0.88, 1.02]**
Total events	1258		1330			
Heterogeneity: $Tau^2 = 0.00$; $Chi^2 = 0.23$, df = 1 (P = 0.63); $I^2 = 0\%$						
Test for overall effect: Z = 1.53 (P = 0.13)						
**11.31.3 New onset vaginal candidiasis ('new medical history'): HPV 16 vaccine vs. AAHS (females, age 16-25; from 0-7 months)**						
V501-005: HPV 16 vaccine vs. AAHS (f, 16-25; 48)	34	1204	26	1205	1.9%	1.31 [0.79, 2.17]
**Subtotal (95% CI)**		**1204**		**1205**	**1.9%**	**1.31 [0.79, 2.17]**
Total events	34		26			
Heterogeneity: Not applicable						
Test for overall effect: Z = 1.05 (P = 0.30)						
**11.31.4 New onset vaginal candidiasis ('new medical history'): HPV 16 vaccine vs. AAHS (females, age 16-25; from 7-48 months)**						
V501-005: HPV 16 vaccine vs. AAHS (f, 16-25; 48)	74	1204	66	1205	0.0%	1.12 [0.81, 1.55]
**Subtotal (95% CI)**		**0**		**0**		**Not estimable**
Total events	0		0			
Heterogeneity: Not applicable						
Test for overall effect: Not applicable						
**Total (95% CI)**		**11198**		**10614**	**100.0%**	**0.95 [0.89, 1.02]**
Total events	1297		1359			
Heterogeneity: $Tau^2 = 0.00$; $Chi^2 = 1.82$, df = 3 (P = 0.61); $I^2 = 0\%$						
Test for overall effect: Z = 1.38 (P = 0.17)						
Test for subgroup differences: $Chi^2 = 1.59$, df = 2 (P = 0.45), $I^2 = 0\%$						

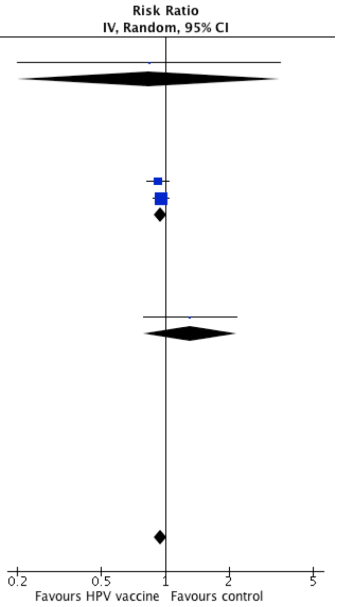

*11.31. Risk ratio for 'medically significant conditions’ (GlaxoSmithKline): not applicable; risk ratio for ‘new medical history’ (Merck Sharp & Dohme): 0.95 [0.89, 1.02]; risk ratio for the follow-up period for the trial V501-005: 1.12 [0.81, 1.55]. The trial V501-005 split the reporting of new onset diseases into the vaccination period and the follow-up period. To avoid double counting of participants in the total risk ratio estimate, we only included the new onset diseases reported in the vaccination period for the trial V501-005.

11.32. Most common new onset diseases (‘new medical history’*) - ‘vaginitis bacterial’: intention to treat analysis

Study or Subgroup	HPV vaccine Events	HPV vaccine Total	Control Events	Control Total	Weight	Risk Ratio IV, Random, 95% CI
**11.32.1 New onset vaginitis bacterial ('new medical history'): Gardasil vs. carrier solution (females and males, age 9-16; follow-up until 18 months)**						
V501-018: Gardasil vs. carrier solu. (f&m,9-16;18)	0	1184	1	597	0.1%	0.17 [0.01, 4.12]
**Subtotal (95% CI)**		**1184**		**597**	**0.1%**	**0.17 [0.01, 4.12]**
Total events	0		1			
Heterogeneity: Not applicable						
Test for overall effect: Z = 1.09 (P = 0.27)						
**11.32.2 New onset vaginitis bacterial ('new medical history'): Gardasil vs. AAHS (females, age 15-26; follow-up until 45 months)**						
V501-013: Gardasil vs. AAHS (f, 16-24; 45)	435	2723	455	2732	38.9%	0.96 [0.85, 1.08]
V501-015: Gardasil vs. AAHS (f, 15-26; 36)	675	6087	665	6080	55.1%	1.01 [0.92, 1.12]
**Subtotal (95% CI)**		**8810**		**8812**	**94.1%**	**0.99 [0.92, 1.07]**
Total events	1110		1120			
Heterogeneity: Tau² = 0.00; Chi² = 0.48, df = 1 (P = 0.49); I² = 0%						
Test for overall effect: Z = 0.23 (P = 0.82)						
**11.32.3 New onset vaginitis bacterial ('new medical history'): Gardasil vs. AAHS (females, age 21-46; from 0-7 months)**						
V501-019: Gardasil vs. AAHS (f, 21-46; 48)	41	1911	52	1908	3.4%	0.79 [0.53, 1.18]
**Subtotal (95% CI)**		**1911**		**1908**	**3.4%**	**0.79 [0.53, 1.18]**
Total events	41		52			
Heterogeneity: Not applicable						
Test for overall effect: Z = 1.16 (P = 0.25)						
**11.32.4 New onset vaginitis bacterial ('new medical history'): Gardasil vs. AAHS (females, age 21-46; from 7-48 months)**						
V501-019: Gardasil vs. AAHS (f, 21-46; 48)	79	1911	88	1908	0.0%	0.90 [0.67, 1.21]
**Subtotal (95% CI)**		**0**		**0**		**Not estimable**
Total events	0		0			
Heterogeneity: Not applicable						
Test for overall effect: Not applicable						
**11.32.5 New onset vaginitis bacterial ('new medical history'): HPV 16 vaccine vs. AAHS (females, age 16-25; from 0-7 months)**						
V501-005: HPV 16 vaccine vs. AAHS (f, 16-25; 48)	34	1204	31	1205	2.4%	1.10 [0.68, 1.77]
**Subtotal (95% CI)**		**1204**		**1205**	**2.4%**	**1.10 [0.68, 1.77]**
Total events	34		31			
Heterogeneity: Not applicable						
Test for overall effect: Z = 0.38 (P = 0.70)						
**11.32.6 New onset vaginitis bacterial ('new medical history'): HPV 16 vaccine vs. AAHS (females, age 16-25; from 7-48 months)**						
V501-005: HPV 16 vaccine vs. AAHS (f, 16-25; 48)	64	1204	72	1205	0.0%	0.89 [0.64, 1.23]
**Subtotal (95% CI)**		**0**		**0**		**Not estimable**
Total events	0		0			
Heterogeneity: Not applicable						
Test for overall effect: Not applicable						
**Total (95% CI)**		**13109**		**12522**	**100.0%**	**0.98 [0.91, 1.06]**
Total events	1185		1204			
Heterogeneity: Tau² = 0.00; Chi² = 3.05, df = 4 (P = 0.55); I² = 0%						
Test for overall effect: Z = 0.41 (P = 0.68)						
Test for subgroup differences: Chi² = 2.57, df = 3 (P = 0.46), I² = 0%						

Risk Ratio
IV, Random, 95% CI

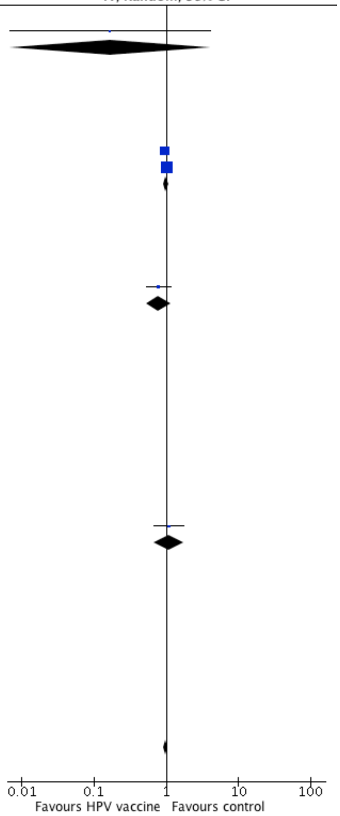

*11.32. Risk ratio for 'medically significant conditions' (GlaxoSmithKline): not applicable; risk ratio for ‘new medical history’ (Merck Sharp & Dohme): 0.98 [0.91, 1.06]; risk ratio for the follow-up periods for the trials V501-005 and V501-019: 0.89 [0.72, 1.11]. The trials V501-005 and V501-019 split the reporting of new onset diseases into the vaccination period and the follow-up period. To avoid double counting of participants in the total risk ratio estimate, we only included the new onset diseases reported in the vaccination period for the trials V501-005 and V501-019.

11.33. Most common new onset diseases (‘new medical history’*) - ‘urinary tract infection’: intention to treat analysis

Study or Subgroup	HPV vaccine Events	HPV vaccine Total	Control Events	Control Total	Weight	Risk Ratio IV, Random, 95% CI
**11.33.1 New onset urinary tract infection('medically significant conditions'): Cervarix vs. Havrix: hepatitis A vaccine (females, age 15-25; follow-up until 48 months)**						
HPV-008: Cervarix vs. Havrix (f, 15-25; 48)	0	9319	1	9325	0.1%	0.33 [0.01, 8.19]
**Subtotal (95% CI)**		**9319**		**9325**	**0.1%**	**0.33 [0.01, 8.19]**
Total events	0		1			
Heterogeneity: Not applicable						
Test for overall effect: Z = 0.67 (P = 0.50)						
**11.33.2 New onset urinary tract infection ('new medical history'): Gardasil vs. carrier solution (females and males, age 9-16; follow-up until 18 months)**						
V501-018: Gardasil vs. carrier solu. (f&m,9-16;18)	15	1184	13	597	1.2%	0.58 [0.28, 1.21]
**Subtotal (95% CI)**		**1184**		**597**	**1.2%**	**0.58 [0.28, 1.21]**
Total events	15		13			
Heterogeneity: Not applicable						
Test for overall effect: Z = 1.44 (P = 0.15)						
**11.33.3 New onset urinary tract infection ('new medical history'): Gardasil vs. AAHS (females, age 15-26; follow-up until 45 months)**						
V501-013: Gardasil vs. AAHS (f, 16-24; 45)	292	2723	276	2732	27.1%	1.06 [0.91, 1.24]
V501-015: Gardasil vs. AAHS (f, 15-26; 36)	630	6087	709	6080	64.0%	0.89 [0.80, 0.98]
**Subtotal (95% CI)**		**8810**		**8812**	**91.1%**	**0.96 [0.81, 1.14]**
Total events	922		985			
Heterogeneity: $Tau^2 = 0.01$; $Chi^2 = 3.56$, df = 1 (P = 0.06); $I^2 = 72\%$						
Test for overall effect: Z = 0.45 (P = 0.65)						
**11.33.4 New onset urinary tract infection ('new medical history'): Gardasil vs. AAHS (females, age 21-46; from 0-7 months)**						
V501-019: Gardasil vs. AAHS (f, 21-46; 48)	21	1911	18	1908	1.7%	1.16 [0.62, 2.18]
**Subtotal (95% CI)**		**1911**		**1908**	**1.7%**	**1.16 [0.62, 2.18]**
Total events	21		18			
Heterogeneity: Not applicable						
Test for overall effect: Z = 0.48 (P = 0.63)						
**11.33.5 New onset urinary tract infection ('new medical history'): Gardasil vs. AAHS (females, age 21-46; from 7-48 months)**						
V501-019: Gardasil vs. AAHS (f, 21-46; 48)	37	1911	44	1908	0.0%	0.84 [0.54, 1.29]
**Subtotal (95% CI)**		**0**		**0**		**Not estimable**
Total events	0		0			
Heterogeneity: Not applicable						
Test for overall effect: Not applicable						
**11.33.6 New onset urinary tract infection ('new medical history'): Gardasil vs. AAHS (males, age 15-27; from 0-7 months)**						
V501-020: Gardasil vs. AAHS (m, 15-27; 36)	5	2032	5	2032	0.4%	1.00 [0.29, 3.45]
**Subtotal (95% CI)**		**2032**		**2032**	**0.4%**	**1.00 [0.29, 3.45]**
Total events	5		5			
Heterogeneity: Not applicable						
Test for overall effect: Z = 0.00 (P = 1.00)						
**11.33.7 New onset urinary tract infection ('new medical history'): Gardasil vs. AAHS (males, age 15-27; from 7-36 months)**						
V501-020: Gardasil vs. AAHS (m, 15-27; 36)	12	2032	15	2033	0.0%	0.80 [0.38, 1.71]
**Subtotal (95% CI)**		**0**		**0**		**Not estimable**
Total events	0		0			
Heterogeneity: Not applicable						
Test for overall effect: Not applicable						
**11.33.8 New onset urinary tract infection ('new medical history'): HPV 16 vaccine vs. AAHS (females, age 16-25; from 0-7 months)**						
V501-005: HPV 16 vaccine vs. AAHS (f, 16-25; 48)	58	1204	64	1205	5.5%	0.91 [0.64, 1.28]
**Subtotal (95% CI)**		**1204**		**1205**	**5.5%**	**0.91 [0.64, 1.28]**
Total events	58		64			
Heterogeneity: Not applicable						
Test for overall effect: Z = 0.55 (P = 0.58)						
**11.33.9 New onset urinary tract infection ('new medical history'): HPV 16 vaccine vs. AAHS (females, age 16-25; from 7-48 months)**						
V501-005: HPV 16 vaccine vs. AAHS (f, 16-25; 48)	100	1204	87	1205	0.0%	1.15 [0.87, 1.52]
**Subtotal (95% CI)**		**0**		**0**		**Not estimable**
Total events	0		0			
Heterogeneity: Not applicable						
Test for overall effect: Not applicable						
**11.33.10 New onset urinary tract infection ('new medical history'): Gardasil 9 vs. saline placebo (females, age 12-26; follow-up until 7 months)**						
V503-006: Gardasil 9 vs. saline (f, 12-26;7)	2	618	0	306	0.1%	2.48 [0.12, 51.49]
**Subtotal (95% CI)**		**618**		**306**	**0.1%**	**2.48 [0.12, 51.49]**
Total events	2		0			
Heterogeneity: Not applicable						
Test for overall effect: Z = 0.59 (P = 0.56)						
**Total (95% CI)**		**25078**		**24185**	**100.0%**	**0.93 [0.86, 1.01]**
Total events	1023		1086			
Heterogeneity: $Tau^2 = 0.00$; $Chi^2 = 6.47$, df = 7 (P = 0.49); $I^2 = 0\%$						
Test for overall effect: Z = 1.68 (P = 0.09)						
Test for subgroup differences: $Chi^2 = 2.99$, df = 6 (P = 0.81), $I^2 = 0\%$						

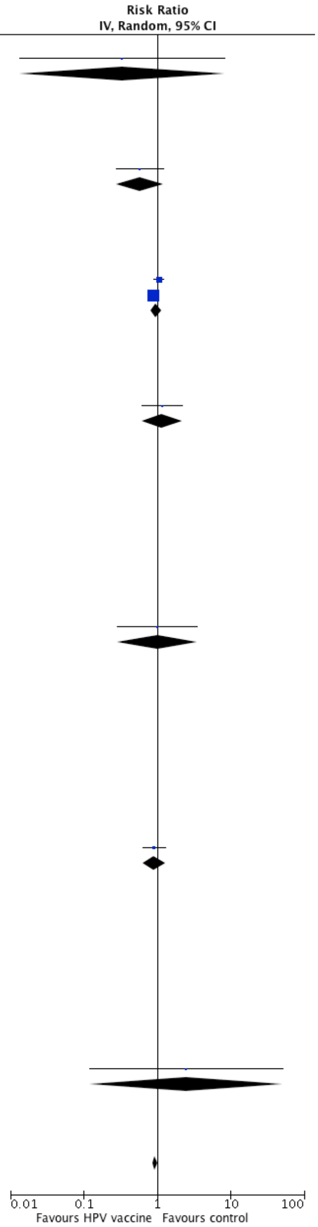

*11.33. Risk ratio for 'medically significant conditions' (GlaxoSmithKline): 0.33 [0.01, 8.19]; risk ratio for ‘new medical history’ (Merck Sharp & Dohme): 0.93 [0.86, 1.02]; risk ratio for the follow-up periods for the trials V501-005, V501-019 and V501-020: 1.03 [0.82, 1.28]. The trials V501-005, V501-019 and V501-020 split the reporting of new onset diseases into the vaccination period and the follow-up period. To avoid double counting of participants in the total risk ratio estimate, we only included the new onset diseases reported in the vaccination period for the trials V501-005, V501-019 and V501-020.

11.34. New onset diseases most increased by the HPV vaccines (‘medically significant conditions’*) - ‘back pain’: intention to treat analysis

Study or Subgroup	HPV vaccine Events	HPV vaccine Total	Control Events	Control Total	Weight	Risk Ratio IV, Random, 95% CI
**11.34.1 New onset back pain ('medically significant conditions'): Cervarix vs. Al(OH)3 (females, age 18–35; follow-up until 12 months)**						
HPV-031: Cervarix vs. Al(OH)3 (f, 18–35; 7)	2	176	1	178	0.4%	2.02 [0.19, 22.11]
HPV-035: Cervarix vs. Al(OH)3 (f, 18–35; 7)	2	150	1	150	0.4%	2.00 [0.18, 21.82]
**Subtotal (95% CI)**		**326**		**328**	**0.7%**	**2.01 [0.37, 10.90]**
Total events	4		2			
Heterogeneity: $Tau^2 = 0.00$; $Chi^2 = 0.00$, df = 1 (P = 0.99); $I^2 = 0\%$						
Test for overall effect: Z = 0.81 (P = 0.42)						
**11.34.2 New onset back pain ('medically significant conditions'): Cervarix vs. Al(OH)3 (females, age 24–72; follow-up until 36 months)**						
HPV-015: Cervarix vs. Al(OH)3 (f, 24–72; 36)	50	2882	40	2871	12.3%	1.25 [0.82, 1.88]
**Subtotal (95% CI)**		**2882**		**2871**	**12.3%**	**1.25 [0.82, 1.88]**
Total events	50		40			
Heterogeneity: Not applicable						
Test for overall effect: Z = 1.04 (P = 0.30)						
**11.34.3 New onset back pain ('medically significant conditions'): Cervarix vs. Aimmugen: hepatitis A vaccine (females, age 22–29; follow-up until 12 months)**						
HPV-063: Cervarix vs. Aimmugen (f, 22–29; 12)	1	375	1	377	0.3%	1.01 [0.06, 16.01]
**Subtotal (95% CI)**		**375**		**377**	**0.3%**	**1.01 [0.06, 16.01]**
Total events	1		1			
Heterogeneity: Not applicable						
Test for overall effect: Z = 0.00 (P = 1.00)						
**11.34.4 New onset back pain ('medically significant conditions'): Cervarix vs. Havrix: hepatitis A vaccine (females, age 10–14; follow-up until 12 months)**						
HPV-013: Cervarix vs. Havrix (f, 10–14; 12)	3	1035	2	1032	0.7%	1.50 [0.25, 8.93]
**Subtotal (95% CI)**		**1035**		**1032**	**0.7%**	**1.50 [0.25, 8.93]**
Total events	3		2			
Heterogeneity: Not applicable						
Test for overall effect: Z = 0.44 (P = 0.66)						
**11.34.5 New onset back pain ('medically significant conditions'): Cervarix vs. Havrix: hepatitis A vaccine (females, age 15–25; follow-up until 48 months)**						
HPV-008: Cervarix vs. Havrix (f, 15–25; 48)	54	9319	35	9325	11.7%	1.54 [1.01, 2.36]
**Subtotal (95% CI)**		**9319**		**9325**	**11.7%**	**1.54 [1.01, 2.36]**
Total events	54		35			
Heterogeneity: Not applicable						
Test for overall effect: Z = 2.01 (P = 0.04)						
**11.34.6 New onset back pain ('new medical history'): Gardasil vs. carrier solution (females and males, age 9–16; follow-up until 18 months)**						
V501-018: Gardasil vs. carrier solu. (f&m,9–16;18)	13	1184	6	597	2.3%	1.09 [0.42, 2.86]
**Subtotal (95% CI)**		**1184**		**597**	**2.3%**	**1.09 [0.42, 2.86]**
Total events	13		6			
Heterogeneity: Not applicable						
Test for overall effect: Z = 0.18 (P = 0.86)						
**11.34.7 New onset back pain ('new medical history'): Gardasil vs. AAHS (females, age 15–26; follow-up until 45 months)**						
V501-013: Gardasil vs. AAHS (f, 16–24; 45)	59	2723	67	2732	17.5%	0.88 [0.63, 1.25]
V501-015: Gardasil vs. AAHS (f, 15–26; 36)	160	6087	132	6080	40.5%	1.21 [0.96, 1.52]
**Subtotal (95% CI)**		**8810**		**8812**	**58.0%**	**1.06 [0.78, 1.44]**
Total events	219		199			
Heterogeneity: $Tau^2 = 0.03$; $Chi^2 = 2.22$, df = 1 (P = 0.14); $I^2 = 55\%$						
Test for overall effect: Z = 0.40 (P = 0.69)						
**11.34.8 New onset back pain ('new medical history'): Gardasil vs. AAHS (females, age 21–46; from 0–7 months)**						
V501-019: Gardasil vs. AAHS (f, 21–46; 48)	19	1911	20	1908	5.4%	0.95 [0.51, 1.77]
**Subtotal (95% CI)**		**1911**		**1908**	**5.4%**	**0.95 [0.51, 1.77]**
Total events	19		20			
Heterogeneity: Not applicable						
Test for overall effect: Z = 0.17 (P = 0.87)						
**11.34.9 New onset back pain ('new medical history'): Gardasil vs. AAHS (females, age 21–46; from 7–48 months)**						
V501-019: Gardasil vs. AAHS (f, 21–46; 48)	26	1911	27	1908	0.0%	0.96 [0.56, 1.64]
**Subtotal (95% CI)**		**0**		**0**		**Not estimable**
Total events	0		0			
Heterogeneity: Not applicable						
Test for overall effect: Not applicable						
**11.34.10 New onset back pain ('new medical history'): Gardasil vs. AAHS (males, age 15–27; from 0–7 months)**						
V501-020: Gardasil vs. AAHS (m, 15–27; 36)	13	2032	15	2032	3.8%	0.87 [0.41, 1.82]
**Subtotal (95% CI)**		**2032**		**2032**	**3.8%**	**0.87 [0.41, 1.82]**
Total events	13		15			
Heterogeneity: Not applicable						
Test for overall effect: Z = 0.38 (P = 0.70)						
**11.34.11 New onset back pain ('new medical history'): Gardasil vs. AAHS (males, age 15–27; from 7–36 months)**						
V501-020: Gardasil vs. AAHS (m, 15–27; 36)	17	2032	25	2033	0.0%	0.68 [0.37, 1.26]
**Subtotal (95% CI)**		**0**		**0**		**Not estimable**
Total events	0		0			
Heterogeneity: Not applicable						
Test for overall effect: Not applicable						
**11.34.12 New onset back pain ('new medical history'): HPV 16 vaccine vs. AAHS (females, age 16–25; from 0–7 months)**						
V501-005: HPV 16 vaccine vs. AAHS (f, 16–25; 48)	17	1204	15	1205	4.4%	1.13 [0.57, 2.26]
**Subtotal (95% CI)**		**1204**		**1205**	**4.4%**	**1.13 [0.57, 2.26]**
Total events	17		15			
Heterogeneity: Not applicable						
Test for overall effect: Z = 0.36 (P = 0.72)						
**11.34.13 New onset back pain ('new medical history'): HPV 16 vaccine vs. AAHS (females, age 16–25; from 7–48 months)**						
V501-005: HPV 16 vaccine vs. AAHS (f, 16–25; 48)	17	1204	19	1205	0.0%	0.90 [0.47, 1.71]
**Subtotal (95% CI)**		**0**		**0**		**Not estimable**
Total events	0		0			
Heterogeneity: Not applicable						
Test for overall effect: Not applicable						
**11.34.14 New onset back pain ('new medical history'): Gardasil 9 vs. saline placebo (females, age 12–26; follow-up until 7 months)**						
V503-006: Gardasil 9 vs. saline (f, 12–26;7)	4	618	1	306	0.4%	1.98 [0.22, 17.64]
**Subtotal (95% CI)**		**618**		**306**	**0.4%**	**1.98 [0.22, 17.64]**
Total events	4		1			
Heterogeneity: Not applicable						
Test for overall effect: Z = 0.61 (P = 0.54)						
**Total (95% CI)**		**29696**		**28793**	**100.0%**	**1.15 [1.00, 1.33]**
Total events	397		336			
Heterogeneity: $Tau^2 = 0.00$; $Chi^2 = 6.11$, df = 12 (P = 0.91); $I^2 = 0\%$						
Test for overall effect: Z = 1.94 (P = 0.05)						
Test for subgroup differences: $Chi^2 = 3.90$, df = 10 (P = 0.95), $I^2 = 0\%$						

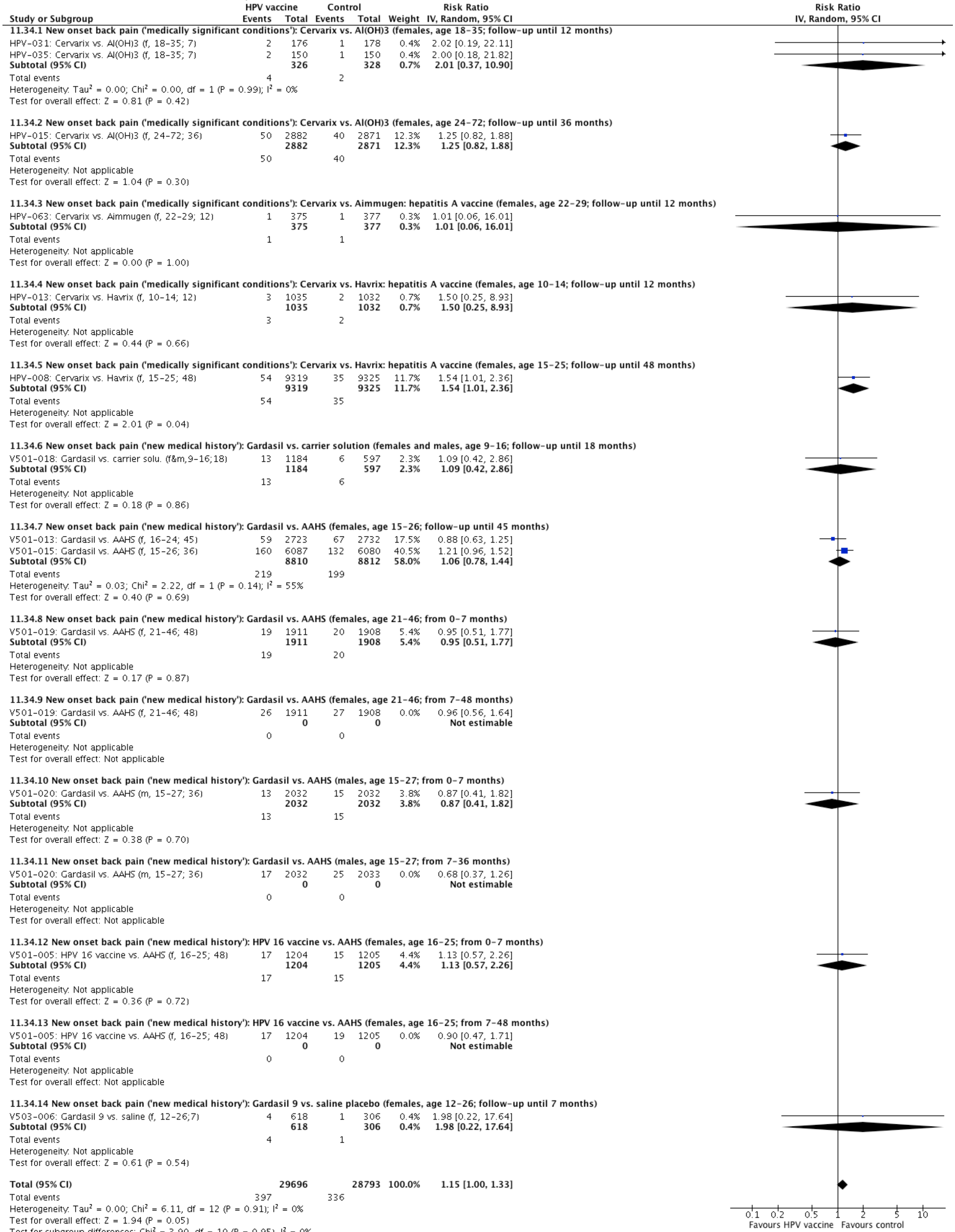

*11.34. Risk ratio for 'medically significant conditions' (GlaxoSmithKline): **1.40 [1.05, 1.86]**; risk ratio for ‘new medical history’ (Merck Sharp & Dohme): 1.08 [0.91, 1.28]; risk ratio for the follow-up periods for the trials V501-005, V501-019 and V501-020: 0.85 [0.60, 1.19]. The trials V501-005, V501-019 and V501-020 split the reporting of new onset diseases into the vaccination period and the follow-up period. To avoid double counting of participants in the total risk ratio estimate, we only included the new onset diseases reported in the vaccination period for the trials V501-005, V501-019 and V501-020.

11.35. New onset diseases most increased by the HPV vaccines (‘medically significant conditions’*) - ‘abdominal pain’: intention to treat analysis

Study or Subgroup	HPV vaccine Events	HPV vaccine Total	Control Events	Control Total	Weight	Risk Ratio IV, Random, 95% CI
**11.35.1 New onset abdominal pain ('medically significant conditions'): Cervarix vs. Al(OH)3 (females, age 15-25; follow-up until 7 months)**						
HPV-038: Cervarix vs. Al(OH)3 (f, 15-25; 7)	0	149	1	76	0.4%	0.17 [0.01, 4.15]
**Subtotal (95% CI)**		**149**		**76**	**0.4%**	**0.17 [0.01, 4.15]**
Total events	0		1			
Heterogeneity: Not applicable						
Test for overall effect: Z = 1.09 (P = 0.28)						
**11.35.2 New onset abdominal pain ('medically significant conditions'): Cervarix vs. Al(OH)3 (females, age 24-72; follow-up until 36 months)**						
HPV-015: Cervarix vs. Al(OH)3 (f, 24-72; 36)	21	2882	14	2871	7.6%	1.49 [0.76, 2.93]
**Subtotal (95% CI)**		**2882**		**2871**	**7.6%**	**1.49 [0.76, 2.93]**
Total events	21		14			
Heterogeneity: Not applicable						
Test for overall effect: Z = 1.17 (P = 0.24)						
**11.35.3 New onset abdominal pain ('medically significant conditions'): Cervarix vs. Aimmugen: hepatitis A vaccine (females, age 22-29; follow-up until 12 months)**						
HPV-063: Cervarix vs. Aimmugen (f, 22-29; 12)	1	375	0	377	0.4%	3.02 [0.12, 73.80]
**Subtotal (95% CI)**		**375**		**377**	**0.4%**	**3.02 [0.12, 73.80]**
Total events	1		0			
Heterogeneity: Not applicable						
Test for overall effect: Z = 0.68 (P = 0.50)						
**11.35.4 New onset abdominal pain ('medically significant conditions'): Cervarix vs. Engerix: hepatitis B vaccine (females and males, age 9-15; follow-up until 12 months)**						
HPV-030: Cervarix vs. Engerix (f, 9-15; 12)	1	247	0	246	0.4%	2.99 [0.12, 72.99]
**Subtotal (95% CI)**		**247**		**246**	**0.4%**	**2.99 [0.12, 72.99]**
Total events	1		0			
Heterogeneity: Not applicable						
Test for overall effect: Z = 0.67 (P = 0.50)						
**11.35.5 New onset abdominal pain ('medically significant conditions'): Cervarix vs. Havrix: hepatitis A vaccine (females, age 9-15; follow-up until 12 months)**						
HPV-013: Cervarix vs. Havrix (f, 10-14; 12)	6	1035	3	1032	2.2%	1.99 [0.50, 7.95]
HPV-033: Cervarix vs. Havrix (f, 9-15; 7)	2	160	0	161	0.5%	5.03 [0.24, 103.97]
**Subtotal (95% CI)**		**1195**		**1193**	**2.7%**	**2.34 [0.66, 8.23]**
Total events	8		3			
Heterogeneity: $Tau^2 = 0.00$; $Chi^2 = 0.30$, df = 1 (P = 0.59); $I^2 = 0\%$						
Test for overall effect: Z = 1.32 (P = 0.19)						
**11.35.6 New onset abdominal pain ('medically significant conditions'): Cervarix vs. Havrix: hepatitis A vaccine (females, age 15-25; follow-up until 48 months)**						
HPV-008: Cervarix vs. Havrix (f, 15-25; 48)	54	9319	42	9325	15.0%	1.29 [0.86, 1.92]
**Subtotal (95% CI)**		**9319**		**9325**	**15.0%**	**1.29 [0.86, 1.92]**
Total events	54		42			
Heterogeneity: Not applicable						
Test for overall effect: Z = 1.23 (P = 0.22)						
**11.35.7 New onset abdominal pain ('new medical history'): Gardasil vs. carrier solution (females and males, age 9-16; follow-up until 18 months)**						
V501-018: Gardasil vs. carrier solu. (f&m,9-16;18)	33	1184	7	597	5.7%	2.38 [1.06, 5.34]
**Subtotal (95% CI)**		**1184**		**597**	**5.7%**	**2.38 [1.06, 5.34]**
Total events	33		7			
Heterogeneity: Not applicable						
Test for overall effect: Z = 2.10 (P = 0.04)						
**11.35.8 New onset abdominal pain ('new medical history'): Gardasil vs. AAHS (females, age 15-26; follow-up until 45 months)**						
V501-013: Gardasil vs. AAHS (f, 16-24; 45)	71	2723	49	2732	16.8%	1.45 [1.01, 2.08]
V501-015: Gardasil vs. AAHS (f, 15-26; 36)	136	6087	154	6080	23.7%	0.88 [0.70, 1.11]
**Subtotal (95% CI)**		**8810**		**8812**	**40.5%**	**1.11 [0.68, 1.81]**
Total events	207		203			
Heterogeneity: $Tau^2 = 0.10$; $Chi^2 = 5.28$, df = 1 (P = 0.02); $I^2 = 81\%$						
Test for overall effect: Z = 0.42 (P = 0.68)						
**11.35.9 New onset abdominal pain ('new medical history'): Gardasil vs. AAHS (females, age 21-46; from 0-7 months)**						
V501-019: Gardasil vs. AAHS (f, 21-46; 48)	9	1911	7	1908	4.1%	1.28 [0.48, 3.44]
**Subtotal (95% CI)**		**1911**		**1908**	**4.1%**	**1.28 [0.48, 3.44]**
Total events	9		7			
Heterogeneity: Not applicable						
Test for overall effect: Z = 0.50 (P = 0.62)						
**11.35.10 New onset abdominal pain ('new medical history'): Gardasil vs. AAHS (females, age 21-46; from 7-48 months)**						
V501-019: Gardasil vs. AAHS (f, 21-46; 48)	13	1911	13	1908	0.0%	1.00 [0.46, 2.15]
**Subtotal (95% CI)**		**0**		**0**		**Not estimable**
Total events	0		0			
Heterogeneity: Not applicable						
Test for overall effect: Not applicable						
**11.35.11 New onset abdominal pain ('new medical history'): Gardasil vs. AAHS (males, age 15-27; from 0-7 months)**						
V501-020: Gardasil vs. AAHS (m, 15-27; 36)	4	2032	2	2032	1.5%	2.00 [0.37, 10.91]
**Subtotal (95% CI)**		**2032**		**2032**	**1.5%**	**2.00 [0.37, 10.91]**
Total events	4		2			
Heterogeneity: Not applicable						
Test for overall effect: Z = 0.80 (P = 0.42)						
**11.35.12 New onset abdominal pain ('new medical history'): Gardasil vs. AAHS (males, age 15-27; from 7-36 months)**						
V501-020: Gardasil vs. AAHS (m, 15-27; 36)	3	2032	5	2033	0.0%	0.60 [0.14, 2.51]
**Subtotal (95% CI)**		**0**		**0**		**Not estimable**
Total events	0		0			
Heterogeneity: Not applicable						
Test for overall effect: Not applicable						
**11.35.13 New onset abdominal pain ('new medical history'): HPV 16 vaccine vs. AAHS (females, age 16-25; from 7-48 months)**						
V501-005: HPV 16 vaccine vs. AAHS (f, 16-25; 48)	95	1204	92	1205	21.1%	1.03 [0.78, 1.36]
**Subtotal (95% CI)**		**1204**		**1205**	**21.1%**	**1.03 [0.78, 1.36]**
Total events	95		92			
Heterogeneity: Not applicable						
Test for overall effect: Z = 0.23 (P = 0.81)						
**11.35.14 New onset abdominal pain ('new medical history'): Gardasil 9 vs. saline placebo (females, age 12-26; follow-up until 7 months)**						
V503-006: Gardasil 9 vs. saline (f, 12-26;7)	0	618	3	306	0.5%	0.07 [0.00, 1.37]
**Subtotal (95% CI)**		**618**		**306**	**0.5%**	**0.07 [0.00, 1.37]**
Total events	0		3			
Heterogeneity: Not applicable						
Test for overall effect: Z = 1.75 (P = 0.08)						
**Total (95% CI)**		**29926**		**28948**	**100.0%**	**1.21 [0.98, 1.50]**
Total events	433		374			
Heterogeneity: $Tau^2 = 0.04$; $Chi^2 = 18.56$, df = 13 (P = 0.14); $I^2 = 30\%$						
Test for overall effect: Z = 1.75 (P = 0.08)						
Test for subgroup differences: $Chi^2 = 11.51$, df = 11 (P = 0.40), $I^2 = 4.5\%$						

Risk Ratio
IV, Random, 95% CI

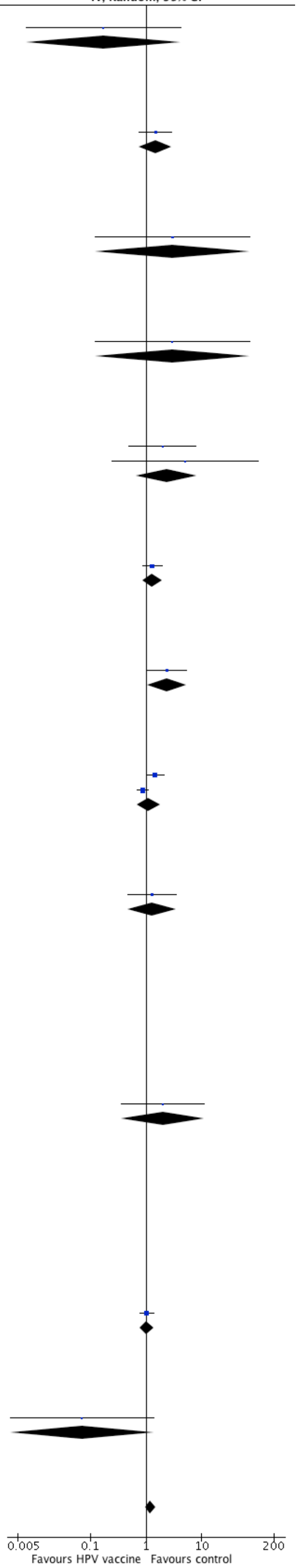

*11.35. Risk ratio for 'medically significant conditions’ (GlaxoSmithKline): **1.38 [1.00, 1.92]**; risk ratio for ‘new medical history’ (Merck Sharp & Dohme): 1.17 [0.87, 1.57]; risk ratio for the follow-up periods for the trials V501-019 and V501-020: 0.89 [0.45, 1.75]. The trials V501-019 and V501-020 split the reporting of new onset diseases into the vaccination period and the follow-up period. To avoid double counting of participants in the total risk ratio estimate, we only included the new onset diseases reported in the vaccination period for the trials V501-019 and V501-020.

11.36. New onset diseases most increased by the HPV vaccines (‘medically significant conditions’*) - ‘headache’: intention to treat analysis

Study or Subgroup	HPV vaccine Events	HPV vaccine Total	Control Events	Control Total	Weight	Risk Ratio IV, Random, 95% CI
**11.36.1 New onset headache ('medically significant conditions'): Cervarix vs. Al(OH)3 (females, age 18-35; follow-up until 7 months)**						
HPV-031: Cervarix vs. Al(OH)3 (f, 18-35; 7)	0	176	1	178	0.2%	0.34 [0.01, 8.22]
HPV-035: Cervarix vs. Al(OH)3 (f, 18-35; 7)	2	150	0	150	0.2%	5.00 [0.24, 103.28]
**Subtotal (95% CI)**		**326**		**328**	**0.4%**	**1.36 [0.10, 19.14]**
Total events	2		1			
Heterogeneity: Tau² = 1.12; Chi² = 1.44, df = 1 (P = 0.23); I² = 31%						
Test for overall effect: Z = 0.23 (P = 0.82)						
**11.36.2 New onset headache ('medically significant conditions'): Cervarix vs. Al(OH)3 (females, age 24-72; follow-up until 36 months)**						
HPV-015: Cervarix vs. Al(OH)3 (f, 24-72; 36)	37	2882	14	2871	4.5%	2.63 [1.43, 4.86]
**Subtotal (95% CI)**		**2882**		**2871**	**4.5%**	**2.63 [1.43, 4.86]**
Total events	37		14			
Heterogeneity: Not applicable						
Test for overall effect: Z = 3.10 (P = 0.002)						
**11.36.3 New onset headache ('medically significant conditions'): Cervarix vs. Aimmugen: hepatitis A vaccine (females, age 22-29; follow-up until 12 months)**						
HPV-063: Cervarix vs. Aimmugen (f, 22-29; 12)	1	375	1	377	0.2%	1.01 [0.06, 16.01]
**Subtotal (95% CI)**		**375**		**377**	**0.2%**	**1.01 [0.06, 16.01]**
Total events	1		1			
Heterogeneity: Not applicable						
Test for overall effect: Z = 0.00 (P = 1.00)						
**11.36.4 New onset headache ('medically significant conditions'): Cervarix vs. Havrix: hepatitis A vaccine (females, age 10-14; follow-up until 12 months)**						
HPV-013: Cervarix vs. Havrix (f, 10-14; 12)	6	1035	8	1032	1.6%	0.75 [0.26, 2.15]
**Subtotal (95% CI)**		**1035**		**1032**	**1.6%**	**0.75 [0.26, 2.15]**
Total events	6		8			
Heterogeneity: Not applicable						
Test for overall effect: Z = 0.54 (P = 0.59)						
**11.36.5 New onset headache ('medically significant conditions'): Cervarix vs. Havrix: hepatitis A vaccine (females, age 15-25; follow-up until 48 months)**						
HPV-008: Cervarix vs. Havrix (f, 15-25; 48)	43	9319	41	9325	8.4%	1.05 [0.68, 1.61]
**Subtotal (95% CI)**		**9319**		**9325**	**8.4%**	**1.05 [0.68, 1.61]**
Total events	43		41			
Heterogeneity: Not applicable						
Test for overall effect: Z = 0.22 (P = 0.82)						
**11.36.6 New onset headache ('medically significant conditions'): Cervarix vs. Twinrix: hepatitis A and B vaccine (females, age 8-15; follow-up until 12 months)**						
HPV-029: Cervarix vs. Twinrix (f, 8-15; 12)	0	270	1	271	0.2%	0.33 [0.01, 8.18]
**Subtotal (95% CI)**		**270**		**271**	**0.2%**	**0.33 [0.01, 8.18]**
Total events	0		1			
Heterogeneity: Not applicable						
Test for overall effect: Z = 0.67 (P = 0.50)						
**11.36.7 New onset headache ('new medical history'): Gardasil vs. carrier solution (females and males, age 9-16; follow-up until 18 months)**						
V501-018: Gardasil vs. carrier solu. (f&m,9-16;18)	58	1184	30	597	8.3%	0.97 [0.63, 1.50]
**Subtotal (95% CI)**		**1184**		**597**	**8.3%**	**0.97 [0.63, 1.50]**
Total events	58		30			
Heterogeneity: Not applicable						
Test for overall effect: Z = 0.12 (P = 0.91)						
**11.36.8 New onset headache ('new medical history'): Gardasil vs. AAHS (females, age 15-26; follow-up until 45 months)**						
V501-013: Gardasil vs. AAHS (f, 16-24; 45)	250	2723	212	2732	25.3%	1.18 [0.99, 1.41]
V501-015: Gardasil vs. AAHS (f, 15-26; 36)	248	6087	251	6080	25.7%	0.99 [0.83, 1.17]
**Subtotal (95% CI)**		**8810**		**8812**	**51.0%**	**1.08 [0.90, 1.29]**
Total events	498		463			
Heterogeneity: Tau² = 0.01; Chi² = 2.10, df = 1 (P = 0.15); I² = 52%						
Test for overall effect: Z = 0.85 (P = 0.40)						
**11.36.9 New onset headache ('new medical history'): Gardasil vs. AAHS (females, age 21-46; from 0-7 months)**						
V501-019: Gardasil vs. AAHS (f, 21-46; 48)	64	1911	80	1908	12.8%	0.80 [0.58, 1.10]
**Subtotal (95% CI)**		**1911**		**1908**	**12.8%**	**0.80 [0.58, 1.10]**
Total events	64		80			
Heterogeneity: Not applicable						
Test for overall effect: Z = 1.37 (P = 0.17)						
**11.36.10 New onset headache ('new medical history'): Gardasil vs. AAHS (females, age 21-46; from 7-48 months)**						
V501-019: Gardasil vs. AAHS (f, 21-46; 48)	23	1911	23	1908	0.0%	1.00 [0.56, 1.77]
**Subtotal (95% CI)**		**0**		**0**		**Not estimable**
Total events	0		0			
Heterogeneity: Not applicable						
Test for overall effect: Not applicable						
**11.36.11 New onset headache ('new medical history'): Gardasil vs. AAHS (males, age 15-27; from 0-7 months)**						
V501-020: Gardasil vs. AAHS (m, 15-27; 36)	23	2032	21	2032	4.9%	1.10 [0.61, 1.97]
**Subtotal (95% CI)**		**2032**		**2032**	**4.9%**	**1.10 [0.61, 1.97]**
Total events	23		21			
Heterogeneity: Not applicable						
Test for overall effect: Z = 0.30 (P = 0.76)						
**11.36.12 New onset headache ('new medical history'): Gardasil vs. AAHS (males, age 15-27; from 7-36 months)**						
V501-020: Gardasil vs. AAHS (m, 15-27; 36)	13	2032	5	2033	0.0%	2.60 [0.93, 7.28]
**Subtotal (95% CI)**		**0**		**0**		**Not estimable**
Total events	0		0			
Heterogeneity: Not applicable						
Test for overall effect: Not applicable						
**11.36.13 New onset headache ('new medical history'): HPV 16 vaccine vs. AAHS (females, age 16-25; from 0-7 months)**						
V501-005: HPV 16 vaccine vs. AAHS (f, 16-25; 48)	30	1204	28	1205	6.2%	1.07 [0.64, 1.78]
**Subtotal (95% CI)**		**1204**		**1205**	**6.2%**	**1.07 [0.64, 1.78]**
Total events	30		28			
Heterogeneity: Not applicable						
Test for overall effect: Z = 0.27 (P = 0.79)						
**11.36.14 New onset headache ('new medical history'): HPV 16 vaccine vs. AAHS (females, age 16-25; from 7-48 months)**						
V501-005: HPV 16 vaccine vs. AAHS (f, 16-25; 48)	19	1204	23	1205	0.0%	0.83 [0.45, 1.51]
**Subtotal (95% CI)**		**0**		**0**		**Not estimable**
Total events	0		0			
Heterogeneity: Not applicable						
Test for overall effect: Not applicable						
**11.36.15 New onset headache ('new medical history'): Gardasil 9 vs. saline placebo (females, age 12-26; follow-up until 7 months)**						
V503-006: Gardasil 9 vs. saline (f, 12-26;7)	9	618	5	306	1.6%	0.89 [0.30, 2.64]
**Subtotal (95% CI)**		**618**		**306**	**1.6%**	**0.89 [0.30, 2.64]**
Total events	9		5			
Heterogeneity: Not applicable						
Test for overall effect: Z = 0.21 (P = 0.84)						
**Total (95% CI)**		**29966**		**29064**	**100.0%**	**1.06 [0.92, 1.22]**
Total events	771		693			
Heterogeneity: Tau² = 0.01; Chi² = 16.28, df = 13 (P = 0.23); I² = 20%						
Test for overall effect: Z = 0.82 (P = 0.41)						
Test for subgroup differences: Chi² = 12.66, df = 11 (P = 0.32), I² = 13.1%						

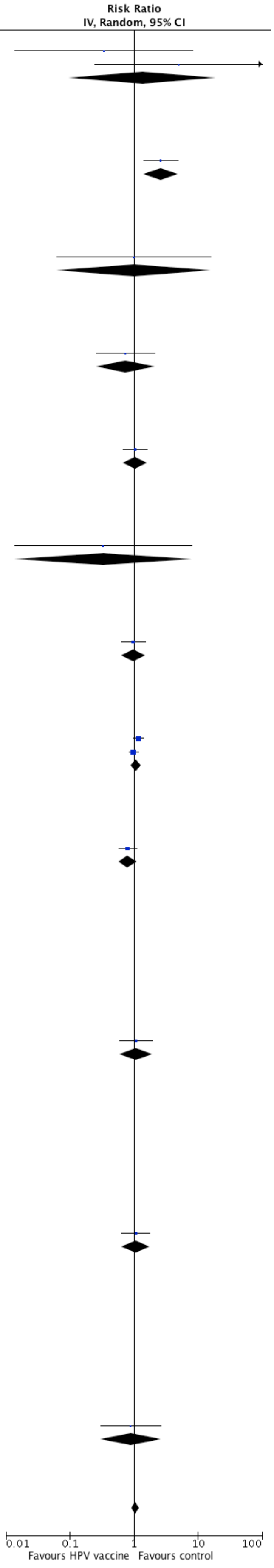

*11.36. Risk ratio for 'medically significant conditions’ (GlaxoSmithKline): 1.29 [0.75, 2.24]; risk ratio for ‘new medical history’ (Merck Sharp & Dohme): 1.04 [0.93, 1.15]; risk ratio for the follow-up periods for the trials V501-005, V501-019 and V501-020: 1.13 [0.65, 1.94]. The trials V501-005, V501-019 and V501-020 split the reporting of new onset diseases into the vaccination period and the follow-up period. To avoid double counting of participants in the total risk ratio estimate, we only included the new onset diseases reported in the vaccination period for the trials V501-005, V501-019 and V501-020.

11.37. New onset diseases most increased by the HPV vaccines (‘new medical history’*) - ‘headache’: intention to treat analysis

See analysis 11.36.

11.38. New onset diseases most increased by the HPV vaccines (‘new medical history’*) - ‘joint sprain’: intention to treat analysis

Study or Subgroup	HPV vaccine Events	HPV vaccine Total	Control Events	Control Total	Weight	Risk Ratio IV, Random, 95% CI
**11.38.1 New onset joint sprain ('medically significant conditions'): Cervarix vs. Aimmugen: hepatitis A vaccine (females, age 22-29; follow-up until 12 months)**						
HPV-063: Cervarix vs. Aimmugen (f, 22-29; 12)	1	375	0	377	1.4%	3.02 [0.12, 73.80]
**Subtotal (95% CI)**		**375**		**377**	**1.4%**	**3.02 [0.12, 73.80]**
Total events	1		0			
Heterogeneity: Not applicable						
Test for overall effect: Z = 0.68 (P = 0.50)						
**11.38.2 New onset joint sprain ('medically significant conditions'): Cervarix vs. Engerix: hepatitis B vaccine (females and males, age 9-15; follow-up until 12 months)**						
HPV-030: Cervarix vs. Engerix (f, 9-15; 12)	0	247	1	246	1.4%	0.33 [0.01, 8.11]
**Subtotal (95% CI)**		**247**		**246**	**1.4%**	**0.33 [0.01, 8.11]**
Total events	0		1			
Heterogeneity: Not applicable						
Test for overall effect: Z = 0.68 (P = 0.50)						
**11.38.3 New onset joint sprain ('medically significant conditions'): Cervarix vs. Havrix: hepatitis A vaccine (females, age 9-15; follow-up until 12 months)**						
HPV-013: Cervarix vs. Havrix (f, 10-14; 12)	1	1035	5	1032	3.0%	0.20 [0.02, 1.70]
HPV-033: Cervarix vs. Havrix (f, 9-15; 7)	0	160	1	161	1.4%	0.34 [0.01, 8.17]
**Subtotal (95% CI)**		**1195**		**1193**	**4.5%**	**0.23 [0.04, 1.39]**
Total events	1		6			
Heterogeneity: Tau² = 0.00; Chi² = 0.07, df = 1 (P = 0.79); I² = 0%						
Test for overall effect: Z = 1.60 (P = 0.11)						
**11.38.4 New onset joint sprain ('medically significant conditions'): Cervarix vs. Havrix: hepatitis A vaccine (females, age 15-25; follow-up until 48 months)**						
HPV-008: Cervarix vs. Havrix (f, 15-25; 48)	9	9319	12	9325	12.8%	0.75 [0.32, 1.78]
**Subtotal (95% CI)**		**9319**		**9325**	**12.8%**	**0.75 [0.32, 1.78]**
Total events	9		12			
Heterogeneity: Not applicable						
Test for overall effect: Z = 0.65 (P = 0.51)						
**11.38.5 New onset joint sprain ('medically significant conditions'): Cervarix vs. Twinrix: hepatitis A and B vaccine (females, age 8-15; follow-up until 12 months)**						
HPV-029: Cervarix vs. Twinrix (f, 8-15; 12)	0	270	2	271	1.6%	0.20 [0.01, 4.16]
**Subtotal (95% CI)**		**270**		**271**	**1.6%**	**0.20 [0.01, 4.16]**
Total events	0		2			
Heterogeneity: Not applicable						
Test for overall effect: Z = 1.04 (P = 0.30)						
**11.38.6 New onset joint sprain ('new medical history'): Gardasil vs. carrier solution (females and males, age 9-16; follow-up until 18 months)**						
V501-018: Gardasil vs. carrier solu. (f&m,9-16;18)	25	1184	12	597	16.7%	1.05 [0.53, 2.08]
**Subtotal (95% CI)**		**1184**		**597**	**16.7%**	**1.05 [0.53, 2.08]**
Total events	25		12			
Heterogeneity: Not applicable						
Test for overall effect: Z = 0.14 (P = 0.89)						
**11.38.7 New onset joint sprain ('new medical history'): Gardasil vs. AAHS (females, age 15-26; follow-up until 45 months)**						
V501-013: Gardasil vs. AAHS (f, 16-24; 45)	24	2723	21	2732	19.3%	1.15 [0.64, 2.05]
V501-015: Gardasil vs. AAHS (f, 15-26; 36)	22	6087	9	6080	14.5%	2.44 [1.13, 5.30]
**Subtotal (95% CI)**		**8810**		**8812**	**33.8%**	**1.60 [0.77, 3.34]**
Total events	46		30			
Heterogeneity: Tau² = 0.16; Chi² = 2.33, df = 1 (P = 0.13); I² = 57%						
Test for overall effect: Z = 1.25 (P = 0.21)						
**11.38.8 New onset joint sprain ('new medical history'): Gardasil vs. AAHS (females, age 21-46; from 0-7 months)**						
V501-019: Gardasil vs. AAHS (f, 21-46; 48)	7	1911	2	1908	5.2%	3.49 [0.73, 16.80]
**Subtotal (95% CI)**		**1911**		**1908**	**5.2%**	**3.49 [0.73, 16.80]**
Total events	7		2			
Heterogeneity: Not applicable						
Test for overall effect: Z = 1.56 (P = 0.12)						
**11.38.9 New onset joint sprain ('new medical history'): Gardasil vs. AAHS (females, age 21-46; from 7-48 months)**						
V501-019: Gardasil vs. AAHS (f, 21-46; 48)	28	1911	19	1908	0.0%	1.47 [0.82, 2.63]
**Subtotal (95% CI)**		**0**		**0**		**Not estimable**
Total events	0		0			
Heterogeneity: Not applicable						
Test for overall effect: Not applicable						
**11.38.10 New onset joint sprain ('new medical history'): Gardasil vs. AAHS (males, age 15-27; from 0-7 months)**						
V501-020: Gardasil vs. AAHS (m, 15-27; 36)	10	2032	2	2032	5.6%	5.00 [1.10, 22.79]
**Subtotal (95% CI)**		**2032**		**2032**	**5.6%**	**5.00 [1.10, 22.79]**
Total events	10		2			
Heterogeneity: Not applicable						
Test for overall effect: Z = 2.08 (P = 0.04)						
**11.38.11 New onset joint sprain ('new medical history'): Gardasil vs. AAHS (males, age 15-27; from 7-36 months)**						
V501-020: Gardasil vs. AAHS (m, 15-27; 36)	9	2032	3	2033	0.0%	3.00 [0.81, 11.07]
**Subtotal (95% CI)**		**0**		**0**		**Not estimable**
Total events	0		0			
Heterogeneity: Not applicable						
Test for overall effect: Not applicable						
**11.38.12 New onset joint sprain ('new medical history'): HPV 16 vaccine vs. AAHS (females, age 16-25; from 0-7 months)**						
V501-005: HPV 16 vaccine vs. AAHS (f, 16-25; 48)	13	1204	16	1205	15.6%	0.81 [0.39, 1.68]
**Subtotal (95% CI)**		**1204**		**1205**	**15.6%**	**0.81 [0.39, 1.68]**
Total events	13		16			
Heterogeneity: Not applicable						
Test for overall effect: Z = 0.56 (P = 0.58)						
**11.38.13 New onset joint sprain ('new medical history'): HPV 16 vaccine vs. AAHS (females, age 16-25; from 7-48 months)**						
V501-005: HPV 16 vaccine vs. AAHS (f, 16-25; 48)	20	1204	18	1205	0.0%	1.11 [0.59, 2.09]
**Subtotal (95% CI)**		**0**		**0**		**Not estimable**
Total events	0		0			
Heterogeneity: Not applicable						
Test for overall effect: Not applicable						
**11.38.14 New onset joint sprain ('new medical history'): Gardasil 9 vs. saline placebo (females, age 12-26; follow-up until 7 months)**						
V503-006: Gardasil 9 vs. saline (f, 12-26;7)	1	618	0	306	1.4%	1.49 [0.06, 36.42]
**Subtotal (95% CI)**		**618**		**306**	**1.4%**	**1.49 [0.06, 36.42]**
Total events	1		0			
Heterogeneity: Not applicable						
Test for overall effect: Z = 0.24 (P = 0.81)						
**Total (95% CI)**		**27165**		**26272**	**100.0%**	**1.18 [0.80, 1.75]**
Total events	113		83			
Heterogeneity: Tau² = 0.12; Chi² = 16.38, df = 12 (P = 0.17); I² = 27%						
Test for overall effect: Z = 0.83 (P = 0.41)						
Test for subgroup differences: Chi² = 13.40, df = 10 (P = 0.20), I² = 25.4%						

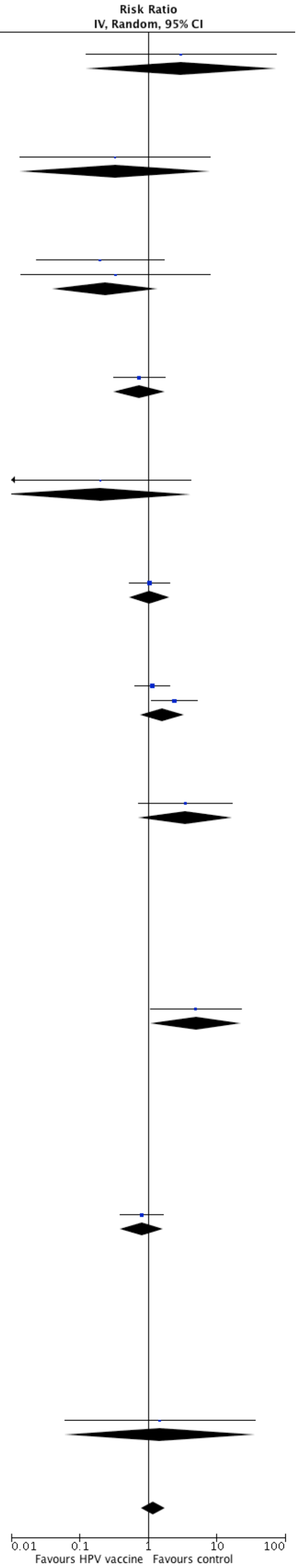

*11.38. Risk ratio for 'medically significant conditions' (GlaxoSmithKline): 0.60 [0.29, 1.22]; risk ratio for ‘new medical history’ (Merck Sharp & Dohme): 1.45 [0.94, 2.24]; risk ratio for the follow-up periods for the trials V501-005, V501-019 and V501-020: 1.40 [0.94, 2.11]. The trials V501-005, V501-019 and V501-020 split the reporting of new onset diseases into the vaccination period and the follow-up period. To avoid double counting of participants in the total risk ratio estimate, we only included the new onset diseases reported in the vaccination period for the trials V501-005, V501-019 and V501-020.

11.39. New onset diseases most increased by the HPV vaccines (‘new medical history’*) - ‘amenorrhoea’: intention to treat analysis

Study or Subgroup	HPV vaccine Events	HPV vaccine Total	Control Events	Control Total	Weight	Risk Ratio IV, Random, 95% CI
**11.39.1 New onset amenorrhoea ('medically significant conditions'): Cervarix vs. Al(OH)3 (females, age 24-72; follow-up until 36 months)**						
HPV-015: Cervarix vs. Al(OH)3 (f, 24-72; 36)	2	2882	5	2871	1.9%	0.40 [0.08, 2.05]
**Subtotal (95% CI)**		**2882**		**2871**	**1.9%**	**0.40 [0.08, 2.05]**
Total events	2		5			
Heterogeneity: Not applicable						
Test for overall effect: Z = 1.10 (P = 0.27)						
**11.39.2 New onset amenorrhoea ('medically significant conditions'): Cervarix vs. Aimmugen: hepatitis A vaccine (females, age 22-29; follow-up until 12 months)**						
HPV-063: Cervarix vs. Aimmugen (f, 22-29; 12)	0	375	1	377	0.5%	0.34 [0.01, 8.20]
**Subtotal (95% CI)**		**375**		**377**	**0.5%**	**0.34 [0.01, 8.20]**
Total events	0		1			
Heterogeneity: Not applicable						
Test for overall effect: Z = 0.67 (P = 0.50)						
**11.39.3 New onset amenorrhoea ('medically significant conditions'): Cervarix vs. Havrix: hepatitis A vaccine (females, age 15-25; follow-up until 48 months)**						
HPV-008: Cervarix vs. Havrix (f, 15-25; 48)	18	9319	25	9319	11.3%	0.72 [0.39, 1.32]
**Subtotal (95% CI)**		**9319**		**9319**	**11.3%**	**0.72 [0.39, 1.32]**
Total events	18		25			
Heterogeneity: Not applicable						
Test for overall effect: Z = 1.06 (P = 0.29)						
**11.39.4 New onset amenorrhoea ('new medical history'): Gardasil vs. carrier solution (females and males, age 9-16; follow-up until 18 months)**						
V501-018: Gardasil vs. carrier solu. (f&m,9-16;18)	7	1184	0	597	0.6%	7.57 [0.43, 132.31]
**Subtotal (95% CI)**		**1184**		**597**	**0.6%**	**7.57 [0.43, 132.31]**
Total events	7		0			
Heterogeneity: Not applicable						
Test for overall effect: Z = 1.39 (P = 0.17)						
**11.39.5 New onset amenorrhoea ('new medical history'): Gardasil vs. AAHS (females, age 15-26; follow-up until 45 months)**						
V501-013: Gardasil vs. AAHS (f, 16-24; 45)	118	2723	97	2732	32.2%	1.22 [0.94, 1.59]
V501-015: Gardasil vs. AAHS (f, 15-26; 36)	220	6087	213	6080	41.1%	1.03 [0.86, 1.24]
**Subtotal (95% CI)**		**8810**		**8812**	**73.3%**	**1.09 [0.93, 1.28]**
Total events	338		310			
Heterogeneity: Tau² = 0.00; Chi² = 1.05, df = 1 (P = 0.31); I² = 5%						
Test for overall effect: Z = 1.11 (P = 0.27)						
**11.39.6 New onset amenorrhoea ('new medical history'): Gardasil vs. AAHS (females, age 21-46; from 0-7 months)**						
V501-019: Gardasil vs. AAHS (f, 21-46; 48)	8	1911	2	1908	2.1%	3.99 [0.85, 18.78]
**Subtotal (95% CI)**		**1911**		**1908**	**2.1%**	**3.99 [0.85, 18.78]**
Total events	8		2			
Heterogeneity: Not applicable						
Test for overall effect: Z = 1.75 (P = 0.08)						
**11.39.7 New onset amenorrhoea ('new medical history'): Gardasil vs. AAHS (females, age 21-46; from 7-48 months)**						
V501-019: Gardasil vs. AAHS (f, 21-46; 48)	16	1911	16	1908	0.0%	1.00 [0.50, 1.99]
**Subtotal (95% CI)**		**0**		**0**		**Not estimable**
Total events	0		0			
Heterogeneity: Not applicable						
Test for overall effect: Not applicable						
**11.39.8 New onset amenorrhoea ('new medical history'): HPV 16 vaccine vs. AAHS (females, age 16-25; from 7-48 months)**						
V501-005: HPV 16 vaccine vs. AAHS (f, 16-25; 48)	21	1204	16	1205	10.2%	1.31 [0.69, 2.50]
**Subtotal (95% CI)**		**1204**		**1205**	**10.2%**	**1.31 [0.69, 2.50]**
Total events	21		16			
Heterogeneity: Not applicable						
Test for overall effect: Z = 0.83 (P = 0.41)						
**Total (95% CI)**		**25685**		**25089**	**100.0%**	**1.09 [0.87, 1.37]**
Total events	394		359			
Heterogeneity: Tau² = 0.02; Chi² = 9.60, df = 7 (P = 0.21); I² = 27%						
Test for overall effect: Z = 0.74 (P = 0.46)						
Test for subgroup differences: Chi² = 8.55, df = 6 (P = 0.20), I² = 29.8%						

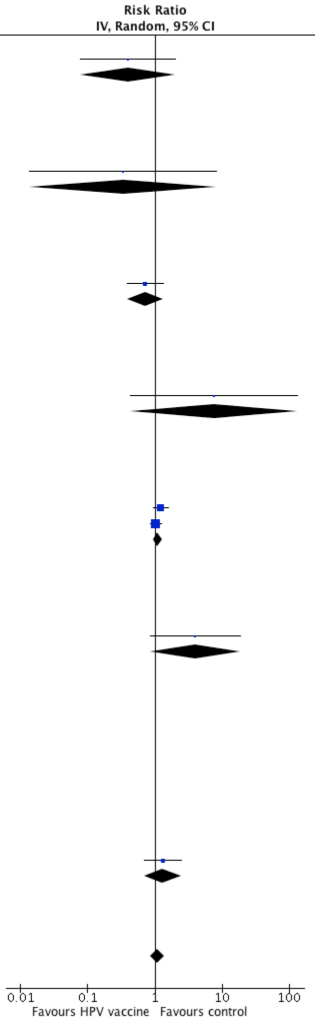

*11.39. Risk ratio for 'medically significant conditions’ (GlaxoSmithKline): 0.66 [0.38, 1.15]; risk ratio for ‘new medical history’ (Merck Sharp & Dohme): 1.17 [0.93, 1.48]; risk ratio for the follow-up period for the trial V501-019: 1.00 [0.50, 1.99]. The trial V501-019 split the reporting of new onset diseases into the vaccination period and the follow-up period. To avoid double counting of participants in the total risk ratio estimate, we only included the new onset diseases reported in the vaccination period for the trial V501-019.

11.40. New onset diseases most decreased by the HPV vaccines (‘medically significant conditions’*) - ‘gynaecological chlamydia infection’: intention to treat analysis

See analysis 11.28.

11.41. New onset diseases most decreased by the HPV vaccines ('medically significant conditions'*) - 'cystitis': intention to treat analysis

Study or Subgroup	HPV vaccine Events	HPV vaccine Total	Control Events	Control Total	Weight	Risk Ratio IV, Random, 95% CI
**11.41.1 New onset cystitis ('medically significant conditions'): Cervarix vs. Al(OH)3 (females, age 24-72; follow-up until 36 months)**						
HPV-015: Cervarix vs. Al(OH)3 (f, 24-72; 36)	7	2882	17	2871	3.0%	0.41 [0.17, 0.99]
**Subtotal (95% CI)**		**2882**		**2871**	**3.0%**	**0.41 [0.17, 0.99]**
Total events	7		17			
Heterogeneity: Not applicable						
Test for overall effect: Z = 1.99 (P = 0.05)						
**11.41.2 New onset cystitis ('medically significant conditions'): Cervarix vs. Aimmugen: hepatitis A vaccine (females, age 22-29; follow-up until 12 months)**						
HPV-063: Cervarix vs. Aimmugen (f, 22-29; 12)	4	375	9	377	1.7%	0.45 [0.14, 1.44]
**Subtotal (95% CI)**		**375**		**377**	**1.7%**	**0.45 [0.14, 1.44]**
Total events	4		9			
Heterogeneity: Not applicable						
Test for overall effect: Z = 1.35 (P = 0.18)						
**11.41.3 New onset cystitis ('medically significant conditions'): Cervarix vs. Engerix: hepatitis B vaccine (females and males, age 9-15; follow-up until 12 months)**						
HPV-030: Cervarix vs. Engerix (f, 9-15; 12)	0	247	1	246	0.2%	0.33 [0.01, 8.11]
**Subtotal (95% CI)**		**247**		**246**	**0.2%**	**0.33 [0.01, 8.11]**
Total events	0		1			
Heterogeneity: Not applicable						
Test for overall effect: Z = 0.68 (P = 0.50)						
**11.41.4 New onset cystitis ('medically significant conditions'): Cervarix vs. Havrix: hepatitis A vaccine (females, age 15-25; follow-up until 48 months)**						
HPV-008: Cervarix vs. Havrix (f, 15-25; 48)	29	9319	37	9325	9.2%	0.78 [0.48, 1.27]
**Subtotal (95% CI)**		**9319**		**9325**	**9.2%**	**0.78 [0.48, 1.27]**
Total events	29		37			
Heterogeneity: Not applicable						
Test for overall effect: Z = 0.98 (P = 0.33)						
**11.41.5 New onset cystitis ('medically significant conditions'): Cervarix vs. Twinrix: hepatitis A and B vaccine (females, age 8-15; follow-up until 12 months)**						
HPV-029: Cervarix vs. Twinrix (f, 8-15; 12)	1	270	0	271	0.2%	3.01 [0.12, 73.59]
**Subtotal (95% CI)**		**270**		**271**	**0.2%**	**3.01 [0.12, 73.59]**
Total events	1		0			
Heterogeneity: Not applicable						
Test for overall effect: Z = 0.68 (P = 0.50)						
**11.41.6 New onset cystitis ('new medical history'): Gardasil vs. carrier solution (females and males, age 9-16; follow-up until 18 months)**						
V501-018: Gardasil vs. carrier solu. (f&m,9-16;18)	2	1184	2	597	0.6%	0.50 [0.07, 3.57]
**Subtotal (95% CI)**		**1184**		**597**	**0.6%**	**0.50 [0.07, 3.57]**
Total events	2		2			
Heterogeneity: Not applicable						
Test for overall effect: Z = 0.69 (P = 0.49)						
**11.41.7 New onset cystitis ('new medical history'): Gardasil vs. AAHS (females, age 15-26; follow-up until 45 months)**						
V501-013: Gardasil vs. AAHS (f, 16-24; 45)	106	2723	94	2732	24.5%	1.13 [0.86, 1.49]
V501-015: Gardasil vs. AAHS (f, 15-26; 36)	300	6087	307	6080	50.6%	0.98 [0.84, 1.14]
**Subtotal (95% CI)**		**8810**		**8812**	**75.1%**	**1.01 [0.88, 1.16]**
Total events	406		401			
Heterogeneity: $Tau^2 = 0.00$; $Chi^2 = 0.85$, df = 1 (P = 0.36); $I^2 = 0\%$						
Test for overall effect: Z = 0.17 (P = 0.86)						
**11.41.8 New onset cystitis ('new medical history'): Gardasil vs. AAHS (females, age 21-46; from 0-7 months)**						
V501-019: Gardasil vs. AAHS (f, 21-46; 48)	14	1911	12	1908	3.9%	1.16 [0.54, 2.51]
**Subtotal (95% CI)**		**1911**		**1908**	**3.9%**	**1.16 [0.54, 2.51]**
Total events	14		12			
Heterogeneity: Not applicable						
Test for overall effect: Z = 0.39 (P = 0.70)						
**11.41.9 New onset cystitis ('new medical history'): Gardasil vs. AAHS (females, age 21-46; from 7-48 months)**						
V501-019: Gardasil vs. AAHS (f, 21-46; 48)	28	1911	27	1908	0.0%	1.04 [0.61, 1.75]
**Subtotal (95% CI)**		**0**		**0**		**Not estimable**
Total events	0		0			
Heterogeneity: Not applicable						
Test for overall effect: Not applicable						
**11.41.10 New onset cystitis ('new medical history'): Gardasil vs. AAHS (males, age 15-27; from 0-7 months)**						
V501-020: Gardasil vs. AAHS (m, 15-27; 36)	1	2032	0	2032	0.2%	3.00 [0.12, 73.60]
**Subtotal (95% CI)**		**2032**		**2032**	**0.2%**	**3.00 [0.12, 73.60]**
Total events	1		0			
Heterogeneity: Not applicable						
Test for overall effect: Z = 0.67 (P = 0.50)						
**11.41.11 New onset cystitis ('new medical history'): Gardasil vs. AAHS (males, age 15-27; from 7-36 months)**						
V501-020: Gardasil vs. AAHS (m, 15-27; 36)	2	2032	0	2033	0.0%	5.00 [0.24, 104.13]
**Subtotal (95% CI)**		**0**		**0**		**Not estimable**
Total events	0		0			
Heterogeneity: Not applicable						
Test for overall effect: Not applicable						
**11.41.12 New onset cystitis ('new medical history'): HPV 16 vaccine vs. AAHS (females, age 16-25; from 0-7 months)**						
V501-005: HPV 16 vaccine vs. AAHS (f, 16-25; 48)	13	1204	19	1205	4.6%	0.68 [0.34, 1.38]
**Subtotal (95% CI)**		**1204**		**1205**	**4.6%**	**0.68 [0.34, 1.38]**
Total events	13		19			
Heterogeneity: Not applicable						
Test for overall effect: Z = 1.06 (P = 0.29)						
**11.41.13 New onset cystitis ('new medical history'): HPV 16 vaccine vs. AAHS (females, age 16-25; from 7-48 months)**						
V501-005: HPV 16 vaccine vs. AAHS (f, 16-25; 48)	11	1204	16	1205	0.0%	0.69 [0.32, 1.48]
**Subtotal (95% CI)**		**0**		**0**		**Not estimable**
Total events	0		0			
Heterogeneity: Not applicable						
Test for overall effect: Not applicable						
**11.41.14 New onset cystitis ('new medical history'): Gardasil 9 vs. saline placebo (females, age 12-26; follow-up until 7 months)**						
V503-006: Gardasil 9 vs. saline (f, 12-26;7)	3	618	4	306	1.1%	0.37 [0.08, 1.65]
**Subtotal (95% CI)**		**618**		**306**	**1.1%**	**0.37 [0.08, 1.65]**
Total events	3		4			
Heterogeneity: Not applicable						
Test for overall effect: Z = 1.30 (P = 0.19)						
**Total (95% CI)**		**28852**		**27950**	**100.0%**	**0.93 [0.80, 1.09]**
Total events	480		502			
Heterogeneity: $Tau^2 = 0.01$; $Chi^2 = 11.85$, df = 11 (P = 0.38); $I^2 = 7\%$						
Test for overall effect: Z = 0.87 (P = 0.38)						
Test for subgroup differences: $Chi^2 = 11.00$, df = 10 (P = 0.36), $I^2 = 9.1\%$						

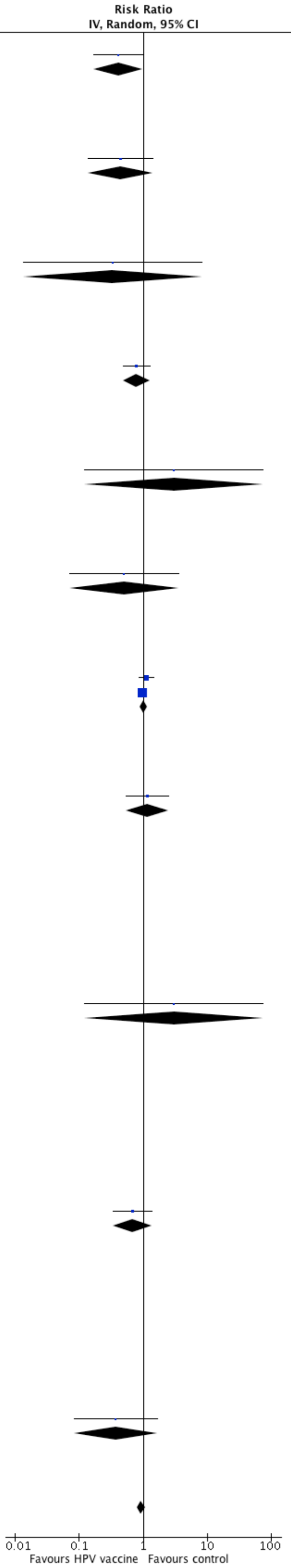

*11.41. Risk ratio for 'medically significant conditions' (GlaxoSmithKline): **0.65 [0.44, 0.96]**; risk ratio for 'new medical history' (Merck Sharp & Dohme): 0.99 [0.87, 1.13]; risk ratio for the follow-up periods for the trials V501-005, V501-019 and V501-020: 0.94 [0.61, 1.44]. The trials V501-005, V501-019 and V501-020 split the reporting of new onset diseases into the vaccination period and the follow-up period. To avoid double counting of participants in the total risk ratio estimate, we only included the new onset diseases reported in the vaccination period for the trials V501-005, V501-019 and V501-020.

11.42. New onset diseases most decreased by the HPV vaccines (‘medically significant conditions’*) - ‘type 2 diabetes mellitus’: intention to treat analysis

Study or Subgroup	HPV vaccine Events	HPV vaccine Total	Control Events	Control Total	Weight	Risk Ratio IV, Random, 95% CI
**11.42.1 New onset type 2 diabetes mellitus ('medically significant conditions'): Cervarix vs. Al(OH)3 (females, age 24-72; follow-up until 36 months)**						
HPV-015: Cervarix vs. Al(OH)3 (f, 24-72; 36)	24	2882	44	2871	63.8%	0.54 [0.33, 0.89]
**Subtotal (95% CI)**		**2882**		**2871**	**63.8%**	**0.54 [0.33, 0.89]**
Total events	24		44			
Heterogeneity: Not applicable						
Test for overall effect: Z = 2.42 (P = 0.02)						
**11.42.2 New onset type 2 diabetes mellitus ('medically significant conditions'): Cervarix vs. Havrix: hepatitis A vaccine (females, age 15-25; follow-up until 48 months)**						
HPV-008: Cervarix vs. Havrix (f, 15-25; 48)	3	9319	2	9325	17.7%	1.50 [0.25, 8.98]
**Subtotal (95% CI)**		**9319**		**9325**	**17.7%**	**1.50 [0.25, 8.98]**
Total events	3		2			
Heterogeneity: Not applicable						
Test for overall effect: Z = 0.44 (P = 0.66)						
**11.42.3 New onset type 2 diabetes mellitus ('new medical history'): Gardasil vs. AAHS (females, age 21-46; from 0-7 months)**						
V501-019: Gardasil vs. AAHS (f, 21-46; 48)	1	1911	0	1908	6.5%	3.00 [0.12, 73.48]
**Subtotal (95% CI)**		**1911**		**1908**	**6.5%**	**3.00 [0.12, 73.48]**
Total events	1		0			
Heterogeneity: Not applicable						
Test for overall effect: Z = 0.67 (P = 0.50)						
**11.42.4 New onset type 2 diabetes mellitus ('new medical history'): Gardasil vs. AAHS (females, age 21-46; from 7-48 months)**						
V501-019: Gardasil vs. AAHS (f, 21-46; 48)	2	1911	1	1908	0.0%	2.00 [0.18, 22.00]
**Subtotal (95% CI)**		**0**		**0**		**Not estimable**
Total events	0		0			
Heterogeneity: Not applicable						
Test for overall effect: Not applicable						
**11.42.5 New onset type 2 diabetes mellitus ('new medical history'): Gardasil vs. AAHS (males, age 15-27; from 7-36 months)**						
V501-020: Gardasil vs. AAHS (m, 15-27; 36)	3	2032	1	2033	12.0%	3.00 [0.31, 28.83]
**Subtotal (95% CI)**		**2032**		**2033**	**12.0%**	**3.00 [0.31, 28.83]**
Total events	3		1			
Heterogeneity: Not applicable						
Test for overall effect: Z = 0.95 (P = 0.34)						
**Total (95% CI)**		**16144**		**16137**	**100.0%**	**0.89 [0.38, 2.09]**
Total events	31		47			
Heterogeneity: Tau² = 0.23; Chi² = 3.98, df = 3 (P = 0.26); I² = 25%						
Test for overall effect: Z = 0.26 (P = 0.79)						
Test for subgroup differences: Chi² = 3.98, df = 3 (P = 0.26), I² = 24.6%						

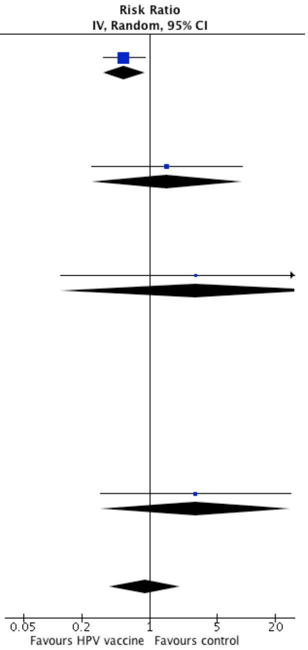

*11.42. Risk ratio for 'medically significant conditions’ (GlaxoSmithKline): 0.62 [0.32, 1.20]; risk ratio for ‘new medical history’ (Merck Sharp & Dohme): 3.00 [0.47, 19.02]; risk ratio for the follow-up period for the trial V501-019: 2.00 [0.18, 22.00]. The trial V501-019 split the reporting of new onset diseases into the vaccination period and the follow-up period. To avoid double counting of participants in the total risk ratio estimate, we only included the new onset diseases reported in the vaccination period for the trial V501-019.

11.43. New onset diseases most decreased by the HPV vaccines ('new medical history'*) - 'vaginal infection': intention to treat analysis

Study or Subgroup	HPV vaccine Events	HPV vaccine Total	Control Events	Control Total	Weight	Risk Ratio IV, Random, 95% CI
**11.43.1 New onset vaginal infection ('new medical history'): Gardasil vs. carrier solution (females and males, age 9-16; follow-up until 18 months)**						
V501-018: Gardasil vs. carrier solu. (f&m,9-16;18)	3	1184	1	597	0.4%	1.51 [0.16, 14.51]
**Subtotal (95% CI)**		**1184**		**597**	**0.4%**	**1.51 [0.16, 14.51]**
Total events	3		1			
Heterogeneity: Not applicable						
Test for overall effect: Z = 0.36 (P = 0.72)						
**11.43.2 New onset vaginal infection ('new medical history'): Gardasil vs. AAHS (females, age 15-26; follow-up until 45 months)**						
V501-013: Gardasil vs. AAHS (f, 16-24; 45)	134	2723	156	2732	37.1%	0.86 [0.69, 1.08]
V501-015: Gardasil vs. AAHS (f, 15-26; 36)	225	6087	259	6080	61.2%	0.87 [0.73, 1.03]
**Subtotal (95% CI)**		**8810**		**8812**	**98.3%**	**0.87 [0.75, 0.99]**
Total events	359		415			
Heterogeneity: $Tau^2 = 0.00$; $Chi^2 = 0.00$, df = 1 (P = 0.96); $I^2 = 0\%$						
Test for overall effect: Z = 2.05 (P = 0.04)						
**11.43.3 New onset vaginal infection ('new medical history'): Gardasil vs. AAHS (females, age 21-46; from 0-7 months)**						
V501-019: Gardasil vs. AAHS (f, 21-46; 48)	6	1911	4	1908	1.2%	1.50 [0.42, 5.30]
**Subtotal (95% CI)**		**1911**		**1908**	**1.2%**	**1.50 [0.42, 5.30]**
Total events	6		4			
Heterogeneity: Not applicable						
Test for overall effect: Z = 0.63 (P = 0.53)						
**11.43.4 New onset vaginal infection ('new medical history'): Gardasil vs. AAHS (females, age 21-46; from 7-48 months)**						
V501-019: Gardasil vs. AAHS (f, 21-46; 48)	8	1911	13	1908	0.0%	0.61 [0.26, 1.48]
**Subtotal (95% CI)**		**0**		**0**		**Not estimable**
Total events	0		0			
Heterogeneity: Not applicable						
Test for overall effect: Not applicable						
**11.43.5 New onset vaginal infection ('new medical history'): Gardasil 9 vs. saline placebo (females, age 12-26; follow-up until 7 months)**						
V503-006: Gardasil 9 vs. saline (f, 12-26;7)	1	618	0	306	0.2%	1.49 [0.06, 36.42]
**Subtotal (95% CI)**		**618**		**306**	**0.2%**	**1.49 [0.06, 36.42]**
Total events	1		0			
Heterogeneity: Not applicable						
Test for overall effect: Z = 0.24 (P = 0.81)						
**Total (95% CI)**		**12523**		**11623**	**100.0%**	**0.87 [0.76, 1.00]**
Total events	369		420			
Heterogeneity: $Tau^2 = 0.00$; $Chi^2 = 1.05$, df = 4 (P = 0.90); $I^2 = 0\%$						
Test for overall effect: Z = 1.93 (P = 0.05)						
Test for subgroup differences: $Chi^2 = 1.05$, df = 3 (P = 0.79), $I^2 = 0\%$						

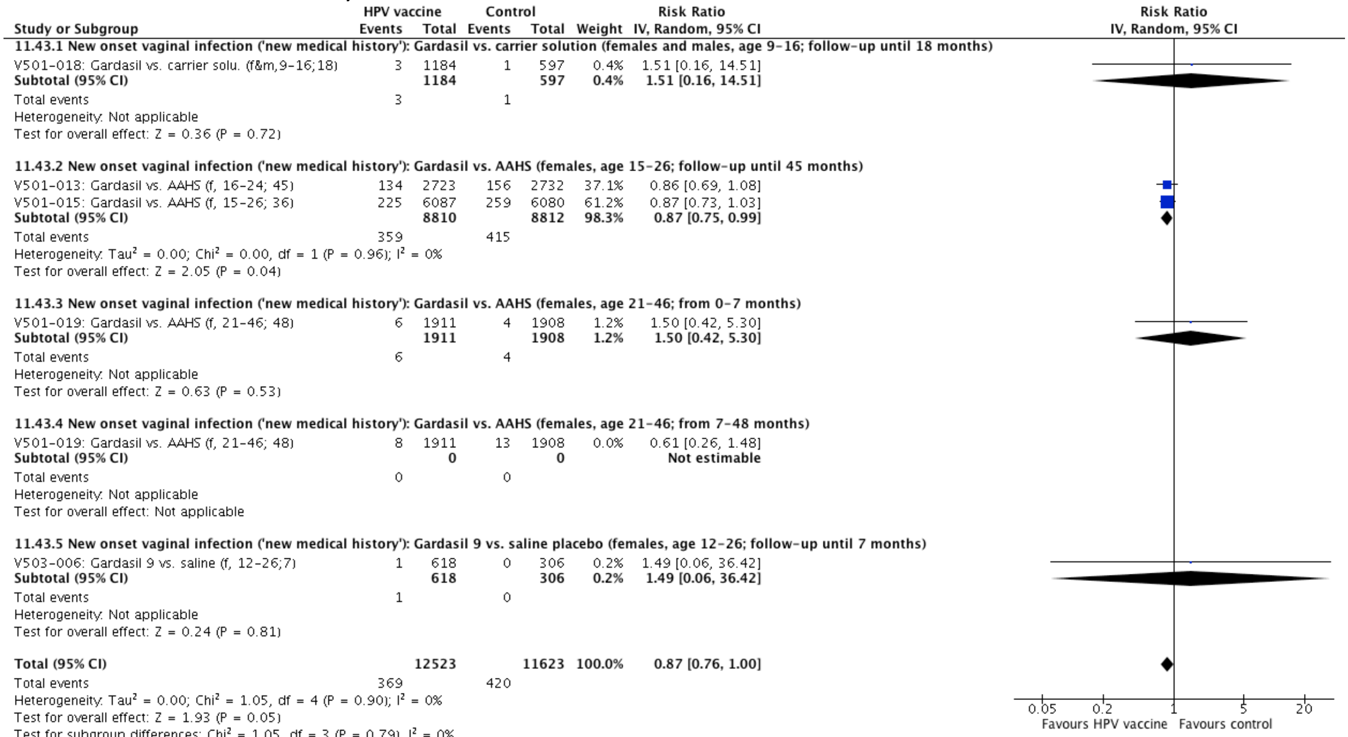

*11.43. Risk ratio for 'medically significant conditions' (GlaxoSmithKline): not applicable; risk ratio for 'new medical history' (Merck Sharp & Dohme): **0.87 [0.76, 1.00]**; risk ratio for the follow-up period for the trial V501-019: 0.61 [0.26, 1.48]. The trial V501-019 split the reporting of new onset diseases into the vaccination period and the follow-up period. To avoid double counting of participants in the total risk ratio estimate, we only included the new onset diseases reported in the vaccination period for the trial V501-019.

11.44. New onset diseases most decreased by the HPV vaccines (‘new medical history’*) - ‘vaginal candidiasis’: intention to treat analysis
See analysis 11.31.

11.45. New onset diseases most decreased by the HPV vaccines (‘new medical history’*) - ‘urinary tract infection’: intention to treat analysis
See analysis 11.33.

12. General harms

12.1. General harms (‘solicited and unsolicited’ and ‘systemic adverse events’*): intention to treat analysis

Study or Subgroup	HPV vaccine Events	HPV vaccine Total	Control Events	Control Total	Weight	Risk Ratio IV, Random, 95% CI
**12.1.1 General harms ('solicited' and 'unsolicited'): Cervarix vs. Al(OH)3 (females, age 9-17; follow-up until 12 months)**						
HPV-058: Cervarix vs. Al(OH)3 (f, 9-17; 12)	294	374	275	376	5.3%	1.07 [0.99, 1.17]
**Subtotal (95% CI)**		**374**		**376**	**5.3%**	**1.07 [0.99, 1.17]**
Total events	294		275			
Heterogeneity: Not applicable						
Test for overall effect: Z = 1.75 (P = 0.08)						
**12.1.2 General harms ('solicited' and 'unsolicited'): Cervarix vs. Al(OH)3 (females, age 15-26; follow-up until 27 months)**						
HPV-001: Cervarix vs. Al(OH)3 (f, 15-26; 27)	458	560	462	553	6.3%	0.98 [0.93, 1.03]
HPV-038: Cervarix vs. Al(OH)3 (f, 15-25; 7)	136	149	55	76	3.2%	1.26 [1.09, 1.46]
**Subtotal (95% CI)**		**709**		**629**	**9.6%**	**1.10 [0.86, 1.41]**
Total events	594		517			
Heterogeneity: Tau^2 = 0.03; Chi^2 = 10.00, df = 1 (P = 0.002); I^2 = 90%						
Test for overall effect: Z = 0.76 (P = 0.45)						
**12.1.3 General harms ('solicited' and 'unsolicited'): Cervarix vs. Al(OH)3 (females, age 18-35; follow-up until 12 months)**						
HPV-003: Cervarix vs. Al(OH)3 (f, 18-30; 12)	28	31	29	30	3.6%	0.93 [0.82, 1.07]
HPV-031: Cervarix vs. Al(OH)3 (f, 18-35; 7)	25	176	20	178	0.4%	1.26 [0.73, 2.19]
HPV-035: Cervarix vs. Al(OH)3 (f, 18-35; 7)	136	150	114	150	4.5%	1.19 [1.08, 1.32]
**Subtotal (95% CI)**		**357**		**358**	**8.5%**	**1.08 [0.88, 1.33]**
Total events	189		163			
Heterogeneity: Tau^2 = 0.02; Chi^2 = 8.35, df = 2 (P = 0.02); I^2 = 76%						
Test for overall effect: Z = 0.74 (P = 0.46)						
**12.1.4 General harms ('solicited' and 'unsolicited'): Cervarix vs. Al(OH)3 (females, age 24-72; follow-up until 36 months)**						
HPV-015: Cervarix vs. Al(OH)3 (f, 24-72; 36)	2111	2882	1970	2871	7.0%	1.07 [1.03, 1.10]
**Subtotal (95% CI)**		**2882**		**2871**	**7.0%**	**1.07 [1.03, 1.10]**
Total events	2111		1970			
Heterogeneity: Not applicable						
Test for overall effect: Z = 3.86 (P = 0.0001)						
**12.1.5 General harms ('solicited' and 'unsolicited'): Cervarix vs. Engerix: hepatitis B vaccine (females and males, age 12-16; follow-up until 72 months)**						
HPV-030: Cervarix vs. Engerix (f, 9-15; 12)	202	247	195	246	5.1%	1.03 [0.95, 1.13]
HPV-040: Cervarix vs. Engerix (f & m, 12-16; 72)	484	604	676	1028	6.1%	1.22 [1.15, 1.29]
**Subtotal (95% CI)**		**851**		**1274**	**11.3%**	**1.12 [0.96, 1.32]**
Total events	686		871			
Heterogeneity: Tau^2 = 0.01; Chi^2 = 9.62, df = 1 (P = 0.002); I^2 = 90%						
Test for overall effect: Z = 1.41 (P = 0.16)						
**12.1.6 General harms ('solicited' and 'unsolicited'): Cervarix vs. Engerix: hepatitis B vaccine (females, age 26-46; follow-up until 12 months)**						
HPV-069: Cervarix vs. Engerix (f, 26-46; 12)	202	606	148	606	2.5%	1.36 [1.14, 1.63]
**Subtotal (95% CI)**		**606**		**606**	**2.5%**	**1.36 [1.14, 1.63]**
Total events	202		148			
Heterogeneity: Not applicable						
Test for overall effect: Z = 3.39 (P = 0.0007)						
**12.1.7 General harms ('solicited' and 'unsolicited'): Cervarix vs. Havrix: hepatitis A vaccine (females, age 9-15; follow-up until 12 months)**						
HPV-013: Cervarix vs. Havrix (f, 10-14; 12)	577	1035	468	1032	5.1%	1.23 [1.13, 1.34]
HPV-033: Cervarix vs. Havrix (f, 9-15; 7)	103	160	96	161	2.7%	1.08 [0.91, 1.28]
**Subtotal (95% CI)**		**1195**		**1193**	**7.8%**	**1.18 [1.04, 1.33]**
Total events	680		564			
Heterogeneity: Tau^2 = 0.00; Chi^2 = 1.76, df = 1 (P = 0.19); I^2 = 43%						
Test for overall effect: Z = 2.68 (P = 0.007)						
**12.1.8 General harms ('solicited' and 'unsolicited'): Cervarix vs. Havrix: hepatitis A vaccine (females, age 15-25; follow-up until 48 months)**						
HPV-008: Cervarix vs. Havrix (f, 15-25; 48)	2626	9319	2531	9325	6.6%	1.04 [0.99, 1.09]
**Subtotal (95% CI)**		**9319**		**9325**	**6.6%**	**1.04 [0.99, 1.09]**
Total events	2626		2531			
Heterogeneity: Not applicable						
Test for overall effect: Z = 1.58 (P = 0.11)						
**12.1.9 General harms ('solicited' and 'unsolicited'): Cervarix vs. Twinrix: hepatitis A and B vaccine (females, age 8-15; follow-up until 12 months)**						
HPV-029: Cervarix vs. Twinrix (f, 8-15; 12)	217	270	191	271	4.8%	1.14 [1.03, 1.26]
**Subtotal (95% CI)**		**270**		**271**	**4.8%**	**1.14 [1.03, 1.26]**
Total events	217		191			
Heterogeneity: Not applicable						
Test for overall effect: Z = 2.65 (P = 0.008)						
**12.1.10 General harms ('systemic adverse events'): Gardasil vs. carrier solution (females and males, age 9-16; follow-up until 18 months)**						
V501-018: Gardasil vs. carrier solu. (f&m,9-16;18)	541	1184	260	597	4.3%	1.05 [0.94, 1.17]
**Subtotal (95% CI)**		**1184**		**597**	**4.3%**	**1.05 [0.94, 1.17]**
Total events	541		260			
Heterogeneity: Not applicable						
Test for overall effect: Z = 0.85 (P = 0.39)						
**12.1.11 General harms ('systemic adverse events'): Gardasil vs. AAHS (females, age 15-26; follow-up until 45 months)**						
V501-013: Gardasil vs. AAHS (f, 16-24; 45)	1746	2723	1701	2732	6.8%	1.03 [0.99, 1.07]
V501-015: Gardasil vs. AAHS (f, 15-26; 36)	448	6087	453	6080	3.8%	0.99 [0.87, 1.12]
**Subtotal (95% CI)**		**8810**		**8812**	**10.6%**	**1.03 [0.99, 1.07]**
Total events	2194		2154			
Heterogeneity: Tau^2 = 0.00; Chi^2 = 0.38, df = 1 (P = 0.54); I^2 = 0%						
Test for overall effect: Z = 1.30 (P = 0.20)						
**12.1.12 General harms ('systemic adverse events'): Gardasil vs. AAHS (females, age 21-46; follow-up until 48 months)**						
V501-019: Gardasil vs. AAHS (f, 21-46; 48)	1121	1911	1135	1908	6.4%	0.99 [0.94, 1.04]
**Subtotal (95% CI)**		**1911**		**1908**	**6.4%**	**0.99 [0.94, 1.04]**
Total events	1121		1135			
Heterogeneity: Not applicable						
Test for overall effect: Z = 0.52 (P = 0.60)						
**12.1.13 General harms ('systemic adverse events'): Gardasil vs. AAHS (males, age 15-27; follow-up until 36 months)**						
V501-020: Gardasil vs. AAHS (m, 15-27; 36)	616	2032	613	2033	4.9%	1.01 [0.92, 1.10]
**Subtotal (95% CI)**		**2032**		**2033**	**4.9%**	**1.01 [0.92, 1.10]**
Total events	616		613			
Heterogeneity: Not applicable						
Test for overall effect: Z = 0.11 (P = 0.91)						
**12.1.14 General harms ('systemic adverse events'): Gardasil 9 vs. saline placebo (females, age 12-26; follow-up until 7 months)**						
V503-006: Gardasil 9 vs. saline (f, 12-26;7)	374	618	177	306	4.2%	1.05 [0.93, 1.17]
**Subtotal (95% CI)**		**618**		**306**	**4.2%**	**1.05 [0.93, 1.17]**
Total events	374		177			
Heterogeneity: Not applicable						
Test for overall effect: Z = 0.77 (P = 0.44)						
**12.1.15 General harms ('systemic adverse events'): HPV 16 vaccine vs. AAHS (females, age 16-25; follow-up until 48 months)**						
V501-005: HPV 16 vaccine vs. AAHS (f, 16-25; 48)	803	1204	825	1205	6.3%	0.97 [0.92, 1.03]
**Subtotal (95% CI)**		**1204**		**1205**	**6.3%**	**0.97 [0.92, 1.03]**
Total events	803		825			
Heterogeneity: Not applicable						
Test for overall effect: Z = 0.93 (P = 0.35)						
**Total (95% CI)**		**32322**		**31764**	**100.0%**	**1.07 [1.03, 1.11]**
Total events	13248		12394			
Heterogeneity: Tau^2 = 0.00; Chi^2 = 86.35, df = 20 (P < 0.00001); I^2 = 77%						
Test for overall effect: Z = 3.74 (P = 0.0002)						
Test for subgroup differences: Chi^2 = 30.88, df = 14 (P = 0.006), I^2 = 54.7%						

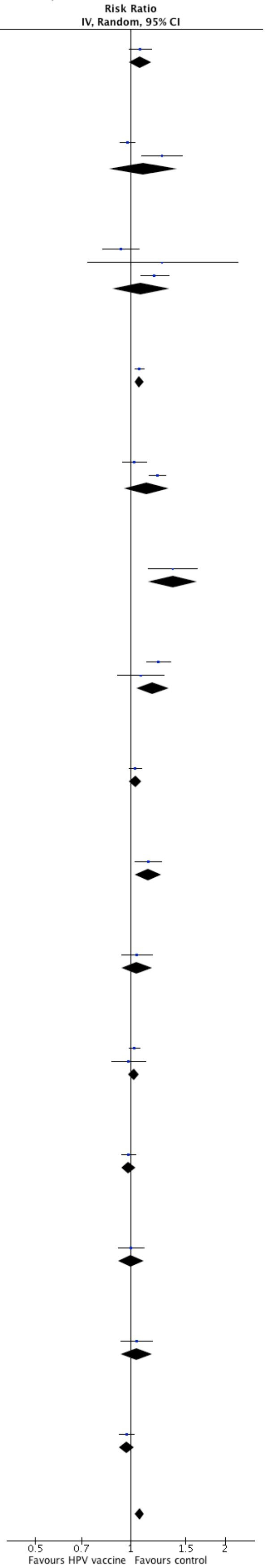

*12.1. Risk ratio for ‘solicited and solicited’ (GlaxoSmithKline): **1.11 [1.06, 1.16]**; risk ratio for ‘systemic adverse events’ (Merck Sharp & Dohme): 1.01 [0.98, 1.03]. The total numbers of participants with general harms in GlaxoSmithKline studies were reported as ‘solicited [SGAE] and unsolicited [UGAE]’ combined.

12.2. General harms (‘solicited,’ ‘unsolicited’ and ‘systemic adverse events’*) reported within the MedDRA system organ class ‘blood and lymphatic system disorders (10005329)': intention to treat analysis

Study or Subgroup	HPV vaccine Events	HPV vaccine Total	Control Events	Control Total	Weight	Risk Ratio IV, Random, 95% CI
**12.2.1 General blood and lymphatic system disorders ('unsolicited'): Cervarix vs. Al(OH)3 (females, age 18-30; follow-up until 12 months)**						
HPV-003: Cervarix vs. Al(OH)3 (f, 18-30; 12)	1	31	1	30	10.0%	0.97 [0.06, 14.78]
**Subtotal (95% CI)**		**31**		**30**	**10.0%**	**0.97 [0.06, 14.78]**
Total events	1		1			
Heterogeneity: Not applicable						
Test for overall effect: Z = 0.02 (P = 0.98)						
**12.2.2 General blood and lymphatic system disorders ('unsolicited'): Cervarix vs. Engerix: hepatitis B vaccine (females and males, age 9-16; follow-up until 72 months)**						
HPV-030: Cervarix vs. Engerix (f, 9-15; 12)	1	247	0	246	7.3%	2.99 [0.12, 72.99]
HPV-040: Cervarix vs. Engerix (f & m, 12-16; 72)	0	1908	2	3128	8.1%	0.33 [0.02, 6.82]
**Subtotal (95% CI)**		**2155**		**3374**	**15.4%**	**0.94 [0.10, 8.45]**
Total events	1		2			
Heterogeneity: Tau² = 0.00; Chi² = 0.97, df = 1 (P = 0.33); I² = 0%						
Test for overall effect: Z = 0.06 (P = 0.95)						
**12.2.3 General blood and lymphatic system disorders ('unsolicited'): Cervarix vs. Twinrix: hepatitis A and B vaccine (females, age 8-15; follow-up until 12 months)**						
HPV-029: Cervarix vs. Twinrix (f, 8-15; 12)	0	270	2	271	8.1%	0.20 [0.01, 4.16]
**Subtotal (95% CI)**		**270**		**271**	**8.1%**	**0.20 [0.01, 4.16]**
Total events	0		2			
Heterogeneity: Not applicable						
Test for overall effect: Z = 1.04 (P = 0.30)						
**12.2.4 General blood and lymphatic system disorders ('systemic adverse events'): Gardasil vs. carrier solution (females and males, age 9-16; follow-up until 18 months)**						
V501-018: Gardasil vs. carrier solu. (f&m,9-16;18)	6	1184	1	597	16.7%	3.03 [0.37, 25.07]
**Subtotal (95% CI)**		**1184**		**597**	**16.7%**	**3.03 [0.37, 25.07]**
Total events	6		1			
Heterogeneity: Not applicable						
Test for overall effect: Z = 1.03 (P = 0.30)						
**12.2.5 General blood and lymphatic system disorders ('systemic adverse events'): Gardasil vs. AAHS (males, age 15-27; follow-up until 36 months)**						
V501-020: Gardasil vs. AAHS (m, 15-27; 36)	7	2032	2	2033	30.3%	3.50 [0.73, 16.84]
**Subtotal (95% CI)**		**2032**		**2033**	**30.3%**	**3.50 [0.73, 16.84]**
Total events	7		2			
Heterogeneity: Not applicable						
Test for overall effect: Z = 1.56 (P = 0.12)						
**12.2.6 General blood and lymphatic system disorders ('systemic adverse events'): Gardasil 9 vs. saline placebo (females, age 12-26; follow-up until 7 months)**						
V503-006: Gardasil 9 vs. saline (f, 12-26;7)	2	618	2	306	19.5%	0.50 [0.07, 3.50]
**Subtotal (95% CI)**		**618**		**306**	**19.5%**	**0.50 [0.07, 3.50]**
Total events	2		2			
Heterogeneity: Not applicable						
Test for overall effect: Z = 0.70 (P = 0.48)						
**Total (95% CI)**		**6290**		**6611**	**100.0%**	**1.33 [0.56, 3.15]**
Total events	17		10			
Heterogeneity: Tau² = 0.00; Chi² = 5.63, df = 6 (P = 0.47); I² = 0%						
Test for overall effect: Z = 0.64 (P = 0.52)						
Test for subgroup differences: Chi² = 4.67, df = 5 (P = 0.46), I² = 0%						

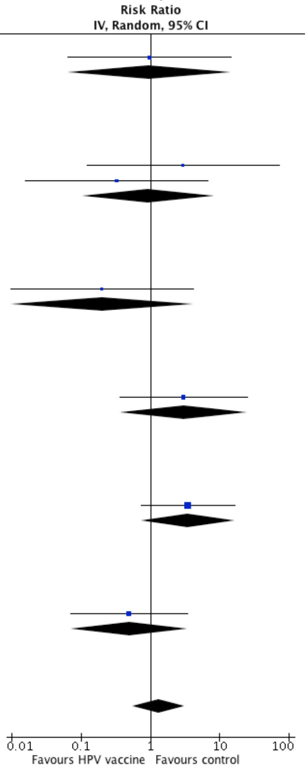

*12.2. Risk ratio for ‘solicited’ (GlaxoSmithKline): not applicable; risk ratio for ‘unsolicited’ (GlaxoSmithKline): 0.65 [0.15, 2.89]; risk ratio for ‘systemic adverse events’ (Merck Sharp & Dohme): 1.85 [0.55, 6.25].

12.3. General harms (‘solicited,’ ‘unsolicited’ and ‘systemic adverse events’*) reported within the MedDRA system organ class ‘cardiac disorders (10007541)': intention to treat analysis

Study or Subgroup	HPV vaccine Events	HPV vaccine Total	Control Events	Control Total	Weight	Risk Ratio IV, Random, 95% CI
**12.3.1 General cardiac disorders ('unsolicited'): Cervarix vs. Al(OH)3 (females, age 18–35; follow-up until 7 months)**						
HPV-031: Cervarix vs. Al(OH)3 (f, 18–35; 7)	1	176	0	178	14.4%	3.03 [0.12, 73.97]
**Subtotal (95% CI)**		**176**		**178**	**14.4%**	**3.03 [0.12, 73.97]**
Total events	1		0			
Heterogeneity: Not applicable						
Test for overall effect: Z = 0.68 (P = 0.50)						
**12.3.2 General cardiac disorders ('unsolicited'): Cervarix vs. Twinrix: hepatitis A and B vaccine (females, age 8–15; follow-up until 12 months)**						
HPV-029: Cervarix vs. Twinrix (f, 8–15; 12)	2	270	0	271	16.0%	5.02 [0.24, 104.04]
**Subtotal (95% CI)**		**270**		**271**	**16.0%**	**5.02 [0.24, 104.04]**
Total events	2		0			
Heterogeneity: Not applicable						
Test for overall effect: Z = 1.04 (P = 0.30)						
**12.3.3 General cardiac disorders ('systemic adverse events'): Gardasil vs. carrier solution (females and males, age 9–16; follow-up until 18 months)**						
V501-018: Gardasil vs. carrier solu. (f&m,9–16;18)	1	1184	0	597	14.4%	1.51 [0.06, 37.11]
**Subtotal (95% CI)**		**1184**		**597**	**14.4%**	**1.51 [0.06, 37.11]**
Total events	1		0			
Heterogeneity: Not applicable						
Test for overall effect: Z = 0.25 (P = 0.80)						
**12.3.4 General cardiac disorders ('systemic adverse events'): Gardasil vs. AAHS (males, age 15–27; follow-up until 36 months)**						
V501-020: Gardasil vs. AAHS (m, 15–27; 36)	0	2032	3	2033	16.8%	0.14 [0.01, 2.77]
**Subtotal (95% CI)**		**2032**		**2033**	**16.8%**	**0.14 [0.01, 2.77]**
Total events	0		3			
Heterogeneity: Not applicable						
Test for overall effect: Z = 1.29 (P = 0.20)						
**12.3.5 General cardiac disorders ('systemic adverse events'): Gardasil 9 vs. saline placebo (females, age 12–26; follow-up until 7 months)**						
V503-006: Gardasil 9 vs. saline (f, 12–26;7)	2	618	2	306	38.5%	0.50 [0.07, 3.50]
**Subtotal (95% CI)**		**618**		**306**	**38.5%**	**0.50 [0.07, 3.50]**
Total events	2		2			
Heterogeneity: Not applicable						
Test for overall effect: Z = 0.70 (P = 0.48)						
**Total (95% CI)**		**4280**		**3385**	**100.0%**	**0.89 [0.26, 2.99]**
Total events	6		5			
Heterogeneity: Tau² = 0.00; Chi² = 3.73, df = 4 (P = 0.44); I² = 0%						
Test for overall effect: Z = 0.19 (P = 0.85)						
Test for subgroup differences: Chi² = 3.73, df = 4 (P = 0.44), I² = 0%						

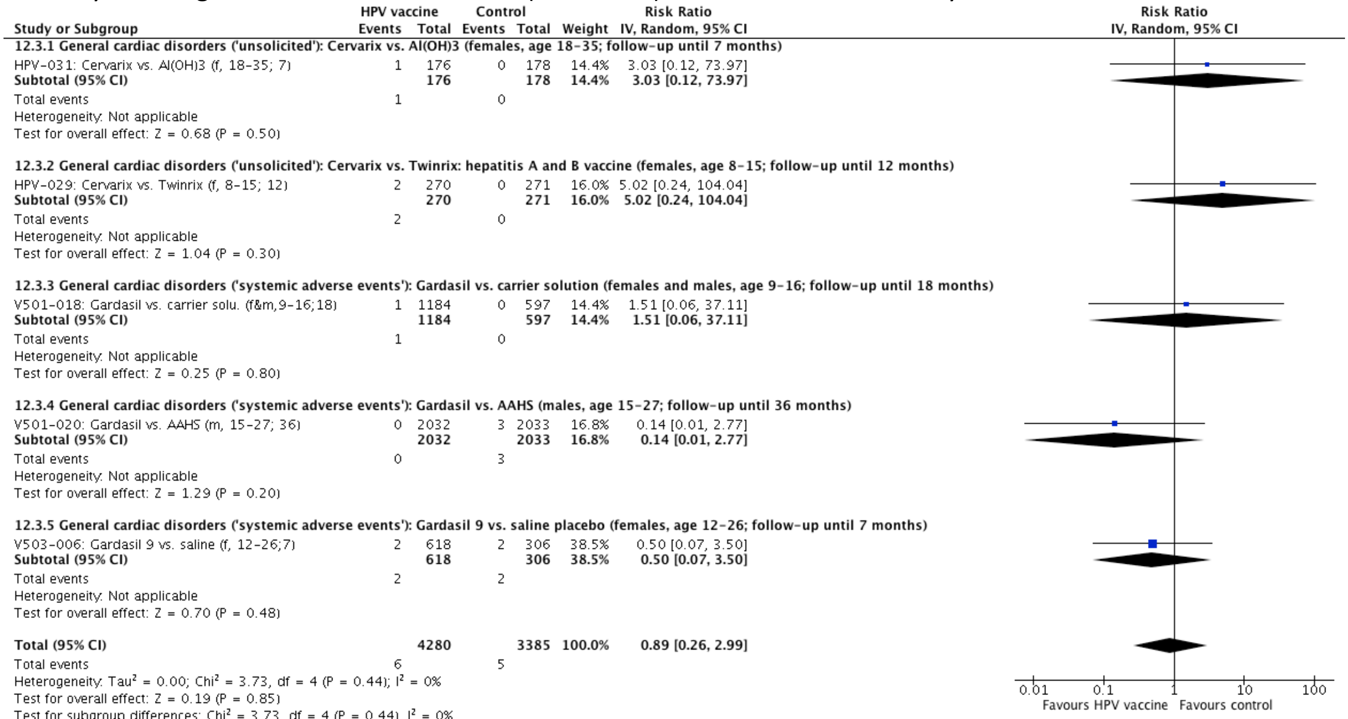

*12.3. Risk ratio for ‘solicited’ (GlaxoSmithKline): not applicable; risk ratio for ‘unsolicited’ (GlaxoSmithKline): 3.95 [0.44, 35.64]; risk ratio for ‘systemic adverse events’ (Merck Sharp & Dohme): 0.46 [0.11, 1.98].

12.4. General harms (‘solicited,’ ‘unsolicited’ and ‘systemic adverse events’*) reported within the MedDRA system organ class ‘congenital familial and genetic disorders (10010331)': intention to treat analysis

Study or Subgroup	HPV vaccine Events	HPV vaccine Total	Control Events	Control Total	Weight	Risk Ratio IV, Random, 95% CI	Risk Ratio IV, Random, 95% CI
**12.4.1 General congenital familial and genetic disorders ('unsolicited'): Cervarix vs. Engerix: hepatitis B vaccine (females and males, age 12-16; follow-up until 72 months)**							
HPV-040: Cervarix vs. Engerix (f & m, 12-16; 72)	0	1908	1	3128	100.0%	0.55 [0.02, 13.40]	
**Subtotal (95% CI)**		**1908**		**3128**	**100.0%**	**0.55 [0.02, 13.40]**	
Total events	0		1				
Heterogeneity: Not applicable							
Test for overall effect: Z = 0.37 (P = 0.71)							
**Total (95% CI)**		**1908**		**3128**	**100.0%**	**0.55 [0.02, 13.40]**	
Total events	0		1				
Heterogeneity: Not applicable							
Test for overall effect: Z = 0.37 (P = 0.71)							
Test for subgroup differences: Not applicable							

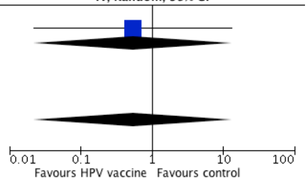

*12.4. Risk ratio for ‘solicited’ (GlaxoSmithKline): not applicable; risk ratio for ‘unsolicited’ (GlaxoSmithKline): 0.55 [0.02, 13.40]; risk ratio for ‘systemic adverse events’ (Merck Sharp & Dohme): not applicable.

12.5. General harms ('solicited,' 'unsolicited' and 'systemic adverse events'*) reported within the MedDRA system organ class 'ear and labyrinth disorders (10013993)': intention to treat analysis

Study or Subgroup	HPV vaccine Events	HPV vaccine Total	Control Events	Control Total	Weight	Risk Ratio IV, Random, 95% CI
**12.5.1 General ear and labyrinth disorders ('unsolicited'): Cervarix vs. Al(OH)3 (females, age 15-25; follow-up until 7 months)**						
HPV-038: Cervarix vs. Al(OH)3 (f, 15-25; 7)	0	149	1	76	1.8%	0.17 [0.01, 4.15]
**Subtotal (95% CI)**		**149**		**76**	**1.8%**	**0.17 [0.01, 4.15]**
Total events	0		1			
Heterogeneity: Not applicable						
Test for overall effect: Z = 1.09 (P = 0.28)						
**12.5.2 General ear and labyrinth disorders ('unsolicited'): Cervarix vs. Engerix: hepatitis B vaccine (females and males, age 9-16; follow-up until 72 months)**						
HPV-030: Cervarix vs. Engerix (f, 9-15; 12)	1	247	4	246	3.9%	0.25 [0.03, 2.21]
HPV-040: Cervarix vs. Engerix (f & m, 12-16; 72)	1	1908	4	3128	3.9%	0.41 [0.05, 3.66]
**Subtotal (95% CI)**		**2155**		**3374**	**7.8%**	**0.32 [0.07, 1.50]**
Total events	2		8			
Heterogeneity: Tau² = 0.00; Chi² = 0.10, df = 1 (P = 0.75); I² = 0%						
Test for overall effect: Z = 1.45 (P = 0.15)						
**12.5.3 General ear and labyrinth disorders ('unsolicited'): Cervarix vs. Engerix: hepatitis B vaccine (females, age 26-46; follow-up until 12 months)**						
HPV-069: Cervarix vs. Engerix (f, 26-46; 12)	0	606	1	606	1.8%	0.33 [0.01, 8.17]
**Subtotal (95% CI)**		**606**		**606**	**1.8%**	**0.33 [0.01, 8.17]**
Total events	0		1			
Heterogeneity: Not applicable						
Test for overall effect: Z = 0.67 (P = 0.50)						
**12.5.4 General ear and labyrinth disorders ('unsolicited'): Cervarix vs. Havrix: hepatitis A vaccine (females, age 9-15; follow-up until 7 months)**						
HPV-033: Cervarix vs. Havrix (f, 9-15; 7)	0	160	1	161	1.8%	0.34 [0.01, 8.17]
**Subtotal (95% CI)**		**160**		**161**	**1.8%**	**0.34 [0.01, 8.17]**
Total events	0		1			
Heterogeneity: Not applicable						
Test for overall effect: Z = 0.67 (P = 0.50)						
**12.5.5 General ear and labyrinth disorders ('unsolicited'): Cervarix vs. Twinrix: hepatitis A and B vaccine (females, age 8-15; follow-up until 12 months)**						
HPV-029: Cervarix vs. Twinrix (f, 8-15; 12)	2	270	4	271	6.5%	0.50 [0.09, 2.72]
**Subtotal (95% CI)**		**270**		**271**	**6.5%**	**0.50 [0.09, 2.72]**
Total events	2		4			
Heterogeneity: Not applicable						
Test for overall effect: Z = 0.80 (P = 0.42)						
**12.5.6 General ear and labyrinth disorders ('systemic adverse events'): Gardasil vs. carrier solution (females and males, age 9-16; follow-up until 18 months)**						
V501-018: Gardasil vs. carrier solu. (f&m,9-16;18)	19	1184	7	597	25.2%	1.37 [0.58, 3.24]
**Subtotal (95% CI)**		**1184**		**597**	**25.2%**	**1.37 [0.58, 3.24]**
Total events	19		7			
Heterogeneity: Not applicable						
Test for overall effect: Z = 0.71 (P = 0.48)						
**12.5.7 General ear and labyrinth disorders ('systemic adverse events'): Gardasil vs. AAHS (males, age 15-27; follow-up until 36 months)**						
V501-020: Gardasil vs. AAHS (m, 15-27; 36)	10	2032	6	2033	18.3%	1.67 [0.61, 4.58]
**Subtotal (95% CI)**		**2032**		**2033**	**18.3%**	**1.67 [0.61, 4.58]**
Total events	10		6			
Heterogeneity: Not applicable						
Test for overall effect: Z = 0.99 (P = 0.32)						
**12.5.8 General ear and labyrinth disorders ('systemic adverse events'): Gardasil 9 vs. saline placebo (females, age 12-26; follow-up until 7 months)**						
V503-006: Gardasil 9 vs. saline (f, 12-26;7)	8	618	3	306	10.7%	1.32 [0.35, 4.94]
**Subtotal (95% CI)**		**618**		**306**	**10.7%**	**1.32 [0.35, 4.94]**
Total events	8		3			
Heterogeneity: Not applicable						
Test for overall effect: Z = 0.41 (P = 0.68)						
**12.5.9 General ear and labyrinth disorders ('systemic adverse events'): HPV 16 vaccine vs. AAHS (females, age 16-25; follow-up until 48 months)**						
V501-005: HPV 16 vaccine vs. AAHS (f, 16-25; 48)	13	1204	9	1205	26.0%	1.45 [0.62, 3.37]
**Subtotal (95% CI)**		**1204**		**1205**	**26.0%**	**1.45 [0.62, 3.37]**
Total events	13		9			
Heterogeneity: Not applicable						
Test for overall effect: Z = 0.85 (P = 0.39)						
**Total (95% CI)**		**8378**		**8629**	**100.0%**	**1.10 [0.71, 1.69]**
Total events	54		40			
Heterogeneity: Tau² = 0.00; Chi² = 7.13, df = 9 (P = 0.62); I² = 0%						
Test for overall effect: Z = 0.42 (P = 0.68)						
Test for subgroup differences: Chi² = 7.03, df = 8 (P = 0.53), I² = 0%						

Risk Ratio IV, Random, 95% CI

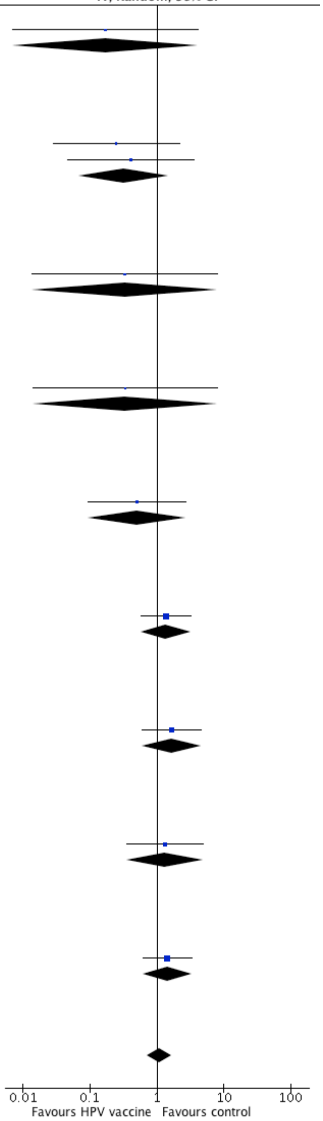

*12.5. Risk ratio for 'solicited' (GlaxoSmithKline): not applicable; risk ratio for 'unsolicited' (GlaxoSmithKline): **0.35 [0.13, 0.93]**; risk ratio for 'systemic adverse events' (Merck Sharp & Dohme): 1.46 [0.91, 2.34].

12.6. General harms (‘solicited,’ ‘unsolicited’ and ‘systemic adverse events’*) reported within the MedDRA system organ class ‘endocrine disorders (10014698)': intention to treat analysis

Study or Subgroup	HPV vaccine Events	HPV vaccine Total	Control Events	Control Total	Weight	Risk Ratio IV, Random, 95% CI
**12.6.1 General endocrine disorders ('unsolicited'): Cervarix vs. Al(OH)3 (females, age 15–25; follow-up until 7 months)**						
HPV-038: Cervarix vs. Al(OH)3 (f, 15-25; 7)	0	149	1	76	50.0%	0.17 [0.01, 4.15]
**Subtotal (95% CI)**		**149**		**76**	**50.0%**	**0.17 [0.01, 4.15]**
Total events	0		1			
Heterogeneity: Not applicable						
Test for overall effect: Z = 1.09 (P = 0.28)						
**12.6.2 General endocrine disorders ('unsolicited'): Cervarix vs. Al(OH)3 (females, age 18–35; follow-up until 7 months)**						
HPV-035: Cervarix vs. Al(OH)3 (f, 18-35; 7)	1	150	0	150	50.0%	3.00 [0.12, 73.06]
**Subtotal (95% CI)**		**150**		**150**	**50.0%**	**3.00 [0.12, 73.06]**
Total events	1		0			
Heterogeneity: Not applicable						
Test for overall effect: Z = 0.67 (P = 0.50)						
**Total (95% CI)**		**299**		**226**	**100.0%**	**0.72 [0.04, 11.85]**
Total events	1		1			
Heterogeneity: $Tau^2 = 1.45$; $Chi^2 = 1.55$, df = 1 (P = 0.21); $I^2 = 35\%$						
Test for overall effect: Z = 0.23 (P = 0.82)						
Test for subgroup differences: $Chi^2 = 1.55$, df = 1 (P = 0.21), $I^2 = 35.4\%$						

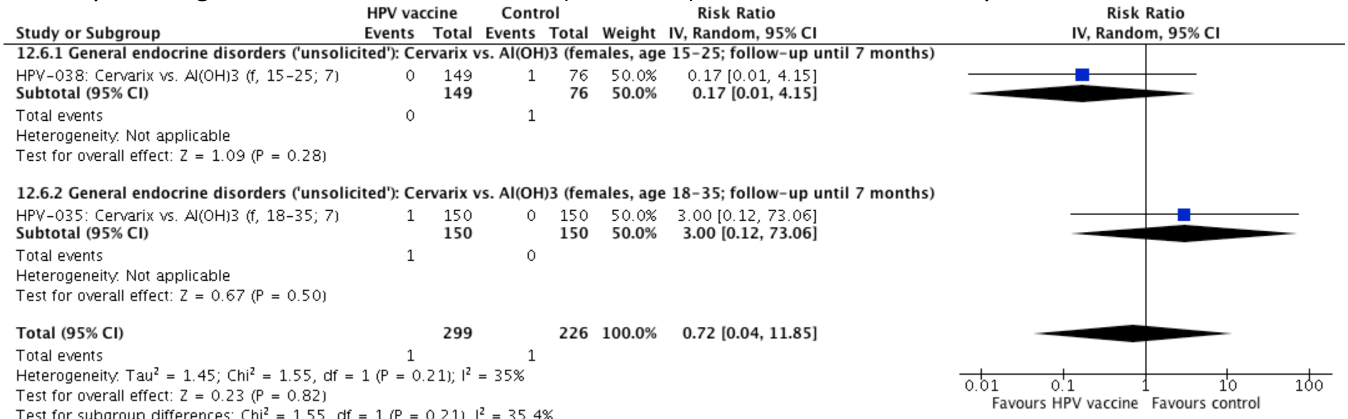

*12.6. Risk ratio for ‘solicited’ (GlaxoSmithKline): not applicable; risk ratio for ‘unsolicited’ (GlaxoSmithKline): 0.72 [0.04, 11.85]; risk ratio for ‘systemic adverse events’ (Merck Sharp & Dohme): not applicable.

12.7. General harms (‘solicited,’ ‘unsolicited’ and ‘systemic adverse events’*) reported within the MedDRA system organ class ‘eye disorders (10015919)': intention to treat analysis

Study or Subgroup	HPV vaccine Events	HPV vaccine Total	Control Events	Control Total	Weight	Risk Ratio IV, Random, 95% CI
**12.7.1 General eye disorders ('unsolicited'): Cervarix vs. Al(OH)3 (females, age 15-25; follow-up until 7 months)**						
HPV-038: Cervarix vs. Al(OH)3 (f, 15-25; 7)	1	149	0	76	2.1%	1.54 [0.06, 37.36]
**Subtotal (95% CI)**		**149**		**76**	**2.1%**	**1.54 [0.06, 37.36]**
Total events	1		0			
Heterogeneity: Not applicable						
Test for overall effect: Z = 0.27 (P = 0.79)						
**12.7.2 General eye disorders ('unsolicited'): Cervarix vs. Al(OH)3 (females, age 18-35; follow-up until 12 months)**						
HPV-003: Cervarix vs. Al(OH)3 (f, 18-30; 12)	0	31	1	30	2.2%	0.32 [0.01, 7.63]
HPV-035: Cervarix vs. Al(OH)3 (f, 18-35; 7)	1	150	1	150	2.8%	1.00 [0.06, 15.84]
**Subtotal (95% CI)**		**181**		**180**	**5.0%**	**0.61 [0.08, 4.91]**
Total events	1		2			
Heterogeneity: Tau² = 0.00; Chi² = 0.28, df = 1 (P = 0.60); I² = 0%						
Test for overall effect: Z = 0.46 (P = 0.64)						
**12.7.3 General eye disorders ('unsolicited'): Cervarix vs. Engerix: hepatitis B vaccine (females and males, age 9-16; follow-up until 72 months)**						
HPV-030: Cervarix vs. Engerix (f, 9-15; 12)	2	247	1	246	3.8%	1.99 [0.18, 21.82]
HPV-040: Cervarix vs. Engerix (f & m, 12-16; 72)	1	1908	2	3128	3.7%	0.82 [0.07, 9.03]
**Subtotal (95% CI)**		**2155**		**3374**	**7.5%**	**1.28 [0.23, 6.97]**
Total events	3		3			
Heterogeneity: Tau² = 0.00; Chi² = 0.26, df = 1 (P = 0.61); I² = 0%						
Test for overall effect: Z = 0.28 (P = 0.78)						
**12.7.4 General eye disorders ('unsolicited'): Cervarix vs. Havrix: hepatitis A vaccine (females, age 9-15; follow-up until 7 months)**						
HPV-033: Cervarix vs. Havrix (f, 9-15; 7)	1	160	1	161	2.8%	1.01 [0.06, 15.95]
**Subtotal (95% CI)**		**160**		**161**	**2.8%**	**1.01 [0.06, 15.95]**
Total events	1		1			
Heterogeneity: Not applicable						
Test for overall effect: Z = 0.00 (P = 1.00)						
**12.7.5 General eye disorders ('unsolicited'): Cervarix vs. Twinrix: hepatitis A and B vaccine (females, age 8-15; follow-up until 12 months)**						
HPV-029: Cervarix vs. Twinrix (f, 8-15; 12)	0	270	1	271	2.1%	0.33 [0.01, 8.18]
**Subtotal (95% CI)**		**270**		**271**	**2.1%**	**0.33 [0.01, 8.18]**
Total events	0		1			
Heterogeneity: Not applicable						
Test for overall effect: Z = 0.67 (P = 0.50)						
**12.7.6 General eye disorders ('systemic adverse events'): Gardasil vs. carrier solution (females and males, age 9-16; follow-up until 18 months)**						
V501-018: Gardasil vs. carrier solu. (f&m,9-16;18)	9	1184	3	597	12.7%	1.51 [0.41, 5.57]
**Subtotal (95% CI)**		**1184**		**597**	**12.7%**	**1.51 [0.41, 5.57]**
Total events	9		3			
Heterogeneity: Not applicable						
Test for overall effect: Z = 0.62 (P = 0.53)						
**12.7.7 General eye disorders ('systemic adverse events'): Gardasil vs. AAHS (males, age 15-27; follow-up until 36 months)**						
V501-020: Gardasil vs. AAHS (m, 15-27; 36)	6	2032	11	2033	21.8%	0.55 [0.20, 1.47]
**Subtotal (95% CI)**		**2032**		**2033**	**21.8%**	**0.55 [0.20, 1.47]**
Total events	6		11			
Heterogeneity: Not applicable						
Test for overall effect: Z = 1.20 (P = 0.23)						
**12.7.8 General eye disorders ('systemic adverse events'): Gardasil 9 vs. saline placebo (females, age 12-26; follow-up until 7 months)**						
V503-006: Gardasil 9 vs. saline (f, 12-26;7)	8	618	2	306	9.0%	1.98 [0.42, 9.27]
**Subtotal (95% CI)**		**618**		**306**	**9.0%**	**1.98 [0.42, 9.27]**
Total events	8		2			
Heterogeneity: Not applicable						
Test for overall effect: Z = 0.87 (P = 0.39)						
**12.7.9 General eye disorders ('systemic adverse events'): HPV 16 vaccine vs. AAHS (females, age 16-25; follow-up until 48 months)**						
V501-005: HPV 16 vaccine vs. AAHS (f, 16-25; 48)	11	1204	16	1205	36.9%	0.69 [0.32, 1.48]
**Subtotal (95% CI)**		**1204**		**1205**	**36.9%**	**0.69 [0.32, 1.48]**
Total events	11		16			
Heterogeneity: Not applicable						
Test for overall effect: Z = 0.96 (P = 0.34)						
**Total (95% CI)**		**7953**		**8203**	**100.0%**	**0.84 [0.53, 1.33]**
Total events	40		39			
Heterogeneity: Tau² = 0.00; Chi² = 4.30, df = 10 (P = 0.93); I² = 0%						
Test for overall effect: Z = 0.74 (P = 0.46)						
Test for subgroup differences: Chi² = 3.76, df = 8 (P = 0.88), I² = 0%						

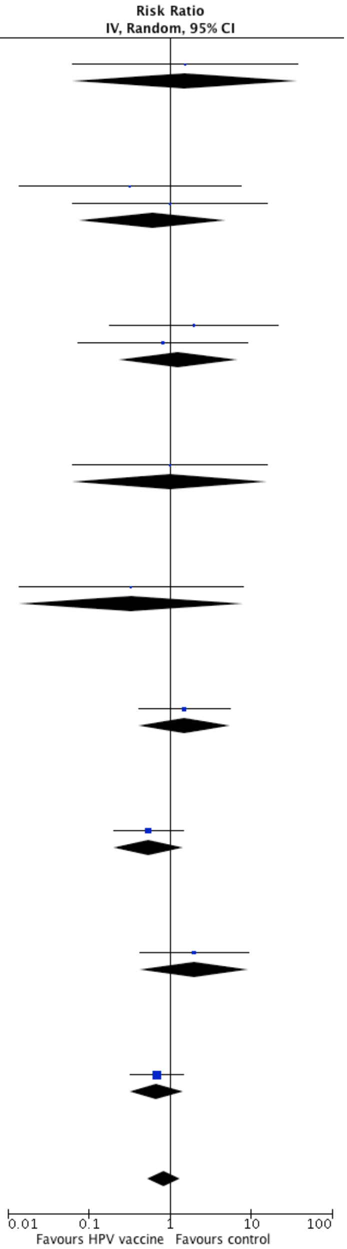

*12.7. Risk ratio for ‘solicited’ (GlaxoSmithKline): not applicable; risk ratio for ‘unsolicited’ (GlaxoSmithKline): 0.90 [0.32, 2.59]; risk ratio for ‘systemic adverse events’ (Merck Sharp & Dohme): 0.81 [0.47, 1.41].

12.8. General harms (‘solicited,’ ‘unsolicited’ and ‘systemic adverse events’*) reported within the MedDRA system organ class ‘gastrointestinal disorders (10017947)': intention to treat analysis

Study or Subgroup	HPV vaccine Events	HPV vaccine Total	Control Events	Control Total	Weight	Risk Ratio IV, Random, 95% CI
**12.8.1 General gastrointestinal disorders ('solicited'): Cervarix vs. Al(OH)3 (females, age 9-17; follow-up until 12 months)**						
HPV-058: Cervarix vs. Al(OH)3 (f, 9-17; 12)	70	374	54	376	3.7%	1.30 [0.94, 1.80]
**Subtotal (95% CI)**		**374**		**376**	**3.7%**	**1.30 [0.94, 1.80]**
Total events	70		54			
Heterogeneity: Not applicable						
Test for overall effect: Z = 1.60 (P = 0.11)						
**12.8.2 General gastrointestinal disorders ('solicited'): Cervarix vs. Al(OH)3 (females, age 15-26; follow-up until 27 months)**						
HPV-001: Cervarix vs. Al(OH)3 (f, 15-26; 27)	259	560	238	553	8.7%	1.07 [0.94, 1.23]
HPV-038: Cervarix vs. Al(OH)3 (f, 15-25; 7)	75	149	17	76	2.3%	2.25 [1.44, 3.52]
**Subtotal (95% CI)**		**709**		**629**	**11.0%**	**1.51 [0.73, 3.10]**
Total events	334		255			
Heterogeneity: Tau^2 = 0.24; Chi^2 = 9.62, df = 1 (P = 0.002); I^2 = 90%						
Test for overall effect: Z = 1.11 (P = 0.27)						
**12.8.3 General gastrointestinal disorders ('solicited'): Cervarix vs. Al(OH)3 (females, age 18-35; follow-up until 12 months)**						
HPV-003: Cervarix vs. Al(OH)3 (f, 18-30; 12)	30	31	18	30	4.1%	1.61 [1.20, 2.18]
HPV-031: Cervarix vs. Al(OH)3 (f, 18-35; 7)	30	176	35	178	2.4%	0.87 [0.56, 1.35]
HPV-035: Cervarix vs. Al(OH)3 (f, 18-35; 7)	60	150	43	150	3.8%	1.40 [1.01, 1.92]
**Subtotal (95% CI)**		**357**		**358**	**10.3%**	**1.29 [0.93, 1.79]**
Total events	120		96			
Heterogeneity: Tau^2 = 0.05; Chi^2 = 5.28, df = 2 (P = 0.07); I^2 = 62%						
Test for overall effect: Z = 1.55 (P = 0.12)						
**12.8.4 General gastrointestinal disorders ('solicited'): Cervarix vs. Al(OH)3 (females, age 24-72; follow-up until 36 months)**						
HPV-015: Cervarix vs. Al(OH)3 (f, 24-72; 36)	911	2882	789	2871	10.5%	1.15 [1.06, 1.25]
**Subtotal (95% CI)**		**2882**		**2871**	**10.5%**	**1.15 [1.06, 1.25]**
Total events	911		789			
Heterogeneity: Not applicable						
Test for overall effect: Z = 3.42 (P = 0.0006)						
**12.8.5 General gastrointestinal disorders ('solicited'): Cervarix vs. Engerix: hepatitis B vaccine (females and males, age 9-15; follow-up until 12 months)**						
HPV-030: Cervarix vs. Engerix (f, 9-15; 12)	94	247	98	246	5.9%	0.96 [0.77, 1.19]
HPV-040: Cervarix vs. Engerix (f & m, 12-16; 72)	137	643	201	1047	6.7%	1.11 [0.91, 1.35]
**Subtotal (95% CI)**		**890**		**1293**	**12.6%**	**1.04 [0.90, 1.20]**
Total events	231		299			
Heterogeneity: Tau^2 = 0.00; Chi^2 = 1.00, df = 1 (P = 0.32); I^2 = 0%						
Test for overall effect: Z = 0.53 (P = 0.60)						
**12.8.6 General gastrointestinal disorders ('solicited'): Cervarix vs. Engerix: hepatitis B vaccine (females, age 26-46; follow-up until 12 months)**						
HPV-069: Cervarix vs. Engerix (f, 26-46; 12)	38	606	27	606	2.0%	1.41 [0.87, 2.28]
**Subtotal (95% CI)**		**606**		**606**	**2.0%**	**1.41 [0.87, 2.28]**
Total events	38		27			
Heterogeneity: Not applicable						
Test for overall effect: Z = 1.39 (P = 0.16)						
**12.8.7 General gastrointestinal disorders ('solicited'): Cervarix vs. Havrix: hepatitis A vaccine (females, age 9-15; follow-up until 12 months)**						
HPV-013: Cervarix vs. Havrix (f, 10-14; 12)	382	1035	346	1032	9.2%	1.10 [0.98, 1.24]
HPV-033: Cervarix vs. Havrix (f, 9-15; 7)	24	160	33	161	2.1%	0.73 [0.45, 1.18]
**Subtotal (95% CI)**		**1195**		**1193**	**11.3%**	**0.96 [0.66, 1.40]**
Total events	406		379			
Heterogeneity: Tau^2 = 0.05; Chi^2 = 2.64, df = 1 (P = 0.10); I^2 = 62%						
Test for overall effect: Z = 0.21 (P = 0.84)						
**12.8.8 General gastrointestinal disorders ('solicited'): Cervarix vs. Havrix: hepatitis A vaccine (females, age 15-25; follow-up until 48 months)**						
HPV-008: Cervarix vs. Havrix (f, 15-25; 48)	1252	9319	1230	9325	10.7%	1.02 [0.95, 1.10]
**Subtotal (95% CI)**		**9319**		**9325**	**10.7%**	**1.02 [0.95, 1.10]**
Total events	1252		1230			
Heterogeneity: Not applicable						
Test for overall effect: Z = 0.49 (P = 0.62)						
**12.8.9 General gastrointestinal disorders ('solicited'): Cervarix vs. Twinrix: hepatitis A and B vaccine (females, age 8-15; follow-up until 12 months)**						
HPV-029: Cervarix vs. Twinrix (f, 8-15; 12)	82	270	97	271	5.4%	0.85 [0.67, 1.08]
**Subtotal (95% CI)**		**270**		**271**	**5.4%**	**0.85 [0.67, 1.08]**
Total events	82		97			
Heterogeneity: Not applicable						
Test for overall effect: Z = 1.34 (P = 0.18)						
**12.8.10 General gastrointestinal disorders ('unsolicited'): Cervarix vs. Al(OH)3 (females, age 9-17; follow-up until 12 months)**						
HPV-058: Cervarix vs. Al(OH)3 (f, 9-17; 12)	7	374	4	376	0.0%	1.76 [0.52, 5.96]
**Subtotal (95% CI)**		**0**		**0**		**Not estimable**
Total events	0		0			
Heterogeneity: Not applicable						
Test for overall effect: Not applicable						
**12.8.11 General gastrointestinal disorders ('unsolicited'): Cervarix vs. Al(OH)3 (females, age 15-25; follow-up until 7 months)**						
HPV-038: Cervarix vs. Al(OH)3 (f, 15-25; 7)	13	149	1	76	0.0%	6.63 [0.88, 49.74]
**Subtotal (95% CI)**		**0**		**0**		**Not estimable**
Total events	0		0			
Heterogeneity: Not applicable						
Test for overall effect: Not applicable						
**12.8.12 General gastrointestinal disorders ('unsolicited'): Cervarix vs. Al(OH)3 (females, age 18-35; follow-up until 12 months)**						
HPV-003: Cervarix vs. Al(OH)3 (f, 18-30; 12)	7	31	1	30	0.0%	6.77 [0.89, 51.80]
HPV-031: Cervarix vs. Al(OH)3 (f, 18-35; 7)	1	176	5	178	0.0%	0.20 [0.02, 1.71]
HPV-035: Cervarix vs. Al(OH)3 (f, 18-35; 7)	19	150	11	150	0.0%	1.73 [0.85, 3.50]
**Subtotal (95% CI)**		**0**		**0**		**Not estimable**
Total events	0		0			
Heterogeneity: Not applicable						
Test for overall effect: Not applicable						
**12.8.13 General gastrointestinal disorders ('unsolicited'): Cervarix vs. Engerix: hepatitis B vaccine (females and males, age 9-16; follow-up until 72 months)**						
HPV-030: Cervarix vs. Engerix (f, 9-15; 12)	14	247	11	246	0.0%	1.27 [0.59, 2.74]
HPV-040: Cervarix vs. Engerix (f & m, 12-16; 72)	4	1908	12	3128	0.0%	0.55 [0.18, 1.69]
**Subtotal (95% CI)**		**0**		**0**		**Not estimable**
Total events	0		0			
Heterogeneity: Not applicable						
Test for overall effect: Not applicable						
**12.8.14 General gastrointestinal disorders ('unsolicited'): Cervarix vs. Engerix: hepatitis B vaccine (females, age 26-46; follow-up until 12 months)**						
HPV-069: Cervarix vs. Engerix (f, 26-46; 12)	1	606	4	606	0.0%	0.25 [0.03, 2.23]
**Subtotal (95% CI)**		**0**		**0**		**Not estimable**
Total events	0		0			
Heterogeneity: Not applicable						
Test for overall effect: Not applicable						
**12.8.15 General gastrointestinal disorders ('unsolicited'): Cervarix vs. Havrix: hepatitis A vaccine (females, age 9-15; follow-up until 7 months)**						
HPV-033: Cervarix vs. Havrix (f, 9-15; 7)	3	160	3	161	0.0%	1.01 [0.21, 4.91]
**Subtotal (95% CI)**		**0**		**0**		**Not estimable**
Total events	0		0			
Heterogeneity: Not applicable						
Test for overall effect: Not applicable						
**12.8.16 General gastrointestinal disorders ('unsolicited'): Cervarix vs. Twinrix: hepatitis A and B vaccine (females, age 8-15; follow-up until 12 months)**						
HPV-029: Cervarix vs. Twinrix (f, 8-15; 12)	6	270	11	271	0.0%	0.55 [0.21, 1.46]
**Subtotal (95% CI)**		**0**		**0**		**Not estimable**
Total events	0		0			
Heterogeneity: Not applicable						
Test for overall effect: Not applicable						
**12.8.17 General gastrointestinal disorders ('systemic adverse events'): Gardasil vs. carrier solution (females and males, age 9-16; follow-up until 18 months)**						
V501-018: Gardasil vs. carrier solu. (f&m,9-16;18)	191	1184	106	597	6.0%	0.91 [0.73, 1.13]
**Subtotal (95% CI)**		**1184**		**597**	**6.0%**	**0.91 [0.73, 1.13]**
Total events	191		106			
Heterogeneity: Not applicable						
Test for overall effect: Z = 0.87 (P = 0.38)						
**12.8.18 General gastrointestinal disorders ('systemic adverse events'): Gardasil vs. AAHS (males, age 15-27; follow-up until 36 months)**						
V501-020: Gardasil vs. AAHS (m, 15-27; 36)	125	2032	120	2033	5.3%	1.04 [0.82, 1.33]
**Subtotal (95% CI)**		**2032**		**2033**	**5.3%**	**1.04 [0.82, 1.33]**
Total events	125		120			
Heterogeneity: Not applicable						
Test for overall effect: Z = 0.33 (P = 0.74)						
**12.8.19 General gastrointestinal disorders ('systemic adverse events'): Gardasil 9 vs. saline placebo (females, age 12-26; follow-up until 7 months)**						
V503-006: Gardasil 9 vs. saline (f, 12-26;7)	108	618	41	306	3.6%	1.30 [0.94, 1.82]
**Subtotal (95% CI)**		**618**		**306**	**3.6%**	**1.30 [0.94, 1.82]**
Total events	108		41			
Heterogeneity: Not applicable						
Test for overall effect: Z = 1.57 (P = 0.12)						
**12.8.20 General gastrointestinal disorders ('systemic adverse events'): HPV 16 vaccine vs. AAHS (females, age 16-25; follow-up until 48 months)**						
V501-005: HPV 16 vaccine vs. AAHS (f, 16-25; 48)	217	1204	242	1205	7.6%	0.90 [0.76, 1.06]
**Subtotal (95% CI)**		**1204**		**1205**	**7.6%**	**0.90 [0.76, 1.06]**
Total events	217		242			
Heterogeneity: Not applicable						
Test for overall effect: Z = 1.29 (P = 0.20)						
**Total (95% CI)**		**21640**		**21063**	**100.0%**	**1.09 [1.01, 1.17]**
Total events	4085		3735			
Heterogeneity: Tau^2 = 0.01; Chi^2 = 44.19, df = 17 (P = 0.0003); I^2 = 62%						
Test for overall effect: Z = 2.16 (P = 0.03)						
Test for subgroup differences: Chi^2 = 21.32, df = 12 (P = 0.05), I^2 = 43.7%						

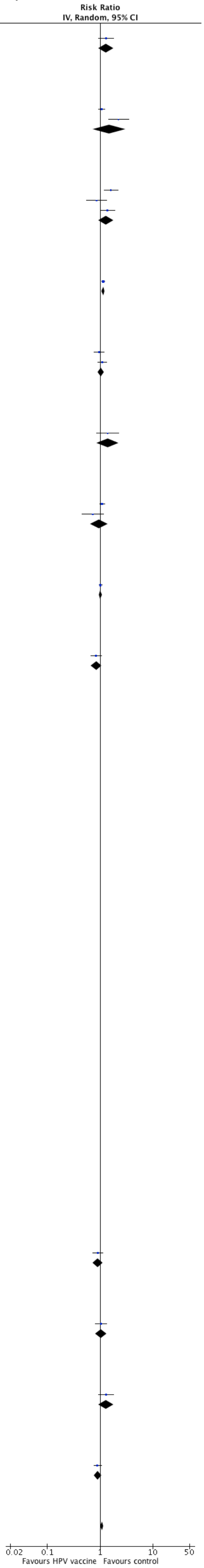

*12.8. Risk ratio for 'solicited' (i.e., ‘gastrointestinal symptoms’) only: **1.12 [1.02, 1.22]**; risk ratio for ‘unsolicited’ (GlaxoSmithKline): 1.05 [0.58, 1.90]; risk ratio for ‘systemic adverse events’ (Merck Sharp & Dohme): 0.98 [0.85, 1.13]. To avoid double counting of participants in the total risk ratio estimate, we excluded the ‘unsolicited’ adverse events from total risk ratio estimate for studies that reported ‘solicited’ adverse events.

12.9. General harms (‘solicited,’ ‘unsolicited’ and ‘systemic adverse events’*) reported within the MedDRA system organ class ‘general disorders and administration site conditions (10018065)': intention to treat analysis

Study or Subgroup	HPV vaccine Events	HPV vaccine Total	Control Events	Control Total	Weight	Risk Ratio IV, Random, 95% CI
**12.9.1 General disorders ('solicited'): Cervarix vs. Al(OH)3 (females, age 9-17; follow-up until 12 months)**						
HPV-058: Cervarix vs. Al(OH)3 (f, 9-17; 12)	223	374	200	376	7.0%	1.12 [0.99, 1.27]
**Subtotal (95% CI)**		**374**		**376**	**7.0%**	**1.12 [0.99, 1.27]**
Total events	223		200			
Heterogeneity: Not applicable						
Test for overall effect: Z = 1.77 (P = 0.08)						
**12.9.2 General disorders ('solicited'): Cervarix vs. Al(OH)3 (females, age 15-26; follow-up until 27 months)**						
HPV-001: Cervarix vs. Al(OH)3 (f, 15-26; 27)	396	560	362	553	8.6%	1.08 [1.00, 1.17]
HPV-038: Cervarix vs. Al(OH)3 (f, 15-25; 7)	110	149	41	76	4.2%	1.37 [1.09, 1.72]
**Subtotal (95% CI)**		**709**		**629**	**12.8%**	**1.19 [0.95, 1.49]**
Total events	506		403			
Heterogeneity: $Tau^2 = 0.02$; $Chi^2 = 3.65$, df = 1 (P = 0.06); $I^2 = 73\%$						
Test for overall effect: Z = 1.47 (P = 0.14)						
**12.9.3 General disorders ('solicited'): Cervarix vs. Al(OH)3 (females, age 18-35; follow-up until 12 months)**						
HPV-031: Cervarix vs. Al(OH)3 (f, 18-35; 7)	135	176	131	178	7.3%	1.04 [0.92, 1.17]
HPV-035: Cervarix vs. Al(OH)3 (f, 18-35; 7)	120	150	98	150	6.5%	1.22 [1.06, 1.41]
**Subtotal (95% CI)**		**326**		**328**	**13.8%**	**1.12 [0.96, 1.32]**
Total events	255		229			
Heterogeneity: $Tau^2 = 0.01$; $Chi^2 = 2.90$, df = 1 (P = 0.09); $I^2 = 66\%$						
Test for overall effect: Z = 1.46 (P = 0.14)						
**12.9.4 General disorders ('solicited'): Cervarix vs. Al(OH)3 (females, age 24-72; follow-up until 36 months)**						
HPV-015: Cervarix vs. Al(OH)3 (f, 24-72; 36)	1424	2882	1164	2871	9.2%	1.22 [1.15, 1.29]
**Subtotal (95% CI)**		**2882**		**2871**	**9.2%**	**1.22 [1.15, 1.29]**
Total events	1424		1164			
Heterogeneity: Not applicable						
Test for overall effect: Z = 6.72 (P < 0.00001)						
**12.9.5 General disorders ('solicited'): Cervarix vs. Engerix: hepatitis B vaccine (females and males, age 9-15; follow-up until 12 months)**						
HPV-030: Cervarix vs. Engerix (f, 9-15; 12)	130	247	140	246	5.9%	0.92 [0.79, 1.09]
HPV-040: Cervarix vs. Engerix (f & m, 12-16; 72)	239	604	496	1028	7.4%	0.82 [0.73, 0.92]
**Subtotal (95% CI)**		**851**		**1274**	**13.3%**	**0.86 [0.77, 0.96]**
Total events	369		636			
Heterogeneity: $Tau^2 = 0.00$; $Chi^2 = 1.40$, df = 1 (P = 0.24); $I^2 = 29\%$						
Test for overall effect: Z = 2.58 (P = 0.010)						
**12.9.6 General disorders ('solicited'): Cervarix vs. Engerix: hepatitis B vaccine (females, age 26-46; follow-up until 12 months)**						
HPV-069: Cervarix vs. Engerix (f, 26-46; 12)	128	606	92	606	3.9%	1.39 [1.09, 1.77]
**Subtotal (95% CI)**		**606**		**606**	**3.9%**	**1.39 [1.09, 1.77]**
Total events	128		92			
Heterogeneity: Not applicable						
Test for overall effect: Z = 2.66 (P = 0.008)						
**12.9.7 General disorders ('solicited'): Cervarix vs. Havrix: hepatitis A vaccine (females, age 9-15; follow-up until 12 months)**						
HPV-013: Cervarix vs. Havrix (f, 10-14; 12)	692	1035	598	1032	9.0%	1.15 [1.08, 1.23]
HPV-033: Cervarix vs. Havrix (f, 9-15; 7)	72	160	58	161	3.4%	1.25 [0.96, 1.63]
**Subtotal (95% CI)**		**1195**		**1193**	**12.4%**	**1.16 [1.09, 1.24]**
Total events	764		656			
Heterogeneity: $Tau^2 = 0.00$; $Chi^2 = 0.32$, df = 1 (P = 0.57); $I^2 = 0\%$						
Test for overall effect: Z = 4.43 (P < 0.00001)						
**12.9.8 General disorders ('solicited'): Cervarix vs. Havrix: hepatitis A vaccine (females, age 15-25; follow-up until 48 months)**						
HPV-008: Cervarix vs. Havrix (f, 15-25; 48)	2156	9319	1994	9325	9.3%	1.08 [1.03, 1.14]
**Subtotal (95% CI)**		**9319**		**9325**	**9.3%**	**1.08 [1.03, 1.14]**
Total events	2156		1994			
Heterogeneity: Not applicable						
Test for overall effect: Z = 2.87 (P = 0.004)						
**12.9.9 General disorders ('solicited'): Cervarix vs. Twinrix: hepatitis A and B vaccine (females, age 8-15; follow-up until 12 months)**						
HPV-029: Cervarix vs. Twinrix (f, 8-15; 12)	161	270	133	271	6.1%	1.22 [1.04, 1.42]
**Subtotal (95% CI)**		**270**		**271**	**6.1%**	**1.22 [1.04, 1.42]**
Total events	161		133			
Heterogeneity: Not applicable						
Test for overall effect: Z = 2.45 (P = 0.01)						
**12.9.10 General disorders ('unsolicited'): Cervarix vs. Al(OH)3 (females, age 9-17; follow-up until 12 months)**						
HPV-058: Cervarix vs. Al(OH)3 (f, 9-17; 12)	1	374	4	376	0.0%	0.25 [0.03, 2.24]
**Subtotal (95% CI)**		**0**		**0**		**Not estimable**
Total events	0		0			
Heterogeneity: Not applicable						
Test for overall effect: Not applicable						
**12.9.11 General disorders ('unsolicited'): Cervarix vs. Al(OH)3 (females, age 15-25; follow-up until 7 months)**						
HPV-038: Cervarix vs. Al(OH)3 (f, 15-25; 7)	16	149	3	76	0.0%	2.72 [0.82, 9.05]
**Subtotal (95% CI)**		**0**		**0**		**Not estimable**
Total events	0		0			
Heterogeneity: Not applicable						
Test for overall effect: Not applicable						
**12.9.12 General disorders ('unsolicited'): Cervarix vs. Al(OH)3 (females, age 18-35; follow-up until 12 months)**						
HPV-003: Cervarix vs. Al(OH)3 (f, 18-30; 12)	1	31	2	30	0.0%	0.48 [0.05, 5.06]
HPV-031: Cervarix vs. Al(OH)3 (f, 18-35; 7)	2	176	2	178	0.0%	1.01 [0.14, 7.10]
HPV-035: Cervarix vs. Al(OH)3 (f, 18-35; 7)	23	150	14	150	0.0%	1.64 [0.88, 3.07]
**Subtotal (95% CI)**		**0**		**0**		**Not estimable**
Total events	0		0			
Heterogeneity: Not applicable						
Test for overall effect: Not applicable						
**12.9.13 General disorders ('unsolicited'): Cervarix vs. Engerix: hepatitis B vaccine (females and males, age 9-16; follow-up until 72 months)**						
HPV-030: Cervarix vs. Engerix (f, 9-15; 12)	32	247	29	246	0.0%	1.10 [0.69, 1.76]
HPV-040: Cervarix vs. Engerix (f & m, 12-16; 72)	27	1908	35	3128	0.0%	1.26 [0.77, 2.08]
**Subtotal (95% CI)**		**0**		**0**		**Not estimable**
Total events	0		0			
Heterogeneity: Not applicable						
Test for overall effect: Not applicable						
**12.9.14 General disorders ('unsolicited'): Cervarix vs. Havrix: hepatitis A vaccine (females, age 9-15; follow-up until 7 months)**						
HPV-033: Cervarix vs. Havrix (f, 9-15; 7)	8	160	1	161	0.0%	8.05 [1.02, 63.62]
**Subtotal (95% CI)**		**0**		**0**		**Not estimable**
Total events	0		0			
Heterogeneity: Not applicable						
Test for overall effect: Not applicable						
**12.9.15 General disorders ('unsolicited'): Cervarix vs. Twinrix: hepatitis A and B vaccine (females, age 8-15; follow-up until 12 months)**						
HPV-029: Cervarix vs. Twinrix (f, 8-15; 12)	10	270	8	271	0.0%	1.25 [0.50, 3.13]
**Subtotal (95% CI)**		**0**		**0**		**Not estimable**
Total events	0		0			
Heterogeneity: Not applicable						
Test for overall effect: Not applicable						
**12.9.16 General disorders ('systemic adverse events'): Gardasil vs. carrier solution (females and males, age 9-16; follow-up until 18 months)**						
V501-018: Gardasil vs. carrier solu. (f&m,9-16;18)	118	1184	52	597	2.8%	1.14 [0.84, 1.56]
**Subtotal (95% CI)**		**1184**		**597**	**2.8%**	**1.14 [0.84, 1.56]**
Total events	118		52			
Heterogeneity: Not applicable						
Test for overall effect: Z = 0.85 (P = 0.40)						
**12.9.17 General disorders ('systemic adverse events'): Gardasil vs. AAHS (males, age 15-27; follow-up until 36 months)**						
V501-020: Gardasil vs. AAHS (m, 15-27; 36)	133	2032	144	2033	4.2%	0.92 [0.74, 1.16]
**Subtotal (95% CI)**		**2032**		**2033**	**4.2%**	**0.92 [0.74, 1.16]**
Total events	133		144			
Heterogeneity: Not applicable						
Test for overall effect: Z = 0.68 (P = 0.50)						
**12.9.18 General disorders ('systemic adverse events'): Gardasil 9 vs. saline placebo (females, age 12-26; follow-up until 7 months)**						
V503-006: Gardasil 9 vs. saline (f, 12-26;7)	62	618	21	306	1.4%	1.46 [0.91, 2.35]
**Subtotal (95% CI)**		**618**		**306**	**1.4%**	**1.46 [0.91, 2.35]**
Total events	62		21			
Heterogeneity: Not applicable						
Test for overall effect: Z = 1.57 (P = 0.12)						
**12.9.19 General disorders ('systemic adverse events'): HPV 16 vaccine vs. AAHS (females, age 16-25; follow-up until 48 months)**						
V501-005: HPV 16 vaccine vs. AAHS (f, 16-25; 48)	102	1204	118	1205	3.7%	0.87 [0.67, 1.11]
**Subtotal (95% CI)**		**1204**		**1205**	**3.7%**	**0.87 [0.67, 1.11]**
Total events	102		118			
Heterogeneity: Not applicable						
Test for overall effect: Z = 1.12 (P = 0.26)						
**Total (95% CI)**		**21570**		**21014**	**100.0%**	**1.10 [1.04, 1.17]**
Total events	6401		5842			
Heterogeneity: $Tau^2 = 0.01$; $Chi^2 = 62.30$, df = 16 (P < 0.00001); $I^2 = 74\%$						
Test for overall effect: Z = 3.08 (P = 0.002)						
Test for subgroup differences: $Chi^2 = 43.57$, df = 12 (P < 0.0001), $I^2 = 72.5\%$						

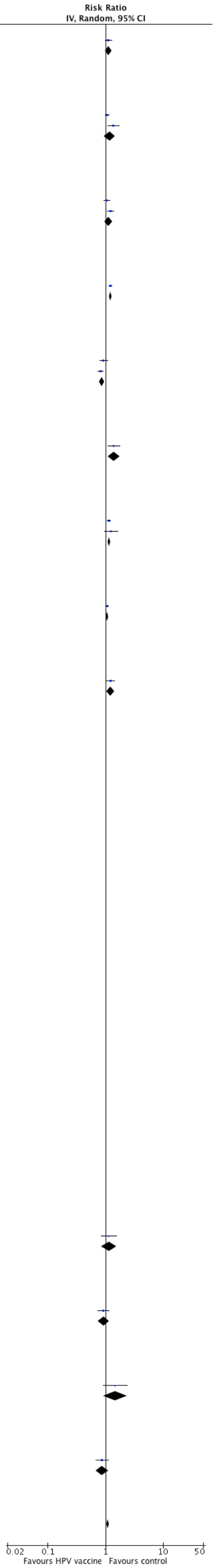

*12.9. Risk ratio for ‘solicited’ (GlaxoSmithKline): **1.11 [1.05, 1.19]**; risk ratio for ‘unsolicited’ (GlaxoSmithKline): 1.31 [0.99, 1.74]; risk ratio for ‘systemic adverse events’ (Merck Sharp & Dohme): 1.01 [0.84, 1.22]. To avoid double counting of participants in the total risk ratio estimate, we excluded the ‘unsolicited’ adverse events from total risk ratio estimate for studies that reported ‘solicited’ adverse events.

12.10. General harms ('solicited,' 'unsolicited' and 'systemic adverse events'*) reported within the MedDRA system organ class 'hepatobiliary disorders (10019805)': intention to treat analysis

Study or Subgroup	HPV vaccine Events	HPV vaccine Total	Control Events	Control Total	Weight	Risk Ratio IV, Random, 95% CI
**12.10.1 General hepatobiliary disorders ('unsolicited'): Cervarix vs. Engerix: hepatitis B vaccine (females, age 26-46; follow-up until 12 months)**						
HPV-069: Cervarix vs. Engerix (f, 26-46; 12)	2	606	1	606	100.0%	2.00 [0.18, 22.00]
**Subtotal (95% CI)**		**606**		**606**	**100.0%**	**2.00 [0.18, 22.00]**
Total events	2		1			
Heterogeneity: Not applicable						
Test for overall effect: Z = 0.57 (P = 0.57)						
**Total (95% CI)**		**606**		**606**	**100.0%**	**2.00 [0.18, 22.00]**
Total events	2		1			
Heterogeneity: Not applicable						
Test for overall effect: Z = 0.57 (P = 0.57)						
Test for subgroup differences: Not applicable						

Risk Ratio
IV, Random, 95% CI

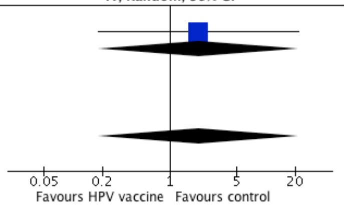

*12.10. Risk ratio for 'solicited' (GlaxoSmithKline): not applicable; risk ratio for 'unsolicited' (GlaxoSmithKline): 2.00 [0.18, 22.00]; risk ratio for 'systemic adverse events' (Merck Sharp & Dohme): not applicable.

12.11. General harms (‘solicited,’ ‘unsolicited’ and ‘systemic adverse events’*) reported within the MedDRA system organ class ‘immune system disorders (10021428)': intention to treat analysis

Study or Subgroup	HPV vaccine Events	HPV vaccine Total	Control Events	Control Total	Weight	Risk Ratio IV, Random, 95% CI
**12.11.1 General immune system disorders ('unsolicited'): Cervarix vs. Al(OH)3 (females, age 18-35; follow-up until 7 months)**						
HPV-031: Cervarix vs. Al(OH)3 (f, 18-35; 7)	0	176	1	178	2.7%	0.34 [0.01, 8.22]
HPV-035: Cervarix vs. Al(OH)3 (f, 18-35; 7)	3	150	1	150	5.5%	3.00 [0.32, 28.52]
**Subtotal (95% CI)**		**326**		**328**	**8.3%**	**1.36 [0.17, 10.68]**
Total events	3		2			
Heterogeneity: Tau² = 0.40; Chi² = 1.20, df = 1 (P = 0.27); I² = 17%						
Test for overall effect: Z = 0.30 (P = 0.77)						
**12.11.2 General immune system disorders ('unsolicited'): Cervarix vs. Engerix: hepatitis B vaccine (females and males, age 9-16; follow-up until 72 months)**						
HPV-030: Cervarix vs. Engerix (f, 9-15; 12)	1	247	1	246	3.7%	1.00 [0.06, 15.83]
HPV-040: Cervarix vs. Engerix (f & m, 12-16; 72)	3	1908	2	3128	8.8%	2.46 [0.41, 14.70]
**Subtotal (95% CI)**		**2155**		**3374**	**12.4%**	**1.88 [0.42, 8.46]**
Total events	4		3			
Heterogeneity: Tau² = 0.00; Chi² = 0.29, df = 1 (P = 0.59); I² = 0%						
Test for overall effect: Z = 0.83 (P = 0.41)						
**12.11.3 General immune system disorders ('unsolicited'): Cervarix vs. Twinrix: hepatitis A and B vaccine (females, age 8-15; follow-up until 12 months)**						
HPV-029: Cervarix vs. Twinrix (f, 8-15; 12)	3	270	1	271	5.5%	3.01 [0.32, 28.77]
**Subtotal (95% CI)**		**270**		**271**	**5.5%**	**3.01 [0.32, 28.77]**
Total events	3		1			
Heterogeneity: Not applicable						
Test for overall effect: Z = 0.96 (P = 0.34)						
**12.11.4 General immune system disorders ('systemic adverse events'): Gardasil vs. carrier solution (females and males, age 9-16; follow-up until 18 months)**						
V501-018: Gardasil vs. carrier solu. (f&m,9-16;18)	4	1184	2	597	9.8%	1.01 [0.19, 5.49]
**Subtotal (95% CI)**		**1184**		**597**	**9.8%**	**1.01 [0.19, 5.49]**
Total events	4		2			
Heterogeneity: Not applicable						
Test for overall effect: Z = 0.01 (P = 0.99)						
**12.11.5 General immune system disorders ('systemic adverse events'): Gardasil vs. AAHS (males, age 15-27; follow-up until 36 months)**						
V501-020: Gardasil vs. AAHS (m, 15-27; 36)	4	2032	3	2033	12.5%	1.33 [0.30, 5.95]
**Subtotal (95% CI)**		**2032**		**2033**	**12.5%**	**1.33 [0.30, 5.95]**
Total events	4		3			
Heterogeneity: Not applicable						
Test for overall effect: Z = 0.38 (P = 0.71)						
**12.11.6 General immune system disorders ('systemic adverse events'): Gardasil 9 vs. saline placebo (females, age 12-26; follow-up until 7 months)**						
V503-006: Gardasil 9 vs. saline (f, 12-26;7)	6	618	2	306	11.0%	1.49 [0.30, 7.32]
**Subtotal (95% CI)**		**618**		**306**	**11.0%**	**1.49 [0.30, 7.32]**
Total events	6		2			
Heterogeneity: Not applicable						
Test for overall effect: Z = 0.49 (P = 0.63)						
**12.11.7 General immune system disorders ('systemic adverse events'): HPV 16 vaccine vs. AAHS (females, age 16-25; follow-up until 48 months)**						
V501-005: HPV 16 vaccine vs. AAHS (f, 16-25; 48)	11	1204	11	1205	40.5%	1.00 [0.44, 2.30]
**Subtotal (95% CI)**		**1204**		**1205**	**40.5%**	**1.00 [0.44, 2.30]**
Total events	11		11			
Heterogeneity: Not applicable						
Test for overall effect: Z = 0.00 (P = 1.00)						
**Total (95% CI)**		**7789**		**8114**	**100.0%**	**1.29 [0.76, 2.18]**
Total events	35		24			
Heterogeneity: Tau² = 0.00; Chi² = 2.76, df = 8 (P = 0.95); I² = 0%						
Test for overall effect: Z = 0.93 (P = 0.35)						
Test for subgroup differences: Chi² = 1.26, df = 6 (P = 0.97), I² = 0%						

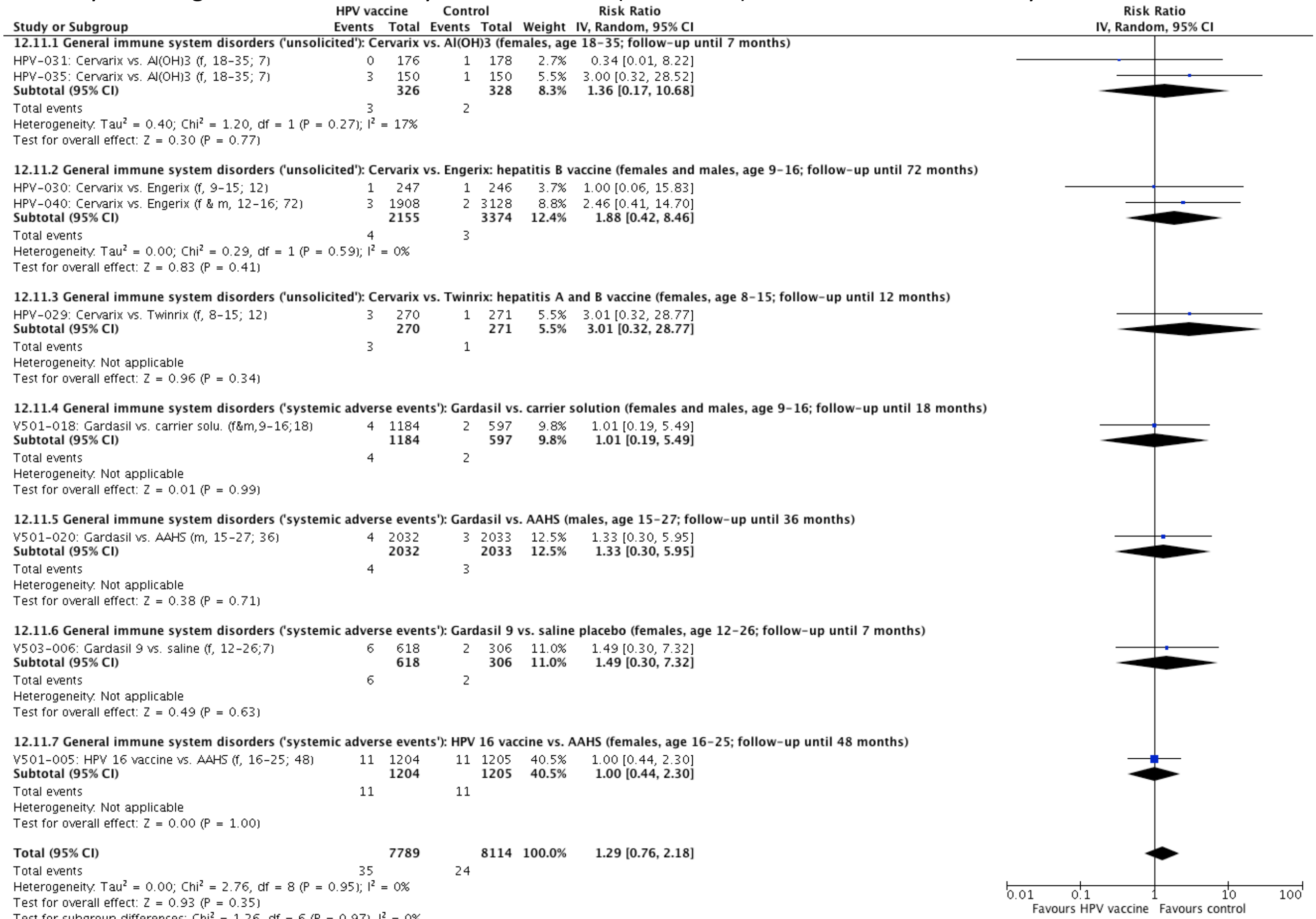

*12.11. Risk ratio for ‘solicited’ (GlaxoSmithKline): not applicable; risk ratio for ‘unsolicited’ (GlaxoSmithKline): 1.91 [0.68, 5.39]; risk ratio for ‘systemic adverse events’ (Merck Sharp & Dohme): 1.12 [0.60, 2.07].

12.12. General harms ('solicited,' 'unsolicited' and 'systemic adverse events'*) reported within the MedDRA system organ class 'infections and infestations (10021881)': intention to treat analysis

Study or Subgroup	HPV vaccine Events	HPV vaccine Total	Control Events	Control Total	Weight	Risk Ratio IV, Random, 95% CI
**12.12.1 General infections and infestations ('unsolicited'): Cervarix vs. Al(OH)3 (females, age 9-17; follow-up until 12 months)**						
HPV-058: Cervarix vs. Al(OH)3 (f, 9-17; 12)	149	374	119	376	11.6%	1.26 [1.04, 1.53]
**Subtotal (95% CI)**		**374**		**376**	**11.6%**	**1.26 [1.04, 1.53]**
Total events	149		119			
Heterogeneity: Not applicable						
Test for overall effect: Z = 2.33 (P = 0.02)						
**12.12.2 General infections and infestations ('unsolicited'): Cervarix vs. Al(OH)3 (females, age 15-25; follow-up until 7 months)**						
HPV-038: Cervarix vs. Al(OH)3 (f, 15-25; 7)	33	149	7	76	2.5%	2.40 [1.12, 5.18]
**Subtotal (95% CI)**		**149**		**76**	**2.5%**	**2.40 [1.12, 5.18]**
Total events	33		7			
Heterogeneity: Not applicable						
Test for overall effect: Z = 2.24 (P = 0.03)						
**12.12.3 General infections and infestations ('unsolicited'): Cervarix vs. Al(OH)3 (females, age 18-35; follow-up until 12 months)**						
HPV-003: Cervarix vs. Al(OH)3 (f, 18-30; 12)	15	31	9	30	3.2%	1.61 [0.84, 3.11]
HPV-031: Cervarix vs. Al(OH)3 (f, 18-35; 7)	6	176	3	178	0.9%	2.02 [0.51, 7.96]
HPV-035: Cervarix vs. Al(OH)3 (f, 18-35; 7)	47	150	41	150	7.3%	1.15 [0.81, 1.63]
**Subtotal (95% CI)**		**357**		**358**	**11.3%**	**1.27 [0.94, 1.72]**
Total events	68		53			
Heterogeneity: Tau^2 = 0.00; Chi^2 = 1.28, df = 2 (P = 0.53); I^2 = 0%						
Test for overall effect: Z = 1.53 (P = 0.13)						
**12.12.4 General infections and infestations ('unsolicited'): Cervarix vs. Engerix: hepatitis B vaccine (females and males, age 9-16; follow-up until 72 months)**						
HPV-030: Cervarix vs. Engerix (f, 9-15; 12)	49	247	46	246	7.1%	1.06 [0.74, 1.52]
HPV-040: Cervarix vs. Engerix (f & m, 12-16; 72)	96	1908	96	3128	9.2%	1.64 [1.24, 2.16]
**Subtotal (95% CI)**		**2155**		**3374**	**16.3%**	**1.34 [0.88, 2.05]**
Total events	145		142			
Heterogeneity: Tau^2 = 0.07; Chi^2 = 3.51, df = 1 (P = 0.06); I^2 = 71%						
Test for overall effect: Z = 1.35 (P = 0.18)						
**12.12.5 General infections and infestations ('unsolicited'): Cervarix vs. Engerix: hepatitis B vaccine (females, age 26-46; follow-up until 12 months)**						
HPV-069: Cervarix vs. Engerix (f, 26-46; 12)	27	606	31	606	4.7%	0.87 [0.53, 1.44]
**Subtotal (95% CI)**		**606**		**606**	**4.7%**	**0.87 [0.53, 1.44]**
Total events	27		31			
Heterogeneity: Not applicable						
Test for overall effect: Z = 0.54 (P = 0.59)						
**12.12.6 General infections and infestations ('unsolicited'): Cervarix vs. Havrix: hepatitis A vaccine (females, age 9-15; follow-up until 7 months)**						
HPV-033: Cervarix vs. Havrix (f, 9-15; 7)	30	160	25	161	5.0%	1.21 [0.74, 1.96]
**Subtotal (95% CI)**		**160**		**161**	**5.0%**	**1.21 [0.74, 1.96]**
Total events	30		25			
Heterogeneity: Not applicable						
Test for overall effect: Z = 0.76 (P = 0.44)						
**12.12.7 General infections and infestations ('unsolicited'): Cervarix vs. Twinrix: hepatitis A and B vaccine (females, age 8-15; follow-up until 12 months)**						
HPV-029: Cervarix vs. Twinrix (f, 8-15; 12)	56	270	44	271	7.2%	1.28 [0.89, 1.83]
**Subtotal (95% CI)**		**270**		**271**	**7.2%**	**1.28 [0.89, 1.83]**
Total events	56		44			
Heterogeneity: Not applicable						
Test for overall effect: Z = 1.34 (P = 0.18)						
**12.12.8 General infections and infestations ('systemic adverse events'): Gardasil vs. carrier solution (females and males, age 9-16; follow-up until 18 months)**						
V501-018: Gardasil vs. carrier solu. (f&m,9-16;18)	134	1184	85	597	9.8%	0.79 [0.62, 1.02]
**Subtotal (95% CI)**		**1184**		**597**	**9.8%**	**0.79 [0.62, 1.02]**
Total events	134		85			
Heterogeneity: Not applicable						
Test for overall effect: Z = 1.78 (P = 0.08)						
**12.12.9 General infections and infestations ('systemic adverse events'): Gardasil vs. AAHS (males, age 15-27; follow-up until 36 months)**						
V501-020: Gardasil vs. AAHS (m, 15-27; 36)	182	2032	187	2033	11.6%	0.97 [0.80, 1.18]
**Subtotal (95% CI)**		**2032**		**2033**	**11.6%**	**0.97 [0.80, 1.18]**
Total events	182		187			
Heterogeneity: Not applicable						
Test for overall effect: Z = 0.27 (P = 0.79)						
**12.12.10 General infections and infestations ('systemic adverse events'): Gardasil 9 vs. saline placebo (females, age 12-26; follow-up until 7 months)**						
V503-006: Gardasil 9 vs. saline (f, 12-26;7)	95	618	39	306	7.4%	1.21 [0.85, 1.71]
**Subtotal (95% CI)**		**618**		**306**	**7.4%**	**1.21 [0.85, 1.71]**
Total events	95		39			
Heterogeneity: Not applicable						
Test for overall effect: Z = 1.06 (P = 0.29)						
**12.12.11 General infections and infestations ('systemic adverse events'): HPV 16 vaccine vs. AAHS (females, age 16-25; follow-up until 48 months)**						
V501-005: HPV 16 vaccine vs. AAHS (f, 16-25; 48)	222	1204	233	1205	12.5%	0.95 [0.81, 1.13]
**Subtotal (95% CI)**		**1204**		**1205**	**12.5%**	**0.95 [0.81, 1.13]**
Total events	222		233			
Heterogeneity: Not applicable						
Test for overall effect: Z = 0.56 (P = 0.57)						
**Total (95% CI)**		**9109**		**9363**	**100.0%**	**1.14 [1.00, 1.30]**
Total events	1141		965			
Heterogeneity: Tau^2 = 0.03; Chi^2 = 28.56, df = 13 (P = 0.008); I^2 = 54%						
Test for overall effect: Z = 1.98 (P = 0.05)						
Test for subgroup differences: Chi^2 = 19.58, df = 10 (P = 0.03), I^2 = 48.9%						

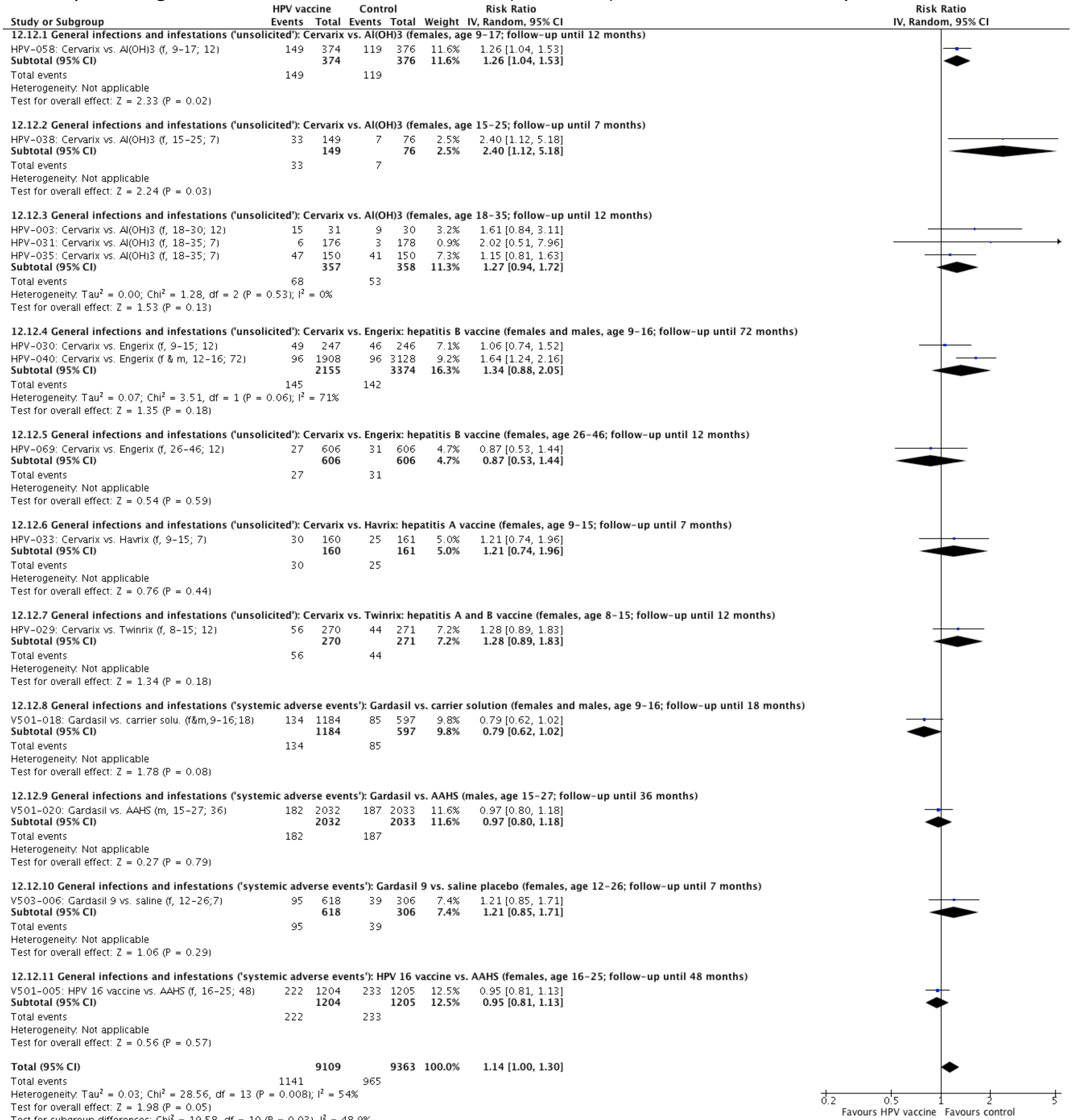

*12.12. Risk ratio for 'solicited' (GlaxoSmithKline): not applicable; risk ratio for 'unsolicited' (GlaxoSmithKline): **1.29 [1.13, 1.47]**; risk ratio for 'systemic adverse events' (Merck Sharp & Dohme): 0.95 [0.84, 1.08].

12.13. General harms (‘solicited,’ ‘unsolicited’ and ‘systemic adverse events’*) reported within the MedDRA system organ class ‘injury poisoning and procedural complications (10022117)': intention to treat analysis

Study or Subgroup	HPV vaccine Events	HPV vaccine Total	Control Events	Control Total	Weight	Risk Ratio IV, Random, 95% CI
**12.13.1 General injury, poisoning and procedural complications ('unsolicited'): Cervarix vs. Al(OH)3 (females, age 9-17; follow-up until 12 months)**						
HPV-058: Cervarix vs. Al(OH)3 (f, 9-17; 12)	3	374	3	376	2.4%	1.01 [0.20, 4.95]
**Subtotal (95% CI)**		**374**		**376**	**2.4%**	**1.01 [0.20, 4.95]**
Total events	3		3			
Heterogeneity: Not applicable						
Test for overall effect: Z = 0.01 (P = 0.99)						
**12.13.2 General injury, poisoning and procedural complications ('unsolicited'): Cervarix vs. Al(OH)3 (females, age 15-25; follow-up until 7 months)**						
HPV-038: Cervarix vs. Al(OH)3 (f, 15-25; 7)	3	149	0	76	0.7%	3.59 [0.19, 68.68]
**Subtotal (95% CI)**		**149**		**76**	**0.7%**	**3.59 [0.19, 68.68]**
Total events	3		0			
Heterogeneity: Not applicable						
Test for overall effect: Z = 0.85 (P = 0.40)						
**12.13.3 General injury, poisoning and procedural complications ('unsolicited'): Cervarix vs. Al(OH)3 (females, age 18-35; follow-up until 12 months)**						
HPV-003: Cervarix vs. Al(OH)3 (f, 18-30; 12)	1	31	0	30	0.6%	2.91 [0.12, 68.66]
HPV-031: Cervarix vs. Al(OH)3 (f, 18-35; 7)	1	176	3	178	1.2%	0.34 [0.04, 3.21]
HPV-035: Cervarix vs. Al(OH)3 (f, 18-35; 7)	2	150	3	150	2.0%	0.67 [0.11, 3.93]
**Subtotal (95% CI)**		**357**		**358**	**3.8%**	**0.68 [0.19, 2.44]**
Total events	4		6			
Heterogeneity: $Tau^2 = 0.00$; $Chi^2 = 1.18$, df = 2 (P = 0.55); $I^2 = 0\%$						
Test for overall effect: Z = 0.59 (P = 0.55)						
**12.13.4 General injury, poisoning and procedural complications ('unsolicited'): Cervarix vs. Engerix: hepatitis B vaccine (females and males, age 9-16; follow-up until 72 months)**						
HPV-030: Cervarix vs. Engerix (f, 9-15; 12)	9	247	4	246	4.6%	2.24 [0.70, 7.18]
HPV-040: Cervarix vs. Engerix (f & m, 12-16; 72)	11	1908	22	3128	11.9%	0.82 [0.40, 1.69]
**Subtotal (95% CI)**		**2155**		**3374**	**16.5%**	**1.22 [0.46, 3.18]**
Total events	20		26			
Heterogeneity: $Tau^2 = 0.26$; $Chi^2 = 2.07$, df = 1 (P = 0.15); $I^2 = 52\%$						
Test for overall effect: Z = 0.40 (P = 0.69)						
**12.13.5 General injury, poisoning and procedural complications ('unsolicited'): Cervarix vs. Engerix: hepatitis B vaccine (females, age 26-46; follow-up until 12 months)**						
HPV-069: Cervarix vs. Engerix (f, 26-46; 12)	1	606	0	606	0.6%	3.00 [0.12, 73.50]
**Subtotal (95% CI)**		**606**		**606**	**0.6%**	**3.00 [0.12, 73.50]**
Total events	1		0			
Heterogeneity: Not applicable						
Test for overall effect: Z = 0.67 (P = 0.50)						
**12.13.6 General injury, poisoning and procedural complications ('unsolicited'): Cervarix vs. Havrix: hepatitis A vaccine (females, age 9-15; follow-up until 7 months)**						
HPV-033: Cervarix vs. Havrix (f, 9-15; 7)	1	160	2	161	1.1%	0.50 [0.05, 5.49]
**Subtotal (95% CI)**		**160**		**161**	**1.1%**	**0.50 [0.05, 5.49]**
Total events	1		2			
Heterogeneity: Not applicable						
Test for overall effect: Z = 0.56 (P = 0.57)						
**12.13.7 General injury, poisoning and procedural complications ('unsolicited'): Cervarix vs. Twinrix: hepatitis A and B vaccine (females, age 8-15; follow-up until 12 months)**						
HPV-029: Cervarix vs. Twinrix (f, 8-15; 12)	9	270	9	271	7.5%	1.00 [0.40, 2.49]
**Subtotal (95% CI)**		**270**		**271**	**7.5%**	**1.00 [0.40, 2.49]**
Total events	9		9			
Heterogeneity: Not applicable						
Test for overall effect: Z = 0.01 (P = 0.99)						
**12.13.8 General injury, poisoning and procedural complications ('systemic adverse events'): Gardasil vs. carrier solution (females and males, age 9-16; follow-up until 18 months)**						
V501-018: Gardasil vs. carrier solu. (f&m,9-16;18)	33	1184	15	597	17.1%	1.11 [0.61, 2.03]
**Subtotal (95% CI)**		**1184**		**597**	**17.1%**	**1.11 [0.61, 2.03]**
Total events	33		15			
Heterogeneity: Not applicable						
Test for overall effect: Z = 0.34 (P = 0.74)						
**12.13.9 General injury, poisoning and procedural complications ('systemic adverse events'): Gardasil vs. AAHS (males, age 15-27; follow-up until 36 months)**						
V501-020: Gardasil vs. AAHS (m, 15-27; 36)	30	2032	24	2033	21.8%	1.25 [0.73, 2.13]
**Subtotal (95% CI)**		**2032**		**2033**	**21.8%**	**1.25 [0.73, 2.13]**
Total events	30		24			
Heterogeneity: Not applicable						
Test for overall effect: Z = 0.82 (P = 0.41)						
**12.13.10 General injury, poisoning and procedural complications ('systemic adverse events'): Gardasil 9 vs. saline placebo (females, age 12-26; follow-up until 7 months)**						
V503-006: Gardasil 9 vs. saline (f, 12-26;7)	13	618	4	306	5.0%	1.61 [0.53, 4.89]
**Subtotal (95% CI)**		**618**		**306**	**5.0%**	**1.61 [0.53, 4.89]**
Total events	13		4			
Heterogeneity: Not applicable						
Test for overall effect: Z = 0.84 (P = 0.40)						
**12.13.11 General injury, poisoning and procedural complications ('systemic adverse events'): HPV 16 vaccine vs. AAHS (females, age 16-25; follow-up until 48 months)**						
V501-005: HPV 16 vaccine vs. AAHS (f, 16-25; 48)	25	1204	33	1205	23.5%	0.76 [0.45, 1.27]
**Subtotal (95% CI)**		**1204**		**1205**	**23.5%**	**0.76 [0.45, 1.27]**
Total events	25		33			
Heterogeneity: Not applicable						
Test for overall effect: Z = 1.06 (P = 0.29)						
**Total (95% CI)**		**9109**		**9363**	**100.0%**	**1.03 [0.81, 1.33]**
Total events	142		122			
Heterogeneity: $Tau^2 = 0.00$; $Chi^2 = 7.70$, df = 13 (P = 0.86); $I^2 = 0\%$						
Test for overall effect: Z = 0.26 (P = 0.80)						
Test for subgroup differences: $Chi^2 = 4.54$, df = 10 (P = 0.92), $I^2 = 0\%$						

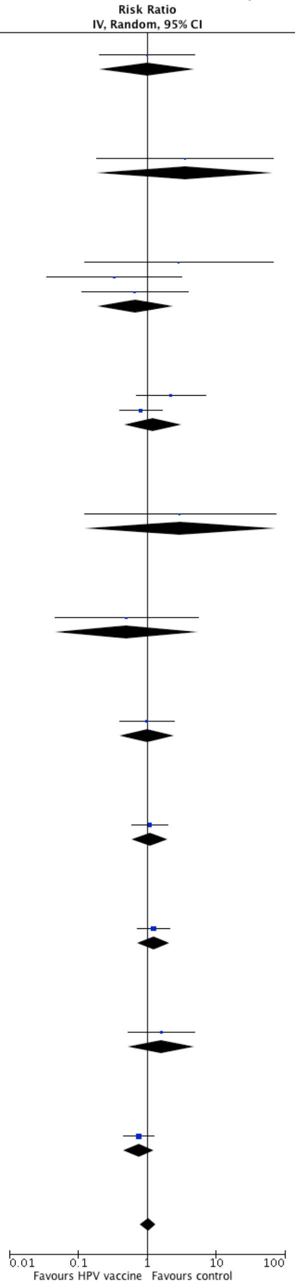

*12.13. Risk ratio for ‘solicited’ (GlaxoSmithKline): not applicable; risk ratio for ‘unsolicited’ (GlaxoSmithKline): 1.02 [0.66, 1.58]; risk ratio for ‘systemic adverse events’ (Merck Sharp & Dohme): 1.04 [0.77, 1.41].

12.14. General harms (‘solicited,’ ‘unsolicited’ and ‘systemic adverse events’*) reported within the MedDRA system organ class ‘investigations (10022891)': intention to treat analysis

Study or Subgroup	HPV vaccine Events	HPV vaccine Total	Control Events	Control Total	Weight	Risk Ratio IV, Random, 95% CI
**12.14.1 General investigations ('unsolicited'): Cervarix vs. Al(OH)3 (females, age 18-35; follow-up until 7 months)**						
HPV-031: Cervarix vs. Al(OH)3 (f, 18-35; 7)	0	176	1	178	13.9%	0.34 [0.01, 8.22]
HPV-035: Cervarix vs. Al(OH)3 (f, 18-35; 7)	1	150	0	150	13.9%	3.00 [0.12, 73.06]
**Subtotal (95% CI)**		**326**		**328**	**27.8%**	**1.01 [0.11, 9.62]**
Total events	1		1			
Heterogeneity: $Tau^2 = 0.00$; $Chi^2 = 0.90$, df = 1 (P = 0.34); $I^2 = 0\%$						
Test for overall effect: Z = 0.01 (P = 1.00)						
**12.14.2 General investigations ('systemic adverse events'): Gardasil vs. AAHS (males, age 15-27; follow-up until 36 months)**						
V501-020: Gardasil vs. AAHS (m, 15-27; 36)	3	2032	2	2033	44.4%	1.50 [0.25, 8.97]
**Subtotal (95% CI)**		**2032**		**2033**	**44.4%**	**1.50 [0.25, 8.97]**
Total events	3		2			
Heterogeneity: Not applicable						
Test for overall effect: Z = 0.44 (P = 0.66)						
**12.14.3 General investigations ('systemic adverse events'): Gardasil 9 vs. saline placebo (females, age 12-26; follow-up until 7 months)**						
V503-006: Gardasil 9 vs. saline (f, 12-26;7)	3	618	1	306	27.8%	1.49 [0.16, 14.22]
**Subtotal (95% CI)**		**618**		**306**	**27.8%**	**1.49 [0.16, 14.22]**
Total events	3		1			
Heterogeneity: Not applicable						
Test for overall effect: Z = 0.34 (P = 0.73)						
**Total (95% CI)**		**2976**		**2667**	**100.0%**	**1.34 [0.41, 4.41]**
Total events	7		4			
Heterogeneity: $Tau^2 = 0.00$; $Chi^2 = 0.99$, df = 3 (P = 0.80); $I^2 = 0\%$						
Test for overall effect: Z = 0.48 (P = 0.63)						
Test for subgroup differences: $Chi^2 = 0.09$, df = 2 (P = 0.96), $I^2 = 0\%$						

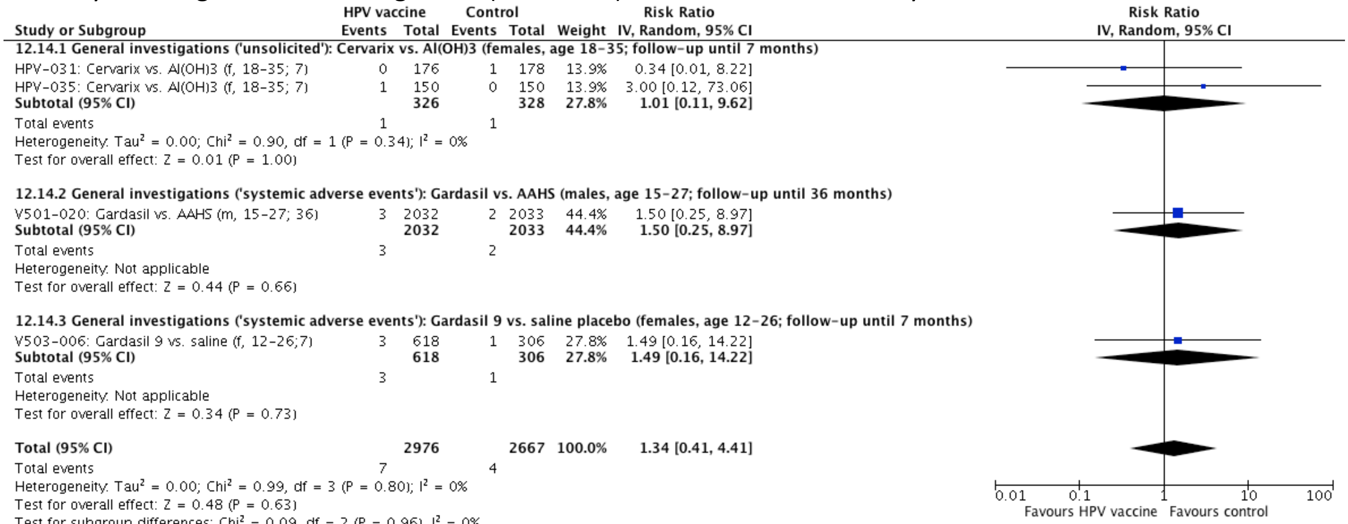

*12.14. Risk ratio for ‘solicited’ (GlaxoSmithKline): not applicable; risk ratio for ‘unsolicited’ (GlaxoSmithKline): 1.01 [0.11, 9.62]; risk ratio for ‘systemic adverse events’ (Merck Sharp & Dohme): 1.49 [0.37, 6.07].

12.15. General harms (‘solicited,’ ‘unsolicited’ and ‘systemic adverse events’*) reported within the MedDRA system organ class ‘metabolism and nutrition disorders (10027433)': intention to treat analysis

Study or Subgroup	HPV vaccine Events	HPV vaccine Total	Control Events	Control Total	Weight	Risk Ratio IV, Random, 95% CI
**12.15.1 General metabolism and nutrition disorders ('unsolicited'): Cervarix vs. Al(OH)3 (females, age 9-17; follow-up until 12 months)**						
HPV-058: Cervarix vs. Al(OH)3 (f, 9-17; 12)	1	374	0	376	4.7%	3.02 [0.12, 73.80]
**Subtotal (95% CI)**		**374**		**376**	**4.7%**	**3.02 [0.12, 73.80]**
Total events	1		0			
Heterogeneity: Not applicable						
Test for overall effect: Z = 0.68 (P = 0.50)						
**12.15.2 General metabolism and nutrition disorders ('unsolicited'): Cervarix vs. Al(OH)3 (females, age 18-35; follow-up until 7 months)**						
HPV-031: Cervarix vs. Al(OH)3 (f, 18-35; 7)	0	176	1	178	4.7%	0.34 [0.01, 8.22]
**Subtotal (95% CI)**		**176**		**178**	**4.7%**	**0.34 [0.01, 8.22]**
Total events	0		1			
Heterogeneity: Not applicable						
Test for overall effect: Z = 0.67 (P = 0.50)						
**12.15.3 General metabolism and nutrition disorders ('systemic adverse events'): Gardasil vs. carrier solution (females and males, age 9-16; follow-up until 18 months)**						
V501-018: Gardasil vs. carrier solu. (f&m,9-16;18)	2	1184	3	597	15.1%	0.34 [0.06, 2.01]
**Subtotal (95% CI)**		**1184**		**597**	**15.1%**	**0.34 [0.06, 2.01]**
Total events	2		3			
Heterogeneity: Not applicable						
Test for overall effect: Z = 1.20 (P = 0.23)						
**12.15.4 General metabolism and nutrition disorders ('systemic adverse events'): Gardasil vs. AAHS (males, age 15-27; follow-up until 36 months)**						
V501-020: Gardasil vs. AAHS (m, 15-27; 36)	1	2032	2	2033	8.4%	0.50 [0.05, 5.51]
**Subtotal (95% CI)**		**2032**		**2033**	**8.4%**	**0.50 [0.05, 5.51]**
Total events	1		2			
Heterogeneity: Not applicable						
Test for overall effect: Z = 0.57 (P = 0.57)						
**12.15.5 General metabolism and nutrition disorders ('systemic adverse events'): Gardasil 9 vs. saline placebo (females, age 12-26; follow-up until 7 months)**						
V503-006: Gardasil 9 vs. saline (f, 12-26;7)	1	618	1	306	6.3%	0.50 [0.03, 7.89]
**Subtotal (95% CI)**		**618**		**306**	**6.3%**	**0.50 [0.03, 7.89]**
Total events	1		1			
Heterogeneity: Not applicable						
Test for overall effect: Z = 0.50 (P = 0.62)						
**12.15.6 General metabolism and nutrition disorders ('systemic adverse events'): HPV 16 vaccine vs. AAHS (females, age 16-25; follow-up until 48 months)**						
V501-005: HPV 16 vaccine vs. AAHS (f, 16-25; 48)	12	1204	8	1205	60.8%	1.50 [0.62, 3.66]
**Subtotal (95% CI)**		**1204**		**1205**	**60.8%**	**1.50 [0.62, 3.66]**
Total events	12		8			
Heterogeneity: Not applicable						
Test for overall effect: Z = 0.89 (P = 0.37)						
**Total (95% CI)**		**5588**		**4695**	**100.0%**	**0.98 [0.49, 1.96]**
Total events	17		15			
Heterogeneity: $Tau^2 = 0.00$; $Chi^2 = 3.70$, df = 5 (P = 0.59); $I^2 = 0\%$						
Test for overall effect: Z = 0.06 (P = 0.96)						
Test for subgroup differences: $Chi^2 = 3.70$, df = 5 (P = 0.59), $I^2 = 0\%$						

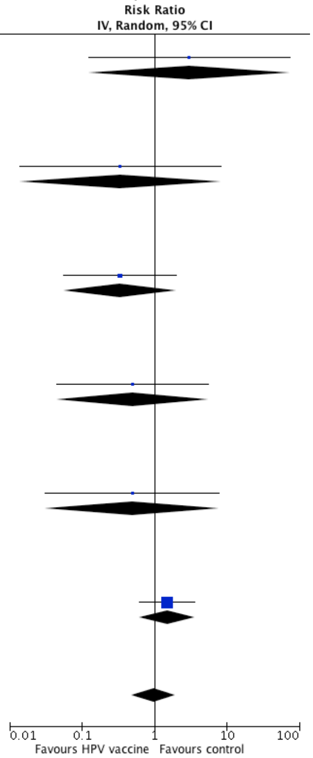

*12.15. Risk ratio for ‘solicited’ (GlaxoSmithKline): not applicable; risk ratio for ‘unsolicited’ (GlaxoSmithKline): 1.01 [0.11, 9.65]; risk ratio for ‘systemic adverse events’ (Merck Sharp & Dohme): 0.98 [0.47, 2.03].

12.16. General harms (‘solicited,’ ‘unsolicited’ and ‘systemic adverse events’*) reported within the MedDRA system organ class ‘musculoskeletal and connective tissue disorders (10028395)': intention to treat analysis

Study or Subgroup	HPV vaccine Events	HPV vaccine Total	Control Events	Control Total	Weight	Risk Ratio IV, Random, 95% CI
**12.16.1 General musculoskeletal and connective tissue disorders ('solicited'): Cervarix vs. Al(OH)3 (females, age 9-17; follow-up until 12 months)**						
HPV-058: Cervarix vs. Al(OH)3 (f, 9-17; 12)	148	374	126	376	7.1%	1.18 [0.98, 1.43]
**Subtotal (95% CI)**		**374**		**376**	**7.1%**	**1.18 [0.98, 1.43]**
Total events	148		126			
Heterogeneity: Not applicable						
Test for overall effect: Z = 1.72 (P = 0.09)						
**12.16.2 General musculoskeletal and connective tissue disorders ('solicited'): Cervarix vs. Al(OH)3 (females, age 15-25; follow-up until 7 months)**						
HPV-038: Cervarix vs. Al(OH)3 (f, 15-25; 7)	132	149	41	76	6.6%	1.64 [1.32, 2.04]
**Subtotal (95% CI)**		**149**		**76**	**6.6%**	**1.64 [1.32, 2.04]**
Total events	132		41			
Heterogeneity: Not applicable						
Test for overall effect: Z = 4.51 (P < 0.00001)						
**12.16.3 General musculoskeletal and connective tissue disorders ('solicited'): Cervarix vs. Al(OH)3 (females, age 18-35; follow-up until 12 months)**						
HPV-003: Cervarix vs. Al(OH)3 (f, 18-30; 12)	1	31	0	30	0.1%	2.91 [0.12, 68.66]
HPV-031: Cervarix vs. Al(OH)3 (f, 18-35; 7)	29	176	27	178	3.2%	1.09 [0.67, 1.76]
HPV-035: Cervarix vs. Al(OH)3 (f, 18-35; 7)	114	150	86	150	7.5%	1.33 [1.12, 1.56]
**Subtotal (95% CI)**		**357**		**358**	**10.8%**	**1.30 [1.11, 1.52]**
Total events	144		113			
Heterogeneity: $Tau^2 = 0.00$; $Chi^2 = 0.84$, df = 2 (P = 0.66); $I^2 = 0\%$						
Test for overall effect: Z = 3.31 (P = 0.0009)						
**12.16.4 General musculoskeletal and connective tissue disorders ('solicited'): Cervarix vs. Al(OH)3 (females, age 24-72; follow-up until 36 months)**						
HPV-015: Cervarix vs. Al(OH)3 (f, 24-72; 36)	1458	2882	1061	2871	8.9%	1.37 [1.29, 1.45]
**Subtotal (95% CI)**		**2882**		**2871**	**8.9%**	**1.37 [1.29, 1.45]**
Total events	1458		1061			
Heterogeneity: Not applicable						
Test for overall effect: Z = 10.28 (P < 0.00001)						
**12.16.5 General musculoskeletal and connective tissue disorders ('solicited'): Cervarix vs. Engerix: hepatitis B vaccine (females and males, age 9-15; follow-up until 12 months)**						
HPV-030: Cervarix vs. Engerix (f, 9-15; 12)	78	247	79	246	5.9%	0.98 [0.76, 1.27]
HPV-040: Cervarix vs. Engerix (f & m, 12-16; 72)	428	604	347	1028	8.5%	2.10 [1.90, 2.32]
**Subtotal (95% CI)**		**851**		**1274**	**14.4%**	**1.45 [0.69, 3.05]**
Total events	506		426			
Heterogeneity: $Tau^2 = 0.28$; $Chi^2 = 28.82$, df = 1 (P < 0.00001); $I^2 = 97\%$						
Test for overall effect: Z = 0.98 (P = 0.33)						
**12.16.6 General musculoskeletal and connective tissue disorders ('solicited'): Cervarix vs. Engerix: hepatitis B vaccine (females, age 26-46; follow-up until 12 months)**						
HPV-069: Cervarix vs. Engerix (f, 26-46; 12)	88	606	56	606	5.0%	1.57 [1.15, 2.15]
**Subtotal (95% CI)**		**606**		**606**	**5.0%**	**1.57 [1.15, 2.15]**
Total events	88		56			
Heterogeneity: Not applicable						
Test for overall effect: Z = 2.81 (P = 0.005)						
**12.16.7 General musculoskeletal and connective tissue disorders ('solicited'): Cervarix vs. Havrix: hepatitis A vaccine (females, age 9-15; follow-up until 12 months)**						
HPV-013: Cervarix vs. Havrix (f, 10-14; 12)	768	1035	544	1032	8.8%	1.41 [1.32, 1.51]
HPV-033: Cervarix vs. Havrix (f, 9-15; 7)	67	160	43	161	5.1%	1.57 [1.14, 2.15]
**Subtotal (95% CI)**		**1195**		**1193**	**13.9%**	**1.41 [1.32, 1.51]**
Total events	835		587			
Heterogeneity: $Tau^2 = 0.00$; $Chi^2 = 0.43$, df = 1 (P = 0.51); $I^2 = 0\%$						
Test for overall effect: Z = 10.22 (P < 0.00001)						
**12.16.8 General musculoskeletal and connective tissue disorders ('solicited'): Cervarix vs. Havrix: hepatitis A vaccine (females, age 15-25; follow-up until 48 months)**						
HPV-008: Cervarix vs. Havrix (f, 15-25; 48)	2240	9319	1932	9325	9.0%	1.16 [1.10, 1.22]
**Subtotal (95% CI)**		**9319**		**9325**	**9.0%**	**1.16 [1.10, 1.22]**
Total events	2240		1932			
Heterogeneity: Not applicable						
Test for overall effect: Z = 5.43 (P < 0.00001)						
**12.16.9 General musculoskeletal and connective tissue disorders ('solicited'): Cervarix vs. Twinrix: hepatitis A and B vaccine (females, age 8-15; follow-up until 12 months)**						
HPV-029: Cervarix vs. Twinrix (f, 8-15; 12)	144	270	115	271	7.3%	1.26 [1.05, 1.50]
**Subtotal (95% CI)**		**270**		**271**	**7.3%**	**1.26 [1.05, 1.50]**
Total events	144		115			
Heterogeneity: Not applicable						
Test for overall effect: Z = 2.52 (P = 0.01)						
**12.16.10 General musculoskeletal and connective tissue disorders ('unsolicited'): Cervarix vs. Al(OH)3 (females, age 9-17; follow-up until 12 months)**						
HPV-058: Cervarix vs. Al(OH)3 (f, 9-17; 12)	2	374	1	376	0.0%	2.01 [0.18, 22.08]
**Subtotal (95% CI)**		**0**		**0**		**Not estimable**
Total events	0		0			
Heterogeneity: Not applicable						
Test for overall effect: Not applicable						
**12.16.11 General musculoskeletal and connective tissue disorders ('unsolicited'): Cervarix vs. Al(OH)3 (females, age 15-25; follow-up until 7 months)**						
HPV-038: Cervarix vs. Al(OH)3 (f, 15-25; 7)	2	149	0	76	0.0%	2.57 [0.12, 52.80]
**Subtotal (95% CI)**		**0**		**0**		**Not estimable**
Total events	0		0			
Heterogeneity: Not applicable						
Test for overall effect: Not applicable						
**12.16.12 General musculoskeletal and connective tissue disorders ('unsolicited'): Cervarix vs. Al(OH)3 (females, age 18-35; follow-up until 12 months)**						
HPV-003: Cervarix vs. Al(OH)3 (f, 18-30; 12)	4	31	1	30	0.0%	3.87 [0.46, 32.67]
HPV-031: Cervarix vs. Al(OH)3 (f, 18-35; 7)	4	176	3	178	0.0%	1.35 [0.31, 5.94]
HPV-035: Cervarix vs. Al(OH)3 (f, 18-35; 7)	5	150	4	150	0.0%	1.25 [0.34, 4.56]
**Subtotal (95% CI)**		**0**		**0**		**Not estimable**
Total events	0		0			
Heterogeneity: Not applicable						
Test for overall effect: Not applicable						
**12.16.13 General musculoskeletal and connective tissue disorders ('unsolicited'): Cervarix vs. Engerix: hepatitis B vaccine (females and males, age 9-16; follow-up until 72 months)**						
HPV-030: Cervarix vs. Engerix (f, 9-15; 12)	31	247	35	246	0.0%	0.88 [0.56, 1.38]
HPV-040: Cervarix vs. Engerix (f & m, 12-16; 72)	16	1908	15	3128	0.0%	1.75 [0.87, 3.53]
**Subtotal (95% CI)**		**0**		**0**		**Not estimable**
Total events	0		0			
Heterogeneity: Not applicable						
Test for overall effect: Not applicable						
**12.16.14 General musculoskeletal and connective tissue disorders ('unsolicited'): Cervarix vs. Engerix: hepatitis B vaccine (females, age 26-46; follow-up until 12 months)**						
HPV-069: Cervarix vs. Engerix (f, 26-46; 12)	1	606	0	606	0.0%	3.00 [0.12, 73.50]
**Subtotal (95% CI)**		**0**		**0**		**Not estimable**
Total events	0		0			
Heterogeneity: Not applicable						
Test for overall effect: Not applicable						
**12.16.15 General musculoskeletal and connective tissue disorders ('unsolicited'): Cervarix vs. Havrix: hepatitis A vaccine (females, age 9-15; follow-up until 12 months)**						
HPV-033: Cervarix vs. Havrix (f, 9-15; 7)	3	160	1	161	0.0%	3.02 [0.32, 28.71]
**Subtotal (95% CI)**		**0**		**0**		**Not estimable**
Total events	0		0			
Heterogeneity: Not applicable						
Test for overall effect: Not applicable						
**12.16.16 General musculoskeletal and connective tissue disorders ('unsolicited'): Cervarix vs. Twinrix: hepatitis A and B vaccine (females, age 8-15; follow-up until 12 months)**						
HPV-029: Cervarix vs. Twinrix (f, 8-15; 12)	9	270	4	271	0.0%	2.26 [0.70, 7.24]
**Subtotal (95% CI)**		**0**		**0**		**Not estimable**
Total events	0		0			
Heterogeneity: Not applicable						
Test for overall effect: Not applicable						
**12.16.17 General musculoskeletal and connective tissue disorders ('systemic adverse events'): Gardasil vs. carrier solution (females and males, age 9-16; follow-up until 18 months)**						
V501-018: Gardasil vs. carrier solu. (f&m,9-16;18)	52	1184	19	597	2.9%	1.38 [0.82, 2.31]
**Subtotal (95% CI)**		**1184**		**597**	**2.9%**	**1.38 [0.82, 2.31]**
Total events	52		19			
Heterogeneity: Not applicable						
Test for overall effect: Z = 1.22 (P = 0.22)						
**12.16.18 General musculoskeletal and connective tissue disorders ('systemic adverse events'): Gardasil vs. AAHS (males, age 15-27; follow-up until 36 months)**						
V501-020: Gardasil vs. AAHS (m, 15-27; 36)	61	2032	50	2033	4.3%	1.22 [0.84, 1.77]
**Subtotal (95% CI)**		**2032**		**2033**	**4.3%**	**1.22 [0.84, 1.77]**
Total events	61		50			
Heterogeneity: Not applicable						
Test for overall effect: Z = 1.06 (P = 0.29)						
**12.16.19 General musculoskeletal and connective tissue disorders ('systemic adverse events'): Gardasil 9 vs. saline placebo (females, age 12-26; follow-up until 7 months)**						
V503-006: Gardasil 9 vs. saline (f, 12-26;7)	54	618	23	306	3.3%	1.16 [0.73, 1.86]
**Subtotal (95% CI)**		**618**		**306**	**3.3%**	**1.16 [0.73, 1.86]**
Total events	54		23			
Heterogeneity: Not applicable						
Test for overall effect: Z = 0.63 (P = 0.53)						
**12.16.20 General musculoskeletal and connective tissue disorders ('systemic adverse events'): HPV 16 vaccine vs. AAHS (females, age 16-25; follow-up until 48 months)**						
V501-005: HPV 16 vaccine vs. AAHS (f, 16-25; 48)	143	1204	134	1205	6.5%	1.07 [0.86, 1.33]
**Subtotal (95% CI)**		**1204**		**1205**	**6.5%**	**1.07 [0.86, 1.33]**
Total events	143		134			
Heterogeneity: Not applicable						
Test for overall effect: Z = 0.58 (P = 0.56)						
**Total (95% CI)**		**21041**		**20491**	**100.0%**	**1.34 [1.21, 1.49]**
Total events	6005		4683			
Heterogeneity: $Tau^2 = 0.03$; $Chi^2 = 126.70$, df = 16 (P < 0.00001); $I^2 = 87\%$						
Test for overall effect: Z = 5.42 (P < 0.00001)						
Test for subgroup differences: $Chi^2 = 36.63$, df = 12 (P = 0.0003), $I^2 = 67.2\%$						

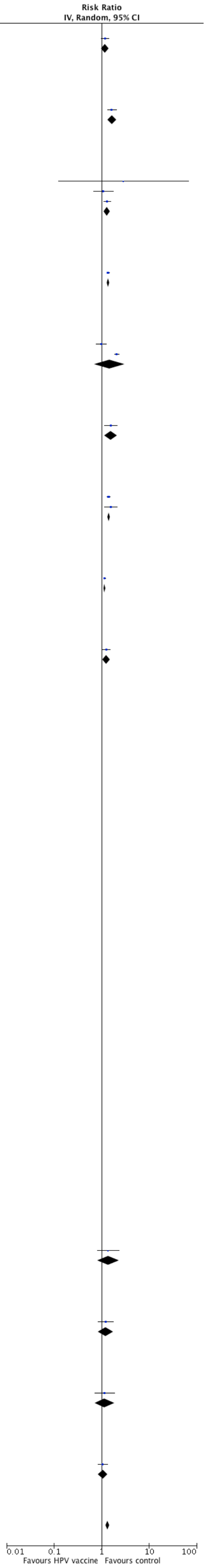

*12.16. Risk ratio for ‘solicited’ (GlaxoSmithKline): **1.38 [1.22, 1.55]**; risk ratio for ‘unsolicited’ (GlaxoSmithKline): 1.26 [0.91, 1.75]; risk ratio for ‘systemic adverse events’ (Merck Sharp & Dohme): 1.14 [0.96, 1.35]. To avoid double counting of participants in the total risk ratio estimate, we excluded the ‘unsolicited’ adverse events from total risk ratio estimate for studies that reported ‘solicited’ adverse events.

12.17. General harms ('solicited,' 'unsolicited' and 'systemic adverse events'*) reported within the MedDRA system organ class 'neoplasms benign, malignant and unspecified (incl. cysts and polyps) (10029104)': intention to treat analysis

Study or Subgroup	HPV vaccine Events	HPV vaccine Total	Control Events	Control Total	Weight	Risk Ratio IV, Random, 95% CI
**12.17.1 General neoplasms ('unsolicited'): Cervarix vs. Al(OH)3 (females, age 18-35; follow-up until 7 months)**						
HPV-031: Cervarix vs. Al(OH)3 (f, 18-35; 7)	0	176	1	178	25.0%	0.34 [0.01, 8.22]
**Subtotal (95% CI)**		**176**		**178**	**25.0%**	**0.34 [0.01, 8.22]**
Total events	0		1			
Heterogeneity: Not applicable						
Test for overall effect: Z = 0.67 (P = 0.50)						
**12.17.2 General neoplasms ('unsolicited'): Cervarix vs. Havrix: hepatitis A vaccine (females, age 9-15; follow-up until 7 months)**						
HPV-033: Cervarix vs. Havrix (f, 9-15; 7)	0	160	1	161	25.0%	0.34 [0.01, 8.17]
**Subtotal (95% CI)**		**160**		**161**	**25.0%**	**0.34 [0.01, 8.17]**
Total events	0		1			
Heterogeneity: Not applicable						
Test for overall effect: Z = 0.67 (P = 0.50)						
**12.17.3 General neoplasms ('systemic adverse events'): Gardasil vs. carrier solution (females and males, age 9-16; follow-up until 18 months)**						
V501-018: Gardasil vs. carrier solu. (f&m,9-16;18)	1	1184	0	597	25.0%	1.51 [0.06, 37.11]
**Subtotal (95% CI)**		**1184**		**597**	**25.0%**	**1.51 [0.06, 37.11]**
Total events	1		0			
Heterogeneity: Not applicable						
Test for overall effect: Z = 0.25 (P = 0.80)						
**12.17.4 General neoplasms ('systemic adverse events'): Gardasil 9 vs. saline placebo (females, age 12-26; follow-up until 7 months)**						
V503-006: Gardasil 9 vs. saline (f, 12-26;7)	1	618	0	306	25.0%	1.49 [0.06, 36.42]
**Subtotal (95% CI)**		**618**		**306**	**25.0%**	**1.49 [0.06, 36.42]**
Total events	1		0			
Heterogeneity: Not applicable						
Test for overall effect: Z = 0.24 (P = 0.81)						
**Total (95% CI)**		**2138**		**1242**	**100.0%**	**0.71 [0.14, 3.51]**
Total events	2		2			
Heterogeneity: Tau² = 0.00; Chi² = 0.84, df = 3 (P = 0.84); I² = 0%						
Test for overall effect: Z = 0.42 (P = 0.67)						
Test for subgroup differences: Chi² = 0.84, df = 3 (P = 0.84), I² = 0%						

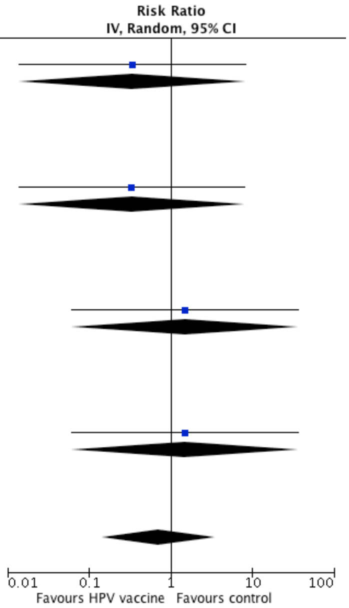

*12.17. Risk ratio for 'solicited' (GlaxoSmithKline): not applicable; risk ratio for 'unsolicited' (GlaxoSmithKline): 0.34 [0.04, 3.22]; risk ratio for 'systemic adverse events' (Merck Sharp & Dohme): 1.50 [0.16, 14.41].

12.18. General harms (‘solicited,’ ‘unsolicited’ and ‘systemic adverse events’*) reported within the MedDRA system organ class ‘nervous system disorders (10029205)': intention to treat analysis

Study or Subgroup	HPV vaccine Events	HPV vaccine Total	Control Events	Control Total	Weight	Risk Ratio IV, Random, 95% CI
**12.18.1 General nervous system disorders ('solicited'): Cervarix vs. Al(OH)3 (females, age 9-17; follow-up until 12 months)**						
HPV-058: Cervarix vs. Al(OH)3 (f, 9-17; 12)	123	374	99	376	3.4%	1.25 [1.00, 1.56]
**Subtotal (95% CI)**		**374**		**376**	**3.4%**	**1.25 [1.00, 1.56]**
Total events	123		99			
Heterogeneity: Not applicable						
Test for overall effect: Z = 1.96 (P = 0.05)						
**12.18.2 General nervous system disorders ('solicited'): Cervarix vs. Al(OH)3 (females, age 15-26; follow-up until 27 months)**						
HPV-001: Cervarix vs. Al(OH)3 (f, 15-26; 27)	331	560	329	553	9.4%	0.99 [0.90, 1.10]
HPV-038: Cervarix vs. Al(OH)3 (f, 15-25; 7)	73	149	30	76	1.8%	1.24 [0.90, 1.71]
**Subtotal (95% CI)**		**709**		**629**	**11.3%**	**1.05 [0.87, 1.27]**
Total events	404		359			
Heterogeneity: $Tau^2 = 0.01$; $Chi^2 = 1.67$, df = 1 (P = 0.20); $I^2 = 40\%$						
Test for overall effect: Z = 0.51 (P = 0.61)						
**12.18.3 General nervous system disorders ('solicited'): Cervarix vs. Al(OH)3 (females, age 18-35; follow-up until 12 months)**						
HPV-003: Cervarix vs. Al(OH)3 (f, 18-30; 12)	16	31	21	30	1.2%	0.74 [0.49, 1.12]
HPV-031: Cervarix vs. Al(OH)3 (f, 18-35; 7)	72	176	71	178	2.8%	1.03 [0.80, 1.32]
HPV-035: Cervarix vs. Al(OH)3 (f, 18-35; 7)	67	150	54	150	2.4%	1.24 [0.94, 1.64]
**Subtotal (95% CI)**		**357**		**358**	**6.3%**	**1.02 [0.79, 1.31]**
Total events	155		146			
Heterogeneity: $Tau^2 = 0.03$; $Chi^2 = 4.22$, df = 2 (P = 0.12); $I^2 = 53\%$						
Test for overall effect: Z = 0.12 (P = 0.91)						
**12.18.4 General nervous system disorders ('solicited'): Cervarix vs. Al(OH)3 (females, age 24-72; follow-up until 36 months)**						
HPV-015: Cervarix vs. Al(OH)3 (f, 24-72; 36)	1165	2882	1074	2871	12.3%	1.08 [1.01, 1.15]
**Subtotal (95% CI)**		**2882**		**2871**	**12.3%**	**1.08 [1.01, 1.15]**
Total events	1165		1074			
Heterogeneity: Not applicable						
Test for overall effect: Z = 2.34 (P = 0.02)						
**12.18.5 General nervous system disorders ('solicited'): Cervarix vs. Engerix: hepatitis B vaccine (females and males, age 9-16; follow-up until 72 months)**						
HPV-030: Cervarix vs. Engerix (f, 9-15; 12)	131	247	129	246	5.2%	1.01 [0.86, 1.20]
HPV-040: Cervarix vs. Engerix (f & m, 12-16; 72)	261	604	371	1028	7.6%	1.20 [1.06, 1.35]
**Subtotal (95% CI)**		**851**		**1274**	**12.8%**	**1.11 [0.94, 1.31]**
Total events	392		500			
Heterogeneity: $Tau^2 = 0.01$; $Chi^2 = 2.55$, df = 1 (P = 0.11); $I^2 = 61\%$						
Test for overall effect: Z = 1.26 (P = 0.21)						
**12.18.6 General nervous system disorders ('solicited'): Cervarix vs. Engerix: hepatitis B vaccine (females, age 26-46; follow-up until 12 months)**						
HPV-069: Cervarix vs. Engerix (f, 26-46; 12)	71	606	46	606	1.6%	1.54 [1.08, 2.20]
**Subtotal (95% CI)**		**606**		**606**	**1.6%**	**1.54 [1.08, 2.20]**
Total events	71		46			
Heterogeneity: Not applicable						
Test for overall effect: Z = 2.41 (P = 0.02)						
**12.18.7 General nervous system disorders ('solicited'): Cervarix vs. Havrix: hepatitis A vaccine (females, age 9-15; follow-up until 12 months)**						
HPV-013: Cervarix vs. Havrix (f, 10-14; 12)	516	1035	464	1032	10.0%	1.11 [1.01, 1.21]
HPV-033: Cervarix vs. Havrix (f, 9-15; 7)	44	160	53	161	1.7%	0.84 [0.60, 1.17]
**Subtotal (95% CI)**		**1195**		**1193**	**11.7%**	**1.01 [0.78, 1.31]**
Total events	560		517			
Heterogeneity: $Tau^2 = 0.02$; $Chi^2 = 2.56$, df = 1 (P = 0.11); $I^2 = 61\%$						
Test for overall effect: Z = 0.07 (P = 0.94)						
**12.18.8 General nervous system disorders ('solicited'): Cervarix vs. Havrix: hepatitis A vaccine (females, age 15-25; follow-up until 48 months)**						
HPV-008: Cervarix vs. Havrix (f, 15-25; 48)	1668	9319	1583	9325	12.5%	1.05 [0.99, 1.12]
**Subtotal (95% CI)**		**9319**		**9325**	**12.5%**	**1.05 [0.99, 1.12]**
Total events	1668		1583			
Heterogeneity: Not applicable						
Test for overall effect: Z = 1.66 (P = 0.10)						
**12.18.9 General nervous system disorders ('solicited'): Cervarix vs. Twinrix: hepatitis A and B vaccine (females, age 8-15; follow-up until 12 months)**						
HPV-029: Cervarix vs. Twinrix (f, 8-15; 12)	124	270	110	271	4.2%	1.13 [0.93, 1.37]
**Subtotal (95% CI)**		**270**		**271**	**4.2%**	**1.13 [0.93, 1.37]**
Total events	124		110			
Heterogeneity: Not applicable						
Test for overall effect: Z = 1.25 (P = 0.21)						
**12.18.13 General nervous system disorders ('unsolicited'): Cervarix vs. Al(OH)3 (females, age 9-17; follow-up until 12 months)**						
HPV-058: Cervarix vs. Al(OH)3 (f, 9-17; 12)	2	374	1	376	0.0%	2.01 [0.18, 22.08]
**Subtotal (95% CI)**		**0**		**0**		**Not estimable**
Total events	0		0			
Heterogeneity: Not applicable						
Test for overall effect: Not applicable						
**12.18.14 General nervous system disorders ('unsolicited'): Cervarix vs. Al(OH)3 (females, age 15-25; follow-up until 7 months)**						
HPV-038: Cervarix vs. Al(OH)3 (f, 15-25; 7)	13	149	7	76	0.0%	0.95 [0.39, 2.28]
**Subtotal (95% CI)**		**0**		**0**		**Not estimable**
Total events	0		0			
Heterogeneity: Not applicable						
Test for overall effect: Not applicable						
**12.18.15 General nervous system disorders ('unsolicited'): Cervarix vs. Al(OH)3 (females, age 18-35; follow-up until 12 months)**						
HPV-003: Cervarix vs. Al(OH)3 (f, 18-30; 12)	5	31	0	30	0.0%	10.66 [0.61, 184.70]
HPV-031: Cervarix vs. Al(OH)3 (f, 18-35; 7)	11	176	2	178	0.0%	5.56 [1.25, 24.73]
HPV-035: Cervarix vs. Al(OH)3 (f, 18-35; 7)	24	150	14	150	0.0%	1.71 [0.92, 3.18]
**Subtotal (95% CI)**		**0**		**0**		**Not estimable**
Total events	0		0			
Heterogeneity: Not applicable						
Test for overall effect: Not applicable						
**12.18.16 General nervous system disorders ('unsolicited'): Cervarix vs. Engerix: hepatitis B vaccine (females and males, age 9-16; follow-up until 72 months)**						
HPV-030: Cervarix vs. Engerix (f, 9-15; 12)	33	247	17	246	0.0%	1.93 [1.11, 3.38]
HPV-040: Cervarix vs. Engerix (f & m, 12-16; 72)	34	1908	30	3128	0.0%	1.86 [1.14, 3.03]
**Subtotal (95% CI)**		**0**		**0**		**Not estimable**
Total events	0		0			
Heterogeneity: Not applicable						
Test for overall effect: Not applicable						
**12.18.17 General nervous system disorders ('unsolicited'): Cervarix vs. Havrix: hepatitis A vaccine (females, age 9-15; follow-up until 7 months)**						
HPV-033: Cervarix vs. Havrix (f, 9-15; 7)	6	160	3	161	0.0%	2.01 [0.51, 7.91]
**Subtotal (95% CI)**		**0**		**0**		**Not estimable**
Total events	0		0			
Heterogeneity: Not applicable						
Test for overall effect: Not applicable						
**12.18.18 General nervous system disorders ('unsolicited'): Cervarix vs. Twinrix: hepatitis A and B vaccine (females, age 8-15; follow-up until 12 months)**						
HPV-029: Cervarix vs. Twinrix (f, 8-15; 12)	17	270	20	271	0.0%	0.85 [0.46, 1.59]
**Subtotal (95% CI)**		**0**		**0**		**Not estimable**
Total events	0		0			
Heterogeneity: Not applicable						
Test for overall effect: Not applicable						
**12.18.19 General nervous system disorders ('systemic adverse events'): Gardasil vs. carrier solution (females and males, age 9-16; follow-up until 18 months)**						
V501-018: Gardasil vs. carrier solu. (f&m,9-16;18)	258	1184	127	597	4.4%	1.02 [0.85, 1.24]
**Subtotal (95% CI)**		**1184**		**597**	**4.4%**	**1.02 [0.85, 1.24]**
Total events	258		127			
Heterogeneity: Not applicable						
Test for overall effect: Z = 0.25 (P = 0.80)						
**12.18.20 General nervous system disorders ('systemic adverse events'): Gardasil vs. AAHS (males, age 15-27; follow-up until 36 months)**						
V501-020: Gardasil vs. AAHS (m, 15-27; 36)	307	2032	231	2033	5.6%	1.33 [1.13, 1.56]
**Subtotal (95% CI)**		**2032**		**2033**	**5.6%**	**1.33 [1.13, 1.56]**
Total events	307		231			
Heterogeneity: Not applicable						
Test for overall effect: Z = 3.51 (P = 0.0005)						
**12.18.21 General nervous system disorders ('systemic adverse events'): Gardasil 9 vs. saline placebo (females, age 12-26; follow-up until 7 months)**						
V503-006: Gardasil 9 vs. saline (f, 12-26;7)	206	618	93	306	3.9%	1.10 [0.90, 1.34]
**Subtotal (95% CI)**		**618**		**306**	**3.9%**	**1.10 [0.90, 1.34]**
Total events	206		93			
Heterogeneity: Not applicable						
Test for overall effect: Z = 0.89 (P = 0.37)						
**12.18.22 General nervous system disorders ('systemic adverse events'): HPV 16 vaccine vs. AAHS (females, age 16-25; follow-up until 48 months)**						
V501-005: HPV 16 vaccine vs. AAHS (f, 16-25; 48)	534	1204	537	1205	10.1%	1.00 [0.91, 1.09]
**Subtotal (95% CI)**		**1204**		**1205**	**10.1%**	**1.00 [0.91, 1.09]**
Total events	534		537			
Heterogeneity: Not applicable						
Test for overall effect: Z = 0.10 (P = 0.92)						
**Total (95% CI)**		**21601**		**21044**	**100.0%**	**1.09 [1.04, 1.14]**
Total events	5967		5422			
Heterogeneity: $Tau^2 = 0.00$; $Chi^2 = 30.18$, df = 17 (P = 0.03); $I^2 = 44\%$						
Test for overall effect: Z = 3.50 (P = 0.0005)						
Test for subgroup differences: $Chi^2 = 17.06$, df = 12 (P = 0.15), $I^2 = 29.7\%$						

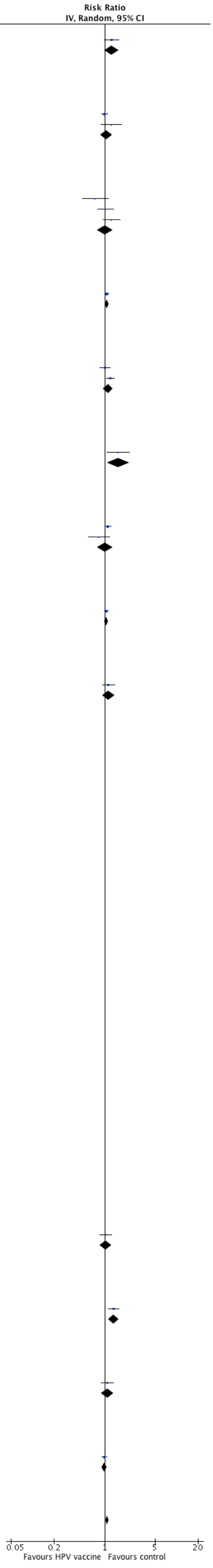

*12.18. Risk ratio for 'solicited’ only: **1.08 [1.03, 1.14]**; risk ratio for ‘unsolicited’ (GlaxoSmithKline): **1.62 [1.17, 2.24]**; risk ratio for ‘systemic adverse events’ (Merck Sharp & Dohme): 1.10 [0.96, 1.26]. To avoid double counting of participants in the total risk ratio estimate, we excluded the ‘unsolicited’ adverse events from total risk ratio estimate for studies that reported ‘solicited’ adverse events.

12.19. General harms (‘solicited,’ ‘unsolicited’ and ‘systemic adverse events’*) reported within the MedDRA system organ class ‘pregnancy, puerperium and perinatal conditions (10036585)': intention to treat analysis

Study or Subgroup	HPV vaccine Events	HPV vaccine Total	Control Events	Control Total	Weight	Risk Ratio IV, Random, 95% CI
**12.19.1 General pregnancy, puerperium and perinatal conditions ('unsolicited'): Cervarix vs. Al(OH)3 (females, age 18-35; follow-up until 7 months)**						
HPV-031: Cervarix vs. Al(OH)3 (f, 18-35; 7)	1	31	0	30	100.0%	2.91 [0.12, 68.66]
**Subtotal (95% CI)**		**31**		**30**	**100.0%**	**2.91 [0.12, 68.66]**
Total events	1		0			
Heterogeneity: Not applicable						
Test for overall effect: Z = 0.66 (P = 0.51)						
**Total (95% CI)**		**31**		**30**	**100.0%**	**2.91 [0.12, 68.66]**
Total events	1		0			
Heterogeneity: Not applicable						
Test for overall effect: Z = 0.66 (P = 0.51)						
Test for subgroup differences: Not applicable						

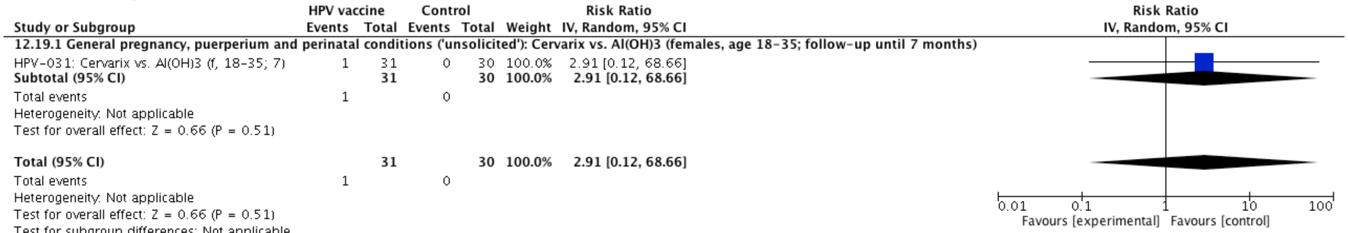

*12.19. Risk ratio for ‘solicited’ (GlaxoSmithKline): not applicable; risk ratio for ‘unsolicited’ (GlaxoSmithKline): 2.91 [0.12, 68.66]; risk ratio for ‘systemic adverse events’ (Merck Sharp & Dohme): not applicable.

12.20. General harms (‘solicited,’ ‘unsolicited’ and ‘systemic adverse events’*) reported within the MedDRA system organ class ‘psychiatric disorders (10037175)': intention to treat analysis

Study or Subgroup	HPV vaccine Events	HPV vaccine Total	Control Events	Control Total	Weight	Risk Ratio IV, Random, 95% CI
**12.20.1 General psychiatric disorders ('unsolicited'): Cervarix vs. Al(OH)3 (females, age 18-35; follow-up until 12 months)**						
HPV-003: Cervarix vs. Al(OH)3 (f, 18-30; 12)	1	31	0	30	1.5%	2.91 [0.12, 68.66]
HPV-035: Cervarix vs. Al(OH)3 (f, 18-35; 7)	1	150	5	150	3.3%	0.20 [0.02, 1.69]
**Subtotal (95% CI)**		**181**		**180**	**4.9%**	**0.59 [0.04, 7.65]**
Total events	2		5			
Heterogeneity: Tau^2 = 1.69; Chi^2 = 1.89, df = 1 (P = 0.17); I^2 = 47%						
Test for overall effect: Z = 0.41 (P = 0.68)						
**12.20.2 General psychiatric disorders ('unsolicited'): Cervarix vs. Engerix: hepatitis B vaccine (females and males, age 9-16; follow-up until 72 months)**						
HPV-030: Cervarix vs. Engerix (f, 9-15; 12)	0	247	2	246	1.7%	0.20 [0.01, 4.13]
HPV-040: Cervarix vs. Engerix (f & m, 12-16; 72)	0	1908	2	3128	1.7%	0.33 [0.02, 6.82]
**Subtotal (95% CI)**		**2155**		**3374**	**3.3%**	**0.26 [0.03, 2.18]**
Total events	0		4			
Heterogeneity: Tau^2 = 0.00; Chi^2 = 0.05, df = 1 (P = 0.82); I^2 = 0%						
Test for overall effect: Z = 1.25 (P = 0.21)						
**12.20.3 General psychiatric disorders ('unsolicited'): Cervarix vs. Havrix: hepatitis A vaccine (females, age 9-15; follow-up until 7 months)**						
HPV-033: Cervarix vs. Havrix (f, 9-15; 7)	1	160	0	161	1.5%	3.02 [0.12, 73.55]
**Subtotal (95% CI)**		**160**		**161**	**1.5%**	**3.02 [0.12, 73.55]**
Total events	1		0			
Heterogeneity: Not applicable						
Test for overall effect: Z = 0.68 (P = 0.50)						
**12.20.4 General psychiatric disorders ('unsolicited'): Cervarix vs. Twinrix: hepatitis A and B vaccine (females, age 8-15; follow-up until 12 months)**						
HPV-029: Cervarix vs. Twinrix (f, 8-15; 12)	1	270	0	271	1.5%	3.01 [0.12, 73.59]
**Subtotal (95% CI)**		**270**		**271**	**1.5%**	**3.01 [0.12, 73.59]**
Total events	1		0			
Heterogeneity: Not applicable						
Test for overall effect: Z = 0.68 (P = 0.50)						
**12.20.5 General psychiatric disorders ('systemic adverse events'): Gardasil vs. carrier solution (females and males, age 9-16; follow-up until 18 months)**						
V501-018: Gardasil vs. carrier solu. (f&m,9-16;18)	3	1184	3	597	6.0%	0.50 [0.10, 2.49]
**Subtotal (95% CI)**		**1184**		**597**	**6.0%**	**0.50 [0.10, 2.49]**
Total events	3		3			
Heterogeneity: Not applicable						
Test for overall effect: Z = 0.84 (P = 0.40)						
**12.20.6 General psychiatric disorders ('systemic adverse events'): Gardasil vs. AAHS (males, age 15-27; follow-up until 36 months)**						
V501-020: Gardasil vs. AAHS (m, 15-27; 36)	7	2032	10	2033	16.4%	0.70 [0.27, 1.84]
**Subtotal (95% CI)**		**2032**		**2033**	**16.4%**	**0.70 [0.27, 1.84]**
Total events	7		10			
Heterogeneity: Not applicable						
Test for overall effect: Z = 0.72 (P = 0.47)						
**12.20.7 General psychiatric disorders ('systemic adverse events'): Gardasil 9 vs. saline placebo (females, age 12-26; follow-up until 7 months)**						
V503-006: Gardasil 9 vs. saline (f, 12-26;7)	4	618	2	306	5.3%	0.99 [0.18, 5.38]
**Subtotal (95% CI)**		**618**		**306**	**5.3%**	**0.99 [0.18, 5.38]**
Total events	4		2			
Heterogeneity: Not applicable						
Test for overall effect: Z = 0.01 (P = 0.99)						
**12.20.8 General psychiatric disorders ('systemic adverse events'): HPV 16 vaccine vs. AAHS (females, age 16-25; follow-up until 48 months)**						
V501-005: HPV 16 vaccine vs. AAHS (f, 16-25; 48)	31	1204	29	1205	61.1%	1.07 [0.65, 1.76]
**Subtotal (95% CI)**		**1204**		**1205**	**61.1%**	**1.07 [0.65, 1.76]**
Total events	31		29			
Heterogeneity: Not applicable						
Test for overall effect: Z = 0.26 (P = 0.79)						
**Total (95% CI)**		**7804**		**8127**	**100.0%**	**0.90 [0.61, 1.33]**
Total events	49		53			
Heterogeneity: Tau^2 = 0.00; Chi^2 = 6.15, df = 9 (P = 0.73); I^2 = 0%						
Test for overall effect: Z = 0.55 (P = 0.59)						
Test for subgroup differences: Chi^2 = 3.76, df = 7 (P = 0.81), I^2 = 0%						

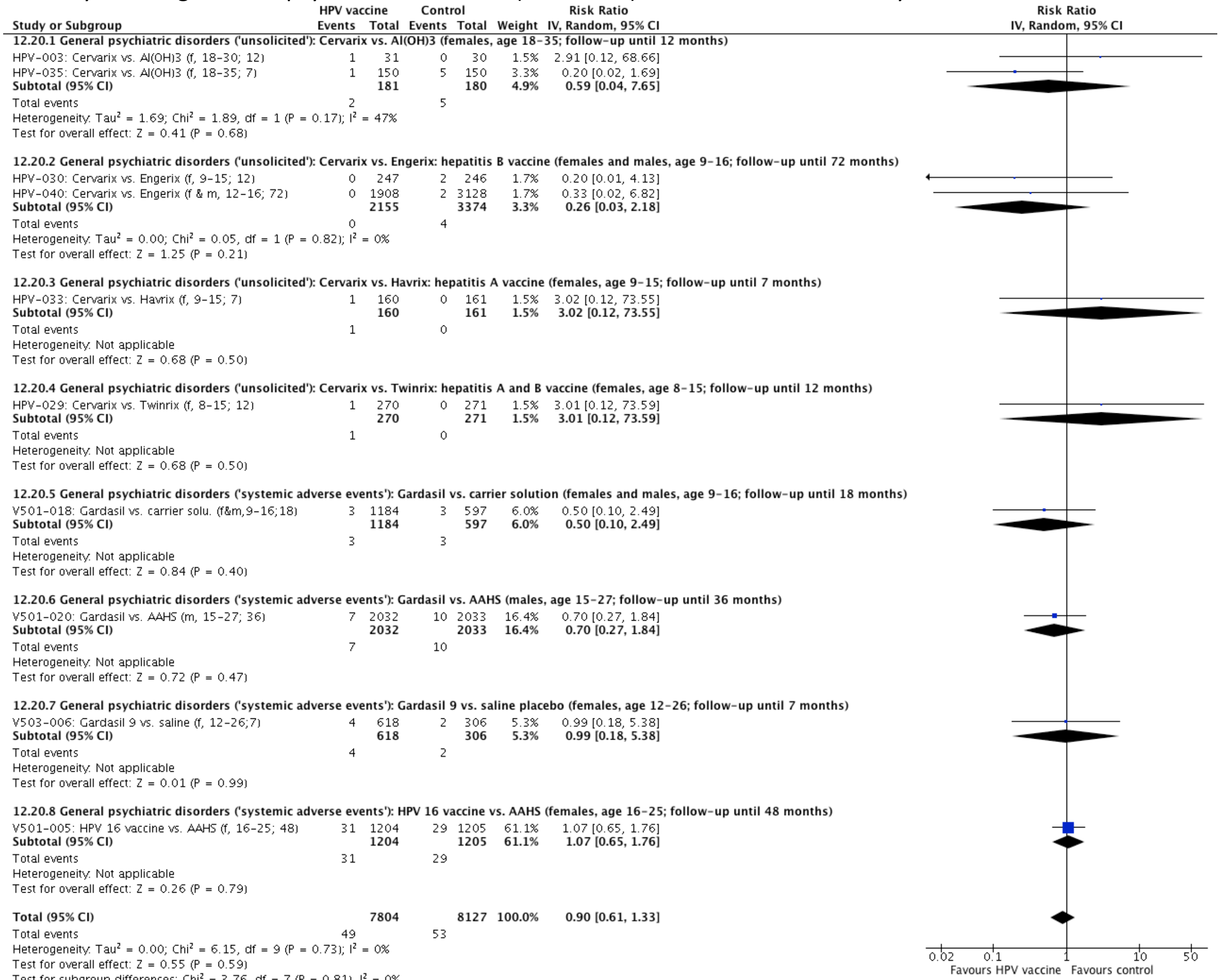

*12.20. Risk ratio for ‘solicited’ (GlaxoSmithKline): not applicable; risk ratio for ‘unsolicited’ (GlaxoSmithKline): 0.64 [0.20, 2.06]; risk ratio for ‘systemic adverse events’ (Merck Sharp & Dohme): 0.94 [0.62, 1.42].

12.21. General harms (‘solicited,’ ‘unsolicited’ and ‘systemic adverse events’*) reported within the MedDRA system organ class 'renal and urinary disorders (10038359)': intention to treat analysis

Study or Subgroup	HPV vaccine Events	HPV vaccine Total	Control Events	Control Total	Weight	Risk Ratio IV, Random, 95% CI
**12.21.1 General renal and urinary disorders ('unsolicited'): Cervarix vs. Al(OH)3 (females, age 18-35; follow-up until 7 months)**						
HPV-031: Cervarix vs. Al(OH)3 (f, 18-35; 7)	0	176	1	178	9.8%	0.34 [0.01, 8.22]
**Subtotal (95% CI)**		**176**		**178**	**9.8%**	**0.34 [0.01, 8.22]**
Total events	0		1			
Heterogeneity: Not applicable						
Test for overall effect: Z = 0.67 (P = 0.50)						
**12.21.2 General renal and urinary disorders ('unsolicited'): Cervarix vs. Engerix: hepatitis B vaccine (females and males, age 9-16; follow-up until 72 months)**						
HPV-030: Cervarix vs. Engerix (f, 9-15; 12)	1	247	1	246	13.1%	1.00 [0.06, 15.83]
HPV-040: Cervarix vs. Engerix (f & m, 12-16; 72)	0	1908	1	3128	9.8%	0.55 [0.02, 13.40]
**Subtotal (95% CI)**		**2155**		**3374**	**22.9%**	**0.77 [0.10, 6.25]**
Total events	1		2			
Heterogeneity: Tau² = 0.00; Chi² = 0.08, df = 1 (P = 0.78); I² = 0%						
Test for overall effect: Z = 0.24 (P = 0.81)						
**12.21.3 General renal and urinary disorders ('systemic adverse events'): Gardasil vs. carrier solution (females and males, age 9-16; follow-up until 18 months)**						
V501-018: Gardasil vs. carrier solu. (f&m,9-16;18)	4	1184	2	597	35.0%	1.01 [0.19, 5.49]
**Subtotal (95% CI)**		**1184**		**597**	**35.0%**	**1.01 [0.19, 5.49]**
Total events	4		2			
Heterogeneity: Not applicable						
Test for overall effect: Z = 0.01 (P = 0.99)						
**12.21.4 General renal and urinary disorders ('systemic adverse events'): Gardasil vs. AAHS (males, age 15-27; follow-up until 36 months)**						
V501-020: Gardasil vs. AAHS (m, 15-27; 36)	6	2032	1	2033	22.4%	6.00 [0.72, 49.82]
**Subtotal (95% CI)**		**2032**		**2033**	**22.4%**	**6.00 [0.72, 49.82]**
Total events	6		1			
Heterogeneity: Not applicable						
Test for overall effect: Z = 1.66 (P = 0.10)						
**12.21.5 General renal and urinary disorders ('systemic adverse events'): Gardasil 9 vs. saline placebo (females, age 12-26; follow-up until 7 months)**						
V503-006: Gardasil 9 vs. saline (f, 12-26;7)	1	618	0	306	9.8%	1.49 [0.06, 36.42]
**Subtotal (95% CI)**		**618**		**306**	**9.8%**	**1.49 [0.06, 36.42]**
Total events	1		0			
Heterogeneity: Not applicable						
Test for overall effect: Z = 0.24 (P = 0.81)						
**Total (95% CI)**		**6165**		**6488**	**100.0%**	**1.32 [0.48, 3.59]**
Total events	12		6			
Heterogeneity: Tau² = 0.00; Chi² = 3.10, df = 5 (P = 0.68); I² = 0%						
Test for overall effect: Z = 0.54 (P = 0.59)						
Test for subgroup differences: Chi² = 3.03, df = 4 (P = 0.55), I² = 0%						

Risk Ratio
IV, Random, 95% CI

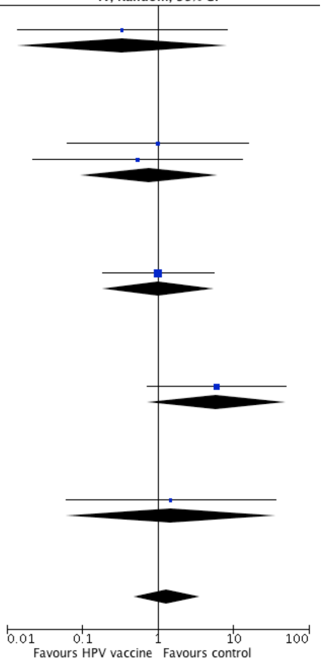

*12.21. Risk ratio for ‘solicited’ (GlaxoSmithKline): not applicable; risk ratio for ‘unsolicited’ (GlaxoSmithKline): 0.60 [0.10, 3.46]; risk ratio for ‘systemic adverse events’ (Merck Sharp & Dohme): 1.94 [0.57, 6.57].

12.22. General harms (‘solicited,’ ‘unsolicited’ and ‘systemic adverse events’*) reported within the MedDRA system organ class ‘reproductive system and breast disorders (10038604)': intention to treat analysis

Study or Subgroup	HPV vaccine Events	HPV vaccine Total	Control Events	Control Total	Weight	Risk Ratio IV, Random, 95% CI
**12.22.1 General reproductive system and breast disorders ('unsolicited'): Cervarix vs. Al(OH)3 (females, age 9-17; follow-up until 12 months)**						
HPV-058: Cervarix vs. Al(OH)3 (f, 9-17; 12)	0	374	1	376	0.4%	0.34 [0.01, 8.20]
**Subtotal (95% CI)**		**374**		**376**	**0.4%**	**0.34 [0.01, 8.20]**
Total events	0		1			
Heterogeneity: Not applicable						
Test for overall effect: Z = 0.67 (P = 0.50)						
**12.22.2 General reproductive system and breast disorders ('unsolicited'): Cervarix vs. Al(OH)3 (females, age 15-25; follow-up until 7 months)**						
HPV-038: Cervarix vs. Al(OH)3 (f, 15-25; 7)	16	149	4	76	3.7%	2.04 [0.71, 5.89]
**Subtotal (95% CI)**		**149**		**76**	**3.7%**	**2.04 [0.71, 5.89]**
Total events	16		4			
Heterogeneity: Not applicable						
Test for overall effect: Z = 1.32 (P = 0.19)						
**12.22.3 General reproductive system and breast disorders ('unsolicited'): Cervarix vs. Al(OH)3 (females, age 18-35; follow-up until 12 months)**						
HPV-003: Cervarix vs. Al(OH)3 (f, 18-30; 12)	5	31	1	30	1.0%	4.84 [0.60, 39.02]
HPV-031: Cervarix vs. Al(OH)3 (f, 18-35; 7)	4	176	3	178	1.9%	1.35 [0.31, 5.94]
HPV-035: Cervarix vs. Al(OH)3 (f, 18-35; 7)	16	150	11	150	7.8%	1.45 [0.70, 3.03]
**Subtotal (95% CI)**		**357**		**358**	**10.7%**	**1.60 [0.85, 2.99]**
Total events	25		15			
Heterogeneity: Tau² = 0.00; Chi² = 1.20, df = 2 (P = 0.55); I² = 0%						
Test for overall effect: Z = 1.47 (P = 0.14)						
**12.22.4 General reproductive system and breast disorders ('unsolicited'): Cervarix vs. Engerix: hepatitis B vaccine (females and males, age 9-15; follow-up until 12 months)**						
HPV-030: Cervarix vs. Engerix (f, 9-15; 12)	7	247	9	246	4.4%	0.77 [0.29, 2.05]
**Subtotal (95% CI)**		**247**		**246**	**4.4%**	**0.77 [0.29, 2.05]**
Total events	7		9			
Heterogeneity: Not applicable						
Test for overall effect: Z = 0.52 (P = 0.61)						
**12.22.5 General reproductive system and breast disorders ('unsolicited'): Cervarix vs. Engerix: hepatitis B vaccine (females, age 26-46; follow-up until 12 months)**						
HPV-069: Cervarix vs. Engerix (f, 26-46; 12)	3	606	0	606	0.5%	7.00 [0.36, 135.23]
**Subtotal (95% CI)**		**606**		**606**	**0.5%**	**7.00 [0.36, 135.23]**
Total events	3		0			
Heterogeneity: Not applicable						
Test for overall effect: Z = 1.29 (P = 0.20)						
**12.22.6 General reproductive system and breast disorders ('unsolicited'): Cervarix vs. Havrix: hepatitis A vaccine (females, age 9-15; follow-up until 7 months**						
HPV-033: Cervarix vs. Havrix (f, 9-15; 7)	1	160	1	161	0.5%	1.01 [0.06, 15.95]
**Subtotal (95% CI)**		**160**		**161**	**0.5%**	**1.01 [0.06, 15.95]**
Total events	1		1			
Heterogeneity: Not applicable						
Test for overall effect: Z = 0.00 (P = 1.00)						
**12.22.7 General reproductive system and breast disorders ('unsolicited'): Cervarix vs. Twinrix: hepatitis A and B vaccine (females, age 8-15; follow-up until 12 months)**						
HPV-029: Cervarix vs. Twinrix (f, 8-15; 12)	6	270	3	271	2.2%	2.01 [0.51, 7.94]
**Subtotal (95% CI)**		**270**		**271**	**2.2%**	**2.01 [0.51, 7.94]**
Total events	6		3			
Heterogeneity: Not applicable						
Test for overall effect: Z = 0.99 (P = 0.32)						
**12.22.8 General reproductive system and breast disorders ('systemic adverse events'): Gardasil vs. carrier solution (females and males, age 9-16; follow-up until 18 months)**						
V501-018: Gardasil vs. carrier solu. (f&m,9-16;18)	15	1184	8	597	5.8%	0.95 [0.40, 2.22]
**Subtotal (95% CI)**		**1184**		**597**	**5.8%**	**0.95 [0.40, 2.22]**
Total events	15		8			
Heterogeneity: Not applicable						
Test for overall effect: Z = 0.13 (P = 0.90)						
**12.22.9 General reproductive system and breast disorders ('systemic adverse events'): Gardasil vs. AAHS (males, age 15-27; follow-up until 36 months)**						
V501-020: Gardasil vs. AAHS (m, 15-27; 36)	8	2032	9	2033	4.6%	0.89 [0.34, 2.30]
**Subtotal (95% CI)**		**2032**		**2033**	**4.6%**	**0.89 [0.34, 2.30]**
Total events	8		9			
Heterogeneity: Not applicable						
Test for overall effect: Z = 0.24 (P = 0.81)						
**12.22.10 General reproductive system and breast disorders ('systemic adverse events'): Gardasil 9 vs. saline placebo (females, age 12-26; follow-up until 7 months)**						
V503-006: Gardasil 9 vs. saline (f, 12-26;7)	36	618	12	306	10.3%	1.49 [0.78, 2.81]
**Subtotal (95% CI)**		**618**		**306**	**10.3%**	**1.49 [0.78, 2.81]**
Total events	36		12			
Heterogeneity: Not applicable						
Test for overall effect: Z = 1.21 (P = 0.22)						
**12.22.11 General reproductive system and breast disorders ('systemic adverse events'): HPV 16 vaccine vs. AAHS (females, age 16-25; follow-up until 48 months)**						
V501-005: HPV 16 vaccine vs. AAHS (f, 16-25; 48)	96	1204	96	1205	56.9%	1.00 [0.76, 1.31]
**Subtotal (95% CI)**		**1204**		**1205**	**56.9%**	**1.00 [0.76, 1.31]**
Total events	96		96			
Heterogeneity: Not applicable						
Test for overall effect: Z = 0.01 (P = 1.00)						
**Total (95% CI)**		**7201**		**6235**	**100.0%**	**1.13 [0.92, 1.38]**
Total events	213		158			
Heterogeneity: Tau² = 0.00; Chi² = 8.72, df = 12 (P = 0.73); I² = 0%						
Test for overall effect: Z = 1.13 (P = 0.26)						
Test for subgroup differences: Chi² = 7.52, df = 10 (P = 0.68), I² = 0%						

Risk Ratio
IV, Random, 95% CI

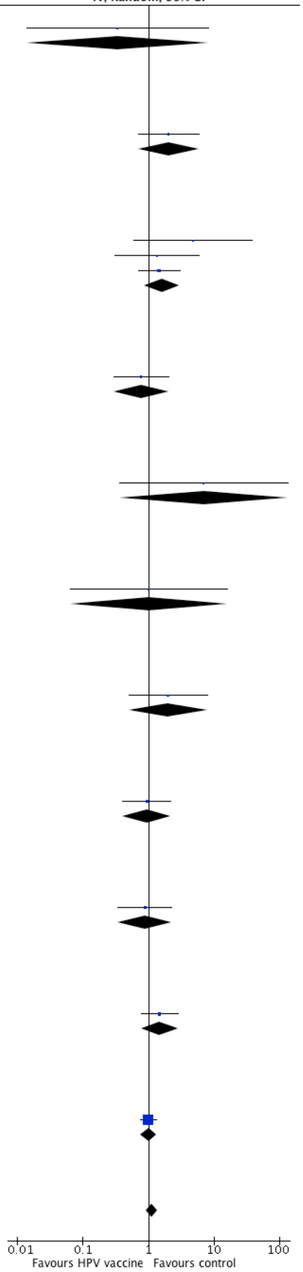

*12.22. Risk ratio for ‘solicited’ (GlaxoSmithKline): not applicable; risk ratio for ‘unsolicited’ (GlaxoSmithKline): 1.46 [0.95, 2.25]; risk ratio for ‘systemic adverse events’ (Merck Sharp & Dohme): 1.04 [0.83, 1.32].

12.23. General harms (‘solicited,’ ‘unsolicited’ and ‘systemic adverse events’*) reported within the MedDRA system organ class ‘respiratory, thoracic and mediastinal disorders (10038738)': intention to treat analysis

Study or Subgroup	HPV vaccine Events	HPV vaccine Total	Control Events	Control Total	Weight	Risk Ratio IV, Random, 95% CI
**12.23.1 General respiratory, thoracic and mediastinal disorders ('unsolicited'): Cervarix vs. Al(OH)3 (females, age 9-17; follow-up until 12 months)**						
HPV-058: Cervarix vs. Al(OH)3 (f, 9-17; 12)	4	374	5	376	1.1%	0.80 [0.22, 2.97]
**Subtotal (95% CI)**		**374**		**376**	**1.1%**	**0.80 [0.22, 2.97]**
Total events	4		5			
Heterogeneity: Not applicable						
Test for overall effect: Z = 0.33 (P = 0.74)						
**12.23.2 General respiratory, thoracic and mediastinal disorders ('unsolicited'): Cervarix vs. Al(OH)3 (females, age 15-25; follow-up until 7 months)**						
HPV-038: Cervarix vs. Al(OH)3 (f, 15-25; 7)	1	149	3	76	0.4%	0.17 [0.02, 1.61]
**Subtotal (95% CI)**		**149**		**76**	**0.4%**	**0.17 [0.02, 1.61]**
Total events	1		3			
Heterogeneity: Not applicable						
Test for overall effect: Z = 1.55 (P = 0.12)						
**12.23.3 General respiratory, thoracic and mediastinal disorders ('unsolicited'): Cervarix vs. Al(OH)3 (females, age 18-35; follow-up until 12 months)**						
HPV-003: Cervarix vs. Al(OH)3 (f, 18-30; 12)	1	31	0	30	0.2%	2.91 [0.12, 68.66]
HPV-031: Cervarix vs. Al(OH)3 (f, 18-35; 7)	1	176	4	178	0.4%	0.25 [0.03, 2.24]
HPV-035: Cervarix vs. Al(OH)3 (f, 18-35; 7)	21	150	16	150	5.1%	1.31 [0.71, 2.41]
**Subtotal (95% CI)**		**357**		**358**	**5.7%**	**1.10 [0.46, 2.63]**
Total events	23		20			
Heterogeneity: $Tau^2 = 0.16$; $Chi^2 = 2.34$, df = 2 (P = 0.31); $I^2 = 15\%$						
Test for overall effect: Z = 0.21 (P = 0.83)						
**12.23.4 General respiratory, thoracic and mediastinal disorders ('unsolicited'): Cervarix vs. Engerix: hepatitis B vaccine (females and males, age 9-16; follow-up until 72 months)**						
HPV-030: Cervarix vs. Engerix (f, 9-15; 12)	24	247	14	246	4.7%	1.71 [0.90, 3.22]
HPV-040: Cervarix vs. Engerix (f & m, 12-16; 72)	28	1908	58	3128	9.3%	0.79 [0.51, 1.24]
**Subtotal (95% CI)**		**2155**		**3374**	**14.0%**	**1.12 [0.53, 2.38]**
Total events	52		72			
Heterogeneity: $Tau^2 = 0.22$; $Chi^2 = 3.76$, df = 1 (P = 0.05); $I^2 = 73\%$						
Test for overall effect: Z = 0.30 (P = 0.76)						
**12.23.5 General respiratory, thoracic and mediastinal disorders ('unsolicited'): Cervarix vs. Havrix: hepatitis A vaccine (females, age 9-15; follow-up until 7 months)**						
HPV-033: Cervarix vs. Havrix (f, 9-15; 7)	4	160	3	161	0.9%	1.34 [0.31, 5.90]
**Subtotal (95% CI)**		**160**		**161**	**0.9%**	**1.34 [0.31, 5.90]**
Total events	4		3			
Heterogeneity: Not applicable						
Test for overall effect: Z = 0.39 (P = 0.70)						
**12.23.6 General respiratory, thoracic and mediastinal disorders ('unsolicited'): Cervarix vs. Twinrix: hepatitis A and B vaccine (females, age 8-15; follow-up until 12 months)**						
HPV-029: Cervarix vs. Twinrix (f, 8-15; 12)	16	270	12	271	3.6%	1.34 [0.65, 2.77]
**Subtotal (95% CI)**		**270**		**271**	**3.6%**	**1.34 [0.65, 2.77]**
Total events	16		12			
Heterogeneity: Not applicable						
Test for overall effect: Z = 0.78 (P = 0.43)						
**12.23.7 General respiratory, thoracic and mediastinal disorders ('systemic adverse events'): Gardasil vs. carrier solution (females and males, age 9-16; follow-up until 18 months)**						
V501-018: Gardasil vs. carrier solu. (f&m,9-16;18)	102	1184	63	597	19.3%	0.82 [0.61, 1.10]
**Subtotal (95% CI)**		**1184**		**597**	**19.3%**	**0.82 [0.61, 1.10]**
Total events	102		63			
Heterogeneity: Not applicable						
Test for overall effect: Z = 1.33 (P = 0.18)						
**12.23.8 General respiratory, thoracic and mediastinal disorders ('systemic adverse events'): Gardasil vs. AAHS (males, age 15-27; follow-up until 36 months)**						
V501-020: Gardasil vs. AAHS (m, 15-27; 36)	70	2032	68	2033	16.3%	1.03 [0.74, 1.43]
**Subtotal (95% CI)**		**2032**		**2033**	**16.3%**	**1.03 [0.74, 1.43]**
Total events	70		68			
Heterogeneity: Not applicable						
Test for overall effect: Z = 0.18 (P = 0.86)						
**12.23.9 General respiratory, thoracic and mediastinal disorders ('systemic adverse events'): Gardasil 9 vs. saline placebo (females, age 12-26; follow-up until 7 months)**						
V503-006: Gardasil 9 vs. saline (f, 12-26;7)	60	618	24	306	9.0%	1.24 [0.79, 1.95]
**Subtotal (95% CI)**		**618**		**306**	**9.0%**	**1.24 [0.79, 1.95]**
Total events	60		24			
Heterogeneity: Not applicable						
Test for overall effect: Z = 0.92 (P = 0.36)						
**12.23.10 General respiratory, thoracic and mediastinal disorders ('systemic adverse events'): HPV 16 vaccine vs. AAHS (females, age 16-25; follow-up until 48 months)**						
V501-005: HPV 16 vaccine vs. AAHS (f, 16-25; 48)	132	1204	124	1205	29.5%	1.07 [0.85, 1.34]
**Subtotal (95% CI)**		**1204**		**1205**	**29.5%**	**1.07 [0.85, 1.34]**
Total events	132		124			
Heterogeneity: Not applicable						
Test for overall effect: Z = 0.54 (P = 0.59)						
**Total (95% CI)**		**8503**		**8757**	**100.0%**	**1.02 [0.89, 1.18]**
Total events	464		394			
Heterogeneity: $Tau^2 = 0.00$; $Chi^2 = 12.63$, df = 12 (P = 0.40); $I^2 = 5\%$						
Test for overall effect: Z = 0.30 (P = 0.76)						
Test for subgroup differences: $Chi^2 = 6.30$, df = 9 (P = 0.71), $I^2 = 0\%$						

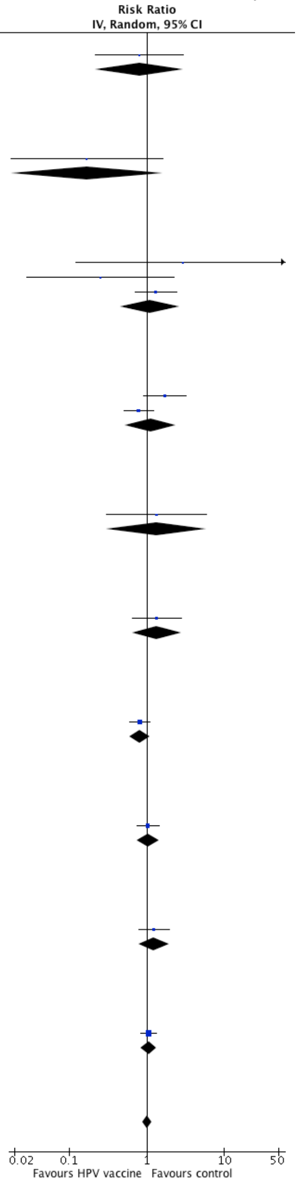

*12.23. Risk ratio for ‘solicited’ (GlaxoSmithKline): not applicable; risk ratio for ‘unsolicited’ (GlaxoSmithKline): 1.08 [0.78, 1.49]; risk ratio for ‘systemic adverse events’ (Merck Sharp & Dohme): 1.00 [0.86, 1.17].

12.24. General harms (‘solicited,’ ‘unsolicited’ and ‘systemic adverse events’*) reported within the MedDRA system organ class ‘skin and subcutaneous tissue disorders (10040785)': intention to treat analysis

Study or Subgroup	HPV vaccine Events	HPV vaccine Total	Control Events	Control Total	Weight	Risk Ratio IV, Random, 95% CI
**12.24.1 General skin and subcutaneous tissue disorders ('solicited'): Cervarix vs. Al(OH)3 (females, age 9-17; follow-up until 12 months)**						
HPV-058: Cervarix vs. Al(OH)3 (f, 9-17; 12)	17	374	7	376	3.0%	2.44 [1.02, 5.82]
**Subtotal (95% CI)**		**374**		**376**	**3.0%**	**2.44 [1.02, 5.82]**
Total events	17		7			
Heterogeneity: Not applicable						
Test for overall effect: Z = 2.01 (P = 0.04)						
**12.24.2 General skin and subcutaneous tissue disorders ('solicited'): Cervarix vs. Al(OH)3 (females, age 15-26; follow-up until 27 months)**						
HPV-001: Cervarix vs. Al(OH)3 (f, 15-26; 27)	60	560	54	553	9.0%	1.10 [0.77, 1.55]
HPV-038: Cervarix vs. Al(OH)3 (f, 15-25; 7)	41	149	10	76	4.7%	2.09 [1.11, 3.94]
**Subtotal (95% CI)**		**709**		**629**	**13.7%**	**1.43 [0.77, 2.67]**
Total events	101		64			
Heterogeneity: $Tau^2 = 0.14$; $Chi^2 = 3.06$, df = 1 (P = 0.08); $I^2 = 67\%$						
Test for overall effect: Z = 1.13 (P = 0.26)						
**12.24.3 General skin and subcutaneous tissue disorders ('solicited'): Cervarix vs. Al(OH)3 (females, age 18-35; follow-up until 12 months)**						
HPV-003: Cervarix vs. Al(OH)3 (f, 18-30; 12)	16	31	7	30	3.8%	2.21 [1.06, 4.60]
HPV-031: Cervarix vs. Al(OH)3 (f, 18-35; 7)	9	176	14	178	3.3%	0.65 [0.29, 1.46]
HPV-035: Cervarix vs. Al(OH)3 (f, 18-35; 7)	16	150	11	150	3.8%	1.45 [0.70, 3.03]
**Subtotal (95% CI)**		**357**		**358**	**11.0%**	**1.30 [0.66, 2.59]**
Total events	41		32			
Heterogeneity: $Tau^2 = 0.22$; $Chi^2 = 4.90$, df = 2 (P = 0.09); $I^2 = 59\%$						
Test for overall effect: Z = 0.76 (P = 0.45)						
**12.24.4 General skin and subcutaneous tissue disorders ('solicited'): Cervarix vs. Al(OH)3 (females, age 24-72; follow-up until 36 months)**						
HPV-015: Cervarix vs. Al(OH)3 (f, 24-72; 36)	443	2882	325	2871	13.5%	1.36 [1.19, 1.55]
**Subtotal (95% CI)**		**2882**		**2871**	**13.5%**	**1.36 [1.19, 1.55]**
Total events	443		325			
Heterogeneity: Not applicable						
Test for overall effect: Z = 4.49 (P < 0.00001)						
**12.24.5 General skin and subcutaneous tissue disorders ('solicited'): Cervarix vs. Engerix: hepatitis B vaccine (females and males, age 9-16; follow-up until 72 months)**						
HPV-030: Cervarix vs. Engerix (f, 9-15; 12)	19	247	17	246	4.8%	1.11 [0.59, 2.09]
HPV-040: Cervarix vs. Engerix (f & m, 12-16; 72)	33	604	48	1028	7.4%	1.17 [0.76, 1.80]
**Subtotal (95% CI)**		**851**		**1274**	**12.2%**	**1.15 [0.81, 1.64]**
Total events	52		65			
Heterogeneity: $Tau^2 = 0.00$; $Chi^2 = 0.02$, df = 1 (P = 0.90); $I^2 = 0\%$						
Test for overall effect: Z = 0.78 (P = 0.44)						
**12.24.6 General skin and subcutaneous tissue disorders ('solicited'): Cervarix vs. Engerix: hepatitis B vaccine (females, age 26-46; follow-up until 12 months)**						
HPV-069: Cervarix vs. Engerix (f, 26-46; 12)	6	606	2	606	1.0%	3.00 [0.61, 14.80]
**Subtotal (95% CI)**		**606**		**606**	**1.0%**	**3.00 [0.61, 14.80]**
Total events	6		2			
Heterogeneity: Not applicable						
Test for overall effect: Z = 1.35 (P = 0.18)						
**12.24.7 General skin and subcutaneous tissue disorders ('solicited'): Cervarix vs. Havrix: hepatitis A vaccine (females, age 9-15; follow-up until 12 months)**						
HPV-013: Cervarix vs. Havrix (f, 10-14; 12)	168	1035	124	1032	11.9%	1.35 [1.09, 1.68]
HPV-033: Cervarix vs. Havrix (f, 9-15; 7)	19	160	12	161	4.2%	1.59 [0.80, 3.17]
**Subtotal (95% CI)**		**1195**		**1193**	**16.1%**	**1.37 [1.12, 1.68]**
Total events	187		136			
Heterogeneity: $Tau^2 = 0.00$; $Chi^2 = 0.20$, df = 1 (P = 0.65); $I^2 = 0\%$						
Test for overall effect: Z = 3.01 (P = 0.003)						
**12.24.8 General skin and subcutaneous tissue disorders ('solicited'): Cervarix vs. Havrix: hepatitis A vaccine (females, age 15-25; follow-up until 48 months)**						
HPV-008: Cervarix vs. Havrix (f, 15-25; 48)	614	9319	502	9325	0.0%	1.22 [1.09, 1.37]
**Subtotal (95% CI)**		**0**		**0**		**Not estimable**
Total events	0		0			
Heterogeneity: Not applicable						
Test for overall effect: Not applicable						
**12.24.9 General skin and subcutaneous tissue disorders ('solicited'): Cervarix vs. Twinrix: hepatitis A and B vaccine (females, age 8-15; follow-up until 12 months)**						
HPV-029: Cervarix vs. Twinrix (f, 8-15; 12)	32	270	21	271	6.0%	1.53 [0.91, 2.58]
**Subtotal (95% CI)**		**270**		**271**	**6.0%**	**1.53 [0.91, 2.58]**
Total events	32		21			
Heterogeneity: Not applicable						
Test for overall effect: Z = 1.59 (P = 0.11)						
**12.24.10 General skin and subcutaneous tissue disorders ('unsolicited'): Cervarix vs. Al(OH)3 (females, age 9-17; follow-up until 12 months)**						
HPV-058: Cervarix vs. Al(OH)3 (f, 9-17; 12)	1	374	1	376	0.0%	1.01 [0.06, 16.01]
**Subtotal (95% CI)**		**0**		**0**		**Not estimable**
Total events	0		0			
Heterogeneity: Not applicable						
Test for overall effect: Not applicable						
**12.24.11 General skin and subcutaneous tissue disorders ('unsolicited'): Cervarix vs. Al(OH)3 (females, age 15-26; follow-up until 27 months)**						
HPV-001: Cervarix vs. Al(OH)3 (f, 15-26; 27)	169	560	151	553	0.0%	1.11 [0.92, 1.33]
HPV-038: Cervarix vs. Al(OH)3 (f, 15-25; 7)	11	149	3	76	0.0%	1.87 [0.54, 6.50]
**Subtotal (95% CI)**		**0**		**0**		**Not estimable**
Total events	0		0			
Heterogeneity: Not applicable						
Test for overall effect: Not applicable						
**12.24.12 General skin and subcutaneous tissue disorders ('unsolicited'): Cervarix vs. Al(OH)3 (females, age 18-35; follow-up until 12 months)**						
HPV-003: Cervarix vs. Al(OH)3 (f, 18-30; 12)	15	31	8	30	0.0%	1.81 [0.90, 3.64]
HPV-031: Cervarix vs. Al(OH)3 (f, 18-35; 7)	0	176	4	178	0.0%	0.11 [0.01, 2.07]
HPV-035: Cervarix vs. Al(OH)3 (f, 18-35; 7)	15	150	5	150	0.0%	3.00 [1.12, 8.05]
**Subtotal (95% CI)**		**0**		**0**		**Not estimable**
Total events	0		0			
Heterogeneity: Not applicable						
Test for overall effect: Not applicable						
**12.24.13 General skin and subcutaneous tissue disorders ('unsolicited'): Cervarix vs. Engerix: hepatitis B vaccine (females and males, age 9-16; follow-up until 72 months)**						
HPV-030: Cervarix vs. Engerix (f, 9-15; 12)	5	247	7	246	0.0%	0.71 [0.23, 2.21]
HPV-040: Cervarix vs. Engerix (f & m, 12-16; 72)	7	1908	9	3128	0.0%	1.28 [0.48, 3.42]
**Subtotal (95% CI)**		**0**		**0**		**Not estimable**
Total events	0		0			
Heterogeneity: Not applicable						
Test for overall effect: Not applicable						
**12.24.14 General skin and subcutaneous tissue disorders ('unsolicited'): Cervarix vs. Engerix: hepatitis B vaccine (females, age 26-46; follow-up until 12 months)**						
HPV-069: Cervarix vs. Engerix (f, 26-46; 12)	0	606	1	606	0.0%	0.33 [0.01, 8.17]
**Subtotal (95% CI)**		**0**		**0**		**Not estimable**
Total events	0		0			
Heterogeneity: Not applicable						
Test for overall effect: Not applicable						
**12.24.15 General skin and subcutaneous tissue disorders ('unsolicited'): Cervarix vs. Havrix: hepatitis A vaccine (females, age 9-15; follow-up until 7 months)**						
HPV-033: Cervarix vs. Havrix (f, 9-15; 7)	5	160	1	161	0.0%	5.03 [0.59, 42.59]
**Subtotal (95% CI)**		**0**		**0**		**Not estimable**
Total events	0		0			
Heterogeneity: Not applicable						
Test for overall effect: Not applicable						
**12.24.16 General skin and subcutaneous tissue disorders ('unsolicited'): Cervarix vs. Twinrix: hepatitis A and B vaccine (females, age 8-15; follow-up until 12 months)**						
HPV-029: Cervarix vs. Twinrix (f, 8-15; 12)	10	270	7	271	0.0%	1.43 [0.55, 3.71]
**Subtotal (95% CI)**		**0**		**0**		**Not estimable**
Total events	0		0			
Heterogeneity: Not applicable						
Test for overall effect: Not applicable						
**12.24.17 General skin and subcutaneous tissue disorders ('systemic adverse events'): Gardasil vs. carrier solution (females and males, age 9-16; follow-up until 18 months)**						
V501-018: Gardasil vs. carrier solu. (f&m,9-16;18)	25	1184	21	597	5.4%	0.60 [0.34, 1.06]
**Subtotal (95% CI)**		**1184**		**597**	**5.4%**	**0.60 [0.34, 1.06]**
Total events	25		21			
Heterogeneity: Not applicable						
Test for overall effect: Z = 1.75 (P = 0.08)						
**12.24.18 General skin and subcutaneous tissue disorders ('systemic adverse events'): Gardasil vs. AAHS (males, age 15-27; follow-up until 36 months)**						
V501-020: Gardasil vs. AAHS (m, 15-27; 36)	26	2032	31	2033	6.1%	0.84 [0.50, 1.41]
**Subtotal (95% CI)**		**2032**		**2033**	**6.1%**	**0.84 [0.50, 1.41]**
Total events	26		31			
Heterogeneity: Not applicable						
Test for overall effect: Z = 0.66 (P = 0.51)						
**12.24.19 General skin and subcutaneous tissue disorders ('systemic adverse events'): Gardasil 9 vs. saline placebo (females, age 12-26; follow-up until 7 months)**						
V503-006: Gardasil 9 vs. saline (f, 12-26;7)	30	618	10	306	4.1%	1.49 [0.74, 3.00]
**Subtotal (95% CI)**		**618**		**306**	**4.1%**	**1.49 [0.74, 3.00]**
Total events	30		10			
Heterogeneity: Not applicable						
Test for overall effect: Z = 1.10 (P = 0.27)						
**12.24.20 General skin and subcutaneous tissue disorders ('systemic adverse events'): HPV 16 vaccine vs. AAHS (females, age 16-25; follow-up until 48 months)**						
V501-005: HPV 16 vaccine vs. AAHS (f, 16-25; 48)	37	1204	57	1205	7.9%	0.65 [0.43, 0.98]
**Subtotal (95% CI)**		**1204**		**1205**	**7.9%**	**0.65 [0.43, 0.98]**
Total events	37		57			
Heterogeneity: Not applicable						
Test for overall effect: Z = 2.08 (P = 0.04)						
**Total (95% CI)**		**12282**		**11719**	**100.0%**	**1.21 [1.03, 1.44]**
Total events	997		771			
Heterogeneity: $Tau^2 = 0.05$; $Chi^2 = 33.50$, df = 16 (P = 0.006); $I^2 = 52\%$						
Test for overall effect: Z = 2.28 (P = 0.02)						
Test for subgroup differences: $Chi^2 = 25.42$, df = 11 (P = 0.008), $I^2 = 56.7\%$						

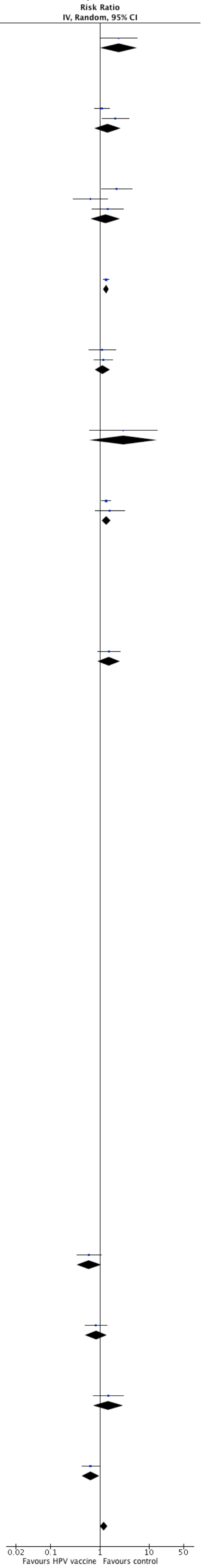

*12.24. Risk ratio for ‘solicited’ (GlaxoSmithKline): **1.31 [1.20, 1.42];** risk ratio for ‘unsolicited’ (GlaxoSmithKline): 1.31 [0.98, 1.74]; risk ratio for ‘systemic adverse events’ (Merck Sharp & Dohme): 0.79 [0.56, 1.10]. To avoid double counting of participants in the total risk ratio estimate, we excluded the ‘unsolicited’ adverse events from total risk ratio estimate for studies that reported ‘solicited’ adverse events.

12.25. General harms (‘solicited,’ ‘unsolicited’ and ‘systemic adverse events’*) reported within the MedDRA system organ class ‘social circumstances (10041244)': intention to treat analysis

Study or Subgroup	HPV vaccine Events	HPV vaccine Total	Control Events	Control Total	Weight	Risk Ratio IV, Random, 95% CI
**12.25.1 General social circumstances ('unsolicited'): Cervarix vs. Twinrix: hepatitis A and B vaccine (females, age 8-15; follow-up until 12 months)**						
HPV-029: Cervarix vs. Twinrix (f, 8-15; 12)	0	270	1	271	50.1%	0.33 [0.01, 8.18]
**Subtotal (95% CI)**		**270**		**271**	**50.1%**	**0.33 [0.01, 8.18]**
Total events	0		1			
Heterogeneity: Not applicable						
Test for overall effect: Z = 0.67 (P = 0.50)						
**12.25.2 General social circumstances ('systemic adverse events'): Gardasil vs. AAHS (males, age 15-27; follow-up until 36 months)**						
V501-020: Gardasil vs. AAHS (m, 15-27; 36)	1	2032	0	2033	49.9%	3.00 [0.12, 73.64]
**Subtotal (95% CI)**		**2032**		**2033**	**49.9%**	**3.00 [0.12, 73.64]**
Total events	1		0			
Heterogeneity: Not applicable						
Test for overall effect: Z = 0.67 (P = 0.50)						
**Total (95% CI)**		**2302**		**2304**	**100.0%**	**1.00 [0.10, 9.60]**
Total events	1		1			
Heterogeneity: Tau² = 0.00; Chi² = 0.90, df = 1 (P = 0.34); I² = 0%						
Test for overall effect: Z = 0.00 (P = 1.00)						
Test for subgroup differences: Chi² = 0.90, df = 1 (P = 0.34), I² = 0%						

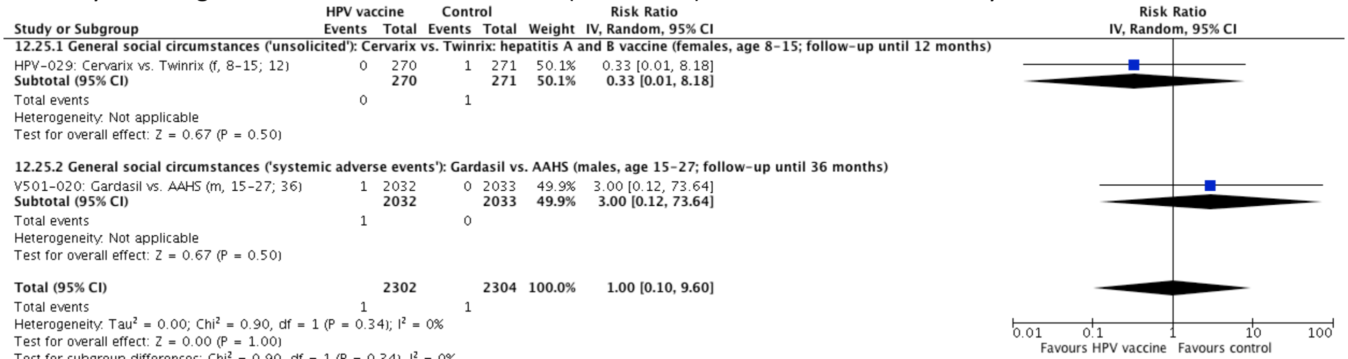

*12.25. Risk ratio for ‘solicited’ (GlaxoSmithKline): not applicable; risk ratio for ‘unsolicited’ (GlaxoSmithKline): 0.33 [0.01, 8.18]; risk ratio for ‘systemic adverse events’ (Merck Sharp & Dohme): 3.00 [0.12, 73.64].

12.26. General harms (‘solicited,’ ‘unsolicited’ and ‘systemic adverse events’) reported within the MedDRA system organ class ‘surgical and medical procedures (10042613)': intention to treat analysis

No cases/data/reports.

12.27. General harms (‘solicited,’ ‘unsolicited’ and ‘systemic adverse events’*) reported within the MedDRA system organ class ‘vascular disorders (10047065)': intention to treat analysis

Study or Subgroup	HPV vaccine Events	HPV vaccine Total	Control Events	Control Total	Weight	Risk Ratio IV, Random, 95% CI
**12.27.1 General vascular disorders ('unsolicited'): Cervarix vs. Al(OH)3 (females, age 9-17; follow-up until 12 months)**						
HPV-058: Cervarix vs. Al(OH)3 (f, 9-17; 12)	0	374	1	376	7.6%	0.34 [0.01, 8.20]
**Subtotal (95% CI)**		**374**		**376**	**7.6%**	**0.34 [0.01, 8.20]**
Total events	0		1			
Heterogeneity: Not applicable						
Test for overall effect: Z = 0.67 (P = 0.50)						
**12.27.2 General vascular disorders ('unsolicited'): Cervarix vs. Engerix: hepatitis B vaccine (females and males, age 9-16; follow-up until 72 months)**						
HPV-030: Cervarix vs. Engerix (f, 9-15; 12)	0	247	2	246	8.4%	0.20 [0.01, 4.13]
HPV-040: Cervarix vs. Engerix (f & m, 12-16; 72)	1	1908	0	3128	7.5%	4.92 [0.20, 120.64]
**Subtotal (95% CI)**		**2155**		**3374**	**15.9%**	**0.95 [0.04, 21.93]**
Total events	1		2			
Heterogeneity: $Tau^2 = 2.61$; $Chi^2 = 2.03$, df = 1 (P = 0.15); $I^2 = 51\%$						
Test for overall effect: Z = 0.03 (P = 0.97)						
**12.27.3 General vascular disorders ('unsolicited'): Cervarix vs. Havrix: hepatitis A vaccine (females, age 9-15; follow-up until 12 months)**						
HPV-033: Cervarix vs. Havrix (f, 9-15; 7)	0	160	1	161	7.6%	0.34 [0.01, 8.17]
**Subtotal (95% CI)**		**160**		**161**	**7.6%**	**0.34 [0.01, 8.17]**
Total events	0		1			
Heterogeneity: Not applicable						
Test for overall effect: Z = 0.67 (P = 0.50)						
**12.27.4 General vascular disorders ('unsolicited'): Cervarix vs. Twinrix: hepatitis A and B vaccine (females, age 8-15; follow-up until 12 months)**						
HPV-029: Cervarix vs. Twinrix (f, 8-15; 12)	1	270	1	271	10.1%	1.00 [0.06, 15.96]
**Subtotal (95% CI)**		**270**		**271**	**10.1%**	**1.00 [0.06, 15.96]**
Total events	1		1			
Heterogeneity: Not applicable						
Test for overall effect: Z = 0.00 (P = 1.00)						
**12.27.5 General vascular disorders ('systemic adverse events'): Gardasil vs. carrier solution (females and males, age 9-16; follow-up until 18 months)**						
V501-018: Gardasil vs. carrier solu. (f&m,9-16;18)	1	1184	3	597	15.1%	0.17 [0.02, 1.61]
**Subtotal (95% CI)**		**1184**		**597**	**15.1%**	**0.17 [0.02, 1.61]**
Total events	1		3			
Heterogeneity: Not applicable						
Test for overall effect: Z = 1.55 (P = 0.12)						
**12.27.6 General vascular disorders ('systemic adverse events'): Gardasil vs. AAHS (males, age 15-27; follow-up until 36 months)**						
V501-020: Gardasil vs. AAHS (m, 15-27; 36)	3	2032	4	2033	34.5%	0.75 [0.17, 3.35]
**Subtotal (95% CI)**		**2032**		**2033**	**34.5%**	**0.75 [0.17, 3.35]**
Total events	3		4			
Heterogeneity: Not applicable						
Test for overall effect: Z = 0.38 (P = 0.71)						
**12.27.7 General vascular disorders ('systemic adverse events'): Gardasil 9 vs. saline placebo (females, age 12-26; follow-up until 7 months)**						
V503-006: Gardasil 9 vs. saline (f, 12-26;7)	5	618	0	306	9.2%	5.46 [0.30, 98.34]
**Subtotal (95% CI)**		**618**		**306**	**9.2%**	**5.46 [0.30, 98.34]**
Total events	5		0			
Heterogeneity: Not applicable						
Test for overall effect: Z = 1.15 (P = 0.25)						
**Total (95% CI)**		**6793**		**7118**	**100.0%**	**0.68 [0.28, 1.63]**
Total events	11		12			
Heterogeneity: $Tau^2 = 0.00$; $Chi^2 = 6.03$, df = 7 (P = 0.54); $I^2 = 0\%$						
Test for overall effect: Z = 0.87 (P = 0.38)						
Test for subgroup differences: $Chi^2 = 3.97$, df = 6 (P = 0.68), $I^2 = 0\%$						

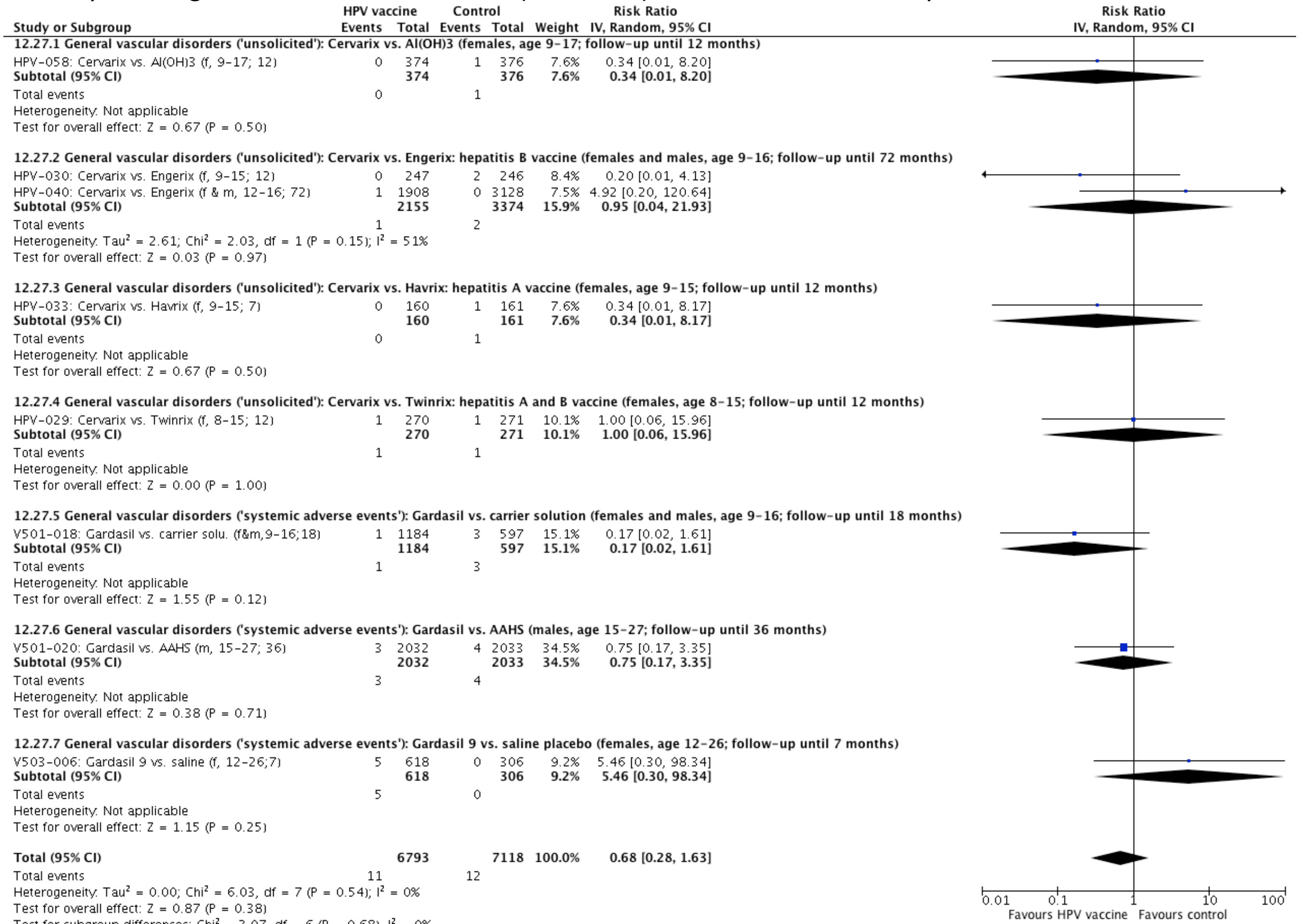

*12.27. Risk ratio for ‘solicited’ (GlaxoSmithKline): not applicable; risk ratio for ‘unsolicited’ (GlaxoSmithKline): 0.65 [0.16, 2.54]; risk ratio for ‘systemic adverse events’ (Merck Sharp & Dohme): 0.74 [0.15, 3.74].

12.28. Most common general harms (‘solicited’ and ‘unsolicited’*) - ‘fatigue’: intention to treat analysis

Study or Subgroup	HPV vaccine Events	HPV vaccine Total	Control Events	Control Total	Weight	Risk Ratio IV, Random, 95% CI
**12.28.1 General fatigue ('solicited'): Cervarix vs. Al(OH)3 (females, age 9-17; follow-up until 12 months)**						
HPV-058: Cervarix vs. Al(OH)3 (f, 9-17; 12)	137	374	126	376	4.3%	1.09 [0.90, 1.33]
**Subtotal (95% CI)**		**374**		**376**	**4.3%**	**1.09 [0.90, 1.33]**
Total events	137		126			
Heterogeneity: Not applicable						
Test for overall effect: Z = 0.89 (P = 0.37)						
**12.28.2 General fatigue ('solicited'): Cervarix vs. Al(OH)3 (females, age 15-26; follow-up until 27 months)**						
HPV-001: Cervarix vs. Al(OH)3 (f, 15-26; 27)	308	560	289	553	10.5%	1.05 [0.94, 1.17]
HPV-038: Cervarix vs. Al(OH)3 (f, 15-25; 7)	103	149	39	76	2.9%	1.35 [1.06, 1.72]
**Subtotal (95% CI)**		**709**		**629**	**13.4%**	**1.16 [0.92, 1.47]**
Total events	411		328			
Heterogeneity: $Tau^2 = 0.02$; $Chi^2 = 3.28$, df = 1 (P = 0.07); $I^2 = 69\%$						
Test for overall effect: Z = 1.24 (P = 0.22)						
**12.28.3 General fatigue ('solicited'): Cervarix vs. Al(OH)3 (females, age 18-35; follow-up until 12 months)**						
HPV-003: Cervarix vs. Al(OH)3 (f, 18-30; 12)	20	31	20	30	1.4%	0.97 [0.67, 1.39]
HPV-031: Cervarix vs. Al(OH)3 (f, 18-35; 7)	84	176	83	178	3.5%	1.02 [0.82, 1.28]
HPV-035: Cervarix vs. Al(OH)3 (f, 18-35; 7)	94	300	75	300	2.6%	1.25 [0.97, 1.62]
**Subtotal (95% CI)**		**507**		**508**	**7.5%**	**1.09 [0.93, 1.27]**
Total events	198		178			
Heterogeneity: $Tau^2 = 0.00$; $Chi^2 = 1.85$, df = 2 (P = 0.40); $I^2 = 0\%$						
Test for overall effect: Z = 1.08 (P = 0.28)						
**12.28.4 General fatigue ('solicited'): Cervarix vs. Al(OH)3 (females, age 24-72; follow-up until 36 months)**						
HPV-015: Cervarix vs. Al(OH)3 (f, 24-72; 36)	1116	2882	916	2871	17.1%	1.21 [1.13, 1.30]
**Subtotal (95% CI)**		**2882**		**2871**	**17.1%**	**1.21 [1.13, 1.30]**
Total events	1116		916			
Heterogeneity: Not applicable						
Test for overall effect: Z = 5.39 (P < 0.00001)						
**12.28.5 General fatigue ('solicited'): Cervarix vs. Engerix: hepatitis B vaccine (females and males, age 9-16; follow-up until 72 months)**						
HPV-030: Cervarix vs. Engerix (f, 9-15; 12)	107	247	104	246	4.0%	1.02 [0.84, 1.26]
HPV-040: Cervarix vs. Engerix (f & m, 12-16; 72)	291	604	411	1028	10.2%	1.21 [1.08, 1.35]
**Subtotal (95% CI)**		**851**		**1274**	**14.2%**	**1.14 [0.98, 1.32]**
Total events	398		515			
Heterogeneity: $Tau^2 = 0.01$; $Chi^2 = 1.87$, df = 1 (P = 0.17); $I^2 = 46\%$						
Test for overall effect: Z = 1.66 (P = 0.10)						
**12.28.6 General fatigue ('solicited'): Cervarix vs. Engerix: hepatitis B vaccine (females, age 26-46; follow-up until 12 months)**						
HPV-069: Cervarix vs. Engerix (f, 26-46; 12)	100	606	68	606	2.2%	1.47 [1.10, 1.96]
**Subtotal (95% CI)**		**606**		**606**	**2.2%**	**1.47 [1.10, 1.96]**
Total events	100		68			
Heterogeneity: Not applicable						
Test for overall effect: Z = 2.64 (P = 0.008)						
**12.28.7 General fatigue ('solicited'): Cervarix vs. Havrix: hepatitis A vaccine (females, age 9-15; follow-up until 12 months)**						
HPV-013: Cervarix vs. Havrix (f, 10-14; 12)	499	1035	434	1032	12.5%	1.15 [1.04, 1.26]
HPV-033: Cervarix vs. Havrix (f, 9-15; 7)	59	160	52	161	2.0%	1.14 [0.84, 1.54]
**Subtotal (95% CI)**		**1195**		**1193**	**14.4%**	**1.15 [1.05, 1.26]**
Total events	558		486			
Heterogeneity: $Tau^2 = 0.00$; $Chi^2 = 0.00$, df = 1 (P = 0.98); $I^2 = 0\%$						
Test for overall effect: Z = 2.93 (P = 0.003)						
**12.28.8 General fatigue ('solicited'): Cervarix vs. Havrix: hepatitis A vaccine (females, age 15-25; follow-up until 48 months)**						
HPV-008: Cervarix vs. Havrix (f, 15-25; 48)	1771	9319	1652	9325	19.4%	1.07 [1.01, 1.14]
**Subtotal (95% CI)**		**9319**		**9325**	**19.4%**	**1.07 [1.01, 1.14]**
Total events	1771		1652			
Heterogeneity: Not applicable						
Test for overall effect: Z = 2.27 (P = 0.02)						
**12.28.9 General fatigue ('solicited'): Cervarix vs. Twinrix: hepatitis A and B vaccine (females, age 8-15; follow-up until 12 months)**						
HPV-029: Cervarix vs. Twinrix (f, 8-15; 12)	133	270	119	271	4.9%	1.12 [0.94, 1.34]
**Subtotal (95% CI)**		**270**		**271**	**4.9%**	**1.12 [0.94, 1.34]**
Total events	133		119			
Heterogeneity: Not applicable						
Test for overall effect: Z = 1.24 (P = 0.21)						
**12.28.10 General fatigue ('unsolicited'): Cervarix vs. Engerix: hepatitis B vaccine (females and males, age 9-16; follow-up until 72 months)**						
HPV-030: Cervarix vs. Engerix (f, 9-15; 12)	2	247	1	246	0.0%	1.99 [0.18, 21.82]
HPV-040: Cervarix vs. Engerix (f & m, 12-16; 72)	0	1908	2	3128	0.0%	0.33 [0.02, 6.82]
**Subtotal (95% CI)**		**0**		**0**		**Not estimable**
Total events	0		0			
Heterogeneity: Not applicable						
Test for overall effect: Not applicable						
**12.28.11 General fatigue ('systemic adverse events'): Gardasil vs. carrier solution (females and males, age 9-16; follow-up until 18 months)**						
V501-018: Gardasil vs. carrier solu. (f&m,9-16;18)	18	1184	7	597	0.3%	1.30 [0.54, 3.09]
**Subtotal (95% CI)**		**1184**		**597**	**0.3%**	**1.30 [0.54, 3.09]**
Total events	18		7			
Heterogeneity: Not applicable						
Test for overall effect: Z = 0.59 (P = 0.56)						
**12.28.12 General fatigue ('systemic adverse events'): Gardasil vs. AAHS (males, age 15-27; follow-up until 36 months)**						
V501-020: Gardasil vs. AAHS (m, 15-27; 36)	13	2032	19	2033	0.4%	0.68 [0.34, 1.38]
**Subtotal (95% CI)**		**2032**		**2033**	**0.4%**	**0.68 [0.34, 1.38]**
Total events	13		19			
Heterogeneity: Not applicable						
Test for overall effect: Z = 1.06 (P = 0.29)						
**12.28.13 General fatigue ('systemic adverse events'): Gardasil 9 vs. saline placebo (females, age 12-26; follow-up until 7 months)**						
V503-006: Gardasil 9 vs. saline (f, 12-26;7)	21	618	11	306	0.4%	0.95 [0.46, 1.94]
**Subtotal (95% CI)**		**618**		**306**	**0.4%**	**0.95 [0.46, 1.94]**
Total events	21		11			
Heterogeneity: Not applicable						
Test for overall effect: Z = 0.15 (P = 0.88)						
**12.28.14 General fatigue ('systemic adverse events'): HPV 16 vaccine vs. AAHS (females, age 16-25; follow-up until 48 months)**						
V501-005: HPV 16 vaccine vs. AAHS (f, 16-25; 48)	59	1204	64	1205	1.5%	0.92 [0.65, 1.30]
**Subtotal (95% CI)**		**1204**		**1205**	**1.5%**	**0.92 [0.65, 1.30]**
Total events	59		64			
Heterogeneity: Not applicable						
Test for overall effect: Z = 0.46 (P = 0.65)						
**Total (95% CI)**		**21751**		**21194**	**100.0%**	**1.13 [1.08, 1.18]**
Total events	4933		4489			
Heterogeneity: $Tau^2 = 0.00$; $Chi^2 = 21.72$, df = 17 (P = 0.20); $I^2 = 22\%$						
Test for overall effect: Z = 5.50 (P < 0.00001)						
Test for subgroup differences: $Chi^2 = 14.15$, df = 12 (P = 0.29), $I^2 = 15.2\%$						

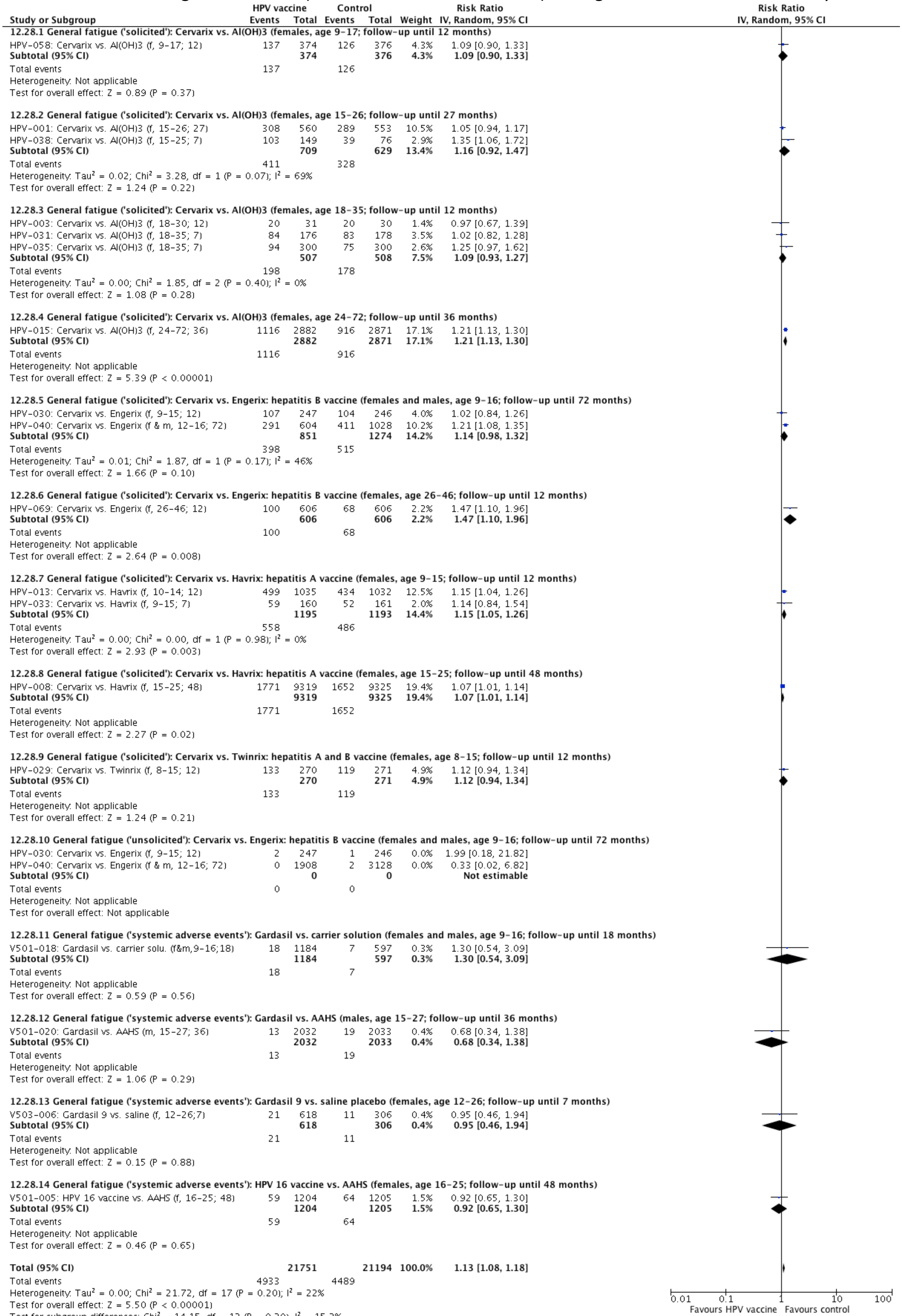

*12.28. Risk ratio for ‘solicited’ (GlaxoSmithKline): **1.14 [1.09, 1.19]**; risk ratio for ‘unsolicited’ (GlaxoSmithKline): 1.00 [0.15, 6.53]; risk ratio for ‘systemic adverse events’ (Merck Sharp & Dohme): 0.92 [0.70, 1.20]. To avoid double counting of participants in the total risk ratio estimate, we excluded the ‘unsolicited’ adverse events from total risk ratio estimate for studies that reported ‘solicited’ adverse events.

12.29. Most common general harms (‘solicited’ and ‘unsolicited’*) - ‘headache’: intention to treat analysis

Study or Subgroup	HPV vaccine Events	HPV vaccine Total	Control Events	Control Total	Weight	Risk Ratio IV, Random, 95% CI
**12.29.1 General headache ('solicited'): Cervarix vs. Al(OH)3 (females, age 9-17; follow-up until 12 months)**						
HPV-058: Cervarix vs. Al(OH)3 (f, 9-17; 12)	123	374	99	376	3.5%	1.25 [1.00, 1.56]
**Subtotal (95% CI)**		**374**		**376**	**3.5%**	**1.25 [1.00, 1.56]**
Total events	123		99			
Heterogeneity: Not applicable						
Test for overall effect: Z = 1.96 (P = 0.05)						
**12.29.2 General headache ('solicited'): Cervarix vs. Al(OH)3 (females, age 15-26; follow-up until 27 months)**						
HPV-001: Cervarix vs. Al(OH)3 (f, 15-26; 27)	331	560	329	553	10.2%	0.99 [0.90, 1.10]
HPV-038: Cervarix vs. Al(OH)3 (f, 15-25; 7)	73	149	30	76	1.8%	1.24 [0.90, 1.71]
**Subtotal (95% CI)**		**709**		**629**	**12.0%**	**1.05 [0.87, 1.27]**
Total events	404		359			
Heterogeneity: $Tau^2 = 0.01$; $Chi^2 = 1.67$, df = 1 (P = 0.20); $I^2 = 40\%$						
Test for overall effect: Z = 0.51 (P = 0.61)						
**12.29.3 General headache ('solicited'): Cervarix vs. Al(OH)3 (females, age 18-35; follow-up until 12 months)**						
HPV-003: Cervarix vs. Al(OH)3 (f, 18-30; 12)	16	31	21	30	1.2%	0.74 [0.49, 1.12]
HPV-031: Cervarix vs. Al(OH)3 (f, 18-35; 7)	72	176	71	178	2.8%	1.03 [0.80, 1.32]
HPV-035: Cervarix vs. Al(OH)3 (f, 18-35; 7)	67	150	54	150	2.4%	1.24 [0.94, 1.64]
**Subtotal (95% CI)**		**357**		**358**	**6.4%**	**1.02 [0.79, 1.31]**
Total events	155		146			
Heterogeneity: $Tau^2 = 0.03$; $Chi^2 = 4.22$, df = 2 (P = 0.12); $I^2 = 53\%$						
Test for overall effect: Z = 0.12 (P = 0.91)						
**12.29.4 General headache ('solicited'): Cervarix vs. Al(OH)3 (females, age 24-72; follow-up until 36 months)**						
HPV-015: Cervarix vs. Al(OH)3 (f, 24-72; 36)	1165	2882	1074	2871	13.6%	1.08 [1.01, 1.15]
**Subtotal (95% CI)**		**2882**		**2871**	**13.6%**	**1.08 [1.01, 1.15]**
Total events	1165		1074			
Heterogeneity: Not applicable						
Test for overall effect: Z = 2.34 (P = 0.02)						
**12.29.5 General headache ('solicited'): Cervarix vs. Engerix: hepatitis B vaccine (females and males, age 9-16; follow-up until 72 months)**						
HPV-030: Cervarix vs. Engerix (f, 9-15; 12)	131	247	129	246	5.4%	1.01 [0.86, 1.20]
HPV-040: Cervarix vs. Engerix (f & m, 12-16; 72)	261	604	371	1028	8.1%	1.20 [1.06, 1.35]
**Subtotal (95% CI)**		**851**		**1274**	**13.5%**	**1.11 [0.94, 1.31]**
Total events	392		500			
Heterogeneity: $Tau^2 = 0.01$; $Chi^2 = 2.55$, df = 1 (P = 0.11); $I^2 = 61\%$						
Test for overall effect: Z = 1.26 (P = 0.21)						
**12.29.6 General headache ('solicited'): Cervarix vs. Engerix: hepatitis B vaccine (females, age 26-46; follow-up until 12 months)**						
HPV-069: Cervarix vs. Engerix (f, 26-46; 12)	71	606	46	606	1.6%	1.54 [1.08, 2.20]
**Subtotal (95% CI)**		**606**		**606**	**1.6%**	**1.54 [1.08, 2.20]**
Total events	71		46			
Heterogeneity: Not applicable						
Test for overall effect: Z = 2.41 (P = 0.02)						
**12.29.7 General headache ('solicited'): Cervarix vs. Havrix: hepatitis A vaccine (females, age 9-15; follow-up until 12 months)**						
HPV-013: Cervarix vs. Havrix (f, 10-14; 12)	516	1035	464	1032	10.8%	1.11 [1.01, 1.21]
HPV-033: Cervarix vs. Havrix (f, 9-15; 7)	44	160	53	161	1.7%	0.84 [0.60, 1.17]
**Subtotal (95% CI)**		**1195**		**1193**	**12.5%**	**1.01 [0.78, 1.31]**
Total events	560		517			
Heterogeneity: $Tau^2 = 0.02$; $Chi^2 = 2.56$, df = 1 (P = 0.11); $I^2 = 61\%$						
Test for overall effect: Z = 0.07 (P = 0.94)						
**12.29.8 General headache ('solicited'): Cervarix vs. Havrix: hepatitis A vaccine (females, age 15-25; follow-up until 48 months)**						
HPV-008: Cervarix vs. Havrix (f, 15-25; 48)	1668	9319	1583	9325	13.9%	1.05 [0.99, 1.12]
**Subtotal (95% CI)**		**9319**		**9325**	**13.9%**	**1.05 [0.99, 1.12]**
Total events	1668		1583			
Heterogeneity: Not applicable						
Test for overall effect: Z = 1.66 (P = 0.10)						
**12.29.9 General headache ('solicited'): Cervarix vs. Twinrix: hepatitis A and B vaccine (females, age 8-15; follow-up until 12 months)**						
HPV-029: Cervarix vs. Twinrix (f, 8-15; 12)	124	270	110	271	4.3%	1.13 [0.93, 1.37]
**Subtotal (95% CI)**		**270**		**271**	**4.3%**	**1.13 [0.93, 1.37]**
Total events	124		110			
Heterogeneity: Not applicable						
Test for overall effect: Z = 1.25 (P = 0.21)						
**12.29.10 General headache ('unsolicited'): Cervarix vs. Al(OH)3 (females, age 9-17; follow-up until 12 months)**						
HPV-058: Cervarix vs. Al(OH)3 (f, 9-17; 12)	1	374	0	376	0.0%	3.02 [0.12, 73.80]
**Subtotal (95% CI)**		**0**		**0**		**Not estimable**
Total events	0		0			
Heterogeneity: Not applicable						
Test for overall effect: Not applicable						
**12.29.11 General headache ('unsolicited'): Cervarix vs. Al(OH)3 (females, age 15-25; follow-up until 7 months)**						
HPV-038: Cervarix vs. Al(OH)3 (f, 15-25; 7)	10	149	5	76	0.0%	1.02 [0.36, 2.88]
**Subtotal (95% CI)**		**0**		**0**		**Not estimable**
Total events	0		0			
Heterogeneity: Not applicable						
Test for overall effect: Not applicable						
**12.29.12 General headache ('unsolicited'): Cervarix vs. Al(OH)3 (females, age 18-35; follow-up until 7 months)**						
HPV-031: Cervarix vs. Al(OH)3 (f, 18-35; 7)	4	176	1	178	0.0%	4.05 [0.46, 35.84]
HPV-035: Cervarix vs. Al(OH)3 (f, 18-35; 7)	8	150	8	150	0.0%	1.00 [0.39, 2.59]
**Subtotal (95% CI)**		**0**		**0**		**Not estimable**
Total events	0		0			
Heterogeneity: Not applicable						
Test for overall effect: Not applicable						
**12.29.13 General headache ('unsolicited'): Cervarix vs. Engerix: hepatitis B vaccine (females and males, age 9-16; follow-up until 72 months)**						
HPV-030: Cervarix vs. Engerix (f, 9-15; 12)	24	247	11	246	0.0%	2.17 [1.09, 4.34]
HPV-040: Cervarix vs. Engerix (f & m, 12-16; 72)	26	1908	20	3128	0.0%	2.13 [1.19, 3.81]
**Subtotal (95% CI)**		**0**		**0**		**Not estimable**
Total events	0		0			
Heterogeneity: Not applicable						
Test for overall effect: Not applicable						
**12.29.14 General headache ('unsolicited'): Cervarix vs. Twinrix: hepatitis A and B vaccine (females, age 8-15; follow-up until 12 months)**						
HPV-029: Cervarix vs. Twinrix (f, 8-15; 12)	13	270	8	271	0.0%	1.63 [0.69, 3.87]
**Subtotal (95% CI)**		**0**		**0**		**Not estimable**
Total events	0		0			
Heterogeneity: Not applicable						
Test for overall effect: Not applicable						
**12.29.15 General headache ('systemic adverse events'): Gardasil vs. carrier solution (females and males, age 9-16; follow-up until 18 months)**						
V501-018: Gardasil vs. carrier solu. (f&m,9-16;18)	221	1184	110	597	3.9%	1.01 [0.82, 1.25]
**Subtotal (95% CI)**		**1184**		**597**	**3.9%**	**1.01 [0.82, 1.25]**
Total events	221		110			
Heterogeneity: Not applicable						
Test for overall effect: Z = 0.12 (P = 0.90)						
**12.29.16 General headache ('systemic adverse events'): Gardasil vs. AAHS (males, age 15-27; follow-up until 36 months)**						
V501-020: Gardasil vs. AAHS (m, 15-27; 36)	179	2032	207	2033	4.5%	0.87 [0.72, 1.05]
**Subtotal (95% CI)**		**2032**		**2033**	**4.5%**	**0.87 [0.72, 1.05]**
Total events	179		207			
Heterogeneity: Not applicable						
Test for overall effect: Z = 1.49 (P = 0.14)						
**12.29.17 General headache ('systemic adverse events'): HPV 16 vaccine vs. AAHS (females, age 16-25; follow-up until 48 months)**						
V501-005: HPV 16 vaccine vs. AAHS (f, 16-25; 48)	499	1204	495	1205	10.4%	1.01 [0.92, 1.11]
**Subtotal (95% CI)**		**1204**		**1205**	**10.4%**	**1.01 [0.92, 1.11]**
Total events	499		495			
Heterogeneity: Not applicable						
Test for overall effect: Z = 0.18 (P = 0.86)						
**Total (95% CI)**		**20983**		**20738**	**100.0%**	**1.06 [1.02, 1.11]**
Total events	5561		5246			
Heterogeneity: $Tau^2 = 0.00$; $Chi^2 = 26.66$, df = 16 (P = 0.05); $I^2 = 40\%$						
Test for overall effect: Z = 2.62 (P = 0.009)						
Test for subgroup differences: $Chi^2 = 13.39$, df = 11 (P = 0.27), $I^2 = 17.9\%$						

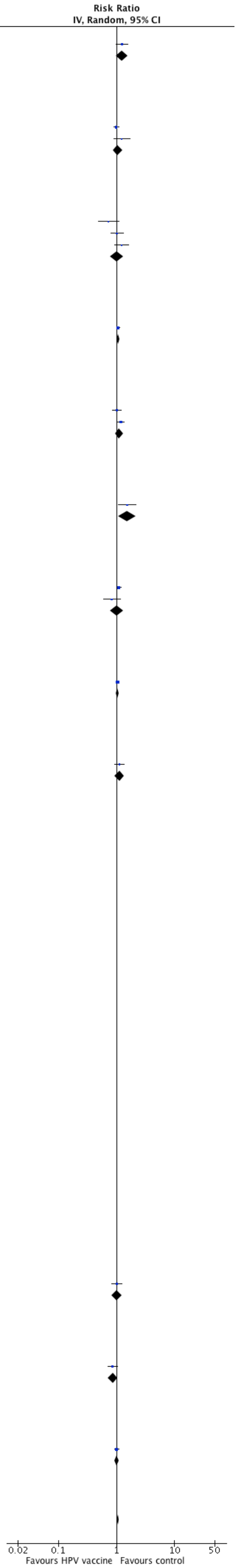

*12.29. Risk ratio for ‘solicited’ (GlaxoSmithKline): **1.08 [1.03, 1.14]**; risk ratio for ‘unsolicited’ (GlaxoSmithKline): **1.76 [1.26, 2.47]**; risk ratio for ‘systemic adverse events’ (Merck Sharp & Dohme): 0.98 [0.90, 1.07]. To avoid double counting of participants in the total risk ratio estimate, we excluded the ‘unsolicited’ adverse events from total risk ratio estimate for studies that reported ‘solicited’ adverse events.

12.30. Most common general harms (‘solicited’ and ‘unsolicited’*) - ‘myalgia’: intention to treat analysis

Study or Subgroup	HPV vaccine Events	HPV vaccine Total	Control Events	Control Total	Weight	Risk Ratio IV, Random, 95% CI
**12.30.1 General myalgia ('solicited'): Cervarix vs. Al(OH)3 (females, age 9-17; follow-up until 12 months)**						
HPV-058: Cervarix vs. Al(OH)3 (f, 9-17; 12)	110	374	93	376	7.9%	1.19 [0.94, 1.51]
**Subtotal (95% CI)**		**374**		**376**	**7.9%**	**1.19 [0.94, 1.51]**
Total events	110		93			
Heterogeneity: Not applicable						
Test for overall effect: Z = 1.44 (P = 0.15)						
**12.30.2 General myalgia ('solicited'): Cervarix vs. Al(OH)3 (females, age 15-25; follow-up until 7 months)**						
HPV-038: Cervarix vs. Al(OH)3 (f, 15-25; 7)	97	298	33	152	6.0%	1.50 [1.06, 2.11]
**Subtotal (95% CI)**		**298**		**152**	**6.0%**	**1.50 [1.06, 2.11]**
Total events	97		33			
Heterogeneity: Not applicable						
Test for overall effect: Z = 2.31 (P = 0.02)						
**12.30.3 General myalgia ('solicited'): Cervarix vs. Al(OH)3 (females, age 18-35; follow-up until 12 months)**						
HPV-003: Cervarix vs. Al(OH)3 (f, 18-30; 12)	1	31	0	30	0.2%	2.91 [0.12, 68.66]
HPV-031: Cervarix vs. Al(OH)3 (f, 18-35; 7)	10	176	11	178	1.9%	0.92 [0.40, 2.11]
HPV-035: Cervarix vs. Al(OH)3 (f, 18-35; 7)	88	150	61	150	7.9%	1.44 [1.14, 1.83]
**Subtotal (95% CI)**		**357**		**358**	**10.0%**	**1.40 [1.12, 1.76]**
Total events	99		72			
Heterogeneity: $Tau^2 = 0.00$; $Chi^2 = 1.25$, df = 2 (P = 0.53); $I^2 = 0\%$						
Test for overall effect: Z = 2.92 (P = 0.003)						
**12.30.4 General myalgia ('solicited'): Cervarix vs. Al(OH)3 (females, age 24-72; follow-up until 36 months)**						
HPV-015: Cervarix vs. Al(OH)3 (f, 24-72; 36)	878	2882	622	2871	10.4%	1.41 [1.29, 1.54]
**Subtotal (95% CI)**		**2882**		**2871**	**10.4%**	**1.41 [1.29, 1.54]**
Total events	878		622			
Heterogeneity: Not applicable						
Test for overall effect: Z = 7.53 (P < 0.00001)						
**12.30.5 General myalgia ('solicited'): Cervarix vs. Engerix: hepatitis B vaccine (females and males, age 9-16; follow-up until 72 months)**						
HPV-030: Cervarix vs. Engerix (f, 9-15; 12)	55	247	53	246	6.2%	1.03 [0.74, 1.44]
HPV-040: Cervarix vs. Engerix (f & m, 12-16; 72)	321	604	250	1028	9.8%	2.19 [1.92, 2.49]
**Subtotal (95% CI)**		**851**		**1274**	**16.0%**	**1.53 [0.73, 3.18]**
Total events	376		303			
Heterogeneity: $Tau^2 = 0.26$; $Chi^2 = 16.78$, df = 1 (P < 0.0001); $I^2 = 94\%$						
Test for overall effect: Z = 1.13 (P = 0.26)						
**12.30.6 General myalgia ('solicited'): Cervarix vs. Engerix: hepatitis B vaccine (females, age 26-46; follow-up until 12 months)**						
HPV-069: Cervarix vs. Engerix (f, 26-46; 12)	69	606	42	606	5.7%	1.64 [1.14, 2.37]
**Subtotal (95% CI)**		**606**		**606**	**5.7%**	**1.64 [1.14, 2.37]**
Total events	69		42			
Heterogeneity: Not applicable						
Test for overall effect: Z = 2.65 (P = 0.008)						
**12.30.7 General myalgia ('solicited'): Cervarix vs. Havrix: hepatitis A vaccine (females, age 9-15; follow-up until 12 months)**						
HPV-013: Cervarix vs. Havrix (f, 10-14; 12)	509	1035	340	1032	10.1%	1.49 [1.34, 1.66]
HPV-033: Cervarix vs. Havrix (f, 9-15; 7)	49	160	29	161	5.2%	1.70 [1.14, 2.55]
**Subtotal (95% CI)**		**1195**		**1193**	**15.3%**	**1.51 [1.36, 1.67]**
Total events	558		369			
Heterogeneity: $Tau^2 = 0.00$; $Chi^2 = 0.37$, df = 1 (P = 0.54); $I^2 = 0\%$						
Test for overall effect: Z = 7.76 (P < 0.00001)						
**12.30.8 General myalgia ('solicited'): Cervarix vs. Havrix: hepatitis A vaccine (females, age 15-25; follow-up until 48 months)**						
HPV-008: Cervarix vs. Havrix (f, 15-25; 48)	1607	9319	1381	9325	10.6%	1.16 [1.09, 1.24]
**Subtotal (95% CI)**		**9319**		**9325**	**10.6%**	**1.16 [1.09, 1.24]**
Total events	1607		1381			
Heterogeneity: Not applicable						
Test for overall effect: Z = 4.52 (P < 0.00001)						
**12.30.9 General myalgia ('solicited'): Cervarix vs. Twinrix: hepatitis A and B vaccine (females, age 8-15; follow-up until 12 months)**						
HPV-029: Cervarix vs. Twinrix (f, 8-15; 12)	99	270	75	271	7.7%	1.32 [1.03, 1.70]
**Subtotal (95% CI)**		**270**		**271**	**7.7%**	**1.32 [1.03, 1.70]**
Total events	99		75			
Heterogeneity: Not applicable						
Test for overall effect: Z = 2.22 (P = 0.03)						
**12.30.10 General myalgia ('unsolicited'): Cervarix vs. Al(OH)3 (females, age 15-25; follow-up until 7 months)**						
HPV-038: Cervarix vs. Al(OH)3 (f, 15-25; 7)	1	149	0	76	0.0%	1.54 [0.06, 37.36]
**Subtotal (95% CI)**		**0**		**0**		**Not estimable**
Total events	0		0			
Heterogeneity: Not applicable						
Test for overall effect: Not applicable						
**12.30.11 General myalgia ('unsolicited'): Cervarix vs. Al(OH)3 (females, age 18-35; follow-up until 7 months)**						
HPV-031: Cervarix vs. Al(OH)3 (f, 18-35; 7)	0	176	1	178	0.0%	0.34 [0.01, 8.22]
**Subtotal (95% CI)**		**0**		**0**		**Not estimable**
Total events	0		0			
Heterogeneity: Not applicable						
Test for overall effect: Not applicable						
**12.30.12 General myalgia ('unsolicited'): Cervarix vs. Engerix: hepatitis B vaccine (females and males, age 12-16; follow-up until 72 months)**						
HPV-040: Cervarix vs. Engerix (f & m, 12-16; 72)	2	1908	0	3128	0.0%	8.20 [0.39, 170.61]
**Subtotal (95% CI)**		**0**		**0**		**Not estimable**
Total events	0		0			
Heterogeneity: Not applicable						
Test for overall effect: Not applicable						
**12.30.13 General myalgia ('unsolicited'): Cervarix vs. Havrix: hepatitis A vaccine (females, age 9-15; follow-up until 7 months)**						
HPV-033: Cervarix vs. Havrix (f, 9-15; 7)	0	160	1	161	0.2%	0.34 [0.01, 8.17]
**Subtotal (95% CI)**		**160**		**161**	**0.2%**	**0.34 [0.01, 8.17]**
Total events	0		1			
Heterogeneity: Not applicable						
Test for overall effect: Z = 0.67 (P = 0.50)						
**12.30.14 General myalgia ('systemic adverse events'): Gardasil vs. carrier solution (females and males, age 9-16; follow-up until 18 months)**						
V501-018: Gardasil vs. carrier solu. (f&m,9-16;18)	31	1184	10	597	2.5%	1.56 [0.77, 3.17]
**Subtotal (95% CI)**		**1184**		**597**	**2.5%**	**1.56 [0.77, 3.17]**
Total events	31		10			
Heterogeneity: Not applicable						
Test for overall effect: Z = 1.24 (P = 0.21)						
**12.30.15 General myalgia ('systemic adverse events'): Gardasil vs. AAHS (males, age 15-27; follow-up until 36 months)**						
V501-020: Gardasil vs. AAHS (m, 15-27; 36)	18	2032	11	2033	2.3%	1.64 [0.78, 3.46]
**Subtotal (95% CI)**		**2032**		**2033**	**2.3%**	**1.64 [0.78, 3.46]**
Total events	18		11			
Heterogeneity: Not applicable						
Test for overall effect: Z = 1.29 (P = 0.20)						
**12.30.16 General myalgia ('systemic adverse events'): Gardasil 9 vs. saline placebo (females, age 12-26; follow-up until 7 months)**						
V503-006: Gardasil 9 vs. saline (f, 12-26;7)	13	618	5	306	1.3%	1.29 [0.46, 3.58]
**Subtotal (95% CI)**		**618**		**306**	**1.3%**	**1.29 [0.46, 3.58]**
Total events	13		5			
Heterogeneity: Not applicable						
Test for overall effect: Z = 0.48 (P = 0.63)						
**12.30.17 General myalgia ('systemic adverse events'): HPV 16 vaccine vs. AAHS (females, age 16-25; follow-up until 48 months)**						
V501-005: HPV 16 vaccine vs. AAHS (f, 16-25; 48)	34	1204	30	1205	4.2%	1.13 [0.70, 1.84]
**Subtotal (95% CI)**		**1204**		**1205**	**4.2%**	**1.13 [0.70, 1.84]**
Total events	34		30			
Heterogeneity: Not applicable						
Test for overall effect: Z = 0.51 (P = 0.61)						
**Total (95% CI)**		**21350**		**20728**	**100.0%**	**1.41 [1.24, 1.60]**
Total events	3989		3047			
Heterogeneity: $Tau^2 = 0.04$; $Chi^2 = 84.24$, df = 17 (P < 0.00001); $I^2 = 80\%$						
Test for overall effect: Z = 5.34 (P < 0.00001)						
Test for subgroup differences: $Chi^2 = 26.48$, df = 13 (P = 0.01), $I^2 = 50.9\%$						

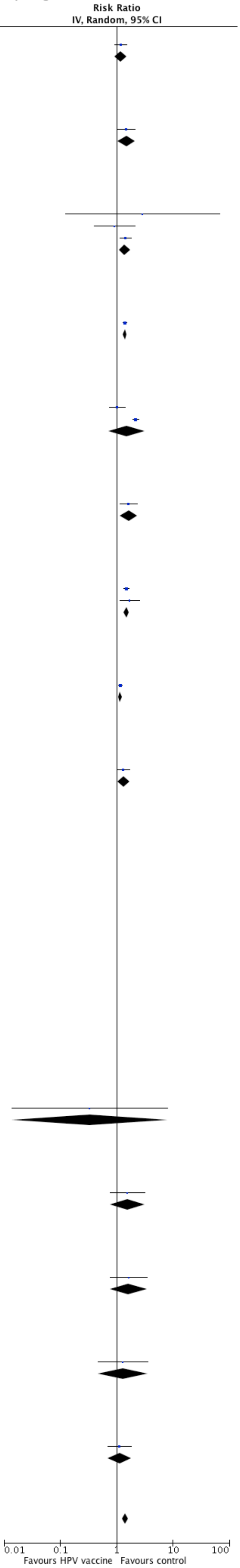

*12.30. Risk ratio for ‘solicited’ (GlaxoSmithKline): **1.42 [1.24, 1.63]**; risk ratio for ‘unsolicited’ (GlaxoSmithKline): 1.15 [0.24, 5.57]; risk ratio for ‘systemic adverse events’ (Merck Sharp & Dohme): 1.33 [0.95, 1.85]. To avoid double counting of participants in the total risk ratio estimate, we excluded the ‘unsolicited’ adverse events from total risk ratio estimate for studies that reported ‘solicited’ adverse events.

12.31. Most common general harms (‘systemic adverse events’) - ‘headache’: intention to treat analysis

See analysis 12.29.

12.32. Most common general harms (‘systemic adverse events’*) - ‘pyrexia’: intention to treat analysis

Study or Subgroup	HPV vaccine Events	HPV vaccine Total	Control Events	Control Total	Weight	Risk Ratio IV, Random, 95% CI
**12.32.1 General pyrexia ('solicited'): Cervarix vs. Al(OH)3 (females, age 9-17; follow-up until 12 months)**						
HPV-058: Cervarix vs. Al(OH)3 (f, 9-17; 12)	86	374	74	376	7.3%	1.17 [0.89, 1.54]
**Subtotal (95% CI)**		**374**		**376**	**7.3%**	**1.17 [0.89, 1.54]**
Total events	86		74			
Heterogeneity: Not applicable						
Test for overall effect: Z = 1.11 (P = 0.27)						
**12.32.2 General pyrexia ('solicited'): Cervarix vs. Al(OH)3 (females, age 15-26; follow-up until 27 months)**						
HPV-001: Cervarix vs. Al(OH)3 (f, 15-26; 27)	88	560	73	553	6.9%	1.19 [0.89, 1.59]
HPV-038: Cervarix vs. Al(OH)3 (f, 15-25; 7)	7	149	2	76	0.3%	1.79 [0.38, 8.39]
**Subtotal (95% CI)**		**709**		**629**	**7.2%**	**1.21 [0.91, 1.60]**
Total events	95		75			
Heterogeneity: Tau^2 = 0.00; Chi^2 = 0.25, df = 1 (P = 0.61); I^2 = 0%						
Test for overall effect: Z = 1.30 (P = 0.19)						
**12.32.3 General pyrexia ('solicited'): Cervarix vs. Al(OH)3 (females, age 18-35; follow-up until 12 months)**						
HPV-003: Cervarix vs. Al(OH)3 (f, 18-30; 12)	11	31	13	30	1.8%	0.82 [0.44, 1.53]
HPV-031: Cervarix vs. Al(OH)3 (f, 18-35; 7)	51	176	48	178	5.4%	1.07 [0.77, 1.50]
HPV-035: Cervarix vs. Al(OH)3 (f, 18-35; 7)	26	150	23	150	2.6%	1.13 [0.68, 1.89]
**Subtotal (95% CI)**		**357**		**358**	**9.8%**	**1.04 [0.81, 1.34]**
Total events	88		84			
Heterogeneity: Tau^2 = 0.00; Chi^2 = 0.70, df = 2 (P = 0.71); I^2 = 0%						
Test for overall effect: Z = 0.30 (P = 0.76)						
**12.32.4 General pyrexia ('solicited'): Cervarix vs. Al(OH)3 (females, age 24-72; follow-up until 36 months)**						
HPV-015: Cervarix vs. Al(OH)3 (f, 24-72; 36)	308	2882	248	2871	14.5%	1.24 [1.06, 1.45]
**Subtotal (95% CI)**		**2882**		**2871**	**14.5%**	**1.24 [1.06, 1.45]**
Total events	308		248			
Heterogeneity: Not applicable						
Test for overall effect: Z = 2.62 (P = 0.009)						
**12.32.5 General pyrexia ('solicited'): Cervarix vs. Engerix: hepatitis B vaccine (females and males, age 9-16; follow-up until 72 months)**						
HPV-030: Cervarix vs. Engerix (f, 9-15; 12)	23	247	36	246	2.8%	0.64 [0.39, 1.04]
HPV-040: Cervarix vs. Engerix (f & m, 12-16; 72)	48	604	85	1028	5.3%	0.96 [0.68, 1.35]
**Subtotal (95% CI)**		**851**		**1274**	**8.1%**	**0.81 [0.55, 1.21]**
Total events	71		121			
Heterogeneity: Tau^2 = 0.04; Chi^2 = 1.83, df = 1 (P = 0.18); I^2 = 45%						
Test for overall effect: Z = 1.02 (P = 0.31)						
**12.32.6 General pyrexia ('solicited'): Cervarix vs. Engerix: hepatitis B vaccine (females, age 26-46; follow-up until 12 months)**						
HPV-069: Cervarix vs. Engerix (f, 26-46; 12)	28	606	24	606	2.4%	1.17 [0.68, 1.99]
**Subtotal (95% CI)**		**606**		**606**	**2.4%**	**1.17 [0.68, 1.99]**
Total events	28		24			
Heterogeneity: Not applicable						
Test for overall effect: Z = 0.57 (P = 0.57)						
**12.32.7 General pyrexia ('solicited'): Cervarix vs. Havrix: hepatitis A vaccine (females, age 9-15; follow-up until 12 months)**						
HPV-013: Cervarix vs. Havrix (f, 10-14; 12)	193	1035	164	1032	12.0%	1.17 [0.97, 1.42]
HPV-033: Cervarix vs. Havrix (f, 9-15; 7)	13	160	6	161	0.8%	2.18 [0.85, 5.59]
**Subtotal (95% CI)**		**1195**		**1193**	**12.9%**	**1.34 [0.81, 2.19]**
Total events	206		170			
Heterogeneity: Tau^2 = 0.07; Chi^2 = 1.60, df = 1 (P = 0.21); I^2 = 37%						
Test for overall effect: Z = 1.15 (P = 0.25)						
**12.32.8 General pyrexia ('solicited'): Cervarix vs. Havrix: hepatitis A vaccine (females, age 15-25; follow-up until 48 months)**						
HPV-008: Cervarix vs. Havrix (f, 15-25; 48)	385	9319	342	9325	16.0%	1.13 [0.98, 1.30]
**Subtotal (95% CI)**		**9319**		**9325**	**16.0%**	**1.13 [0.98, 1.30]**
Total events	385		342			
Heterogeneity: Not applicable						
Test for overall effect: Z = 1.63 (P = 0.10)						
**12.32.9 General pyrexia ('solicited'): Cervarix vs. Twinrix: hepatitis A and B vaccine (females, age 8-15; follow-up until 12 months)**						
HPV-029: Cervarix vs. Twinrix (f, 8-15; 12)	28	270	14	271	1.8%	2.01 [1.08, 3.73]
**Subtotal (95% CI)**		**270**		**271**	**1.8%**	**2.01 [1.08, 3.73]**
Total events	28		14			
Heterogeneity: Not applicable						
Test for overall effect: Z = 2.21 (P = 0.03)						
**12.32.10 General pyrexia ('unsolicited'): Cervarix vs. Al(OH)3 (females, age 9-17; follow-up until 12 months)**						
HPV-058: Cervarix vs. Al(OH)3 (f, 9-17; 12)	1	374	2	376		Not estimable
**Subtotal (95% CI)**		**0**		**0**		**Not estimable**
Total events	0		0			
Heterogeneity: Not applicable						
Test for overall effect: Not applicable						
**12.32.11 General pyrexia ('unsolicited'): Cervarix vs. Al(OH)3 (females, age 18-35; follow-up until 7 months)**						
HPV-031: Cervarix vs. Al(OH)3 (f, 18-35; 7)	2	176	0	178		Not estimable
HPV-035: Cervarix vs. Al(OH)3 (f, 18-35; 7)	1	150	1	150		Not estimable
**Subtotal (95% CI)**		**0**		**0**		**Not estimable**
Total events	0		0			
Heterogeneity: Not applicable						
Test for overall effect: Not applicable						
**12.32.12 General pyrexia ('unsolicited'): Cervarix vs. Engerix: hepatitis B vaccine (females and males, age 9-16; follow-up until 72 months)**						
HPV-030: Cervarix vs. Engerix (f, 9-15; 12)	12	247	5	246		Not estimable
HPV-040: Cervarix vs. Engerix (f & m, 12-16; 72)	18	1908	23	3128		Not estimable
**Subtotal (95% CI)**		**0**		**0**		**Not estimable**
Total events	0		0			
Heterogeneity: Not applicable						
Test for overall effect: Not applicable						
**12.32.13 General pyrexia ('unsolicited'): Cervarix vs. Havrix: hepatitis A vaccine (females, age 9-15; follow-up until 7 months)**						
HPV-033: Cervarix vs. Havrix (f, 9-15; 7)	1	160	1	161		Not estimable
**Subtotal (95% CI)**		**0**		**0**		**Not estimable**
Total events	0		0			
Heterogeneity: Not applicable						
Test for overall effect: Not applicable						
**12.32.14 General pyrexia ('unsolicited'): Cervarix vs. Twinrix: hepatitis A and B vaccine (females, age 8-15; follow-up until 12 months)**						
HPV-029: Cervarix vs. Twinrix (f, 8-15; 12)	5	270	3	271		Not estimable
**Subtotal (95% CI)**		**0**		**0**		**Not estimable**
Total events	0		0			
Heterogeneity: Not applicable						
Test for overall effect: Not applicable						
**12.32.15 General pyrexia ('systemic adverse events'): Gardasil vs. carrier solution (females and males, age 9-16; follow-up until 18 months)**						
V501-018: Gardasil vs. carrier solu. (f&m,9-16;18)	100	1184	45	597	5.4%	1.12 [0.80, 1.57]
**Subtotal (95% CI)**		**1184**		**597**	**5.4%**	**1.12 [0.80, 1.57]**
Total events	100		45			
Heterogeneity: Not applicable						
Test for overall effect: Z = 0.66 (P = 0.51)						
**12.32.16 General pyrexia ('systemic adverse events'): Gardasil vs. AAHS (males, age 15-27; follow-up until 36 months)**						
V501-020: Gardasil vs. AAHS (m, 15-27; 36)	120	2032	125	2033	8.8%	0.96 [0.75, 1.22]
**Subtotal (95% CI)**		**2032**		**2033**	**8.8%**	**0.96 [0.75, 1.22]**
Total events	120		125			
Heterogeneity: Not applicable						
Test for overall effect: Z = 0.33 (P = 0.74)						
**12.32.17 General pyrexia ('systemic adverse events'): Gardasil 9 vs. saline placebo (females, age 12-26; follow-up until 7 months)**						
V503-006: Gardasil 9 vs. saline (f, 12-26;7)	41	618	10	306	1.6%	2.03 [1.03, 4.00]
**Subtotal (95% CI)**		**618**		**306**	**1.6%**	**2.03 [1.03, 4.00]**
Total events	41		10			
Heterogeneity: Not applicable						
Test for overall effect: Z = 2.05 (P = 0.04)						
**12.32.18 General pyrexia ('systemic adverse events'): HPV 16 vaccine vs. AAHS (females, age 16-25; follow-up until 48 months)**						
V501-005: HPV 16 vaccine vs. AAHS (f, 16-25; 48)	43	1204	54	1205	4.2%	0.80 [0.54, 1.18]
**Subtotal (95% CI)**		**1204**		**1205**	**4.2%**	**0.80 [0.54, 1.18]**
Total events	43		54			
Heterogeneity: Not applicable						
Test for overall effect: Z = 1.13 (P = 0.26)						
**Total (95% CI)**		**21601**		**21044**	**100.0%**	**1.12 [1.02, 1.22]**
Total events	1599		1386			
Heterogeneity: Tau^2 = 0.01; Chi^2 = 21.94, df = 17 (P = 0.19); I^2 = 23%						
Test for overall effect: Z = 2.49 (P = 0.01)						
Test for subgroup differences: Chi^2 = 15.95, df = 12 (P = 0.19), I^2 = 24.8%						

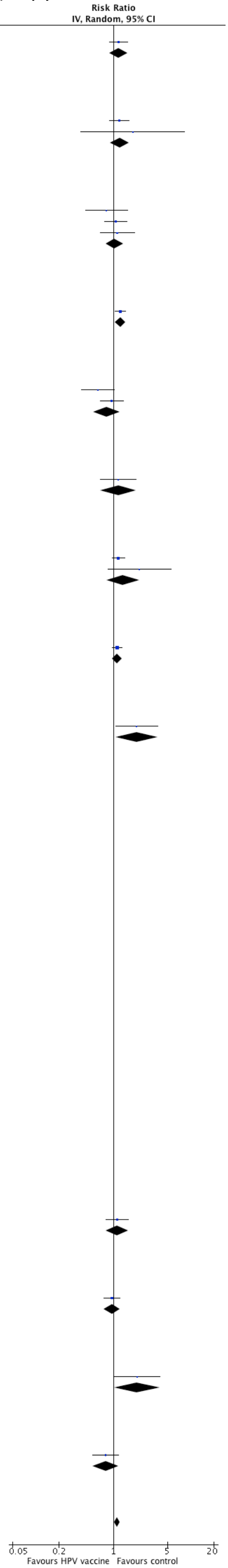

*12.32. Risk ratio for ‘solicited’ (GlaxoSmithKline): **1.15 [1.06, 1.25]**; risk ratio for ‘unsolicited’ (GlaxoSmithKline): 1.47 [0.93, 2.34]; risk ratio for ‘systemic adverse events’ (Merck Sharp & Dohme): 1.05 [0.80, 1.36]. To avoid double counting of participants in the total risk ratio estimate, we excluded the ‘unsolicited’ adverse events from total risk ratio estimate for studies that reported ‘solicited’ adverse events.

12.33. Most common general harms (‘systemic adverse events’*) - ‘nasopharyngitis’: intention to treat analysis

Study or Subgroup	HPV vaccine Events	HPV vaccine Total	Control Events	Control Total	Weight	Risk Ratio IV, Random, 95% CI
**12.33.1 General nasopharyngitis ('unsolicited'): Cervarix vs. Al(OH)3 (females, age 9-17; follow-up until 12 months)**						
HPV-058: Cervarix vs. Al(OH)3 (f, 9-17; 12)	22	374	19	376	9.2%	1.16 [0.64, 2.11]
**Subtotal (95% CI)**		**374**		**376**	**9.2%**	**1.16 [0.64, 2.11]**
Total events	22		19			
Heterogeneity: Not applicable						
Test for overall effect: Z = 0.50 (P = 0.62)						
**12.33.2 General nasopharyngitis ('unsolicited'): Cervarix vs. Al(OH)3 (females, age 18-35; follow-up until 7 months)**						
HPV-031: Cervarix vs. Al(OH)3 (f, 18-35; 7)	1	176	1	178	0.8%	1.01 [0.06, 16.04]
HPV-035: Cervarix vs. Al(OH)3 (f, 18-35; 7)	13	150	14	150	7.4%	0.93 [0.45, 1.91]
**Subtotal (95% CI)**		**326**		**328**	**8.2%**	**0.93 [0.47, 1.87]**
Total events	14		15			
Heterogeneity: Tau² = 0.00; Chi² = 0.00, df = 1 (P = 0.95); I² = 0%						
Test for overall effect: Z = 0.19 (P = 0.85)						
**12.33.3 General nasopharyngitis ('unsolicited'): Cervarix vs. Engerix: hepatitis B vaccine (females and males, age 9-16; follow-up until 72 months)**						
HPV-030: Cervarix vs. Engerix (f, 9-15; 12)	30	247	28	246	11.2%	1.07 [0.66, 1.73]
HPV-040: Cervarix vs. Engerix (f & m, 12-16; 72)	37	1908	17	3128	9.6%	3.57 [2.01, 6.32]
**Subtotal (95% CI)**		**2155**		**3374**	**20.8%**	**1.93 [0.59, 6.30]**
Total events	67		45			
Heterogeneity: Tau² = 0.66; Chi² = 9.98, df = 1 (P = 0.002); I² = 90%						
Test for overall effect: Z = 1.09 (P = 0.28)						
**12.33.4 General nasopharyngitis ('unsolicited'): Cervarix vs. Engerix: hepatitis B vaccine (females, age 26-46; follow-up until 12 months)**						
HPV-069: Cervarix vs. Engerix (f, 26-46; 12)	0	606	1	606	0.6%	0.33 [0.01, 8.17]
**Subtotal (95% CI)**		**606**		**606**	**0.6%**	**0.33 [0.01, 8.17]**
Total events	0		1			
Heterogeneity: Not applicable						
Test for overall effect: Z = 0.67 (P = 0.50)						
**12.33.5 General nasopharyngitis ('unsolicited'): Cervarix vs. Havrix: hepatitis A vaccine (females, age 9-15; follow-up until 7 months)**						
HPV-033: Cervarix vs. Havrix (f, 9-15; 7)	7	160	5	161	3.9%	1.41 [0.46, 4.35]
**Subtotal (95% CI)**		**160**		**161**	**3.9%**	**1.41 [0.46, 4.35]**
Total events	7		5			
Heterogeneity: Not applicable						
Test for overall effect: Z = 0.60 (P = 0.55)						
**12.33.6 General nasopharyngitis ('unsolicited'): Cervarix vs. Twinrix: hepatitis A and B vaccine (females, age 8-15; follow-up until 12 months)**						
HPV-029: Cervarix vs. Twinrix (f, 8-15; 12)	22	270	15	271	8.6%	1.47 [0.78, 2.78]
**Subtotal (95% CI)**		**270**		**271**	**8.6%**	**1.47 [0.78, 2.78]**
Total events	22		15			
Heterogeneity: Not applicable						
Test for overall effect: Z = 1.19 (P = 0.23)						
**12.33.7 General nasopharyngitis ('systemic adverse events'): Gardasil vs. carrier solution (females and males, age 9-16; follow-up until 18 months)**						
V501-018: Gardasil vs. carrier solu. (f&m,9-16;18)	34	1184	22	597	10.4%	0.78 [0.46, 1.32]
**Subtotal (95% CI)**		**1184**		**597**	**10.4%**	**0.78 [0.46, 1.32]**
Total events	34		22			
Heterogeneity: Not applicable						
Test for overall effect: Z = 0.93 (P = 0.35)						
**12.33.8 General nasopharyngitis ('systemic adverse events'): Gardasil vs. AAHS (males, age 15-27; follow-up until 36 months)**						
V501-020: Gardasil vs. AAHS (m, 15-27; 36)	44	2032	50	2033	12.9%	0.88 [0.59, 1.31]
**Subtotal (95% CI)**		**2032**		**2033**	**12.9%**	**0.88 [0.59, 1.31]**
Total events	44		50			
Heterogeneity: Not applicable						
Test for overall effect: Z = 0.62 (P = 0.53)						
**12.33.9 General nasopharyngitis ('systemic adverse events'): Gardasil 9 vs. saline placebo (females, age 12-26; follow-up until 7 months)**						
V503-006: Gardasil 9 vs. saline (f, 12-26;7)	44	618	17	306	10.1%	1.28 [0.74, 2.21]
**Subtotal (95% CI)**		**618**		**306**	**10.1%**	**1.28 [0.74, 2.21]**
Total events	44		17			
Heterogeneity: Not applicable						
Test for overall effect: Z = 0.90 (P = 0.37)						
**12.33.10 General nasopharyngitis ('systemic adverse events'): HPV 16 vaccine vs. AAHS (females, age 16-25; follow-up until 48 months)**						
V501-005: HPV 16 vaccine vs. AAHS (f, 16-25; 48)	85	1204	88	1205	15.4%	0.97 [0.73, 1.29]
**Subtotal (95% CI)**		**1204**		**1205**	**15.4%**	**0.97 [0.73, 1.29]**
Total events	85		88			
Heterogeneity: Not applicable						
Test for overall effect: Z = 0.23 (P = 0.82)						
**Total (95% CI)**		**8929**		**9257**	**100.0%**	**1.17 [0.91, 1.50]**
Total events	339		277			
Heterogeneity: Tau² = 0.08; Chi² = 21.98, df = 11 (P = 0.02); I² = 50%						
Test for overall effect: Z = 1.23 (P = 0.22)						
Test for subgroup differences: Chi² = 5.77, df = 9 (P = 0.76), I² = 0%						

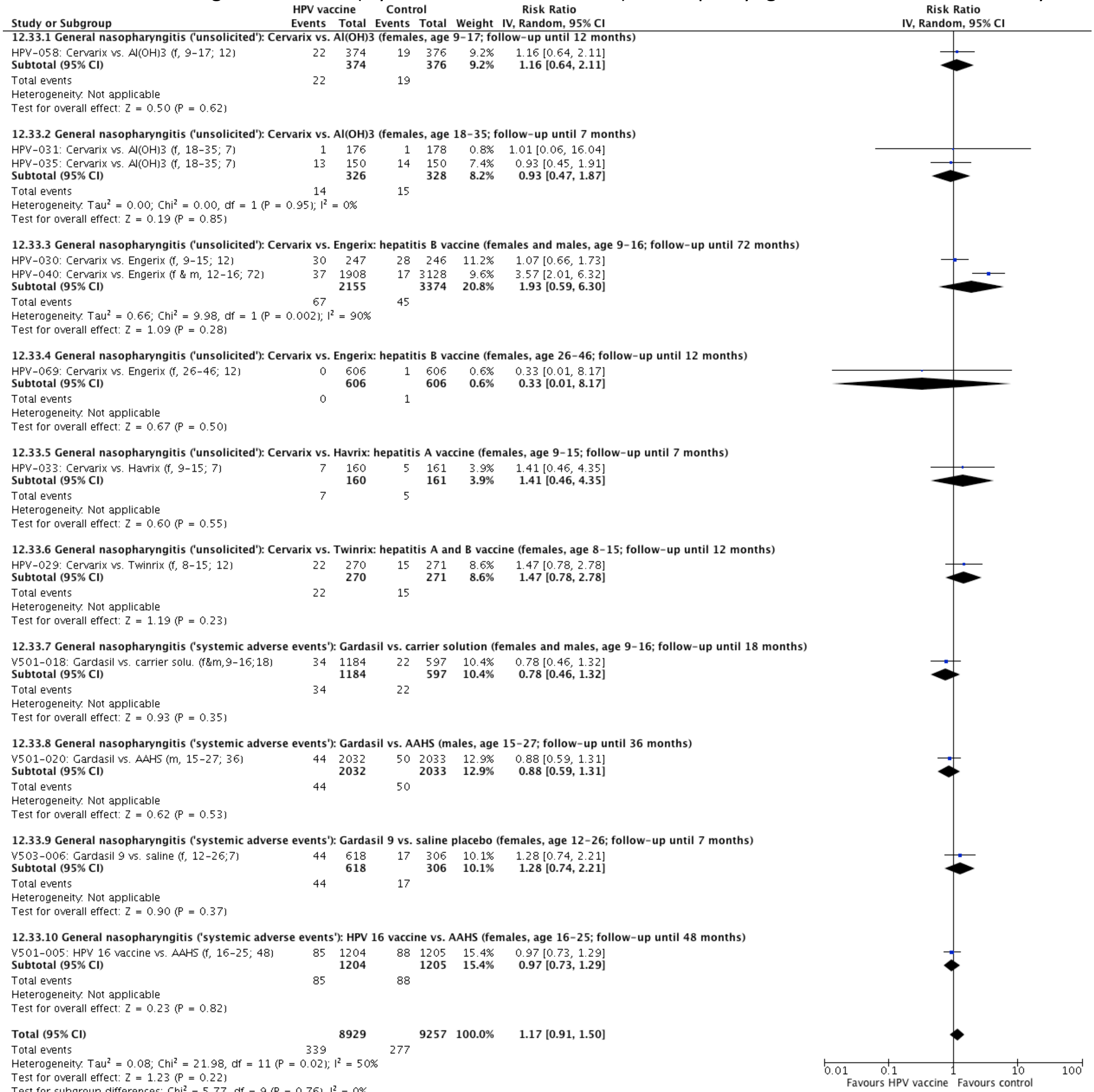

*12.33. Risk ratio for ‘solicited’ (GlaxoSmithKline): not applicable; risk ratio for ‘unsolicited’ (GlaxoSmithKline): 1.40 [0.94, 2.09]; risk ratio for ‘systemic adverse events’ (Merck Sharp & Dohme): 0.95 [0.78, 1.16].

12.34. General harms most increased by the HPV vaccines (‘solicited’ and ‘unsolicited’) - ‘myalgia’: intention to treat analysis
See analysis 12.30.

12.35. General harms most increased by the HPV vaccines (‘solicited’ and ‘unsolicited’) - ‘fatigue’: intention to treat analysis
See analysis 12.28.

12.36. General harms most increased by the HPV vaccines (‘solicited’ and ‘unsolicited’) - ‘headache’: intention to treat analysis
See analysis 12.29.

12.37. General harms most increased by the HPV vaccines (‘systemic adverse events’) - ‘myalgia’: intention to treat analysis
See analysis 12.30.

12.38. General harms most increased by the HPV vaccines (‘systemic adverse events’) - ‘pyrexia’: intention to treat analysis
See analysis 12.32.

12.39. General harms most increased by the HPV vaccines (‘systemic adverse events’*) - ‘nausea’: intention to treat analysis

Study or Subgroup	HPV vaccine Events	HPV vaccine Total	Control Events	Control Total	Weight	Risk Ratio IV, Random, 95% CI
**12.39.1 General nausea ('unsolicited'): Cervarix vs. Al(OH)3 (females, age 15-25; follow-up until 7 months)**						
HPV-038: Cervarix vs. Al(OH)3 (f, 15-25; 7)	1	149	0	76	0.9%	1.54 [0.06, 37.36]
**Subtotal (95% CI)**		**149**		**76**	**0.9%**	**1.54 [0.06, 37.36]**
Total events	1		0			
Heterogeneity: Not applicable						
Test for overall effect: Z = 0.27 (P = 0.79)						
**12.39.2 General nausea ('unsolicited'): Cervarix vs. Engerix: hepatitis B vaccine (females and males, age 9-16; follow-up until 72 months)**						
HPV-030: Cervarix vs. Engerix (f, 9-15; 12)	2	247	2	246	2.4%	1.00 [0.14, 7.01]
HPV-040: Cervarix vs. Engerix (f & m, 12-16; 72)	0	1908	1	3128	0.9%	0.55 [0.02, 13.40]
**Subtotal (95% CI)**		**2155**		**3374**	**3.3%**	**0.85 [0.16, 4.48]**
Total events	2		3			
Heterogeneity: Tau² = 0.00; Chi² = 0.10, df = 1 (P = 0.75); I² = 0%						
Test for overall effect: Z = 0.20 (P = 0.84)						
**12.39.3 General nausea ('unsolicited'): Cervarix vs. Twinrix: hepatitis A and B vaccine (females, age 8-15; follow-up until 12 months)**						
HPV-029: Cervarix vs. Twinrix (f, 8-15; 12)	2	270	0	271	1.0%	5.02 [0.24, 104.04]
**Subtotal (95% CI)**		**270**		**271**	**1.0%**	**5.02 [0.24, 104.04]**
Total events	2		0			
Heterogeneity: Not applicable						
Test for overall effect: Z = 1.04 (P = 0.30)						
**12.39.4 General nausea ('systemic adverse events'): Gardasil vs. carrier solution (females and males, age 9-16; follow-up until 18 months)**						
V501-018: Gardasil vs. carrier solu. (f&m,9-16;18)	38	1184	22	597	21.7%	0.87 [0.52, 1.46]
**Subtotal (95% CI)**		**1184**		**597**	**21.7%**	**0.87 [0.52, 1.46]**
Total events	38		22			
Heterogeneity: Not applicable						
Test for overall effect: Z = 0.53 (P = 0.60)						
**12.39.5 General nausea ('systemic adverse events'): Gardasil vs. AAHS (males, age 15-27; follow-up until 36 months)**						
V501-020: Gardasil vs. AAHS (m, 15-27; 36)	27	2032	16	2033	17.2%	1.69 [0.91, 3.12]
**Subtotal (95% CI)**		**2032**		**2033**	**17.2%**	**1.69 [0.91, 3.12]**
Total events	27		16			
Heterogeneity: Not applicable						
Test for overall effect: Z = 1.67 (P = 0.10)						
**12.39.6 General nausea ('systemic adverse events'): Gardasil 9 vs. saline placebo (females, age 12-26; follow-up until 7 months)**						
V503-006: Gardasil 9 vs. saline (f, 12-26;7)	52	618	12	306	17.3%	2.15 [1.16, 3.96]
**Subtotal (95% CI)**		**618**		**306**	**17.3%**	**2.15 [1.16, 3.96]**
Total events	52		12			
Heterogeneity: Not applicable						
Test for overall effect: Z = 2.44 (P = 0.01)						
**12.39.7 General nausea ('systemic adverse events'): HPV 16 vaccine vs. AAHS (females, age 16-25; follow-up until 48 months)**						
V501-005: HPV 16 vaccine vs. AAHS (f, 16-25; 48)	91	1204	95	1205	38.5%	0.96 [0.73, 1.26]
**Subtotal (95% CI)**		**1204**		**1205**	**38.5%**	**0.96 [0.73, 1.26]**
Total events	91		95			
Heterogeneity: Not applicable						
Test for overall effect: Z = 0.30 (P = 0.76)						
**Total (95% CI)**		**7612**		**7862**	**100.0%**	**1.21 [0.89, 1.65]**
Total events	213		148			
Heterogeneity: Tau² = 0.04; Chi² = 9.35, df = 7 (P = 0.23); I² = 25%						
Test for overall effect: Z = 1.22 (P = 0.22)						
Test for subgroup differences: Chi² = 9.25, df = 6 (P = 0.16), I² = 35.2%						

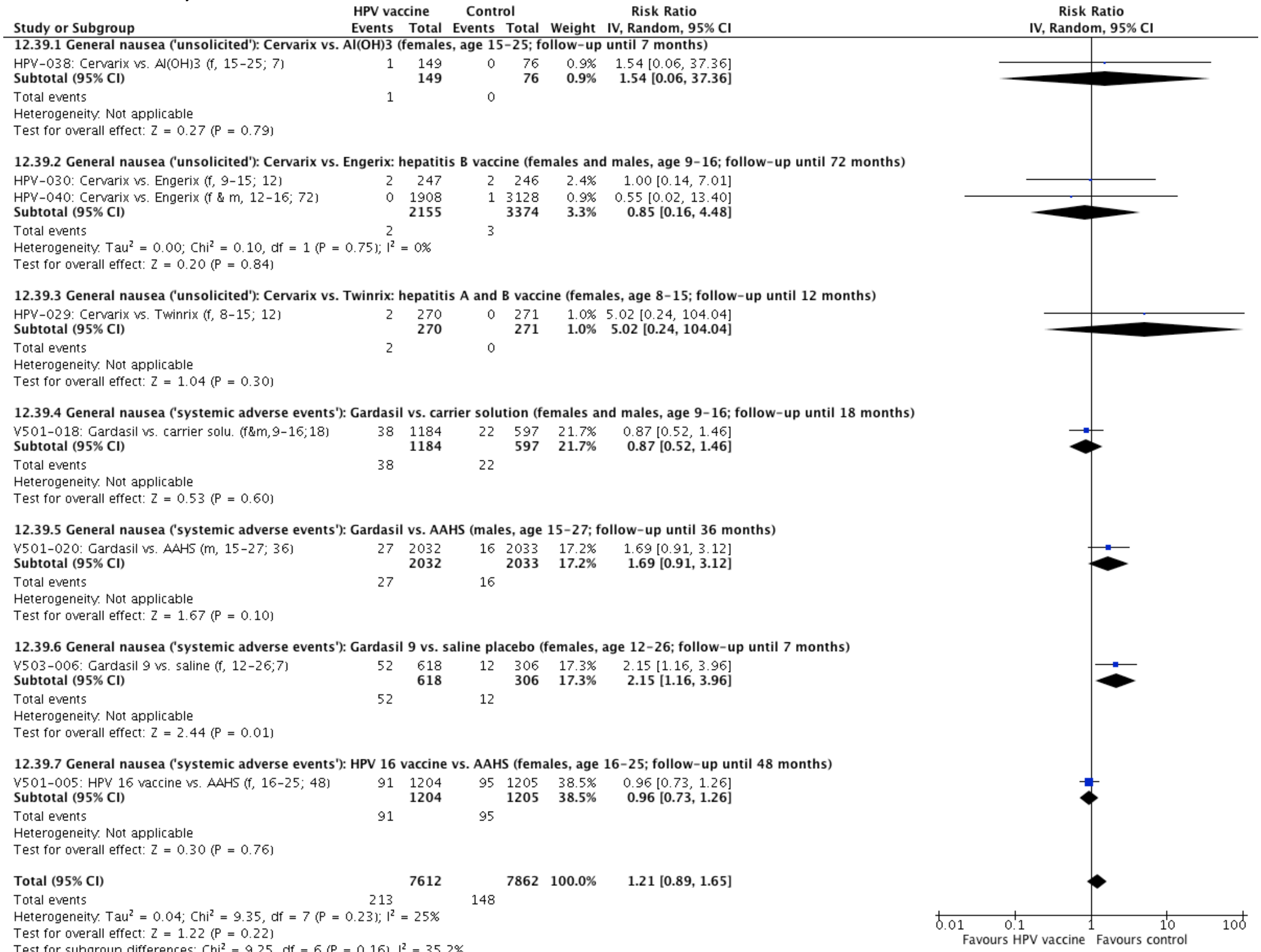

*12.39. Risk ratio for ‘solicited’ (GlaxoSmithKline): not applicable; risk ratio for ‘unsolicited’ (GlaxoSmithKline): 1.32 [0.35, 4.98]; risk ratio for ‘systemic adverse events’ (Merck Sharp & Dohme): 1.25 [0.84, 1.86].

12.40. General harms most decreased by the HPV vaccines ('solicited' and 'unsolicited'*) - 'influenza': intention to treat analysis

Study or Subgroup	HPV vaccine Events	HPV vaccine Total	Control Events	Control Total	Weight	Risk Ratio IV, Random, 95% CI
**12.40.1 General influenza ('unsolicited'): Cervarix vs. Al(OH)3 (females, age 18-35; follow-up until 7 months)**						
HPV-035: Cervarix vs. Al(OH)3 (f, 18-35; 7)	18	150	15	150	16.7%	1.20 [0.63, 2.29]
**Subtotal (95% CI)**		**150**		**150**	**16.7%**	**1.20 [0.63, 2.29]**
Total events	18		15			
Heterogeneity: Not applicable						
Test for overall effect: Z = 0.55 (P = 0.58)						
**12.40.2 General influenza ('unsolicited'): Cervarix vs. Engerix: hepatitis B vaccine (females and males, age 9-16; follow-up until 72 months)**						
HPV-030: Cervarix vs. Engerix (f, 9-15; 12)	2	247	4	246	4.8%	0.50 [0.09, 2.69]
HPV-040: Cervarix vs. Engerix (f & m, 12-16; 72)	5	1905	23	3128	10.9%	0.36 [0.14, 0.94]
**Subtotal (95% CI)**		**2152**		**3374**	**15.7%**	**0.39 [0.17, 0.90]**
Total events	7		27			
Heterogeneity: $Tau^2 = 0.00$; $Chi^2 = 0.11$, df = 1 (P = 0.74); $I^2 = 0\%$						
Test for overall effect: Z = 2.22 (P = 0.03)						
**12.40.3 General influenza ('unsolicited'): Cervarix vs. Engerix: hepatitis B vaccine (females, age 26-46; follow-up until 12 months)**						
HPV-069: Cervarix vs. Engerix (f, 26-46; 12)	1	606	0	606	1.5%	3.00 [0.12, 73.50]
**Subtotal (95% CI)**		**606**		**606**	**1.5%**	**3.00 [0.12, 73.50]**
Total events	1		0			
Heterogeneity: Not applicable						
Test for overall effect: Z = 0.67 (P = 0.50)						
**12.40.4 General influenza ('unsolicited'): Cervarix vs. Twinrix: hepatitis A and B vaccine (females, age 8-15; follow-up until 12 months)**						
HPV-029: Cervarix vs. Twinrix (f, 8-15; 12)	4	270	1	271	3.0%	4.01 [0.45, 35.69]
**Subtotal (95% CI)**		**270**		**271**	**3.0%**	**4.01 [0.45, 35.69]**
Total events	4		1			
Heterogeneity: Not applicable						
Test for overall effect: Z = 1.25 (P = 0.21)						
**12.40.5 General influenza ('systemic adverse events'): Gardasil vs. carrier solution (females and males, age 9-16; follow-up until 18 months)**						
V501-018: Gardasil vs. carrier solu. (f&m,9-16;18)	10	1184	12	597	13.0%	0.42 [0.18, 0.97]
**Subtotal (95% CI)**		**1184**		**597**	**13.0%**	**0.42 [0.18, 0.97]**
Total events	10		12			
Heterogeneity: Not applicable						
Test for overall effect: Z = 2.04 (P = 0.04)						
**12.40.6 General influenza ('systemic adverse events'): Gardasil vs. AAHS (males, age 15-27; follow-up until 36 months)**						
V501-020: Gardasil vs. AAHS (m, 15-27; 36)	42	2032	44	2033	22.3%	0.96 [0.63, 1.45]
**Subtotal (95% CI)**		**2032**		**2033**	**22.3%**	**0.96 [0.63, 1.45]**
Total events	42		44			
Heterogeneity: Not applicable						
Test for overall effect: Z = 0.22 (P = 0.83)						
**12.40.7 General influenza ('systemic adverse events'): Gardasil 9 vs. saline placebo (females, age 12-26; follow-up until 7 months)**						
V503-006: Gardasil 9 vs. saline (f, 12-26;7)	11	618	6	306	10.7%	0.91 [0.34, 2.43]
**Subtotal (95% CI)**		**618**		**306**	**10.7%**	**0.91 [0.34, 2.43]**
Total events	11		6			
Heterogeneity: Not applicable						
Test for overall effect: Z = 0.19 (P = 0.85)						
**12.40.8 General influenza ('systemic adverse events'): HPV 16 vaccine vs. AAHS (females, age 16-25; follow-up until 48 months)**						
V501-005: HPV 16 vaccine vs. AAHS (f, 16-25; 48)	26	1204	15	1205	17.1%	1.73 [0.92, 3.26]
**Subtotal (95% CI)**		**1204**		**1205**	**17.1%**	**1.73 [0.92, 3.26]**
Total events	26		15			
Heterogeneity: Not applicable						
Test for overall effect: Z = 1.71 (P = 0.09)						
**Total (95% CI)**		**8216**		**8542**	**100.0%**	**0.91 [0.61, 1.36]**
Total events	119		120			
Heterogeneity: $Tau^2 = 0.14$; $Chi^2 = 14.37$, df = 8 (P = 0.07); $I^2 = 44\%$						
Test for overall effect: Z = 0.47 (P = 0.64)						
Test for subgroup differences: $Chi^2 = 14.26$, df = 7 (P = 0.05), $I^2 = 50.9\%$						

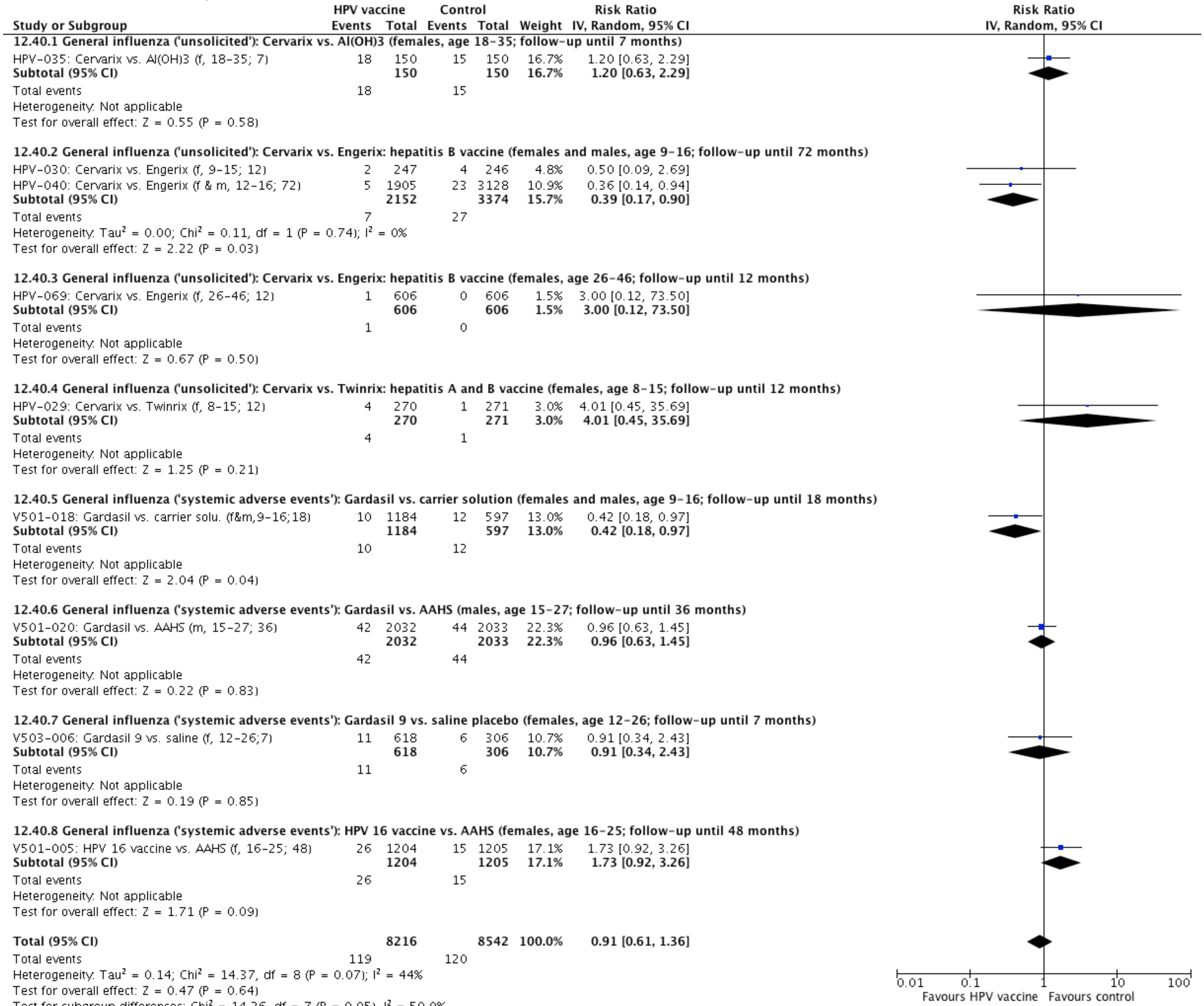

*12.40. Risk ratio for 'solicited' (GlaxoSmithKline): not applicable; risk ratio for 'unsolicited' (GlaxoSmithKline): 0.88 [0.39, 1.97]; risk ratio for 'systemic adverse events' (Merck Sharp & Dohme): 0.94 [0.56, 1.58].

12.41. General harms most decreased by the HPV vaccines (‘solicited’ and ‘unsolicited’*) - ‘cough’: intention to treat analysis

Study or Subgroup	HPV vaccine Events	HPV vaccine Total	Control Events	Control Total	Weight	Risk Ratio IV, Random, 95% CI
**12.41.1 General cough ('unsolicited'): Cervarix vs. Al(OH)3 (females, age 9-17; follow-up until 12 months)**						
HPV-058: Cervarix vs. Al(OH)3 (f, 9-17; 12)	3	374	1	376	1.8%	3.02 [0.32, 28.86]
**Subtotal (95% CI)**		**374**		**376**	**1.8%**	**3.02 [0.32, 28.86]**
Total events	3		1			
Heterogeneity: Not applicable						
Test for overall effect: Z = 0.96 (P = 0.34)						
**12.41.2 General cough ('unsolicited'): Cervarix vs. Al(OH)3 (females, age 18-35; follow-up until 7 months)**						
HPV-031: Cervarix vs. Al(OH)3 (f, 18-35; 7)	0	176	3	178	1.1%	0.14 [0.01, 2.78]
HPV-035: Cervarix vs. Al(OH)3 (f, 18-35; 7)	6	150	6	150	7.6%	1.00 [0.33, 3.03]
**Subtotal (95% CI)**		**326**		**328**	**8.7%**	**0.63 [0.13, 3.17]**
Total events	6		9			
Heterogeneity: Tau² = 0.57; Chi² = 1.44, df = 1 (P = 0.23); I² = 31%						
Test for overall effect: Z = 0.56 (P = 0.58)						
**12.41.3 General cough ('unsolicited'): Cervarix vs. Engerix: hepatitis B vaccine (females and males, age 9-16; follow-up until 72 months)**						
HPV-030: Cervarix vs. Engerix (f, 9-15; 12)	1	247	2	246	1.6%	0.50 [0.05, 5.46]
HPV-040: Cervarix vs. Engerix (f & m, 12-16; 72)	6	1908	16	3128	10.6%	0.61 [0.24, 1.57]
**Subtotal (95% CI)**		**2155**		**3374**	**12.3%**	**0.60 [0.25, 1.43]**
Total events	7		18			
Heterogeneity: Tau² = 0.00; Chi² = 0.03, df = 1 (P = 0.87); I² = 0%						
Test for overall effect: Z = 1.16 (P = 0.25)						
**12.41.4 General cough ('unsolicited'): Cervarix vs. Havrix: hepatitis A vaccine (females, age 9-15; follow-up until 7 months)**						
HPV-033: Cervarix vs. Havrix (f, 9-15; 7)	1	160	1	161	1.2%	1.01 [0.06, 15.95]
**Subtotal (95% CI)**		**160**		**161**	**1.2%**	**1.01 [0.06, 15.95]**
Total events	1		1			
Heterogeneity: Not applicable						
Test for overall effect: Z = 0.00 (P = 1.00)						
**12.41.5 General cough ('unsolicited'): Cervarix vs. Twinrix: hepatitis A and B vaccine (females, age 8-15; follow-up until 12 months)**						
HPV-029: Cervarix vs. Twinrix (f, 8-15; 12)	3	270	2	271	2.9%	1.51 [0.25, 8.94]
**Subtotal (95% CI)**		**270**		**271**	**2.9%**	**1.51 [0.25, 8.94]**
Total events	3		2			
Heterogeneity: Not applicable						
Test for overall effect: Z = 0.45 (P = 0.65)						
**12.41.6 General cough ('systemic adverse events'): Gardasil vs. carrier solution (females and males, age 9-16; follow-up until 18 months)**						
V501-018: Gardasil vs. carrier solu. (f&m,9-16;18)	14	1184	14	597	17.3%	0.50 [0.24, 1.05]
**Subtotal (95% CI)**		**1184**		**597**	**17.3%**	**0.50 [0.24, 1.05]**
Total events	14		14			
Heterogeneity: Not applicable						
Test for overall effect: Z = 1.83 (P = 0.07)						
**12.41.7 General cough ('systemic adverse events'): Gardasil vs. AAHS (males, age 15-27; follow-up until 36 months)**						
V501-020: Gardasil vs. AAHS (m, 15-27; 36)	15	2032	17	2033	19.5%	0.88 [0.44, 1.76]
**Subtotal (95% CI)**		**2032**		**2033**	**19.5%**	**0.88 [0.44, 1.76]**
Total events	15		17			
Heterogeneity: Not applicable						
Test for overall effect: Z = 0.35 (P = 0.72)						
**12.41.8 General cough ('systemic adverse events'): Gardasil 9 vs. saline placebo (females, age 12-26; follow-up until 7 months)**						
V503-006: Gardasil 9 vs. saline (f, 12-26;7)	12	618	5	306	8.7%	1.19 [0.42, 3.34]
**Subtotal (95% CI)**		**618**		**306**	**8.7%**	**1.19 [0.42, 3.34]**
Total events	12		5			
Heterogeneity: Not applicable						
Test for overall effect: Z = 0.33 (P = 0.74)						
**12.41.9 General cough ('systemic adverse events'): HPV 16 vaccine vs. AAHS (females, age 16-25; follow-up until 48 months)**						
V501-005: HPV 16 vaccine vs. AAHS (f, 16-25; 48)	25	1204	20	1205	27.5%	1.25 [0.70, 2.24]
**Subtotal (95% CI)**		**1204**		**1205**	**27.5%**	**1.25 [0.70, 2.24]**
Total events	25		20			
Heterogeneity: Not applicable						
Test for overall effect: Z = 0.75 (P = 0.45)						
**Total (95% CI)**		**8323**		**8651**	**100.0%**	**0.89 [0.65, 1.21]**
Total events	86		87			
Heterogeneity: Tau² = 0.00; Chi² = 7.70, df = 10 (P = 0.66); I² = 0%						
Test for overall effect: Z = 0.76 (P = 0.45)						
Test for subgroup differences: Chi² = 6.35, df = 8 (P = 0.61), I² = 0%						

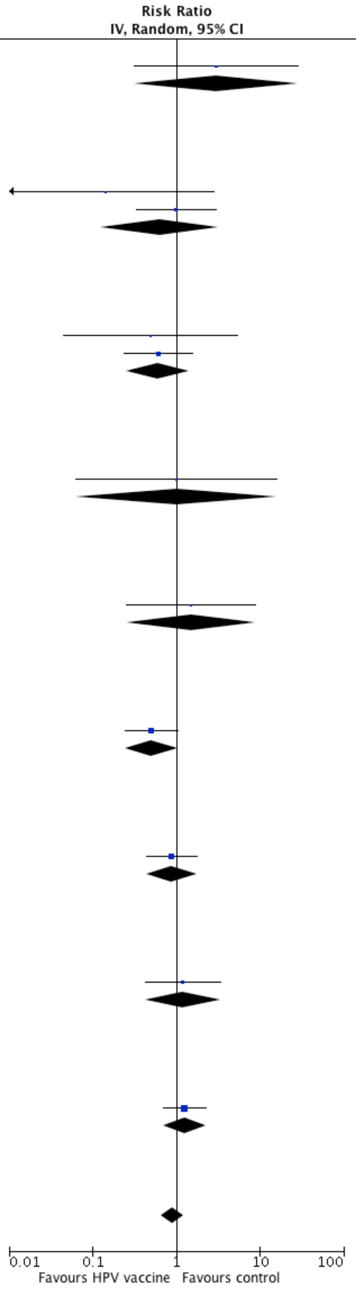

*12.41. Risk ratio for ‘solicited’ (GlaxoSmithKline): not applicable; risk ratio for ‘unsolicited’ (GlaxoSmithKline): 0.83 [0.46, 1.49]; risk ratio for ‘systemic adverse events’ (Merck Sharp & Dohme): 0.90 [0.60, 1.37].

12.42. General harms most decreased by the HPV vaccines (‘solicited’ and ‘unsolicited’*) - ‘oropharyngeal pain’: intention to treat analysis

Study or Subgroup	HPV vaccine Events	HPV vaccine Total	Control Events	Control Total	Weight	Risk Ratio IV, Random, 95% CI
**12.42.1 General oropharyngeal pain ('unsolicited'): Cervarix vs. Al(OH)3 (females, age 9-17; follow-up until 12 months)**						
HPV-058: Cervarix vs. Al(OH)3 (f, 9-17; 12)	1	374	3	376	1.9%	0.34 [0.04, 3.21]
**Subtotal (95% CI)**		**374**		**376**	**1.9%**	**0.34 [0.04, 3.21]**
Total events	1		3			
Heterogeneity: Not applicable						
Test for overall effect: Z = 0.95 (P = 0.34)						
**12.42.2 General oropharyngeal pain ('unsolicited'): Cervarix vs. Engerix: hepatitis B vaccine (females and males, age 9-16; follow-up until 72 months)**						
HPV-030: Cervarix vs. Engerix (f, 9-15; 12)	7	247	7	246	8.7%	1.00 [0.35, 2.80]
HPV-040: Cervarix vs. Engerix (f & m, 12-16; 72)	16	1908	34	3128	23.9%	0.77 [0.43, 1.39]
**Subtotal (95% CI)**		**2155**		**3374**	**32.6%**	**0.82 [0.49, 1.37]**
Total events	23		41			
Heterogeneity: $Tau^2 = 0.00$; $Chi^2 = 0.18$, df = 1 (P = 0.67); $I^2 = 0\%$						
Test for overall effect: Z = 0.75 (P = 0.45)						
**12.42.3 General oropharyngeal pain ('unsolicited'): Cervarix vs. Twinrix: hepatitis A and B vaccine (females, age 8-15; follow-up until 12 months)**						
HPV-029: Cervarix vs. Twinrix (f, 8-15; 12)	9	270	5	271	8.0%	1.81 [0.61, 5.32]
**Subtotal (95% CI)**		**270**		**271**	**8.0%**	**1.81 [0.61, 5.32]**
Total events	9		5			
Heterogeneity: Not applicable						
Test for overall effect: Z = 1.07 (P = 0.28)						
**12.42.4 General oropharyngeal pain ('systemic adverse events'): Gardasil vs. AAHS (males, age 15-27; follow-up until 36 months)**						
V501-020: Gardasil vs. AAHS (m, 15-27; 36)	38	2032	37	2033	37.3%	1.03 [0.66, 1.61]
**Subtotal (95% CI)**		**2032**		**2033**	**37.3%**	**1.03 [0.66, 1.61]**
Total events	38		37			
Heterogeneity: Not applicable						
Test for overall effect: Z = 0.12 (P = 0.91)						
**12.42.5 General oropharyngeal pain ('systemic adverse events'): Gardasil 9 vs. saline placebo (females, age 8-15; follow-up until 7 months)**						
V503-006: Gardasil 9 vs. saline (f, 12-26;7)	40	618	11	306	20.1%	1.80 [0.94, 3.46]
**Subtotal (95% CI)**		**618**		**306**	**20.1%**	**1.80 [0.94, 3.46]**
Total events	40		11			
Heterogeneity: Not applicable						
Test for overall effect: Z = 1.76 (P = 0.08)						
**Total (95% CI)**		**5449**		**6360**	**100.0%**	**1.10 [0.80, 1.50]**
Total events	111		97			
Heterogeneity: $Tau^2 = 0.02$; $Chi^2 = 5.56$, df = 5 (P = 0.35); $I^2 = 10\%$						
Test for overall effect: Z = 0.58 (P = 0.56)						
Test for subgroup differences: $Chi^2 = 5.39$, df = 4 (P = 0.25), $I^2 = 25.8\%$						

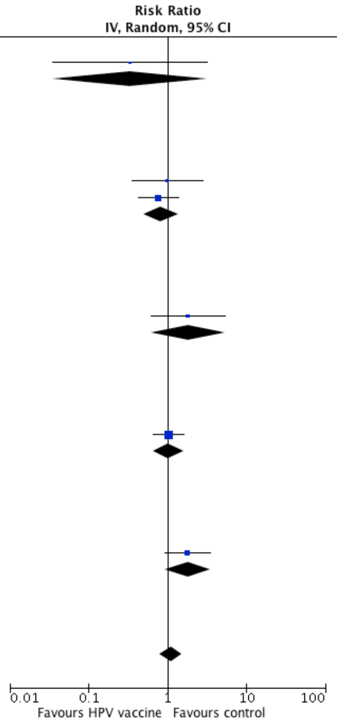

*12.42. Risk ratio for ‘solicited’ (GlaxoSmithKline): not applicable; risk ratio for ‘unsolicited’ (GlaxoSmithKline): 0.91 [0.58, 1.43]; risk ratio for ‘systemic adverse events’ (Merck Sharp & Dohme): 1.29 [0.75, 2.22].

12.43. General harms most decreased by the HPV vaccines (‘systemic adverse events’*) - ‘fungal infection’: intention to treat analysis

Study or Subgroup	HPV vaccine Events	HPV vaccine Total	Control Events	Control Total	Weight	Risk Ratio IV, Random, 95% CI
**12.43.1 General fungal infection ('unsolicited'): Cervarix vs. Al(OH)3 (females, age 18-35; follow-up until 7 months)**						
HPV-031: Cervarix vs. Al(OH)3 (f, 18-35; 7)	1	176	0	178	25.9%	3.03 [0.12, 73.97]
**Subtotal (95% CI)**		**176**		**178**	**25.9%**	**3.03 [0.12, 73.97]**
Total events	1		0			
Heterogeneity: Not applicable						
Test for overall effect: Z = 0.68 (P = 0.50)						
**12.43.2 General fungal infection ('unsolicited'): Cervarix vs. Engerix: hepatitis B vaccine (females and males, age 9-15; follow-up until 12 months)**						
HPV-030: Cervarix vs. Engerix (f, 9-15; 12)	1	247	0	246	25.9%	2.99 [0.12, 72.99]
**Subtotal (95% CI)**		**247**		**246**	**25.9%**	**2.99 [0.12, 72.99]**
Total events	1		0			
Heterogeneity: Not applicable						
Test for overall effect: Z = 0.67 (P = 0.50)						
**12.43.3 General fungal infection ('systemic adverse events'): HPV 16 vaccine vs. AAHS (females, age 16-25; follow-up until 48 months)**						
V501-005: HPV 16 vaccine vs. AAHS (f, 16-25; 48)	2	1204	11	1205	48.2%	0.18 [0.04, 0.82]
**Subtotal (95% CI)**		**1204**		**1205**	**48.2%**	**0.18 [0.04, 0.82]**
Total events	2		11			
Heterogeneity: Not applicable						
Test for overall effect: Z = 2.22 (P = 0.03)						
**Total (95% CI)**		**1627**		**1629**	**100.0%**	**0.78 [0.09, 6.43]**
Total events	4		11			
Heterogeneity: Tau^2 = 1.82; Chi^2 = 4.11, df = 2 (P = 0.13); I^2 = 51%						
Test for overall effect: Z = 0.23 (P = 0.82)						
Test for subgroup differences: Chi^2 = 4.11, df = 2 (P = 0.13), I^2 = 51.3%						

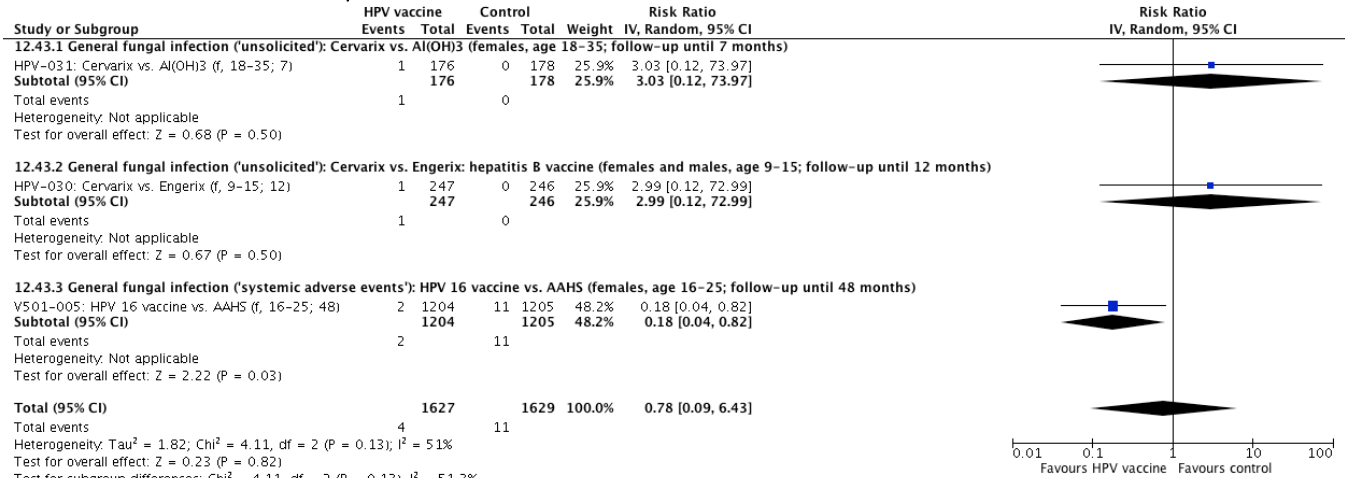

*12.43. Risk ratio for ‘solicited’ (GlaxoSmithKline): not applicable; risk ratio for ‘unsolicited’ (GlaxoSmithKline): 3.01 [0.31, 28.83]; risk ratio for ‘systemic adverse events’ (Merck Sharp & Dohme): **0.18 [0.04, 0.82]**.

12.44. General harms most decreased by the HPV vaccines (‘systemic adverse events’*) - ‘sinus headache’: intention to treat analysis

Study or Subgroup	HPV vaccine Events	HPV vaccine Total	Control Events	Control Total	Weight	Risk Ratio IV, Random, 95% CI
**12.44.1 General sinus headache ('systemic adverse events'): Gardasil vs. carrier solution (females and males, age 9-16; follow-up until 18 months)**						
V501-018: Gardasil vs. carrier solu. (f&m,9-16;18)	1	1184	0	597	6.8%	1.51 [0.06, 37.11]
**Subtotal (95% CI)**		**1184**		**597**	**6.8%**	**1.51 [0.06, 37.11]**
Total events	1		0			
Heterogeneity: Not applicable						
Test for overall effect: Z = 0.25 (P = 0.80)						
**12.44.2 General sinus headache ('systemic adverse events'): Gardasil vs. AAHS (males, age 15-27; follow-up until 36 months)**						
V501-020: Gardasil vs. AAHS (m, 15-27; 36)	1	2032	1	2033	9.0%	1.00 [0.06, 15.98]
**Subtotal (95% CI)**		**2032**		**2033**	**9.0%**	**1.00 [0.06, 15.98]**
Total events	1		1			
Heterogeneity: Not applicable						
Test for overall effect: Z = 0.00 (P = 1.00)						
**12.44.3 General sinus headache ('systemic adverse events'): Gardasil 9 vs. saline placebo (females, age 12-26; follow-up until 7 months)**						
V503-006: Gardasil 9 vs. saline (f, 12-26;7)	2	618	3	306	21.8%	0.33 [0.06, 1.97]
**Subtotal (95% CI)**		**618**		**306**	**21.8%**	**0.33 [0.06, 1.97]**
Total events	2		3			
Heterogeneity: Not applicable						
Test for overall effect: Z = 1.22 (P = 0.22)						
**12.44.4 General sinus headache ('systemic adverse events'): HPV 16 vaccine vs. AAHS (females, age 16-25; follow-up until 48 months)**						
V501-005: HPV 16 vaccine vs. AAHS (f, 16-25; 48)	5	1204	11	1205	62.4%	0.45 [0.16, 1.31]
**Subtotal (95% CI)**		**1204**		**1205**	**62.4%**	**0.45 [0.16, 1.31]**
Total events	5		11			
Heterogeneity: Not applicable						
Test for overall effect: Z = 1.46 (P = 0.14)						
**Total (95% CI)**		**5038**		**4141**	**100.0%**	**0.49 [0.21, 1.14]**
Total events	9		15			
Heterogeneity: Tau² = 0.00; Chi² = 0.94, df = 3 (P = 0.82); I² = 0%						
Test for overall effect: Z = 1.66 (P = 0.10)						
Test for subgroup differences: Chi² = 0.94, df = 3 (P = 0.82), I² = 0%						

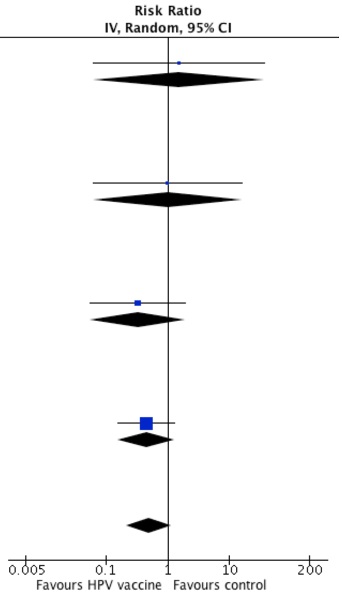

*12.44. Risk ratio for ‘solicited’ (GlaxoSmithKline): not applicable; risk ratio for ‘unsolicited’ (GlaxoSmithKline): not applicable; risk ratio for ‘systemic adverse events’ (Merck Sharp & Dohme): 0.49 [0.21, 1.14].

12.45. General harms most decreased by the HPV vaccines (‘systemic adverse events’*) - ‘joint injury’: intention to treat analysis

Study or Subgroup	HPV vaccine Events	HPV vaccine Total	Control Events	Control Total	Weight	Risk Ratio IV, Random, 95% CI
**12.45.1 General joint injury ('unsolicited'): Cervarix vs. Engerix: hepatitis B vaccine (females, age 26-46; follow-up until 12 months)**						
HPV-069: Cervarix vs. Engerix (f, 26-46; 12)	1	606	0	606	19.2%	3.00 [0.12, 73.50]
**Subtotal (95% CI)**		**606**		**606**	**19.2%**	**3.00 [0.12, 73.50]**
Total events	1		0			
Heterogeneity: Not applicable						
Test for overall effect: Z = 0.67 (P = 0.50)						
**12.45.2 General joint injury ('unsolicited'): Cervarix vs. Twinrix: hepatitis A and B vaccine (females, age 8-15; follow-up until 12 months)**						
HPV-029: Cervarix vs. Twinrix (f, 8-15; 12)	1	270	0	271	19.2%	3.01 [0.12, 73.59]
**Subtotal (95% CI)**		**270**		**271**	**19.2%**	**3.01 [0.12, 73.59]**
Total events	1		0			
Heterogeneity: Not applicable						
Test for overall effect: Z = 0.68 (P = 0.50)						
**12.45.3 General joint injury ('systemic adverse events'): Gardasil vs. carrier solution (females and males, age 9-16; follow-up until 18 months)**						
V501-018: Gardasil vs. carrier solu. (f&m,9-16;18)	0	1184	2	597	21.2%	0.10 [0.00, 2.10]
**Subtotal (95% CI)**		**1184**		**597**	**21.2%**	**0.10 [0.00, 2.10]**
Total events	0		2			
Heterogeneity: Not applicable						
Test for overall effect: Z = 1.48 (P = 0.14)						
**12.45.4 General joint injury ('systemic adverse events'): Gardasil 9 vs. saline placebo (females, age 12-26; follow-up until 7 months)**						
V503-006: Gardasil 9 vs. saline (f, 12-26;7)	0	618	1	306	19.2%	0.17 [0.01, 4.05]
**Subtotal (95% CI)**		**618**		**306**	**19.2%**	**0.17 [0.01, 4.05]**
Total events	0		1			
Heterogeneity: Not applicable						
Test for overall effect: Z = 1.10 (P = 0.27)						
**12.45.5 General joint injury ('systemic adverse events'): Gardasil vs. AAHS (males, age 15-27; follow-up until 36 months)**						
V501-020: Gardasil vs. AAHS (m, 15-27; 36)	0	2032	2	2033	21.2%	0.20 [0.01, 4.17]
**Subtotal (95% CI)**		**2032**		**2033**	**21.2%**	**0.20 [0.01, 4.17]**
Total events	0		2			
Heterogeneity: Not applicable						
Test for overall effect: Z = 1.04 (P = 0.30)						
**Total (95% CI)**		**4710**		**3813**	**100.0%**	**0.47 [0.11, 2.01]**
Total events	2		5			
Heterogeneity: Tau² = 0.18; Chi² = 4.29, df = 4 (P = 0.37); I² = 7%						
Test for overall effect: Z = 1.01 (P = 0.31)						
Test for subgroup differences: Chi² = 4.29, df = 4 (P = 0.37), I² = 6.7%						

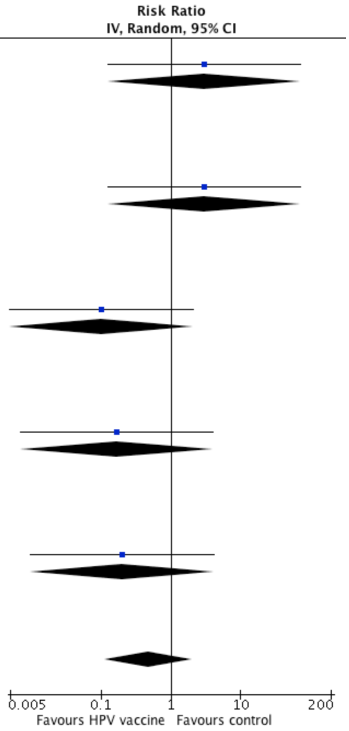

*12.45. Risk ratio for ‘solicited’ (GlaxoSmithKline): not applicable; risk ratio for ‘unsolicited’ (GlaxoSmithKline): 3.01 [0.31, 28.83]; risk ratio for ‘systemic adverse events’ (Merck Sharp & Dohme): **0.15 [0.03, 0.88]**.

13. Harms of special interest

13.1. Anaphylaxis*: intention to treat analysis

Study or Subgroup	HPV vaccine Events	HPV vaccine Total	Control Events	Control Total	Weight	Risk Ratio IV, Random, 95% CI
**13.1.1 Serious anaphylactic reaction: Cervarix vs. Al(OH)3 (females, age 24-72; follow-up until 36 months)**						
HPV-015: Cervarix vs. Al(OH)3 (f, 24-72; 36)	0	2882	2	2871	6.6%	0.20 [0.01, 4.15]
**Subtotal (95% CI)**		**2882**		**2871**	**6.6%**	**0.20 [0.01, 4.15]**
Total events	0		2			
Heterogeneity: Not applicable						
Test for overall effect: Z = 1.04 (P = 0.30)						
**13.1.2 Serious anaphylactic reaction: Cervarix vs. Havrix: hepatitis A vaccine (females, age 15-25; follow-up until 48 months)**						
HPV-008: Cervarix vs. Havrix (f, 15-25; 48)	0	9319	1	9325	5.9%	0.33 [0.01, 8.19]
**Subtotal (95% CI)**		**9319**		**9325**	**5.9%**	**0.33 [0.01, 8.19]**
Total events	0		1			
Heterogeneity: Not applicable						
Test for overall effect: Z = 0.67 (P = 0.50)						
**13.1.3 Serious anaphylactic reaction: Gardasil vs. AAHS (females, age 15-26; follow-up until 45 months)**						
V501-015: Gardasil vs. AAHS (f, 15-26; 36)	0	6087	1	6080	5.9%	0.33 [0.01, 8.17]
**Subtotal (95% CI)**		**6087**		**6080**	**5.9%**	**0.33 [0.01, 8.17]**
Total events	0		1			
Heterogeneity: Not applicable						
Test for overall effect: Z = 0.67 (P = 0.50)						
**13.1.4 Serious anaphylactic shock: Cervarix vs. Havrix: hepatitis A vaccine (females, age 15-25; follow-up until 48 months)**						
HPV-008: Cervarix vs. Havrix (f, 15-25; 48)	2	9319	0	9325	6.6%	5.00 [0.24, 104.20]
**Subtotal (95% CI)**		**9319**		**9325**	**6.6%**	**5.00 [0.24, 104.20]**
Total events	2		0			
Heterogeneity: Not applicable						
Test for overall effect: Z = 1.04 (P = 0.30)						
**13.1.5 New onset anaphylactic reaction ('medically significant conditions'): Cervarix vs. Al(OH)3 (females, age 24-72; follow-up until 36 months)**						
HPV-015: Cervarix vs. Al(OH)3 (f, 24-72; 36)	1	2882	2	2871	10.6%	0.50 [0.05, 5.49]
**Subtotal (95% CI)**		**2882**		**2871**	**10.6%**	**0.50 [0.05, 5.49]**
Total events	1		2			
Heterogeneity: Not applicable						
Test for overall effect: Z = 0.57 (P = 0.57)						
**13.1.6 New onset anaphylactic reaction ('medically significant conditions'): Cervarix vs. Havrix: hepatitis A vaccine (females, age 10-14; follow-up until 12 months)**						
HPV-013: Cervarix vs. Havrix (f, 10-14; 12)	0	1035	1	1032	5.9%	0.33 [0.01, 8.15]
**Subtotal (95% CI)**		**1035**		**1032**	**5.9%**	**0.33 [0.01, 8.15]**
Total events	0		1			
Heterogeneity: Not applicable						
Test for overall effect: Z = 0.67 (P = 0.50)						
**13.1.7 New onset anaphylactic reaction ('medically significant conditions'): Cervarix vs. Havrix: hepatitis A vaccine (females, age 15-25; follow-up until 48 months)**						
HPV-008: Cervarix vs. Havrix (f, 15-25; 48)	0	9319	2	9325	6.6%	0.20 [0.01, 4.17]
**Subtotal (95% CI)**		**9319**		**9325**	**6.6%**	**0.20 [0.01, 4.17]**
Total events	0		2			
Heterogeneity: Not applicable						
Test for overall effect: Z = 1.04 (P = 0.30)						
**13.1.8 New onset anaphylactic reaction ('new medical history'): Gardasil vs. carrier solution (females and males, age 9-16; follow-up until 18 months)**						
V501-018: Gardasil vs. carrier solu. (f&m,9-16;18)	1	1184	0	597	5.9%	1.51 [0.06, 37.11]
**Subtotal (95% CI)**		**1184**		**597**	**5.9%**	**1.51 [0.06, 37.11]**
Total events	1		0			
Heterogeneity: Not applicable						
Test for overall effect: Z = 0.25 (P = 0.80)						
**13.1.9 New onset anaphylactic reaction ('new medical history'): Gardasil vs. AAHS (females, age 15-26; follow-up until 36 months)**						
V501-015: Gardasil vs. AAHS (f, 15-26; 36)	4	6087	2	6080	21.1%	2.00 [0.37, 10.90]
**Subtotal (95% CI)**		**6087**		**6080**	**21.1%**	**2.00 [0.37, 10.90]**
Total events	4		2			
Heterogeneity: Not applicable						
Test for overall effect: Z = 0.80 (P = 0.42)						
**13.1.10 New onset anaphylactic shock ('medically significant conditions'): Cervarix vs. Havrix: hepatitis A vaccine (females, age 15-25; follow-up until 48 months)**						
HPV-008: Cervarix vs. Havrix (f, 15-25; 48)	3	9319	0	9325	6.9%	7.00 [0.36, 135.59]
**Subtotal (95% CI)**		**9319**		**9325**	**6.9%**	**7.00 [0.36, 135.59]**
Total events	3		0			
Heterogeneity: Not applicable						
Test for overall effect: Z = 1.29 (P = 0.20)						
**13.1.11 New onset anaphylactic shock ('new medical history'): Gardasil vs. AAHS (females, age 15-26; follow-up until 45 months)**						
V501-013: Gardasil vs. AAHS (f, 16-24; 45)	1	2723	0	2732	5.9%	3.01 [0.12, 73.85]
V501-015: Gardasil vs. AAHS (f, 15-26; 36)	1	6087	0	6080	5.9%	3.00 [0.12, 73.54]
**Subtotal (95% CI)**		**8810**		**8812**	**11.9%**	**3.00 [0.31, 28.87]**
Total events	2		0			
Heterogeneity: $Tau^2 = 0.00$; $Chi^2 = 0.00$, df = 1 (P = 1.00); $I^2 = 0\%$						
Test for overall effect: Z = 0.95 (P = 0.34)						
**13.1.12 New onset anaphylactic shock ('new medical history'): Gardasil vs. AAHS (females, age 21-46; follow-up until 48 months)**						
V501-019: Gardasil vs. AAHS (f, 21-46; 48)	0	1911	1	1908	5.9%	0.33 [0.01, 8.16]
**Subtotal (95% CI)**		**1911**		**1908**	**5.9%**	**0.33 [0.01, 8.16]**
Total events	0		1			
Heterogeneity: Not applicable						
Test for overall effect: Z = 0.67 (P = 0.50)						

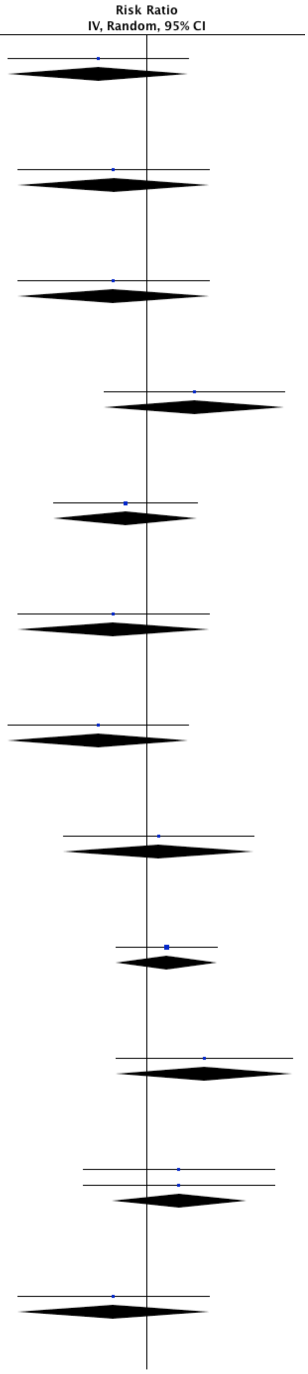

*13.1. Risk ratio for serious anaphylactic shock or reaction: 0.59 [0.13, 2.82]. Risk ratio for new onset anaphylactic shock or reaction: 1.18 [0.48, 2.91].

13.2. Chronic fatigue syndrome (CFS): intention to treat analysis
No cases/data/reports.

13.3. Chronic regional pain syndrome (CRPS): intention to treat analysis
No cases/data/reports.

13.4. Guillain–Barré syndrome (GBS): intention to treat analysis
No cases/data/reports.

13.5. Premature ovarian failure (POF): intention to treat analysis

Study or Subgroup	HPV vaccine Events	HPV vaccine Total	Control Events	Control Total	Weight	Risk Ratio IV, Random, 95% CI
**13.5.1 New onset ovarian failure ('new medical history'): Gardasil vs. AAHS (females, age 21-46; follow-up until 48 months)**						
V501-019: Gardasil vs. AAHS (f, 21-46; 48)	1	1911	0	1908	100.0%	3.00 [0.12, 73.48]
**Subtotal (95% CI)**		**1911**		**1908**	**100.0%**	**3.00 [0.12, 73.48]**
Total events	1		0			
Heterogeneity: Not applicable						
Test for overall effect: Z = 0.67 (P = 0.50)						
**Total (95% CI)**		**1911**		**1908**	**100.0%**	**3.00 [0.12, 73.48]**
Total events	1		0			
Heterogeneity: Not applicable						
Test for overall effect: Z = 0.67 (P = 0.50)						
Test for subgroup differences: Not applicable						

Risk Ratio
IV, Random, 95% CI

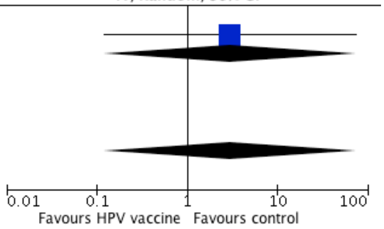

*11.7. Risk ratio for 'medically significant conditions' (GlaxoSmithKline): not applicable; risk ratio for 'new medical history' (Merck Sharp & Dohme): 3.00 [0.12, 73.48]

13.6. Postural orthostatic tachycardia syndrome (POTS): intention to treat analysis

No cases/data/reports.

13.7. Syncope: intention to treat analysis

Study or Subgroup	HPV vaccine Events	HPV vaccine Total	Control Events	Control Total	Weight	Risk Ratio IV, Random, 95% CI
**13.7.1 Serious syncope: Cervarix vs. Engerix: hepatitis B vaccine (females and males, age 9-16; follow-up until 72 months)**						
HPV-040: Cervarix vs. Engerix (f & m, 12-16; 72)	2	2436	0	1267	1.5%	2.60 [0.12, 54.15]
**Subtotal (95% CI)**		**2436**		**1267**	**1.5%**	**2.60 [0.12, 54.15]**
Total events	2		0			
Heterogeneity: Not applicable						
Test for overall effect: Z = 0.62 (P = 0.54)						
**13.7.2 Serious syncope: Cervarix vs. Havrix: hepatitis A vaccine (females, age 15-25; follow-up until 48 months)**						
HPV-008: Cervarix vs. Havrix (f, 15-25; 48)	0	9319	2	9325	1.5%	0.20 [0.01, 4.17]
**Subtotal (95% CI)**		**9319**		**9325**	**1.5%**	**0.20 [0.01, 4.17]**
Total events	0		2			
Heterogeneity: Not applicable						
Test for overall effect: Z = 1.04 (P = 0.30)						
**13.7.3 Serious syncope: Gardasil vs. AAHS (females, age 15-26; follow-up until 45 months)**						
V501-013: Gardasil vs. AAHS (f, 16-24; 45)	0	2723	1	2732	1.4%	0.33 [0.01, 8.21]
**Subtotal (95% CI)**		**2723**		**2732**	**1.4%**	**0.33 [0.01, 8.21]**
Total events	0		1			
Heterogeneity: Not applicable						
Test for overall effect: Z = 0.67 (P = 0.50)						
**13.7.4 Serious syncope: Gardasil 9 vs. saline placebo (females, age 12-26; follow-up until 7 months)**						
V503-006: Gardasil 9 vs. saline (f, 12-26;7)	1	618	0	306	1.4%	1.49 [0.06, 36.42]
**Subtotal (95% CI)**		**618**		**306**	**1.4%**	**1.49 [0.06, 36.42]**
Total events	1		0			
Heterogeneity: Not applicable						
Test for overall effect: Z = 0.24 (P = 0.81)						
**13.7.5 Serious syncope: HPV 16 vaccine vs. AAHS (females, age 16-25; follow-up until 48 months)**						
V501-005: HPV 16 vaccine vs. AAHS (f, 16-25; 48)	1	1204	0	1205	1.4%	3.00 [0.12, 73.63]
**Subtotal (95% CI)**		**1204**		**1205**	**1.4%**	**3.00 [0.12, 73.63]**
Total events	1		0			
Heterogeneity: Not applicable						
Test for overall effect: Z = 0.67 (P = 0.50)						
**13.7.6 New onset syncope ('medically significant conditions'): Cervarix vs. Al(OH)3 (females, age 24-72; follow-up until 36 months)**						
HPV-015: Cervarix vs. Al(OH)3 (f, 24-72; 36)	4	2882	2	2871	4.7%	1.99 [0.37, 10.87]
**Subtotal (95% CI)**		**2882**		**2871**	**4.7%**	**1.99 [0.37, 10.87]**
Total events	4		2			
Heterogeneity: Not applicable						
Test for overall effect: Z = 0.80 (P = 0.43)						
**13.7.7 New onset syncope ('medically significant conditions'): Cervarix vs. Havrix: hepatitis A vaccine (females, age 10-14; follow-up until 12 months)**						
HPV-013: Cervarix vs. Havrix (f, 10-14; 12)	3	1035	2	1032	4.2%	1.50 [0.25, 8.93]
**Subtotal (95% CI)**		**1035**		**1032**	**4.2%**	**1.50 [0.25, 8.93]**
Total events	3		2			
Heterogeneity: Not applicable						
Test for overall effect: Z = 0.44 (P = 0.66)						
**13.7.8 New onset syncope ('medically significant conditions'): Cervarix vs. Havrix: hepatitis A vaccine (females, age 15-25; follow-up until 48 months)**						
HPV-008: Cervarix vs. Havrix (f, 15-25; 48)	12	9319	10	9325	16.7%	1.20 [0.52, 2.78]
**Subtotal (95% CI)**		**9319**		**9325**	**16.7%**	**1.20 [0.52, 2.78]**
Total events	12		10			
Heterogeneity: Not applicable						
Test for overall effect: Z = 0.43 (P = 0.67)						
**13.7.9 New onset syncope ('new medical history'): Gardasil vs. carrier solution (females and males, age 9-16; follow-up until 18 months)**						
V501-018: Gardasil vs. carrier solu. (f&m,9-16;18)	4	1184	3	597	6.0%	0.67 [0.15, 2.99]
**Subtotal (95% CI)**		**1184**		**597**	**6.0%**	**0.67 [0.15, 2.99]**
Total events	4		3			
Heterogeneity: Not applicable						
Test for overall effect: Z = 0.52 (P = 0.60)						
**13.7.10 New onset syncope ('new medical history'): Gardasil vs. AAHS (females, age 15-26; follow-up until 45 months)**						
V501-013: Gardasil vs. AAHS (f, 16-24; 45)	19	2723	6	2732	14.4%	3.18 [1.27, 7.94]
V501-015: Gardasil vs. AAHS (f, 15-26; 36)	18	6087	30	6080	29.1%	0.60 [0.33, 1.07]
**Subtotal (95% CI)**		**8810**		**8812**	**43.5%**	**1.33 [0.26, 6.79]**
Total events	37		36			
Heterogeneity: $Tau^2 = 1.24$; $Chi^2 = 9.06$, df = 1 (P = 0.003); $I^2 = 89\%$						
Test for overall effect: Z = 0.34 (P = 0.73)						
**13.7.11 New onset syncope ('new medical history'): Gardasil vs. AAHS (females, age 21-46; follow-up until 48 months)**						
V501-019: Gardasil vs. AAHS (f, 21-46; 48)	2	1911	6	1908	5.2%	0.33 [0.07, 1.65]
**Subtotal (95% CI)**		**1911**		**1908**	**5.2%**	**0.33 [0.07, 1.65]**
Total events	2		6			
Heterogeneity: Not applicable						
Test for overall effect: Z = 1.35 (P = 0.18)						
**13.7.12 New onset syncope ('new medical history'): Gardasil vs. AAHS (males, age 15-27; follow-up until 36 months)**						
V501-020: Gardasil vs. AAHS (m, 15-27; 36)	0	2032	1	2033	1.4%	0.33 [0.01, 8.18]
**Subtotal (95% CI)**		**2032**		**2033**	**1.4%**	**0.33 [0.01, 8.18]**
Total events	0		1			
Heterogeneity: Not applicable						
Test for overall effect: Z = 0.67 (P = 0.50)						
**13.7.13 General syncope ('unsolicited'): Cervarix vs. Engerix: hepatitis B vaccine (females and males, age 9-16; follow-up until 72 months)**						
HPV-030: Cervarix vs. Engerix (f, 9-15; 12)	1	247	3	246	2.7%	0.33 [0.03, 3.17]
HPV-040: Cervarix vs. Engerix (f & m, 12-16; 72)	1	1908	0	3128	1.4%	4.92 [0.20, 120.64]
**Subtotal (95% CI)**		**2155**		**3374**	**4.1%**	**1.00 [0.07, 13.36]**
Total events	2		3			
Heterogeneity: $Tau^2 = 1.64$; $Chi^2 = 1.82$, df = 1 (P = 0.18); $I^2 = 45\%$						
Test for overall effect: Z = 0.00 (P = 1.00)						
**13.7.14 General syncope ('unsolicited'): Cervarix vs. Twinrix: hepatitis A and B vaccine (females, age 8-15; follow-up until 12 months)**						
HPV-029: Cervarix vs. Twinrix (f, 8-15; 12)	1	270	1	271	1.8%	1.00 [0.06, 15.96]
**Subtotal (95% CI)**		**270**		**271**	**1.8%**	**1.00 [0.06, 15.96]**
Total events	1		1			
Heterogeneity: Not applicable						
Test for overall effect: Z = 0.00 (P = 1.00)						
**13.7.15 General syncope ('systemic adverse events'): Gardasil vs. carrier solution (females and males, age 9-16; follow-up until 18 months)**						
V501-018: Gardasil vs. carrier solu. (f&m,9-16;18)	0	1184	1	597	1.4%	0.17 [0.01, 4.12]
**Subtotal (95% CI)**		**1184**		**597**	**1.4%**	**0.17 [0.01, 4.12]**
Total events	0		1			
Heterogeneity: Not applicable						
Test for overall effect: Z = 1.09 (P = 0.27)						
**13.7.16 General syncope ('systemic adverse events'): Gardasil vs. AAHS (males, age 15-27; follow-up until 36 months)**						
V501-020: Gardasil vs. AAHS (m, 15-27; 36)	1	2032	2	2033	2.4%	0.50 [0.05, 5.51]
**Subtotal (95% CI)**		**2032**		**2033**	**2.4%**	**0.50 [0.05, 5.51]**
Total events	1		2			
Heterogeneity: Not applicable						
Test for overall effect: Z = 0.57 (P = 0.57)						
**13.7.17 General syncope ('systemic adverse events'): Gardasil 9 vs. saline placebo (females, age 12-26; follow-up until 7 months)**						
V503-006: Gardasil 9 vs. saline (f, 12-26;7)	3	618	0	306	1.6%	3.47 [0.18, 67.00]
**Subtotal (95% CI)**		**618**		**306**	**1.6%**	**3.47 [0.18, 67.00]**
Total events	3		0			
Heterogeneity: Not applicable						
Test for overall effect: Z = 0.82 (P = 0.41)						

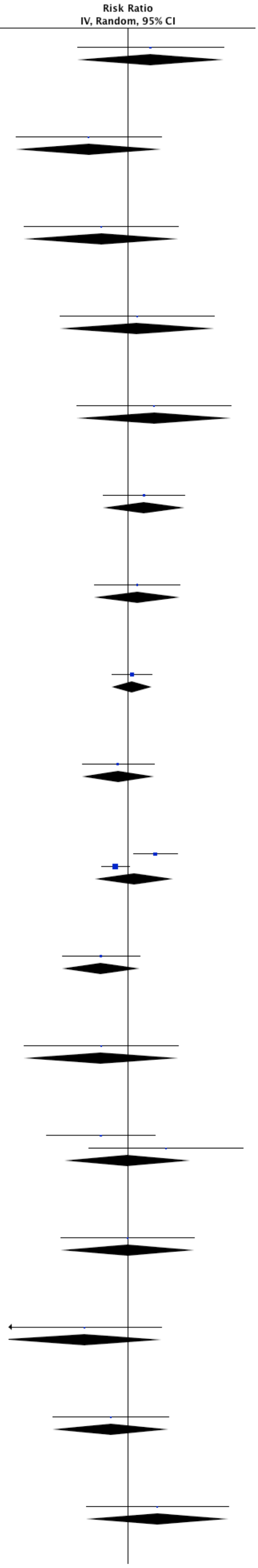

*13.7. Risk ratio for serious syncope: 0.94 [0.23, 3.81]. Risk ratio for new onset syncope: 1.03 [0.58, 1.84]. Risk ratio for general syncope: 0.77 [0.25, 2.34].

14. Post hoc exploratory harm analyses

14.1. Serious harms judged as 'definitely associated'* with chronic regional pain syndrome (CRPS): intention to treat analysis

Study or Subgroup	HPV vaccine Events	HPV vaccine Total	Control Events	Control Total	Weight	Risk Ratio IV, Random, 95% CI
**14.1.1 Serious harms 'definitely associated' with CRPS: Cervarix vs. Al(OH)3 (females, age 18-35; follow-up until 7 months)**						
HPV-035: Cervarix vs. Al(OH)3 (f, 18-35; 7)	4	150	0	150	1.3%	9.00 [0.49, 165.71]
**Subtotal (95% CI)**		**150**		**150**	**1.3%**	**9.00 [0.49, 165.71]**
Total events	4		0			
Heterogeneity: Not applicable						
Test for overall effect: Z = 1.48 (P = 0.14)						
**14.1.2 Serious harms 'definitely associated' with CRPS: Cervarix vs. Al(OH)3 (females, age 24-72; follow-up until 36 months)**						
HPV-015: Cervarix vs. Al(OH)3 (f, 24-72; 36)	15	2882	9	2871	15.9%	1.66 [0.73, 3.79]
**Subtotal (95% CI)**		**2882**		**2871**	**15.9%**	**1.66 [0.73, 3.79]**
Total events	15		9			
Heterogeneity: Not applicable						
Test for overall effect: Z = 1.20 (P = 0.23)						
**14.1.3 Serious harms 'definitely associated' with CRPS: Cervarix vs. Aimmugen: hepatitis A vaccine (females, age 22-29; follow-up until 12 months)**						
HPV-063: Cervarix vs. Aimmugen (f, 22-29; 12)	0	375	1	377	1.1%	0.34 [0.01, 8.20]
**Subtotal (95% CI)**		**375**		**377**	**1.1%**	**0.34 [0.01, 8.20]**
Total events	0		1			
Heterogeneity: Not applicable						
Test for overall effect: Z = 0.67 (P = 0.50)						
**14.1.4 Serious harms 'definitely associated' with CRPS: Cervarix vs. Engerix: hepatitis B vaccine (females and males, age 9-16; follow-up until 72 months)**						
HPV-040: Cervarix vs. Engerix (f & m, 12-16; 72)	3	2436	0	1267	1.2%	3.64 [0.19, 70.46]
**Subtotal (95% CI)**		**2436**		**1267**	**1.2%**	**3.64 [0.19, 70.46]**
Total events	3		0			
Heterogeneity: Not applicable						
Test for overall effect: Z = 0.86 (P = 0.39)						
**14.1.5 Serious harms 'definitely associated' with CRPS: Cervarix vs. Havrix: hepatitis A vaccine (females, age 10-14; follow-up until 12 months)**						
HPV-013: Cervarix vs. Havrix (f, 10-14; 12)	2	1035	2	1032	2.8%	1.00 [0.14, 7.07]
**Subtotal (95% CI)**		**1035**		**1032**	**2.8%**	**1.00 [0.14, 7.07]**
Total events	2		2			
Heterogeneity: Not applicable						
Test for overall effect: Z = 0.00 (P = 1.00)						
**14.1.6 Serious harms 'definitely associated' with CRPS: Cervarix vs. Havrix: hepatitis A vaccine (females, age 15-25; follow-up until 48 months)**						
HPV-008: Cervarix vs. Havrix (f, 15-25; 48)	59	9319	39	9325	66.3%	1.51 [1.01, 2.27]
**Subtotal (95% CI)**		**9319**		**9325**	**66.3%**	**1.51 [1.01, 2.27]**
Total events	59		39			
Heterogeneity: Not applicable						
Test for overall effect: Z = 2.01 (P = 0.04)						
**14.1.7 Serious harms 'definitely associated' with CRPS: Gardasil vs. carrier solution (females and males, age 9-16; follow-up until 18 months)**						
V501-018: Gardasil vs. carrier solu. (f&m,9-16;18)	1	1184	0	597	1.1%	1.51 [0.06, 37.11]
**Subtotal (95% CI)**		**1184**		**597**	**1.1%**	**1.51 [0.06, 37.11]**
Total events	1		0			
Heterogeneity: Not applicable						
Test for overall effect: Z = 0.25 (P = 0.80)						
**14.1.8 Serious harms 'definitely associated' with CRPS: Gardasil vs. AAHS (females, age 15-26; follow-up until 45 months)**						
V501-013: Gardasil vs. AAHS (f, 16-24; 45)	1	2723	2	2732	1.9%	0.50 [0.05, 5.53]
V501-015: Gardasil vs. AAHS (f, 15-26; 36)	4	6087	3	6080	4.8%	1.33 [0.30, 5.95]
**Subtotal (95% CI)**		**8810**		**8812**	**6.7%**	**1.01 [0.28, 3.61]**
Total events	5		5			
Heterogeneity: Tau² = 0.00; Chi² = 0.46, df = 1 (P = 0.50); I² = 0%						
Test for overall effect: Z = 0.02 (P = 0.98)						
**14.1.9 Serious harms 'definitely associated' with CRPS: Gardasil vs. AAHS (females, age 21-46; follow-up until 48 months)**						
V501-019: Gardasil vs. AAHS (f, 21-46; 48)	4	1911	0	1908	1.3%	8.99 [0.48, 166.79]
**Subtotal (95% CI)**		**1911**		**1908**	**1.3%**	**8.99 [0.48, 166.79]**
Total events	4		0			
Heterogeneity: Not applicable						
Test for overall effect: Z = 1.47 (P = 0.14)						
**14.1.10 Serious harms 'definitely associated' with CRPS: Gardasil vs. AAHS (males, age 15-27; follow-up until 36 months)**						
V501-020: Gardasil vs. AAHS (m, 15-27; 36)	1	2032	0	2033	1.1%	3.00 [0.12, 73.64]
**Subtotal (95% CI)**		**2032**		**2033**	**1.1%**	**3.00 [0.12, 73.64]**
Total events	1		0			
Heterogeneity: Not applicable						
Test for overall effect: Z = 0.67 (P = 0.50)						
**14.1.11 Serious harms 'definitely associated' with CRPS: HPV 16 vaccine vs. AAHS (females, age 16-25; follow-up until 48 months)**						
V501-005: HPV 16 vaccine vs. AAHS (f, 16-25; 48)	1	1204	1	1205	1.4%	1.00 [0.06, 15.98]
**Subtotal (95% CI)**		**1204**		**1205**	**1.4%**	**1.00 [0.06, 15.98]**
Total events	1		1			
Heterogeneity: Not applicable						
Test for overall effect: Z = 0.00 (P = 1.00)						
**Total (95% CI)**		**31338**		**29577**	**100.0%**	**1.54 [1.11, 2.14]**
Total events	95		57			
Heterogeneity: Tau² = 0.00; Chi² = 5.37, df = 11 (P = 0.91); I² = 0%						
Test for overall effect: Z = 2.58 (P = 0.010)						
Test for subgroup differences: Chi² = 4.92, df = 10 (P = 0.90), I² = 0%						

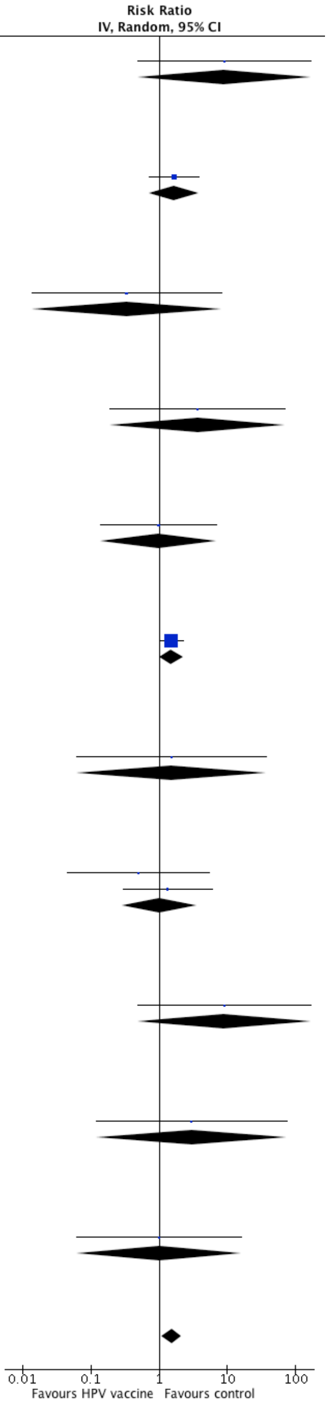

*14.1. Risk ratio for GlaxoSmithKline studies (i.e., HPV-0xx): **1.55 [1.09, 2.20]**; risk ratio for Merck Sharp & Dohme studies (i.e., V50x-xxx): 1.47 [0.56, 3.89]. We asked a physician with clinical expertise in CRPS to assess the reported MedDRA preferred terms as 'definitely,' 'probably,' 'probably not' or 'definitely not' associated with the syndromes. We sent an Excel sheet to the physician with all the reported MedDRA terms. The physician was blinded, as the Excel sheet contained no outcome data. When the physician had assessed all the MedDRA terms, we synthesized the data for those MedDRA terms that the physician judged 'definitely' associated with CRPS and compared it to the reported serious harms.

- Reported MedDRA terms and number of harms per MedDRA term for the serious harms that were judged ‘definitely associated’ with chronic regional pain syndrome (CRPS):

**Physician judgment**	**MedDRA system organ class**	**MedDRA preferred term**	**HPV vaccine**	**Comparator**
‘Definitely associated with CRPS’	Cardiac disorders (10007541)	Arrhythmia (10003119))	2	0
‘Definitely associated with CRPS’	Cardiac disorders (10007541)	Sinus tachycardia (10040752)	2	1
‘Definitely associated with CRPS’	Cardiac disorders (10007541)	Supraventricular tachycardia (10042604)	3	1
‘Definitely associated with CRPS’	Cardiac disorders (10007541)	Tachycardia (10043071)	1	0
‘Definitely associated with CRPS’	Ear and labyrinth disorders (10013993)	Vertigo (10047340)	3	2
‘Definitely associated with CRPS’	Eye disorders (10015919)	Vision blurred (10047513)	1	0
‘Definitely associated with CRPS’	Gastrointestinal disorders (10017947)	Abdominal pain (10000081)	25	18
‘Definitely associated with CRPS’	Gastrointestinal disorders (10017947)	Abdominal pain lower (10000084)	5	5
‘Definitely associated with CRPS’	Gastrointestinal disorders (10017947)	Abdominal pain upper (10000087)	4	5
‘Definitely associated with CRPS’	Gastrointestinal disorders (10017947)	Constipation (10010774)	2	2
‘Definitely associated with CRPS’	Gastrointestinal disorders (10017947)	Diarrhoea (10012735)	4	1
‘Definitely associated with CRPS’	Gastrointestinal disorders (10017947)	Dyspepsia (10013946)	1	2
‘Definitely associated with CRPS’	Gastrointestinal disorders (10017947)	Irritable bowel syndrome (10023003)	4	1
‘Definitely associated with CRPS’	Gastrointestinal disorders (10017947)	Nausea (10028813)	2	1
‘Definitely associated with CRPS’	Gastrointestinal disorders (10017947)	Vomiting (10047700)	3	2
‘Definitely associated with CRPS’	General disorders and administration site conditions (10018065)	Fatigue (10016256)	1	1
‘Definitely associated with CRPS’	General disorders and administration site conditions (10018065)	Non-cardiac chest pain	1	0
‘Definitely associated with CRPS’	Musculoskeletal and connective tissue disorders (10028395)	Back pain (10003988)	9	5
‘Definitely associated with CRPS’	Musculoskeletal and connective tissue disorders (10028395)	Musculoskeletal pain (10028391)	0	1
‘Definitely associated with CRPS’	Musculoskeletal and connective tissue disorders (10028395)	Pain in extremity (10033425)	2	1
‘Definitely associated with CRPS’	Nervous system disorders (10029205)	Dizziness (10013573)	5	2
‘Definitely associated with CRPS’	Nervous system disorders (10029205)	Headache (10019211)	11	4
‘Definitely associated with CRPS’	Nervous system disorders (10029205)	Neuropathy peripheral (10029331)	0	1
‘Definitely associated with CRPS’	Nervous system disorders (10029205)	Paraesthesia (10033775)	1	1
‘Definitely associated with CRPS’	Nervous system disorders (10029205)	Tension headache (10043269)	3	0
**Total**			**95**	**57**

14.2. Serious harms judged as ‘definitely associated’* with postural orthostatic tachycardia syndrome (POTS): intention to treat analysis

Study or Subgroup	HPV vaccine Events	HPV vaccine Total	Control Events	Control Total	Weight	Risk Ratio IV, Random, 95% CI
**14.2.1 Serious harms 'definitely associated' with POTS: Cervarix vs. Al(OH)3 (females, age 18–35; follow-up until 7 months)**						
HPV-035: Cervarix vs. Al(OH)3 (f, 18–35; 7)	3	150	0	150	2.5%	7.00 [0.36, 134.36]
**Subtotal (95% CI)**		**150**		**150**	**2.5%**	**7.00 [0.36, 134.36]**
Total events	3		0			
Heterogeneity: Not applicable						
Test for overall effect: Z = 1.29 (P = 0.20)						
**14.2.2 Serious harms 'definitely associated' with POTS: Cervarix vs. Al(OH)3 (females, age 24–72; follow-up until 36 months)**						
HPV-015: Cervarix vs. Al(OH)3 (f, 24–72; 36)	9	2882	5	2871	18.3%	1.79 [0.60, 5.34]
**Subtotal (95% CI)**		**2882**		**2871**	**18.3%**	**1.79 [0.60, 5.34]**
Total events	9		5			
Heterogeneity: Not applicable						
Test for overall effect: Z = 1.05 (P = 0.29)						
**14.2.3 Serious harms 'definitely associated' with POTS: Cervarix vs. Aimmugen: hepatitis A vaccine (females, age 22–29; follow-up until 12 months)**						
HPV-063: Cervarix vs. Aimmugen (f, 22–29; 12)	0	375	1	377	2.1%	0.34 [0.01, 8.20]
**Subtotal (95% CI)**		**375**		**377**	**2.1%**	**0.34 [0.01, 8.20]**
Total events	0		1			
Heterogeneity: Not applicable						
Test for overall effect: Z = 0.67 (P = 0.50)						
**14.2.4 Serious harms 'definitely associated' with POTS: Cervarix vs. Engerix: hepatitis B vaccine (females and males, age 9–16; follow-up until 72 months)**						
HPV-040: Cervarix vs. Engerix (f & m, 12–16; 72)	3	2436	1	1267	4.3%	1.56 [0.16, 14.99]
**Subtotal (95% CI)**		**2436**		**1267**	**4.3%**	**1.56 [0.16, 14.99]**
Total events	3		1			
Heterogeneity: Not applicable						
Test for overall effect: Z = 0.39 (P = 0.70)						
**14.2.5 Serious harms 'definitely associated' with POTS: Cervarix vs. Havrix: hepatitis A vaccine (females, age 10–14; follow-up until 12 months)**						
HPV-013: Cervarix vs. Havrix (f, 10–14; 12)	0	1035	2	1032	2.4%	0.20 [0.01, 4.15]
**Subtotal (95% CI)**		**1035**		**1032**	**2.4%**	**0.20 [0.01, 4.15]**
Total events	0		2			
Heterogeneity: Not applicable						
Test for overall effect: Z = 1.04 (P = 0.30)						
**14.2.6 Serious harms 'definitely associated' with POTS: Cervarix vs. Havrix: hepatitis A vaccine (females, age 15–25; follow-up until 48 months)**						
HPV-008: Cervarix vs. Havrix (f, 15–25; 48)	28	9319	12	9325	47.9%	2.33 [1.19, 4.59]
**Subtotal (95% CI)**		**9319**		**9325**	**47.9%**	**2.33 [1.19, 4.59]**
Total events	28		12			
Heterogeneity: Not applicable						
Test for overall effect: Z = 2.46 (P = 0.01)						
**14.2.7 Serious harms 'definitely associated' with POTS: Gardasil vs. AAHS (females, age 15–26; follow-up until 45 months)**						
V501-013: Gardasil vs. AAHS (f, 16–24; 45)	3	2723	3	2732	8.5%	1.00 [0.20, 4.97]
V501-015: Gardasil vs. AAHS (f, 15–26; 36)	3	6087	1	6080	4.3%	3.00 [0.31, 28.80]
**Subtotal (95% CI)**		**8810**		**8812**	**12.8%**	**1.44 [0.39, 5.33]**
Total events	6		4			
Heterogeneity: $Tau^2 = 0.00$; $Chi^2 = 0.60$, df = 1 (P = 0.44); $I^2 = 0\%$						
Test for overall effect: Z = 0.55 (P = 0.58)						
**14.2.8 Serious harms 'definitely associated' with POTS: Gardasil vs. AAHS (females, age 21–46; follow-up until 48 months)**						
V501-019: Gardasil vs. AAHS (f, 21–46; 48)	4	1911	0	1908	2.6%	8.99 [0.48, 166.79]
**Subtotal (95% CI)**		**1911**		**1908**	**2.6%**	**8.99 [0.48, 166.79]**
Total events	4		0			
Heterogeneity: Not applicable						
Test for overall effect: Z = 1.47 (P = 0.14)						
**14.2.9 Serious harms 'definitely associated' with POTS: Gardasil vs. AAHS (males, age 15–27; follow-up until 36 months)**						
V501-020: Gardasil vs. AAHS (m, 15–27; 36)	1	2032	0	2033	2.1%	3.00 [0.12, 73.64]
**Subtotal (95% CI)**		**2032**		**2033**	**2.1%**	**3.00 [0.12, 73.64]**
Total events	1		0			
Heterogeneity: Not applicable						
Test for overall effect: Z = 0.67 (P = 0.50)						
**14.2.10 Serious harms 'definitely associated' with POTS: Gardasil 9 vs. saline placebo (females, age 12–26; follow-up until 7 months)**						
V503-006: Gardasil 9 vs. saline (f, 12–26;7)	1	618	0	306	2.1%	1.49 [0.06, 36.42]
**Subtotal (95% CI)**		**618**		**306**	**2.1%**	**1.49 [0.06, 36.42]**
Total events	1		0			
Heterogeneity: Not applicable						
Test for overall effect: Z = 0.24 (P = 0.81)						
**14.2.11 Serious harms 'definitely associated' with POTS: HPV 16 vaccine vs. AAHS (females, age 16–25; follow-up until 48 months)**						
V501-005: HPV 16 vaccine vs. AAHS (f, 16–25; 48)	1	1204	1	1205	2.8%	1.00 [0.06, 15.98]
**Subtotal (95% CI)**		**1204**		**1205**	**2.8%**	**1.00 [0.06, 15.98]**
Total events	1		1			
Heterogeneity: Not applicable						
Test for overall effect: Z = 0.00 (P = 1.00)						
**Total (95% CI)**		**30772**		**29286**	**100.0%**	**1.92 [1.21, 3.07]**
Total events	56		26			
Heterogeneity: $Tau^2 = 0.00$; $Chi^2 = 6.55$, df = 11 (P = 0.83); $I^2 = 0\%$						
Test for overall effect: Z = 2.74 (P = 0.006)						
Test for subgroup differences: $Chi^2 = 5.96$, df = 10 (P = 0.82), $I^2 = 0\%$						

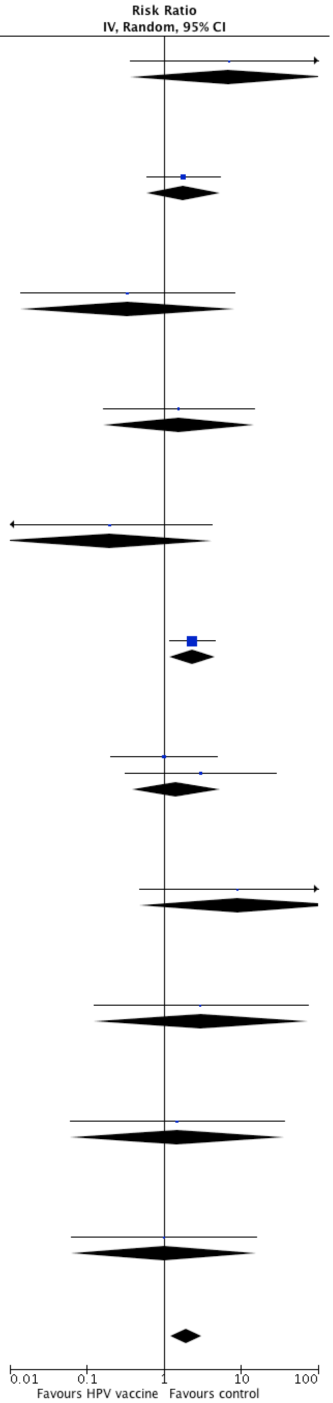

*14.2. Risk ratio for GlaxoSmithKline studies (i.e., HPV-0xx): **1.95 [1.15, 3.32]**; risk ratio for Merck Sharp & Dohme studies (i.e., V50x-xxx): 1.82 [0.68, 4.89]. We asked a physician with clinical expertise in POTS to assess the reported MedDRA preferred terms as 'definitely,' 'probably,' 'probably not' or ‘definitely not’ associated with the syndromes. We sent an Excel sheet to the physician with all the reported MedDRA terms. The physician was blinded, as the Excel sheet contained no outcome data. When the physician had assessed all the MedDRA terms, we synthesized the data for those MedDRA terms that the physician judged ‘definitely’ associated with POTS and compared it to the reported serious harms.

- Reported MedDRA terms and number of harms per MedDRA term for the serious harms that were judged 'definitely associated' with postural orthostatic tachycardia syndrome (POTS):

Physician judgment	MedDRA system organ class	MedDRA preferred term	HPV vaccine	Comparator
'Definitely associated with POTS'	Cardiac disorders (10007541)	Sinus tachycardia (10040752)	2	1
'Definitely associated with POTS'	Cardiac disorders (10007541)	Supraventricular tachycardia	3	1
'Definitely associated with POTS'	Cardiac disorders (10007541)	Tachycardia (10043071)	1	0
'Definitely associated with POTS'	Ear and labyrinth disorders (10013993)	Vertigo (10047340)	3	2
'Definitely associated with POTS'	Ear and labyrinth disorders (10013993)	Vertigo positional	0	1
'Definitely associated with POTS'	Gastrointestinal disorders (10017947)	Vomiting (10047700)	1	0
'Definitely associated with POTS'	Gastrointestinal disorders (10017947)	Constipation (10010774)	2	2
'Definitely associated with POTS'	Gastrointestinal disorders (10017947)	Diarrhoea (10012735)	4	1
'Definitely associated with POTS'	Gastrointestinal disorders (10017947)	Dyspepsia (10013946)	1	2
'Definitely associated with POTS'	Gastrointestinal disorders (10017947)	Irritable bowel syndrome	4	1
'Definitely associated with POTS'	Gastrointestinal disorders (10017947)	Nausea (10028813)	2	1
'Definitely associated with POTS'	Gastrointestinal disorders (10017947)	Vomiting (10047700)	3	2
'Definitely associated with POTS'	General disorders and administration site conditions (10018065)	Fatigue (10016256)	1	1
'Definitely associated with POTS'	General disorders and administration site conditions (10018065)	Non-cardiac chest pain	1	0
'Definitely associated with POTS'	Nervous system disorders (10029205)	Dizziness (10013573)	5	2
'Definitely associated with POTS'	Nervous system disorders (10029205)	Headache (10019211)	11	4
'Definitely associated with POTS'	Nervous system disorders (10029205)	Syncope (10042772)	4	3
'Definitely associated with POTS'	Nervous system disorders (10029205)	Tension headache (10043269)	3	0
'Definitely associated with POTS'	Respiratory, thoracic and mediastinal disorders (10038738)	Dyspnoea (10013968)	2	1
'Definitely associated with POTS'	Respiratory, thoracic and mediastinal disorders (10038738)	Hyperventilation (10020910)	2	0
'Definitely associated with POTS'	Vascular disorders (10047065)	Hypotension (10021097)	1	1
**Total**			**56**	**26**

14.3. New onset diseases ('medically significant conditions' and 'new medical history'*) judged as ‘definitely associated’** with chronic regional pain syndrome (CRPS): intention to treat analysis

Study or Subgroup	HPV vaccine Events	HPV vaccine Total	Control Events	Control Total	Weight	Risk Ratio IV, Random, 95% CI
**14.3.1 New onset diseases 'definitely associated' with CRPS ('medically significant conditions'): Cervarix vs. Al(OH)3 (females, age 9-17; follow-up until 12 months)**						
HPV-058: Cervarix vs. Al(OH)3 (f, 9-17; 12)	4	374	1	376	0.1%	4.02 [0.45, 35.81]
**Subtotal (95% CI)**		**374**		**376**	**0.1%**	**4.02 [0.45, 35.81]**
Total events	4		1			
Heterogeneity: Not applicable						
Test for overall effect: Z = 1.25 (P = 0.21)						
**14.3.2 New onset diseases 'definitely associated' with CRPS ('medically significant conditions'): Cervarix vs. Al(OH)3 (females, age 15-25; follow-up until 7 months)**						
HPV-038: Cervarix vs. Al(OH)3 (f, 15-25; 7)	7	149	2	76	0.1%	1.79 [0.38, 8.39]
**Subtotal (95% CI)**		**149**		**76**	**0.1%**	**1.79 [0.38, 8.39]**
Total events	7		2			
Heterogeneity: Not applicable						
Test for overall effect: Z = 0.73 (P = 0.46)						
**14.3.3 New onset diseases 'definitely associated' with CRPS ('medically significant conditions'): Cervarix vs. Al(OH)3 (females, age 18-35; follow-up until 7 months)**						
HPV-031: Cervarix vs. Al(OH)3 (f, 18-35; 7)	5	176	5	178	0.2%	1.01 [0.30, 3.43]
HPV-035: Cervarix vs. Al(OH)3 (f, 18-35; 7)	16	150	4	150	0.2%	4.00 [1.37, 11.69]
**Subtotal (95% CI)**		**326**		**328**	**0.4%**	**2.08 [0.54, 7.98]**
Total events	21		9			
Heterogeneity: $Tau^2 = 0.60$; $Chi^2 = 2.75$, df = 1 (P = 0.10); $I^2 = 64\%$						
Test for overall effect: Z = 1.06 (P = 0.29)						
**14.3.4 New onset diseases 'definitely associated' with CRPS ('medically significant conditions'): Cervarix vs. Al(OH)3 (females, age 24-72; follow-up until 36 months)**						
HPV-015: Cervarix vs. Al(OH)3 (f, 24-72; 36)	317	2882	260	2871	8.3%	1.21 [1.04, 1.42]
**Subtotal (95% CI)**		**2882**		**2871**	**8.3%**	**1.21 [1.04, 1.42]**
Total events	317		260			
Heterogeneity: Not applicable						
Test for overall effect: Z = 2.45 (P = 0.01)						
**14.3.5 New onset diseases 'definitely associated' with CRPS ('medically significant conditions'): Cervarix vs. Aimmugen: hepatitis A vaccine (females, age 22-29; follow-up until 12 months)**						
HPV-063: Cervarix vs. Aimmugen (f, 22-29; 12)	10	375	16	377	0.5%	0.63 [0.29, 1.37]
**Subtotal (95% CI)**		**375**		**377**	**0.5%**	**0.63 [0.29, 1.37]**
Total events	10		16			
Heterogeneity: Not applicable						
Test for overall effect: Z = 1.17 (P = 0.24)						
**14.3.6 New onset diseases 'definitely associated' with CRPS ('medically significant conditions'): Cervarix vs. Engerix: hepatitis B vaccine (females and males, age 9-15; follow-up until 12 months)**						
HPV-030: Cervarix vs. Engerix (f, 9-15; 12)	6	247	5	246	0.2%	1.20 [0.37, 3.86]
**Subtotal (95% CI)**		**247**		**246**	**0.2%**	**1.20 [0.37, 3.86]**
Total events	6		5			
Heterogeneity: Not applicable						
Test for overall effect: Z = 0.30 (P = 0.77)						
**14.3.7 New onset diseases 'definitely associated' with CRPS ('medically significant conditions'): Cervarix vs. Havrix: hepatitis A vaccine (females, age 9-15; follow-up until 12 months)**						
HPV-013: Cervarix vs. Havrix (f, 10-14; 12)	31	1035	26	1032	1.0%	1.19 [0.71, 1.99]
HPV-033: Cervarix vs. Havrix (f, 9-15; 7)	4	160	1	161	0.1%	4.03 [0.45, 35.62]
**Subtotal (95% CI)**		**1195**		**1193**	**1.1%**	**1.35 [0.65, 2.84]**
Total events	35		27			
Heterogeneity: $Tau^2 = 0.09$; $Chi^2 = 1.14$, df = 1 (P = 0.29); $I^2 = 12\%$						
Test for overall effect: Z = 0.81 (P = 0.42)						
**14.3.8 New onset diseases 'definitely associated' with CRPS ('medically significant conditions'): Cervarix vs. Havrix: hepatitis A vaccine (females, age 15-25; follow-up until 48 months)**						
HPV-008: Cervarix vs. Havrix (f, 15-25; 48)	399	9319	363	9325	9.7%	1.10 [0.96, 1.26]
**Subtotal (95% CI)**		**9319**		**9325**	**9.7%**	**1.10 [0.96, 1.26]**
Total events	399		363			
Heterogeneity: Not applicable						
Test for overall effect: Z = 1.34 (P = 0.18)						
**14.3.9 New onset diseases 'definitely associated' with CRPS ('medically significant conditions'): Cervarix vs. Twinrix: hepatitis A and B vaccine (females, age 8-15; follow-up until 12 months)**						
HPV-029: Cervarix vs. Twinrix (f, 8-15; 12)	3	270	1	271	0.1%	3.01 [0.32, 28.77]
**Subtotal (95% CI)**		**270**		**271**	**0.1%**	**3.01 [0.32, 28.77]**
Total events	3		1			
Heterogeneity: Not applicable						
Test for overall effect: Z = 0.96 (P = 0.34)						
**14.3.10 New onset diseases 'definitely associated' with CRPS ('new medical history'): Gardasil vs. carrier solution (females and males, age 9-16; follow-up until 18 months)**						
V501-018: Gardasil vs. carrier solu. (f&m,9-16;18)	314	1184	169	597	8.1%	0.94 [0.80, 1.10]
**Subtotal (95% CI)**		**1184**		**597**	**8.1%**	**0.94 [0.80, 1.10]**
Total events	314		169			
Heterogeneity: Not applicable						
Test for overall effect: Z = 0.80 (P = 0.42)						
**14.3.11 New onset diseases 'definitely associated' with CRPS ('new medical history'): Gardasil vs. AAHS (females, age 15-26; follow-up until 45 months)**						
V501-013: Gardasil vs. AAHS (f, 16-24; 45)	998	2723	996	2732	18.9%	1.01 [0.94, 1.08]
V501-015: Gardasil vs. AAHS (f, 15-26; 36)	1860	6087	1904	6080	21.9%	0.98 [0.93, 1.03]
**Subtotal (95% CI)**		**8810**		**8812**	**40.8%**	**0.99 [0.95, 1.03]**
Total events	2858		2900			
Heterogeneity: $Tau^2 = 0.00$; $Chi^2 = 0.44$, df = 1 (P = 0.51); $I^2 = 0\%$						
Test for overall effect: Z = 0.63 (P = 0.53)						
**14.3.12 New onset diseases 'definitely associated' with CRPS ('new medical history'): Gardasil vs. AAHS (females, age 21-46; follow-up until 48 months)**						
V501-019: Gardasil vs. AAHS (f, 21-46; 48)	592	1911	578	1908	14.8%	1.02 [0.93, 1.13]
**Subtotal (95% CI)**		**1911**		**1908**	**14.8%**	**1.02 [0.93, 1.13]**
Total events	592		578			
Heterogeneity: Not applicable						
Test for overall effect: Z = 0.46 (P = 0.65)						
**14.3.13 New onset diseases 'definitely associated' with CRPS ('new medical history'): Gardasil vs. AAHS (males, age 15-27; follow-up until 36 months)**						
V501-020: Gardasil vs. AAHS (m, 15-27; 36)	179	2032	166	2033	5.6%	1.08 [0.88, 1.32]
**Subtotal (95% CI)**		**2032**		**2033**	**5.6%**	**1.08 [0.88, 1.32]**
Total events	179		166			
Heterogeneity: Not applicable						
Test for overall effect: Z = 0.74 (P = 0.46)						
**14.3.14 New onset diseases 'definitely associated' with CRPS ('new medical history'): Gardasil 9 vs. saline placebo (females, age 12-26; follow-up until 7 months)**						
V503-006: Gardasil 9 vs. saline (f, 12-26;7)	42	618	15	306	0.8%	1.39 [0.78, 2.46]
**Subtotal (95% CI)**		**618**		**306**	**0.8%**	**1.39 [0.78, 2.46]**
Total events	42		15			
Heterogeneity: Not applicable						
Test for overall effect: Z = 1.12 (P = 0.26)						
**14.3.15 New onset diseases 'definitely associated' with CRPS ('new medical history'): HPV 16 vaccine vs. AAHS (females, age 16-25; follow-up until 48 months)**						
V501-005: HPV 16 vaccine vs. AAHS (f, 16-25; 48)	292	1204	278	1205	9.3%	1.05 [0.91, 1.21]
**Subtotal (95% CI)**		**1204**		**1205**	**9.3%**	**1.05 [0.91, 1.21]**
Total events	292		278			
Heterogeneity: Not applicable						
Test for overall effect: Z = 0.68 (P = 0.50)						
**Total (95% CI)**		**30896**		**29924**	**100.0%**	**1.04 [0.98, 1.10]**
Total events	5079		4790			
Heterogeneity: $Tau^2 = 0.00$; $Chi^2 = 23.94$, df = 17 (P = 0.12); $I^2 = 29\%$						
Test for overall effect: Z = 1.36 (P = 0.17)						
Test for subgroup differences: $Chi^2 = 16.98$, df = 14 (P = 0.26), $I^2 = 17.5\%$						

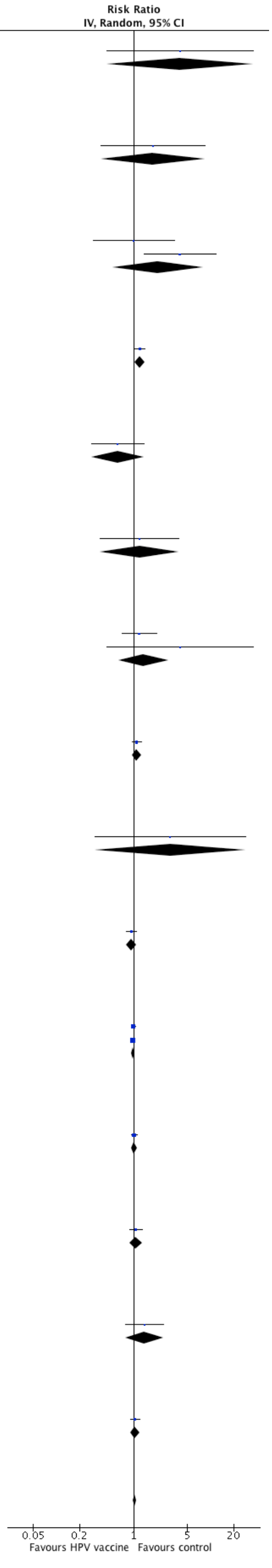

*14.3. Risk ratio for 'medically significant conditions’ (GlaxoSmithKline): **1.18 [1.02, 1.37]**; risk ratio for ‘new medical history’ (Merck Sharp & Dohme): 1.00 [0.96, 1.03]. **We asked a physician with clinical expertise in CRPS to assess the reported MedDRA preferred terms as 'definitely,' 'probably,' 'probably not' or ‘definitely not’ associated with the syndromes. We sent an Excel sheet to the physician with all the reported MedDRA terms. The physician was blinded, as the Excel sheet contained no outcome data. When the physician had assessed all the MedDRA terms, we synthesized the data for those MedDRA terms that the physician judged ‘definitely’ associated with CRPS and compared it to the reported new onset diseases.

- Reported MedDRA terms and number of harms per MedDRA term for the new onset diseases judged as 'definitely associated' with chronic regional pain syndrome (CRPS)

Physician judgement	MedDRA system organ class	MedDRA preferred term	HPV vaccine	Comparator
'Definitely associated with CRPS'	Cardiac disorders (10007541)	Arrhythmia (10003119)	19	12
'Definitely associated with CRPS'	Cardiac disorders (10007541)	Atrial tachycardia	1	1
'Definitely associated with CRPS'	Cardiac disorders (10007541)	Cardiovascular disorder	1	1
'Definitely associated with CRPS'	Cardiac disorders (10007541)	Cardiovascular insufficiency	1	0
'Definitely associated with CRPS'	Cardiac disorders (10007541)	Extra systoles	2	0
'Definitely associated with CRPS'	Cardiac disorders (10007541)	Palpitations (10033557)	23	16
'Definitely associated with CRPS'	Cardiac disorders (10007541)	Sinus arrhythmia	0	1
'Definitely associated with CRPS'	Cardiac disorders (10007541)	Sinus tachycardia (10040752)	5	5
'Definitely associated with CRPS'	Cardiac disorders (10007541)	Supraventricular tachycardia (10042604)	7	2
'Definitely associated with CRPS'	Cardiac disorders (10007541)	Tachycardia (10043071)	16	17
'Definitely associated with CRPS'	Cardiac disorders (10007541)	Tachycardia paroxysmal	1	1
'Definitely associated with CRPS'	Ear and labyrinth disorders (10013993)	Tinnitus (10043882)	13	11
'Definitely associated with CRPS'	Ear and labyrinth disorders (10013993)	Vertigo (10047340)	75	66
'Definitely associated with CRPS'	Ear and labyrinth disorders (10013993)	Vertigo positional	6	7
'Definitely associated with CRPS'	Eye disorders (10015919)	Dry eye (10013774)	8	10
'Definitely associated with CRPS'	Eye disorders (10015919)	Eye disorder	1	1
'Definitely associated with CRPS'	Eye disorders (10015919)	Photophobia	1	0
'Definitely associated with CRPS'	Eye disorders (10015919)	Vision blurred (10047513)	4	7
'Definitely associated with CRPS'	Eye disorders (10015919)	Visual acuity reduced (10047531)	4	4
'Definitely associated with CRPS'	Eye disorders (10015919)	Visual disturbance	5	1
'Definitely associated with CRPS'	Eye disorders (10015919)	Visual impairment (10047571)	1	1
'Definitely associated with CRPS'	Eye disorders (10015919)	Vitreous detachment	0	1
'Definitely associated with CRPS'	Eye disorders (10015919)	Xerophthalmia (10048221)	2	0
'Definitely associated with CRPS'	Gastrointestinal disorders (10017947)	Abdominal discomfort (10000059)	20	23
'Definitely associated with CRPS'	Gastrointestinal disorders (10017947)	Abdominal pain (10000081)	457	400
'Definitely associated with CRPS'	Gastrointestinal disorders (10017947)	Abdominal pain lower (10000084)	183	187
'Definitely associated with CRPS'	Gastrointestinal disorders (10017947)	Abdominal pain upper (10000087)	143	133
'Definitely associated with CRPS'	Gastrointestinal disorders (10017947)	Abdominal symptom	2	0
'Definitely associated with CRPS'	Gastrointestinal disorders (10017947)	Abdominal tenderness	4	6
'Definitely associated with CRPS'	Gastrointestinal disorders (10017947)	Bowel movement irregularity	1	1
'Definitely associated with CRPS'	Gastrointestinal disorders (10017947)	Change of bowel habit (10008399)	5	0
'Definitely associated with CRPS'	Gastrointestinal disorders (10017947)	Constipation (10010774)	160	130
'Definitely associated with CRPS'	Gastrointestinal disorders (10017947)	Diarrhoea (10012735)	320	273
'Definitely associated with CRPS'	Gastrointestinal disorders (10017947)	Dry mouth	1	2
'Definitely associated with CRPS'	Gastrointestinal disorders (10017947)	Dyspepsia (10013946)	154	141
'Definitely associated with CRPS'	Gastrointestinal disorders (10017947)	Epigastric discomfort (10053155)	3	4
'Definitely associated with CRPS'	Gastrointestinal disorders (10017947)	Food poisoning (10016952)	60	48
'Definitely associated with CRPS'	Gastrointestinal disorders (10017947)	Frequent bowel movements	1	0
'Definitely associated with CRPS'	Gastrointestinal disorders (10017947)	Gastric Disorder	3	1
'Definitely associated with CRPS'	Gastrointestinal disorders (10017947)	Gastrointestinal disorder (10017944)	6	12
'Definitely associated with CRPS'	Gastrointestinal disorders (10017947)	Gastrointestinal hypomotility	0	1
'Definitely associated with CRPS'	Gastrointestinal disorders (10017947)	Gastrointestinal pain	4	5
'Definitely associated with CRPS'	Gastrointestinal disorders (10017947)	Intestinal functional disorder (10061247)	1	2
'Definitely associated with CRPS'	Gastrointestinal disorders (10017947)	Irritable bowel syndrome (10023003)	117	114
'Definitely associated with CRPS'	Gastrointestinal disorders (10017947)	Nausea (10028813)	168	188
'Definitely associated with CRPS'	Gastrointestinal disorders (10017947)	Stomach discomfort	2	11
'Definitely associated with CRPS'	Gastrointestinal disorders (10017947)	Vomiting (10047700)	112	117
'Definitely associated with CRPS'	General disorders and administration site conditions (10018065)	Asthenia (10003549)	21	16
'Definitely associated with CRPS'	General disorders and administration site conditions (10018065)	Axillary pain (10048750)	4	2
'Definitely associated with CRPS'	General disorders and administration site conditions (10018065)	Discomfort	3	0
'Definitely associated with CRPS'	General disorders and administration site conditions (10018065)	Facial pain (10016059)	2	2
'Definitely associated with CRPS'	General disorders and administration site conditions (10018065)	Fatigue (10016256)	73	72
'Definitely associated with CRPS'	General disorders and administration site conditions (10018065)	Feeling cold	3	2

'Definitely associated with CRPS'	General disorders and administration site conditions (10018065)	Feeling of body temperature change	3	6
'Definitely associated with CRPS'	General disorders and administration site conditions (10018065)	General Symptom	2	1
'Definitely associated with CRPS'	General disorders and administration site conditions (10018065)	Local swelling (10024770)	5	5
'Definitely associated with CRPS'	General disorders and administration site conditions (10018065)	Malaise (10025482)	20	14
'Definitely associated with CRPS'	General disorders and administration site conditions (10018065)	Mucosal dryness	1	0
'Definitely associated with CRPS'	General disorders and administration site conditions (10018065)	Non-cardiac chest pain (10062501)	19	9
'Definitely associated with CRPS'	General disorders and administration site conditions (10018065)	Pain (10033371)	17	28
'Definitely associated with CRPS'	General disorders and administration site conditions (10018065)	Suprapubic pain	3	2
'Definitely associated with CRPS'	General disorders and administration site conditions (10018065)	Tenderness	3	1
'Definitely associated with CRPS'	Investigations (10022891)	Abdomen Scan	2	0
'Definitely associated with CRPS'	Investigations (10022891)	Abdomen scan normal	0	1
'Definitely associated with CRPS'	Investigations (10022891)	Abdominal X-Ray	3	4
'Definitely associated with CRPS'	Investigations (10022891)	Blood glucose	1	2
'Definitely associated with CRPS'	Investigations (10022891)	Blood glucose normal	1	1
'Definitely associated with CRPS'	Investigations (10022891)	Blood Test	13	13
'Definitely associated with CRPS'	Investigations (10022891)	Diagnostic procedure	1	1
'Definitely associated with CRPS'	Investigations (10022891)	Heart rate irregular (10019304)	4	0
'Definitely associated with CRPS'	Investigations (10022891)	Investigation	2	0
'Definitely associated with CRPS'	Investigations (10022891)	Laboratory Test	0	3
'Definitely associated with CRPS'	Investigations (10022891)	Lumbar puncture	3	2
'Definitely associated with CRPS'	Investigations (10022891)	Medical observation	1	0
'Definitely associated with CRPS'	Investigations (10022891)	Physical examination	1	1
'Definitely associated with CRPS'	Investigations (10022891)	Tilt table test	1	0
'Definitely associated with CRPS'	Investigations (10022891)	Ultrasound abdomen	41	44
'Definitely associated with CRPS'	Investigations (10022891)	Ultrasound abdomen normal	7	8
'Definitely associated with CRPS'	Investigations (10022891)	Ultrasound scan	475	519
'Definitely associated with CRPS'	Investigations (10022891)	Ultrasound scan normal	1	4
'Definitely associated with CRPS'	Musculoskeletal and connective tissue disorders (10028395)	Arthralgia (10003239)	190	193
'Definitely associated with CRPS'	Musculoskeletal and connective tissue disorders (10028395)	Back pain (10003988)	462	410
'Definitely associated with CRPS'	Musculoskeletal and connective tissue disorders (10028395)	Fibromyalgia (10048439)	13	12
'Definitely associated with CRPS'	Musculoskeletal and connective tissue disorders (10028395)	Limb discomfort (10061224)	2	1
'Definitely associated with CRPS'	Musculoskeletal and connective tissue disorders (10028395)	Muscle atrophy	0	1
'Definitely associated with CRPS'	Musculoskeletal and connective tissue disorders (10028395)	Muscle contracture (10062575)	21	21
'Definitely associated with CRPS'	Musculoskeletal and connective tissue disorders (10028395)	Muscle cramp	1	0
'Definitely associated with CRPS'	Musculoskeletal and connective tissue disorders (10028395)	Muscle disorder	1	5
'Definitely associated with CRPS'	Musculoskeletal and connective tissue disorders (10028395)	Muscle spasms (10028334)	51	38
'Definitely associated with CRPS'	Musculoskeletal and connective tissue disorders (10028395)	Muscle twitching (10028347)	3	3
'Definitely associated with CRPS'	Musculoskeletal and connective tissue disorders (10028395)	Muscle weakness	2	0
'Definitely associated with CRPS'	Musculoskeletal and connective tissue disorders (10028395)	Muscular weakness	2	2
'Definitely associated with CRPS'	Musculoskeletal and connective tissue disorders (10028395)	Musculoskeletal discomfort (10053156)	3	3
'Definitely associated with CRPS'	Musculoskeletal and connective tissue disorders (10028395)	Musculoskeletal pain (10028391)	37	40
'Definitely associated with CRPS'	Musculoskeletal and connective tissue disorders (10028395)	Musculoskeletal stiffness (10052904)	16	17
'Definitely associated with CRPS'	Musculoskeletal and connective tissue disorders (10028395)	Myalgia (10028411)	79	78
'Definitely associated with CRPS'	Musculoskeletal and connective tissue disorders (10028395)	Neck pain (10028836)	52	52
'Definitely associated with CRPS'	Musculoskeletal and connective tissue disorders (10028395)	Osteopenia (10049088)	2	11
'Definitely associated with CRPS'	Musculoskeletal and connective tissue disorders (10028395)	Osteoporosis (10031282)	3	6
'Definitely associated with CRPS'	Musculoskeletal and connective tissue disorders (10028395)	Pain in extremity (10033425)	95	90
'Definitely associated with CRPS'	Musculoskeletal and connective tissue disorders (10028395)	Pain in jaw (10033433)	8	9
'Definitely associated with CRPS'	Neoplasm's benign, malignant and unspecified (10029104)	Nervous system neoplasm benign	1	0
'Definitely associated with CRPS'	Nervous system disorders (10029205)	Autonomous nervous system imbalance	3	1
'Definitely associated with CRPS'	Nervous system disorders (10029205)	Balance disorder (10049848)	3	1
'Definitely associated with CRPS'	Nervous system disorders (10029205)	Burning sensation (10006784)	3	1
'Definitely associated with CRPS'	Nervous system disorders (10029205)	Disturbance in attention (10013496)	1	1
'Definitely associated with CRPS'	Nervous system disorders (10029205)	Dizziness (10013573)	139	131
'Definitely associated with CRPS'	Nervous system disorders (10029205)	Dizziness postural	0	2
'Definitely associated with CRPS'	Nervous system disorders (10029205)	Dysesthesia	0	2
'Definitely associated with CRPS'	Nervous system disorders (10029205)	Headache (10019211)	855	756

‘Definitely associated with CRPS’	Nervous system disorders (10029205)	Hyperaesthesia	3	1
‘Definitely associated with CRPS’	Nervous system disorders (10029205)	Lethargy (10024264)	7	8
‘Definitely associated with CRPS’	Nervous system disorders (10029205)	Nervous system disorder	1	1
‘Definitely associated with CRPS’	Nervous system disorders (10029205)	Neuralgia (10029223)	6	8
‘Definitely associated with CRPS’	Nervous system disorders (10029205)	Neurological symptom	1	0
‘Definitely associated with CRPS’	Nervous system disorders (10029205)	Neuropathy peripheral (10029331)	2	7
‘Definitely associated with CRPS’	Nervous system disorders (10029205)	Paraesthesia (10033775)	15	11
‘Definitely associated with CRPS’	Nervous system disorders (10029205)	Restless legs syndrome (10058920)	3	11
‘Definitely associated with CRPS’	Nervous system disorders (10029205)	Tension headache (10043269)	80	68
‘Definitely associated with CRPS’	Renal and urinary disorders (10038359)	Cystitis interstitial (10011796)	5	3
‘Definitely associated with CRPS’	Renal and urinary disorders (10038359)	Cystitis non-infective	1	4
‘Definitely associated with CRPS’	Renal and urinary disorders (10038359)	Cystitis-like symptom	39	44
‘Definitely associated with CRPS’	Renal and urinary disorders (10038359)	Micturition disorder (10027561)	2	0
‘Definitely associated with CRPS’	Renal and urinary disorders (10038359)	Urogenital disorder	4	1
‘Definitely associated with CRPS’	Skin and subcutaneous tissue disorders (10040785)	Nail growth abnormal	1	0
‘Definitely associated with CRPS’	Skin and subcutaneous tissue disorders (10040785)	Pain of skin (10033474)	0	1
‘Definitely associated with CRPS’	Skin and subcutaneous tissue disorders (10040785)	Skin burning sensation	0	1
‘Definitely associated with CRPS’	Vascular disorders (10047065)	Peripheral vascular disorder	0	1
‘Definitely associated with CRPS’	Vascular disorders (10047065)	Raynaud's phenomenon (10037912)	2	6
**Total**			**5,079**	**4,790**

14.4. New onset diseases ('medically significant conditions' and 'new medical history'*) judged as ‘definitely associated’** with postural orthostatic tachycardia syndrome (POTS): intention to treat analysis

Study or Subgroup	HPV vaccine Events	HPV vaccine Total	Control Events	Control Total	Weight	Risk Ratio IV, Random, 95% CI
**14.4.1 New onset diseases 'definitely associated' with POTS ('medically significant conditions'): Cervarix vs. Al(OH)3 (females, age 9-17; follow-up until 12 months)**						
HPV-058: Cervarix vs. Al(OH)3 (f, 9-17; 12)	3	374	1	376	0.1%	3.02 [0.32, 28.86]
**Subtotal (95% CI)**		**374**		**376**	**0.1%**	**3.02 [0.32, 28.86]**
Total events	3		1			
Heterogeneity: Not applicable						
Test for overall effect: Z = 0.96 (P = 0.34)						
**14.4.2 New onset diseases 'definitely associated' with POTS ('medically significant conditions'): Cervarix vs. Al(OH)3 (females, age 15-25; follow-up until 7 months)**						
HPV-038: Cervarix vs. Al(OH)3 (f, 15-25; 7)	5	149	2	76	0.2%	1.28 [0.25, 6.42]
**Subtotal (95% CI)**		**149**		**76**	**0.2%**	**1.28 [0.25, 6.42]**
Total events	5		2			
Heterogeneity: Not applicable						
Test for overall effect: Z = 0.29 (P = 0.77)						
**14.4.3 New onset diseases 'definitely associated' with POTS ('medically significant conditions'): Cervarix vs. Al(OH)3 (females, age 18-35; follow-up until 7 months)**						
HPV-031: Cervarix vs. Al(OH)3 (f, 18-35; 7)	2	176	2	178	0.1%	1.01 [0.14, 7.10]
HPV-035: Cervarix vs. Al(OH)3 (f, 18-35; 7)	12	150	3	150	0.3%	4.00 [1.15, 13.89]
**Subtotal (95% CI)**		**326**		**328**	**0.4%**	**2.49 [0.69, 8.96]**
Total events	14		5			
Heterogeneity: Tau² = 0.25; Chi² = 1.36, df = 1 (P = 0.24); I² = 26%						
Test for overall effect: Z = 1.39 (P = 0.16)						
**14.4.4 New onset diseases 'definitely associated' with POTS ('medically significant conditions'): Cervarix vs. Al(OH)3 (females, age 24-72; follow-up until 36 months)**						
HPV-015: Cervarix vs. Al(OH)3 (f, 24-72; 36)	215	2882	153	2871	7.7%	1.40 [1.15, 1.71]
**Subtotal (95% CI)**		**2882**		**2871**	**7.7%**	**1.40 [1.15, 1.71]**
Total events	215		153			
Heterogeneity: Not applicable						
Test for overall effect: Z = 3.28 (P = 0.001)						
**14.4.5 New onset diseases 'definitely associated' with POTS ('medically significant conditions'): Cervarix vs. Aimmugen: hepatitis A vaccine (females, age 22-29; follow-up until 12 months)**						
HPV-063: Cervarix vs. Aimmugen (f, 22-29; 12)	9	375	15	377	0.6%	0.60 [0.27, 1.36]
**Subtotal (95% CI)**		**375**		**377**	**0.6%**	**0.60 [0.27, 1.36]**
Total events	9		15			
Heterogeneity: Not applicable						
Test for overall effect: Z = 1.22 (P = 0.22)						
**14.4.6 New onset diseases 'definitely associated' with POTS ('medically significant conditions'): Cervarix vs. Engerix: hepatitis B vaccine (females and males, age 9-15; follow-up until 12 months)**						
HPV-030: Cervarix vs. Engerix (f, 9-15; 12)	4	247	4	246	0.2%	1.00 [0.25, 3.94]
**Subtotal (95% CI)**		**247**		**246**	**0.2%**	**1.00 [0.25, 3.94]**
Total events	4		4			
Heterogeneity: Not applicable						
Test for overall effect: Z = 0.01 (P = 1.00)						
**14.4.7 New onset diseases 'definitely associated' with POTS ('medically significant conditions'): Cervarix vs. Havrix: hepatitis A vaccine (females, age 9-15; follow-up until 12 months)**						
HPV-013: Cervarix vs. Havrix (f, 10-14; 12)	29	1035	28	1032	1.6%	1.03 [0.62, 1.72]
HPV-033: Cervarix vs. Havrix (f, 9-15; 7)	4	160	1	161	0.1%	4.03 [0.45, 35.62]
**Subtotal (95% CI)**		**1195**		**1193**	**1.7%**	**1.33 [0.47, 3.73]**
Total events	33		29			
Heterogeneity: Tau² = 0.27; Chi² = 1.42, df = 1 (P = 0.23); I² = 29%						
Test for overall effect: Z = 0.54 (P = 0.59)						
**14.4.8 New onset diseases 'definitely associated' with POTS ('medically significant conditions'): Cervarix vs. Havrix: hepatitis A vaccine (females, age 15-25; follow-up until 48 months)**						
HPV-008: Cervarix vs. Havrix (f, 15-25; 48)	319	9319	292	9325	10.8%	1.09 [0.94, 1.28]
**Subtotal (95% CI)**		**9319**		**9325**	**10.8%**	**1.09 [0.94, 1.28]**
Total events	319		292			
Heterogeneity: Not applicable						
Test for overall effect: Z = 1.12 (P = 0.26)						
**14.4.9 New onset diseases 'definitely associated' with POTS ('medically significant conditions'): Cervarix vs. Twinrix: hepatitis A and B vaccine (females, age 8-15; follow-up until 12 months)**						
HPV-029: Cervarix vs. Twinrix (f, 8-15; 12)	2	270	1	271	0.1%	2.01 [0.18, 22.01]
**Subtotal (95% CI)**		**270**		**271**	**0.1%**	**2.01 [0.18, 22.01]**
Total events	2		1			
Heterogeneity: Not applicable						
Test for overall effect: Z = 0.57 (P = 0.57)						
**14.4.10 New onset diseases 'definitely associated' with POTS ('new medical history'): Gardasil vs. carrier solution (females and males, age 9-16; follow-up until 18 months)**						
V501-018: Gardasil vs. carrier solu. (f&m,9-16;18)	246	1184	131	597	8.5%	0.95 [0.78, 1.14]
**Subtotal (95% CI)**		**1184**		**597**	**8.5%**	**0.95 [0.78, 1.14]**
Total events	246		131			
Heterogeneity: Not applicable						
Test for overall effect: Z = 0.57 (P = 0.57)						
**14.4.11 New onset diseases 'definitely associated' with POTS ('new medical history'): Gardasil vs. AAHS (females, age 15-26; follow-up until 45 months)**						
V501-013: Gardasil vs. AAHS (f, 16-24; 45)	762	2723	725	2732	18.5%	1.05 [0.97, 1.15]
V501-015: Gardasil vs. AAHS (f, 15-26; 36)	1271	6087	1295	6080	21.0%	0.98 [0.92, 1.05]
**Subtotal (95% CI)**		**8810**		**8812**	**39.4%**	**1.01 [0.94, 1.09]**
Total events	2033		2020			
Heterogeneity: Tau² = 0.00; Chi² = 1.67, df = 1 (P = 0.20); I² = 40%						
Test for overall effect: Z = 0.32 (P = 0.75)						
**14.4.12 New onset diseases 'definitely associated' with POTS ('new medical history'): Gardasil vs. AAHS (females, age 21-46; follow-up until 48 months)**						
V501-019: Gardasil vs. AAHS (f, 21-46; 48)	380	1911	345	1908	13.1%	1.10 [0.96, 1.25]
**Subtotal (95% CI)**		**1911**		**1908**	**13.1%**	**1.10 [0.96, 1.25]**
Total events	380		345			
Heterogeneity: Not applicable						
Test for overall effect: Z = 1.42 (P = 0.16)						
**14.4.13 New onset diseases 'definitely associated' with POTS ('new medical history'): Gardasil vs. AAHS (males, age 15-27; follow-up until 36 months)**						
V501-020: Gardasil vs. AAHS (m, 15-27; 36)	133	2032	109	2033	5.7%	1.22 [0.95, 1.56]
**Subtotal (95% CI)**		**2032**		**2033**	**5.7%**	**1.22 [0.95, 1.56]**
Total events	133		109			
Heterogeneity: Not applicable						
Test for overall effect: Z = 1.59 (P = 0.11)						
**14.4.14 New onset diseases 'definitely associated' with POTS ('new medical history'): Gardasil 9 vs. saline placebo (females, age 12-26; follow-up until 7 months)**						
V503-006: Gardasil 9 vs. saline (f, 12-26;7)	32	618	14	306	1.1%	1.13 [0.61, 2.09]
**Subtotal (95% CI)**		**618**		**306**	**1.1%**	**1.13 [0.61, 2.09]**
Total events	32		14			
Heterogeneity: Not applicable						
Test for overall effect: Z = 0.40 (P = 0.69)						
**14.4.15 New onset diseases 'definitely associated' with POTS ('new medical history'): HPV 16 vaccine vs. AAHS (females, age 16-25; follow-up until 48 months)**						
V501-005: HPV 16 vaccine vs. AAHS (f, 16-25; 48)	247	1204	231	1205	10.4%	1.07 [0.91, 1.26]
**Subtotal (95% CI)**		**1204**		**1205**	**10.4%**	**1.07 [0.91, 1.26]**
Total events	247		231			
Heterogeneity: Not applicable						
Test for overall effect: Z = 0.83 (P = 0.41)						
**Total (95% CI)**		**30896**		**29924**	**100.0%**	**1.08 [1.01, 1.15]**
Total events	3675		3352			
Heterogeneity: Tau² = 0.00; Chi² = 24.03, df = 17 (P = 0.12); I² = 29%						
Test for overall effect: Z = 2.17 (P = 0.03)						
Test for subgroup differences: Chi² = 17.08, df = 14 (P = 0.25), I² = 18.0%						

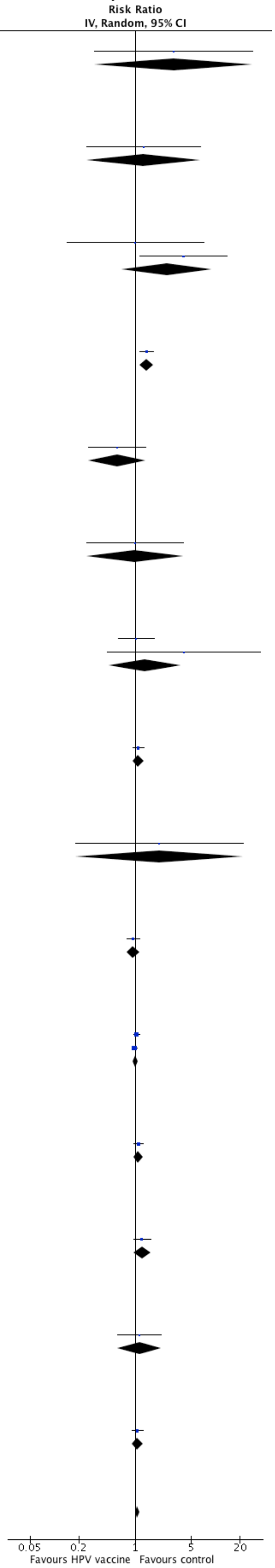

*14.4. Risk ratio for 'medically significant conditions' (GlaxoSmithKline): **1.21 [1.00, 1.45]**; risk ratio for ‘new medical history’ (Merck Sharp & Dohme): 1.03 [0.98, 1.07]. **We asked a physician with clinical expertise in POTS to assess the reported MedDRA preferred terms as 'definitely,' 'probably,' 'probably not' or ‘definitely not’ associated with the syndromes. We sent an Excel sheet to the physician with all the reported MedDRA terms. The physician was blinded, as the Excel sheet contained no outcome data. When the physician had assessed all the MedDRA terms, we synthesized the data for those MedDRA terms that the physician judged ‘definitely’ associated with POTS and compared it to the reported new onset diseases.

- Reported MedDRA terms and number of harms per MedDRA term for the new onset diseases that were judged 'definitely associated' with postural orthostatic tachycardia syndrome (POTS):

Physician judgment	MedDRA system organ class	MedDRA preferred term	HPV vaccine	Comparator
'Definitely associated with POTS'	Cardiac disorders (10007541)	Arrhythmia (10003119)	9	12
'Definitely associated with POTS'	Cardiac disorders (10007541)	Atrial tachycardia	1	1
'Definitely associated with POTS'	Cardiac disorders (10007541)	Cardiovascular disorder	1	1
'Definitely associated with POTS'	Cardiac disorders (10007541)	Cardiovascular insufficiency	1	0
'Definitely associated with POTS'	Cardiac disorders (10007541)	Extra systoles	2	0
'Definitely associated with POTS'	Cardiac disorders (10007541)	Mitral valve prolapse (10027730)	9	10
'Definitely associated with POTS'	Cardiac disorders (10007541)	Palpitations (10033557)	23	16
'Definitely associated with POTS'	Cardiac disorders (10007541)	Sinus arrhythmia	0	1
'Definitely associated with POTS'	Cardiac disorders (10007541)	Sinus tachycardia (10040752)	5	5
'Definitely associated with POTS'	Cardiac disorders (10007541)	Supraventricular tachycardia (10042604)	7	2
'Definitely associated with POTS'	Cardiac disorders (10007541)	Tachycardia (10043071)	16	17
'Definitely associated with POTS'	Cardiac disorders (10007541)	Tachycardia paroxysmal	1	1
'Definitely associated with POTS'	Ear and labyrinth disorders (10013993)	Tinnitus (10043882)	13	11
'Definitely associated with POTS'	Ear and labyrinth disorders (10013993)	Vertigo (10047340)	75	66
'Definitely associated with POTS'	Ear and labyrinth disorders (10013993)	Vertigo positional	6	7
'Definitely associated with POTS'	Eye disorders (10015919)	Dry eye (10013774)	8	10
'Definitely associated with POTS'	Eye disorders (10015919)	Photophobia	1	0
'Definitely associated with POTS'	Eye disorders (10015919)	Vision blurred (10047513)	4	7
'Definitely associated with POTS'	Eye disorders (10015919)	Visual acuity reduced (10047531)	4	4
'Definitely associated with POTS'	Eye disorders (10015919)	Visual disturbance	5	1
'Definitely associated with POTS'	Eye disorders (10015919)	Visual impairment (10047571)	1	1
'Definitely associated with POTS'	Eye disorders (10015919)	Vitreous detachment	0	1
'Definitely associated with POTS'	Eye disorders (10015919)	Xerophthalmia (10048221)	2	0
'Definitely associated with POTS'	Gastrointestinal disorders (10017947)	Abdominal discomfort (10000059)	20	23
'Definitely associated with POTS'	Gastrointestinal disorders (10017947)	Abdominal pain (10000081)	457	400
'Definitely associated with POTS'	Gastrointestinal disorders (10017947)	Abdominal pain lower (10000084)	183	187
'Definitely associated with POTS'	Gastrointestinal disorders (10017947)	Abdominal pain upper (10000087)	143	133
'Definitely associated with POTS'	Gastrointestinal disorders (10017947)	Abdominal symptom	2	0
'Definitely associated with POTS'	Gastrointestinal disorders (10017947)	Abdominal tenderness	4	6
'Definitely associated with POTS'	Gastrointestinal disorders (10017947)	Bowel movement irregularity	1	1
'Definitely associated with POTS'	Gastrointestinal disorders (10017947)	Change of bowel habit (10008399)	5	0
'Definitely associated with POTS'	Gastrointestinal disorders (10017947)	Constipation (10010774)	160	130
'Definitely associated with POTS'	Gastrointestinal disorders (10017947)	Diarrhoea (10012735)	320	273
'Definitely associated with POTS'	Gastrointestinal disorders (10017947)	Dry Mouth	1	2
'Definitely associated with POTS'	Gastrointestinal disorders (10017947)	Dyspepsia (10013946)	154	141
'Definitely associated with POTS'	Gastrointestinal disorders (10017947)	Epigastric discomfort (10053155)	3	4
'Definitely associated with POTS'	Gastrointestinal disorders (10017947)	Food poisoning (10016952)	60	48
'Definitely associated with POTS'	Gastrointestinal disorders (10017947)	Frequent bowel movements	1	0
'Definitely associated with POTS'	Gastrointestinal disorders (10017947)	Gastric Disorder	3	1
'Definitely associated with POTS'	Gastrointestinal disorders (10017947)	Gastrointestinal disorder (10017944)	6	12
'Definitely associated with POTS'	Gastrointestinal disorders (10017947)	Gastrointestinal hypomotility	4	1
'Definitely associated with POTS'	Gastrointestinal disorders (10017947)	Gastrointestinal pain	4	5
'Definitely associated with POTS'	Gastrointestinal disorders (10017947)	Intestinal functional disorder (10061247)	1	2
'Definitely associated with POTS'	Gastrointestinal disorders (10017947)	Irritable bowel syndrome (10023003)	117	114
'Definitely associated with POTS'	Gastrointestinal disorders (10017947)	Nausea (10028813)	168	188
'Definitely associated with POTS'	Gastrointestinal disorders (10017947)	Stomach discomfort	2	11
'Definitely associated with POTS'	Gastrointestinal disorders (10017947)	Vomiting (10047700)	112	117
'Definitely associated with POTS'	General disorders and administration site conditions (10018065)	Asthenia (10003549)	21	16
'Definitely associated with POTS'	General disorders and administration site conditions (10018065)	Chest discomfort (10008469)	6	3
'Definitely associated with POTS'	General disorders and administration site conditions (10018065)	Chest pain (10008479)	35	23
'Definitely associated with POTS'	General disorders and administration site conditions (10018065)	Discomfort	3	0
'Definitely associated with POTS'	General disorders and administration site conditions (10018065)	Fatigue (10016256)	73	72
'Definitely associated with POTS'	General disorders and administration site conditions (10018065)	General Symptom	2	1

'Definitely associated with POTS'	General disorders and administration site conditions (10018065)	Malaise (10025482)	20	14
'Definitely associated with POTS'	General disorders and administration site conditions (10018065)	Mucosal dryness	1	0
'Definitely associated with POTS'	General disorders and administration site conditions (10018065)	Non-cardiac chest pain (10062501)	19	9
'Definitely associated with POTS'	Investigations (10022891)	Cardiac imaging Procedure	1	0
'Definitely associated with POTS'	Investigations (10022891)	Cardiac stress test	0	1
'Definitely associated with POTS'	Investigations (10022891)	Chest X-ray	19	16
'Definitely associated with POTS'	Investigations (10022891)	Chest X-ray normal	2	5
'Definitely associated with POTS'	Investigations (10022891)	Diagnostic procedure	1	1
'Definitely associated with POTS'	Investigations (10022891)	Heart rate increased	1	1
'Definitely associated with POTS'	Investigations (10022891)	Heart rate irregular (10019304)	4	0
'Definitely associated with POTS'	Investigations (10022891)	Investigation	2	0
'Definitely associated with POTS'	Investigations (10022891)	Laboratory test	0	3
'Definitely associated with POTS'	Investigations (10022891)	Medical observation	1	0
'Definitely associated with POTS'	Investigations (10022891)	Physical examination	1	1
'Definitely associated with POTS'	Investigations (10022891)	Tilt table test	1	0
'Definitely associated with POTS'	Musculoskeletal and connective tissue disorders (10028395)	Musculoskeletal chest pain (10050819)	25	26
'Definitely associated with POTS'	Neoplasms benign, malignant and unspecified (10029104)	Nervous system neoplasm benign	1	0
'Definitely associated with POTS'	Nervous system disorders (10029205)	Autonomic nervous system imbalance	3	1
'Definitely associated with POTS'	Nervous system disorders (10029205)	Balance disorder (10049848)	3	1
'Definitely associated with POTS'	Nervous system disorders (10029205)	Circadian rhythm disorder	1	0
'Definitely associated with POTS'	Nervous system disorders (10029205)	Disturbance in attention (10013496)	1	1
'Definitely associated with POTS'	Nervous system disorders (10029205)	Dizziness (10013573)	139	131
'Definitely associated with POTS'	Nervous system disorders (10029205)	Dizziness postural	0	2
'Definitely associated with POTS'	Nervous system disorders (10029205)	Headache (10019211)	855	756
'Definitely associated with POTS'	Nervous system disorders (10029205)	Lethargy (10024264)	7	8
'Definitely associated with POTS'	Nervous system disorders (10029205)	Loss of consciousness	8	5
'Definitely associated with POTS'	Nervous system disorders (10029205)	Nervous system disorder	1	1
'Definitely associated with POTS'	Nervous system disorders (10029205)	Neurological symptom	1	0
'Definitely associated with POTS'	Nervous system disorders (10029205)	Presyncope (10036653)	4	6
'Definitely associated with POTS'	Nervous system disorders (10029205)	Restless legs syndrome (10058920)	9	11
'Definitely associated with POTS'	Nervous system disorders (10029205)	Syncope (10042772)	62	60
'Definitely associated with POTS'	Nervous system disorders (10029205)	Syncope vasovagal	12	7
'Definitely associated with POTS'	Nervous system disorders (10029205)	Tension headache (10043269)	80	68
'Definitely associated with POTS'	Renal and urinary disorders (10038359)	Cystitis interstitial (10011796)	5	3
'Definitely associated with POTS'	Renal and urinary disorders (10038359)	Cystitis non-infective	1	4
'Definitely associated with POTS'	Renal and urinary disorders (10038359)	Cystitis-like symptom	39	44
'Definitely associated with POTS'	Renal and urinary disorders (10038359)	Micturition disorder (10027561)	2	0
'Definitely associated with POTS'	Renal and urinary disorders (10038359)	Urogenital disorder	4	1
'Definitely associated with POTS'	Respiratory, thoracic and mediastinal disorders (10038738)	Dyspnoea (10013968)	43	24
'Definitely associated with POTS'	Respiratory, thoracic and mediastinal disorders (10038738)	Hyperventilation (10020910)	5	11
'Definitely associated with POTS'	Vascular disorders (10047065)	Hypotension (10021097)	20	29
'Definitely associated with POTS'	Vascular disorders (10047065)	Orthostatic hypotension	4	6
'Definitely associated with POTS'	Vascular disorders (10047065)	Peripheral vascular disorder	0	1
'Definitely associated with POTS'	Vascular disorders (10047065)	Raynaud's phenomenon (10037912)	2	6
**Total**			**3,675**	**3,352**

14.5. General harms ('solicited,' 'unsolicited' and 'systemic adverse events') judged as 'definitely associated' with chronic regional pain syndrome (CRPS): intention to treat analysis

This meta-analysis was inappropriate, as some of the numerators exceeded the denominators making the result nonsensical.

14.6. General harms ('solicited,' 'unsolicited' and 'systemic adverse events') judged as 'definitely associated' with postural orthostatic tachycardia syndrome (POTS): intention to treat analysis

This meta-analysis was inappropriate, as some of the numerators exceeded the denominators making the result nonsensical.

14.7. Serious harms part of VigiBase's largest HPV vaccine-associated harm clusters* – 'expected systemic reactions': intention to treat analysis

Study or Subgroup	HPV vaccine Events	HPV vaccine Total	Control Events	Control Total	Weight	Risk Ratio IV, Random, 95% CI
**14.7.1 Serious expected systemic reactions: Cervarix vs. Al(OH)3 (females, age 18–35; follow-up until 7 months)**						
HPV-035: Cervarix vs. Al(OH)3 (f, 18–35; 7)	2	150	0	150	5.5%	5.00 [0.24, 103.28]
**Subtotal (95% CI)**		**150**		**150**	**5.5%**	**5.00 [0.24, 103.28]**
Total events	2		0			
Heterogeneity: Not applicable						
Test for overall effect: Z = 1.04 (P = 0.30)						
**14.7.2 Serious expected systemic reactions: Cervarix vs. Al(OH)3 (females, age 24–72; follow-up until 36 months)**						
HPV-015: Cervarix vs. Al(OH)3 (f, 24–72; 36)	3	2882	1	2871	9.8%	2.99 [0.31, 28.71]
**Subtotal (95% CI)**		**2882**		**2871**	**9.8%**	**2.99 [0.31, 28.71]**
Total events	3		1			
Heterogeneity: Not applicable						
Test for overall effect: Z = 0.95 (P = 0.34)						
**14.7.3 Serious expected systemic reactions: Cervarix vs. Engerix: hepatitis B vaccine (females and males, age 12–16; follow-up until 72 months)**						
HPV-040: Cervarix vs. Engerix (f & m, 12–16; 72)	1	2436	0	1267	4.9%	1.56 [0.06, 38.29]
**Subtotal (95% CI)**		**2436**		**1267**	**4.9%**	**1.56 [0.06, 38.29]**
Total events	1		0			
Heterogeneity: Not applicable						
Test for overall effect: Z = 0.27 (P = 0.79)						
**14.7.4 Serious expected systemic reactions: Cervarix vs. Havrix: hepatitis A vaccine (females, age 10–14; follow-up until 12 months)**						
HPV-013: Cervarix vs. Havrix (f, 10–14; 12)	0	1035	1	1032	4.9%	0.33 [0.01, 8.15]
**Subtotal (95% CI)**		**1035**		**1032**	**4.9%**	**0.33 [0.01, 8.15]**
Total events	0		1			
Heterogeneity: Not applicable						
Test for overall effect: Z = 0.67 (P = 0.50)						
**14.7.5 Serious expected systemic reactions: Cervarix vs. Havrix: hepatitis A vaccine (females, age 15–25; follow-up until 48 months)**						
HPV-008: Cervarix vs. Havrix (f, 15–25; 48)	13	9319	4	9325	40.1%	3.25 [1.06, 9.97]
**Subtotal (95% CI)**		**9319**		**9325**	**40.1%**	**3.25 [1.06, 9.97]**
Total events	13		4			
Heterogeneity: Not applicable						
Test for overall effect: Z = 2.06 (P = 0.04)						
**14.7.6 Serious expected systemic reactions: Gardasil vs. AAHS (females, age 15–26; follow-up until 45 months)**						
V501-013: Gardasil vs. AAHS (f, 16–24; 45)	1	2723	2	2732	8.7%	0.50 [0.05, 5.53]
V501-015: Gardasil vs. AAHS (f, 15–26; 36)	3	6087	2	6080	15.7%	1.50 [0.25, 8.96]
**Subtotal (95% CI)**		**8810**		**8812**	**24.4%**	**1.01 [0.24, 4.25]**
Total events	4		4			
Heterogeneity: $Tau^2 = 0.00$; $Chi^2 = 0.51$, df = 1 (P = 0.47); $I^2 = 0\%$						
Test for overall effect: Z = 0.02 (P = 0.99)						
**14.7.7 Serious expected systemic reactions: Gardasil vs. AAHS (females, age 21–46; follow-up until 48 months)**						
V501-019: Gardasil vs. AAHS (f, 21–46; 48)	2	1911	0	1908	5.5%	4.99 [0.24, 103.91]
**Subtotal (95% CI)**		**1911**		**1908**	**5.5%**	**4.99 [0.24, 103.91]**
Total events	2		0			
Heterogeneity: Not applicable						
Test for overall effect: Z = 1.04 (P = 0.30)						
**14.7.8 Serious expected systemic reactions: HPV 16 vaccine vs. AAHS (females, age 16–25; follow-up until 48 months)**						
V501-005: HPV 16 vaccine vs. AAHS (f, 16–25; 48)	0	1204	1	1205	4.9%	0.33 [0.01, 8.18]
**Subtotal (95% CI)**		**1204**		**1205**	**4.9%**	**0.33 [0.01, 8.18]**
Total events	0		1			
Heterogeneity: Not applicable						
Test for overall effect: Z = 0.67 (P = 0.50)						
**Total (95% CI)**		**27747**		**26570**	**100.0%**	**1.96 [0.96, 3.98]**
Total events	25		11			
Heterogeneity: $Tau^2 = 0.00$; $Chi^2 = 5.35$, df = 8 (P = 0.72); $I^2 = 0\%$						
Test for overall effect: Z = 1.86 (P = 0.06)						
Test for subgroup differences: $Chi^2 = 4.84$, df = 7 (P = 0.68), $I^2 = 0\%$						

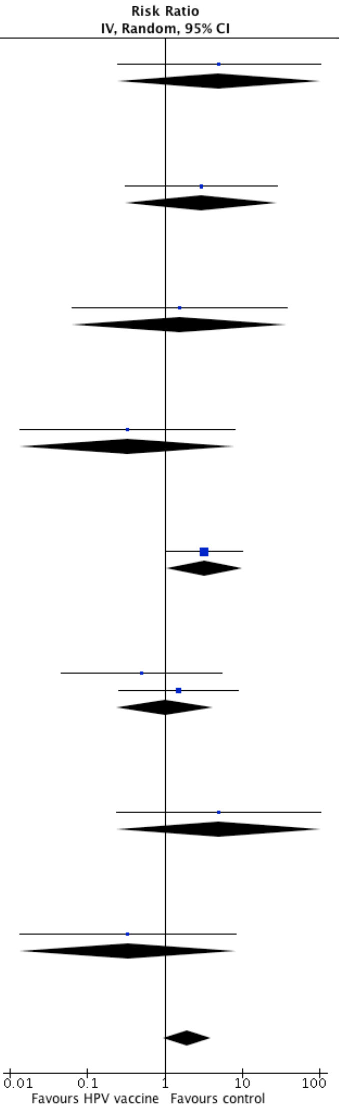

*14.7. Risk ratio for GlaxoSmithKline studies (i.e., HPV-0xx): **2.65 [1.10, 6.38]**; risk ratio for Merck Sharp & Dohme studies (i.e., V50x-xxx): 1.11 [0.33, 3.70]. VigiBase's largest HPV vaccine-associated harm cluster consists of the harms 'headache, nausea, pyrexia, dizziness and vomiting' (1).

- Reported MedDRA terms and number of harms per MedDRA term for the serious harms that were part of VigiBase's largest HPV vaccine-associated harm clusters 'expected systemic reactions':

VigiBase term	MedDRA system organ class	MedDRA preferred term	HPV vaccine	Comparator
Dizziness	Nervous system disorders (10029205)	Dizziness (10013573)	5	2
Headache	Nervous system disorders (10029205)	Headache (10019211)	11	4
Nausea	Gastrointestinal disorders (10017947)	Nausea (10028813)	2	1
Pyrexia	General disorders and administration site conditions (10018065)	Pyrexia (10037660)	4	2
Vomiting	Gastrointestinal disorders (10017947)	Vomiting (10047700)	3	2
**Total**			**25**	**11**

14.8. Serious harms part of VigiBase's 2nd largest HPV vaccine-associated harm cluster* - 'allergic/hypersensitivity reactions': intention to treat analysis

Study or Subgroup	HPV vaccine Events	HPV vaccine Total	Control Events	Control Total	Weight	Risk Ratio IV, Random, 95% CI
**14.8.1 Serious allergic/hypersensitivity reactions : Cervarix vs. Aimmugen: hepatitis A vaccine (females, age 22-29; follow-up until 12 months)**						
HPV-063: Cervarix vs. Aimmugen (f, 22-29; 12)	1	375	0	377	36.0%	3.02 [0.12, 73.80]
**Subtotal (95% CI)**		**375**		**377**	**36.0%**	**3.02 [0.12, 73.80]**
Total events	1		0			
Heterogeneity: Not applicable						
Test for overall effect: Z = 0.68 (P = 0.50)						
**14.8.2 Serious allergic/hypersensitivity reactions : Cervarix vs. Havrix: hepatitis A vaccine (females, age 15-25; follow-up until 48 months)**						
HPV-008: Cervarix vs. Havrix (f, 15-25; 48)	1	9319	2	9325	64.0%	0.50 [0.05, 5.52]
**Subtotal (95% CI)**		**9319**		**9325**	**64.0%**	**0.50 [0.05, 5.52]**
Total events	1		2			
Heterogeneity: Not applicable						
Test for overall effect: Z = 0.57 (P = 0.57)						
**Total (95% CI)**		**9694**		**9702**	**100.0%**	**0.96 [0.14, 6.52]**
Total events	2		2			
Heterogeneity: Tau² = 0.00; Chi² = 0.78, df = 1 (P = 0.38); I² = 0%						
Test for overall effect: Z = 0.05 (P = 0.96)						
Test for subgroup differences: Chi² = 0.78, df = 1 (P = 0.38), I² = 0%						

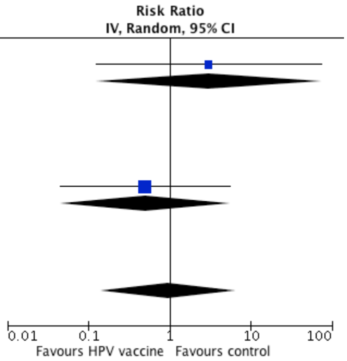

*14.8. Risk ratio for GlaxoSmithKline studies (i.e., HPV-0xx): 0.96 [0.14, 6.52]; risk ratio for Merck Sharp & Dohme studies (i.e., V50x-xxx): not applicable. VigiBase's 2nd largest HPV vaccine-associated harm cluster consists of 'pruritis, urticaria, rash and erythema' (1).

- Reported MedDRA terms and number of harms per MedDRA term for the serious harms that were part of VigiBase's 2nd largest HPV vaccine-associated harm cluster 'allergic/hypersensitivity reactions':

VigiBase term	MedDRA system organ class	MedDRA preferred term	HPV vaccine	Comparator
Erythema	Skin and subcutaneous tissue disorders (10040785)	Erythema	0	0
Pruritus	Skin and subcutaneous tissue disorders (10040785)	Pruritus	0	0
Rash	Skin and subcutaneous tissue disorders (10040785)	Rash	0	0
Urticaria	Skin and subcutaneous tissue disorders (10040785)	Urticaria (10046735)	2	2
**Total**			**2**	**2**

14.9. Serious harms part of VigiBase's 3rd largest HPV vaccine-associated harm cluster* - 'vasovagal reactions': intention to treat analysis

Study or Subgroup	HPV vaccine Events	HPV vaccine Total	Control Events	Control Total	Weight	Risk Ratio IV, Random, 95% CI
**14.9.1 Serious vasovagal reactions: Cervarix vs. Al(OH)3 (females, age 18-35; follow-up until 7 months)**						
HPV-035: Cervarix vs. Al(OH)3 (f, 18-35; 7)	1	150	0	150	9.2%	3.00 [0.12, 73.06]
**Subtotal (95% CI)**		**150**		**150**	**9.2%**	**3.00 [0.12, 73.06]**
Total events	1		0			
Heterogeneity: Not applicable						
Test for overall effect: Z = 0.67 (P = 0.50)						
**14.9.2 Serious vasovagal reactions: Cervarix vs. Al(OH)3 (females, age 24-72; follow-up until 36 months)**						
HPV-015: Cervarix vs. Al(OH)3 (f, 24-72; 36)	0	2882	1	2871	9.2%	0.33 [0.01, 8.15]
**Subtotal (95% CI)**		**2882**		**2871**	**9.2%**	**0.33 [0.01, 8.15]**
Total events	0		1			
Heterogeneity: Not applicable						
Test for overall effect: Z = 0.68 (P = 0.50)						
**14.9.3 Serious vasovagal reactions: Cervarix vs. Engerix: hepatitis B vaccine (females and males, age 12-16; follow-up until 72 months)**						
HPV-040: Cervarix vs. Engerix (f & m, 12-16; 72)	2	2436	0	1267	10.2%	2.60 [0.12, 54.15]
**Subtotal (95% CI)**		**2436**		**1267**	**10.2%**	**2.60 [0.12, 54.15]**
Total events	2		0			
Heterogeneity: Not applicable						
Test for overall effect: Z = 0.62 (P = 0.54)						
**14.9.4 Serious vasovagal reactions: Cervarix vs. Havrix: hepatitis A vaccine (females, age 15-25; follow-up until 48 months)**						
HPV-008: Cervarix vs. Havrix (f, 15-25; 48)	2	9319	2	9325	24.5%	1.00 [0.14, 7.10]
**Subtotal (95% CI)**		**9319**		**9325**	**24.5%**	**1.00 [0.14, 7.10]**
Total events	2		2			
Heterogeneity: Not applicable						
Test for overall effect: Z = 0.00 (P = 1.00)						
**14.9.5 Serious vasovagal reactions: Gardasil vs. AAHS (females, age 15-26; follow-up until 45 months)**						
V501-013: Gardasil vs. AAHS (f, 16-24; 45)	0	2723	2	2732	10.2%	0.20 [0.01, 4.18]
V501-015: Gardasil vs. AAHS (f, 15-26; 36)	1	6087	0	6080	9.2%	3.00 [0.12, 73.54]
**Subtotal (95% CI)**		**8810**		**8812**	**19.4%**	**0.74 [0.05, 10.42]**
Total events	1		2			
Heterogeneity: Tau² = 1.12; Chi² = 1.44, df = 1 (P = 0.23); I² = 31%						
Test for overall effect: Z = 0.22 (P = 0.82)						
**14.9.6 Serious vasovagal reactions: Gardasil vs. AAHS (females, age 21-46; follow-up until 48 months)**						
V501-019: Gardasil vs. AAHS (f, 21-46; 48)	1	1911	0	1908	9.2%	3.00 [0.12, 73.48]
**Subtotal (95% CI)**		**1911**		**1908**	**9.2%**	**3.00 [0.12, 73.48]**
Total events	1		0			
Heterogeneity: Not applicable						
Test for overall effect: Z = 0.67 (P = 0.50)						
**14.9.7 Serious vasovagal reactions: Gardasil 9 vs. saline placebo (females, age 12-26; follow-up until 7 months)**						
V503-006: Gardasil 9 vs. saline (f, 12-26;7)	1	618	0	306	9.2%	1.49 [0.06, 36.42]
**Subtotal (95% CI)**		**618**		**306**	**9.2%**	**1.49 [0.06, 36.42]**
Total events	1		0			
Heterogeneity: Not applicable						
Test for overall effect: Z = 0.24 (P = 0.81)						
**14.9.8 Serious vasovagal reactions: HPV 16 vaccine vs. AAHS (females, age 16-25; follow-up until 48 months)**						
V501-005: HPV 16 vaccine vs. AAHS (f, 16-25; 48)	1	1204	0	1205	9.2%	3.00 [0.12, 73.63]
**Subtotal (95% CI)**		**1204**		**1205**	**9.2%**	**3.00 [0.12, 73.63]**
Total events	1		0			
Heterogeneity: Not applicable						
Test for overall effect: Z = 0.67 (P = 0.50)						
**Total (95% CI)**		**27330**		**25844**	**100.0%**	**1.31 [0.50, 3.46]**
Total events	9		5			
Heterogeneity: Tau² = 0.00; Chi² = 3.48, df = 8 (P = 0.90); I² = 0%						
Test for overall effect: Z = 0.55 (P = 0.58)						
Test for subgroup differences: Chi² = 1.93, df = 7 (P = 0.96), I² = 0%						

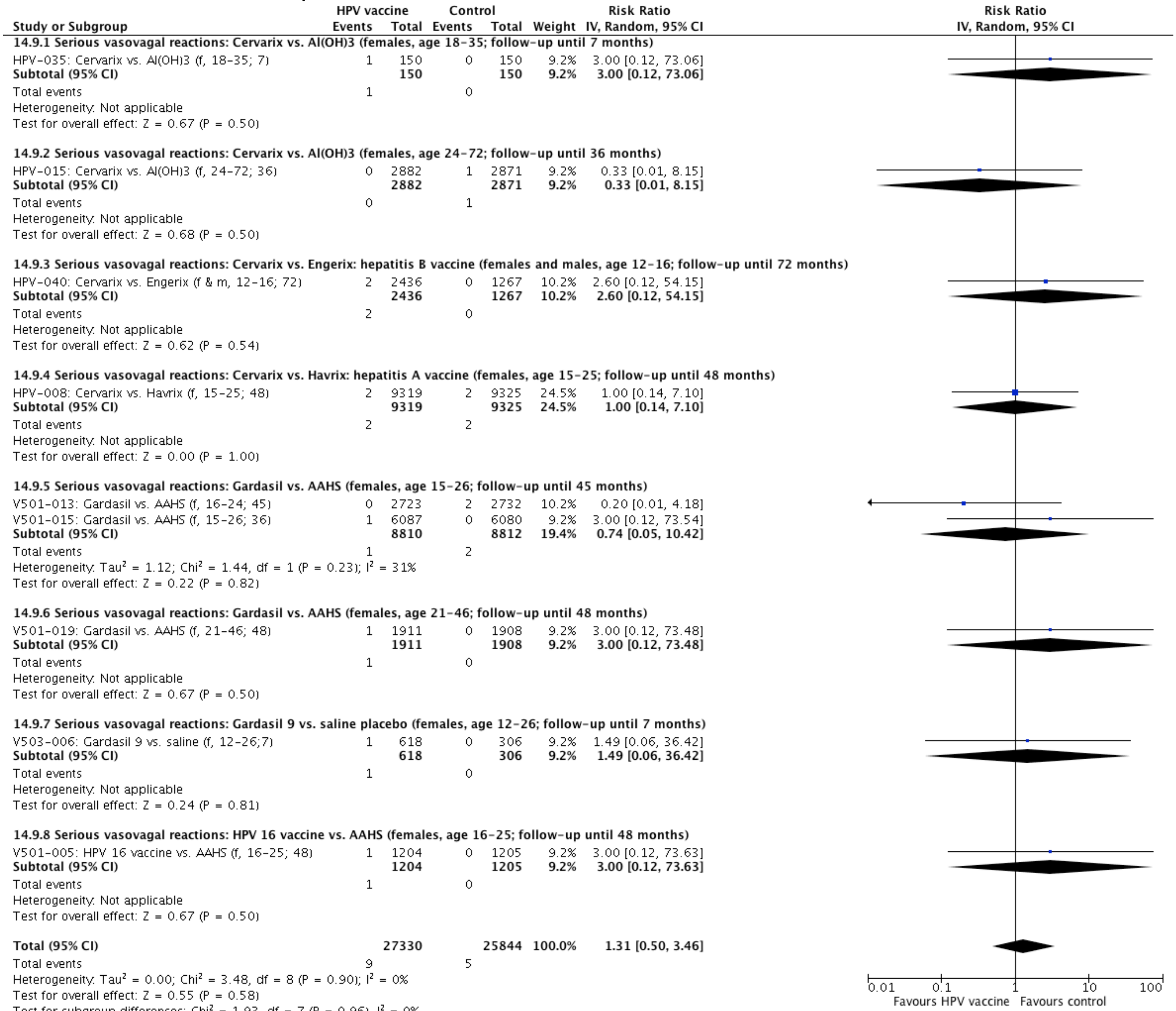

*14.9. Risk ratio for GlaxoSmithKline studies (i.e., HPV-0xx): 1.20 [0.32, 4.55]; risk ratio for Merck Sharp & Dohme studies (i.e., V50x-xxx): 1.45 [0.35, 5.98]. VigiBase's 3rd largest HPV vaccine-associated harm cluster consists of 'syncope, dizziness, loss of consciousness, pallor and seizure' (1).

- Reported MedDRA terms and number of harms per MedDRA term for the serious harms that were part of VigiBase's 3rd largest HPV vaccine-associated harm cluster 'vasovagal reactions':

VigiBase term	MedDRA system organ class	MedDRA term	HPV vaccine	Comparator
Dizziness	Nervous system disorders (10029205)	Dizziness (10013573)	5	2
Loss of consciousness	Nervous system disorders (10029205)	Loss of consciousness	0	0
Pallor	Vascular disorders (10047065)	Pallor	0	0
Seizure	Nervous system disorders (10029205)	Seizure	0	0
Syncope	Nervous system disorders (10029205)	Syncope (10042772)	4	3
**Total**			**9**	**5**

14.10. New onset diseases ('medically significant conditions' and 'new medical history'*) part of VigiBase's largest HPV vaccine-associated harm clusters** - 'expected systemic reactions': intention to treat analysis

Study or Subgroup	HPV vaccine Events	HPV vaccine Total	Control Events	Control Total	Weight	Risk Ratio IV, Random, 95% CI
**14.10.1 New onset expected systemic reactions ('medically significant conditions'): Cervarix vs. Al(OH)3 (females, age 9-17; follow-up until 12 months)**						
HPV-058: Cervarix vs. Al(OH)3 (f, 9-17; 12)	1	374	2	376	0.2%	0.50 [0.05, 5.52]
**Subtotal (95% CI)**		**374**		**376**	**0.2%**	**0.50 [0.05, 5.52]**
Total events	1		2			
Heterogeneity: Not applicable						
Test for overall effect: Z = 0.56 (P = 0.57)						
**14.10.2 New onset expected systemic reactions ('medically significant conditions'): Cervarix vs. Al(OH)3 (females, age 15-25; follow-up until 7 months)**						
HPV-038: Cervarix vs. Al(OH)3 (f, 15-25; 7)	1	149	0	76	0.1%	1.54 [0.06, 37.36]
**Subtotal (95% CI)**		**149**		**76**	**0.1%**	**1.54 [0.06, 37.36]**
Total events	1		0			
Heterogeneity: Not applicable						
Test for overall effect: Z = 0.27 (P = 0.79)						
**14.10.3 New onset expected systemic reactions ('medically significant conditions'): Cervarix vs. Al(OH)3 (females, age 18-35; follow-up until 7 months)**						
HPV-031: Cervarix vs. Al(OH)3 (f, 18-35; 7)	2	176	1	178	0.2%	2.02 [0.19, 22.11]
HPV-035: Cervarix vs. Al(OH)3 (f, 18-35; 7)	6	150	1	150	0.2%	6.00 [0.73, 49.24]
**Subtotal (95% CI)**		**326**		**328**	**0.4%**	**3.73 [0.77, 18.12]**
Total events	8		2			
Heterogeneity: Tau^2 = 0.00; Chi^2 = 0.45, df = 1 (P = 0.50); I^2 = 0%						
Test for overall effect: Z = 1.63 (P = 0.10)						
**14.10.4 New onset expected systemic reactions ('medically significant conditions'): Cervarix vs. Al(OH)3 (females, age 24-72; follow-up until 36 months)**						
HPV-015: Cervarix vs. Al(OH)3 (f, 24-72; 36)	67	2882	41	2871	5.9%	1.63 [1.11, 2.39]
**Subtotal (95% CI)**		**2882**		**2871**	**5.9%**	**1.63 [1.11, 2.39]**
Total events	67		41			
Heterogeneity: Not applicable						
Test for overall effect: Z = 2.48 (P = 0.01)						
**14.10.5 New onset expected systemic reactions ('medically significant conditions'): Cervarix vs. Aimmugen: hepatitis A vaccine (females, age 22-29; follow-up until 12 months)**						
HPV-063: Cervarix vs. Aimmugen (f, 22-29; 12)	3	375	8	377	0.6%	0.38 [0.10, 1.41]
**Subtotal (95% CI)**		**375**		**377**	**0.6%**	**0.38 [0.10, 1.41]**
Total events	3		8			
Heterogeneity: Not applicable						
Test for overall effect: Z = 1.45 (P = 0.15)						
**14.10.6 New onset expected systemic reactions ('medically significant conditions'): Cervarix vs. Engerix: hepatitis B vaccine (females and males, age 9-15; follow-up until 12 months)**						
HPV-030: Cervarix vs. Engerix (f, 9-15; 12)	2	247	2	246	0.3%	1.00 [0.14, 7.01]
**Subtotal (95% CI)**		**247**		**246**	**0.3%**	**1.00 [0.14, 7.01]**
Total events	2		2			
Heterogeneity: Not applicable						
Test for overall effect: Z = 0.00 (P = 1.00)						
**14.10.7 New onset expected systemic reactions ('medically significant conditions'): Cervarix vs. Havrix: hepatitis A vaccine (females, age 9-15; follow-up until 12 months)**						
HPV-013: Cervarix vs. Havrix (f, 10-14; 12)	14	1035	10	1032	1.5%	1.40 [0.62, 3.13]
HPV-033: Cervarix vs. Havrix (f, 9-15; 7)	1	160	0	161	0.1%	3.02 [0.12, 73.55]
**Subtotal (95% CI)**		**1195**		**1193**	**1.6%**	**1.46 [0.67, 3.20]**
Total events	15		10			
Heterogeneity: Tau^2 = 0.00; Chi^2 = 0.21, df = 1 (P = 0.65); I^2 = 0%						
Test for overall effect: Z = 0.95 (P = 0.34)						
**14.10.8 New onset expected systemic reactions ('medically significant conditions'): Cervarix vs. Havrix: hepatitis A vaccine (females, age 15-25; follow-up until 48 months)**						
HPV-008: Cervarix vs. Havrix (f, 15-25; 48)	86	9319	91	9325	9.0%	0.95 [0.71, 1.27]
**Subtotal (95% CI)**		**9319**		**9325**	**9.0%**	**0.95 [0.71, 1.27]**
Total events	86		91			
Heterogeneity: Not applicable						
Test for overall effect: Z = 0.37 (P = 0.71)						
**14.10.9 New onset expected systemic reactions ('medically significant conditions'): Cervarix vs. Twinrix: hepatitis A and B vaccine (females, age 8-15; follow-up until 12 months)**						
HPV-029: Cervarix vs. Twinrix (f, 8-15; 12)	1	270	1	271	0.1%	1.00 [0.06, 15.96]
**Subtotal (95% CI)**		**270**		**271**	**0.1%**	**1.00 [0.06, 15.96]**
Total events	1		1			
Heterogeneity: Not applicable						
Test for overall effect: Z = 0.00 (P = 1.00)						
**14.10.10 New onset expected systemic reactions ('new medical history'): Gardasil vs. carrier solution (females and males, age 9-16; follow-up until 18 months)**						
V501-018: Gardasil vs. carrier solu. (f&m,9-16;18)	132	1184	73	597	10.3%	0.91 [0.70, 1.19]
**Subtotal (95% CI)**		**1184**		**597**	**10.3%**	**0.91 [0.70, 1.19]**
Total events	132		73			
Heterogeneity: Not applicable						
Test for overall effect: Z = 0.67 (P = 0.50)						
**14.10.11 New onset expected systemic reactions ('new medical history'): Gardasil vs. AAHS (females, age 15-26; follow-up until 45 months)**						
V501-013: Gardasil vs. AAHS (f, 16-24; 45)	387	2723	344	2732	21.7%	1.13 [0.99, 1.29]
V501-015: Gardasil vs. AAHS (f, 15-26; 36)	516	6087	527	6080	24.1%	0.98 [0.87, 1.10]
**Subtotal (95% CI)**		**8810**		**8812**	**45.8%**	**1.05 [0.91, 1.20]**
Total events	903		871			
Heterogeneity: Tau^2 = 0.01; Chi^2 = 2.49, df = 1 (P = 0.11); I^2 = 60%						
Test for overall effect: Z = 0.63 (P = 0.53)						
**14.10.12 New onset expected systemic reactions ('new medical history'): Gardasil vs. AAHS (females, age 21-46; follow-up until 48 months)**						
V501-019: Gardasil vs. AAHS (f, 21-46; 48)	132	1911	161	1908	13.3%	0.82 [0.66, 1.02]
**Subtotal (95% CI)**		**1911**		**1908**	**13.3%**	**0.82 [0.66, 1.02]**
Total events	132		161			
Heterogeneity: Not applicable						
Test for overall effect: Z = 1.77 (P = 0.08)						
**14.10.13 New onset expected systemic reactions ('new medical history'): Gardasil vs. AAHS (males, age 15-27; follow-up until 36 months)**						
V501-020: Gardasil vs. AAHS (m, 15-27; 36)	50	2032	37	2033	5.1%	1.35 [0.89, 2.06]
**Subtotal (95% CI)**		**2032**		**2033**	**5.1%**	**1.35 [0.89, 2.06]**
Total events	50		37			
Heterogeneity: Not applicable						
Test for overall effect: Z = 1.41 (P = 0.16)						
**14.10.14 New onset expected systemic reactions ('new medical history'): Gardasil 9 vs. saline placebo (females, age 12-26; follow-up until 7 months)**						
V503-006: Gardasil 9 vs. saline (f, 12-26;7)	15	618	8	306	1.4%	0.93 [0.40, 2.17]
**Subtotal (95% CI)**		**618**		**306**	**1.4%**	**0.93 [0.40, 2.17]**
Total events	15		8			
Heterogeneity: Not applicable						
Test for overall effect: Z = 0.17 (P = 0.86)						
**14.10.15 New onset expected systemic reactions ('new medical history'): HPV 16 vaccine vs. AAHS (females, age 16-25; follow-up until 48 months)**						
V501-005: HPV 16 vaccine vs. AAHS (f, 16-25; 48)	49	1204	51	1205	5.9%	0.96 [0.66, 1.41]
**Subtotal (95% CI)**		**1204**		**1205**	**5.9%**	**0.96 [0.66, 1.41]**
Total events	49		51			
Heterogeneity: Not applicable						
Test for overall effect: Z = 0.20 (P = 0.84)						
**Total (95% CI)**		**30896**		**29924**	**100.0%**	**1.03 [0.93, 1.14]**
Total events	1465		1358			
Heterogeneity: Tau^2 = 0.01; Chi^2 = 21.55, df = 17 (P = 0.20); I^2 = 21%						
Test for overall effect: Z = 0.49 (P = 0.62)						
Test for subgroup differences: Chi^2 = 18.33, df = 14 (P = 0.19), I^2 = 23.6%						

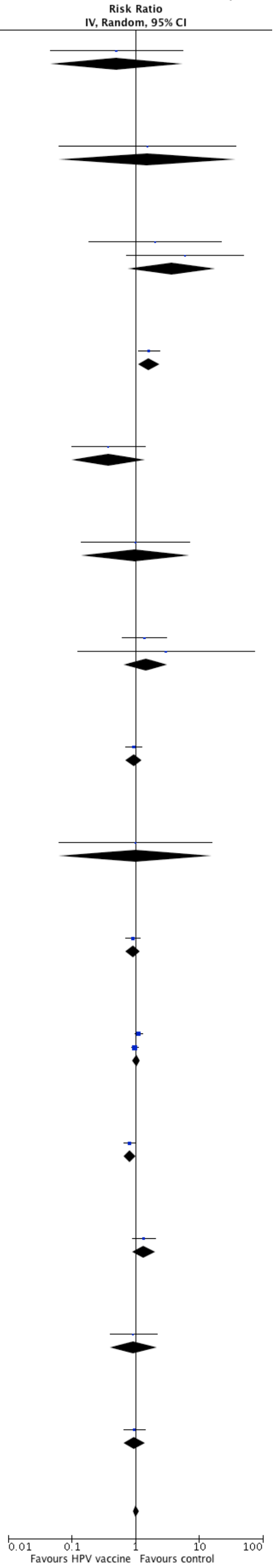

*14.10. Risk ratio for 'medically significant conditions' (GlaxoSmithKline): 1.19 [0.90, 1.57]; risk ratio for 'new medical history' (Merck Sharp & Dohme): 1.00 [0.90, 1.11]. **VigiBase's largest HPV vaccine-associated harm cluster consists of 'headache, nausea, pyrexia, dizziness and vomiting' (1).

- Reported MedDRA terms and number of harms per MedDRA term for the new onset diseases that were part of VigiBase's largest HPV vaccine-associated harm clusters 'expected systemic reactions':

VigiBase term	New onset disease category	MedDRA system organ class	MedDRA preferred term	HPV vaccine	Comparator
Headache	'Medically significant conditions'	Nervous system disorders (10029205)	Headache (10019211)	89	66
	'New medical history'	Nervous system disorders (10029205)	Headache (10019211)	766	690
Nausea	'Medically significant conditions'	Gastrointestinal disorders (10017947)	Nausea (10028813)	21	16
	'New medical history'	Gastrointestinal disorders (10017947)	Nausea (10028813)	147	172
Pyrexia	'Medically significant conditions'	General disorders and administration site conditions (10018065)	Pyrexia (10037660)	29	25
	'New medical history'	General disorders and administration site conditions (10018065)	Pyrexia (10037660)	162	141
Dizziness	'Medically significant conditions'	Nervous system disorders (10029205)	Dizziness (10013573)	30	29
	'New medical history'	Nervous system disorders (10029205)	Dizziness (10013573)	109	102
Vomiting	'Medically significant conditions'	Gastrointestinal disorders (10017947)	Vomiting (10047700)	15	21
	'New medical history'	Gastrointestinal disorders (10017947)	Vomiting (10047700)	97	96
**Total**				**1,465**	**1,358**

14.11. New onset diseases ('medically significant conditions' and 'new medical history'*) part of VigiBase's 2nd largest HPV vaccine-associated harm cluster** - 'allergic/hypersensitivity reactions': intention to treat analysis

Study or Subgroup	HPV vaccine Events	HPV vaccine Total	Control Events	Control Total	Weight	Risk Ratio IV, Random, 95% CI
**14.11.1 New onset allergic/hypersensitivity reactions ('medically significant conditions'): Cervarix vs. Al(OH)3 (females, age 15-25; follow-up until 7 months)**						
HPV-038: Cervarix vs. Al(OH)3 (f, 15-25; 7)	1	149	0	76	0.6%	1.54 [0.06, 37.36]
**Subtotal (95% CI)**		**149**		**76**	**0.6%**	**1.54 [0.06, 37.36]**
Total events	1		0			
Heterogeneity: Not applicable						
Test for overall effect: Z = 0.27 (P = 0.79)						
**14.11.2 New onset allergic/hypersensitivity reactions ('medically significant conditions'): Cervarix vs. Al(OH)3 (females, age 18-35; follow-up until 7 months)**						
HPV-031: Cervarix vs. Al(OH)3 (f, 18-35; 7)	0	176	2	178	0.7%	0.20 [0.01, 4.18]
HPV-035: Cervarix vs. Al(OH)3 (f, 18-35; 7)	3	150	1	150	1.2%	3.00 [0.32, 28.52]
**Subtotal (95% CI)**		**326**		**328**	**1.8%**	**0.95 [0.07, 12.97]**
Total events	3		3			
Heterogeneity: Tau^2 = 1.78; Chi^2 = 1.96, df = 1 (P = 0.16); I^2 = 49%						
Test for overall effect: Z = 0.04 (P = 0.97)						
**14.11.3 New onset allergic/hypersensitivity reactions ('medically significant conditions'): Cervarix vs. Al(OH)3 (females, age 24-72; follow-up until 36 months)**						
HPV-015: Cervarix vs. Al(OH)3 (f, 24-72; 36)	16	2882	19	2871	8.8%	0.84 [0.43, 1.63]
**Subtotal (95% CI)**		**2882**		**2871**	**8.8%**	**0.84 [0.43, 1.63]**
Total events	16		19			
Heterogeneity: Not applicable						
Test for overall effect: Z = 0.52 (P = 0.60)						
**14.11.4 New onset allergic/hypersensitivity reactions ('medically significant conditions'): Cervarix vs. Aimmugen: hepatitis A vaccine (females, age 22-29; follow-up until 12 months)**						
HPV-063: Cervarix vs. Aimmugen (f, 22-29; 12)	6	375	5	377	3.7%	1.21 [0.37, 3.92]
**Subtotal (95% CI)**		**375**		**377**	**3.7%**	**1.21 [0.37, 3.92]**
Total events	6		5			
Heterogeneity: Not applicable						
Test for overall effect: Z = 0.31 (P = 0.75)						
**14.11.5 New onset allergic/hypersensitivity reactions ('medically significant conditions'): Cervarix vs. Engerix: hepatitis B vaccine (females and males, age 9-15; follow-up until 12 months)**						
HPV-030: Cervarix vs. Engerix (f, 9-15; 12)	1	247	1	246	0.8%	1.00 [0.06, 15.83]
**Subtotal (95% CI)**		**247**		**246**	**0.8%**	**1.00 [0.06, 15.83]**
Total events	1		1			
Heterogeneity: Not applicable						
Test for overall effect: Z = 0.00 (P = 1.00)						
**14.11.6 New onset allergic/hypersensitivity reactions ('medically significant conditions'): Cervarix vs. Engerix: hepatitis B vaccine (females, age 26-46; follow-up until 12 months)**						
HPV-069: Cervarix vs. Engerix (f, 26-46; 12)	1	606	0	606	0.6%	3.00 [0.12, 73.50]
**Subtotal (95% CI)**		**606**		**606**	**0.6%**	**3.00 [0.12, 73.50]**
Total events	1		0			
Heterogeneity: Not applicable						
Test for overall effect: Z = 0.67 (P = 0.50)						
**14.11.7 New onset allergic/hypersensitivity reactions ('medically significant conditions'): Cervarix vs. Havrix: hepatitis A vaccine (females, age 9-15; follow-up until 12 months)**						
HPV-013: Cervarix vs. Havrix (f, 10-14; 12)	6	1035	4	1032	3.3%	1.50 [0.42, 5.28]
HPV-033: Cervarix vs. Havrix (f, 9-15; 7)	1	160	0	161	0.6%	3.02 [0.12, 73.55]
**Subtotal (95% CI)**		**1195**		**1193**	**3.9%**	**1.64 [0.51, 5.32]**
Total events	7		4			
Heterogeneity: Tau^2 = 0.00; Chi^2 = 0.16, df = 1 (P = 0.69); I^2 = 0%						
Test for overall effect: Z = 0.83 (P = 0.41)						
**14.11.8 New onset allergic/hypersensitivity reactions ('medically significant conditions'): Cervarix vs. Havrix: hepatitis A vaccine (females, age 15-25; follow-up until 48 months)**						
HPV-008: Cervarix vs. Havrix (f, 15-25; 48)	48	9319	36	9325	13.7%	1.33 [0.87, 2.05]
**Subtotal (95% CI)**		**9319**		**9325**	**13.7%**	**1.33 [0.87, 2.05]**
Total events	48		36			
Heterogeneity: Not applicable						
Test for overall effect: Z = 1.31 (P = 0.19)						
**14.11.9 New onset allergic/hypersensitivity reactions ('medically significant conditions'): Cervarix vs. Twinrix: hepatitis A and B vaccine (females, age 8-15; follow-up until 12 months)**						
HPV-029: Cervarix vs. Twinrix (f, 8-15; 12)	0	270	1	271	0.6%	0.33 [0.01, 8.18]
**Subtotal (95% CI)**		**270**		**271**	**0.6%**	**0.33 [0.01, 8.18]**
Total events	0		1			
Heterogeneity: Not applicable						
Test for overall effect: Z = 0.67 (P = 0.50)						
**14.11.10 New onset allergic/hypersensitivity reactions ('new medical history'): Gardasil vs. carrier solution (females and males, age 9-16; follow-up until 18 months)**						
V501-018: Gardasil vs. carrier solu. (f&m,9-16;18)	19	1184	12	597	8.0%	0.80 [0.39, 1.63]
**Subtotal (95% CI)**		**1184**		**597**	**8.0%**	**0.80 [0.39, 1.63]**
Total events	19		12			
Heterogeneity: Not applicable						
Test for overall effect: Z = 0.62 (P = 0.54)						
**14.11.11 New onset allergic/hypersensitivity reactions ('new medical history'): Gardasil vs. AAHS (females, age 15-26; follow-up until 45 months)**						
V501-013: Gardasil vs. AAHS (f, 16-24; 45)	37	2723	52	2732	14.1%	0.71 [0.47, 1.08]
V501-015: Gardasil vs. AAHS (f, 15-26; 36)	74	6087	103	6080	17.6%	0.72 [0.53, 0.97]
**Subtotal (95% CI)**		**8810**		**8812**	**31.6%**	**0.72 [0.56, 0.91]**
Total events	111		155			
Heterogeneity: Tau^2 = 0.00; Chi^2 = 0.00, df = 1 (P = 0.98); I^2 = 0%						
Test for overall effect: Z = 2.70 (P = 0.007)						
**14.11.12 New onset allergic/hypersensitivity reactions ('new medical history'): Gardasil vs. AAHS (females, age 21-46; follow-up until 48 months)**						
V501-019: Gardasil vs. AAHS (f, 21-46; 48)	26	1911	6	1908	5.9%	4.33 [1.78, 10.49]
**Subtotal (95% CI)**		**1911**		**1908**	**5.9%**	**4.33 [1.78, 10.49]**
Total events	26		6			
Heterogeneity: Not applicable						
Test for overall effect: Z = 3.24 (P = 0.001)						
**14.11.13 New onset allergic/hypersensitivity reactions ('new medical history'): Gardasil vs. AAHS (males, age 15-27; follow-up until 36 months)**						
V501-020: Gardasil vs. AAHS (m, 15-27; 36)	15	2032	10	2033	6.9%	1.50 [0.68, 3.33]
**Subtotal (95% CI)**		**2032**		**2033**	**6.9%**	**1.50 [0.68, 3.33]**
Total events	15		10			
Heterogeneity: Not applicable						
Test for overall effect: Z = 1.00 (P = 0.32)						
**14.11.14 New onset allergic/hypersensitivity reactions ('new medical history'): Gardasil 9 vs. saline placebo (females, age 12-26; follow-up until 7 months)**						
V503-006: Gardasil 9 vs. saline (f, 12-26;7)	4	618	2	306	2.0%	0.99 [0.18, 5.38]
**Subtotal (95% CI)**		**618**		**306**	**2.0%**	**0.99 [0.18, 5.38]**
Total events	4		2			
Heterogeneity: Not applicable						
Test for overall effect: Z = 0.01 (P = 0.99)						
**14.11.15 New onset allergic/hypersensitivity reactions ('new medical history'): HPV 16 vaccine vs. AAHS (females, age 16-25; follow-up until 48 months)**						
V501-005: HPV 16 vaccine vs. AAHS (f, 16-25; 48)	26	1204	25	1205	11.1%	1.04 [0.60, 1.79]
**Subtotal (95% CI)**		**1204**		**1205**	**11.1%**	**1.04 [0.60, 1.79]**
Total events	26		25			
Heterogeneity: Not applicable						
Test for overall effect: Z = 0.14 (P = 0.89)						
**Total (95% CI)**		**31128**		**30154**	**100.0%**	**1.05 [0.82, 1.35]**
Total events	284		279			
Heterogeneity: Tau^2 = 0.07; Chi^2 = 24.77, df = 17 (P = 0.10); I^2 = 31%						
Test for overall effect: Z = 0.40 (P = 0.69)						
Test for subgroup differences: Chi^2 = 22.60, df = 14 (P = 0.07), I^2 = 38.1%						

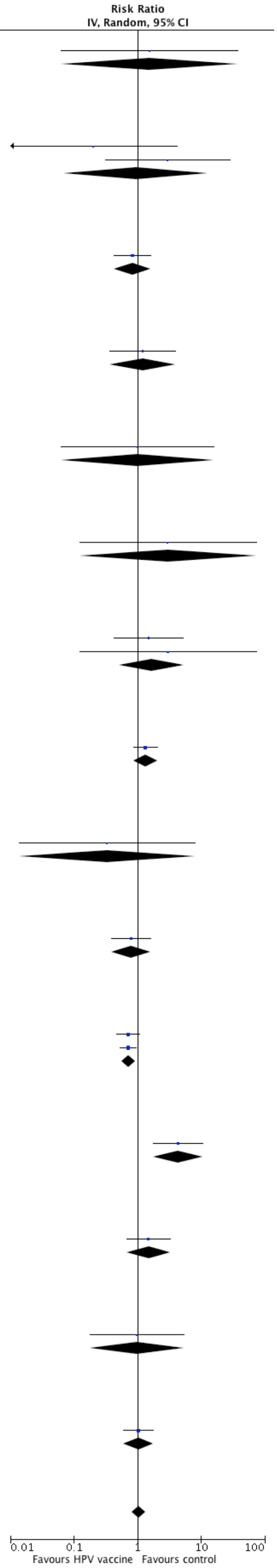

*14.11. Risk ratio for 'medically significant conditions' (GlaxoSmithKline): 1.17 [0.84, 1.64]; risk ratio for 'new medical history' (Merck Sharp & Dohme): 1.19 [0.87, 1.64]. **VigiBase's 2nd largest HPV vaccine-associated harm cluster consists of 'pruritis, urticaria, rash and erythema' (1).

- Reported MedDRA terms and number of harms per MedDRA term for the new onset diseases that were part of VigiBase's 2nd largest HPV vaccine-associated harm cluster 'allergic/hypersensitivity reactions':

VigiBase term	New onset disease category	MedDRA system organ class	MedDRA preferred term	HPV vaccine	Comparator
Pruritus	'Medically significant conditions'	Skin and subcutaneous tissue disorders (10040785)	Pruritus (10037087)	8	5
	'New medical history'	Skin and subcutaneous tissue disorders (10040785)	Pruritus (10037087)	46	37
Urticaria	'Medically significant conditions'	Skin and subcutaneous tissue disorders (10040785)	Urticaria (10046735)	37	28
	'New medical history'	Skin and subcutaneous tissue disorders (10040785)	Urticaria (10046735)	54	57
Rash	'Medically significant conditions'	Skin and subcutaneous tissue disorders (10040785)	Rash (10037844)	38	33
	'New medical history'	Skin and subcutaneous tissue disorders (10040785)	Rash (10037844)	86	108
Erythema	'Medically significant conditions'	Skin and subcutaneous tissue disorders (10040785)	Erythema (10015150)	0	3
	'New medical history'	Skin and subcutaneous tissue disorders (10040785)	Erythema (10015150)	15	8
**Total**				**284**	**279**

14.12. New onset diseases ('medically significant conditions' and 'new medical history'*) part of VigiBase's 3rd largest HPV vaccine-associated harm cluster** - 'vasovagal reactions': intention to treat analysis

Study or Subgroup	HPV vaccine Events	HPV vaccine Total	Control Events	Control Total	Weight	Risk Ratio IV, Random, 95% CI
**14.12.1 New onset vasovagal reactions ('medically significant conditions'): Cervarix vs. Al(OH)3 (females, age 18-35; follow-up until 7 months)**						
HPV-035: Cervarix vs. Al(OH)3 (f, 18-35; 7)	4	150	1	150	1.8%	4.00 [0.45, 35.37]
**Subtotal (95% CI)**		**150**		**150**	**1.8%**	**4.00 [0.45, 35.37]**
Total events	4		1			
Heterogeneity: Not applicable						
Test for overall effect: Z = 1.25 (P = 0.21)						
**14.12.2 New onset vasovagal reactions ('medically significant conditions'): Cervarix vs. Al(OH)3 (females, age 24-72; follow-up until 36 months)**						
HPV-015: Cervarix vs. Al(OH)3 (f, 24-72; 36)	10	2882	21	2871	10.1%	0.47 [0.22, 1.01]
**Subtotal (95% CI)**		**2882**		**2871**	**10.1%**	**0.47 [0.22, 1.01]**
Total events	10		21			
Heterogeneity: Not applicable						
Test for overall effect: Z = 1.95 (P = 0.05)						
**14.12.3 New onset vasovagal reactions ('medically significant conditions'): Cervarix vs. Aimmugen: hepatitis A vaccine (females, age 22-29; follow-up until 12 months)**						
HPV-063: Cervarix vs. Aimmugen (f, 22-29; 12)	2	375	3	377	2.6%	0.67 [0.11, 3.99]
**Subtotal (95% CI)**		**375**		**377**	**2.6%**	**0.67 [0.11, 3.99]**
Total events	2		3			
Heterogeneity: Not applicable						
Test for overall effect: Z = 0.44 (P = 0.66)						
**14.12.4 New onset vasovagal reactions ('medically significant conditions'): Cervarix vs. Engerix: hepatitis B vaccine (females and males, age 9-15; follow-up until 12 months)**						
HPV-030: Cervarix vs. Engerix (f, 9-15; 12)	2	247	0	246	1.0%	4.98 [0.24, 103.20]
**Subtotal (95% CI)**		**247**		**246**	**1.0%**	**4.98 [0.24, 103.20]**
Total events	2		0			
Heterogeneity: Not applicable						
Test for overall effect: Z = 1.04 (P = 0.30)						
**14.12.5 New onset vasovagal reactions ('medically significant conditions'): Cervarix vs. Havrix: hepatitis A vaccine (females, age 10-14; follow-up until 12 months)**						
HPV-013: Cervarix vs. Havrix (f, 10-14; 12)	7	1035	4	1032	5.0%	1.74 [0.51, 5.94]
**Subtotal (95% CI)**		**1035**		**1032**	**5.0%**	**1.74 [0.51, 5.94]**
Total events	7		4			
Heterogeneity: Not applicable						
Test for overall effect: Z = 0.89 (P = 0.37)						
**14.12.6 New onset vasovagal reactions ('medically significant conditions'): Cervarix vs. Havrix: hepatitis A vaccine (females, age 15-25; follow-up until 48 months)**						
HPV-008: Cervarix vs. Havrix (f, 15-25; 48)	39	9319	22	9325	14.8%	1.77 [1.05, 2.99]
**Subtotal (95% CI)**		**9319**		**9325**	**14.8%**	**1.77 [1.05, 2.99]**
Total events	39		22			
Heterogeneity: Not applicable						
Test for overall effect: Z = 2.15 (P = 0.03)						
**14.12.7 New onset vasovagal reactions ('new medical history'): Gardasil vs. carrier solution (females and males, age 9-16; follow-up until 18 months)**						
V501-018: Gardasil vs. carrier solu. (f&m,9-16;18)	10	1184	10	597	8.3%	0.50 [0.21, 1.20]
**Subtotal (95% CI)**		**1184**		**597**	**8.3%**	**0.50 [0.21, 1.20]**
Total events	10		10			
Heterogeneity: Not applicable						
Test for overall effect: Z = 1.54 (P = 0.12)						
**14.12.8 New onset vasovagal reactions ('new medical history'): Gardasil vs. AAHS (females, age 15-26; follow-up until 45 months)**						
V501-013: Gardasil vs. AAHS (f, 16-24; 45)	48	2723	31	2732	16.7%	1.55 [0.99, 2.43]
V501-015: Gardasil vs. AAHS (f, 15-26; 36)	76	6087	86	6080	20.7%	0.88 [0.65, 1.20]
**Subtotal (95% CI)**		**8810**		**8812**	**37.4%**	**1.14 [0.66, 1.98]**
Total events	124		117			
Heterogeneity: Tau² = 0.12; Chi² = 4.16, df = 1 (P = 0.04); I² = 76%						
Test for overall effect: Z = 0.47 (P = 0.64)						
**14.12.9 New onset vasovagal reactions ('new medical history'): Gardasil vs. AAHS (females, age 21-46; follow-up until 48 months)**						
V501-019: Gardasil vs. AAHS (f, 21-46; 48)	30	1911	32	1908	15.5%	0.94 [0.57, 1.53]
**Subtotal (95% CI)**		**1911**		**1908**	**15.5%**	**0.94 [0.57, 1.53]**
Total events	30		32			
Heterogeneity: Not applicable						
Test for overall effect: Z = 0.26 (P = 0.79)						
**14.12.10 New onset vasovagal reactions ('new medical history'): Gardasil vs. AAHS (males, age 15-27; follow-up until 36 months)**						
V501-020: Gardasil vs. AAHS (m, 15-27; 36)	3	2032	2	2033	2.6%	1.50 [0.25, 8.97]
**Subtotal (95% CI)**		**2032**		**2033**	**2.6%**	**1.50 [0.25, 8.97]**
Total events	3		2			
Heterogeneity: Not applicable						
Test for overall effect: Z = 0.44 (P = 0.66)						
**14.12.11 New onset vasovagal reactions ('new medical history'): Gardasil 9 vs. saline placebo (females, age 12-26; follow-up until 7 months)**						
V503-006: Gardasil 9 vs. saline (f, 12-26;7)	1	618	0	306	0.9%	1.49 [0.06, 36.42]
**Subtotal (95% CI)**		**618**		**306**	**0.9%**	**1.49 [0.06, 36.42]**
Total events	1		0			
Heterogeneity: Not applicable						
Test for overall effect: Z = 0.24 (P = 0.81)						
**Total (95% CI)**		**28563**		**27657**	**100.0%**	**1.06 [0.78, 1.44]**
Total events	232		212			
Heterogeneity: Tau² = 0.09; Chi² = 18.83, df = 11 (P = 0.06); I² = 42%						
Test for overall effect: Z = 0.40 (P = 0.69)						
Test for subgroup differences: Chi² = 14.75, df = 10 (P = 0.14), I² = 32.2%						

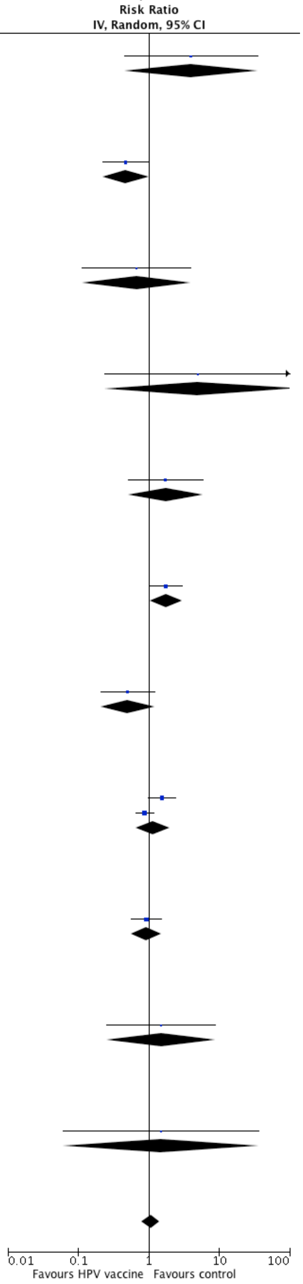

*14.12. Risk ratio for 'medically significant conditions' (GlaxoSmithKline): 1.26 [0.61, 2.58]; risk ratio for 'new medical history' (Merck Sharp & Dohme): 1.00 [0.74, 1.34]. **VigiBase's 3rd largest HPV vaccine-associated harm cluster consists of 'syncope, dizziness, loss of consciousness, pallor and seizure' (1).

- Reported MedDRA terms and number of harms per MedDRA term for the new onset diseases that were part of VigiBase's 3rd largest HPV vaccine-associated harm cluster 'vasovagal reactions':

VigiBase term	New onset disease category	MedDRA system organ class	MedDRA preferred term	HPV vaccine	Comparator
Syncope	'Medically significant conditions'	Nervous system disorders (10029205)	Syncope (10042772)	19	14
	'New medical history'	Nervous system disorders (10029205)	Syncope (10042772)	43	46
Dizziness	'Medically significant conditions'	Nervous system disorders (10029205)	Dizziness (10013573)	30	29
	'New medical history'	Nervous system disorders (10029205)	Dizziness (10013573)	109	102
Loss of consciousness	'Medically significant conditions'	Nervous system disorders (10029205)	Loss of consciousness	1	2
	'New medical history'	Nervous system disorders (10029205)	Loss of consciousness	7	3
Pallor	'Medically significant conditions'	Vascular disorders (10047065)	Pallor	0	0
	'New medical history'	Vascular disorders (10047065)	Pallor	2	3
Seizure	'Medically significant conditions'	Nervous system disorders (10029205)	Convulsion (10010904)	9	6
	'New medical history'	Nervous system disorders (10029205)	Convulsion (10010904)	6	6
	'Medically significant conditions'	Nervous system disorders (10029205)	Generalized seizure	1	0
	'New medical history'	Nervous system disorders (10029205)	Generalized seizure	0	0
	'Medically significant conditions'	Nervous system disorders (10029205)	Grand mal convulsion (10018659)	4	0
	'New medical history'	Nervous system disorders (10029205)	Grand mal convulsion (10018659)	1	1
**Total**				**232**	**212**

14.13. General harms (‘solicited,’ ‘unsolicited’ and ‘systemic adverse events’) part of VigiBase’s largest HPV vaccine-associated harm clusters - ‘expected systemic reactions’: intention to treat analysis

This meta-analysis was inappropriate, as some of the numerators exceeded the denominators making the result nonsensical, but there were more ‘expected systemic reactions’ in the HPV vaccine group:

**VigiBase term**	**General harms category**	**MedDRA system organ class**	**MedDRA preferred term**	**HPV vaccine**	**Comparator**
Headache	‘Solicited’	Nervous system disorders (10029205)	Headache (10019211)	7,621	7,125
	‘Unsolicited’	Nervous system disorders (10029205)	Headache (10019211)	86	53
	‘Systemic adverse events’	Nervous system disorders (10029205)	Headache (10019211)	899	812
Nausea	‘Solicited’	Gastrointestinal disorders (10017947)	Nausea (10028813)	0	0
	‘Unsolicited’	Gastrointestinal disorders (10017947)	Nausea (10028813)	5	3
	‘Systemic adverse events’	Gastrointestinal disorders (10017947)	Nausea (10028813)	208	145
Pyrexia	‘Solicited’	General disorders (10018065)	Pyrexia/fever (10037660)	1,531	1,378
	‘Unsolicited’	General disorders (10018065)	Pyrexia/fever (10037660)	40	35
	‘Systemic adverse events’	General disorders (10018065)	Pyrexia/fever (10037660)	304	234
Dizziness	‘Solicited’	Nervous system disorders (10029205)	Dizziness (10013573)	0	0
	‘Unsolicited’	Nervous system disorders (10029205)	Dizziness (10013573)	37	19
	‘Systemic adverse events’	Nervous system disorders (10029205)	Dizziness (10013573)	127	95
Vomiting	‘Solicited’	Gastrointestinal disorders (10017947)	Vomiting (10047700)	0	0
	‘Unsolicited’	Gastrointestinal disorders (10017947)	Vomiting (10047700)	2	9
	‘Systemic adverse events’	Gastrointestinal disorders (10017947)	Vomiting (10047700)	66	40
**Total**				**10,926**	**9,948**

14.14. General harms (‘solicited,’ ‘unsolicited’ and ‘systemic adverse events’*) part of VigiBase’s 2nd largest HPV vaccine-associated harm cluster** - ‘allergic/hypersensitivity reactions’: intention to treat analysis

Study or Subgroup	HPV vaccine Events	HPV vaccine Total	Control Events	Control Total	Weight	Risk Ratio IV, Random, 95% CI
**14.14.1 General allergic/hypersensitivity reactions ('solicited'): Cervarix vs. Al(OH)3 (females, age 9-17; follow-up until 12 months)**						
HPV-058: Cervarix vs. Al(OH)3 (f, 9-17; 12)	20	374	9	376	1.7%	2.23 [1.03, 4.84]
**Subtotal (95% CI)**		**374**		**376**	**1.7%**	**2.23 [1.03, 4.84]**
Total events	20		9			
Heterogeneity: Not applicable						
Test for overall effect: Z = 2.04 (P = 0.04)						
**14.14.2 General allergic/hypersensitivity reactions ('solicited'): Cervarix vs. Al(OH)3 (females, age 15-26; follow-up until 27 months)**						
HPV-001: Cervarix vs. Al(OH)3 (f, 15-26; 27)	74	560	65	553	8.1%	1.12 [0.82, 1.54]
HPV-038: Cervarix vs. Al(OH)3 (f, 15-25; 7)	54	149	11	76	2.8%	2.50 [1.39, 4.50]
**Subtotal (95% CI)**		**709**		**629**	**10.9%**	**1.61 [0.74, 3.52]**
Total events	128		76			
Heterogeneity: $Tau^2 = 0.26$; $Chi^2 = 5.58$, df = 1 (P = 0.02); $I^2 = 82\%$						
Test for overall effect: Z = 1.20 (P = 0.23)						
**14.14.3 General allergic/hypersensitivity reactions ('solicited'): Cervarix vs. Al(OH)3 (females, age 18-35; follow-up until 12 months)**						
HPV-003: Cervarix vs. Al(OH)3 (f, 18-30; 12)	2	31	0	30	0.1%	4.84 [0.24, 96.89]
HPV-031: Cervarix vs. Al(OH)3 (f, 18-35; 7)	10	176	14	178	1.6%	0.72 [0.33, 1.58]
HPV-035: Cervarix vs. Al(OH)3 (f, 18-35; 7)	18	150	13	150	2.2%	1.38 [0.70, 2.72]
**Subtotal (95% CI)**		**357**		**358**	**3.9%**	**1.10 [0.60, 2.02]**
Total events	30		27			
Heterogeneity: $Tau^2 = 0.06$; $Chi^2 = 2.49$, df = 2 (P = 0.29); $I^2 = 20\%$						
Test for overall effect: Z = 0.31 (P = 0.76)						
**14.14.4 General allergic/hypersensitivity reactions ('solicited'): Cervarix vs. Al(OH)3 (females, age 24-72; follow-up until 36 months)**						
HPV-015: Cervarix vs. Al(OH)3 (f, 24-72; 36)	555	2882	411	2871	22.6%	1.35 [1.20, 1.51]
**Subtotal (95% CI)**		**2882**		**2871**	**22.6%**	**1.35 [1.20, 1.51]**
Total events	555		411			
Heterogeneity: Not applicable						
Test for overall effect: Z = 4.98 (P < 0.00001)						
**14.14.5 General allergic/hypersensitivity reactions ('solicited'): Cervarix vs. Engerix: hepatitis B vaccine (females and males, age 9-16; follow-up until 72 months)**						
HPV-030: Cervarix vs. Engerix (f, 9-15; 12)	24	247	18	246	2.8%	1.33 [0.74, 2.38]
HPV-040: Cervarix vs. Engerix (f & m, 12-16; 72)	37	643	57	1047	5.4%	1.06 [0.71, 1.58]
**Subtotal (95% CI)**		**890**		**1293**	**8.2%**	**1.14 [0.82, 1.58]**
Total events	61		75			
Heterogeneity: $Tau^2 = 0.00$; $Chi^2 = 0.40$, df = 1 (P = 0.53); $I^2 = 0\%$						
Test for overall effect: Z = 0.76 (P = 0.45)						
**14.14.6 General allergic/hypersensitivity reactions ('solicited'): Cervarix vs. Engerix: hepatitis B vaccine (females, age 26-46; follow-up until 12 months)**						
HPV-069: Cervarix vs. Engerix (f, 26-46; 12)	7	606	2	606	0.4%	3.50 [0.73, 16.78]
**Subtotal (95% CI)**		**606**		**606**	**0.4%**	**3.50 [0.73, 16.78]**
Total events	7		2			
Heterogeneity: Not applicable						
Test for overall effect: Z = 1.57 (P = 0.12)						
**14.14.7 General allergic/hypersensitivity reactions ('solicited'): Cervarix vs. Havrix: hepatitis A vaccine (females, age 9-15; follow-up until 12 months)**						
HPV-013: Cervarix vs. Havrix (f, 10-14; 12)	219	1035	145	1032	15.2%	1.51 [1.24, 1.82]
HPV-033: Cervarix vs. Havrix (f, 9-15; 7)	24	160	14	161	2.5%	1.73 [0.93, 3.21]
**Subtotal (95% CI)**		**1195**		**1193**	**17.7%**	**1.52 [1.27, 1.83]**
Total events	243		159			
Heterogeneity: $Tau^2 = 0.00$; $Chi^2 = 0.17$, df = 1 (P = 0.68); $I^2 = 0\%$						
Test for overall effect: Z = 4.51 (P < 0.00001)						
**14.14.8 General allergic/hypersensitivity reactions ('solicited'): Cervarix vs. Havrix: hepatitis A vaccine (females, age 15-25; follow-up until 48 months)**						
HPV-008: Cervarix vs. Havrix (f, 15-25; 48)	787	9319	638	9325	24.5%	1.23 [1.12, 1.36]
**Subtotal (95% CI)**		**9319**		**9325**	**24.5%**	**1.23 [1.12, 1.36]**
Total events	787		638			
Heterogeneity: Not applicable						
Test for overall effect: Z = 4.11 (P < 0.0001)						
**14.14.9 General allergic/hypersensitivity reactions ('solicited'): Cervarix vs. Twinrix: hepatitis A and B vaccine (females, age 8-15; follow-up until 12 months)**						
HPV-029: Cervarix vs. Twinrix (f, 8-15; 12)	37	270	28	271	4.3%	1.33 [0.84, 2.10]
**Subtotal (95% CI)**		**270**		**271**	**4.3%**	**1.33 [0.84, 2.10]**
Total events	37		28			
Heterogeneity: Not applicable						
Test for overall effect: Z = 1.20 (P = 0.23)						
**14.14.10 General allergic/hypersensitivity reactions ('unsolicited'): Cervarix vs. Al(OH)3 (females, age 18-35; follow-up until 7 months)**						
HPV-031: Cervarix vs. Al(OH)3 (f, 18-35; 7)	0	176	1	178	0.1%	0.34 [0.01, 8.22]
HPV-035: Cervarix vs. Al(OH)3 (f, 18-35; 7)	3	150	1	150	0.2%	3.00 [0.32, 28.52]
**Subtotal (95% CI)**		**326**		**328**	**0.3%**	**1.36 [0.17, 10.68]**
Total events	3		2			
Heterogeneity: $Tau^2 = 0.40$; $Chi^2 = 1.20$, df = 1 (P = 0.27); $I^2 = 17\%$						
Test for overall effect: Z = 0.30 (P = 0.77)						
**14.14.11 General allergic/hypersensitivity reactions ('unsolicited'): Cervarix vs. Engerix: hepatitis B vaccine (females and males, age 9-16; follow-up until 72 months)**						
HPV-030: Cervarix vs. Engerix (f, 9-15; 12)	1	247	3	246	0.2%	0.33 [0.03, 3.17]
HPV-040: Cervarix vs. Engerix (f & m, 12-16; 72)	3	1908	4	3128	0.5%	1.23 [0.28, 5.49]
**Subtotal (95% CI)**		**2155**		**3374**	**0.7%**	**0.82 [0.24, 2.87]**
Total events	4		7			
Heterogeneity: $Tau^2 = 0.00$; $Chi^2 = 0.90$, df = 1 (P = 0.34); $I^2 = 0\%$						
Test for overall effect: Z = 0.30 (P = 0.76)						
**14.14.12 General allergic/hypersensitivity reactions ('unsolicited'): Cervarix vs. Havrix: hepatitis A vaccine (females, age 9-15; follow-up until 7 months)**						
HPV-033: Cervarix vs. Havrix (f, 9-15; 7)	1	160	0	161	0.1%	3.02 [0.12, 73.55]
**Subtotal (95% CI)**		**160**		**161**	**0.1%**	**3.02 [0.12, 73.55]**
Total events	1		0			
Heterogeneity: Not applicable						
Test for overall effect: Z = 0.68 (P = 0.50)						
**14.14.13 General allergic/hypersensitivity reactions ('unsolicited'): Cervarix vs. Twinrix: hepatitis A and B vaccine (females, age 8-15; follow-up until 12 months)**						
HPV-029: Cervarix vs. Twinrix (f, 8-15; 12)	1	270	0	271	0.1%	3.01 [0.12, 73.59]
**Subtotal (95% CI)**		**270**		**271**	**0.1%**	**3.01 [0.12, 73.59]**
Total events	1		0			
Heterogeneity: Not applicable						
Test for overall effect: Z = 0.68 (P = 0.50)						
**14.14.14 General allergic/hypersensitivity reactions ('systemic adverse events'): Gardasil vs. carrier solution (females and males, age 9-16; follow-up until 18 months)**						
V501-018: Gardasil vs. carrier solu. (f&m,9-16;18)	8	1184	9	597	1.1%	0.45 [0.17, 1.16]
**Subtotal (95% CI)**		**1184**		**597**	**1.1%**	**0.45 [0.17, 1.16]**
Total events	8		9			
Heterogeneity: Not applicable						
Test for overall effect: Z = 1.66 (P = 0.10)						
**14.14.15 General allergic/hypersensitivity reactions ('systemic adverse events'): Gardasil vs. AAHS (males, age 15-27; follow-up until 36 months)**						
V501-020: Gardasil vs. AAHS (m, 15-27; 36)	6	2032	7	2033	0.9%	0.86 [0.29, 2.55]
**Subtotal (95% CI)**		**2032**		**2033**	**0.9%**	**0.86 [0.29, 2.55]**
Total events	6		7			
Heterogeneity: Not applicable						
Test for overall effect: Z = 0.28 (P = 0.78)						
**14.14.16 General allergic/hypersensitivity reactions ('systemic adverse events'): Gardasil 9 vs. saline placebo (females, age 12-26; follow-up until 7 months)**						
V503-006: Gardasil 9 vs. saline (f, 12-26;7)	3	618	4	306	0.5%	0.37 [0.08, 1.65]
**Subtotal (95% CI)**		**618**		**306**	**0.5%**	**0.37 [0.08, 1.65]**
Total events	3		4			
Heterogeneity: Not applicable						
Test for overall effect: Z = 1.30 (P = 0.19)						
**14.14.17 General allergic/hypersensitivity reactions ('systemic adverse events'): HPV 16 vaccine vs. AAHS (females, age 16-25; follow-up until 48 months)**						
V501-005: HPV 16 vaccine vs. AAHS (f, 16-25; 48)	18	1204	15	1205	2.1%	1.20 [0.61, 2.37]
**Subtotal (95% CI)**		**1204**		**1205**	**2.1%**	**1.20 [0.61, 2.37]**
Total events	18		15			
Heterogeneity: Not applicable						
Test for overall effect: Z = 0.53 (P = 0.60)						
**Total (95% CI)**		**24551**		**25197**	**100.0%**	**1.30 [1.18, 1.45]**
Total events	1912		1469			
Heterogeneity: $Tau^2 = 0.01$; $Chi^2 = 28.77$, df = 23 (P = 0.19); $I^2 = 20\%$						
Test for overall effect: Z = 5.07 (P < 0.00001)						
Test for subgroup differences: $Chi^2 = 18.11$, df = 16 (P = 0.32), $I^2 = 11.7\%$						

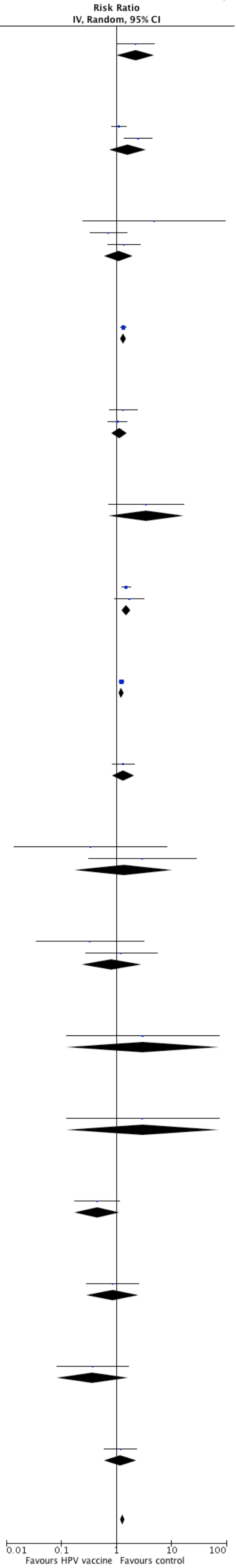

*14.14. Risk ratio for ‘solicited’ (GlaxoSmithKline): **1.34 [1.21, 1.48]**; risk ratio for ‘unsolicited’ (GlaxoSmithKline): 1.19 [0.47, 3.06]; risk ratio for ‘systemic adverse events’ (Merck Sharp & Dohme): 0.75 [0.43, 1.30]. **VigiBase’s 2nd largest HPV vaccine-associated harm cluster consists of ‘pruritis, urticaria, rash and erythema’ (1).

- Reported MedDRA terms and number of harms per MedDRA term for the general harms that were part of VigiBase's 2nd largest HPV vaccine-associated harm cluster 'allergic/hypersensitivity reactions':

VigiBase term	General harms category	MedDRA system organ class	MedDRA preferred term	HPV vaccine	Comparator
Pruritis	'Solicited'	Skin and subcutaneous tissue disorders (10040785)	Pruritis	0	0
	'Unsolicited'	Skin and subcutaneous tissue disorders (10040785)	Pruritis	0	0
	'Systemic adverse events'	Skin and subcutaneous tissue disorders (10040785)	Pruritis	0	0
Urticaria	'Solicited'	Skin and subcutaneous tissue disorders (10040785)	Urticaria	888	712
	'Unsolicited'	Skin and subcutaneous tissue disorders (10040785)	Urticaria	9	5
	'Systemic adverse events'	Skin and subcutaneous tissue disorders (10040785)	Urticaria	8	7
Rash	'Solicited'	Skin and subcutaneous tissue disorders (10040785)	Rash (10037844)	980	713
	'Unsolicited'	Skin and subcutaneous tissue disorders (10040785)	Rash (10037844)	9	7
	'Systemic adverse events'	Skin and subcutaneous tissue disorders (10040785)	Rash (10037844)	16	22
Erythema	'Solicited'	Skin and subcutaneous tissue disorders (10040785)	Erythema (10015150)	0	0
	'Unsolicited'	Skin and subcutaneous tissue disorders (10040785)	Erythema (10015150)	0	2
	'Systemic adverse events'	Skin and subcutaneous tissue disorders (10040785)	Erythema (10015150)	2	1
**Total**				**1,912**	**1,469**

14.15. General harms (‘solicited,’ ‘unsolicited’ and ‘systemic adverse events’*) part of VigiBase’s 3rd largest HPV vaccine-associated harm cluster** - ‘vasovagal reactions’: intention to treat analysis

Study or Subgroup	HPV vaccine Events	HPV vaccine Total	Control Events	Control Total	Weight	Risk Ratio IV, Random, 95% CI
**14.15.1 General vasovagal reactions ('unsolicited'): Cervarix vs. Al(OH)3 (females, age 9-17; follow-up until 12 months)**						
HPV-058: Cervarix vs. Al(OH)3 (f, 9-17; 12)	0	374	1	376	0.6%	0.34 [0.01, 8.20]
**Subtotal (95% CI)**		**374**		**376**	**0.6%**	**0.34 [0.01, 8.20]**
Total events	0		1			
Heterogeneity: Not applicable						
Test for overall effect: Z = 0.67 (P = 0.50)						
**14.15.2 General vasovagal reactions ('unsolicited'): Cervarix vs. Al(OH)3 (females, age 15-25; follow-up until 7 months)**						
HPV-038: Cervarix vs. Al(OH)3 (f, 15-25; 7)	1	149	1	76	0.9%	0.51 [0.03, 8.04]
**Subtotal (95% CI)**		**149**		**76**	**0.9%**	**0.51 [0.03, 8.04]**
Total events	1		1			
Heterogeneity: Not applicable						
Test for overall effect: Z = 0.48 (P = 0.63)						
**14.15.3 General vasovagal reactions ('unsolicited'): Cervarix vs. Al(OH)3 (females, age 18-35; follow-up until 12 months)**						
HPV-003: Cervarix vs. Al(OH)3 (f, 18-30; 12)	4	31	0	30	0.8%	8.72 [0.49, 155.27]
HPV-031: Cervarix vs. Al(OH)3 (f, 18-35; 7)	3	176	1	178	1.3%	3.03 [0.32, 28.89]
HPV-035: Cervarix vs. Al(OH)3 (f, 18-35; 7)	11	150	6	150	6.7%	1.83 [0.70, 4.83]
**Subtotal (95% CI)**		**357**		**358**	**8.8%**	**2.26 [0.96, 5.28]**
Total events	18		7			
Heterogeneity: Tau² = 0.00; Chi² = 1.09, df = 2 (P = 0.58); I² = 0%						
Test for overall effect: Z = 1.88 (P = 0.06)						
**14.15.4 General vasovagal reactions ('unsolicited'): Cervarix vs. Engerix: hepatitis B vaccine (females and males, age 9-16; follow-up until 72 months)**						
HPV-030: Cervarix vs. Engerix (f, 9-15; 12)	7	247	4	246	4.3%	1.74 [0.52, 5.88]
HPV-040: Cervarix vs. Engerix (f & m, 12-16; 72)	6	1908	5	3128	4.5%	1.97 [0.60, 6.44]
**Subtotal (95% CI)**		**2155**		**3374**	**8.9%**	**1.85 [0.79, 4.33]**
Total events	13		9			
Heterogeneity: Tau² = 0.00; Chi² = 0.02, df = 1 (P = 0.89); I² = 0%						
Test for overall effect: Z = 1.43 (P = 0.15)						
**14.15.5 General vasovagal reactions ('unsolicited'): Cervarix vs. Havrix: hepatitis A vaccine (females, age 9-15; follow-up until 7 months)**						
HPV-033: Cervarix vs. Havrix (f, 9-15; 7)	5	160	3	161	3.2%	1.68 [0.41, 6.90]
**Subtotal (95% CI)**		**160**		**161**	**3.2%**	**1.68 [0.41, 6.90]**
Total events	5		3			
Heterogeneity: Not applicable						
Test for overall effect: Z = 0.72 (P = 0.47)						
**14.15.6 General vasovagal reactions ('unsolicited'): Cervarix vs. Twinrix: hepatitis A and B vaccine (females, age 8-15; follow-up until 12 months)**						
HPV-029: Cervarix vs. Twinrix (f, 8-15; 12)	3	270	2	271	2.0%	1.51 [0.25, 8.94]
**Subtotal (95% CI)**		**270**		**271**	**2.0%**	**1.51 [0.25, 8.94]**
Total events	3		2			
Heterogeneity: Not applicable						
Test for overall effect: Z = 0.45 (P = 0.65)						
**14.15.7 General vasovagal reactions ('systemic adverse events'): Gardasil vs. carrier solution (females and males, age 9-16; follow-up until 18 months)**						
V501-018: Gardasil vs. carrier solu. (f&m,9-16;18)	26	1184	12	597	13.1%	1.09 [0.56, 2.15]
**Subtotal (95% CI)**		**1184**		**597**	**13.1%**	**1.09 [0.56, 2.15]**
Total events	26		12			
Heterogeneity: Not applicable						
Test for overall effect: Z = 0.26 (P = 0.80)						
**14.15.8 General vasovagal reactions ('systemic adverse events'): Gardasil vs. AAHS (males, age 15-27; follow-up until 36 months)**						
V501-020: Gardasil vs. AAHS (m, 15-27; 36)	21	2032	20	2033	15.9%	1.05 [0.57, 1.93]
**Subtotal (95% CI)**		**2032**		**2033**	**15.9%**	**1.05 [0.57, 1.93]**
Total events	21		20			
Heterogeneity: Not applicable						
Test for overall effect: Z = 0.16 (P = 0.87)						
**14.15.9 General vasovagal reactions ('systemic adverse events'): Gardasil 9 vs. saline placebo (females, age 12-26; follow-up until 7 months)**						
V503-006: Gardasil 9 vs. saline (f, 12-26;7)	33	618	6	306	8.4%	2.72 [1.15, 6.43]
**Subtotal (95% CI)**		**618**		**306**	**8.4%**	**2.72 [1.15, 6.43]**
Total events	33		6			
Heterogeneity: Not applicable						
Test for overall effect: Z = 2.29 (P = 0.02)						
**14.15.10 General vasovagal reactions ('systemic adverse events'): HPV 16 vaccine vs. AAHS (females, age 16-25; follow-up until 48 months)**						
V501-005: HPV 16 vaccine vs. AAHS (f, 16-25; 48)	53	1204	62	1205	38.2%	0.86 [0.60, 1.22]
**Subtotal (95% CI)**		**1204**		**1205**	**38.2%**	**0.86 [0.60, 1.22]**
Total events	53		62			
Heterogeneity: Not applicable						
Test for overall effect: Z = 0.85 (P = 0.39)						
**Total (95% CI)**		**8503**		**8757**	**100.0%**	**1.20 [0.93, 1.55]**
Total events	173		123			
Heterogeneity: Tau² = 0.01; Chi² = 12.60, df = 12 (P = 0.40); I² = 5%						
Test for overall effect: Z = 1.40 (P = 0.16)						
Test for subgroup differences: Chi² = 11.49, df = 9 (P = 0.24), I² = 21.7%						

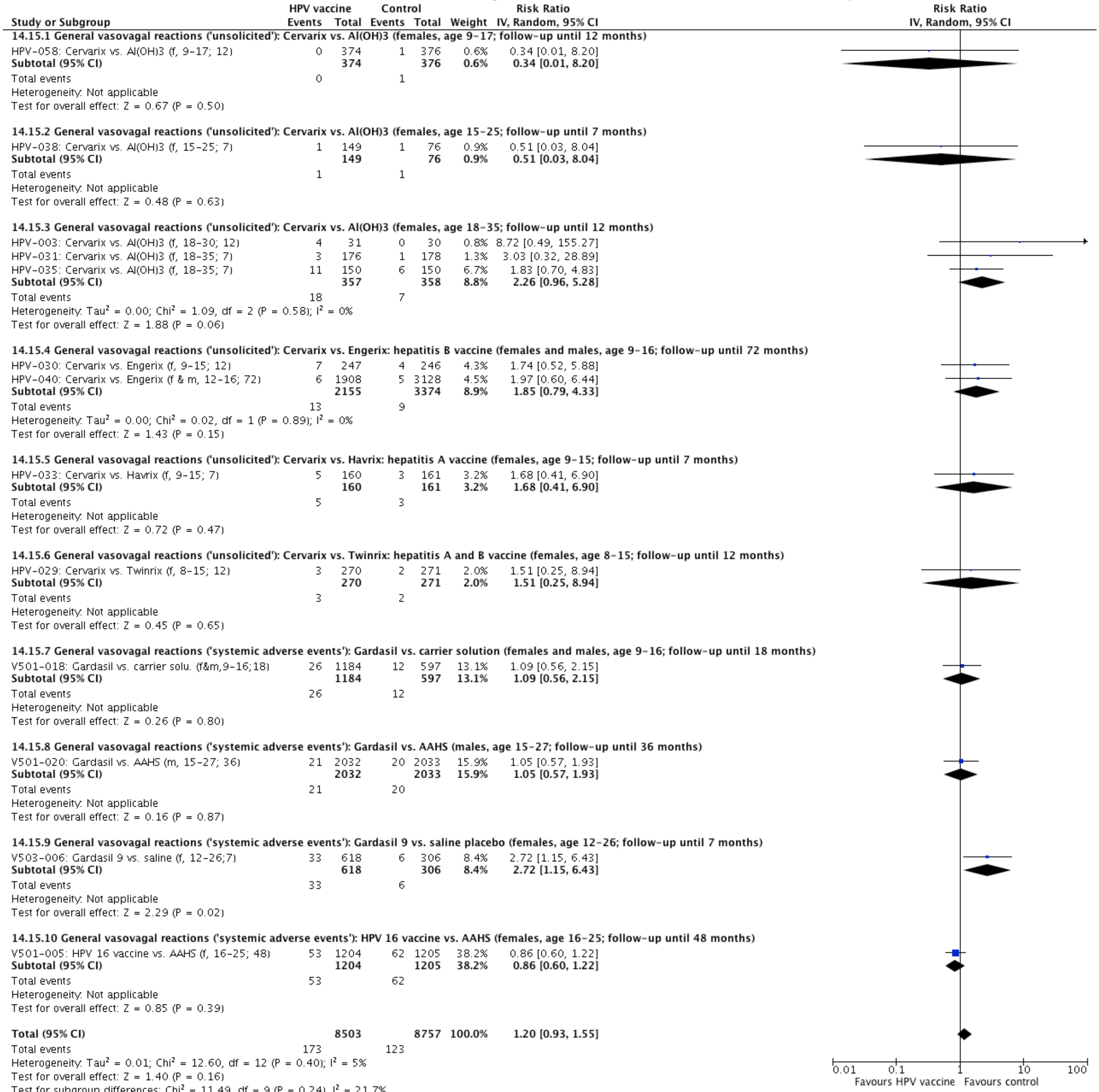

*14.15. Risk ratio for ‘solicited’ (GlaxoSmithKline): not applicable; risk ratio for ‘unsolicited’ (GlaxoSmithKline): **1.77 [1.06, 2.95]**; risk ratio for ‘systemic adverse events’ (Merck Sharp & Dohme): 1.15 [0.75, 1.74]. **VigiBase’s 3rd largest HPV vaccine-associated harm cluster consists of ‘syncope, dizziness, loss of consciousness, pallor and seizure’ (1).

- Reported MedDRA terms and number of harms per MedDRA term for the general harms that were part of VigiBase's 3rd largest HPV vaccine-associated harm cluster 'vasovagal reactions':

VigiBase term	General harms category	MedDRA system organ class	MedDRA preferred term	HPV vaccine	Comparator
Syncope	'Solicited'	Nervous system disorders (10029205)	Syncope (10042772)	0	0
	'Unsolicited'	Nervous system disorders (10029205)	Syncope (10042772)	3	4
	'Systemic adverse events'	Nervous system disorders (10029205)	Syncope (10042772)	4	3
Dizziness	'Solicited'	Nervous system disorders (10029205)	Dizziness (10013573)	0	0
	'Unsolicited'	Nervous system disorders (10029205)	Dizziness (10013573)	37	19
	'Systemic adverse events'	Nervous system disorders (10029205)	Dizziness (10013573)	127	95
Loss of consciousness	'Solicited'	Nervous system disorders (10029205)	Pyrexia/fever (10037660)	0	0
	'Unsolicited'	Nervous system disorders (10029205)	Pyrexia/fever (10037660)	0	0
	'Systemic adverse events'	Nervous system disorders (10029205)	Pyrexia/fever (10037660)	0	0
Seizure	'Solicited'	Nervous system disorders (10029205)	Seizure	0	0
	'Unsolicited'	Nervous system disorders (10029205)	Seizure	0	0
	'Systemic adverse events'	Nervous system disorders (10029205)	Convulsion (10010904)	1	0
Pallor	'Solicited'	Vascular disorders (10047065)	Pallor	0	0
	'Unsolicited'	Vascular disorders (10047065)	Pallor	0	0
	'Systemic adverse events'	Vascular disorders (10047065)	Pallor	1	2
**Total**				**173**	**123**

15. Definitions of harms categories

- *Serious harms*
    - GlaxoSmithKline: “any untoward medical occurrence that: a. resulted in death, b. was life-threatening, NOTE: The term 'life-threatening' in the definition of 'serious' refers to an event in which the subject was at risk of death at the time of the event. It did not refer to an event, which hypothetically might have caused death, if it were more severe. c. required hospitalization or prolongation of existing hospitalization, NOTE: In general, hospitalization signified that the subject had been detained (usually involving at least an overnight stay) at the hospital or emergency ward for observation and/or treatment that would not have been appropriate in the physician's office or out-patient setting. Complications that occurred during hospitalization were AEs [adverse events]. If a complication prolonged hospitalization or fulfilled any other serious criteria, the event was serious. When in doubt as to whether "hospitalization" occurred or was necessary, the AE was to be considered serious. Hospitalization for elective treatment of a pre-existing condition that did not worsen from baseline was not considered an AE. d. resulted in disability/incapacity, NOTE: The term disability means a substantial disruption of a person's ability to conduct normal life functions. This definition was not intended to include experiences of relatively minor medical significance such as uncomplicated headache, nausea, vomiting, diarrhoea, influenza, and accidental trauma (e.g. sprained ankle) which may interfere or prevent everyday life functions but did not constitute a substantial disruption. e. was a congenital anomaly/birth defect in the offspring of a study subject.”
    - Merck Sharp & Dohme: “A serious adverse experience is any adverse experience occurring at any dose that: Results in death; or that is life threatening (places the subject/patient, in the view of the investigator, at immediate risk of death from the experience as it occurred. [Note: This does not include an adverse experience that, had it occurred in a more severe form, might have caused death.]); or that results in a persistent or significant disability/incapacity (substantial disruption of one's ability to conduct normal life functions); or that results in or prolongs an existing inpatient hospitalization (hospitalized is defined as an inpatient admission, regardless of length of stay, even if the hospitalization is a precautionary measure for continued observation.) (Note: Hospitalization [including hospitalization for an elective procedure] for a pre-existing condition which has not worsened does not constitute a serious adverse experience); or that is a congenital anomaly/birth defect (in offspring of subject taking the product regardless of time to diagnosis); or ALSO: Other important medical events that may not result in death, not be life threatening, or not require hospitalization may be considered a serious adverse experience when, based upon appropriate medical judgment, the event may jeopardize the subject/patient and may require medical or surgical intervention to prevent one of the (t) outcomes listed above. In addition, Merck Sharp & Dohme & Co., Inc. requires the collection of the following: cancer, or overdose (whether accidental or intentional).”
- *New onset diseases*
    - ‘Medically significant conditions’ (GlaxoSmithKline): “Adverse events prompting emergency room or physician visits that are not (1) related to common diseases or (2) routine visits for physical examination or vaccination, or SAEs that are not related to common diseases. Serious adverse events related to common diseases were reported but are not classified as medically significant conditions for analysis purposes. Common diseases include: upper respiratory infections, sinusitis, pharyngitis, gastroenteritis, urinary tract infections, cervicovaginal yeast infections, menstrual cycle abnormalities and injury.”
    - ‘New medical history’ (Merck Sharp & Dohme): Merck Sharp & Dohme did not provide a formal definition for 'new medical history' but described 'new medical history' as “all new reported diagnoses” in the clinical study report of trial V501-019.
- *General harms*
    - ‘Solicited’ general adverse events (GlaxoSmithKline): “Adverse events to be recorded as endpoints in the clinical study. The presence/occurrence/intensity of these events is actively solicited from the subject or an observer during a specified post-vaccination follow-up period.”
    - ‘Unsolicited’ general adverse event (GlaxoSmithKline): “Any AE [adverse event] reported in addition to those solicited during the clinical study. Also, any "solicited" symptom with onset

outside the specified period of follow-up for solicited symptoms was reported as an unsolicited AE."

- 'Systemic adverse event' (Merck Sharp & Dohme): "…any systemic clinical adverse event that developed on the day of vaccination or during the 14 days after vaccination was recorded on the VRC [vaccination report card] along with the date it started and the last date it was present."

16. References

1. Chandler RE, Juhlin K, Fransson J, Caster O, Edwards IR, Norén GN. Current Safety Concerns with Human Papillomavirus Vaccine: A Cluster Analysis of Reports in VigiBase(®). *Drug Saf*. 2017 Jan;40(1):81–90.
